# Computational identification of ultra-conserved elements in the human genome: a hypothesis on homologous DNA pairing

**DOI:** 10.1093/nargab/lqae074

**Published:** 2024-07-02

**Authors:** Emily R Crossley, Larisa Fedorova, Oleh A Mulyar, Ryan Freeman, Sadik Khuder, Alexei Fedorov

**Affiliations:** Program of Bioinformatics and Proteomics/Genomics, University of Toledo, Toledo, OH 43606, USA; CRI Genetics LLC, Santa Monica, CA 90404, USA; CRI Genetics LLC, Santa Monica, CA 90404, USA; CRI Genetics LLC, Santa Monica, CA 90404, USA; Program of Bioinformatics and Proteomics/Genomics, University of Toledo, Toledo, OH 43606, USA; Department of Medicine, University of Toledo, Toledo, OH 43606, USA; Program of Bioinformatics and Proteomics/Genomics, University of Toledo, Toledo, OH 43606, USA; CRI Genetics LLC, Santa Monica, CA 90404, USA; Department of Medicine, University of Toledo, Toledo, OH 43606, USA

## Abstract

Thousands of prolonged sequences of human ultra-conserved non-coding elements (UCNEs) share only one common feature: peculiarities in the unique composition of their dinucleotides. Here we investigate whether the numerous weak signals emanating from these dinucleotide arrangements can be used for computational identification of UCNEs within the human genome. For this purpose, we analyzed 4272 UCNE sequences, encompassing 1 393 448 nucleotides, alongside equally sized control samples of randomly selected human genomic sequences. Our research identified nine different features of dinucleotide arrangements that enable differentiation of UCNEs from the rest of the genome. We employed these nine features, implementing three Machine Learning techniques – Support Vector Machine, Random Forest, and Artificial Neural Networks – to classify UCNEs, achieving an accuracy rate of 82–84%, with specific conditions allowing for over 90% accuracy. Notably, the strongest feature for UCNE identification was the frequency ratio between GpC dinucleotides and the sum of GpG and CpC dinucleotides. Additionally, we investigated the entire pool of 31 046 SNPs located within UCNEs for their representation in the ClinVar database, which catalogs human SNPs with known phenotypic effects. The presence of UCNE-associated SNPs in ClinVar aligns with the expectation of a random distribution, emphasizing the enigmatic nature of UCNE phenotypic manifestation.

## Introduction

Ultra-conserved non-coding elements (UCNEs or UCEs) are widespread in the genomes of all mammals and other vertebrates. Discovered between 2002 and 2004, they captured the immediate attention of the scientific community (1,2). Yet, the mystery of why these long non-coding DNA fragments have remained unchanged for hundreds of millions of years persists. Theodosius Dobzhansky's famous assertion, ‘Nothing makes sense in Biology except in the light of Evolution’ (3), appears to be paradoxically inapplicable to UCNEs; their biological functions continue to elude us. Several population studies suggest that UCNEs should be under strong selection pressure (4–7). This selection pressure contradicts the observation that the vast majority of mutations inside UCNE sequences do not show any phenotypic effects (5,8) (see also our results in this paper). There are several hypotheses about the possible functional roles of UCNEs, including their presence in non-coding RNAs (9,10) and how they may act as enhancers that regulate gene transcription (11). However, less than 20% of UCNE sequences are present inside the entire pool of non-coding RNAs (ncRNAs), and the intersection between datasets of UCNE and ncRNA sequences remains at the level of random occurrence (12). In a recent review, Snetkova *et al.* (11) thoroughly examined UCNE’s potential enhancer roles. The authors emphasized the inexplicability of uninterrupted sequence conservation in two hundred nucleotide-long UCNEs. They concluded that ‘ultraconservation is likely to be maintained by multiple forces’. In addition, McCole and co-authors suggested that ultraconserved elements may occupy specific arenas of 3D mammalian genome organization (13).

There are several definitions of UCNE sequences, and their count depends on a particular set of rules for their characterization. Our research uses the UCNEbase database, which classifies a DNA fragment as a UCNE if it is at least 200 bp long and shares 95% identity between humans and chickens (14). This database contains 4272 elements, with an average UCNE size of about 300 nucleotides. An alternative definition of UCNEs requires sequences to exceed 100 nucleotides with complete identity across several mammals (15). All in all, UCNEs are widespread throughout all chromosomes and are most frequently located inside intergenic regions or within large introns. Remarkably, UCNEs share no nucleotide sequence similarity with each other and lack significant enrichment of any oligonucleotides (≥10 bases long) that may serve as common functional motifs for enhancers or other DNA regulatory elements (12).

Recently, our team demonstrated that the only common nucleotide similarity across UCNE sequences is their unique dinucleotide composition (12,16). In general, UCNEs are GC-poor nucleotide sequences that are strongly enriched with GpC dinucleotides and deficient in GpG and CpC dinucleotides. Because dinucleotides (also frequently referred to as ‘nearest neighbor doublets’ (17)) are the most critical elements for different DNA conformations, we are convinced that the key to the cryptic properties of UCNEs is hidden in their specific DNA conformations. In this paper, we examine the prediction power of dinucleotide arrangements for computational differentiation of UCNEs from whole genome sequences. At the end, we propose our hypothesis that specific, non-canonical DNA conformation of UCNEs may be integral to the homologous pairing of double-stranded DNAs during meiosis.

## Materials and methods

### Databases

We used our purified set of 4272 UCNEs sequences described and available from Fedorova *et al.* (12). This set was created from the human UCNEbase database (14) (https://epd.expasy.org/ucnebase/). Since no one has ever found any specific orientation in UCNE sequences (where is the beginning vs. the end of a UCNE?), we processed only the reference positive strand of all UCNE sequences. For distances between dinucleotides, we used the measurement scheme from our previous paper by Fedorova *et al.* (16). The minimal distance corresponds to the shortest distance between intersected dinucleotides. Intersected dinucleotides are pairs of nucleotides that share one nucleotide. For instance, in the triplet ATG, AT and TG are considered intersected dinucleotides. In this example, the distance between AT and TG in ATG is L = 1 nucleotide.

Human genome sequence with masked repetitive elements (shown in lower case letters) was downloaded from https://hgdownload.soe.ucsc.edu/downloads.html UCSC genome browser as an assembly of the human genome (hg38, GRCh38 Genome Reference Consortium Human Reference 38, accession: GCA_000001405.15), accessed on 20 March 2024. This whole genome was used to create randomly selected DNA fragments (Whole Genome Elements, WGE) and unique (non-repetitive) Whole Genome Elements (uWGE). Sequences of WGEs and uWGEs were truncated to match the length of the UCNE sequences, resulting in each WGE and uWGE sets having the same sequence length distribution as the UCNE database.

ClinVar database has been downloaded from NCBI FTP site (https://ftp.ncbi.nlm.nih.gov/pub/clinvar/vcf_GRCh37/) in the format VCFv4.1 (fileDate = 2024-01-27), last accessed on 20 March 2024.

### Feature calculation for machine learning (ML)

Features were calculated using a series of our Perl programs: make_features_F1_F2_F8.pl, make_feature_F3.pl, make_features_F4_F5.pl, make_features_F6_F7.pl, make_feature_F9.pl.

The feature calculations for UCNE sequences and uWGE sequences were combined into one table using the *feature_input_table.pl* program.

All Perl programs are available on our website (http://bpg.utoledo.edu/∼afedorov/ lab/UCNE3.html, accessed on March 20, 2024), in a package that includes an Instruction Manual (UCNE3instruction.docx). In addition, this package of programs and instructions is available in the Supplementary File S1.

*Feature #1* (F1) and *Feature #2* (F2) are related to frequencies of GpC, GpG and CpC dinucleotides (see Results section for further explanation).

*Feature #3* (F3) encompasses 17 dinucleotide pairs separated by one or two nucleotides: TAn(n)GA, TAn(n)GC, TAn(n)GG, TAn(n)GT, ACn(n)TA, CCn(n)TA, GCn(n)TA, TCn(n)TA, AAn(n)GC, ATn(n)GC, GCn(n)TT, AGn(n) AT, GGn(n)AT, AGn(n)GT, ACn(n)CT, ATn(n)CT, GCn(n)TC. See Results section for more details.

*Feature #4* (F4) is the combined frequency of GC-rich triplets. F4 includes eight triplets: GGG, CCC, GAG, CCT, CCA, CTC, TGG, AGG

*Feature #5* (F5) is the combined frequency of AT-rich triplets. F5 includes four triplets: TTA, TAA, ATT, AAT.

*Feature #6* (F6) is the combined frequency of 17 adjacent dinucleotide pairs that are enriched within UCNE sequences compared to uWGE sequences. F6 includes: AATT, TACA, TTAC, GTAA, TTAT, ATAA, AATG, CATT, ATTA, TAAT, TCAT, ATGA, TTAA, TCAA, TTGA, CAAT, ATTG.

*Feature #7* (F7) is the combined frequency of 18 dinucleotide pairs that are separated by one nucleotide (*e.g*. ACnGC) that are enriched within UCNE sequences compared to uWGE sequences. F7 includes: ACnGC, GCnGT, ACnGT, AAnAG, CTnTT, TGnCA, CTnAT, ATnAG, CAnTA, TAnTG, TTnAT, ATnAA, TTnTC, GAnAA, TAnTA, GTnAT, ATnAC, ATnAT.

*Feature #8* (F8) is the ratio of Purine/Pyrimidine (Pyrimidine/Purine) dinucleotides (TpG, CpA, GpT, ApC) to Purine/Purine (Pyrimidine/Pyrimidine) dinucleotides (ApG, CpT, GpA, TpC).

*Feature #9* (F9) represents GC-content of the sequences and is explained further in the Results section.

### Data preprocessing

Before Machine Learning algorithms were employed, the data was pre-processed in R version 4.2.3 and Python version 3.12.2. First the input data was randomly split into 70% training and 30% testing sets using the caTools R package (18). Both training and testing data were normalized using the generic scale function in R.

The data was preprocessed in the same manner using the SciKit Learn package and workflow in Python (19).

### Machine learning implementation

Once the data was prepared, three ML algorithms were applied using our R code, *ML_model.R* and Python code *ML_model.py*. SVM model was trained and tested using the e1071 R package (20). The classifier was manually tuned for the best parameters. Our model used the radial kernel, 1.0 cost and 0.155 gamma. The random forest model was trained and tested using the randomForest R package (21). Our model used all default parameters which produced 500 trees. Finally, the neural network model was trained and tested using the nnet R package (22). Our network had 5 hidden layers, 0.1 decay, and a maximum number of weights at 1000. The Receiver operator characteristics (ROC) curves and the area under the curve (AUC) were computed using the pROC R package (23) and plotted using the ggplot2 package (24).

To validate our results, we applied the three models with the same parameters using the built in functions provided by the SciKit Learn package in Python (19). All codes are available in the Supplementary File S1.

### Statistics

In preprocessing, both the training and testing dataset is scaled using the generic scale function in R. The mean is subtracted from each element and divided by the standard deviation to normalize the dataset. Once preprocessed and ML algorithms were employed, each confusion matrix was extracted and evaluated using the Caret R package (25) (Supplementary Figure S1). From the confusion matrix we calculated the sensitivity, specificity, 95% confidence interval, and total accuracy for each model (Supplementary Table S1).

## Results

### Datasets

**Figure 1.**
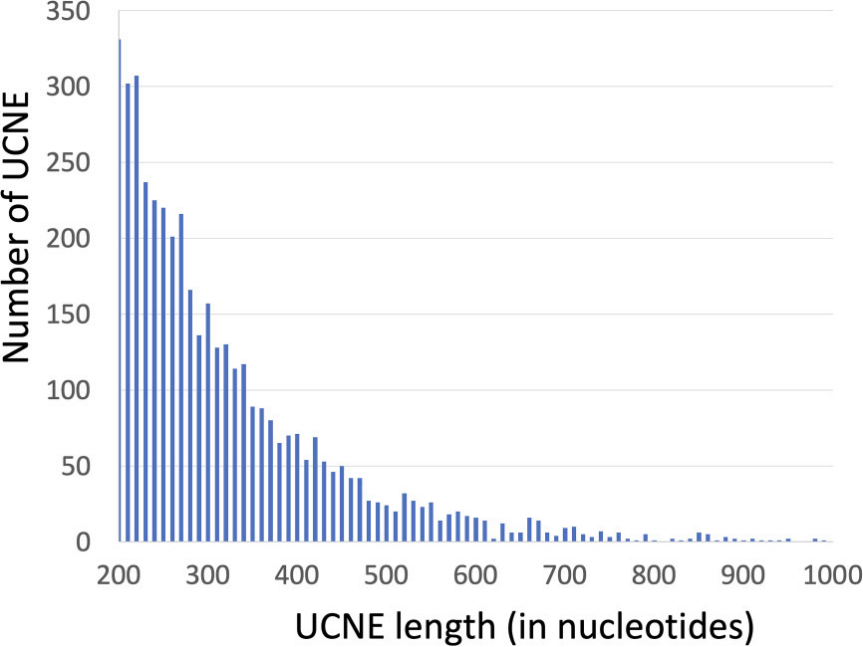
Length distribution of UCNE sequences. Randomly generated WGE and uWGE sequences have the same length distribution.

Ultra-conserved non-coding elements (UCNEs) vary in length, typically beginning at around 200 base pairs (bp) and infrequently extending up to 1000 bp. The distribution of UСNEs by their length is shown in Figure 1. For an accurate comparison of UCNEs with the rest of the genome, we created two types of datasets from randomly chosen human genome fragments. The first dataset, named Whole-Genome Elements (WGEs), contains randomly chosen fragments with the same length distribution as the UCNE database. The second dataset, named unique Whole-Genome Elements (uWGE), consists of unique genomic sequences, that lack DNA repetitive elements. Each WGE and uWGE dataset was designed to match the UCNE dataset in both the number of sequences and their cumulative length.

### Generation of features of UCNE and genomic sequences for machine learning (ML)

For every UCNE and control sequence from both WGE and uWGE, we calculated how frequently a particular dinucleotide pattern appears. To get a frequency for this pattern, we divided the total number of pattern occurrences by the length of the DNA sequence it was found in. This pattern frequency, calculated for each nucleotide sequence in our databases, serves as a ‘*feature’* or input variable for ML algorithms. In total, we generated nine different features, which are exemplified in Table 1 and described in more detail below.

**Table 1.** Example of input data of nine features of UCNE and uWGE sequences for further ML analysis

ID	F1	F2	F3	F4	F5	F6	F7	F8	F9	Class
WGunique-1	12.86	17.14	171.43	68.57	11.79	5	4.29	0.37	52.14	0
WGunique-2	11.07	9.88	47.83	41.5	13.04	4.74	5.53	0.62	45.06	0
WGunique-3	17.71	25.14	116.67	74.57	0.86	1.43	4.57	1.17	64.29	0
WGunique-4	3.11	5.36	70.73	21.28	55.02	14.53	10.55	1.03	28.37	0
WGunique-5	6.42	7.92	120.69	29.55	42.4	11.99	9.64	0.79	35.55	0
…										
chr21_Griselda	13.33	11.9	105.88	35.71	45.71	13.81	12.86	1.1	43.33	1
chr21_Gwyneth	4.63	2.78	62.5	12.5	51.39	15.74	6.94	2.46	27.31	1
chr21_Hana	8.7	9.13	180	35.22	27.39	9.57	9.57	1.22	40.87	1
chr21_Havana	15.79	16.54	100	53.01	6.77	1.88	5.64	0.77	55.64	1
chr21_Hector	5.31	8.16	64.29	29.6	22.2	7.02	7.59	1.05	36.05	1

The first column represents identifiers for each sequence, the last column dataset type: 0 – uWGE, 1 – UCNE. The real table contains data on 4272 UCNEs and the same number of uWGE sequences.

#### Feature #1 and #2

**Figure 2.**
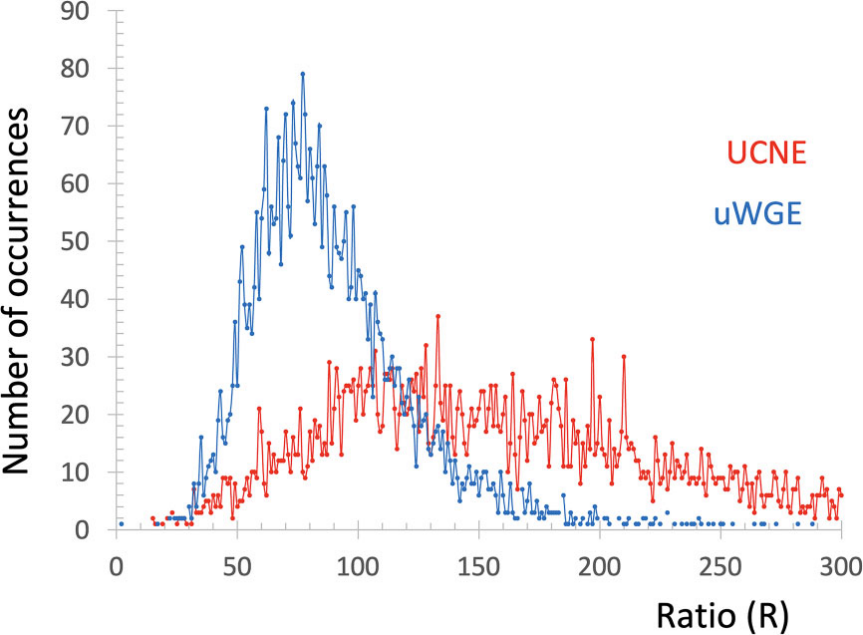
Distribution of number of UCNE and uWGE by the ratio (*R*) of their feature #1 to feature #2 values. This ratio was normalized for interpretation simplicity *R* = (2*F1/F2)×100%.

In our previous research, we found that UCNE sequences are unique from the whole genome primarily due to the overabundance of GpC dinucleotides and underabundance of CC and GG dinucleotides Fedorova *et al.* (12). Interestingly, CpG dinucleotides within UCNE are underrepresented as expected for randomly chosen fragments of the human genome and could not serve as markers for ultraconserved DNA. Therefore, we chose the frequency of GpC dinucleotide (F_GpC_) as Feature #1 (F1) and the combined frequency of CC + GG dinucleotides (F_CC+GG_) as Feature #2 (F2). These are outlined in Table 1. To evaluate our features for their UCNE-prediction ability, we plotted their frequency distributions within UCNE and control uWGE sequences, as demonstrated in Figure 2. In this figure, we used the ratio of F_GpC_/(F_CC+GG_) because these frequencies exhibit inverse patterns in the two datasets: F_GpC_ is overrepresented in the UCNE dataset, while (F_CC_+ _GG_) is underrepresented. Figure 2 demonstrates distinct peaks for UCNEs versus uWGEs, indicating that the prediction ability of UCNE using this ratio is 74% accurate. Initially we considered the F_GpC_/(F_CC+GG_) ratio to be a single feature. However, subsequent ML experiments showed that employing F_GpC_ and F_CC+GG_ as separate features (F1 and F2) yielded marginally better results for the Support Vector Machine (SVM) ML approach. Therefore, we used F_GpC_ and F_CC+GG_ as F1 and F2 in our final experiments. We evaluated all generated features for their UCNE prediction ability with the same simple approach and incorporated a feature into our ML analysis if its prediction power exceeded 67%.

#### Feature #3

**Figure 3.**
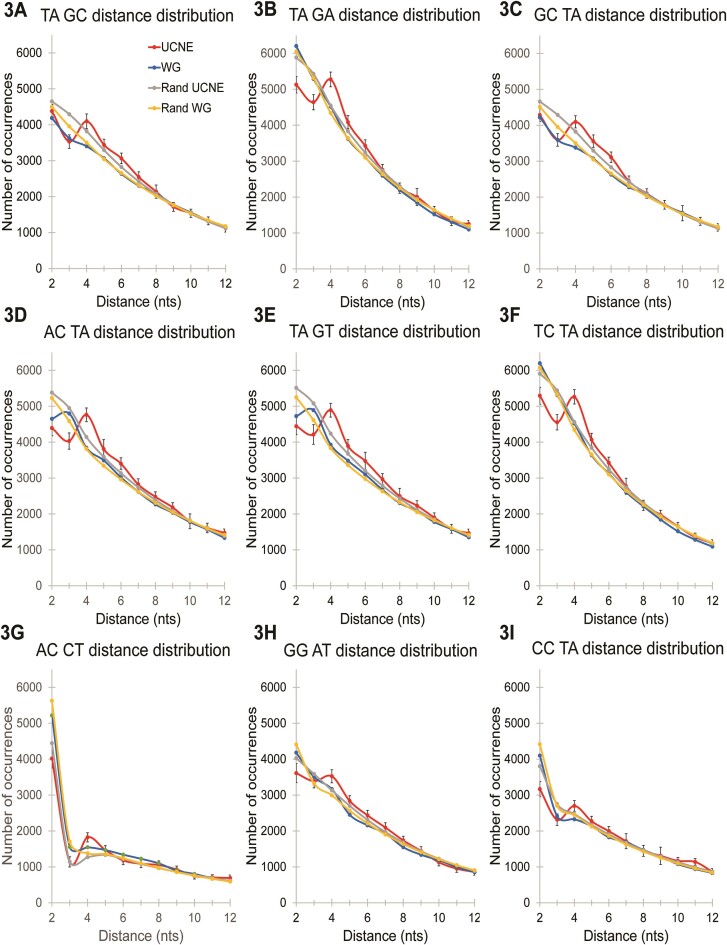
Distribution of spacing distances between pairs of particular dinucleotides for UCNE (red), uWGE (blue), random UCNE (yellow) and random uWGE (gray) as described in Fedorova *et al.* (16). The 99.7% confidence intervals (±3σ) are demonstrated for UCNE datasets as vertical bars. Statistical errors for averaged uWGE, random uWGE, and random UCNE are 30 times less than standard deviation for UCNE and are invisible in these graphs.

One of the prominent patterns of dinucleotide arrangements described in Fedorova *et al.* (16) is shown in Figure 3. Seventeen different dinucleotide pairs have the same peak across UCNE curves. This characteristic peak, which is absent in whole genome and quasi-random curves, always exists at the same distance and signifies a specific spatial arrangement where two dinucleotides are separated by two nucleotides (referred to as a distance of L = 4 nucleotides in previous nomenclature). Since the occurrence of a particular dinucleotide pair (for instance, *TAnnGC* as depicted in Figure 3a, with *n* standing for any nucleotide) happens approximately once every 250 nucleotides, this infrequency renders the pattern insufficient to serve as a standalone feature. To address this, we aggregated the occurrence of all seventeen dinucleotide patterns, nine of which are shown in Figure 3, and calculated the total number of their occurrences inside each UCNE and uWGE sequence for two specified distances: L = 3 (e.g. *TAnGC*) and L = 4 (e.g. *TAnnGC*). We then divided the total number of occurrences of all patterns at L4 by the total number of all patterns at L3. This ratio, defined as Feature #3 (F3) provides a comparative metric: values greater than 1 are indicative of UCNEs, while values less than are indicative of whole genome sequences. Initial assessments demonstrated that the prediction power for F3 is 68%.

#### Features #4-8

We examined patterns for all 256 possible pairs of dinucleotides, which are presented in Supplementary File S2. By analogy to the described feature F3, we grouped patterns according to their shape characteristics that distinguish UCNE from uWGE sequences and evaluated these groups for their prediction ability. Using this approach, we generated five more features (F4 through F8) for ML, as described in the Materials and Methods section. F4 and F5, in particular, quantify the frequency of GC-rich and AT-rich triplets (here, triplets are defined as two overlapping dinucleotides separated by one nucleotide at distance L = 1). Feature #6 (F6) is composed of 17 pairs of adjacent dinucleotides (L = 2), and Feature #7 (F7) consists of a set of 18 dinucleotide pairs separated by a single nucleotide (e.g. ACnGC where L = 3), which appear significantly more often in UCNE sequences than in uWGE sequences. Lastly, Feature #8 (F8) is the ratio of dinucleotides composed of alternating purine and pyrimidine bases (TpG, CpA, GpT, ApC) to the dinucleotides composed of homopyrimidine or homopurine bases (ApG, CpT, GpA, TpC).

#### Feature #9

**Figure 4.**
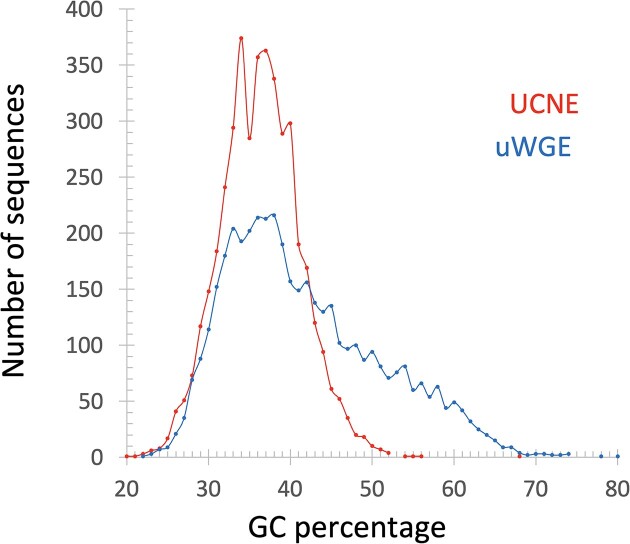
Distribution of GC-content in UCNE and uWGE sequences.

In the study by Fedorova *et al.* (12), it was established that the UCNE dataset is GC-poor compared to the whole genome datasets (showing a GC content of 37% and 42%, respectively). Building on this observation, we identified the GC content as our ninth feature for ML. We calculated the GC-content percentage for each examined nucleotide sequence. The distribution of GC-content for UCNE and uWGE datasets is demonstrated in Figure 4. While there is significant overlap in the data, the distribution suggests a threshold for classification: if a tested sequence has a GC-content above 46%, it is likely associated with WGE, whereas if the GC-content is between 32% and 40%, there is a high probability that it belongs to UCNE.

### Machine learning for UCNE classification

**Figure 5.**
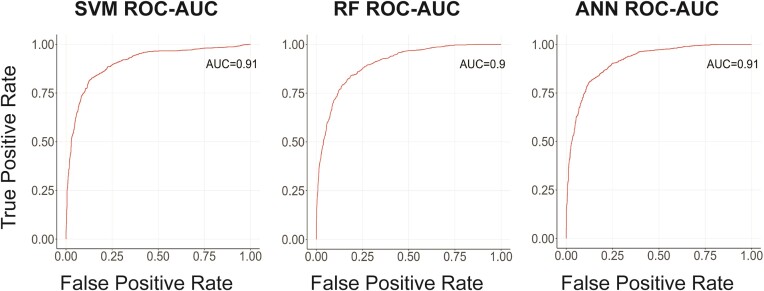
The receiver Operating Characteristic (ROC) for classification performance for each ML model.

We compiled all nine described features into a single dataset, organized in a format compatible with the ML R-package, illustrated in Table 1. The dataset comprises 4272 pairs of UCNE and WGE sequences, with the sequence class indicated in the final column (1 for UCNE, 0 for WGE). We acknowledge that different features have different scales in Table 1, yet at the initial step of ML, they were normalized using a standard R package to ensure consistency in our approach. We always set ML in the following proportion: 70% of the data allocated for training and 30% reserved for testing the model's performance. We employed three very popular ML approaches—Support Vector Machine (SVM), Random Forest (RF) and Artificial Neural Network (ANN)—to rigorously test for classification of UCNE versus whole genome. Additionally, we used independent packages in R and Python for these three ML approaches (R-package and Python (Sci kit learn)). Our models achieved a consistently high accuracy rate in classifying UCNE sequences, with performance typically above 80% and in some conditions higher than 90% (see below this section). Because the whole genome is 2000 times larger than the entire UCNE sample, we generated one hundred WGE and uWGE subsets, observing the variability of approximately 1% (one sigma) between them, indicating stable performance across different genomic samples. The results presented for UCNE classification accuracy are average for WGE and uWGE subsets (not extreme). We optimized the parameters of ML for our project. For the SVM, we selected the radial kernel, while for RF we used default parameters, as they demonstrated excellent performance. The ANN was configured with the following parameters: a network size of 5 and a decay parameter of 0.1. Figure 5 illustrates the Receiver Operating Characteristic (ROC) values for ML prediction ability between UCNE and uWGE. We plotted the ROC curve (Figure 5) and calculated the Area Under this Curve (AUC). Both the SVM and ANN models had an AUC of 0.91, while the RF model had an AUC of 0.90.

Altogether, the classification accuracy for SVM and ANN was 83% and for RF - 82%. When instead of unique uWGE we used randomly selected genomic fragments from all chromosomes that frequently contain different types of DNA repeats (WGE datasets), the accuracy decreased slightly (82% for SVM, 81% for ANN, and 80% for RF). However, when we created WGE datasets from the same chromosome (by picking the next WGE sequence by walking 5000 nts down the chromosome and so on), accuracy rates for all three ML techniques frequently exceeded 90%. This improved performance likely reflects how different chromosomes slightly vary from each other by nucleotide composition and are comprised of isochores—long DNA segments containing millions of nucleotides with relatively homogeneous GC content (26). Finally, increasing the length of WGE sequences (let's say having them all 500 nucleotides long) increases the ML prediction ability by a few percentage points. This is attributable to the greater number of pattern occurrences in longer DNA fragments, which redUCNEs the statistical variability and hence increases the reliability of our ML-based predictions.

Overall, our research showed that integrating weak but numerous signals in dinucleotide arrangements, which are specific to UCNEs, allowed us to effectively distinguish these evolutionarily stable DNA fragments with an impressive accuracy of 84%.

### Negligible proportion of 31 046 SNPs inside UCNEs in ClinVar database

To evaluate the effects of genetic variations within UCNEs on phenotypic traits, we turned our investigation to the ClinVar NCBI database, which contains 2 346 913 human variations with reported effects, to determine the presence of UCNE-associated SNPs. From the entire set of 31 046 human SNPs identified within UCNEs by Fedorova *et al.* (12) we sought to establish their representation in the ClinVar NCBI database. This data is cataloged in Table 2. To contextualize these findings statistically, we created one hundred random sets of human SNPs from the 1000 Genomes Project dataset, with each set the same size as our UCNE dataset (31 046 SNPs each). An analysis of how these one hundred random SNP datasets overlap with ClinVar is also detailed in Table 2. The phenotypic effects of SNPs are described in the first column of this table and were obtained ‘as is’ from the field ‘CLNSIG’ of ClinVar VCF dataset. The data in Table 2 reveals that SNPs within UCNEs are not enriched in ClinVar. From our UCNE-related SNP investigation, the sole ‘pathogenic’ SNP (rs139649711) we identified from Table 2 is located within the *FOXP2_Griselda* UCNE element inside the FOXP2 gene. The *FOXP2_Griselda* UCNE sequence is 412 bp long and overlaps an intron and an exon of the FOXP2 gene. The SNP is located at the beginning of the intron (10th position). Additionally, the sole ‘likely-pathogenic’ UCNE SNP (rs374400665) is located inside the leucine-rich pentatricopeptide repeat-containing gene (LRPPRC) within the *LRPPRC_Trystan* UCNE element that is also partly intronic and exonic. This SNP is inside the exon and is a missense variant. Both aforementioned SNPs are outliers, given that the vast majority of UCNE sequences are classified as non-coding sequences, located in either intergenic regions or introns. The comparatively smaller number of UCNE SNP matches with ClinVar, relative to random SNP subsets, align with expectations because SNPs inside UCNEs tend to be enriched with very rare variants, as previously reported by Fedorova and co-authors (12). Monte Carlo simulations involving 100 random SNP subsets demonstrated that the frequency of pathogenic SNPs inside UCNEs found in ClinVar does not exceed the frequency expected from randomly selected SNPs from the entire genome. This finding is statistically significant with a *P*-value of 0.01.

**Table 2.** Characterization of SNPs from UCNE and random SNP datasets, that are present in ClinVar database

ClinVar Significance categories	Number of instances for UCNE SNPs	Average number of instances for 100 Random SNP datasets
Pathogenic	1	2.1
Likely_pathogenic	1	1.1
Conflicting_classifications_ of_pathogenicity	7	14
Benign	37	64
Likely_benign	25	48
Uncertain_significance	17	48
risk_factor	0	0.07
drug_response	0	0.01

## Discussion

### Does dinucleotide composition of UCNE create specific DNA conformation?

Recently, our team characterized numerous weak signals in dinucleotide composition/arrangement within UCNE sequences, which set them apart from other sequences in the human genome as well as computer-generated quasi-random sequences (16). The main goal of this paper was to explore whether dinucleotide non-randomness could be used to predict UCNEs computationally. The strongest distinguishing factors of UCNE sequences from the whole genome are the overabundance of GpC dinucleotides and underabundance of CpC and GpG dinucleotides. The frequency of these dinucleotides alone allows us to differentiate between UCNEs and WGEs with 74% accuracy. Combining the main prominent signals in UCNE dinucleotide arrangements allows us to improve the ability to differentiate between UCNEs and WGEs by up to 84%. This underscores the crucial role of dinucleotides in the formation of UCNEs. We previously discussed that dinucleotides are pivotal for the realization of different DNA conformations (16). A review about ‘sequence-dependent structural properties of B-DNA’ by Da Rosa and co-authors (27) described 16 parameters that affect how nucleotide bases are spatially arranged relative to each other. These local space variations in bases position/orientation define multiple DNA conformations and their sub-forms. Svozil *et al.* (28) performed an extensive computational analysis of all known DNA structures in the Protein Data Bank (PDB). They studied the distribution of 7739 dinucleotides inside BI (canonical), BII, and restB (unclassified) conformations of B-form DNA; AI, AII, restA conformations of A-form; A/B, and B/A conformations (see Table 8 and Figure 2 of Svozil *et al.* (28)). According to their findings, specified in their Table 8 (page 3700), GpC dinucleotides have the strongest overrepresentation in restB-form and B/A-form and the strongest underrepresentation in the canonical BI-form. Conversely, the GpG dinucleotide has very strong preference to BII-conformation and avoidance of B/A and restB conformations. These observations suggest that strong peculiarities of GpC, GpG, and CpC inside UCNEs should cause their DNA sequences to adopt a specific conformation, which differ from the canonical BI-form. The ability to change its conformations is a pivotal property of DNA. As pointed out by Pellionisz (29) about genomic attributes, ‘DNA is an unsupervised operating system’ rather than merely a simple set of instructions. Therefore, we conjecture that the unique dinucleotide composition of UCNEs is somehow intrinsically related to their specific DNA conformations.

### How UCNEs may keep their sequences intact for hundreds of millions of years

Numerous studies on the mutational dynamics inside UCNEs suggest that UCNEs undergo a strong negative (purifying) selection against mutations to preserve the UCNE sequence through time (4,5,7,30–32). This process implies that mutations inside UCNEs tend to produce deleterious alleles. Carriers of deleterious alleles have fewer offspring each generation, leading to a reduced frequency of the mutation within the gene pool. The inexplicable problem with this scenario is that the mutations inside UCNEs rarely create observable phenotypic effects. This raises the question as to how purifying selection could work on every base of a prolonged UCNE sequence, which can span hundreds of nucleotides long. Even those who support the functionality of UCNEs as enhancer elements agree that enhancers are typically much shorter than UCNEs. Futhermore, not every base within an enhancer is essential for its function, leading to the expectation of evolutionary non-conservation for certain nucleotides within the UCNEs (Snetkova *et al.* (11)). Given the absence of satisfactory explanations for these puzzling properties of UCNEs, we propose our own hypothesis on this subject.

### Homologous DNA pairing hypothesis

Scientists have not discovered phenotypic effects for most of the mutant alleles within UCNE sequences for humans or mice (5,8,33). We bioinformatically confirmed this phenomenon as well by demonstrating that 31 046 mutant alleles inside UCNEs are practically not present in the ClinVar database (see Table 2). While obvious phenotypic effects of mutations inside UCNEs are hard to detect, they may still have significant consequences during specific stages of development, such as gametes. Indeed, mammalian male organisms produce millions of spermatozoids, each with its own unique set of mutant alleles inside the entire pool of UCNEs. Phenotypic variations between single haploid cells are challenging to observe and thus, they have not been extensively studied. Furthermore, there is another enigmatic process associated with gametogenesis, initially known as Crick's unpairing hypothesis (34). This process is the conjugation of homologous chromosomes during meiosis. In a helical DNA duplex, the bases are inward facing. A key question arises: how could inward-facing bases in one DNA duplex look outwards to recognize homologous bases in another DNA duplex to start conjugation? There are several suggestions on how this may occur, investigated by Forsdyke 2007 (35); Falaschi 2008 (36); Baldwin et al. 2008 (37); Kornyshev and Leikin (38); Mazur and Gladyshev (39), Sen and Gilbert (40), among others. However, a consensus has yet to be reached. The initial chromosomal conjugation is known as *recombination-independent homologous double-stranded dsDNA pairing* and was recently thoroughly reviewed by Mazur and Gladyshev (39). This process has not been comprehended yet and controversial opinions on this subject exist in the literature. One of the problems with mammalian dsDNA pairing is that chromosomes are enriched with thousands of copies of interspersed repetitive elements (e.g. *Alu* in humans and *B-1* in mice), which could potentially interrupt proper dsDNA-dsDNA homologous pairing. We noticed that UCNE sequences may be ideal candidates to resolve this dsDNA pairing predicament. Indeed, UCNE sequences: (i) are long enough (>200 nts) to ensure specific and strong dsDNA–dsDNA pairing; (ii) have no similarity to each other, so their homologous interaction would be chromosomal-specific; (iii) as we’ve demonstrated, UCNE likely have a specific DNA conformation that we surmise could act as a signal for the initiation of homologous dsDNA pairing. Supporting this idea, Mazur and Gladyshev (39) recently published their scenario for non-recombinant initial pairing of dsDNA homologs. According to their hypothesis, this process happens via specific DNA conformation known as C-form DNA, which is structurally akin to the B-form but has certain distinguishing features, such as a shallower main groove than B-form. Molecular dynamic computations by Mazur (41) demonstrated that dsDNA homologous pairing may be initiated between homologous DNA in the C-form conformation without any protein assistance (see Figure 2 for details on page 580 of Mazur and Gladyshev (39)). These facts allowed us to draw clear parallels between the Mazur and Gladyshev hypothesis (39) and our investigations of possible DNA conformations of UCNE sequences.

We hypothesize that specific DNA conformation in UCNEs, like C-form DNA, may initiate dsDNA pairing of homologous chromosomes in mammals and other vertebrates. Should there be an excessive number of UCNE mutations, they may interfere with dsDNA-dsDNA homologous pairing and affect meiosis. Thus, purifying selection against non-proper dsDNA homologs pairing may be triggered and act against its cell-host during meiosis, thereby keeping UCNE sequences ultra-conserved over evolutionary timescales.

## Supplementary Material

lqae074_Supplemental_Files

## Data Availability

Supporting data can be downloaded at http://bpg.utoledo.edu/∼afedorov/lab/UCNE3.html.
