# Supplementary material for: Computational identification of ultra-conserved elements in the human genome: a hypothesis on homologous DNA pairing
Source: NAR Genom Bioinform. 2024 Jul 2;6(3):lqae074. doi: 10.1093/nargab/lqae074 (PMC11217675; doi:10.1093/nargab/lqae074)
Supplement: lqae074_Supplemental_Files [file lqae074_supplemental_files.zip › SupplementaryFile1_revision.pdf]

Supplementary File S1

Supplementary Figure S1

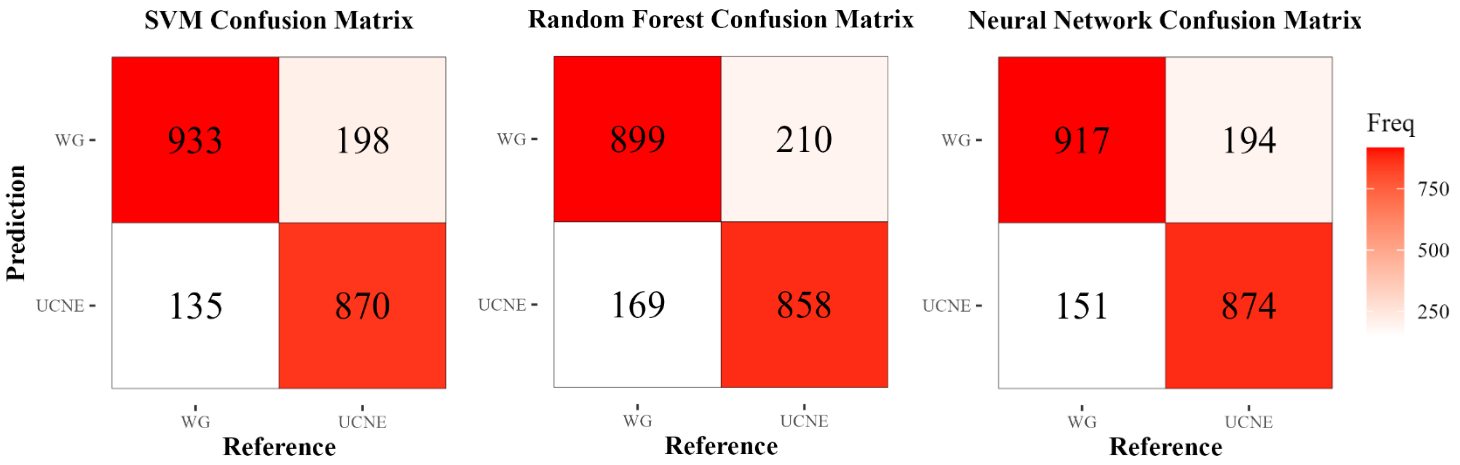

Supplementary Figure S1: Confusion matrix results from each model: SVM, random forest, and neural network. X axis represents predictions from the models and the Y axis represents the true reference group. Each box within the matrix represents the total counts between predictions versus references. The red color gradient represents less frequent counts (white) to more frequent counts (red).

Supplementary Table S1

**Model**	**Accuracy**	**Specificity**	**Sensitivity**	**95% CI**
SVM	84.4%	81.4%	87.4%	0.828,0.859
Random Forest	82.5%	80.1%	84.5%	0.808,0.841
Neural Network	83.4%	82.0%	85.0%	0.818,0.850

Supplementary Table S1: Statistical results of each model: SVM, Random Forest, and Neural Network. Columns from left to right: Accuracy, Specificity, Sensitivity, and 95% confidence interval.

Instruction Manual for Programs

Feature Calculation Programs
Features were calculated using five Perl programs. Every program has two options for input data, UCE or WG, that will be calculated separately per input data.

Command line:
perl make_features_F3.pl UCE or perl make_features_F3.pl WG

Therefore, to calculate the feature for both UCE and WG sequences, the program must be ran twice with the corresponding argument as shown above. Each program saves the specified feature in a separate file. Each output file is named according to what feature/s are calculated and input data. For example, score128_UCE or score128_WG.

Feature table Program
Once all the feature calculation programs are ran for both UCE and WG sequence, the final input table can be created using the F_input_tab.pl program. This program will result in a csv file named "input_table.csv" that can be used for machine learning algorithms.

Command line:
perl feature_input_table.pl

Machine Learning Models in R
Three machine learning models were trained and tested in R version 4.2.3; SVM, Random Forest, and Neural Network using the ML_model.R code. This code contains the preprocessing, model training/testing, results analysis and statistics, and ROC-AUC curves.
For this code 8 packages must be installed and loaded into the environment: e1071, caTools, caret, pROC, randomForest, nnet, predtools, and ggplot2 (read materials and methods for more information).

The same three models were trained and tested in Python version 3.12.2 using the SciKit Learn package as well (see materials and methods for more information). This code is available in ML_model.py and follows the workflow available on the SciKit Learn website (https://scikit-learn.org/stable/).

Perl Programs
make_features_F1_F2_F8.pl

```
#!/usr/local/perl
#Program for counting features 1, 2, and 8.

@GC1 = ('GG', 'CC');  # Feature 1 DNT ratio:  #GC/(#CC +#GG)
@GC2 = ('GC'); # Feature 2, GC counts
@RY = ('TG', 'CA', 'GT', 'AC'); #Feature 8, Purine Pyrimidine pairs
@YY = ('AG', 'CT', 'GA', 'TC'); #Feature 8,Purine Purine pairs

$rich=0;
$TOT=$zero=$more=$less=0;
```

```
%DIST=%DISTgc=();
$min=$max=0;
$c_uce=$c_wg=$unknown=0;

chomp($ARGV[0]);

#open UCE or WG sequences
if($ARGV[0] eq "UCE") {
        $FILE ="score128_UCE";
        open(OUT, ">$FILE");
        $/='>';
            #open (IN2, "../../UCEfasta_sorted.txt") or die;
       open(IN2, "UCEdatasetNOV2023") or die "Couldn't open file fasta, $!";
}
elsif($ARGV[0] eq "WG") {
        $FILE ="score128_WG";
        open(OUT, ">$FILE");
        $/='>';
            #open (IN2, "../../DATA/chr11_unique377") or die;
        open(IN2, "WGdatasetNOV2023_1") or die "Couldn't open file fasta, $!";
        #open(IN2, "WGdataset2024_5") or die;
}
else{print "wrong ARGV[0]\n"; die;}
#Create Output file
open(OUT, ">$FILE");
#Calculate features 1, 2, and 8
while(<IN2>) {
            @line=(); $id='';
            $c++;
            chop($_); chomp($_);
            @line=split(/\n/, $_);
            if ($ARGV[0] eq 'WG' ) {$id =substr($line[0],0,17);}
        else {if($line[0] =~/^([0-9a-zA-Z_\-]+)/) {$id=$1;}}

            $seq ='';
            for $x (1..$#line) {
                        chomp($line[$x]);
                        $seq .=$line[$x];
            }
            #upper case
            $seq2=uc($seq);
            @S=split(//,$seq2); #split by each nt
            $tot=$tot2=0; $length =length($seq2);
            $ry=$yy=0;

            if ($c>1) {
                        $X=0;$R=1;
#                       print "\n\n$c\n$seq2 \n";
                        #Calculation of Feature 1
                        for $x (0..$#GC1) { #GG / CC
                                    for $z (0..$#S) { #for each nt
```

```
				$di=$S[$z] . $S[$z+1]; #create dnt code
				if($di eq $GC1[$x])  { #if match GG / CC
					$tot++;
		#				print "$x \t $tot \n";
				}
			}
		}
		$tot/=$length;
#		print "$tot \t$length \n";
		#Calculation of Feature 2
		for $x (0..$#GC2) { #GC
			while ($seq2 =~ /$GC2[$x]/g) { #if match GC
				$tot2++;
		#			print " $&\n";
			}
		}
		$tot2 *=2; #mult GC count by 2
		$tot2/=$length;
#		print "$tot2 \t$length \n";

		#Calculation of Feature 8
		for $x (0..$#RY) {
			while ($seq2 =~ /$RY[$x]/g) {
				$ry++;
			}
		}
		for $x (0..$#YY) {
			while ($seq2 =~ /$YY[$x]/g) {
				$yy++;
			}
		}

		if($yy){$R=$ry/$yy;}  #here we calculated multiplication coefficient R
		else{print "PROBLEM with entry $c \n";}
		#if($tot) {$X= $tot2/$tot; $X*=100;} #ratio of GC/GG+CC
		$tot*=100; #mult GG + CC by 10
		$tot2*=100; #mult GC by 10
		$R**=2; #square R

		#Round each feature
		$score2a=sprintf("%.2f",$tot); #F1
		$score2b=sprintf("%.2f",$tot2); #F2
		$score2c=sprintf("%.2f",$R); #F8

		print OUT "$id\t$score2a\t$score2b\t$score2c\n";

		#print OUT "$id,$scoreX\n";
		#$X=sprintf("%.2f",$X); #REMOVE INT
		#print OUT "$id,$X\n";
		$DIST{$X}++; #calc distribution
		if($X<87) {$min++;} #boundrary
```

```
                    else{$max++;}
          }
}
```

make_features_F3.pl

```
#!/usr/local/perl
#Program for counting feature 3, ratio of L4/L3

#L=4 patterns
@p4 = (
          'TA[ATGC]{2}GA',
          'TA[ATGC]{2}GC',
          'TA[ATGC]{2}GG',
          'TA[ATGC]{2}GT',
          'AC[ATGC]{2}TA',
          'CC[ATGC]{2}TA',
          'GC[ATGC]{2}TA',
          'TC[ATGC]{2}TA',
          'AA[ATGC]{2}GC',
          'AT[ATGC]{2}GC',
          'GC[ATGC]{2}TT',
          'AG[ATGC]{2}AT',
          'GG[ATGC]{2}AT',
          'AG[ATGC]{2}GT',
          'AC[ATGC]{2}CT',
          'AT[ATGC]{2}CT',
          'GA[ATGC]{2}TC',
          'GA[ATGC]{2}TC'
);
#L=3 patterns
@p3 = (
          'TA[ATGC]{1}GA',
          'TA[ATGC]{1}GC',
          'TA[ATGC]{1}GG',
          'TA[ATGC]{1}GT',
          'AC[ATGC]{1}TA',
          'CC[ATGC]{1}TA',
          'GC[ATGC]{1}TA',
          'TC[ATGC]{1}TA',
          'AA[ATGC]{1}GC',
          'AT[ATGC]{1}GC',
          'GC[ATGC]{1}TT',
          'AG[ATGC]{1}AT',
          'GG[ATGC]{1}AT',
          'AG[ATGC]{1}GT',
          'AC[ATGC]{1}CT',
          'AT[ATGC]{1}CT',
          'GA[ATGC]{1}TC',
          'GA[ATGC]{1}TC'
```

```
);

#opening data based off usr input
chomp($ARGV[0]);
if($ARGV[0] eq "UCE") {
	$FILE ="score3_UCE";
	open(OUT, ">$FILE");
	$/='>';
	open(IN2, "UCEdatasetNOV2023") or die "Couldn't open file fasta, $!";
}
elsif($ARGV[0] eq "WG") {
	$FILE ="score3_WG";
	open(OUT, ">$FILE");
	$/='>';
	#open(IN2, "../../DATA/chr11_unique377") or die "Couldn't open file fasta, $!";
	open(IN2, "WGdatasetNOV2023_1") or die;
	#open(IN2, "WGdataset2024_5") or die;
}

else{print "wrong ARGV[0]\n"; die;}
#opening output file
open(OUT, ">$FILE");
$/='>';
while(<IN2>) {
	@line=(); $id='';
	$c++;
	chop($_); chomp($_);
	@line=split(/\n/, $_);
	if ($ARGV[0] eq 'WG' ) {$id =substr($line[0],0,17);} #extract names for WG or UCE
	else {if($line[0] =~/^([0-9a-zA-Z_\-]+)/) {$id=$1;}}
	#print "$c\t$id\n";
	#create sequences
	$seq ='';
	for $x (1..$#line) {
		chomp($line[$x]);
		$seq .=$line[$x];
	}
	#convert all to uppercase
	$seq2=uc($seq);
	#print "$seq2\n";
	#MATCH DNTs for L3 and L4.
	$tot=$tot2=0; $s1a=$s1b=$score1a=$score1b=0; $length =length($seq2);
	if ($c> 1) {
		#print "\n\n$c\n$seq2 \n";
		#CALC counts for L=4 pattern
		for $x (0..$#p4) {
			while($seq2 =~ /$p4[$x]/g) {
				$tot++;
				#print "PATTERN is $&\n";
			}
		}
```

```
#           print "$id\t$length \n";
            #CALC counts for L=3 pattern
            for $x (0..$#p3) {
                    while($seq2 =~ /$p3[$x]/g) {
                            $tot2++;
                            #print " $&\n";
                    }
            }

            #Calculate ratio of L4 to L3 DNTs
#print "$tot\t$tot2\n";
            if($tot2) {$X= $tot/$tot2; $X*=100; } #L4/L3 * 100
        $X=sprintf("%.2f",$X); #round to two decimal point
            print OUT "$id\t$X\n"; #print ID, print score
    }

}
```

make_features_F4_F5.pl

```
#!/usr/local/perl
# Program for counting features 4 and 5, this program calculates relative frequencies of GC-rich  and AT-
rich triplets

%DISTat=%DISTgc=%DIST=();
$wg=$uce=0; $ATmin=$ATmax=$GCmin=$GCmax=0;
chomp($ARGV[0]);

#open UCE or WG sequences
if($ARGV[0] eq "UCE") {
     $FILE ="score45_UCE";
    open(OUT, ">$FILE");
     $/='>';
#       open (IN2, "../../UCEfasta_sorted.txt") or die;
     open(IN2, "UCEdatasetNOV2023") or die "Couldn't open file fasta, $!";
}
elsif($ARGV[0] eq "WG") {
     $FILE ="score45_WG";
     open(OUT, ">$FILE");
     $/='>';
        #open (IN2, "../../DATA/chr11_unique377") or die;
    open(IN2, "WGdatasetNOV2023_1") or die "Couldn't open file fasta, $!";
        #open (IN2, "WGdataset2024_5") or die;
}
else{print "wrong ARGV[0]\n"; die;}

#Create output file
open(OUT, ">$FILE");

while(<IN2>) {
```

```
        @line=();
        $c++;
        chop($_); chomp($_);
        @line=split(/\n/, $_);
        $seq ='';
        if ($ARGV[0] eq 'WG' ) {$id =substr($line[0],0,17);} #extract names for sequence
    else {if($line[0] =~/^([0-9a-zA-Z_\-]+)/) {$id=$1;}}
        #create sequences
        for $x (1..$#line) {
                chomp($line[$x]);
                $seq .=$line[$x];
        }
        #upper case
        $seq2=uc($seq);
        @S=split(//,$seq2);
        $length =length($seq2);
        $matchGC=$matchAT=0; $triAT=$triGC='';
        $scoreAT=$scoreGC=0; $ratio=0;
        if ($c>1) {
        #       print "\n\n$c\n$seq2 \n";
                for $z (0..($#S-2)) { #Count GC trips
                        $triGC=$S[$z] . $S[$z+1] .$S[$z+2];
                        if ($triGC =~/GGG|CCC|GAG|CCT|CCA|CTC|TGG|AGG/) {
                                $matchGC++;
                        }
                }
                for $z (0..($#S-2)) { #Count at trips
                        $triAT=$S[$z] . $S[$z+1] .$S[$z+2];
                        if ($triAT =~/TTA|TAA|ATT|AAT/) {
                                $matchAT++;
                        }
                }
                #Normalize  counts by length of sequence.
                if($length) {$scoreAT= $matchAT/$length *300;}
                $scoreAT =sprintf("%.2f",$scoreAT); #remove INT

                if($length) {$scoreGC= $matchGC/$length *300;}
                $scoreGC =sprintf("%.2f",$scoreGC); #remove int


                print OUT "$id\t$scoreGC\t$scoreAT\n";  #printing our SCORES 4 & 5
        }
}
```

make_features_F6_F7.pl

```
#!/usr/local/perl
#Program for counting features 6 and 7, fourmers and fivemers enriched in UCEs.

#feature 6
@p2a = (
```

```
        'AATT',
        'TACA',
        'TTAC',
         'GTAA',
         'TTAT',
         'ATAA',
         'AATG',
         'CATT',
         'ATTA',
        'TAAT',
         'TCAT',
         'ATGA',
         'TTAA',
         'TCAA',
         'TTGA',
         'CAAT',
         'ATTG'
);

#feature 7
@p2b = (
            'AC[ATGC]{1}GC',
        'GC[ATGC]{1}GT',
         'AC[ATGC]{1}GT',
        'AA[ATGC]{1}AG',
         'CT[ATGC]{1}TT',
         'TG[ATGC]{1}CA',
         'CT[ATGC]{1}AT',
         'AT[ATGC]{1}AG',
         'CA[ATGC]{1}TA',
         'TA[ATGC]{1}TG',
         'TT[ATGC]{1}AT',
         'AT[ATGC]{1}AA',
         'TT[ATGC]{1}TC',
         'GA[ATGC]{1}AA',
         'TA{ATGC]{1}TA',
         'GT[ATGC]{1}AT',
         'AT[ATGC]{1}AC',
         'AT[ATGC]{1}AT'

);

#opening data based off usr input
chomp($ARGV[0]);

if($ARGV[0] eq "UCE") {
            $FILE ="score67_UCE";
            open(OUT, ">$FILE");
            $/='>';
            open(IN2, "UCEdatasetNOV2023") or die "Couldn't open file fasta, $!";
}
```

```
elsif($ARGV[0] eq "WG") {
	$FILE ="score67_WG";
	open(OUT, ">$FILE");
	$/='>';
	open(IN2, "WGdatasetNOV2023_1") or die;
	#open(IN2, "../../DATA/chr11_unique1") or die "Couldn't open file fasta, $!";
	#open (IN2, "WGdataset2024_5") or die;
}
else{print "wrong ARGV[0]\n"; die;}

#opening output file
open(OUT, ">$FILE");

$count;
$/='>';
while(<IN2>) {
	@line=(); $id='';
	$c++;
	chop($_); chomp($_);
	@line=split(/\n/, $_);
	if ($ARGV[0] eq 'WG' ) {$id =substr($line[0],0,17);} #extract names for WG or UCE
	else {if($line[0] =~/^([0-9a-zA-Z_\-]+)/) {$id=$1;}}
	#print "$c\t$id\n";

	$seq ='';
	for $x (1..$#line) { #create sequences
		chomp($line[$x]);
		$seq .=$line[$x];
	}
	#upper case
	$seq2=uc($seq);
	#print "$seq2\n";

	#MATCH PATTERNS
	$tot=$tot2=0; $score2a=$score3a==0; $length =length($seq2);
	if ($c>1) {
		#print "\n\n$c\n$seq2 \n";
		for $x (0..$#p2a) { #count F6, fourmers
			while($seq2 =~ /$p2a[$x]/g) {
				$tot++; $count ++;
				#print "PATTERN is $&\n";
			}
		}
#		print "$tot \t$length \n";
		#print "$tot\t$tot2\n";
		for $x (0..$#p2b) { #count F7, fivemers
			while($seq2 =~ /$p2b[$x]/g){
				$tot2++;}
				}
		#normalize counts by length
		$tot/=$length; $tot2/=$length;
```

```
                    #multiple features by 100
                    $tot *=100;
                    $tot2 *=100;
                    #round features
              $score2a=sprintf("%.2f",$tot);
                    $score3a=sprintf("%.2f",$tot2);

                    print OUT "$id\t$score2a\t$score3a\n"; #print ID, print score
             #print "total : $count\n";
        }

}
```

make_features_F9.pl

```
#!/usr/local/perl
#Program for counting Feature 9,  for GC-percentage

%DIST=%DISTgc=();
$min=$max=0;

#open UCE or WG sequences data
if($ARGV[0] eq "UCE") {
        $FILE ="score9_UCE";
        open(OUT, ">$FILE");
        $/='>';
#           open(IN2, "../../UCEfasta_sorted.txt") or die;
        open(IN2, "UCEdatasetNOV2023") or die "Couldn't open file fasta, $!";
}
elsif($ARGV[0] eq "WG") {
        $FILE ="score9_WG";
        open(OUT, ">$FILE");
        $/='>';
            #open (IN2, "../../DATA/chr11_unique377") or die;
        open(IN2, "WGdatasetNOV2023_1") or die "Couldn't open file fasta, $!";
        #open (IN2, "WGdataset2024_5") or die;
}
else{print "wrong ARGV[0]\n"; die;}
#create output file
open(OUT, ">$FILE");


while(<IN2>) {
            @line=(); $id='';
            $c++;
            if ($c==1) {next;} #?
            chop($_); chomp($_);

            @line=split(/\n/, $_);
            if ($ARGV[0] eq 'WG' ) {$id =substr($line[0],0,17);} #extract names for UCE or WG sequence
        else {if($line[0] =~/^([0-9a-zA-Z_\-]+)/) {$id=$1;}}
```

```
        $seq ='';
        for $x (1..$#line) { #create sequences
                chomp($line[$x]);
                $seq .=$line[$x];
        }
        #upper case
        $seq2=uc($seq);
        @S=split(//,$seq2);

        $tot=$tot2=0; $length =length($seq2);
        $ry=$yy=0;

        #count G and C nucleotides
        $percent=0;
      while ($seq2 =~ /G/g) {$percent++;} #count G
      while ($seq2 =~ /C/g) {$percent++;} #count C
        #multiple by 100 to create percentage
        $percent *=100; #calc %

        if ($length) {$percent /=$length;} #normalize by length
        $percent =sprintf("%.2f",$percent); #Round feature

        print  OUT "$id\t$percent\n";
        #print "$id,$length\n";
}
```

feature_input_table.pl

```
#!/usr/local/perl
#Program that combines UCE and WG feature calculations into one Machine learning input file.
#create output files
open(OUT, ">input_table.csv");
#WG files
#extract Feature 3
open(IN1, "score3_WG") or die "Couldn't open file fasta, $!";
$c=0; %S1=();
while(<IN1>){
        chomp;
        $c++;
        @L=split(/\t/,$_);
        unless($L[1]) {print "$c\t$_\n";} #warning that score is absent
        if($S1{$L[0]}) {print "SEEN KEY $c \t $_\n";} #warning that the same key exist
        $S1{$L[0]}=$L[1];
}

#Extract features 1,2, and 8
open(IN2, "score128_WG") or die "Couldn't open file fasta, $!";
$c=0; %S2=(); %S2_b=();
while(<IN2>){
        chomp;
        $c++;
        @L=split(/\t/,$_);
```

```
	unless($L[1]) {print "$c\t$_\n";}
	if($S2{$L[0]}) {print "SEEN KEY $c \t $_\n";}
	$S2{$L[0]}=$L[1].",".$L[2];#cont value of hash like S4

	if($S2_b{$L[0]}) {print "SEEN KEY $c \t $_ \n";}
	$S2_b{$L[0]}=$L[3];
}
#Extract feature 9
open(IN3, "score9_WG") or die "Couldn't open file fasta, $!";
$c=0; %S3=();
while(<IN3>){
	chomp;
	$c++;
	@L=split(/\t/,$_);
	unless($L[1]) {print "$c\t$_\n";}
	if($S3{$L[0]}) {print "SEEN KEY $c \t $_\n";}
	$S3{$L[0]}=$L[1];
}
#Extract feature 4 and 5
open(IN4, "score45_WG") or die "Couldn't open file fasta, $!";
$c=0; %S4=();
while(<IN4>){
	chomp;
	$c++;
	@L=split(/\t/,$_);
	unless($L[1]) {print "$c\t$_\n";}
	if($S4{$L[0]}) {print "SEEN KEY $c \t $_\n";}
	$S4{$L[0]}=$L[1] . "," . $L[2]; #cont value of hash
	#print OUT "$L[0],$S1{$L[0]},$S2{$L[0]},$S3{$L[0]},$S4{$L[0]},0\n";
}

#Extract feature 6 and 7
open(IN4, "score67_WG") or die "Couldn't open file fasta, $!";
$c=0; %S6=();
while(<IN4>){
	chomp;
	$c++;
	@L=split(/\t/,$_);
	unless($L[1]) {print "$c\t$_\n";}
	if($S6{$L[0]}) {print "SEEN KEY $c \t $_\n";}
	$S6{$L[0]}=$L[1] . "," . $L[2]; #cont value of hash
#	print OUT "$L[0],$S1{$L[0]},$S2{$L[0]},$S3{$L[0]},$S4{$L[0]},$S6{$L[0]},0\n";

	#PRINT features in order into new file
	print OUT
"$L[0],$S2{$L[0]},$S1{$L[0]},$S4{$L[0]},$S6{$L[0]},$S2_b{$L[0]},$S3{$L[0]},0\n";
	#print OUT "$L[0],$S1{$L[0]},$S2{$L[0]},$S3{$L[0]},$S4{$L[0]},1\n";
}

#UCE FILES, same process as above
```

```
open(IN1, "score3_UCE") or die "Couldn't open file fasta, $!";
$c=0; %S1=();
while(<IN1>){
	chomp;
	$c++;
	@L=split(/\t/,$_);
	unless($L[1]) {print "$c\t$_\n";} #warning that score is absent
	if($S1{$L[0]}) {print "SEEN KEY $c \t $_\n";} #warning that the same key exist
	$S1{$L[0]}=$L[1];
}

open(IN2, "score128_UCE") or die "Couldn't open file fasta, $!";
$c=0; %S2=(); %S2_b=();
while(<IN2>){
	chomp;
	$c++;
	@L=split(/\t/,$_);
	unless($L[1]) {print "$c\t$_\n";}
	if($S2{$L[0]}) {print "SEEN KEY $c \t $_\n";}
	$S2{$L[0]}=$L[1].",".$L[2];#cont value of hash like S4

	if($S2_b{$L[0]}) {print "SEEN KEY $c \t $_ \n";}
    $S2_b{$L[0]}=$L[3];


}
open(IN3, "score9_UCE") or die "Couldn't open file fasta, $!";
$c=0; %S3=();
while(<IN3>){
	chomp;
	$c++;
	@L=split(/\t/,$_);
	unless($L[1]) {print "$c\t$_\n";}
	if($S3{$L[0]}) {print "SEEN KEY $c \t $_\n";}
	$S3{$L[0]}=$L[1];
}
open(IN4, "score45_UCE") or die "Couldn't open file fasta, $!";
$c=0; %S4=();
while(<IN4>){
	chomp;
	$c++;
	@L=split(/\t/,$_);
	unless($L[1]) {print "$c\t$_\n";}
	if($S4{$L[0]}) {print "SEEN KEY $c \t $_\n";}
	$S4{$L[0]}=$L[1] . "," . $L[2]; #cont value of hash
	#print OUT "$L[0],$S1{$L[0]},$S2{$L[0]},$S3{$L[0]},$S4{$L[0]},1\n";
}

open(IN4, "score67_UCE") or die "Couldn't open file fasta, $!";
$c=0; %S6=();
while(<IN4>){
```

```
            chomp;
            $c++;
            @L=split(/\t/,$_);
            unless($L[1]) {print "$c\t$_\n";}
            if($S6{$L[0]}) {print "SEEN KEY $c \t $_\n";}
            $S6{$L[0]}=$L[1] . "," . $L[2]; #cont value of hash
            #print OUT "$L[0],$S1{$L[0]},$S2{$L[0]},$S3{$L[0]},$S4{$L[0]},$S6{$L[0]},1\n";

            #PRINT features in order into new file
            print OUT
"$L[0],$S2{$L[0]},$S1{$L[0]},$S4{$L[0]},$S6{$L[0]},$S2_b{$L[0]},$S3{$L[0]},1\n";
            #print OUT "$L[0],$S1{$L[0]},$S2{$L[0]},$S3{$L[0]},$S4{$L[0]},1\n";
}
```

R programs

ML_models.R

```
#install/load packages
library(e1071) #SVM
library(caTools)
library(caret)

#Program for data prep, machine learning models, and visualizations.

###PREP####
#input data is merged UCE and WG data & change group assignment to binary
#dataset up : ID, v1,v2,...Group
#group is WG(0) vs UCE(1).
dataset = read.csv("input_table.csv")

#removing ID column
rownames(dataset) <- dataset$ID
dataset = dataset[,-1]

#setting the group assignments
dataset$Group = factor(dataset$Group , levels = c(0, 1))

#set seed for random number generation
set.seed(123)
#splits data based of group variable 75% train vs 25% test
split = sample.split(dataset$Group, SplitRatio = 0.75)
training_set = subset(dataset, split == TRUE)
test_set = subset(dataset, split == FALSE)

# Feature Scaling
#standardizes varibales. subtracting mean and divides by std. except the 6th        column(group)
training_set[-10] = scale(training_set[-10])
test_set[-10] = scale(test_set[-10])
####SVM####
```

```
#forumula = relationship between response variable and predictor variable. "."      means all other
variables as predictors
#type
#kernel
classifier = svm(formula = Group ~ .,
                 data = training_set,
                 type = 'C-classification',
                 kernel = 'radial',
                 cost = 1.0,
                 gamma = 0.155,
                 probability = TRUE


)


#prediction using the trained model, test_set(minus group)
y_pred_SVM <- predict(classifier, newdata=test_set[-10], probability=TRUE)
y_pred_SVM

#creates confusion matrix. compares Group column to prediction results.
#cm = table(test_set[,10], y_pred_SVM)
#cm
cm_SVM <- confusionMatrix(factor(y_pred_SVM), factor(test_set[,10]), dnn = c("Prediction",
"Reference"))

#confusionMatrix(table(y_pred_SVM,test_set[,10]))
###CM plot###
plt_SVM <- as.data.frame(cm_SVM$table)
plt_SVM$Prediction <- factor(plt_SVM$Prediction, levels=rev(levels(plt_SVM$Prediction)))

SVM_CM <- ggplot(plt_SVM, aes(Reference, Prediction, fill=Freq)) +
  geom_tile(color = "black") +
  ggtitle("SVM Confusion Matrix")+
  geom_text(aes(label=Freq),size=6,family="serif") +
  scale_fill_gradient(low="white", high="red",breaks=c(250,500,750)) +
  labs(x = "Reference", y = "Prediction") +
  theme(panel.background = element_blank(),
        axis.text.x = element_text(family="serif", size=8),
        axis.text.y = element_text(family="serif", size=8),
        plot.title=element_text( hjust=0.5, vjust=0, family="serif",face='bold',size=12),
        axis.title.x = element_text(size = 12,family="serif",face='bold'),
        axis.title.y = element_text(size = 12,family="serif",face='bold'),
        legend.position = "none"
        #legend.key.size = unit(0.5, 'cm'), #change legend key size
        #legend.key.height = unit(0.5, 'cm'), #change legend key height
        #legend.key.width = unit(0.5, 'cm'), #change legend key width
        #legend.title = element_text(size=10,family="serif"), #change legend title font size
        #legend.text = element_text(size=8,family="serif")) +
        )+ scale_x_discrete(labels= c("WG","UCNE")) +
        scale_y_discrete(labels= c("UCNE","WG"))
```

```
SVM_CM
ggsave(SVM_CM, file="SVM_CM.png", width=3, height=3)
####SVM ROC-AUC###
library(pROC)
SVM_prediction <- attr(y_pred_SVM, "probabilities")
SVM_predictions <- as.data.frame(SVM_prediction)
ROC_SVM <- roc(test_set$Group, SVM_predictions[,2])
#plot(ROC_rf, col = "red", main = "ROC For Random Forest")
myauc <- paste("AUC=",round(auc(ROC_SVM), digits=2), sep='')
roc_s <- ggroc(ROC_SVM, legacy.axes=TRUE, colour="red") +
  annotate(geom="text", family="sans", x=0.9, y=0.9, label=myauc,size=8) +
  ggtitle("SVM ROC-AUC") +
  theme(panel.grid.major = element_line(color = '#e0e0e0', size=0.2),
        panel.grid.minor = element_blank(),
        panel.background = element_blank(),
        axis.line = element_line(colour = "black"),
        plot.title=element_text( hjust=0.5, vjust=0.5, family="sans", face='bold',size=30),
        axis.title.x = element_text(size = 28,family="sans",face='bold'),
        axis.title.y = element_text(size = 28,family="sans",face='bold'),
        axis.text.x = element_text(family="sans", size=24),
        axis.text.y = element_text(family="sans", size=24)
  )
roc_s
ggsave(roc_s, file="SVM_roc_fin.png", width=7, height=7)


##Randdom Forest####
library("randomForest")
randf <- randomForest(Group ~ ., data=training_set, importance=TRUE,
                      proximity=TRUE)
y_pred_rf <- predict(randf, newdata=test_set[-10], probability=TRUE)
rf_prediction <- predict(randf, test_set, type = "prob")
cm_rf <- confusionMatrix(factor(y_pred_rf), factor(test_set[,10]), dnn = c("Prediction", "Reference"))
plt_rf <- as.data.frame(cm_rf$table)
plt_rf$Prediction <- factor(plt_rf$Prediction, levels=rev(levels(plt_rf$Prediction)))
cm_rf <- ggplot(plt_rf, aes(Reference, Prediction, fill=Freq)) +
  geom_tile(color = "black") +
  ggtitle("Random Forest Confusion Matrix")+
  geom_text(aes(label=Freq),size=6,family="serif") +
  scale_fill_gradient(low="white", high="red",breaks=c(250,500,750)) +
  labs(x = "Reference", y = "Prediction") +
  theme(panel.background = element_blank(),
        axis.text.x = element_text(family="serif", size=8),
        axis.text.y = element_text(family="serif", size=8),
        plot.title=element_text( hjust=0.5, vjust=0, family="serif",face='bold',size=12),
        axis.title.x = element_text(size = 12,family="serif",face='bold'),
        axis.title.y = element_text(size = 12,family="serif",face='bold'),
        legend.position = "none") +
        #legend.key.size = unit(0.5, 'cm'), #change legend key size
        #legend.key.height = unit(0.5, 'cm'), #change legend key height
```

```
	#legend.key.width = unit(0.5, 'cm'), #change legend key width
	#legend.title = element_text(size=10,family="serif"), #change legend title font size
	#legend.text = element_text(size=8,family="serif")) +
  scale_x_discrete(labels= c("WG","UCNE")) +
  scale_y_discrete(labels= c("UCNE","WG"))

cm_rf
ggsave(cm_rf, file="RF_CM.png", width=3, height=3)
library(pROC) #RF ROC-AUC
ROC_rf <- roc(test_set$Group, rf_prediction[,2])
#plot(ROC_rf, col = "red", main = "ROC For Random Forest")
rf_auc <- auc(ROC_rf)
myauc <- paste("AUC=",round(auc(ROC_rf), digits=2), sep='')
roc_rf <- ggroc(ROC_rf, legacy.axes=TRUE, colour="red") +
  annotate(geom="text", family="sans", x=0.9, y=0.9, label=myauc,size=8) +
  ggtitle("RF ROC-AUC") +
  theme(panel.grid.major = element_line(color = '#e0e0e0', size=0.2),
	panel.grid.minor = element_blank(),
	panel.background = element_blank(),
	axis.line = element_line(colour = "black"),
	plot.title=element_text( hjust=0.5, vjust=0.5, family="sans", face='bold',size=30),
	axis.title.x = element_text(size = 28,family="sans",face='bold'),
	axis.title.y = element_text(size = 28,family="sans",face='bold'),
	axis.text.x = element_text(family="sans", size=24),
	axis.text.y = element_text(family="sans", size=24)
  )

roc_rf
ggsave(roc_rf, file="RF_roc_fin.png", width=7, height=7)

###Neural Network####
library(nnet)

(nn1<- nnet( Group  ~ ., data= training_set, size=5, decay = 0.1, MaxNWts = 1000))

nn1.pr.test <- predict(nn1, test_set, type='raw')


nn1.pr.class <- predict(nn1, test_set, type='class')

cm <- confusionMatrix(factor(nn1.pr.class), factor(test_set[,10]), dnn = c("Prediction", "Reference"))

plt <- as.data.frame(cm$table)
plt$Prediction <- factor(plt$Prediction, levels=rev(levels(plt$Prediction)))

NN_CM <- ggplot(plt, aes(Reference, Prediction, fill=Freq)) +
  geom_tile(color = "black") +
  ggtitle("Neural Network Confusion Matrix")+
  geom_text(aes(label=Freq),size=6,family="serif") +
  scale_fill_gradient(low="white", high="red",breaks=c(250,500,750)) +
  labs(x = "Reference", y = "Prediction") +
```

```
  theme(panel.background = element_blank(),
        axis.text.x = element_text(family="serif", size=8),
        axis.text.y = element_text(family="serif", size=8),
        plot.title=element_text( hjust=0.5, vjust=0, family="serif",face='bold',size=12),
        axis.title.x = element_text(size = 12,family="serif",face='bold'),
        axis.title.y = element_text(size = 12,family="serif",face='bold'),
        legend.position = "none") +
        #legend.key.size = unit(0.5, 'cm'), #change legend key size
        #legend.key.height = unit(0.5, 'cm'), #change legend key height
        #legend.key.width = unit(0.5, 'cm'), #change legend key width
        #legend.title = element_text(size=10,family="serif"), #change legend title font size
        #legend.text = element_text(size=8,family="serif")) +
  scale_x_discrete(labels= c("WG","UCNE")) +
  scale_y_discrete(labels= c("UCNE","WG"))

NN_CM
ggsave(NN_CM, file="NN_CM.png", width=3, height=3)

library(pROC) #ANN ROC-AUC
ROC_nn1 <- roc(test_set$Group, nn1.pr.test[,1])
myauc <- paste("AUC=",round(auc(ROC_nn1), digits=2), sep='')
roc_n <- ggroc(ROC_nn1, legacy.axes=TRUE, colour="red") +
  annotate(geom="text", family="sans", x=0.9, y=0.9, label=myauc,size=8) +
  ggtitle("ANN ROC-AUC") +
  theme(panel.grid.major = element_line(color = '#e0e0e0', size=0.2),
        panel.grid.minor = element_blank(),
        panel.background = element_blank(),
        axis.line = element_line(colour = "black"),
        plot.title=element_text( hjust=0.5, vjust=0.5, family="sans", face='bold',size=30),
        axis.title.x = element_text(size = 28,family="sans",face='bold'),
        axis.title.y = element_text(size = 28,family="sans",face='bold'),
        axis.text.x = element_text(family="sans", size=24),
        axis.text.y = element_text(family="sans", size=24)
  )
roc_n
ggsave(roc_n, file="NN_roc_fin.png", width=7, height=7)
```

Python Program

ML_models.py

```
#Program for data prep, Machine learning models, and data visualizations in SciKit Learn
#prepare environment
import pandas as pd #data manipulation
from sklearn.ensemble import RandomForestClassifier
from sklearn import svm
#from sklearn.svm import LinearSVC
from sklearn.metrics import confusion_matrix, classification_report #to evaluate model
from sklearn.preprocessing import StandardScaler # to scale data
from sklearn.model_selection import train_test_split #split
```

```
#loading dataset
path = 'path/input_table.csv'
data = pd.read_csv(path, header =0, index_col = 0)

#print first five rows
data.head()

#info about categories
data.info()
#seperate variables from Group
X = data.drop ('Group', axis = 1)
y = data['Group']
X.head()
y.head()

#seperate train/test data
X_train,X_test,y_train,y_test = train_test_split(X,y,test_size=0.25,random_state=42)

X_train.shape

X_test.shape

#scale data (data in array form)
sc = StandardScaler()
X_train = sc.fit_transform(X_train)
X_test = sc.transform(X_test)
X_test

"""Random Forest Model"""
rfc = RandomForestClassifier(n_estimators = 500)
rfc.fit(X_train,y_train)

#predict using test set
pred_rfc = rfc.predict(X_test)

#print accurarcy report
print(classification_report(y_test,pred_rfc))

#print confusion matrix
print(confusion_matrix(y_test,pred_rfc))

rfc.feature_importances_
print(f"train accuracy: {rfc.score(X_train, y_train):.3f}")
print(f"test accuracy: {rfc.score(X_test, y_test):.3f}")
#calc probabilities
prob_Xtest=rfc.predict_proba(X_test)
#prob array into df
prob_df = pd.DataFrame(prob_Xtest)
print(prob_df)
```

```
"""Neural Networking Model"""
from sklearn.neural_network import MLPClassifier
#create classifier
mlpc = MLPClassifier(hidden_layer_sizes=(5,5,5), max_iter=1000)
#train
mlpc.fit(X_train,y_train)
#predict
pred_mlpc = mlpc.predict(X_test)
#print accurarcy report
print(classification_report(y_test,pred_mlpc))
"""SVM"""
from sklearn import svm
#create classifier
slf=svm.SVC(kernel='rbf',gamma=0.155)
#train model
slf.fit(X_train,y_train)
#prediction
pred_slf=slf.predict(X_test)
print(classification_report(y_test,pred_slf))
```
