# Supplementary material for: Computational identification of ultra-conserved elements in the human genome: a hypothesis on homologous DNA pairing
Source: NAR Genom Bioinform. 2024 Jul 2;6(3):lqae074. doi: 10.1093/nargab/lqae074 (PMC11217675; doi:10.1093/nargab/lqae074)
Supplement: lqae074_Supplemental_Files [file lqae074_supplemental_files.zip › Supplementary File S2.zip!/Supplementary File S2.pdf]

Supplementary File S2

Supplementary Figure S2: Distance distribution for all 256 dinucleotides for UCNE (red), WG (Green), Rand UCNE (Gray), and Rand WG (Yellow).

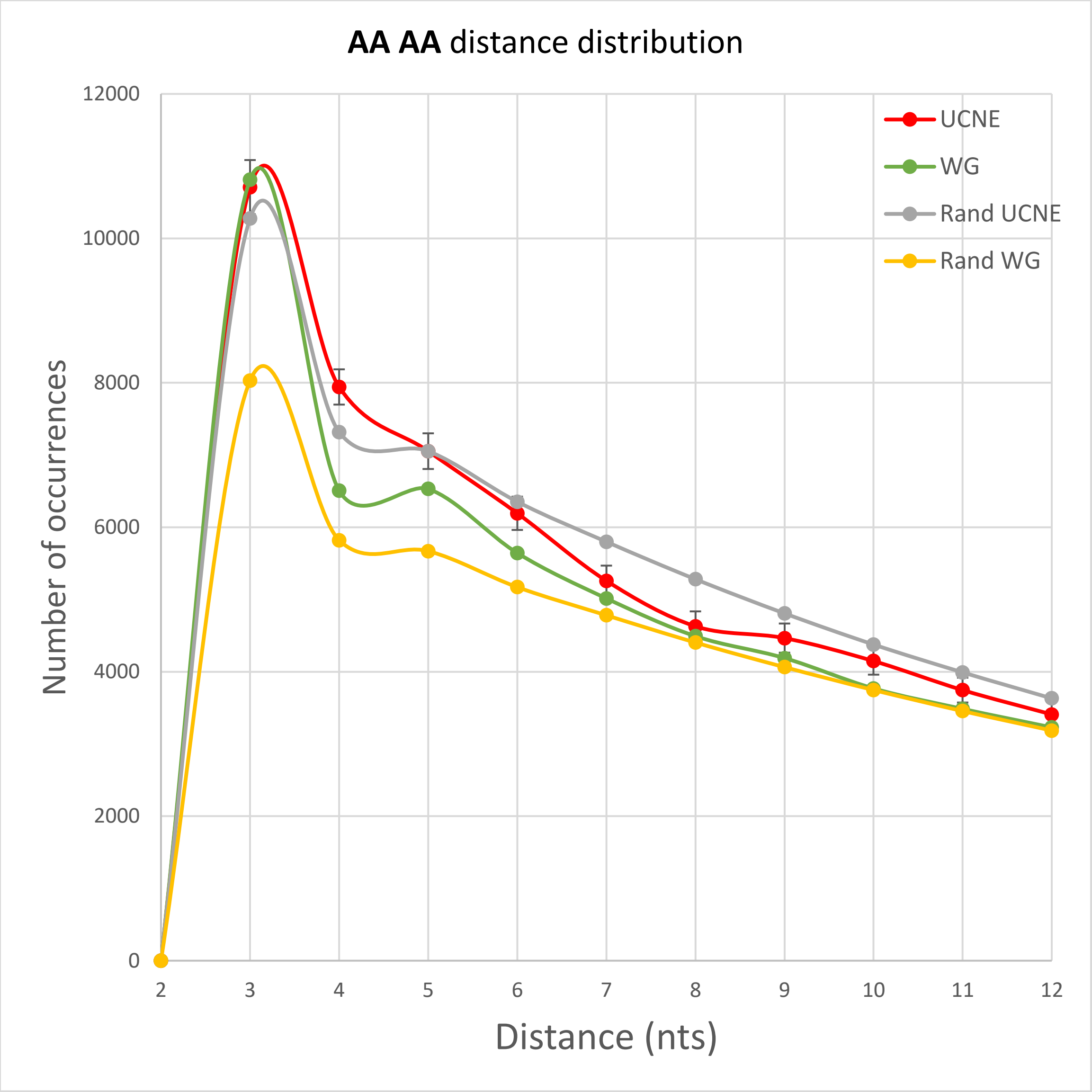

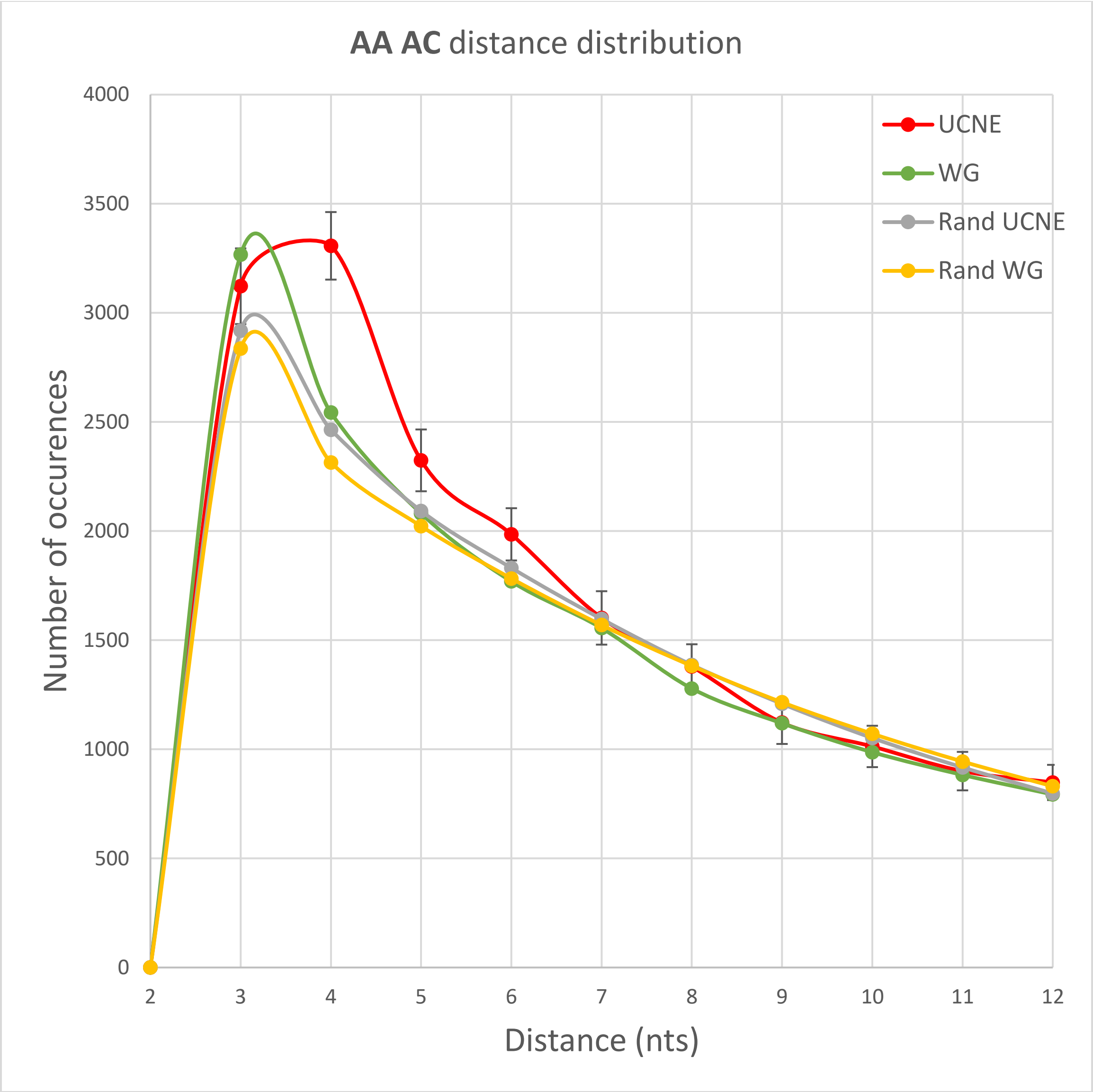

AA AC distance distribution
4000
3500
3000
2500
2000
1500
1000
500
0
Number of occurences
2
3
4
5
6
7
8
9
10
11
12
Distance (nts)
UCNE
WG
Rand UCNE
Rand WG

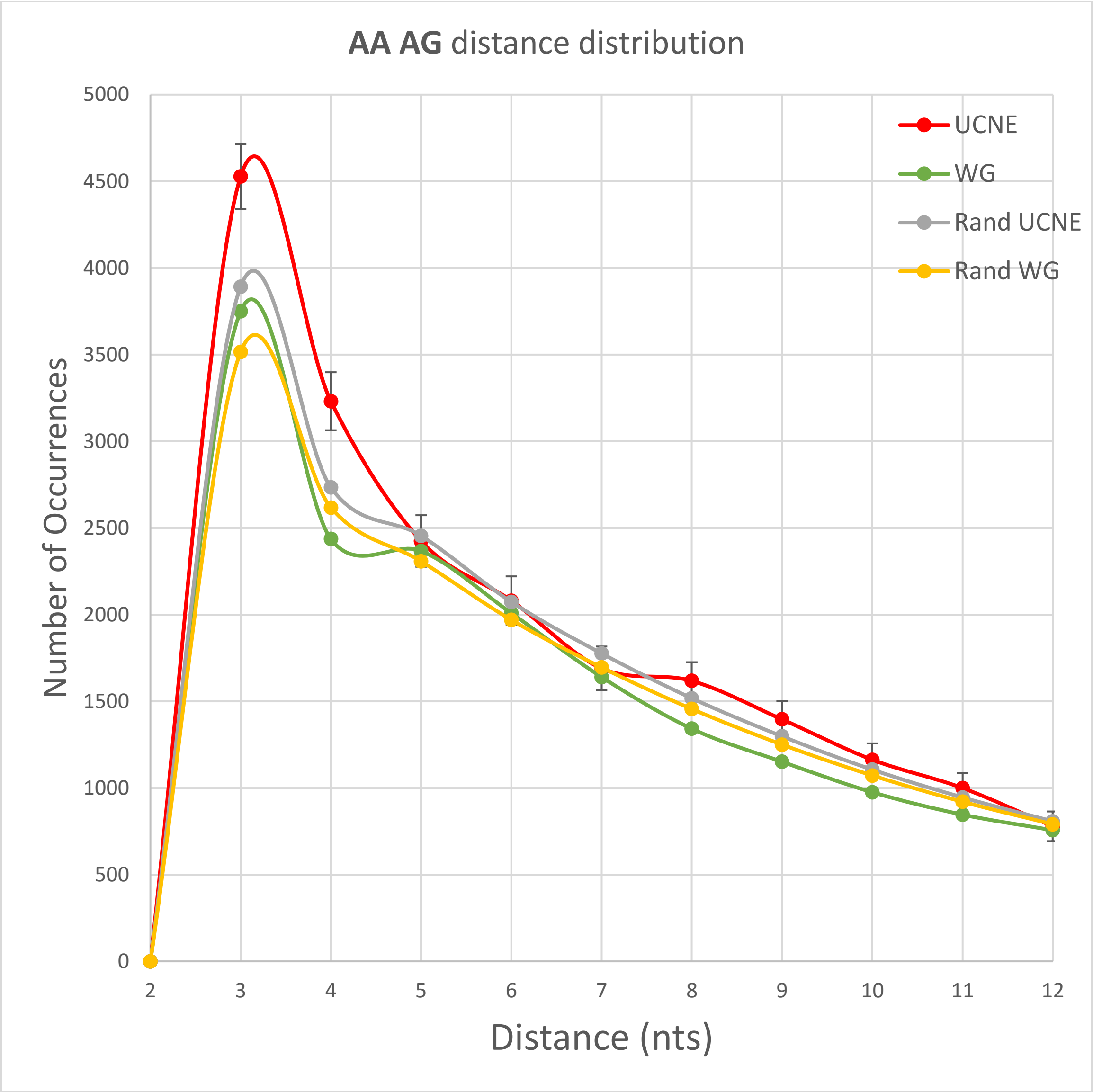

AA AG distance distribution
UCNE
WG
Rand UCNE
Rand WG
Number of Occurrences
Distance (nts)
0
500
1000
1500
2000
2500
3000
3500
4000
4500
5000
2
3
4
5
6
7
8
9
10
11
12

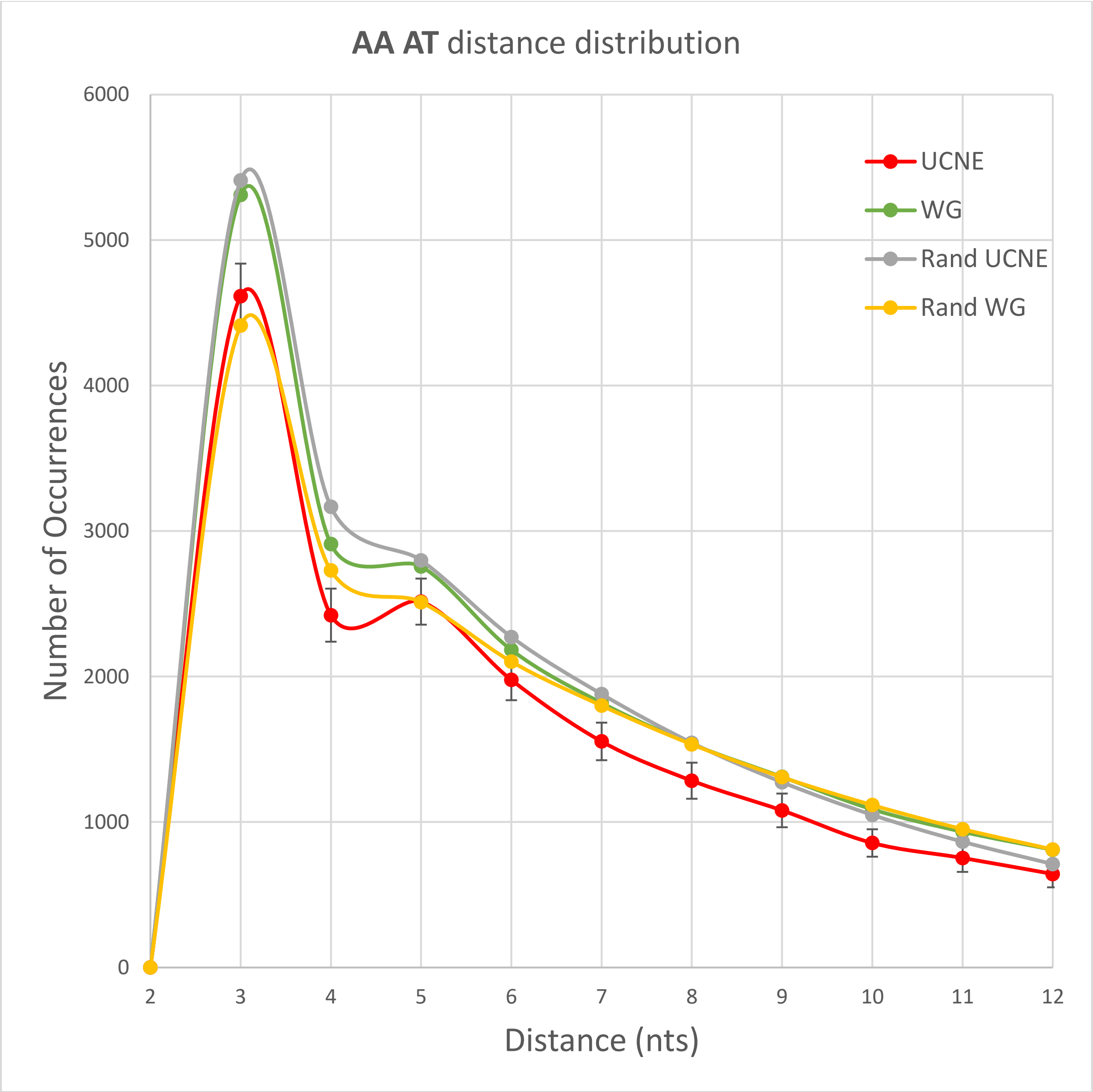
AA AT distance distribution
Number of Occurrences
Distance (nts)
UCNE
WG
Rand UCNE
Rand WG
0
1000
2000
3000
4000
5000
6000
2
3
4
5
6
7
8
9
10
11
12

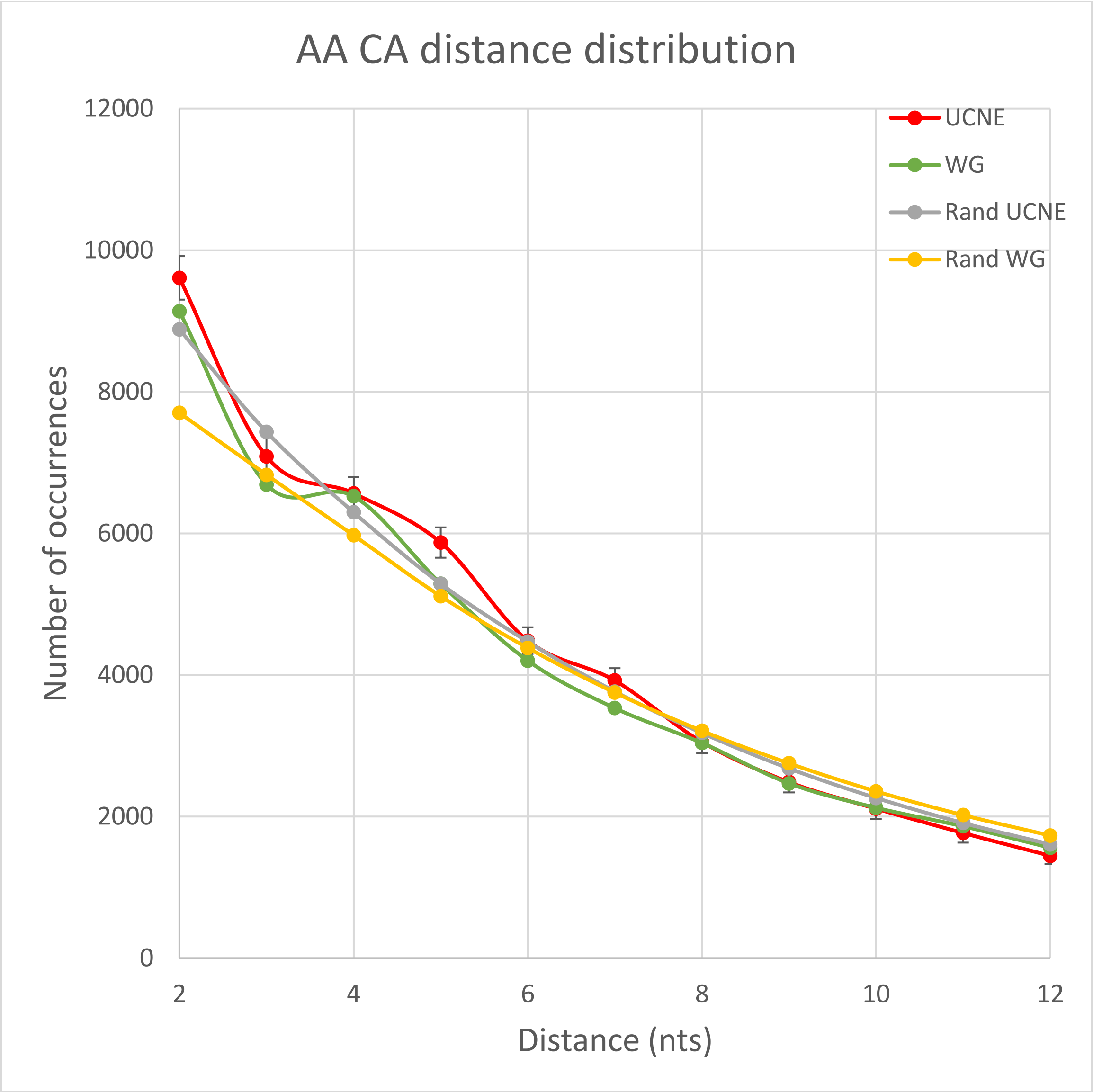
AA CA distance distribution
12000
10000
8000
6000
4000
2000
0
Number of occurrences
2
4
6
8
10
12
Distance (nts)
UCNE
WG
Rand UCNE
Rand WG

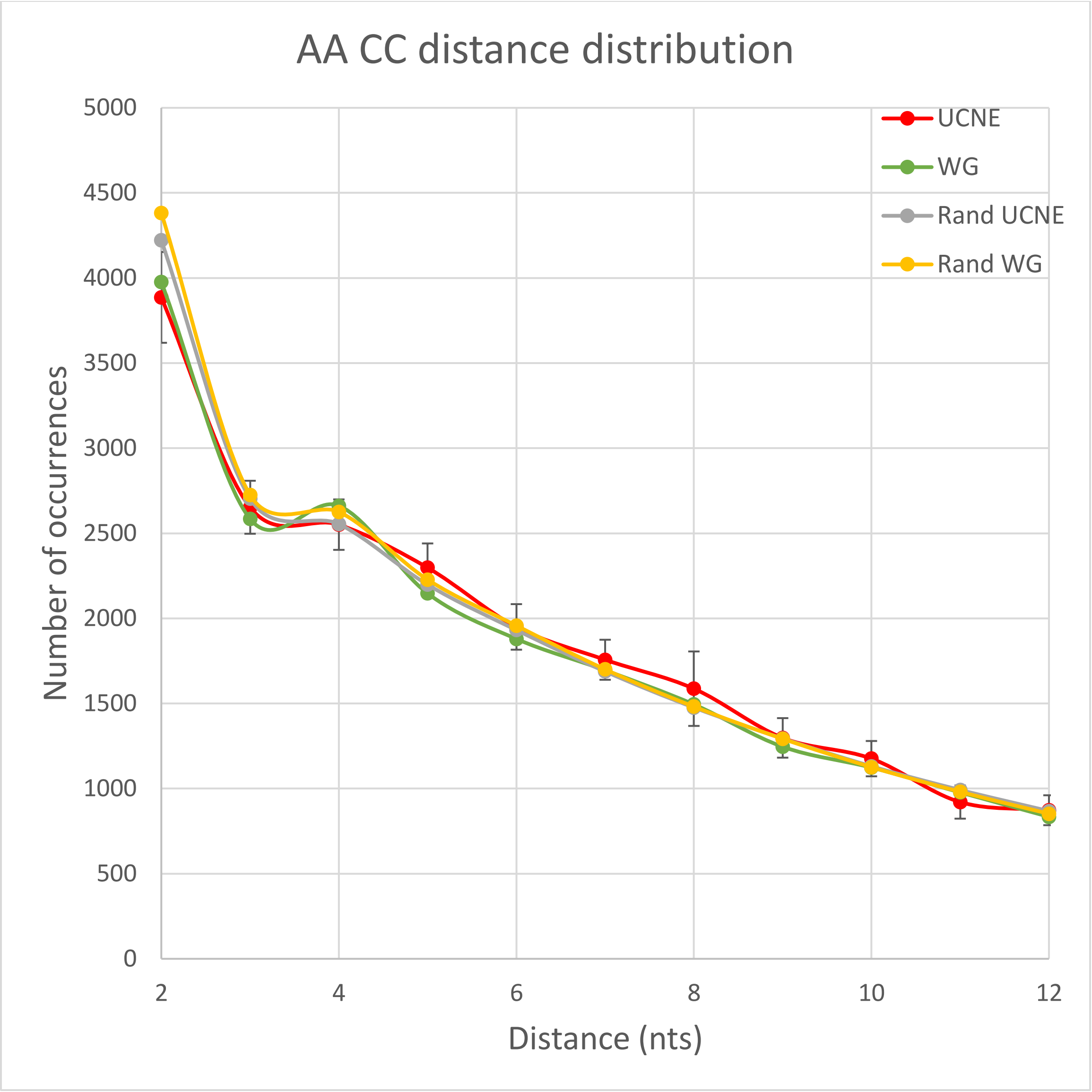
AA CC distance distribution
5000
4500
4000
3500
3000
2500
2000
1500
1000
500
0
Number of occurrences
2
4
6
8
10
12
Distance (nts)
UCNE
WG
Rand UCNE
Rand WG

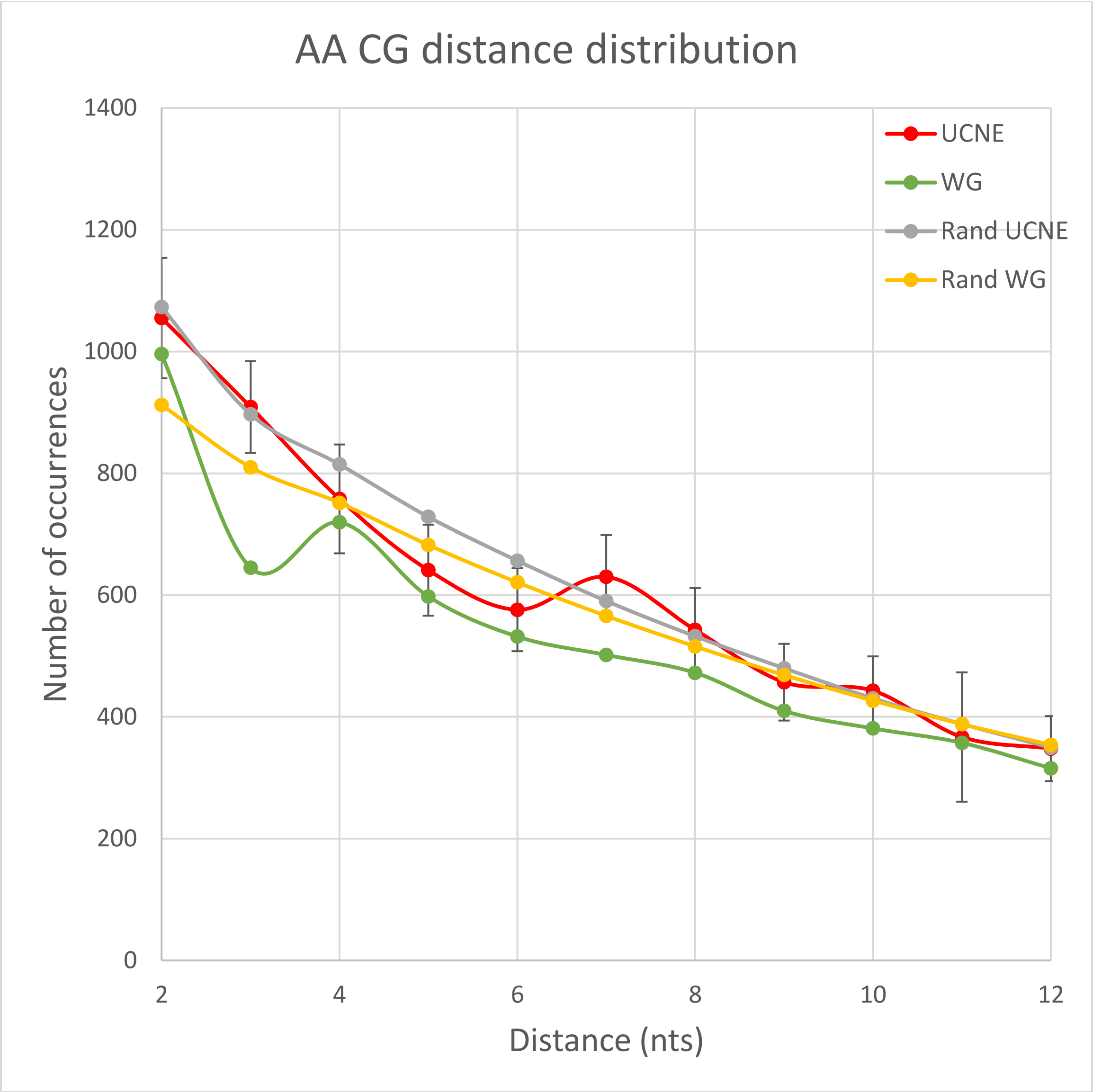
AA CG distance distribution
1400
1200
1000
800
600
400
200
0
2
4
6
8
10
12
Number of occurrences
Distance (nts)
UCNE
WG
Rand UCNE
Rand WG

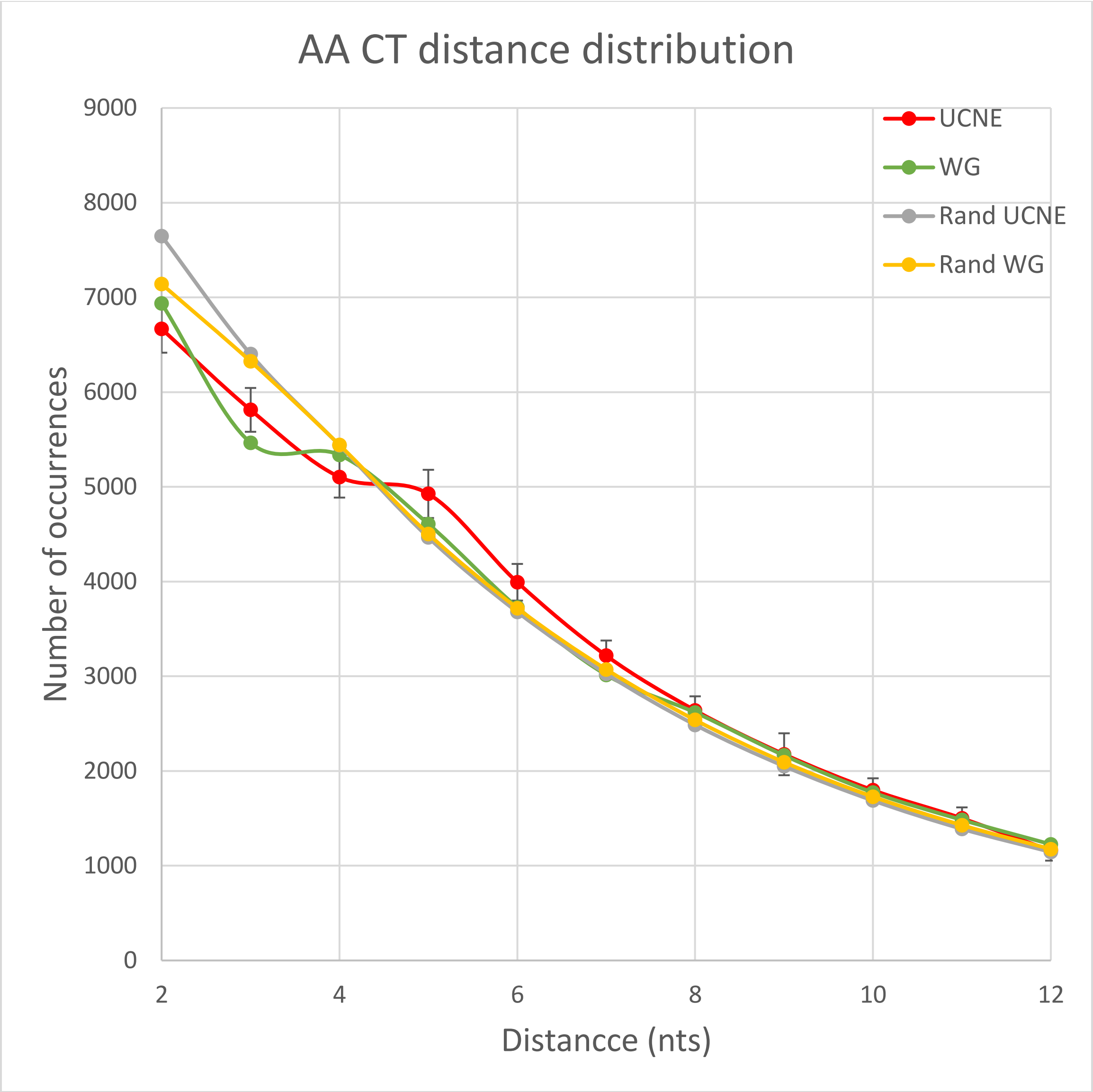

AA CT distance distribution
9000
8000
7000
6000
5000
4000
3000
2000
1000
0
2
4
6
8
10
12
Number of occurrences
Distancce (nts)
UCNE
WG
Rand UCNE
Rand WG

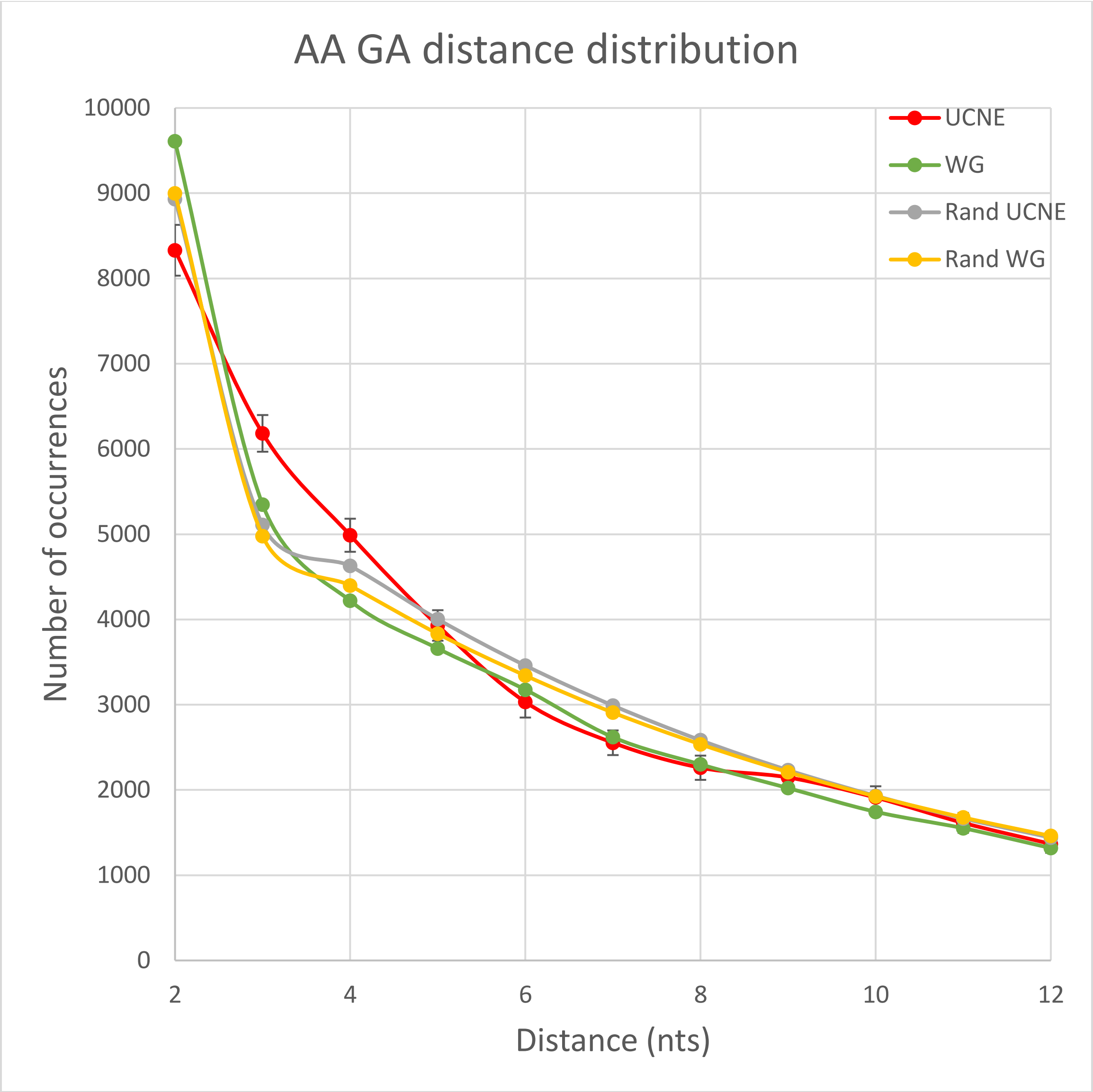
AA GA distance distribution
Number of occurrences
Distance (nts)
UCNE
WG
Rand UCNE
Rand WG
10000
9000
8000
7000
6000
5000
4000
3000
2000
1000
0
2
4
6
8
10
12

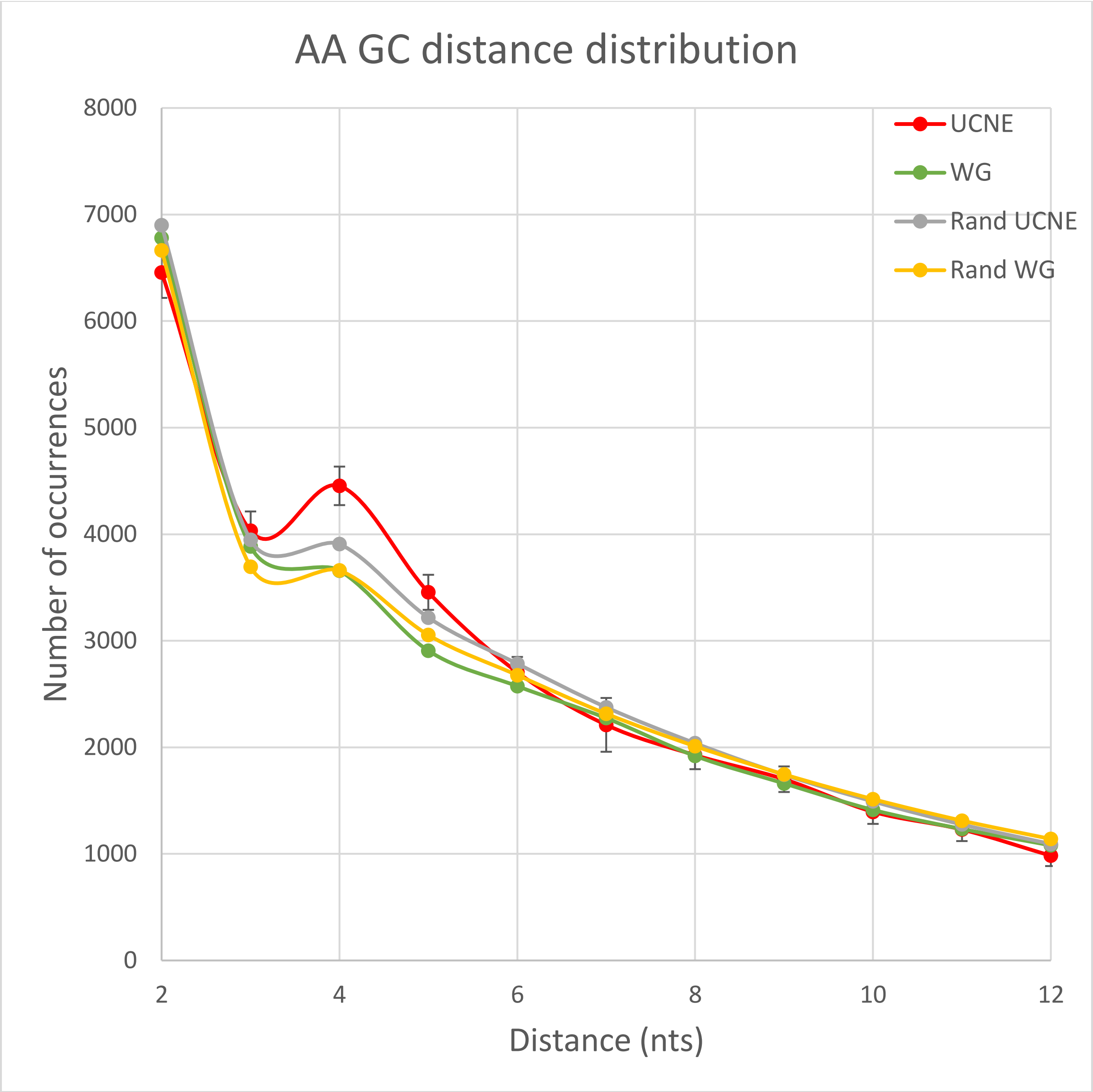
AA GC distance distribution
8000
7000
6000
5000
4000
3000
2000
1000
0
Number of occurrences
2
4
6
8
10
12
Distance (nts)
UCNE
WG
Rand UCNE
Rand WG

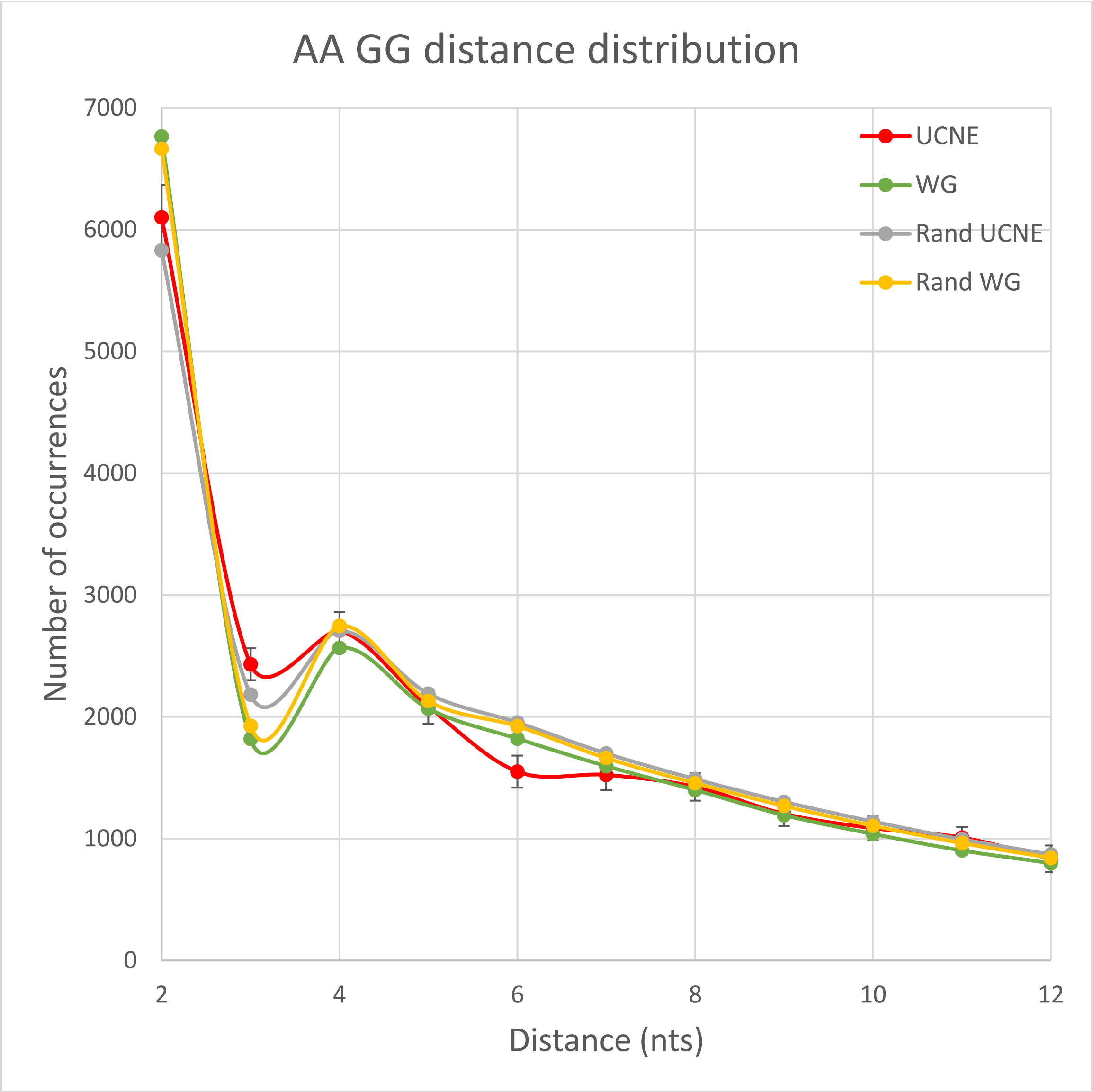

AA GG distance distribution
7000
6000
5000
4000
3000
2000
1000
0
2
4
6
8
10
12
Number of occurrences
Distance (nts)
UCNE
WG
Rand UCNE
Rand WG

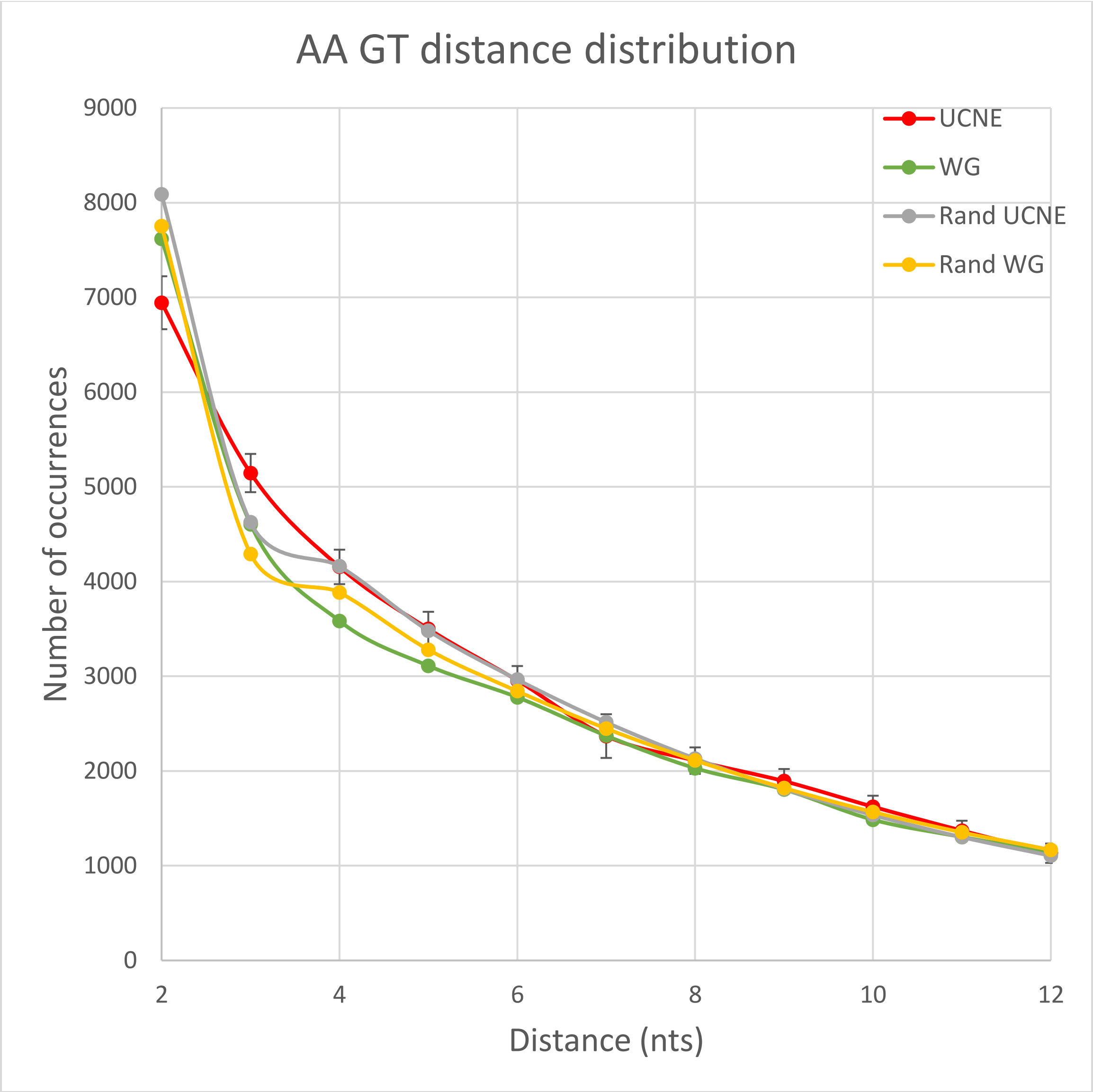
AA GT distance distribution
Number of occurrences
Distance (nts)
UCNE
WG
Rand UCNE
Rand WG
0
1000
2000
3000
4000
5000
6000
7000
8000
9000
2
4
6
8
10
12

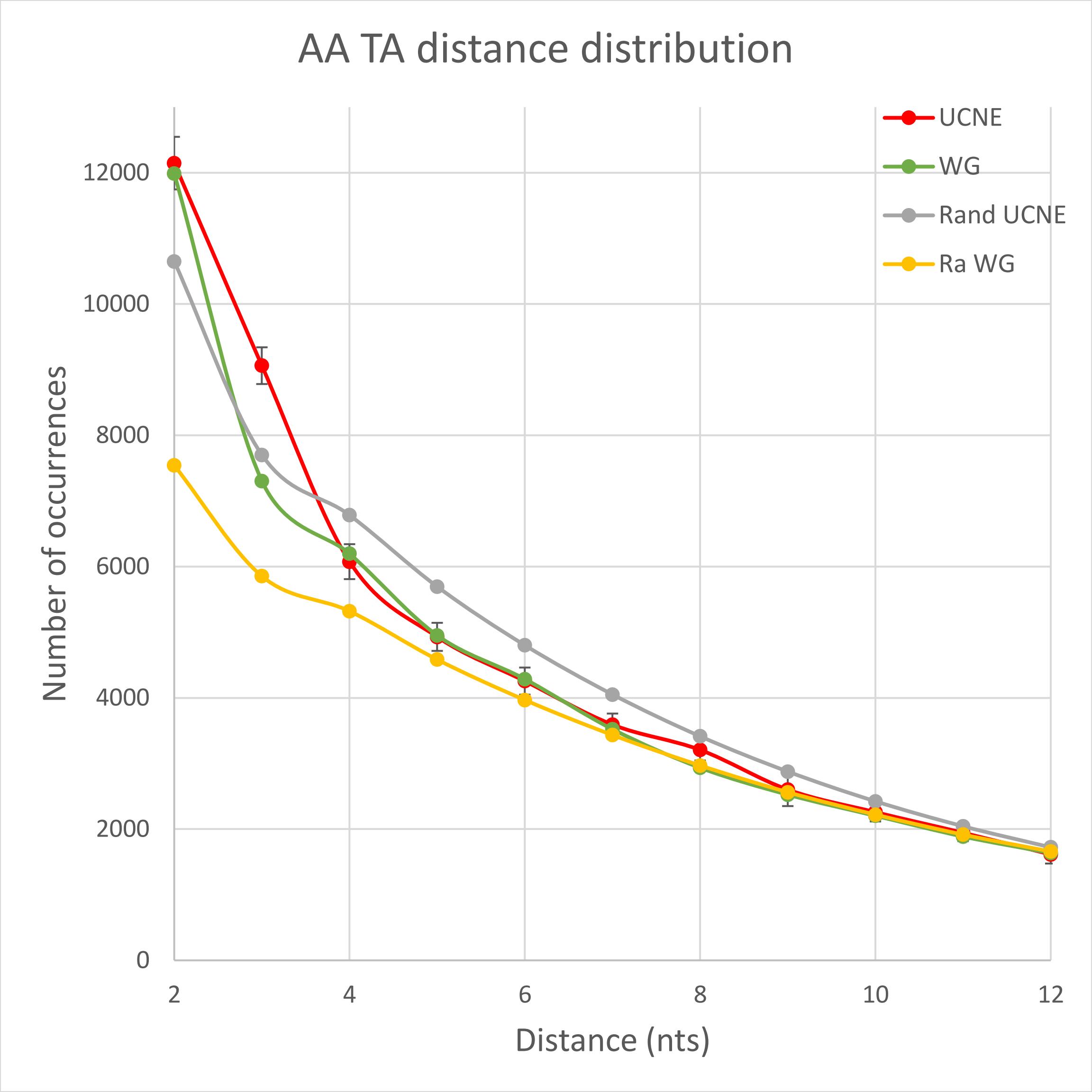
AA TA distance distribution
UCNE
WG
Rand UCNE
Ra WG
Number of occurrences
0
2000
4000
6000
8000
10000
12000
2
4
6
8
10
12
Distance (nts)

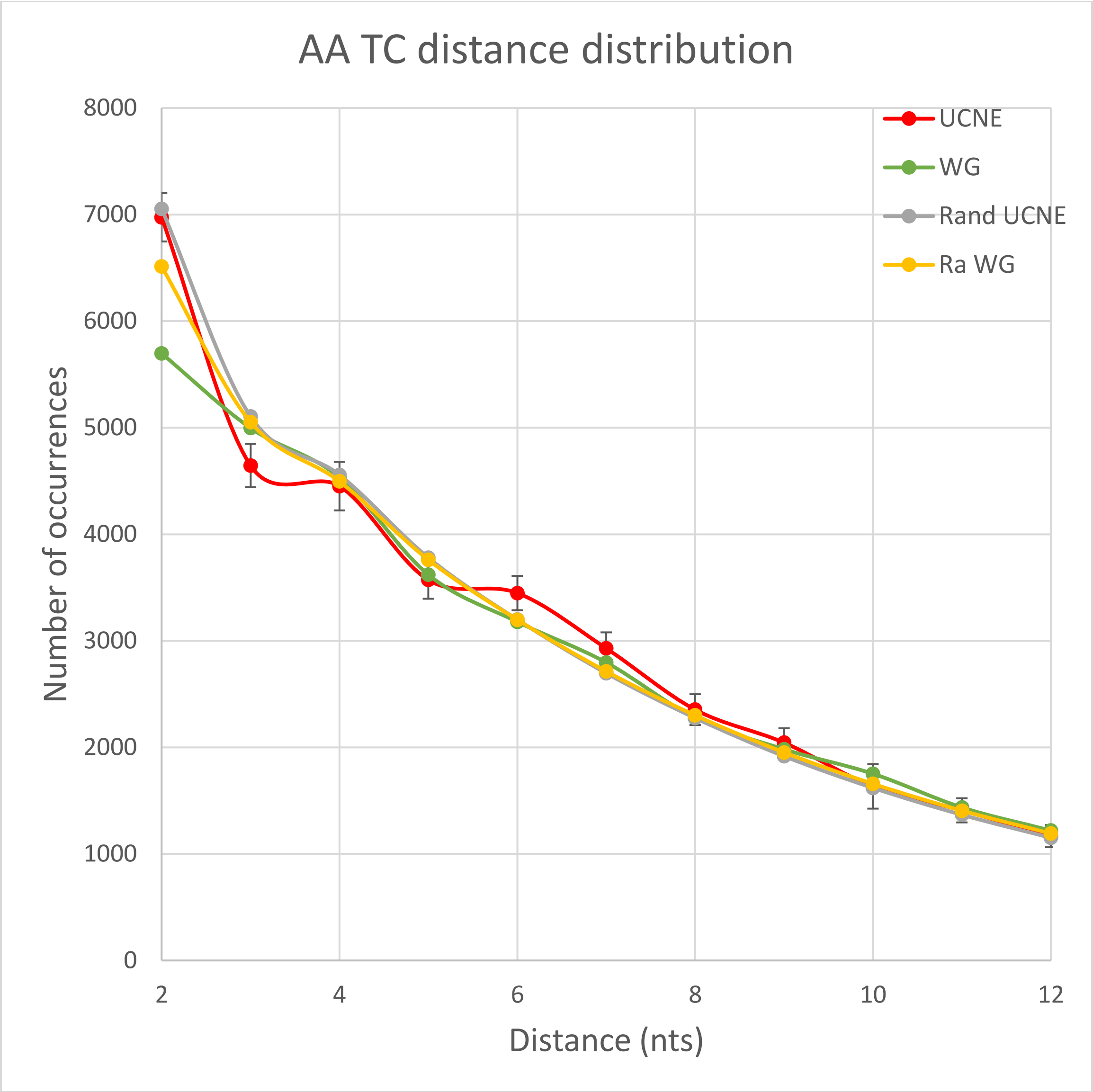
AA TC distance distribution
8000
7000
6000
5000
4000
3000
2000
1000
0
2
4
6
8
10
12
Number of occurrences
Distance (nts)
UCNE
WG
Rand UCNE
Ra WG

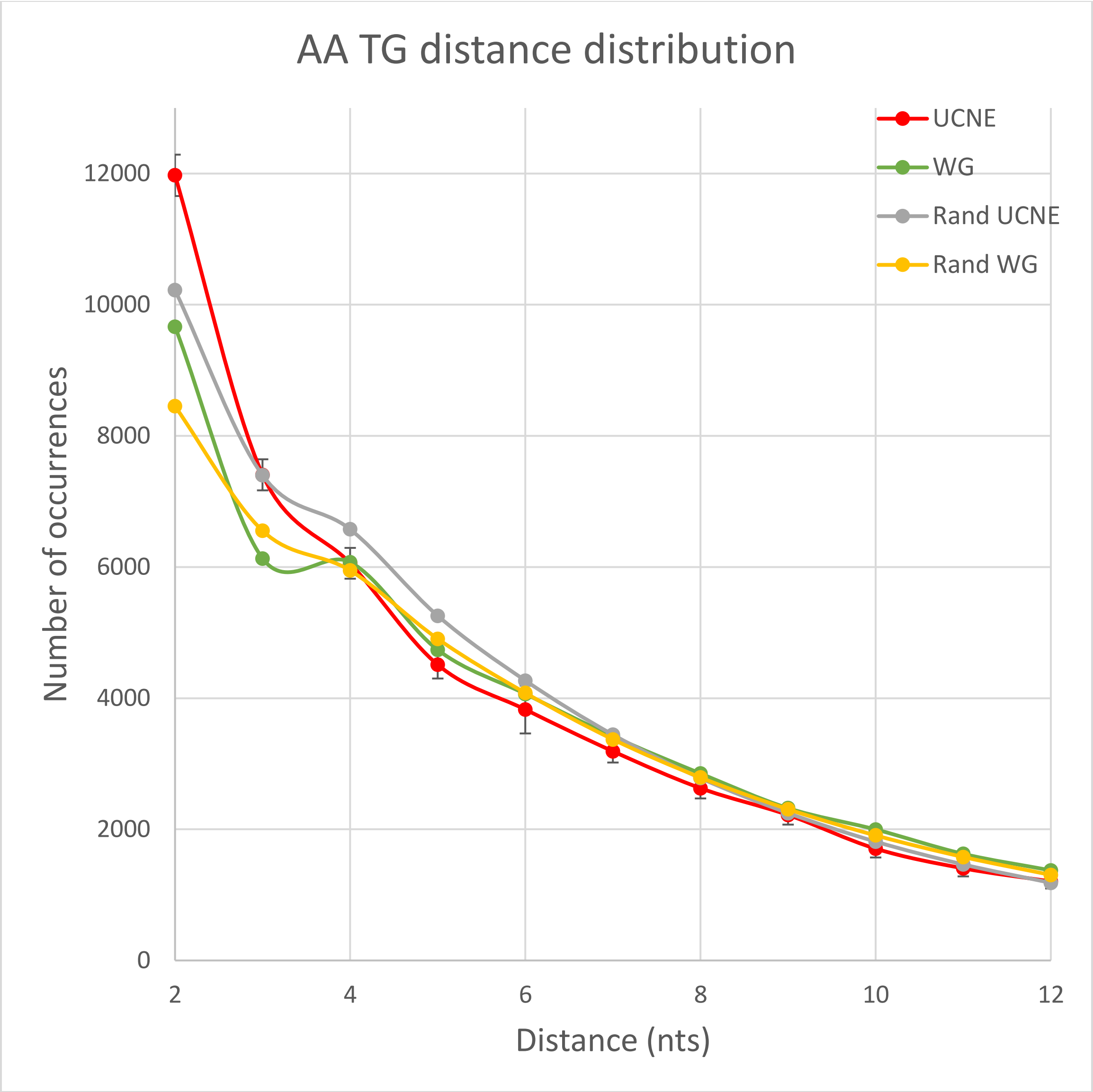
AA TG distance distribution
UCNE
WG
Rand UCNE
Rand WG
Number of occurrences
0
2000
4000
6000
8000
10000
12000
2
4
6
8
10
12
Distance (nts)

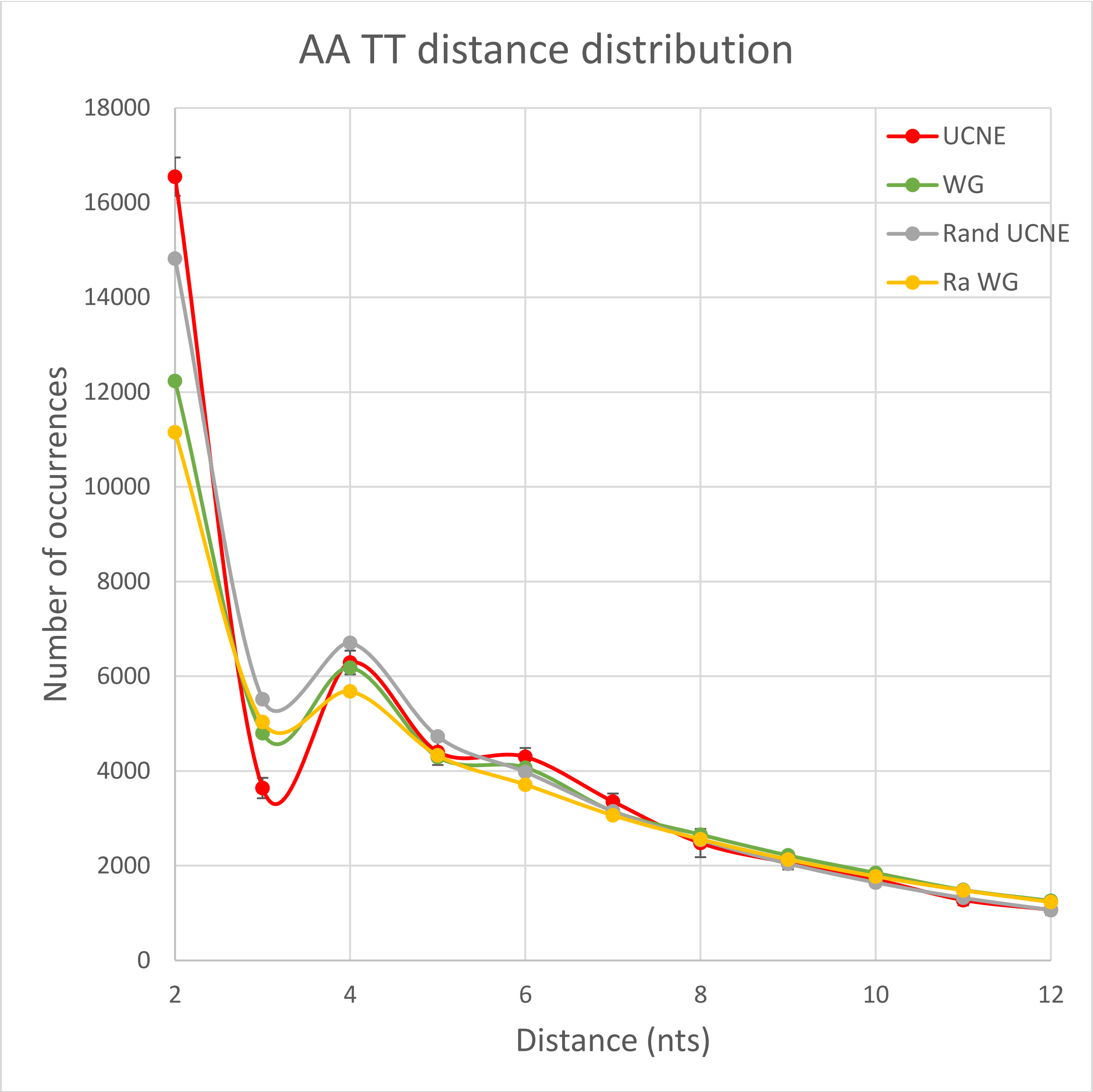

AA TT distance distribution
UCNE
WG
Rand UCNE
Ra WG
Number of occurrences
Distance (nts)
0
2000
4000
6000
8000
10000
12000
14000
16000
18000
2
4
6
8
10
12

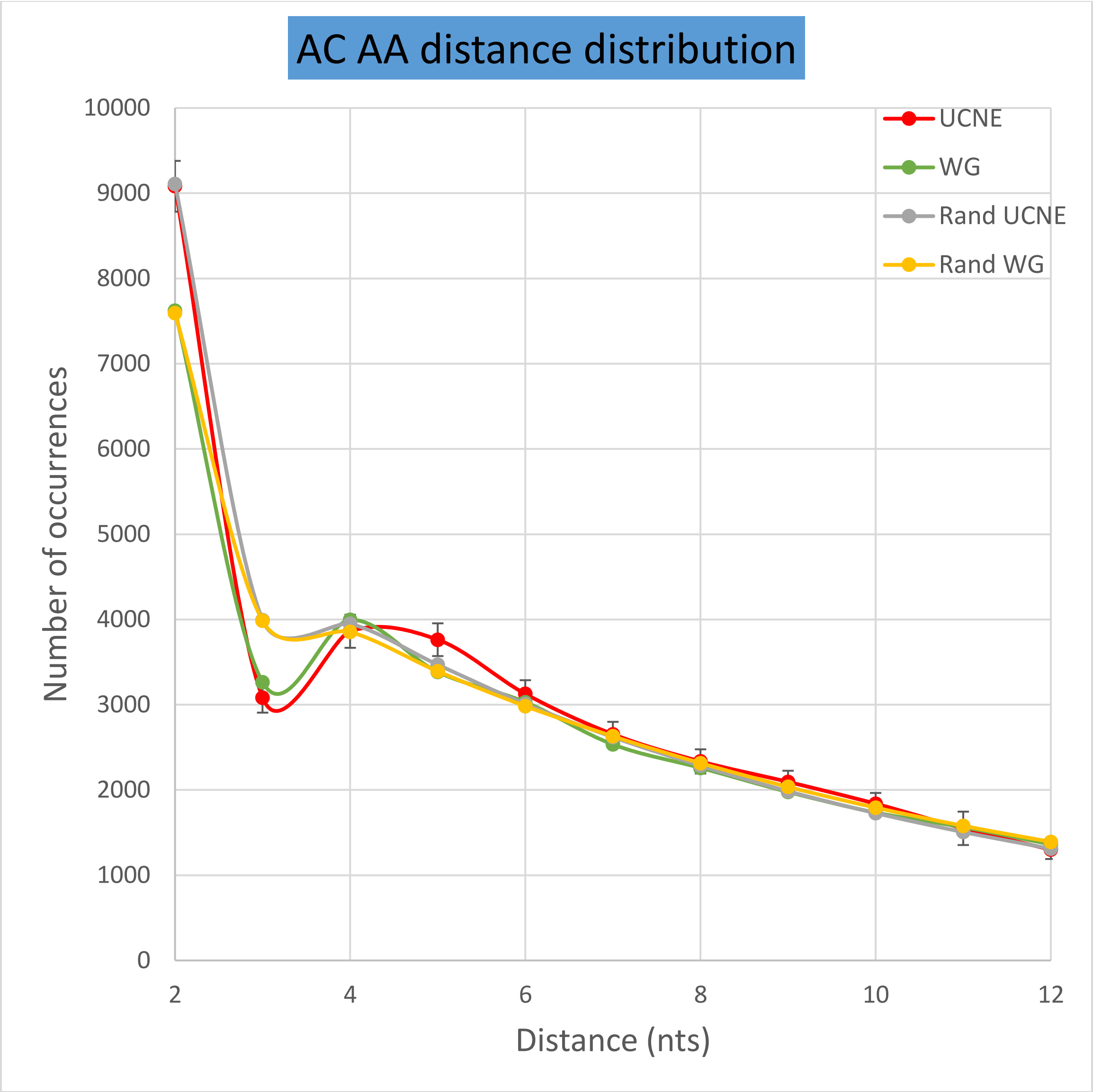
AC AA distance distribution
Number of occurrences
0
1000
2000
3000
4000
5000
6000
7000
8000
9000
10000
2
4
6
8
10
12
Distance (nts)
UCNE
WG
Rand UCNE
Rand WG

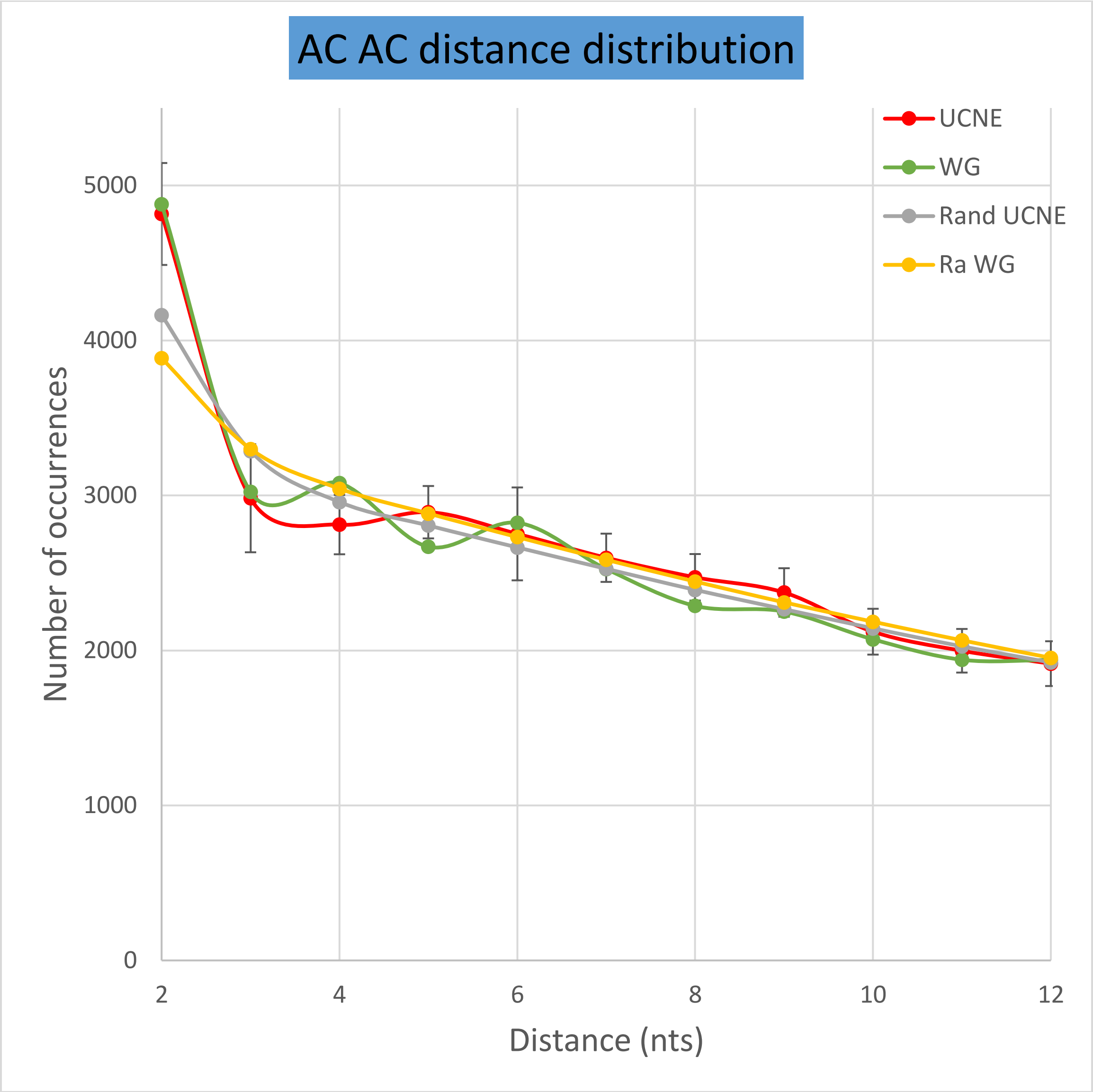
AC AC distance distribution
UCNE
WG
Rand UCNE
Ra WG
Number of occurrences
5000
4000
3000
2000
1000
0
2
4
6
8
10
12
Distance (nts)

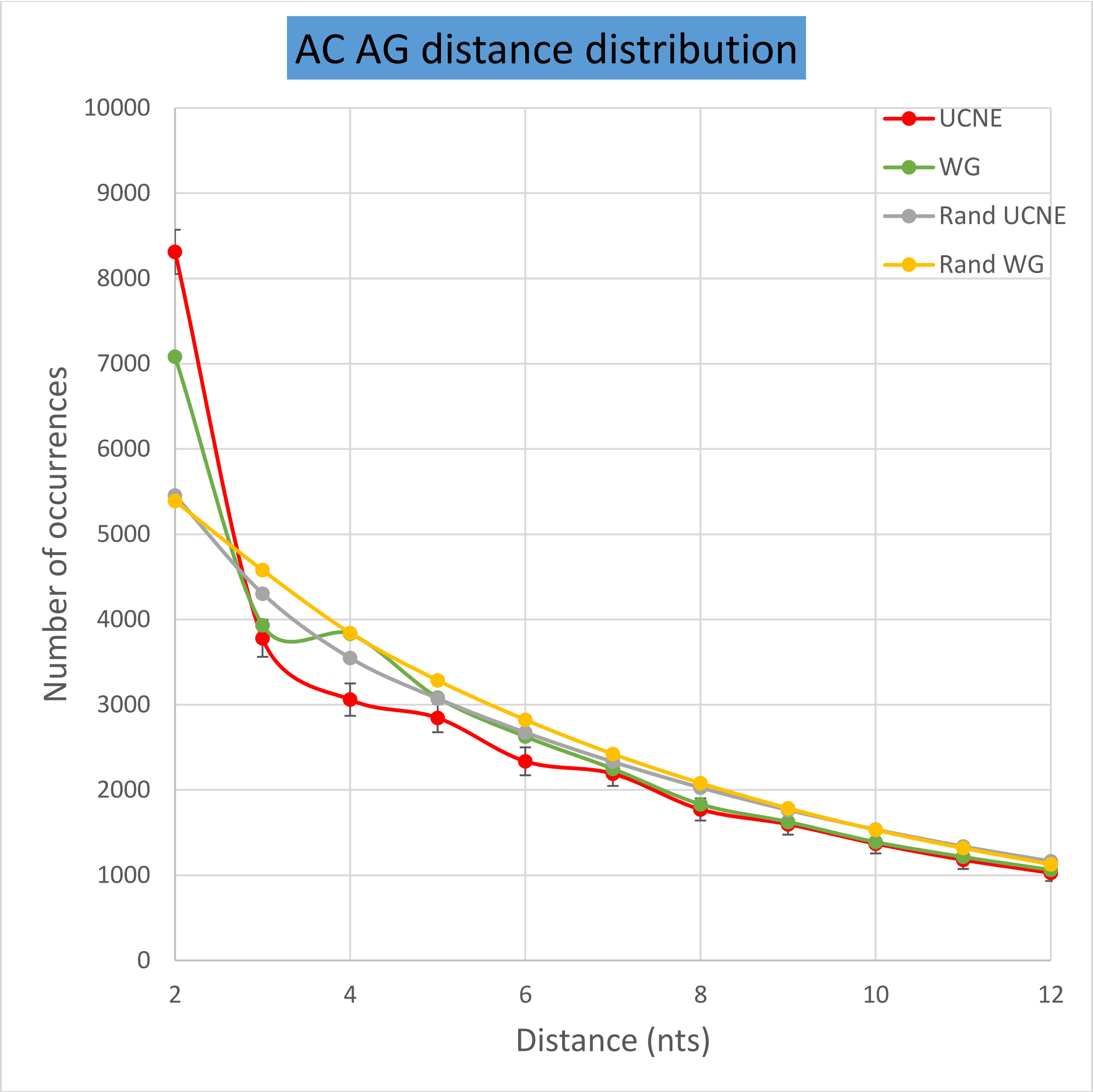
AC AG distance distribution
UCNE
WG
Rand UCNE
Rand WG
Number of occurrences
10000
9000
8000
7000
6000
5000
4000
3000
2000
1000
0
2
4
6
8
10
12
Distance (nts)

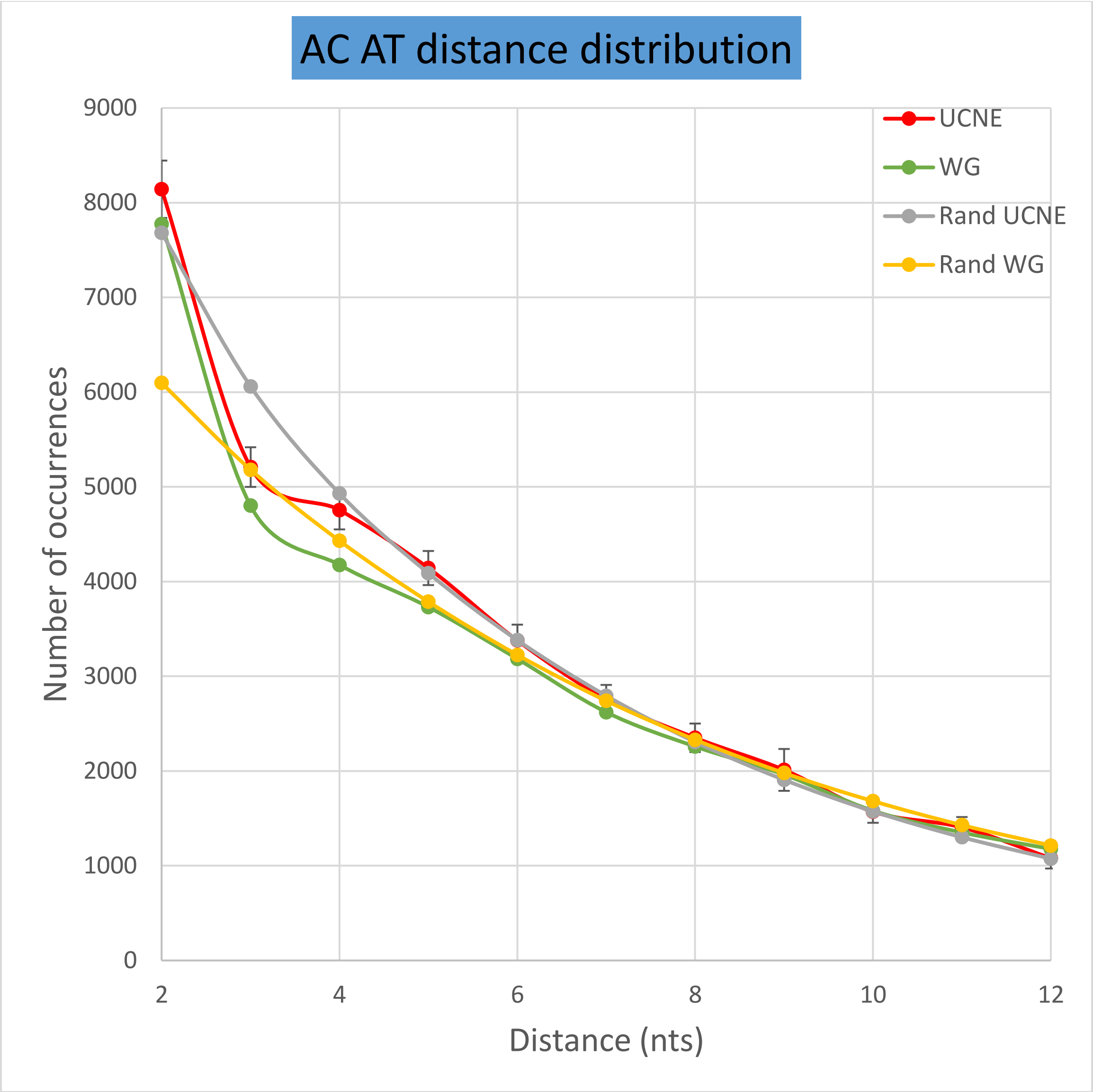
AC AT distance distribution
Number of occurrences
Distance (nts)
UCNE
WG
Rand UCNE
Rand WG
0
1000
2000
3000
4000
5000
6000
7000
8000
9000
2
4
6
8
10
12

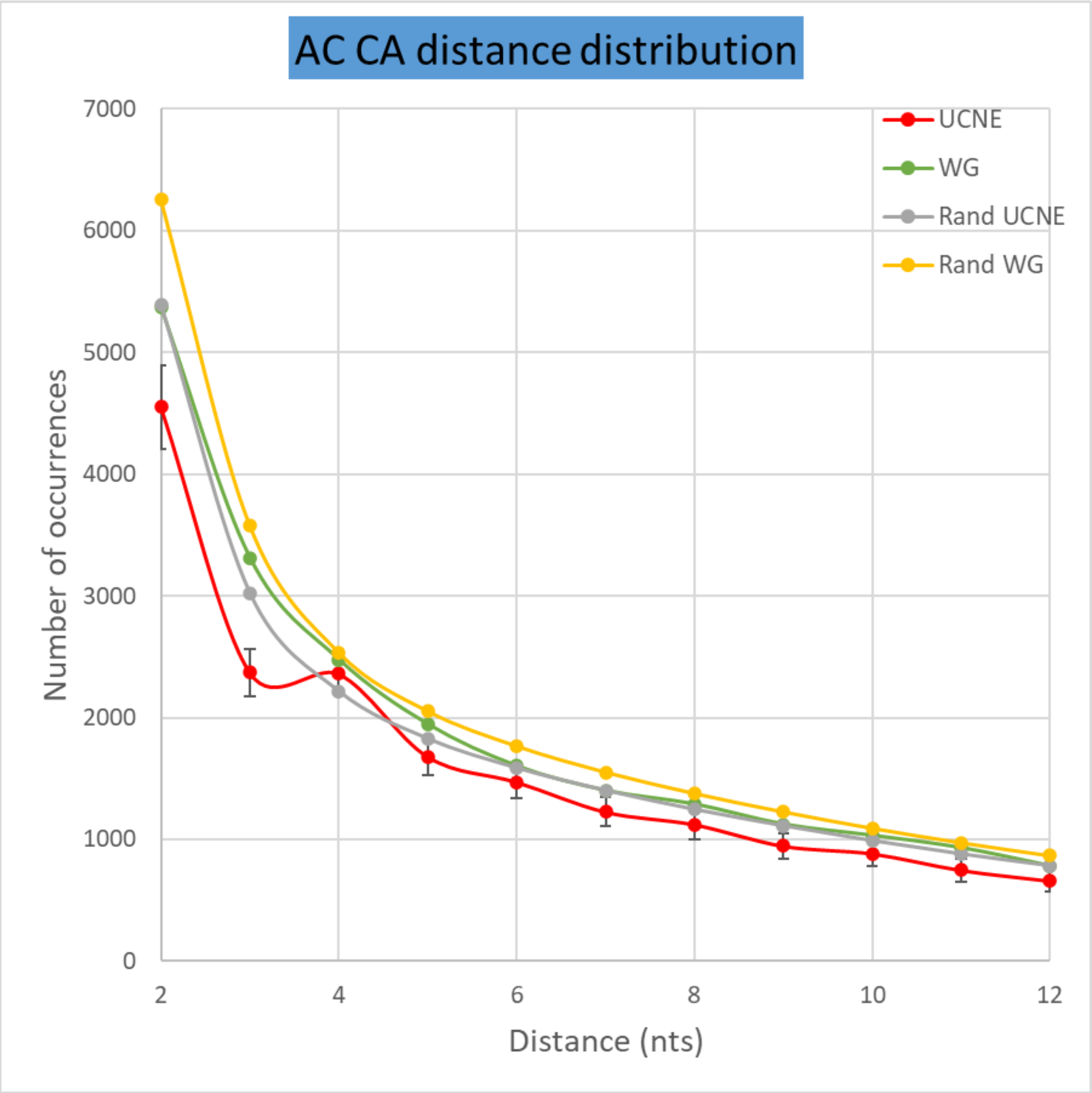
AC CA distance distribution
UCNE
WG
Rand UCNE
Rand WG
Number of occurrences
0
1000
2000
3000
4000
5000
6000
7000
2
4
6
8
10
12
Distance (nts)

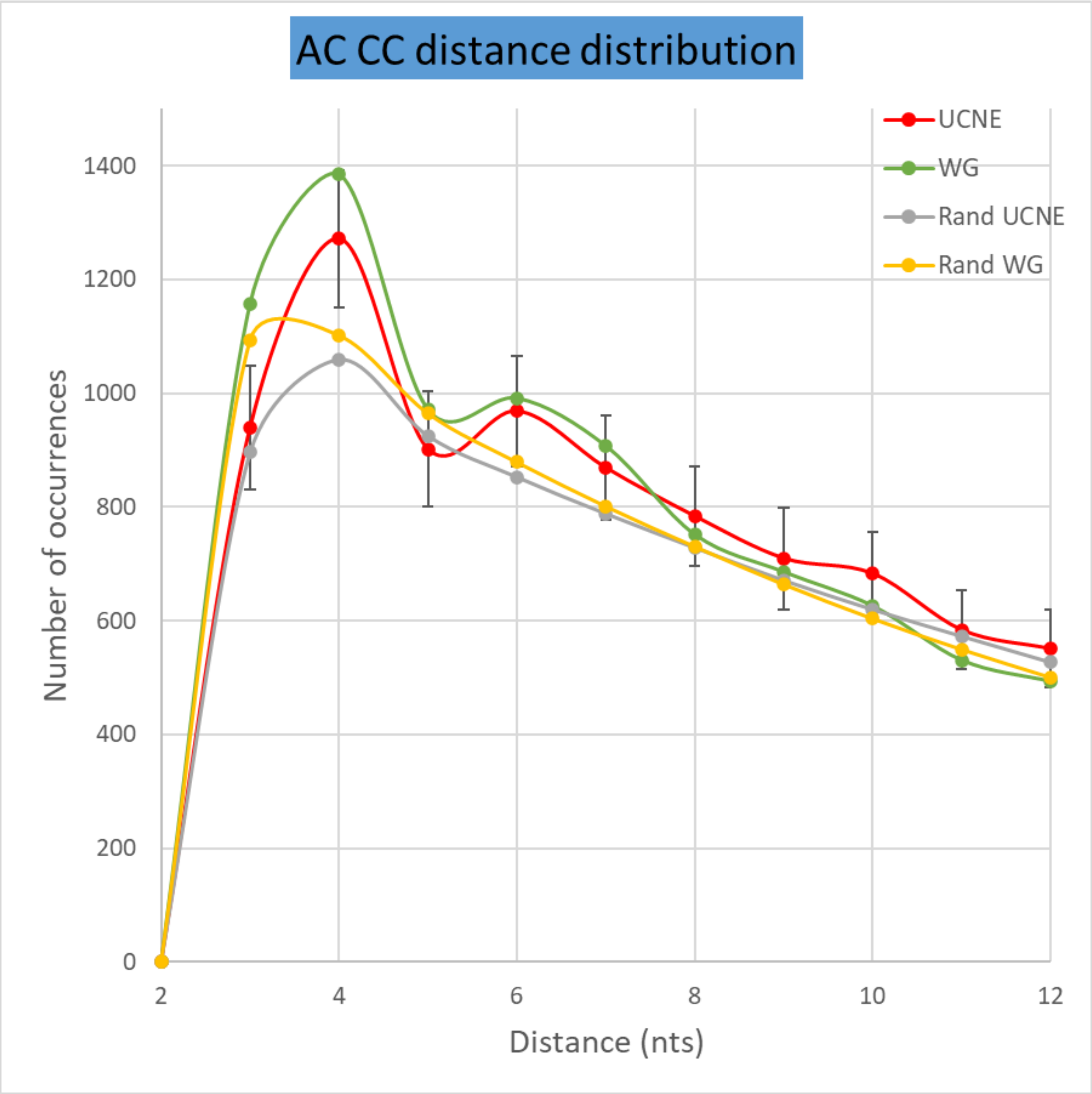
AC CC distance distribution
UCNE
WG
Rand UCNE
Rand WG
Number of occurrences
0
200
400
600
800
1000
1200
1400
2
4
6
8
10
12
Distance (nts)

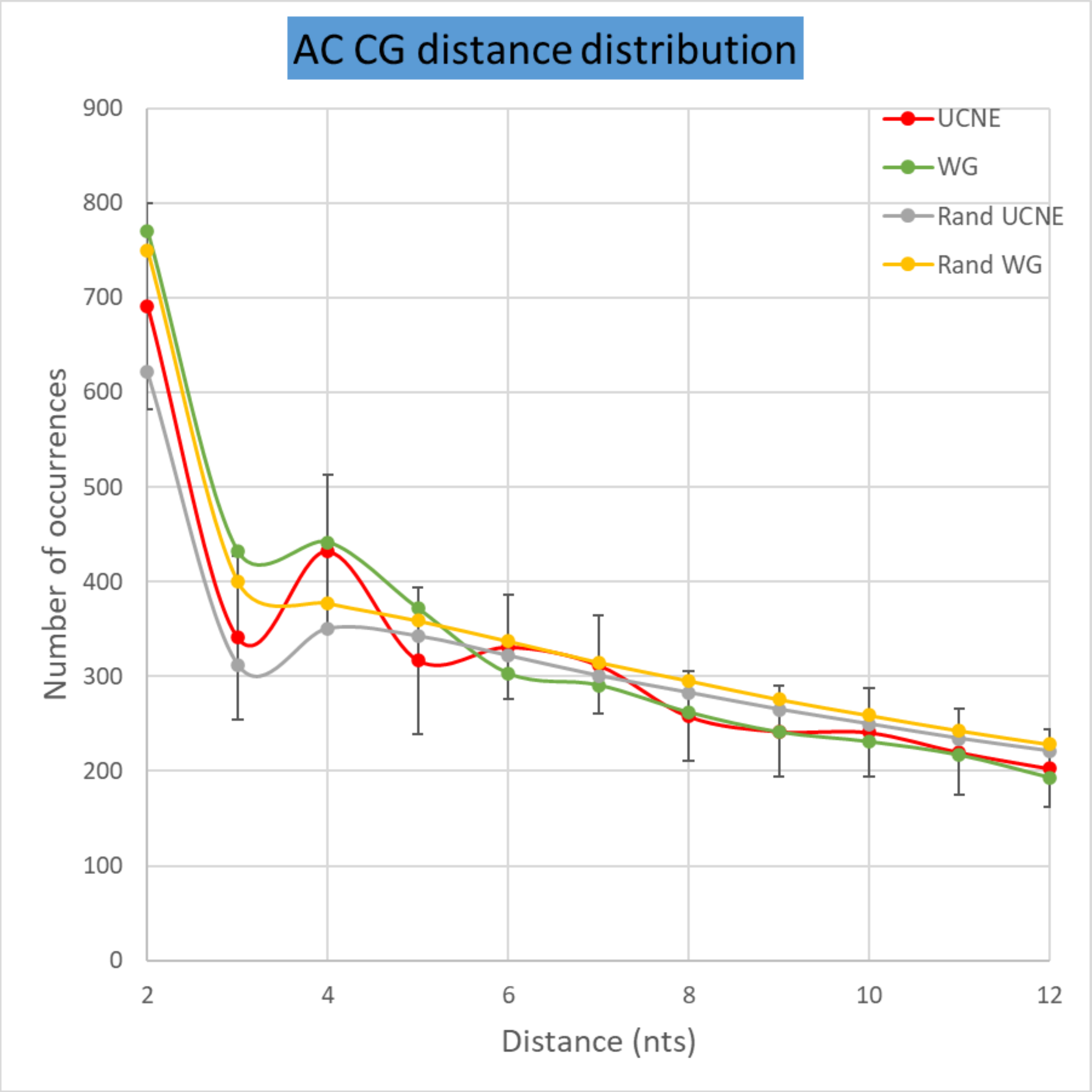

AC CG distance distribution
UCNE
WG
Rand UCNE
Rand WG
Number of occurrences
900
800
700
600
500
400
300
200
100
0
2
4
6
8
10
12
Distance (nts)

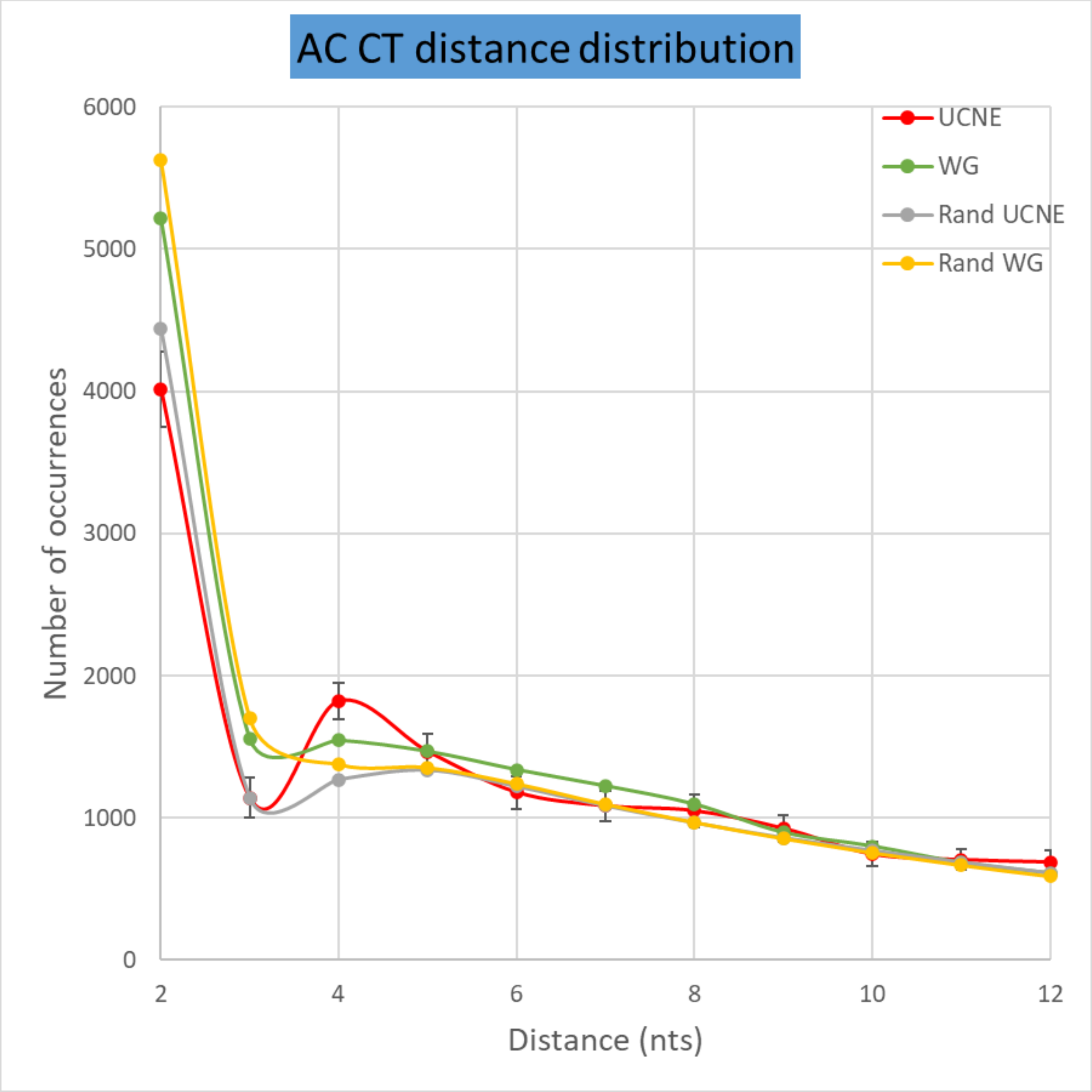
AC CT distance distribution
Number of occurrences
Distance (nts)
6000
5000
4000
3000
2000
1000
0
2
4
6
8
10
12
UCNE
WG
Rand UCNE
Rand WG

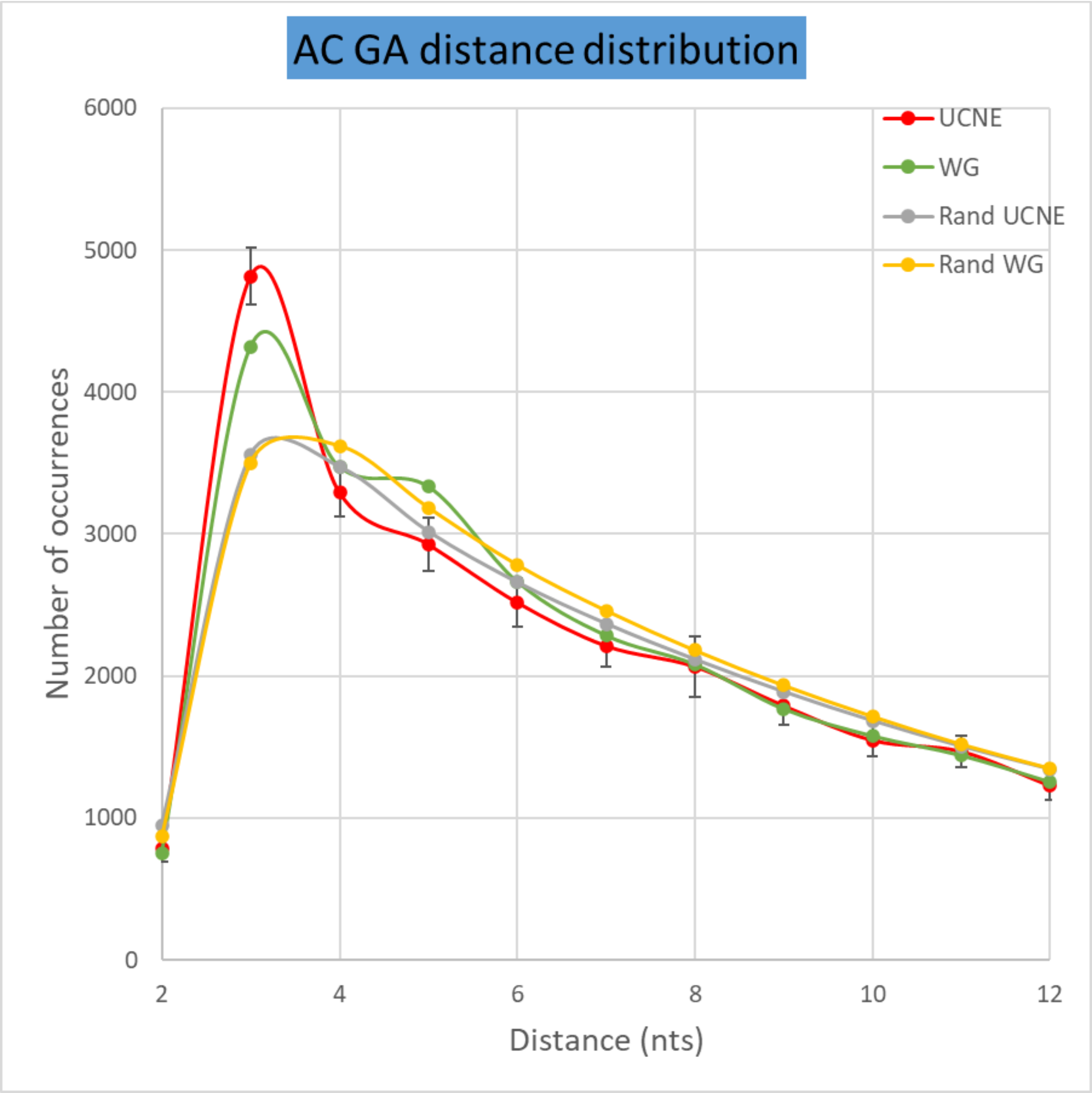
AC GA distance distribution
6000
5000
4000
3000
2000
1000
0
2
4
6
8
10
12
Number of occurrences
Distance (nts)
UCNE
WG
Rand UCNE
Rand WG

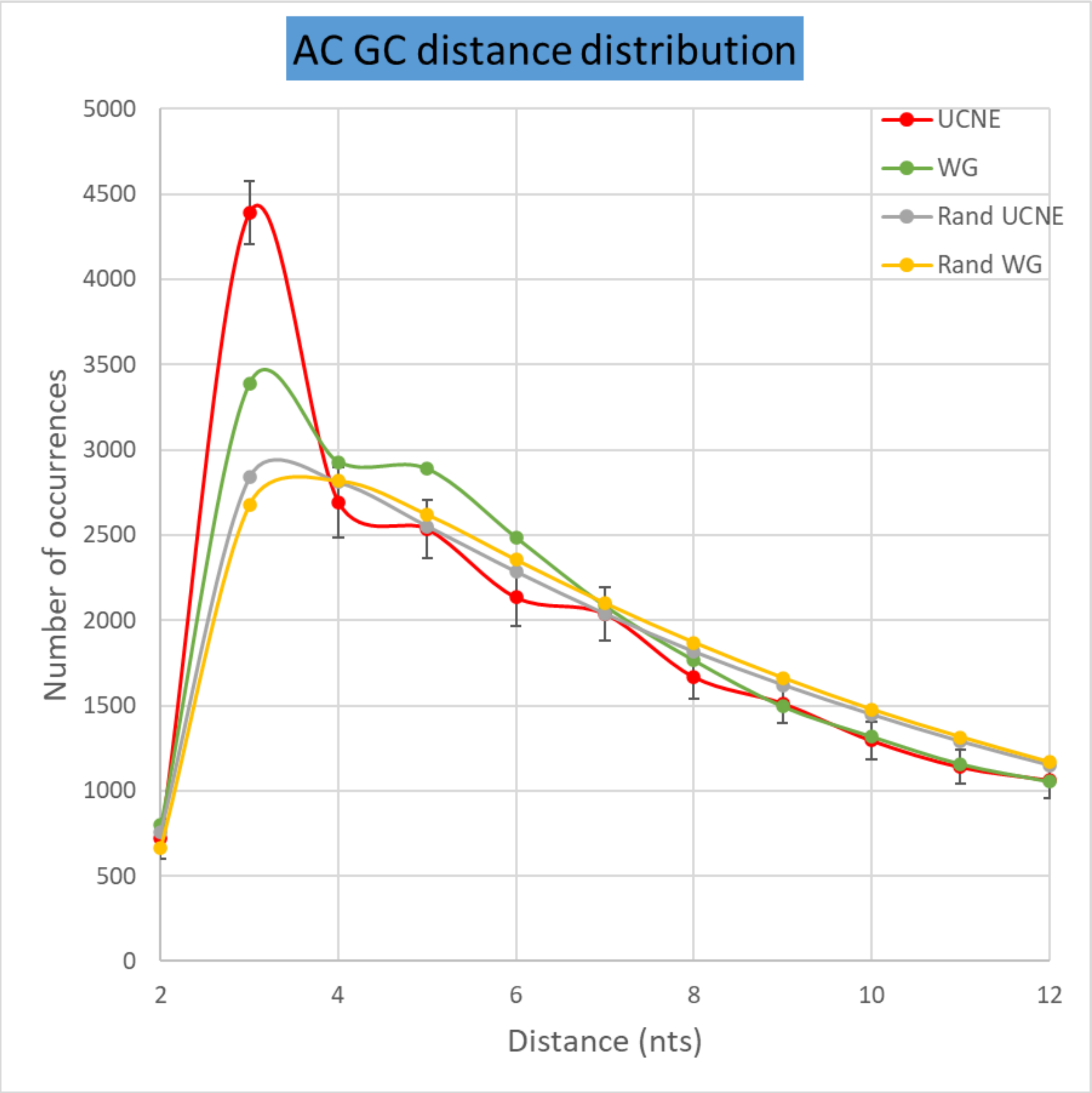
AC GC distance distribution
Number of occurrences
Distance (nts)
UCNE
WG
Rand UCNE
Rand WG
5000
4500
4000
3500
3000
2500
2000
1500
1000
500
0
2
4
6
8
10
12

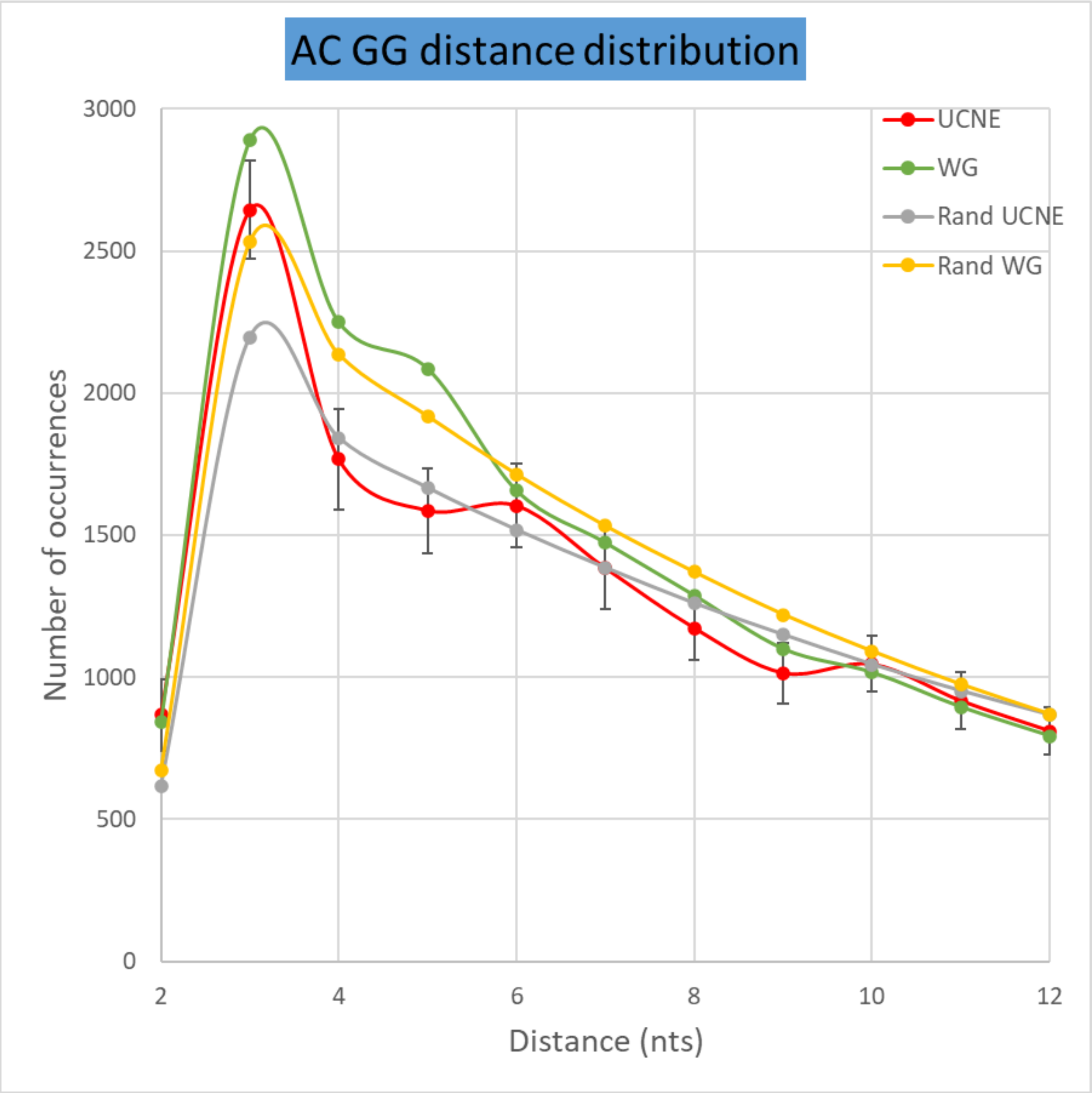
AC GG distance distribution
UCNE
WG
Rand UCNE
Rand WG
Number of occurrences
3000
2500
2000
1500
1000
500
0
2
4
6
8
10
12
Distance (nts)

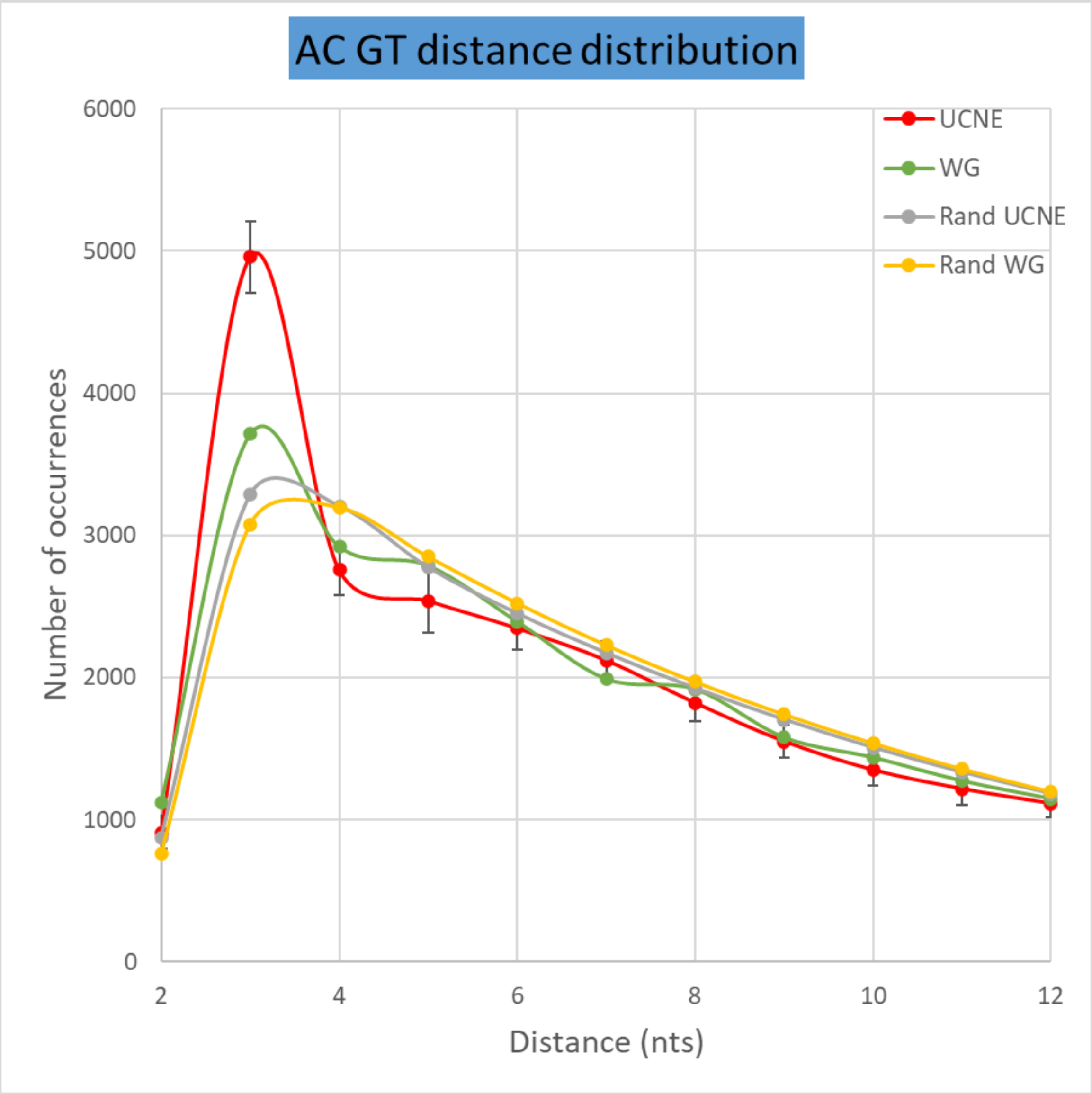

AC GT distance distribution
UCNE
WG
Rand UCNE
Rand WG
Number of occurrences
0
1000
2000
3000
4000
5000
6000
2
4
6
8
10
12
Distance (nts)

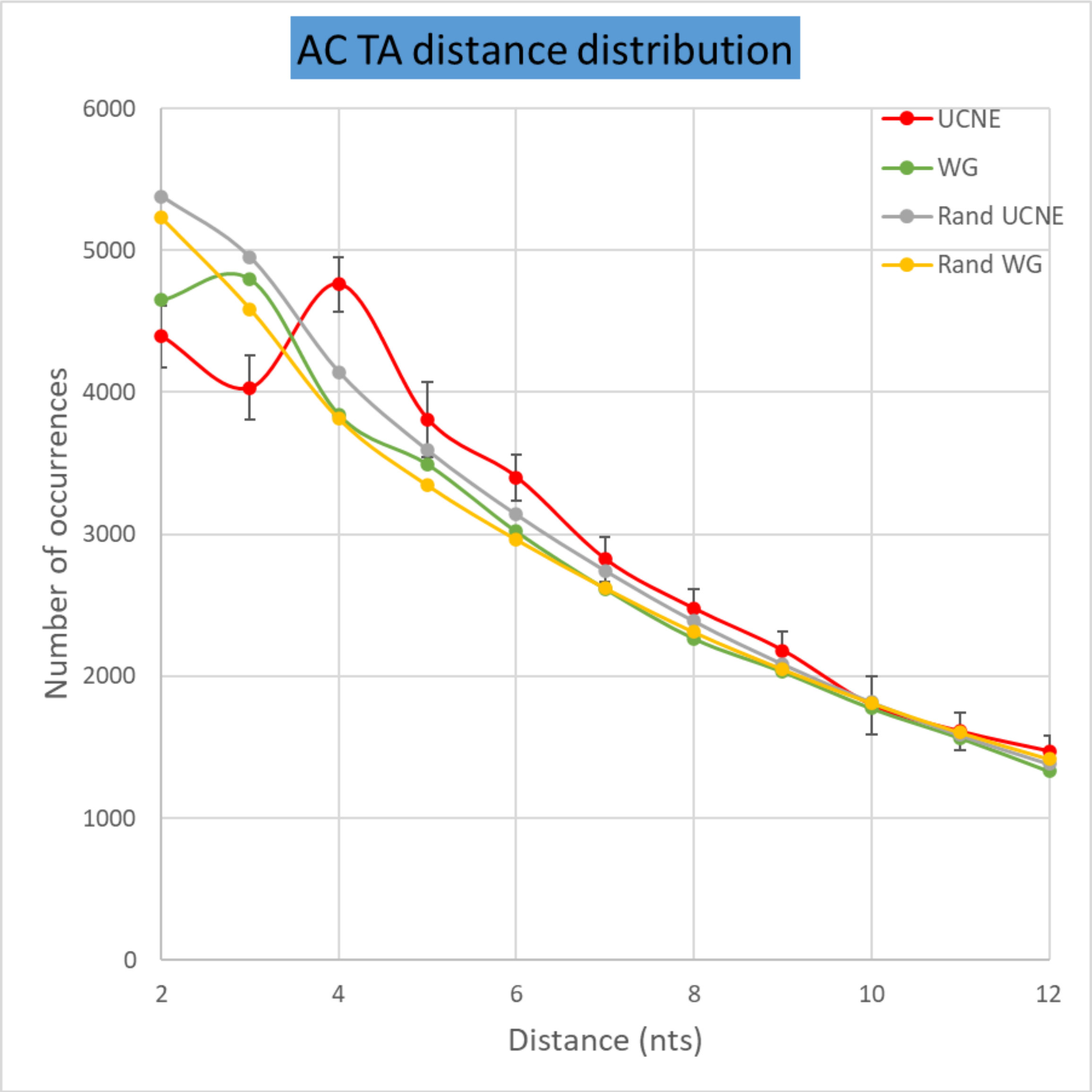
AC TA distance distribution
UCNE
WG
Rand UCNE
Rand WG
Number of occurrences
6000
5000
4000
3000
2000
1000
0
2
4
6
8
10
12
Distance (nts)

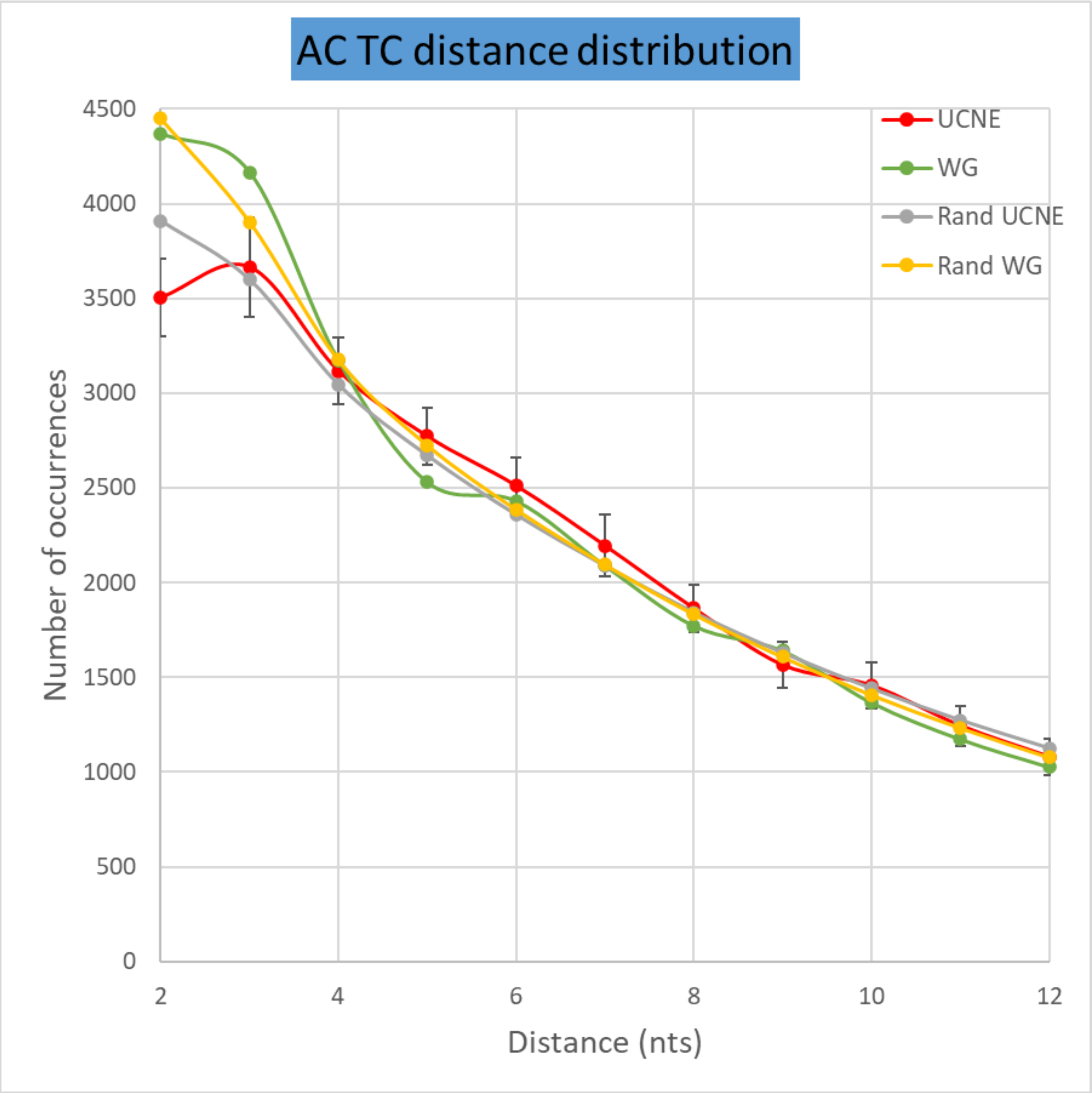
AC TC distance distribution
UCNE
WG
Rand UCNE
Rand WG
Number of occurrences
Distance (nts)
0
500
1000
1500
2000
2500
3000
3500
4000
4500
2
4
6
8
10
12

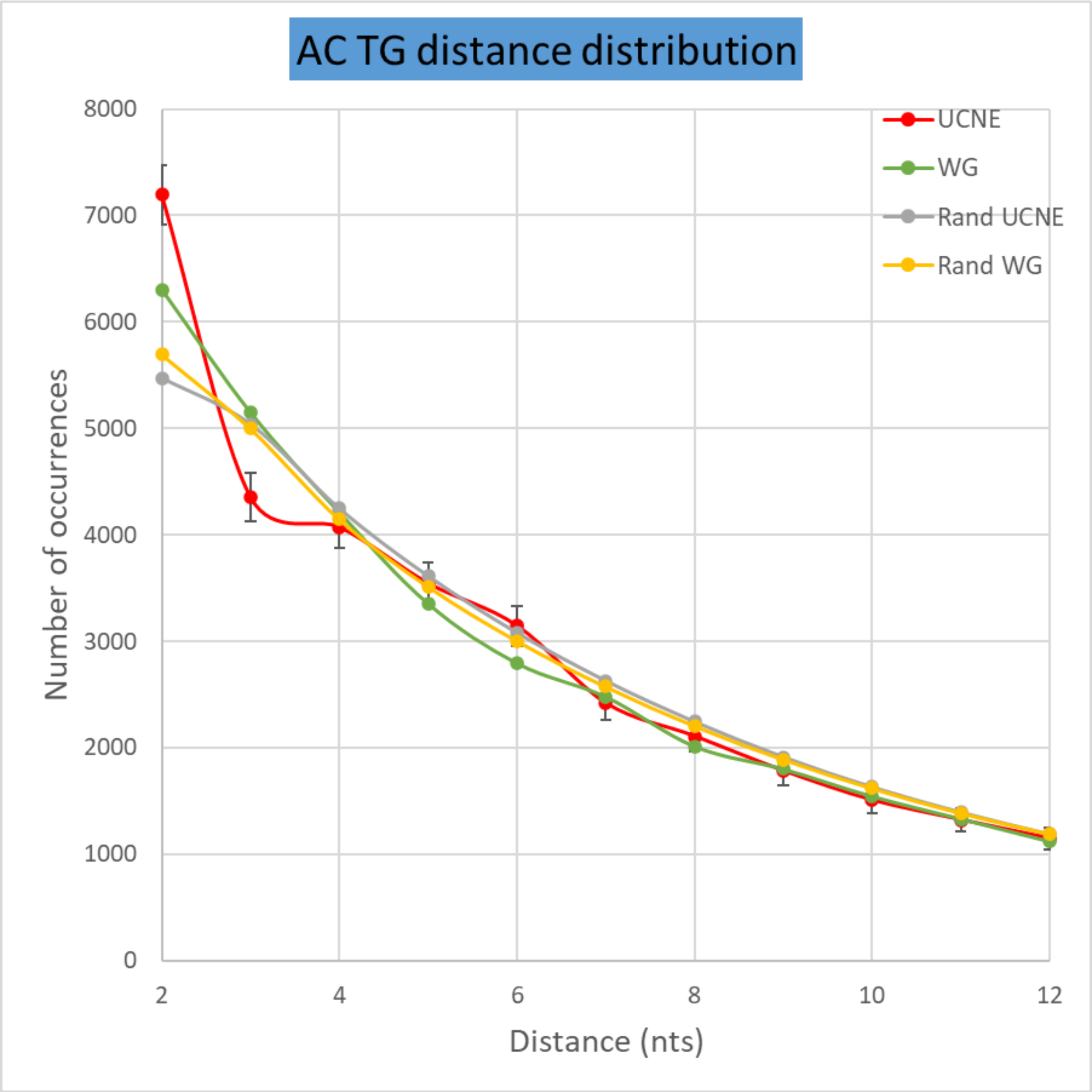
AC TG distance distribution
8000
7000
6000
5000
4000
3000
2000
1000
0
2
4
6
8
10
12
Number of occurrences
Distance (nts)
UCNE
WG
Rand UCNE
Rand WG

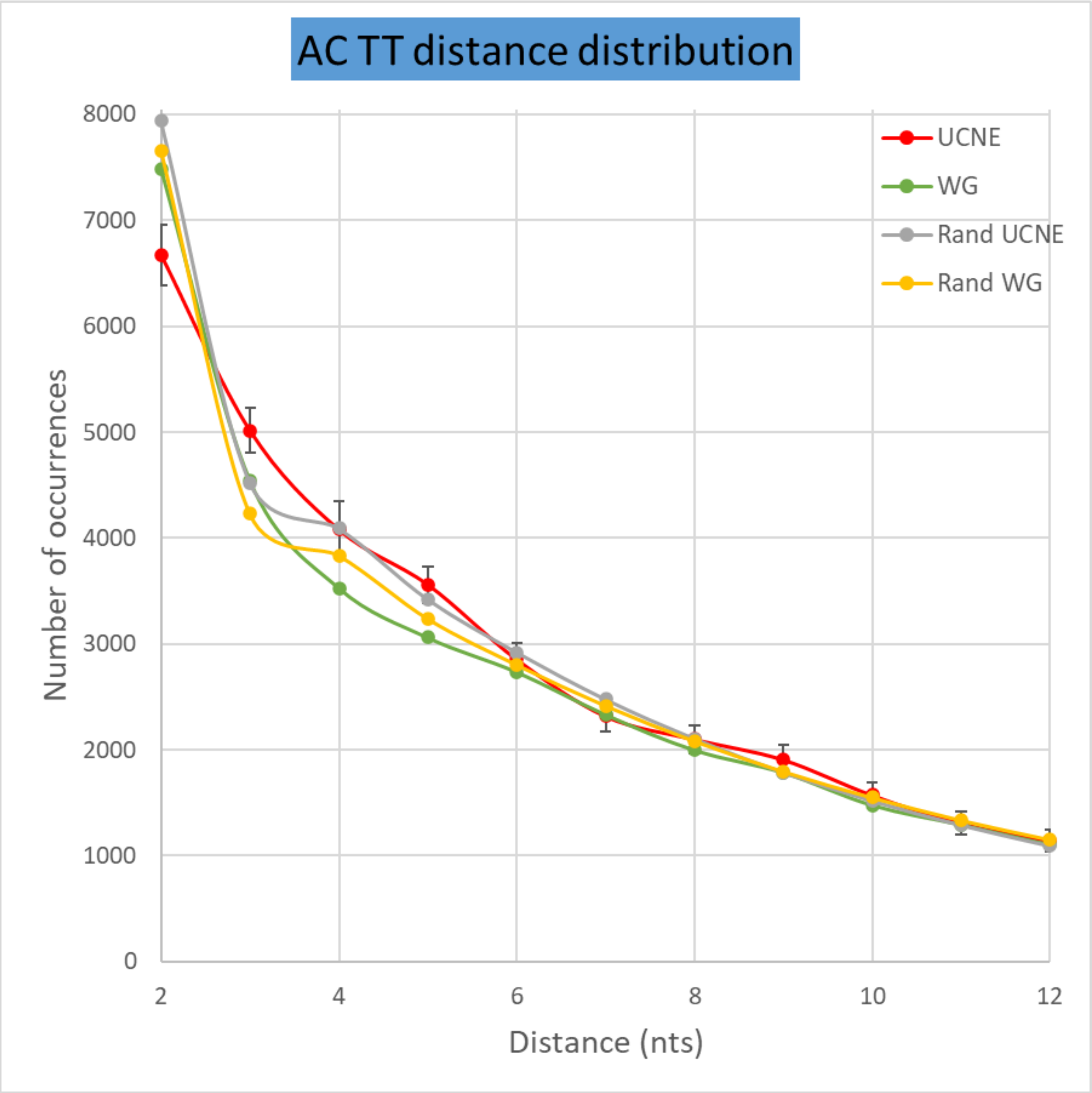
AC TT distance distribution
8000
7000
6000
5000
4000
3000
2000
1000
0
Number of occurrences
2
4
6
8
10
12
Distance (nts)
UCNE
WG
Rand UCNE
Rand WG

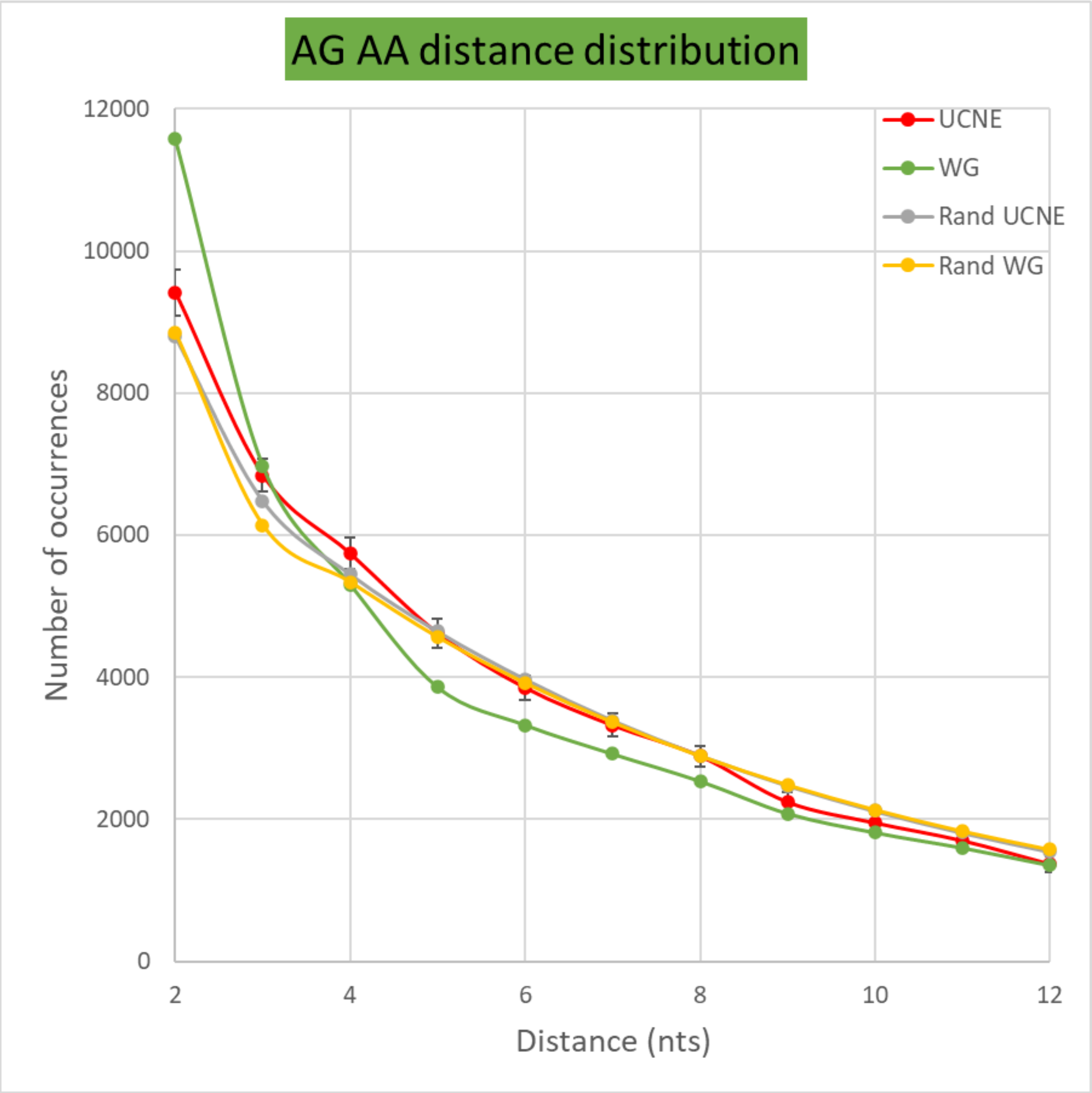

AG AA distance distribution
UCNE
WG
Rand UCNE
Rand WG
Number of occurrences
0
2000
4000
6000
8000
10000
12000
2
4
6
8
10
12
Distance (nts)

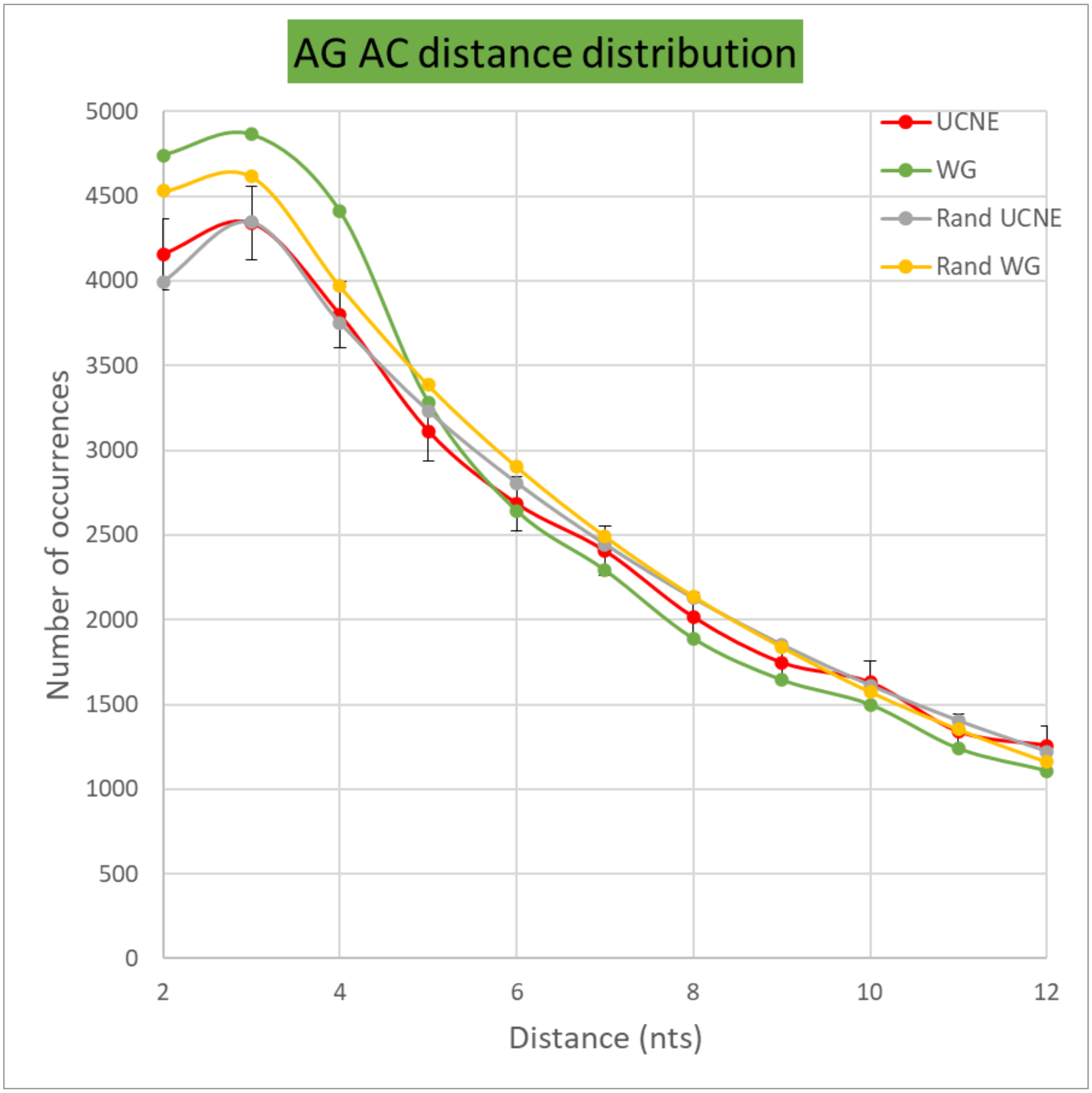

AG AC distance distribution
Number of occurrences
5000
4500
4000
3500
3000
2500
2000
1500
1000
500
0
2
4
6
8
10
12
Distance (nts)
UCNE
WG
Rand UCNE
Rand WG

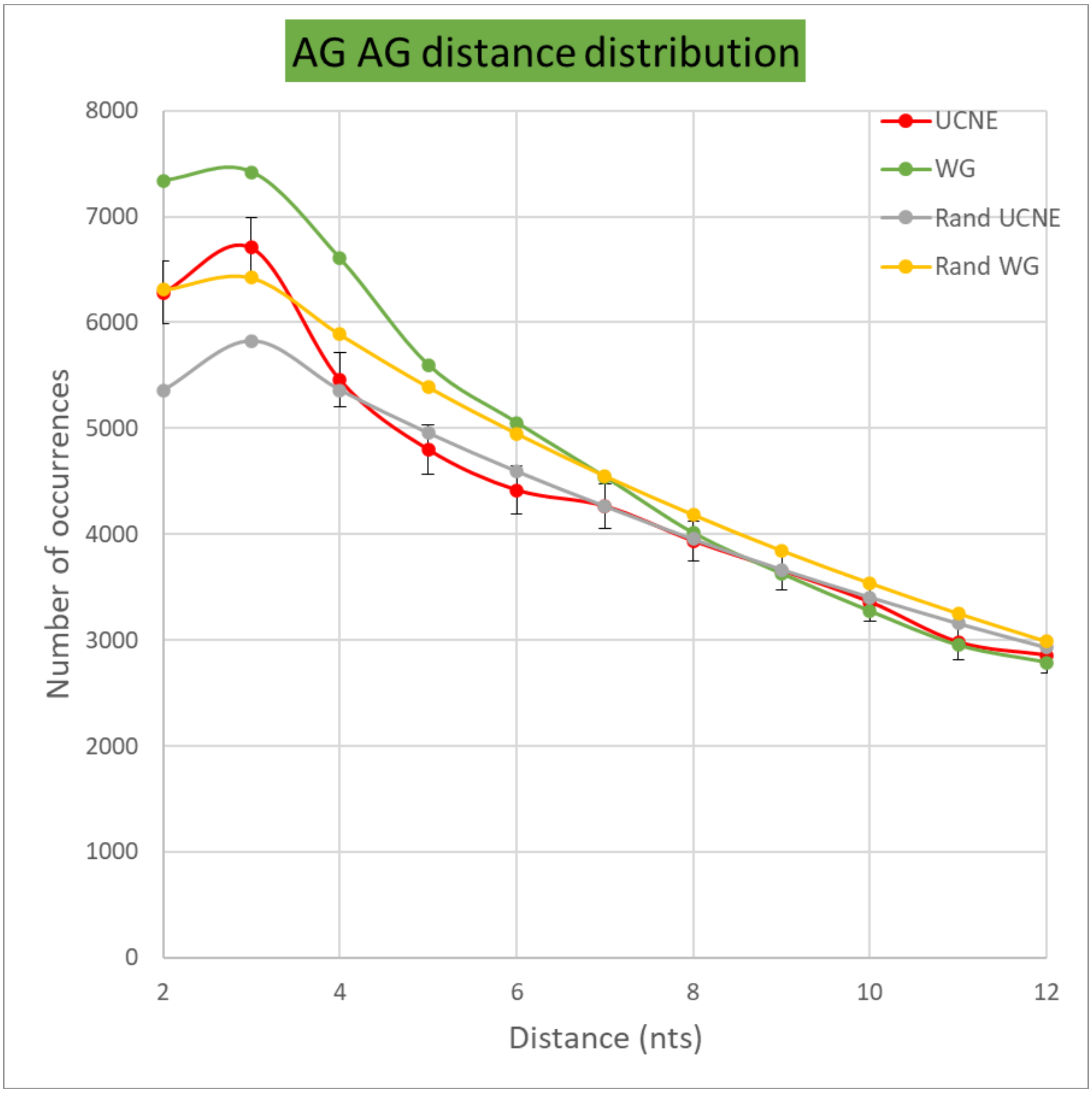

AG AG distance distribution
Number of occurrences
Distance (nts)
UCNE
WG
Rand UCNE
Rand WG
8000
7000
6000
5000
4000
3000
2000
1000
0
2
4
6
8
10
12

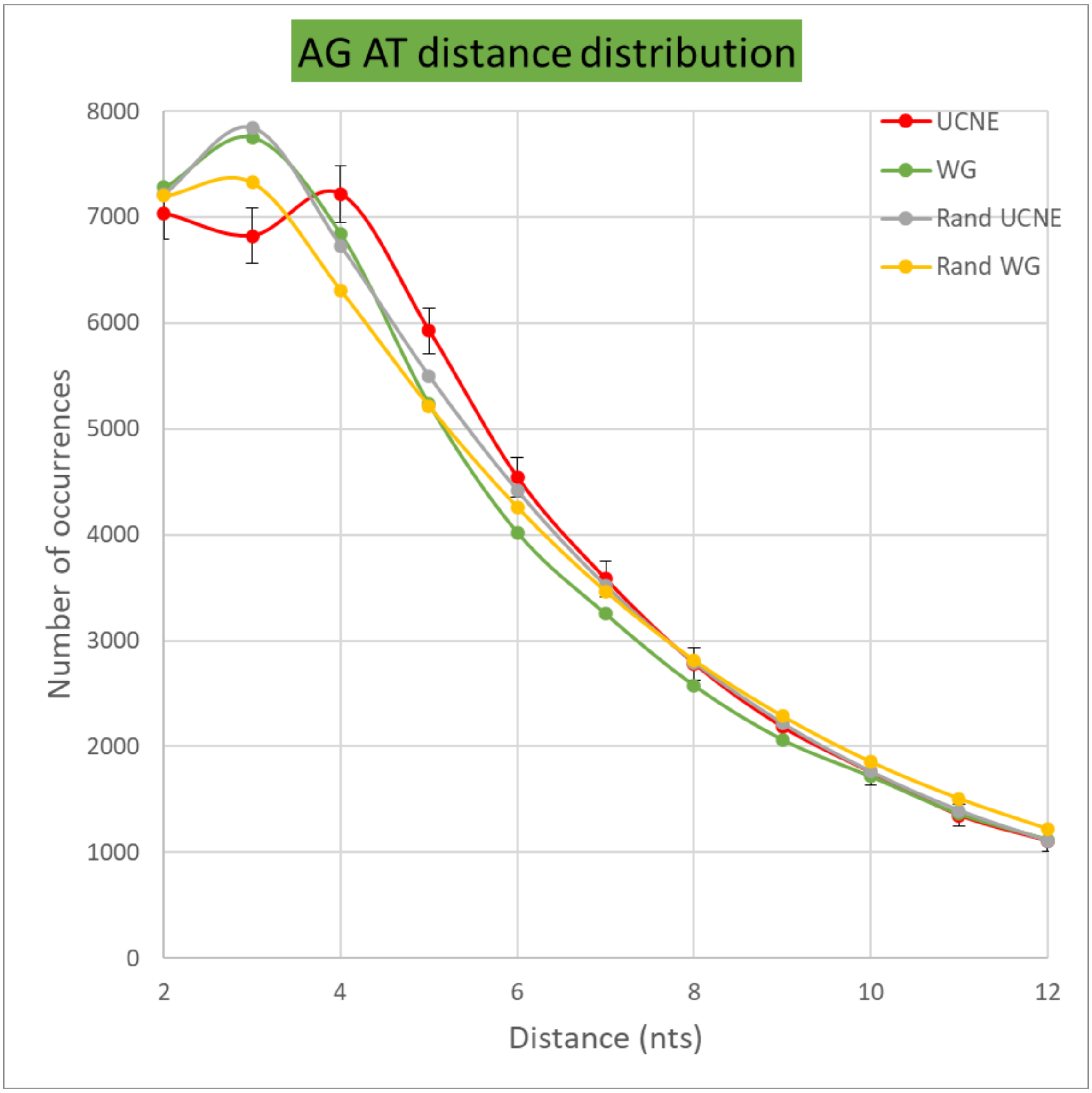

AG AT distance distribution
8000
7000
6000
5000
4000
3000
2000
1000
0
2
4
6
8
10
12
Number of occurrences
Distance (nts)
UCNE
WG
Rand UCNE
Rand WG

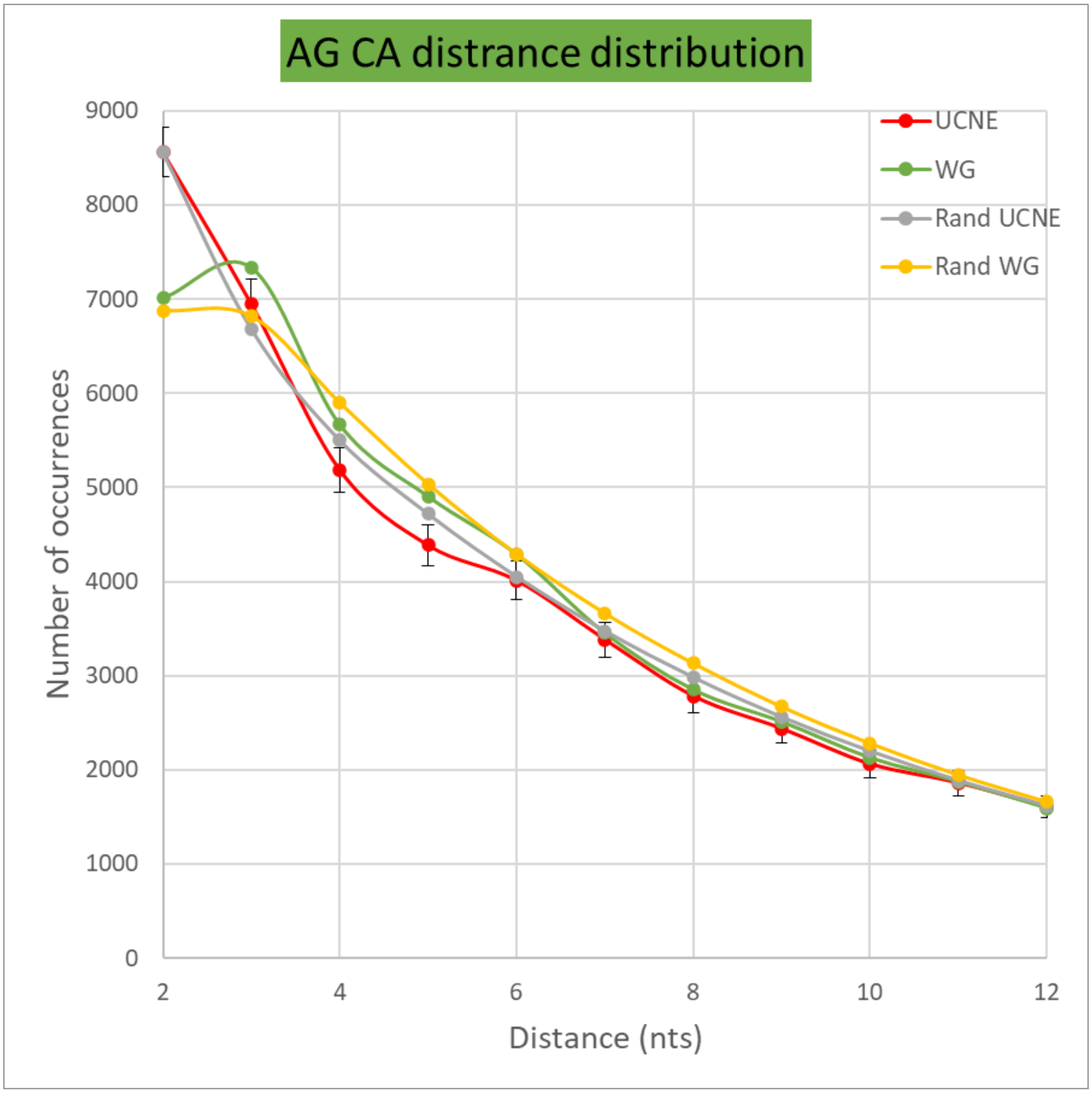
AG CA distrance distribution
UCNE
WG
Rand UCNE
Rand WG
Number of occurrences
9000
8000
7000
6000
5000
4000
3000
2000
1000
0
2
4
6
8
10
12
Distance (nts)

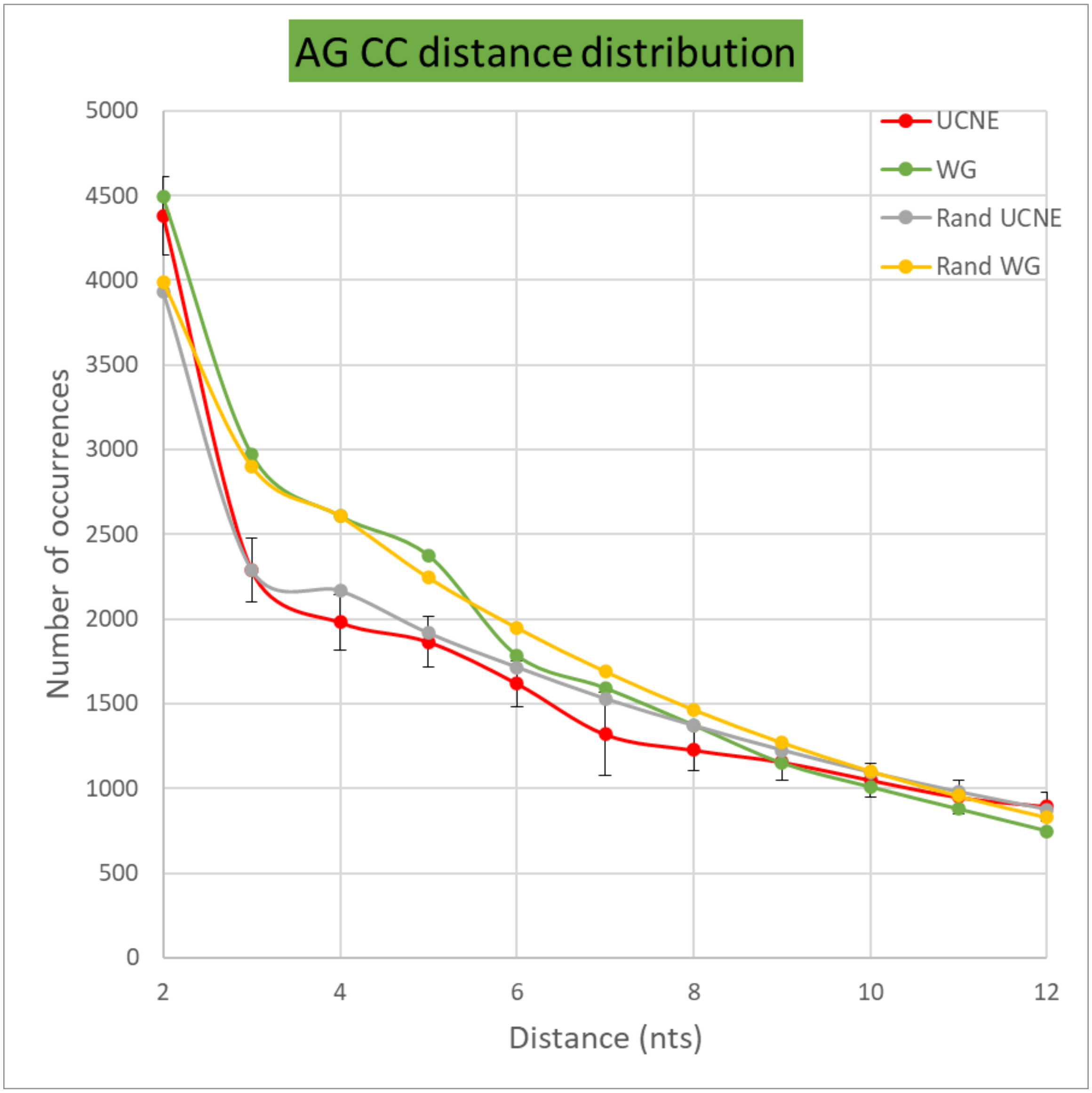
AG CC distance distribution
Number of occurrences
Distance (nts)
UCNE
WG
Rand UCNE
Rand WG
5000
4500
4000
3500
3000
2500
2000
1500
1000
500
0
2
4
6
8
10
12

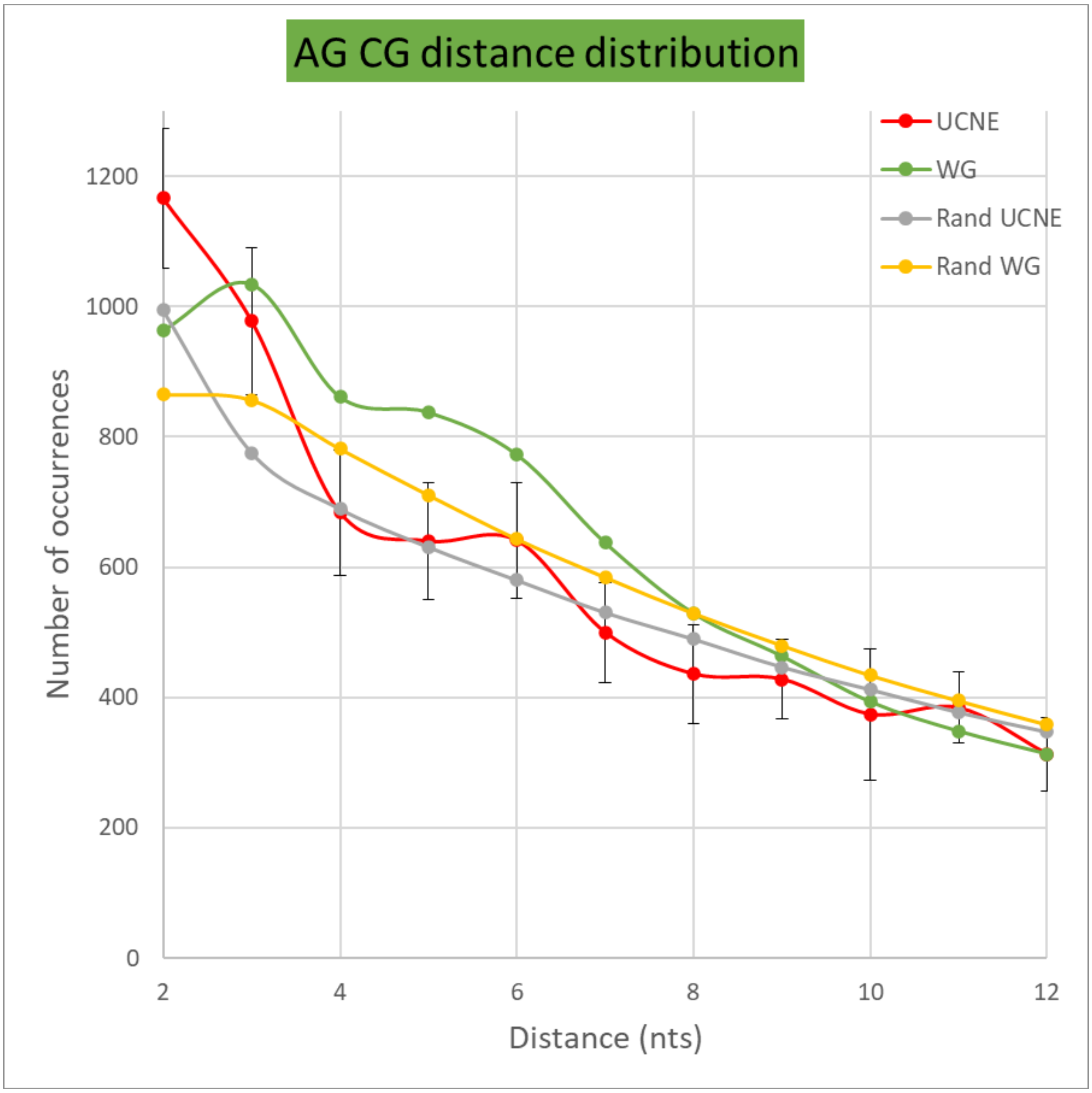
AG CG distance distribution
UCNE
WG
Rand UCNE
Rand WG
Number of occurrences
0
200
400
600
800
1000
1200
2
4
6
8
10
12
Distance (nts)

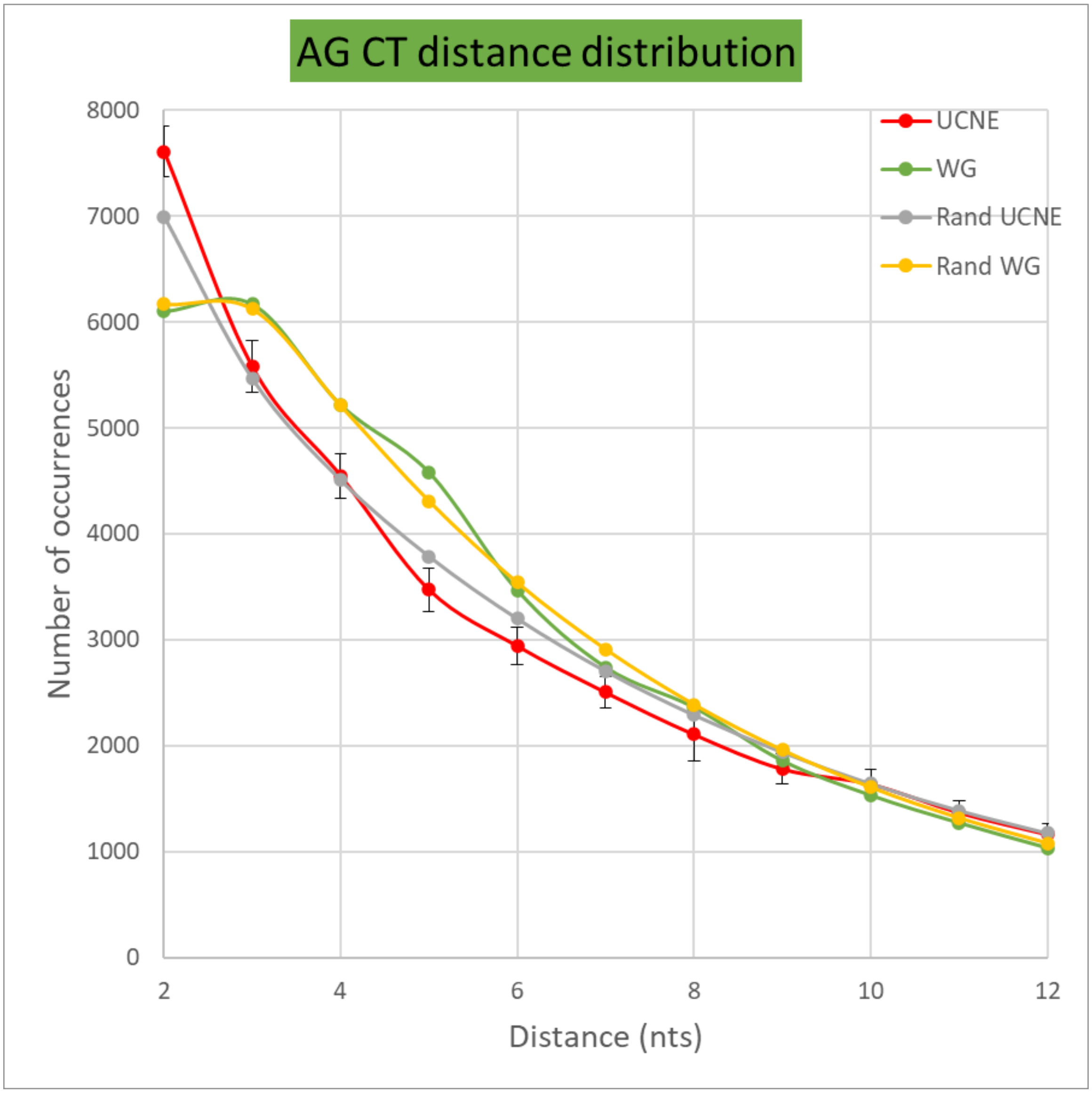
AG CT distance distribution
UCNE
WG
Rand UCNE
Rand WG
Number of occurrences
Distance (nts)
0
1000
2000
3000
4000
5000
6000
7000
8000
2
4
6
8
10
12

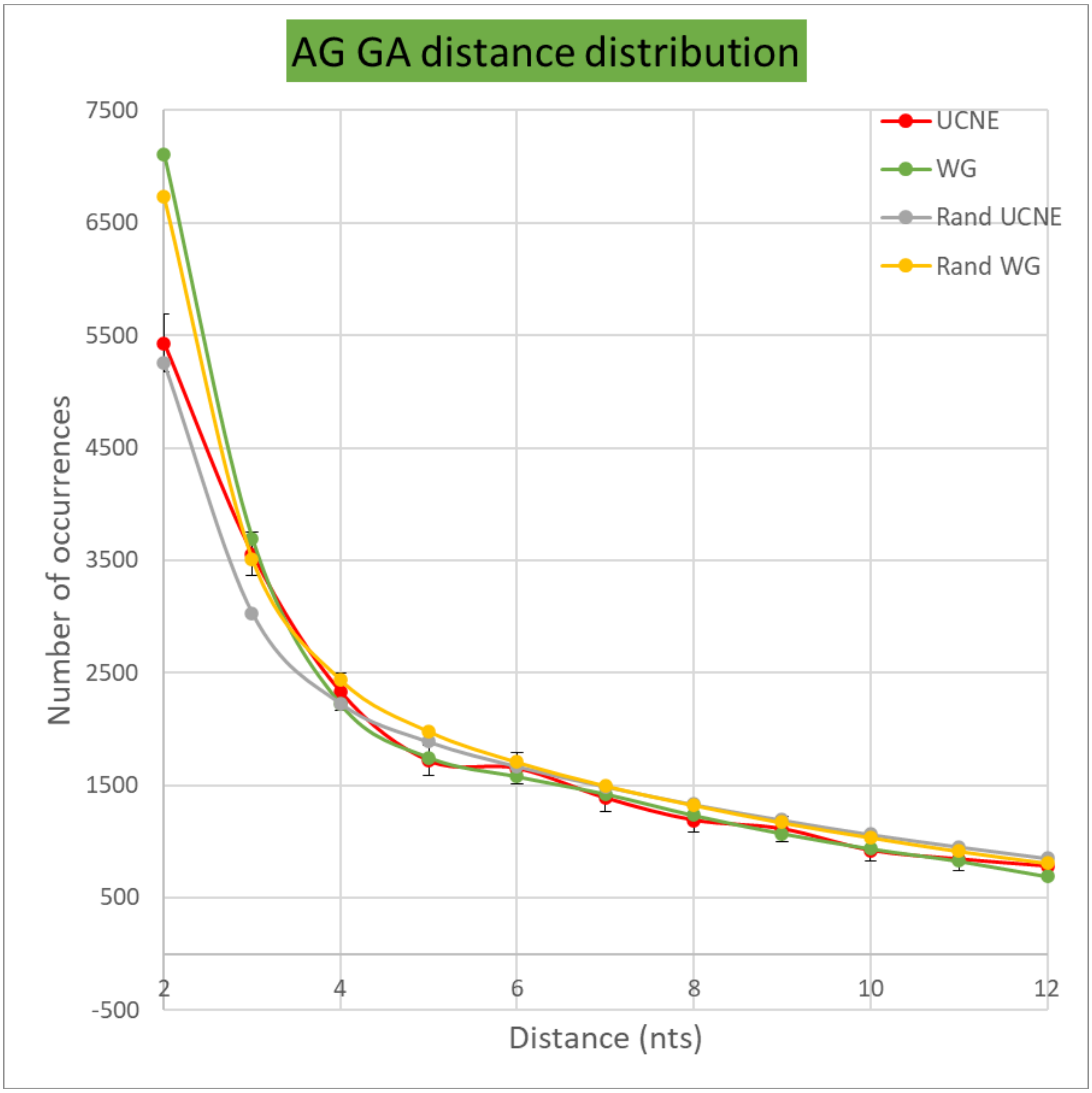

AG GA distance distribution
7500
6500
5500
4500
3500
2500
1500
500
-500
Number of occurrences
2
4
6
8
10
12
Distance (nts)
UCNE
WG
Rand UCNE
Rand WG

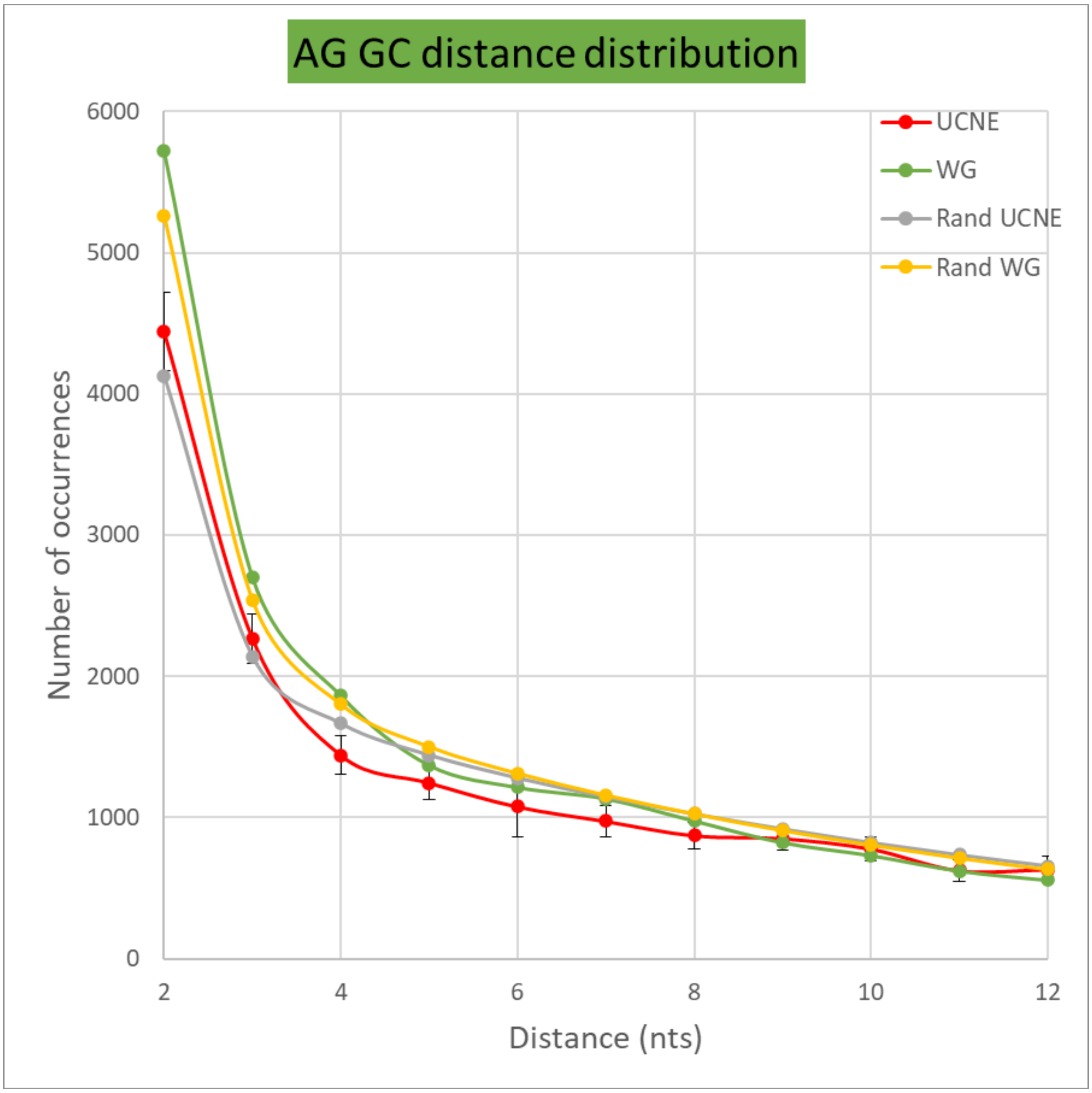
AG GC distance distribution
UCNE
WG
Rand UCNE
Rand WG
Number of occurrences
6000
5000
4000
3000
2000
1000
0
2
4
6
8
10
12
Distance (nts)

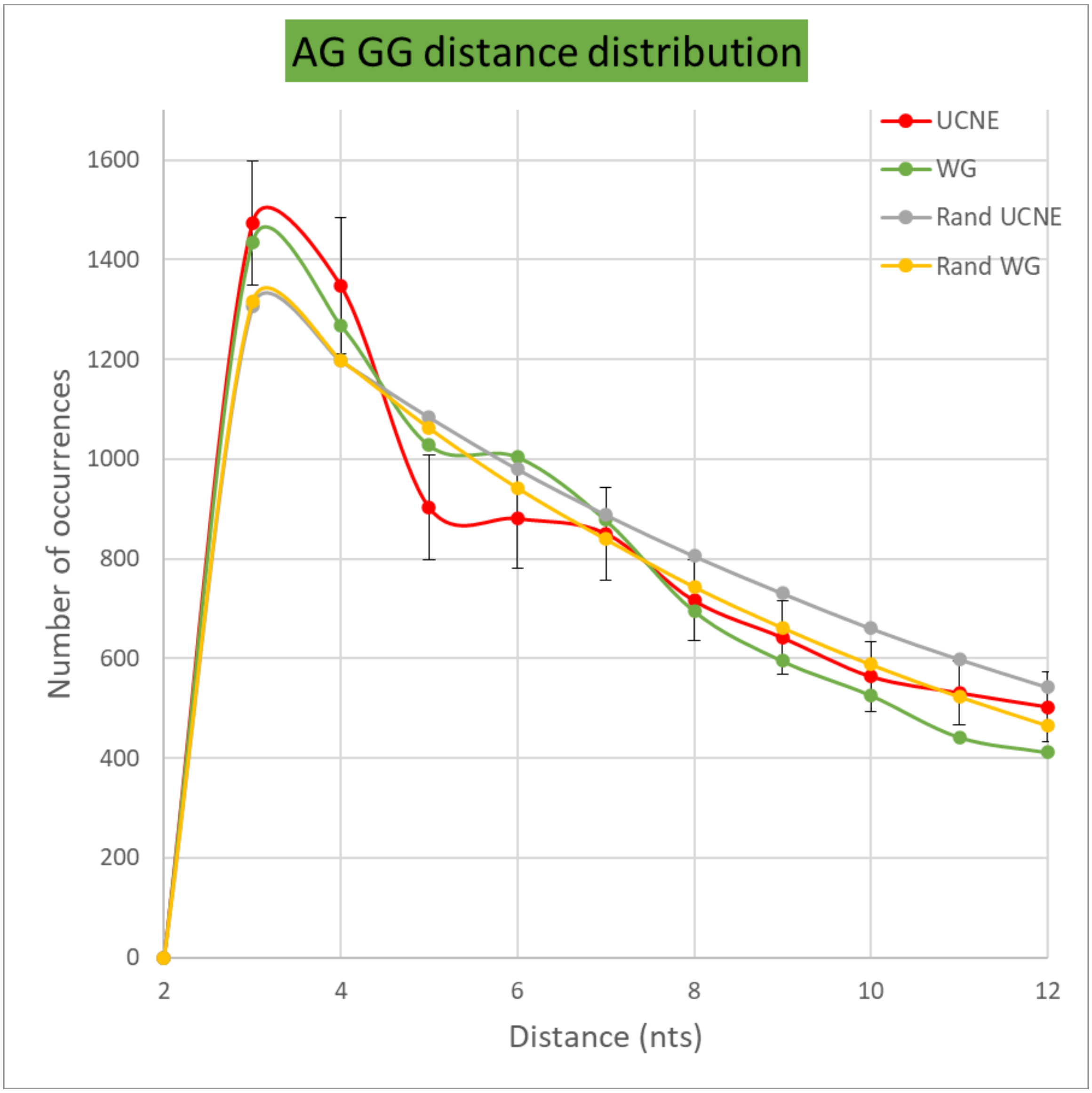
AG GG distance distribution
UCNE
WG
Rand UCNE
Rand WG
Number of occurrences
0
200
400
600
800
1000
1200
1400
1600
2
4
6
8
10
12
Distance (nts)

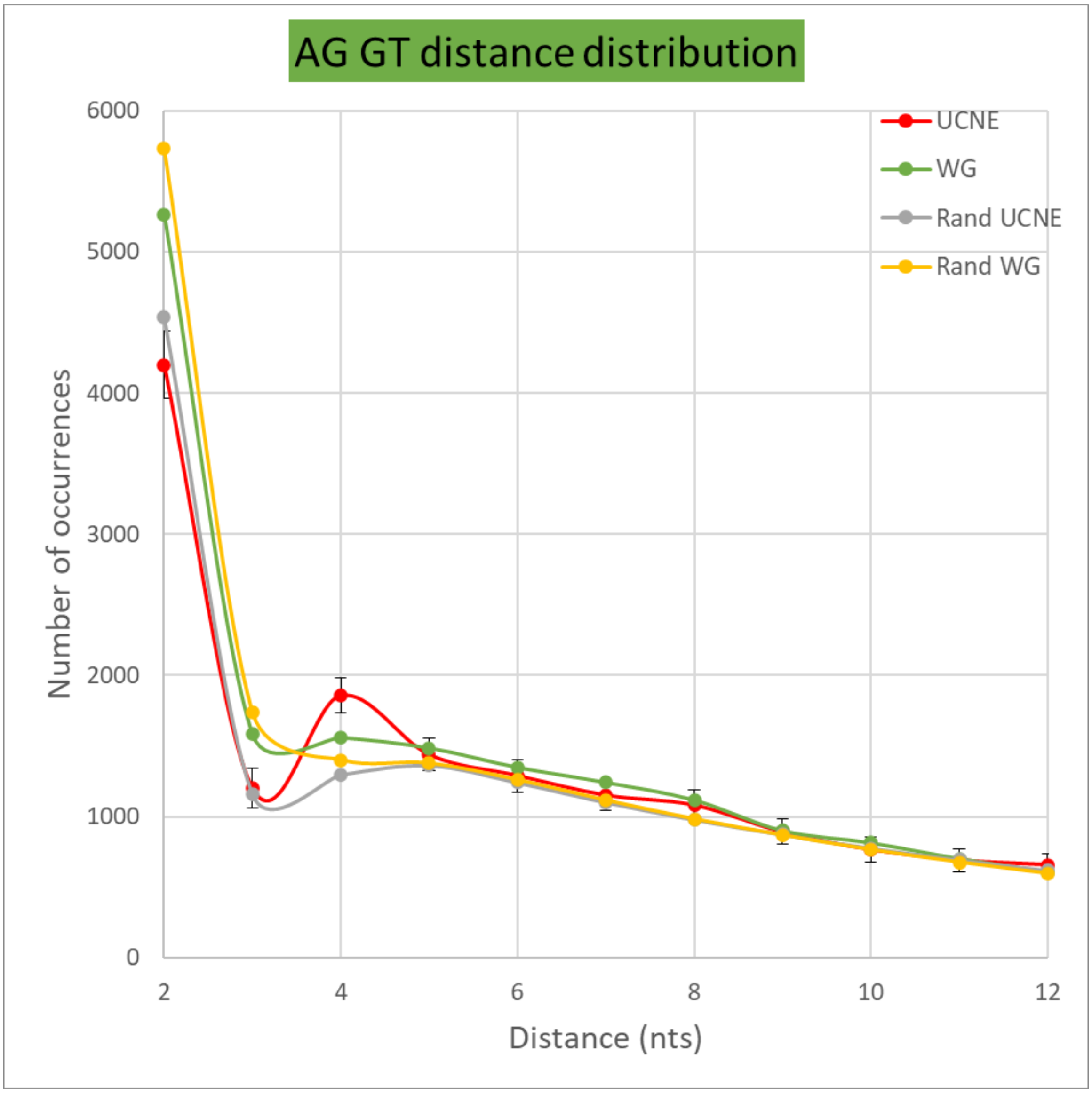

AG GT distance distribution
UCNE
WG
Rand UCNE
Rand WG
Number of occurrences
0
1000
2000
3000
4000
5000
6000
2
4
6
8
10
12
Distance (nts)

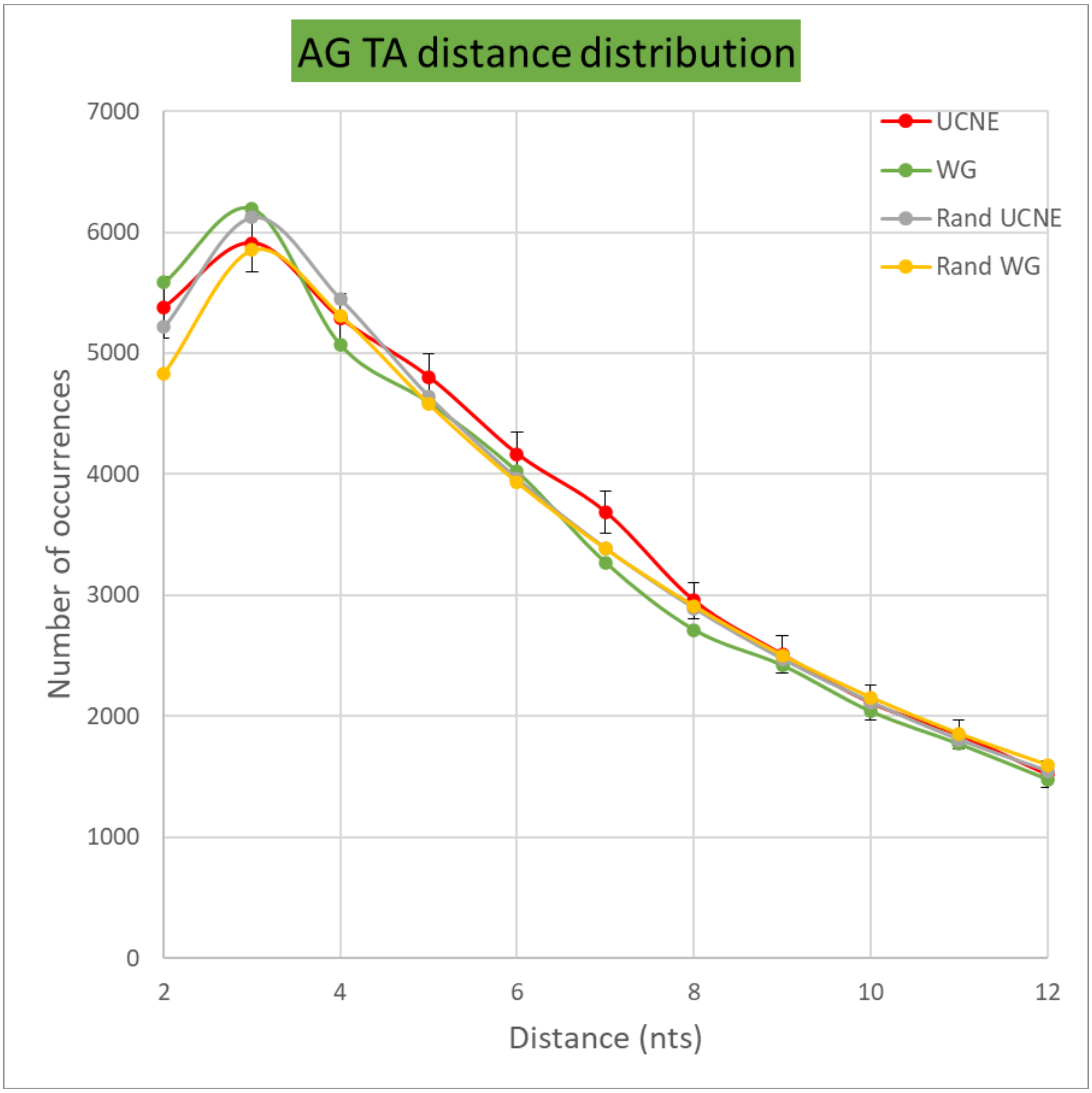
AG TA distance distribution
UCNE
WG
Rand UCNE
Rand WG
Number of occurrences
0
1000
2000
3000
4000
5000
6000
7000
2
4
6
8
10
12
Distance (nts)

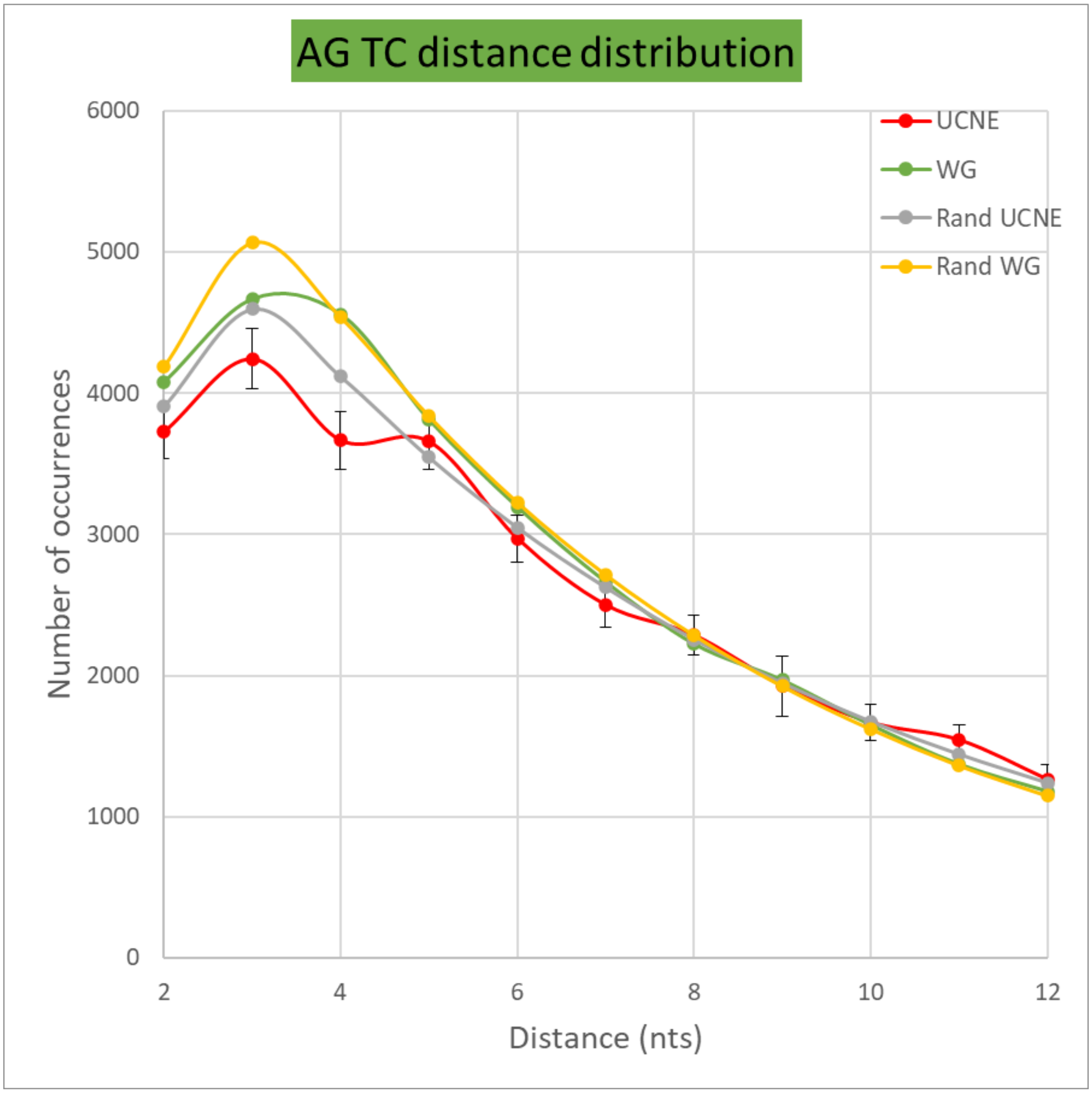

AG TC distance distribution
6000
5000
4000
3000
2000
1000
0
Number of occurrences
2
4
6
8
10
12
Distance (nts)
UCNE
WG
Rand UCNE
Rand WG

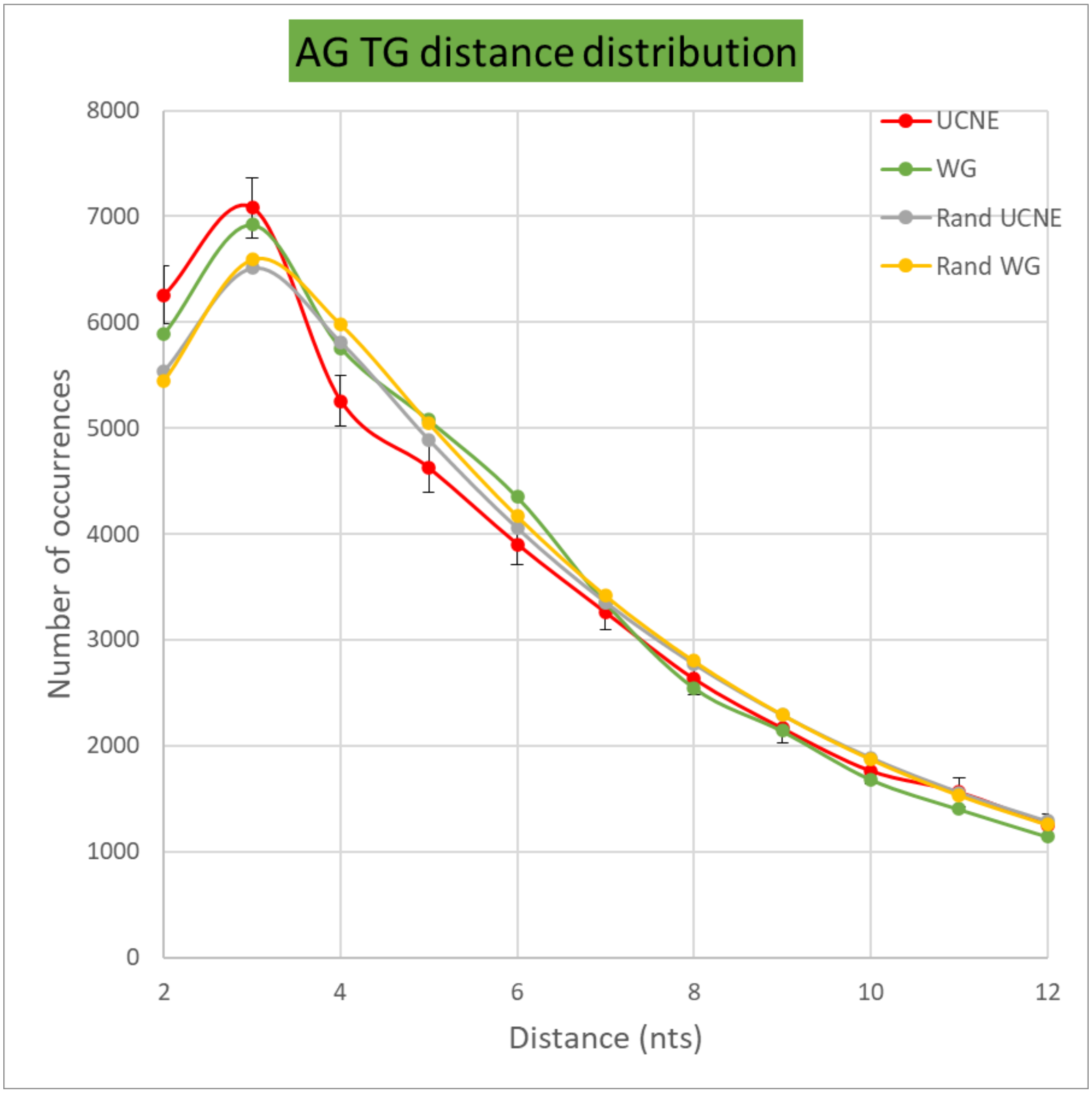

AG TG distance distribution
8000
7000
6000
5000
4000
3000
2000
1000
0
Number of occurrences
2
4
6
8
10
12
Distance (nts)
UCNE
WG
Rand UCNE
Rand WG

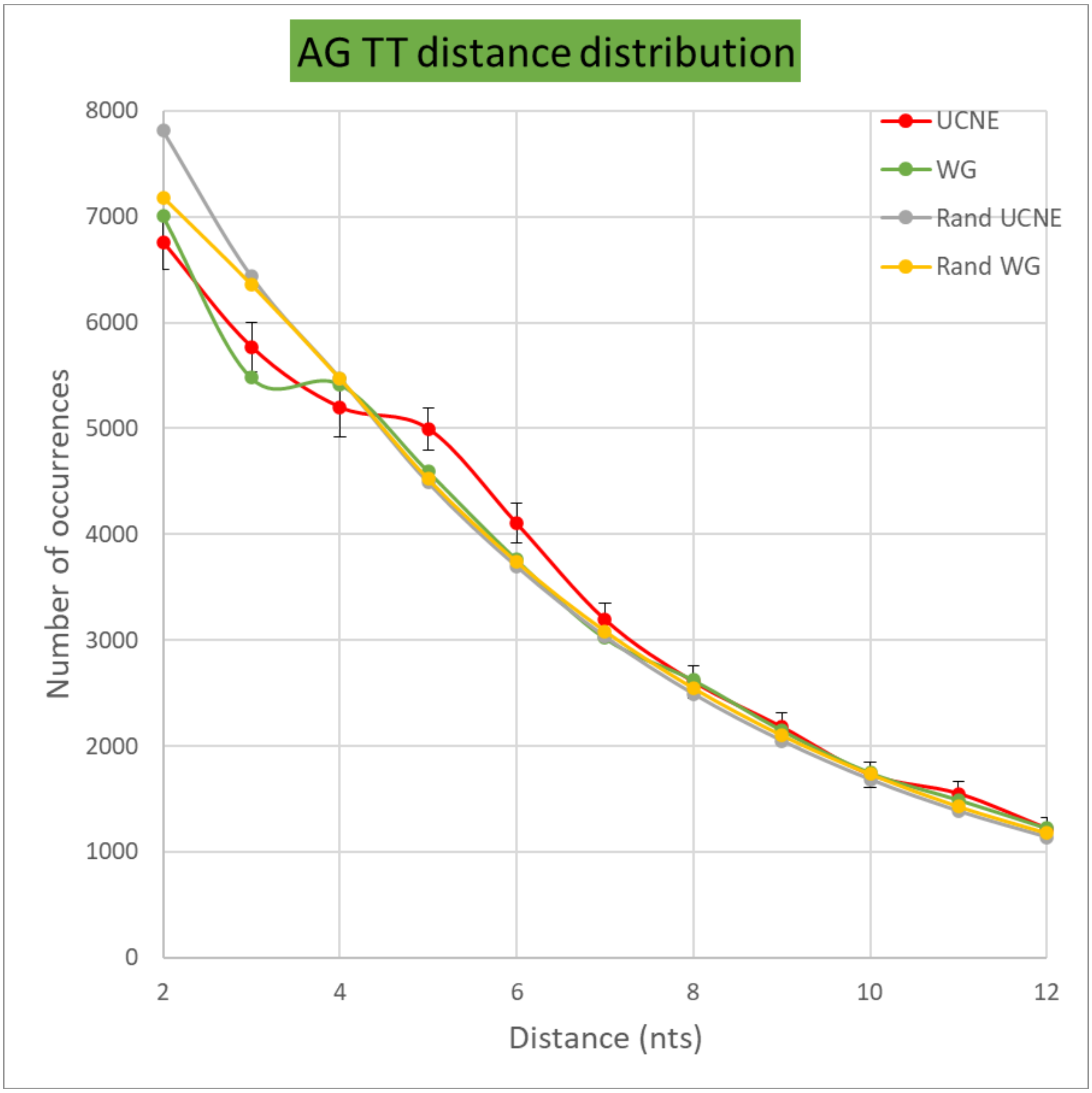

AG TT distance distribution
8000
7000
6000
5000
4000
3000
2000
1000
0
Number of occurrences
2
4
6
8
10
12
Distance (nts)
UCNE
WG
Rand UCNE
Rand WG

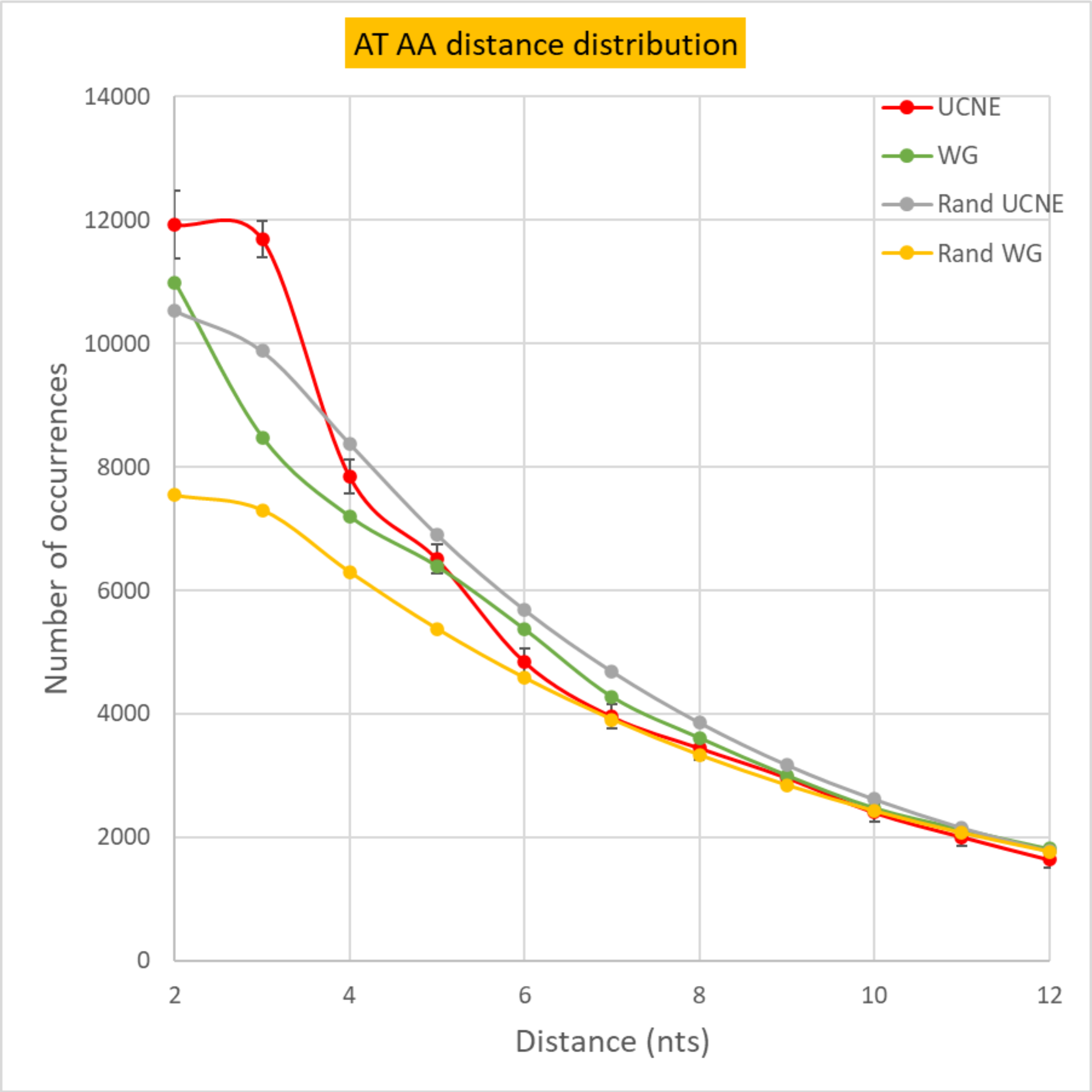
AT AA distance distribution
UCNE
WG
Rand UCNE
Rand WG
Number of occurrences
Distance (nts)
0
2000
4000
6000
8000
10000
12000
14000
2
4
6
8
10
12

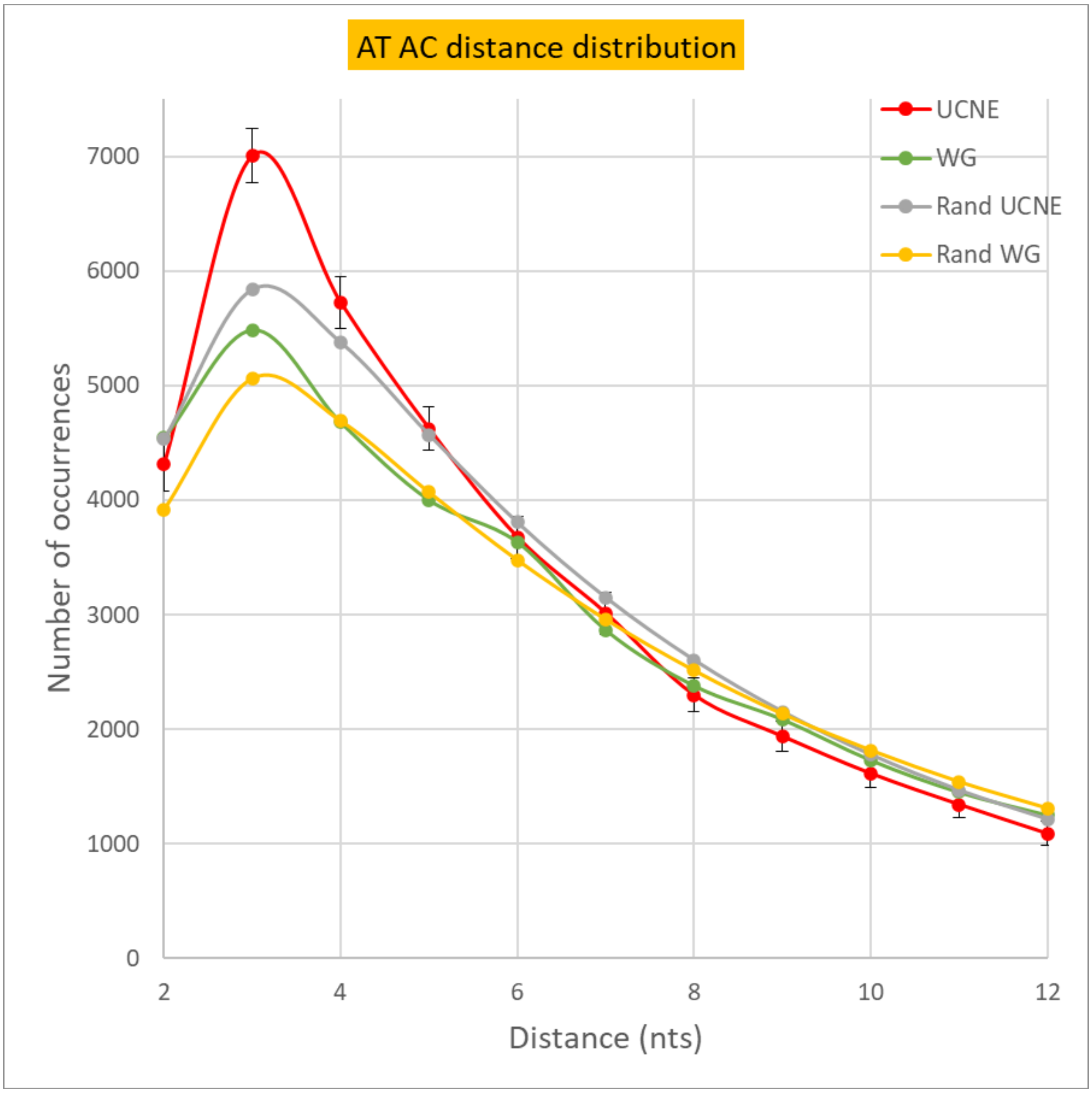
AT AC distance distribution
UCNE
WG
Rand UCNE
Rand WG
Number of occurrences
0
1000
2000
3000
4000
5000
6000
7000
2
4
6
8
10
12
Distance (nts)

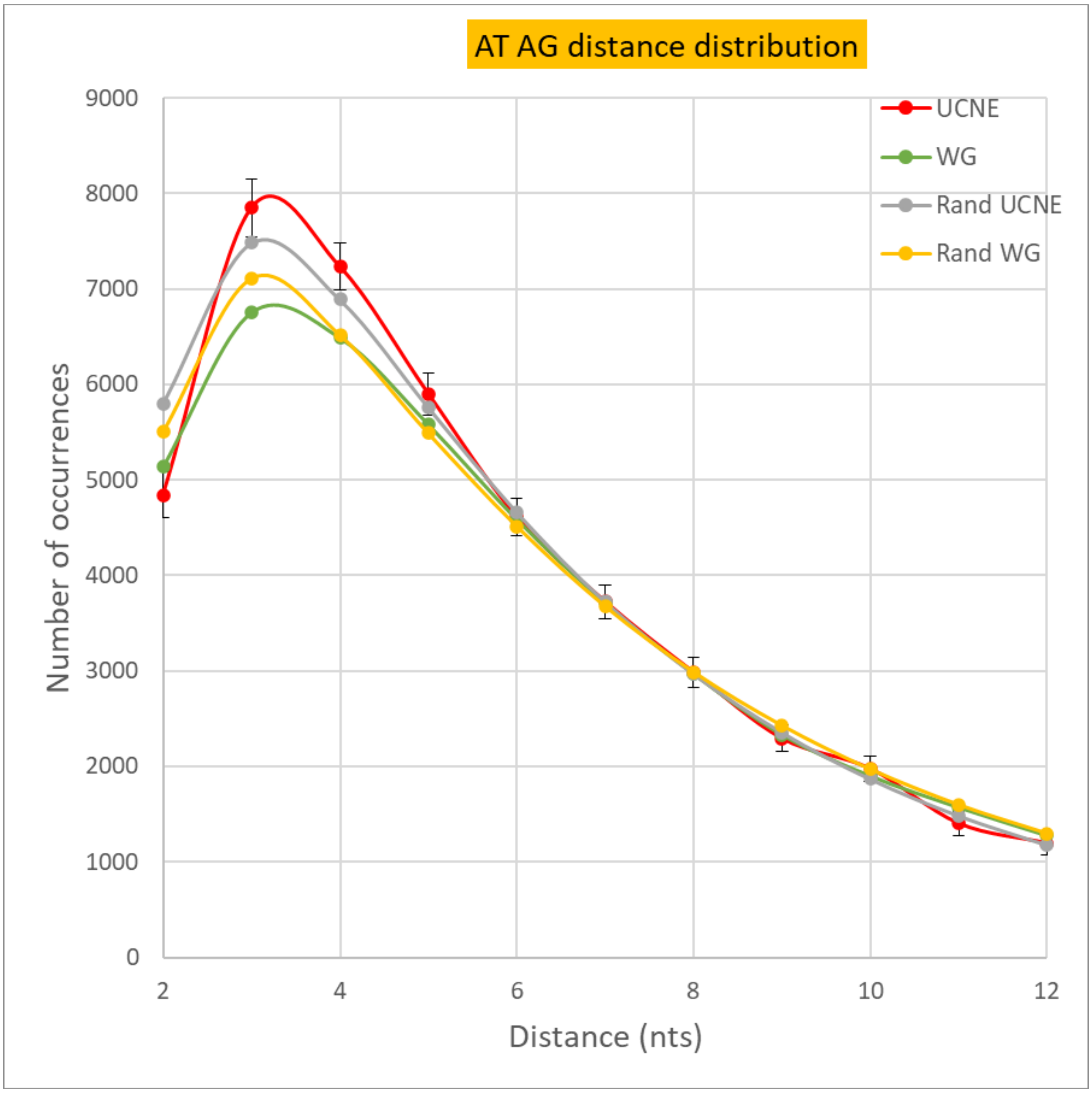
AT AG distance distribution
UCNE
WG
Rand UCNE
Rand WG
Number of occurrences
9000
8000
7000
6000
5000
4000
3000
2000
1000
0
2
4
6
8
10
12
Distance (nts)

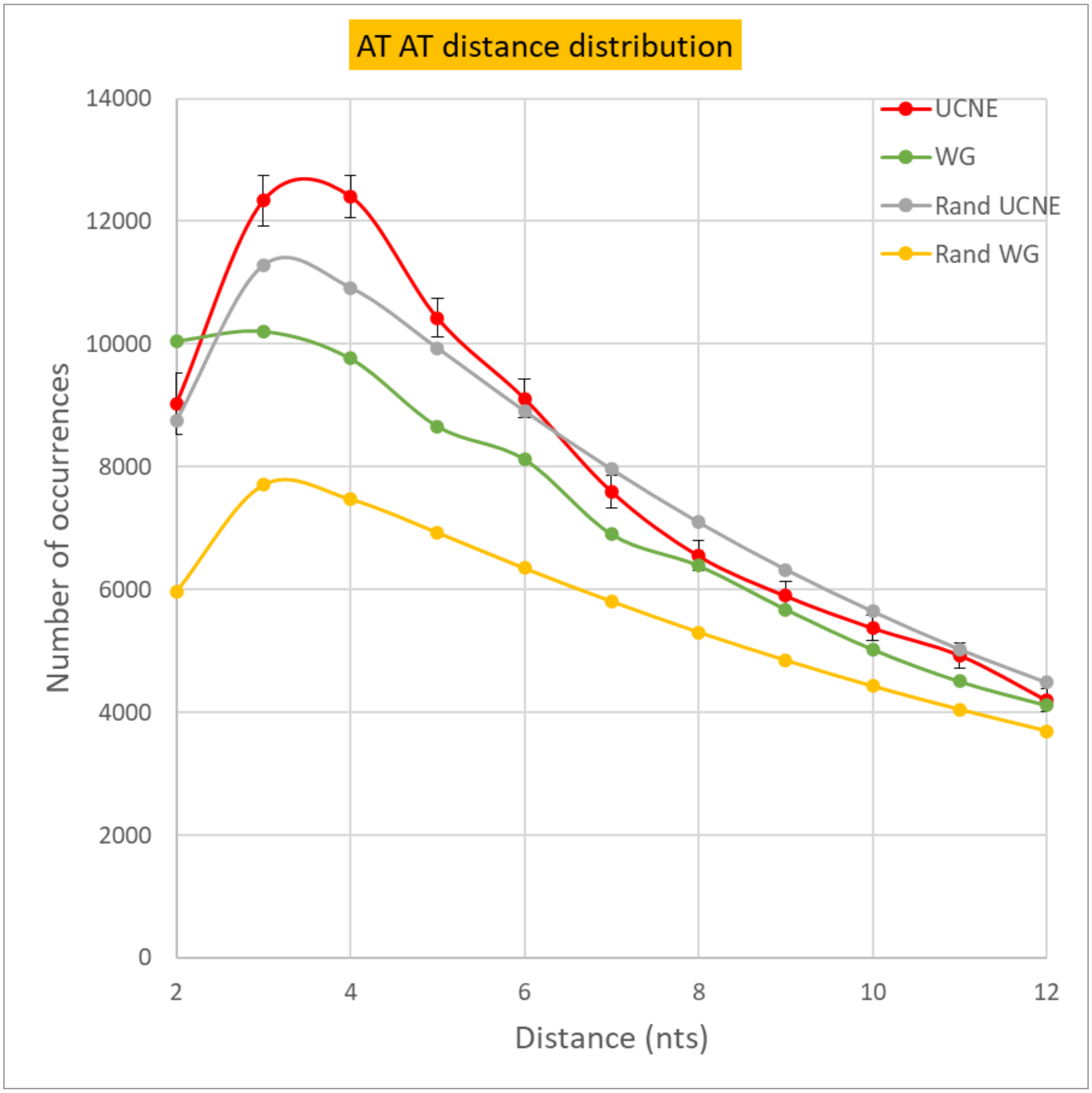
AT AT distance distribution
UCNE
WG
Rand UCNE
Rand WG
Number of occurrences
14000
12000
10000
8000
6000
4000
2000
0
2
4
6
8
10
12
Distance (nts)

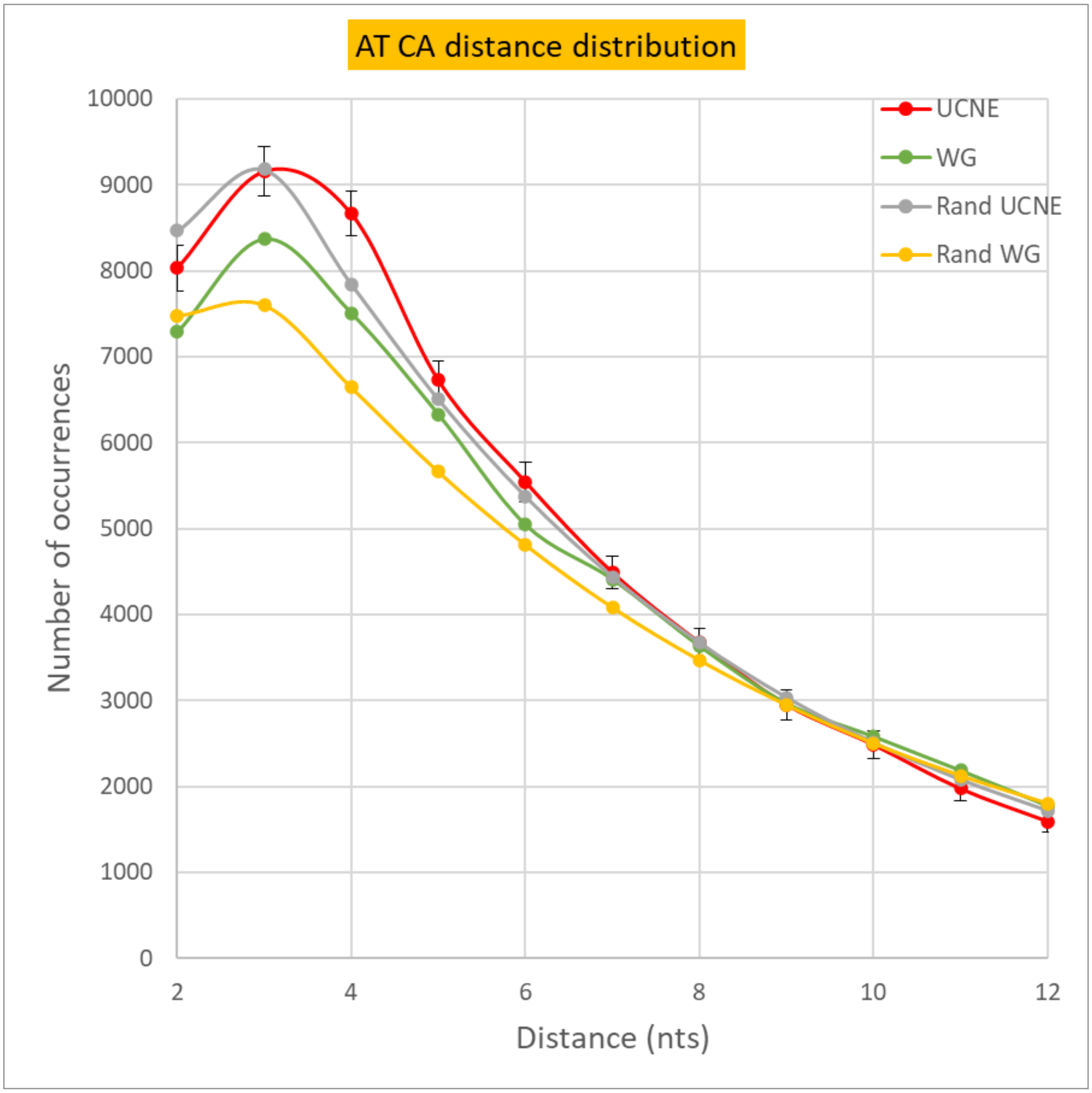
AT CA distance distribution
Number of occurrences
Distance (nts)
UCNE
WG
Rand UCNE
Rand WG
0
1000
2000
3000
4000
5000
6000
7000
8000
9000
10000
2
4
6
8
10
12

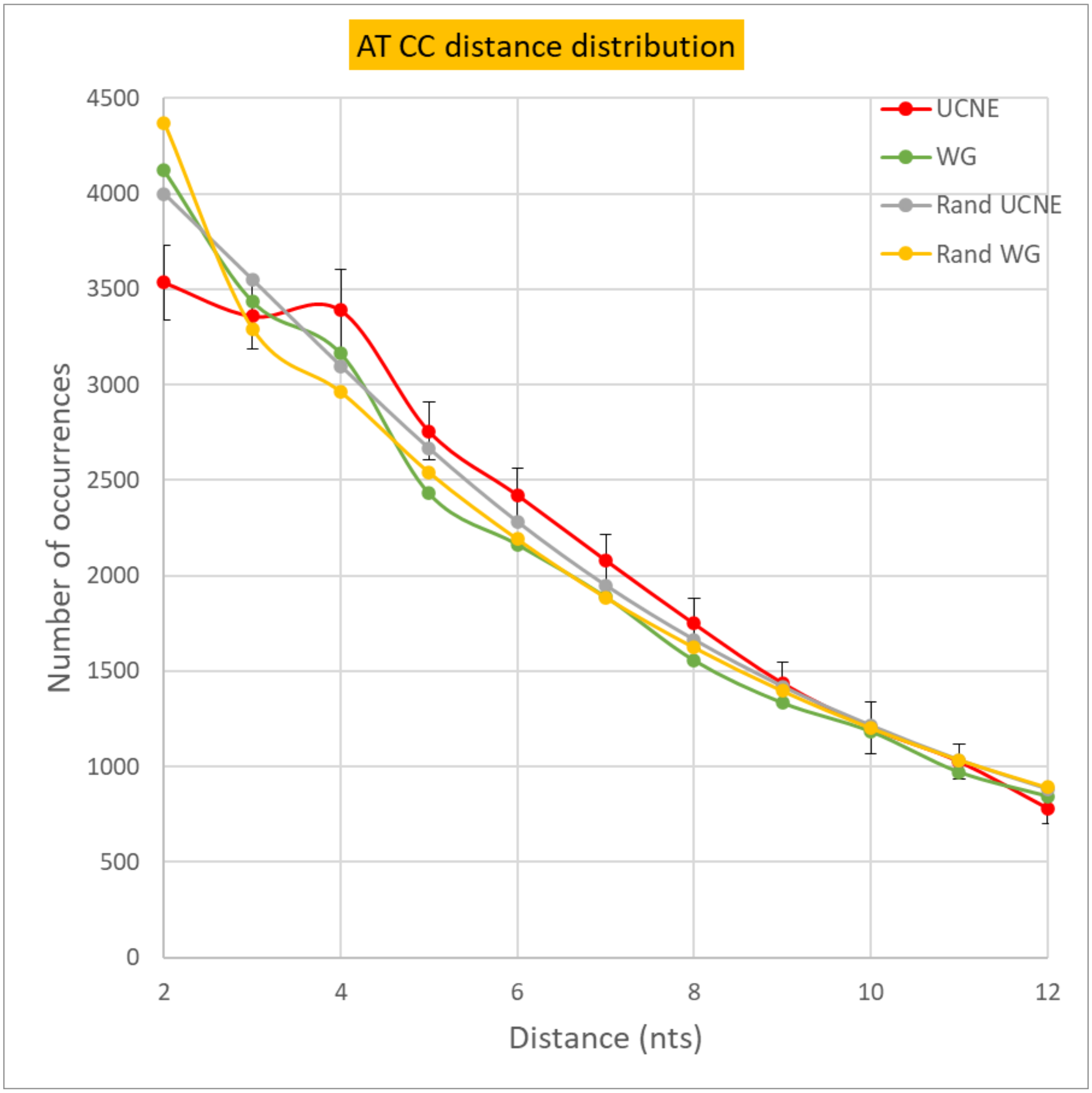
AT CC distance distribution
Number of occurrences
4500
4000
3500
3000
2500
2000
1500
1000
500
0
2
4
6
8
10
12
Distance (nts)
UCNE
WG
Rand UCNE
Rand WG

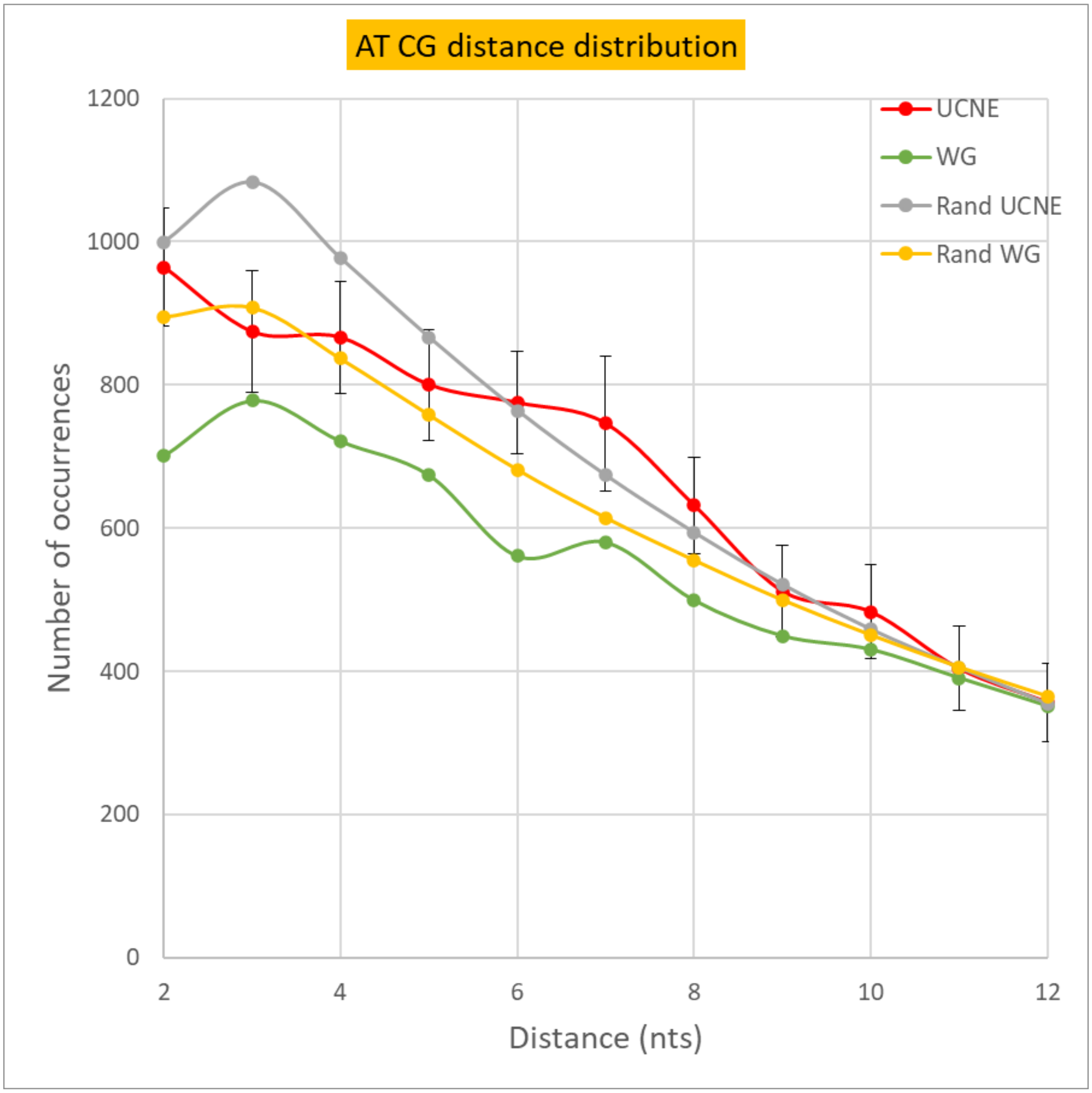
AT CG distance distribution
1200
1000
800
600
400
200
0
Number of occurrences
2
4
6
8
10
12
Distance (nts)
UCNE
WG
Rand UCNE
Rand WG

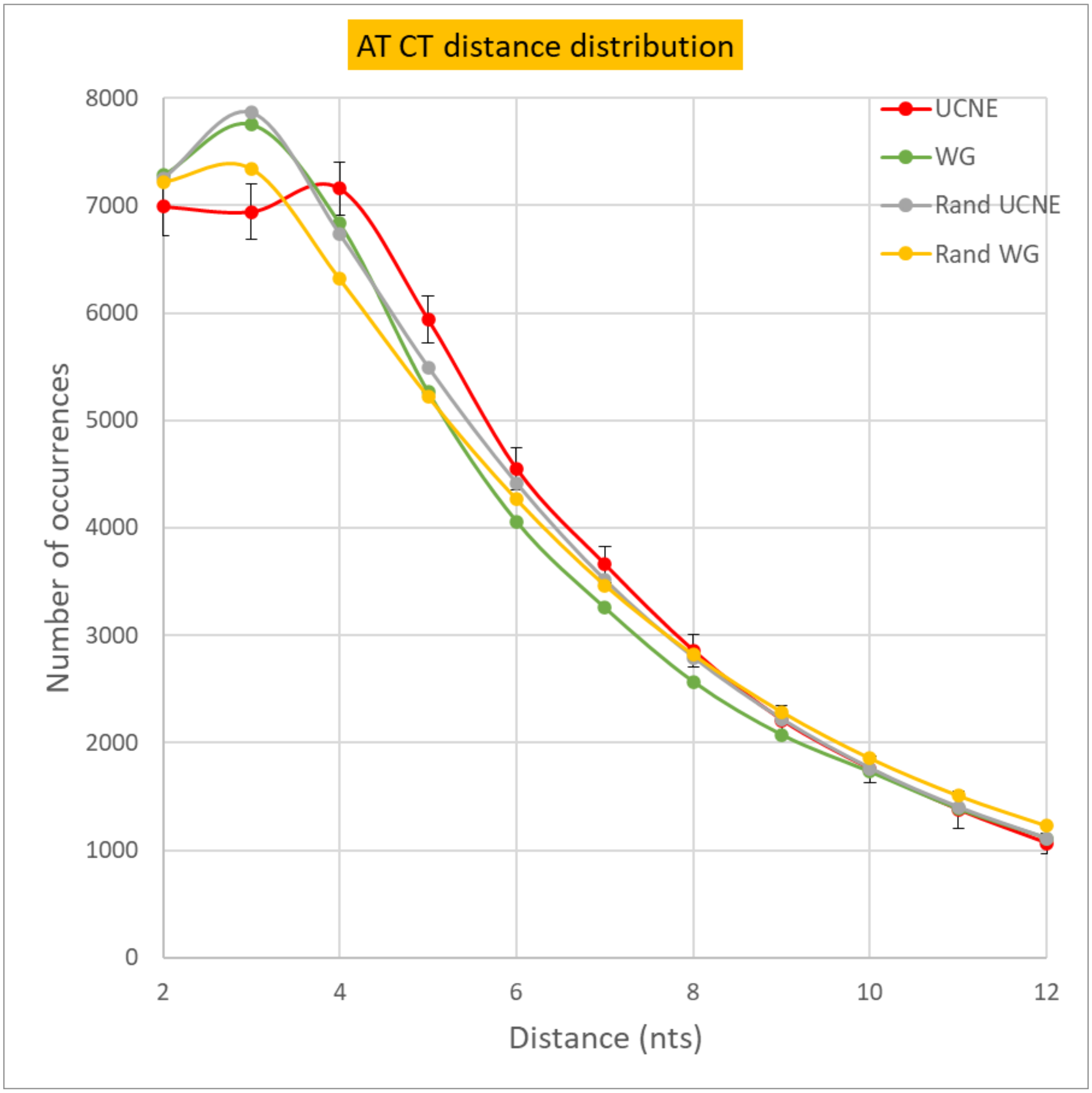
AT CT distance distribution
8000
7000
6000
5000
4000
3000
2000
1000
0
Number of occurrences
2
4
6
8
10
12
Distance (nts)
UCNE
WG
Rand UCNE
Rand WG

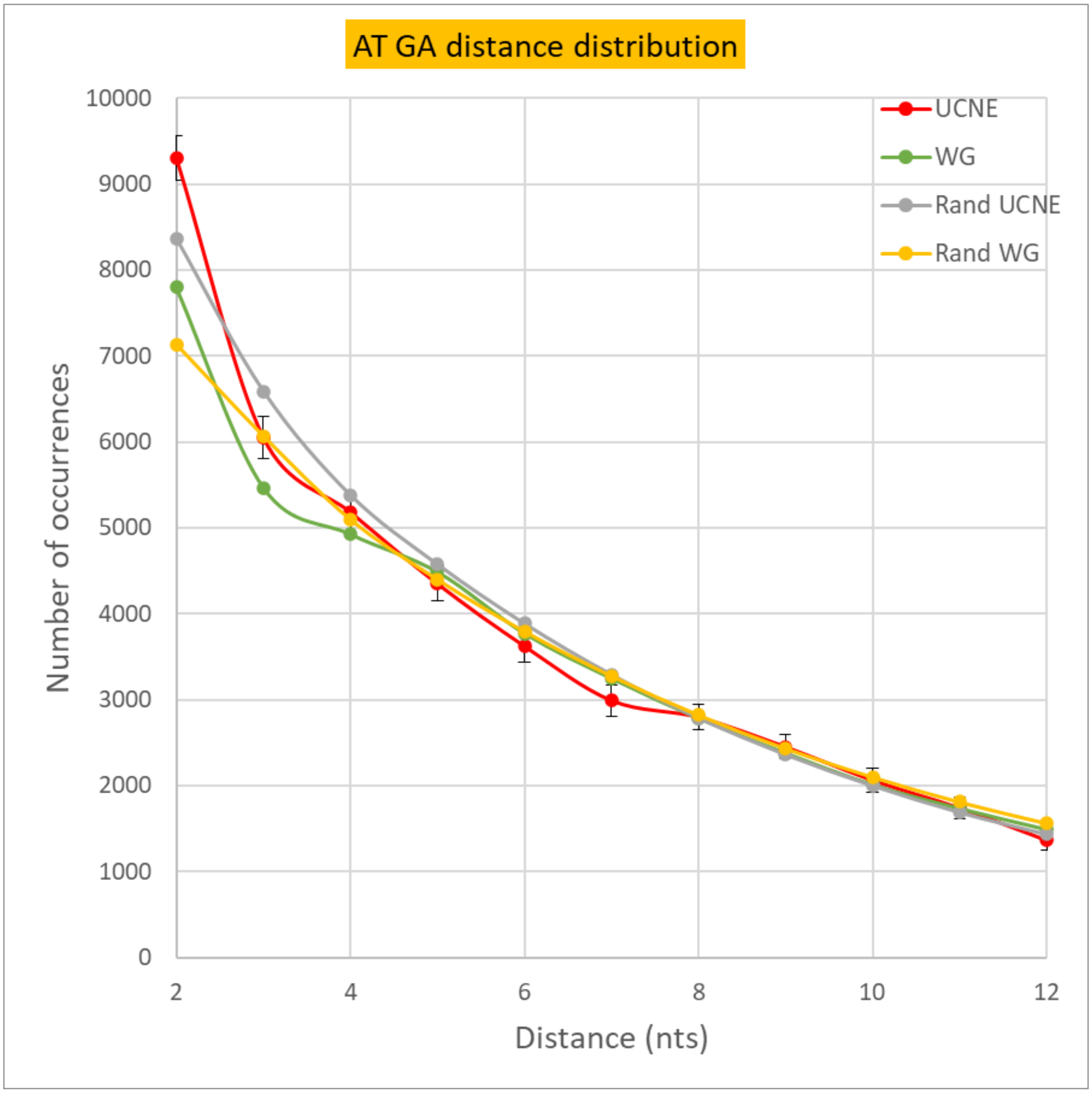

AT GA distance distribution
UCNE
WG
Rand UCNE
Rand WG
Number of occurrences
10000
9000
8000
7000
6000
5000
4000
3000
2000
1000
0
2
4
6
8
10
12
Distance (nts)

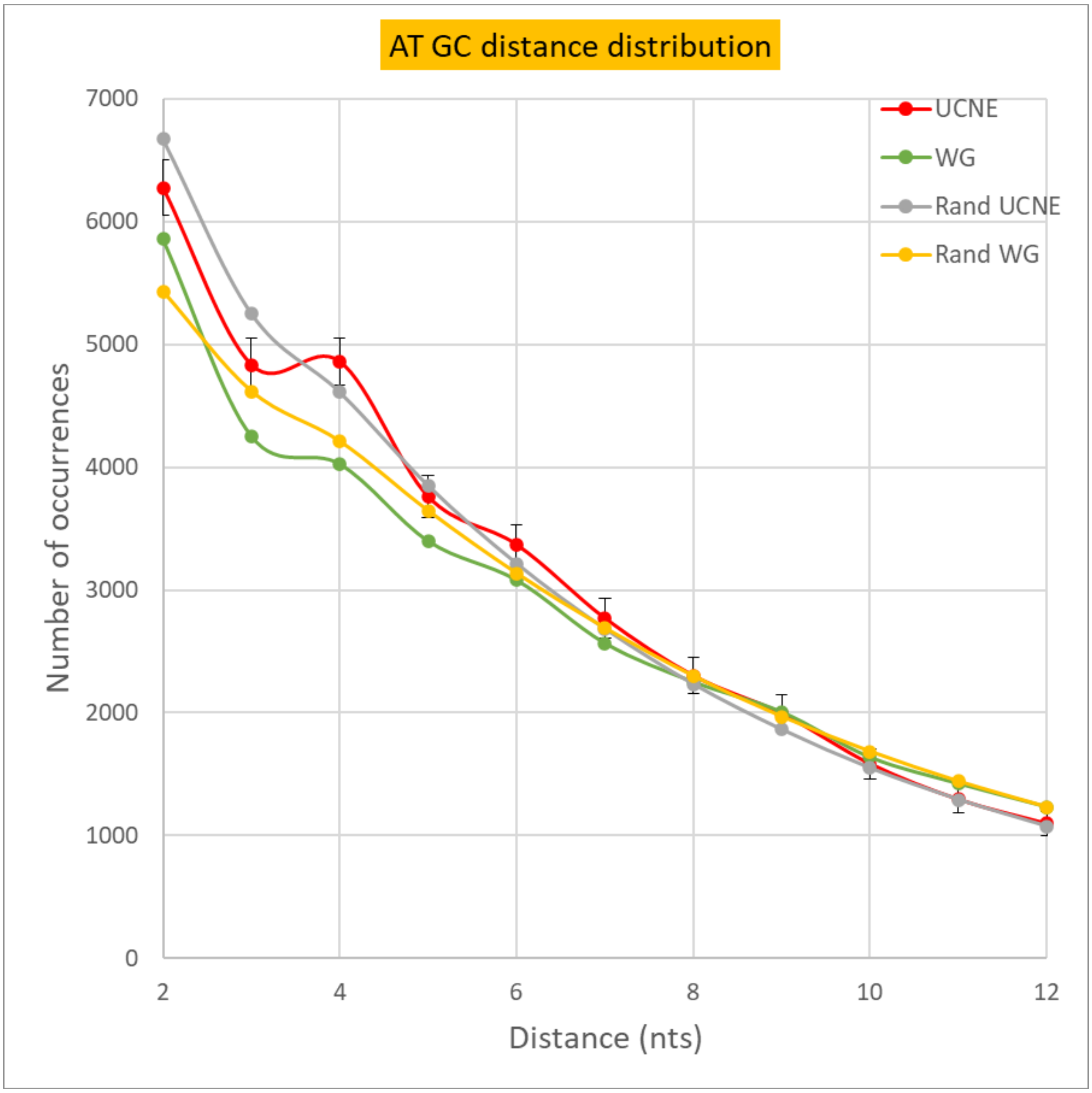
AT GC distance distribution
UCNE
WG
Rand UCNE
Rand WG
7000
6000
5000
4000
3000
2000
1000
0
Number of occurrences
2
4
6
8
10
12
Distance (nts)

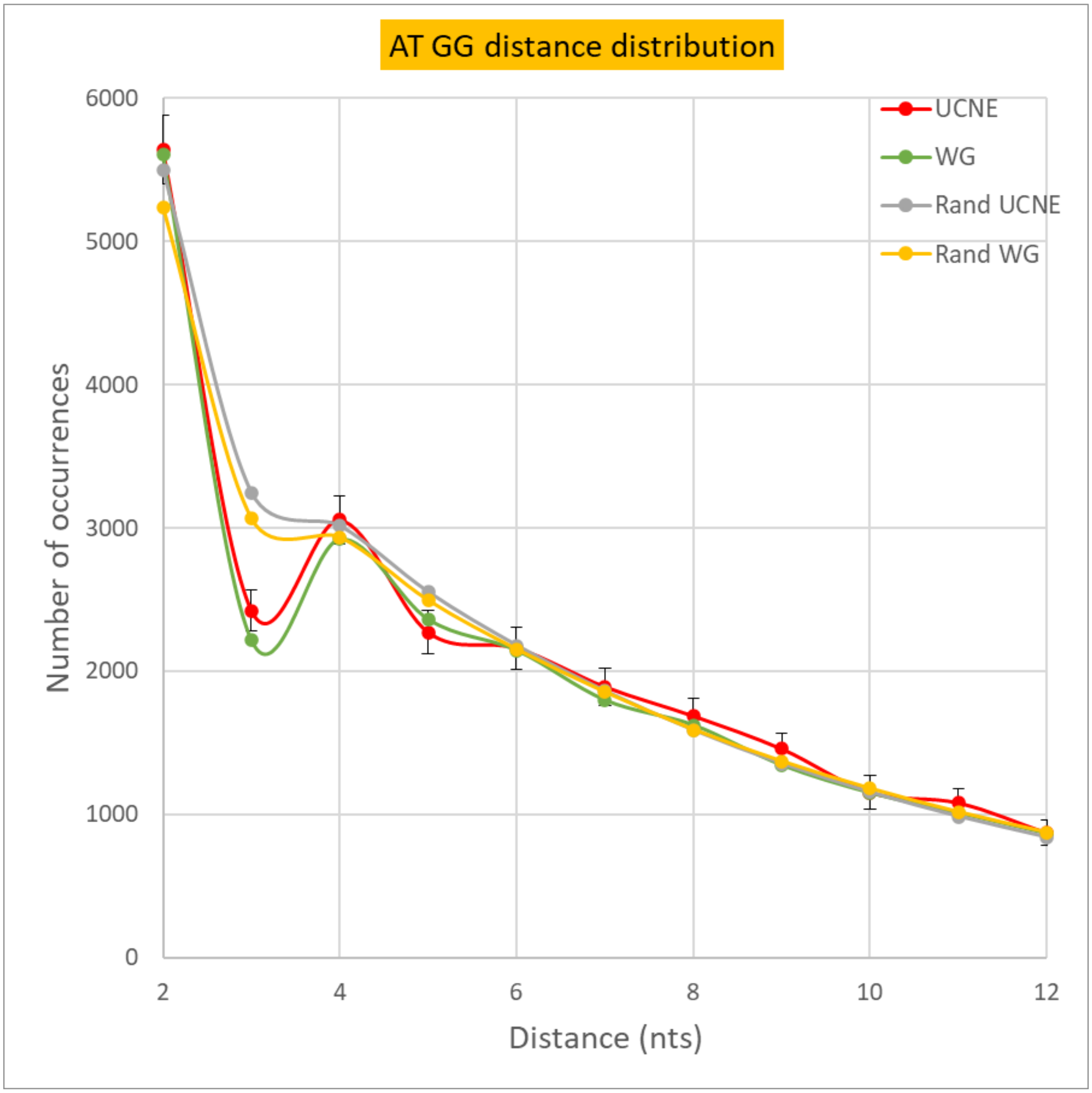

AT GG distance distribution
6000
5000
4000
3000
2000
1000
0
Number of occurrences
2
4
6
8
10
12
Distance (nts)
UCNE
WG
Rand UCNE
Rand WG

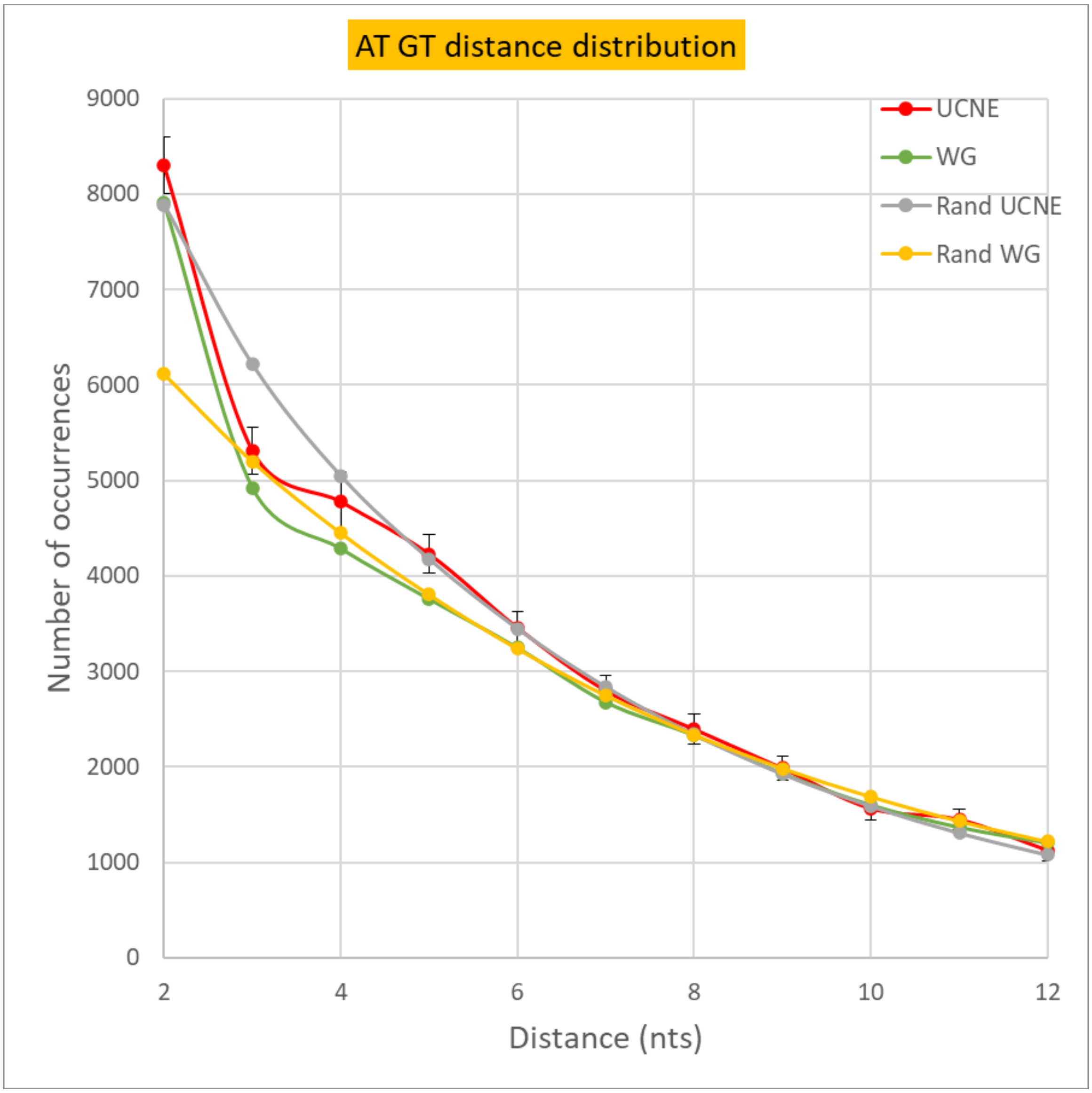
AT GT distance distribution
UCNE
WG
Rand UCNE
Rand WG
Number of occurrences
0
1000
2000
3000
4000
5000
6000
7000
8000
9000
2
4
6
8
10
12
Distance (nts)

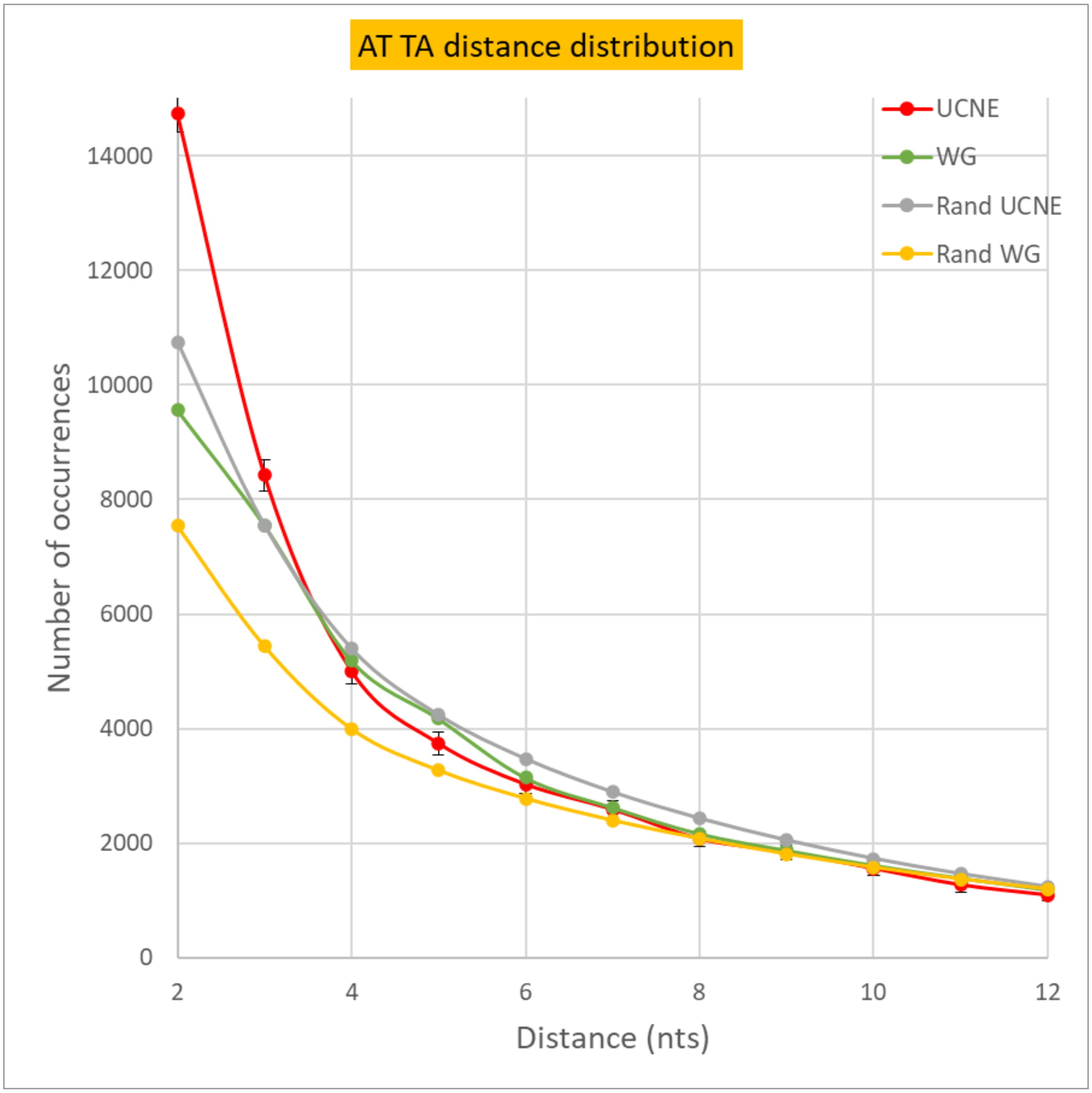

AT TA distance distribution
UCNE
WG
Rand UCNE
Rand WG
Number of occurrences
0
2000
4000
6000
8000
10000
12000
14000
2
4
6
8
10
12
Distance (nts)

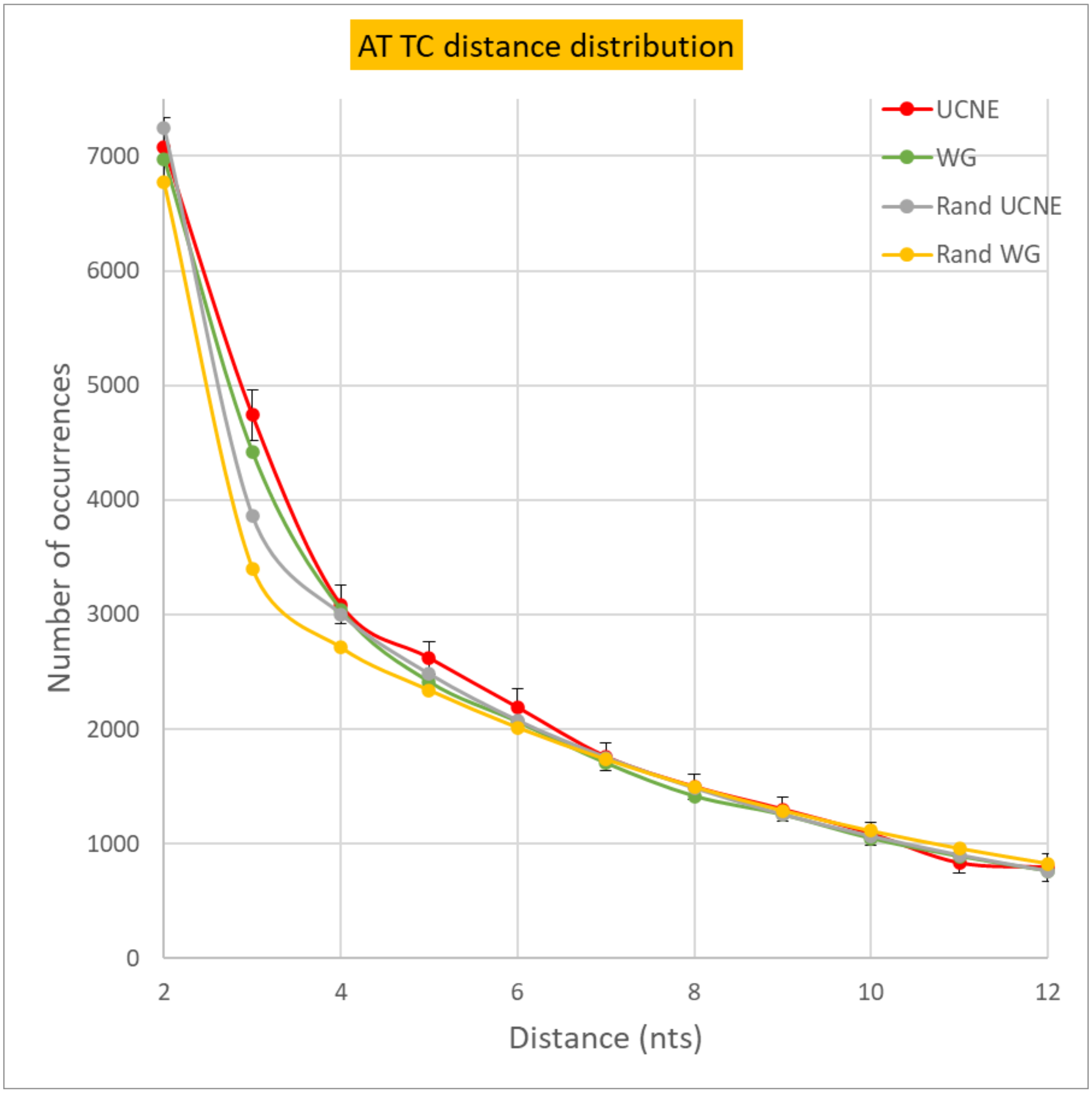
AT TC distance distribution
UCNE
WG
Rand UCNE
Rand WG
Number of occurrences
0
1000
2000
3000
4000
5000
6000
7000
2
4
6
8
10
12
Distance (nts)

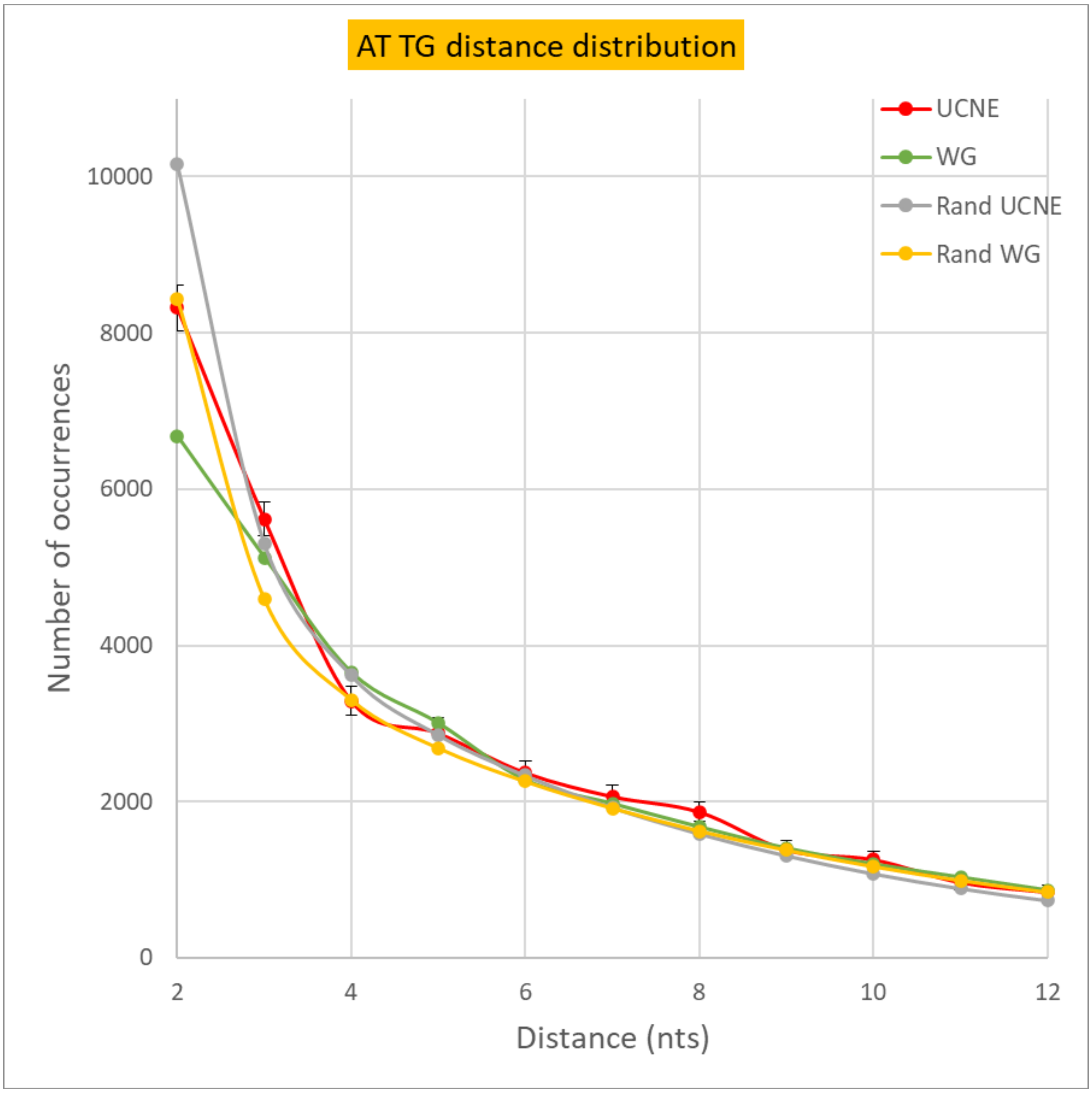

AT TG distance distribution
UCNE
WG
Rand UCNE
Rand WG
Number of occurrences
0
2000
4000
6000
8000
10000
2
4
6
8
10
12
Distance (nts)

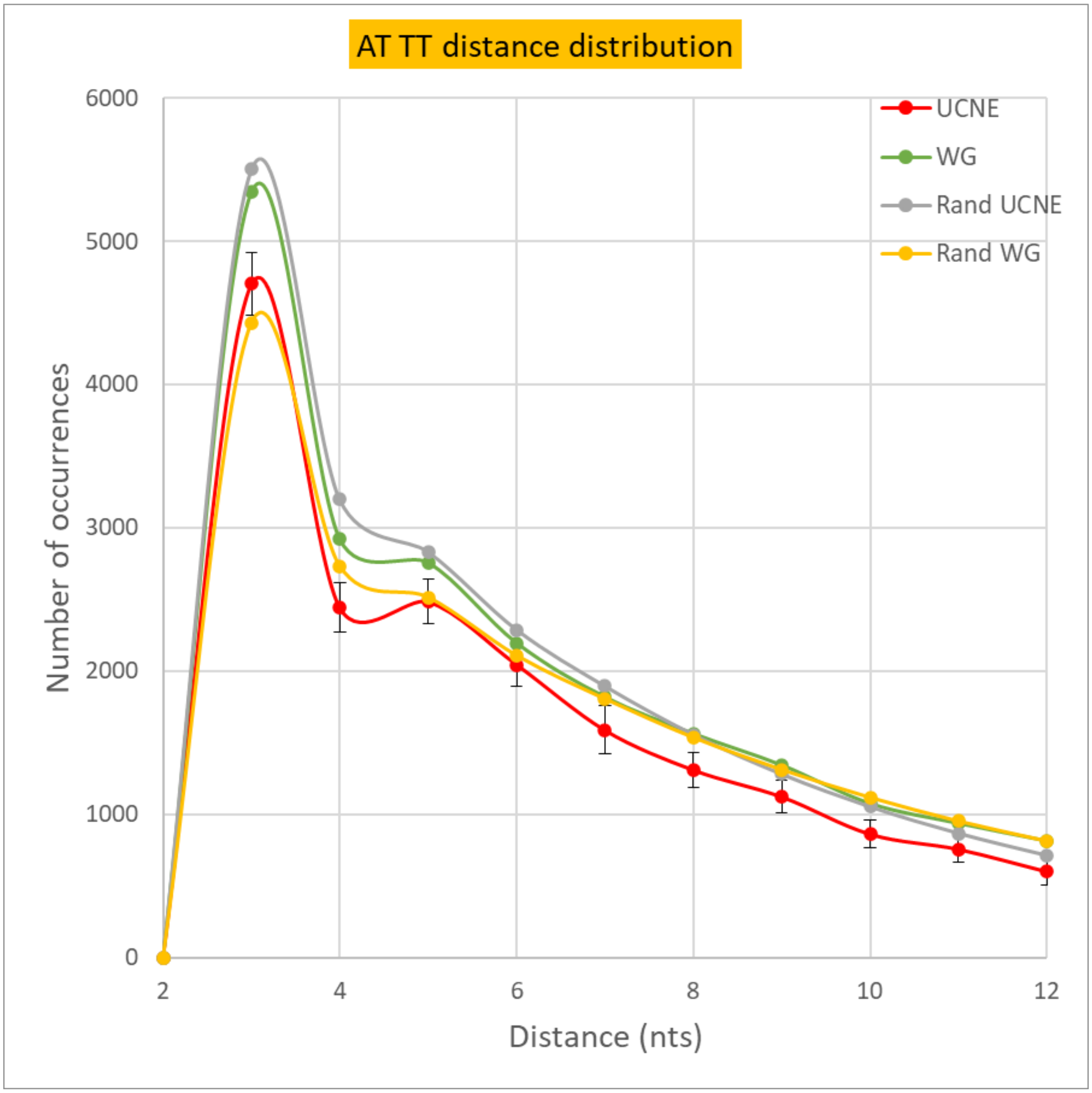

AT TT distance distribution
UCNE
WG
Rand UCNE
Rand WG
Number of occurrences
0
1000
2000
3000
4000
5000
6000
2
4
6
8
10
12
Distance (nts)

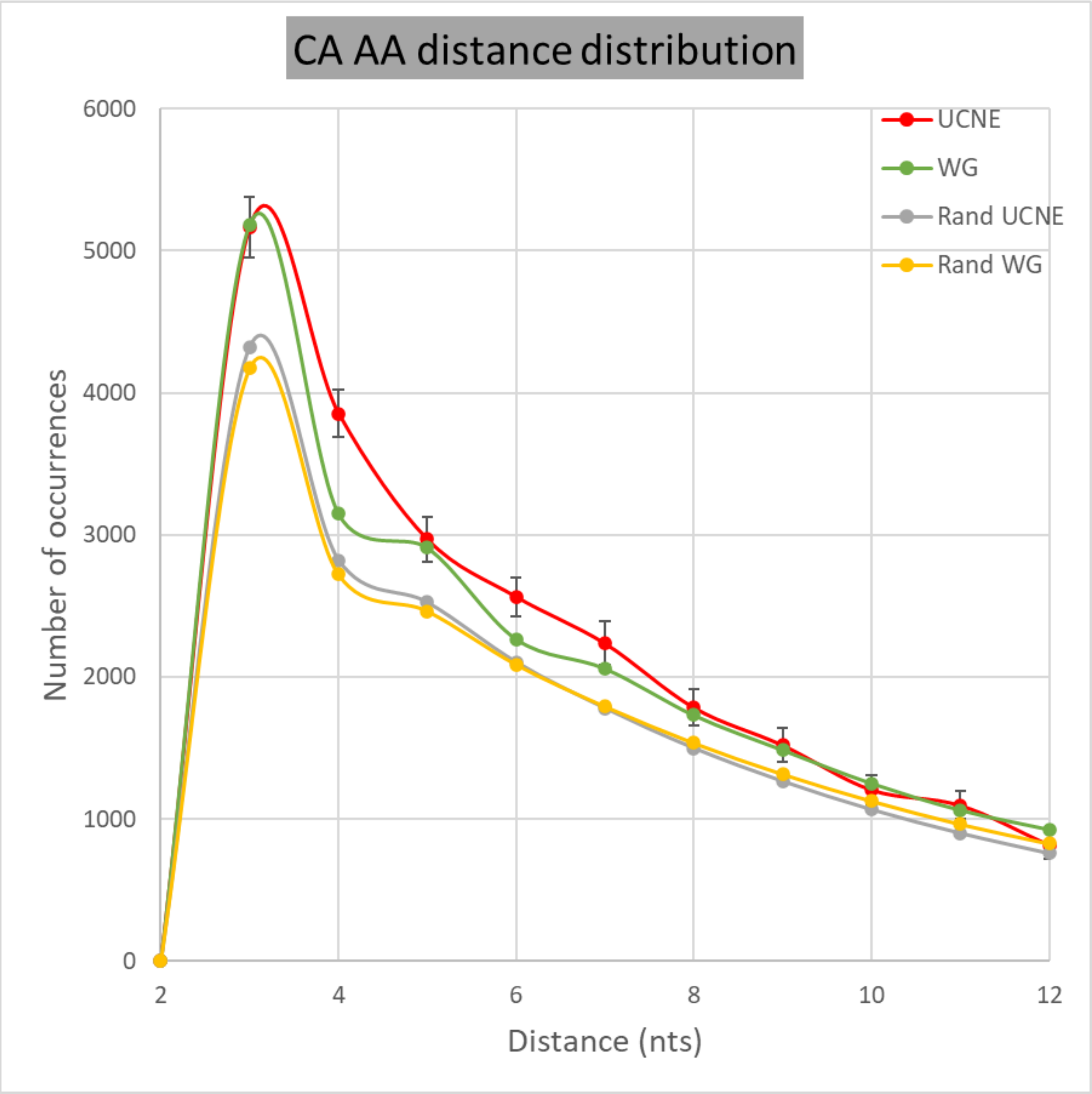

CA AA distance distribution
6000
5000
4000
3000
2000
1000
0
2
4
6
8
10
12
Number of occurrences
Distance (nts)
UCNE
WG
Rand UCNE
Rand WG

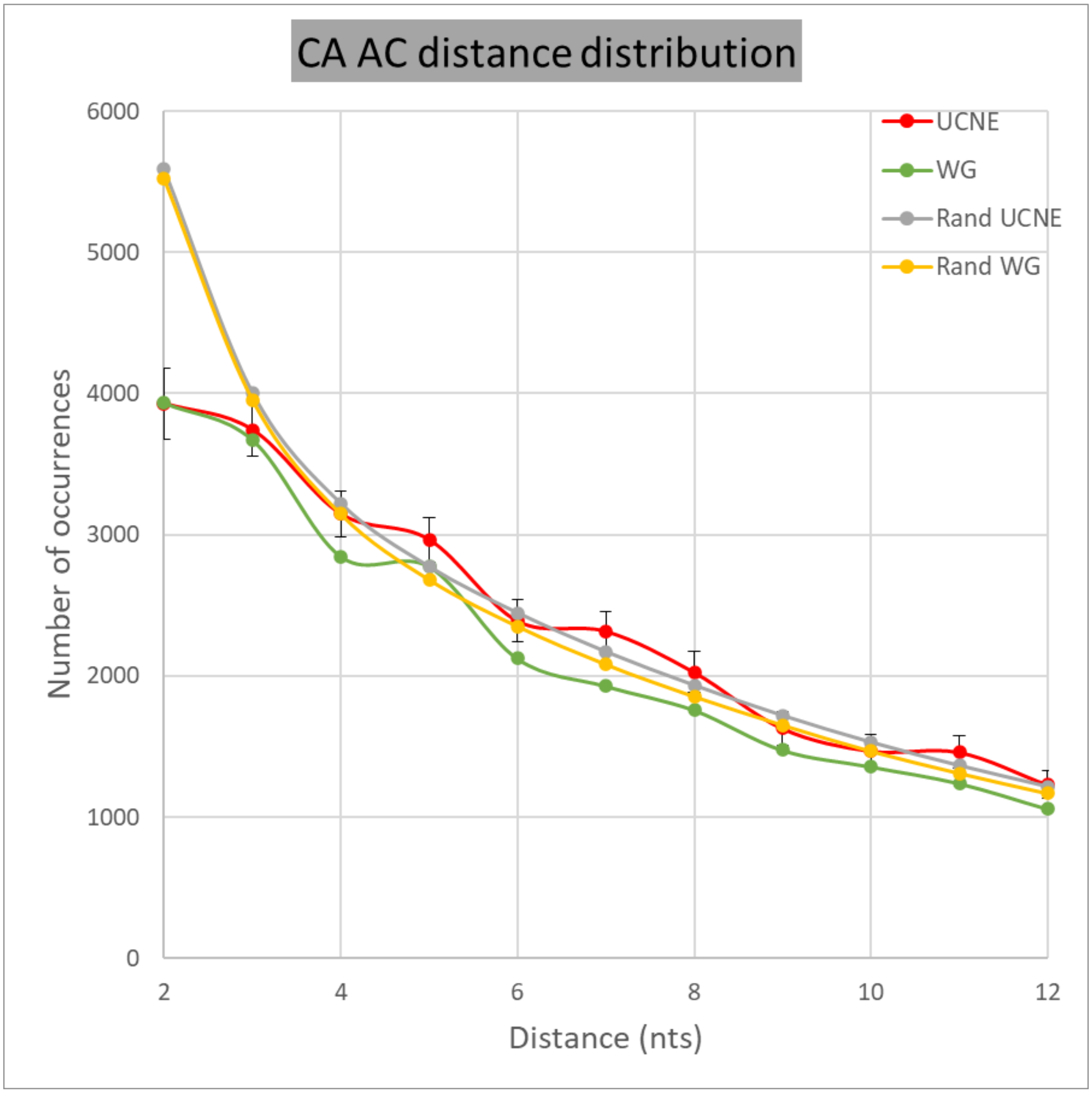
CA AC distance distribution
UCNE
WG
Rand UCNE
Rand WG
Number of occurrences
0
1000
2000
3000
4000
5000
6000
2
4
6
8
10
12
Distance (nts)

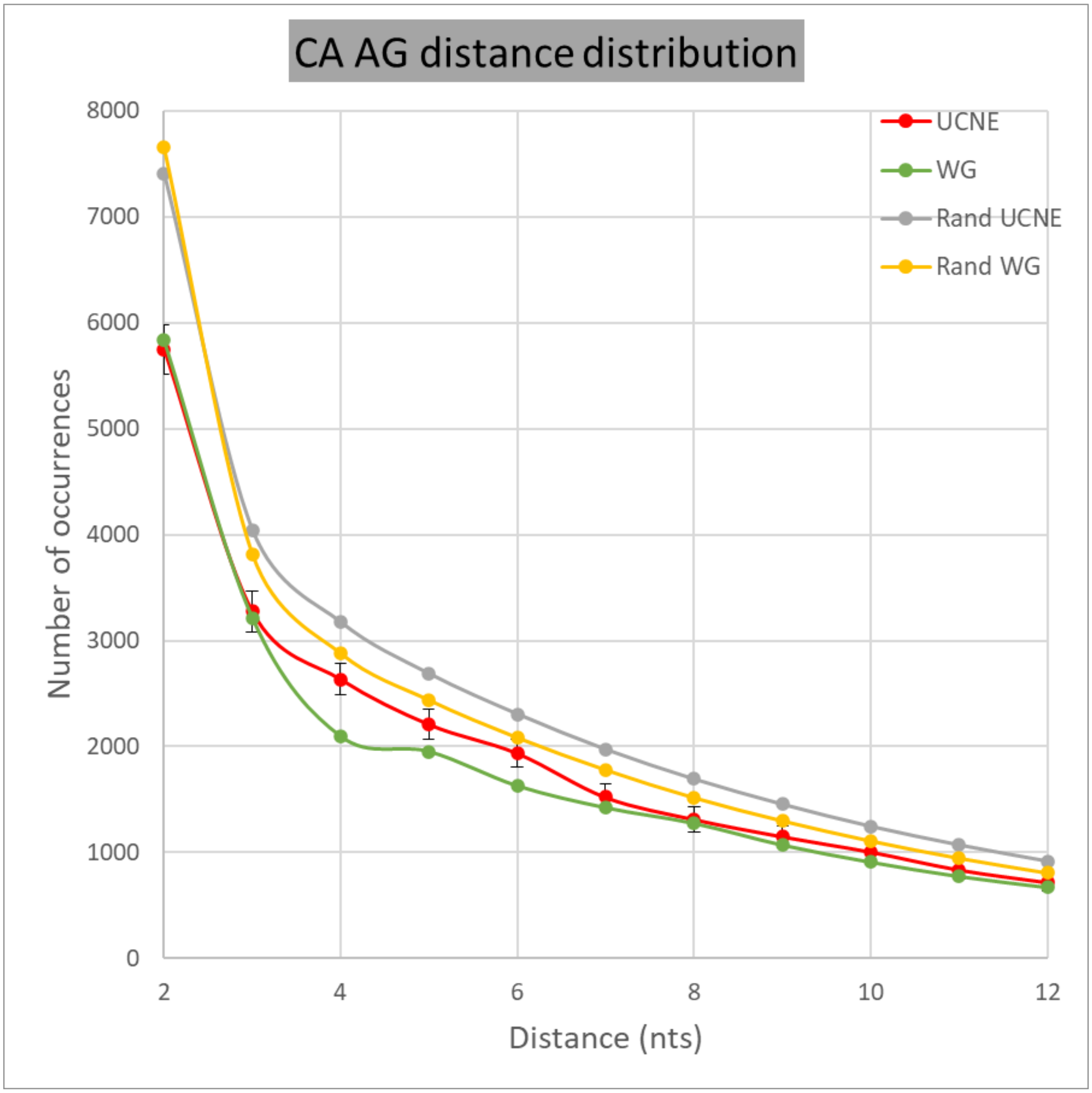

CA AG distance distribution
UCNE
WG
Rand UCNE
Rand WG
Number of occurrences
Distance (nts)
0
1000
2000
3000
4000
5000
6000
7000
8000
2
4
6
8
10
12

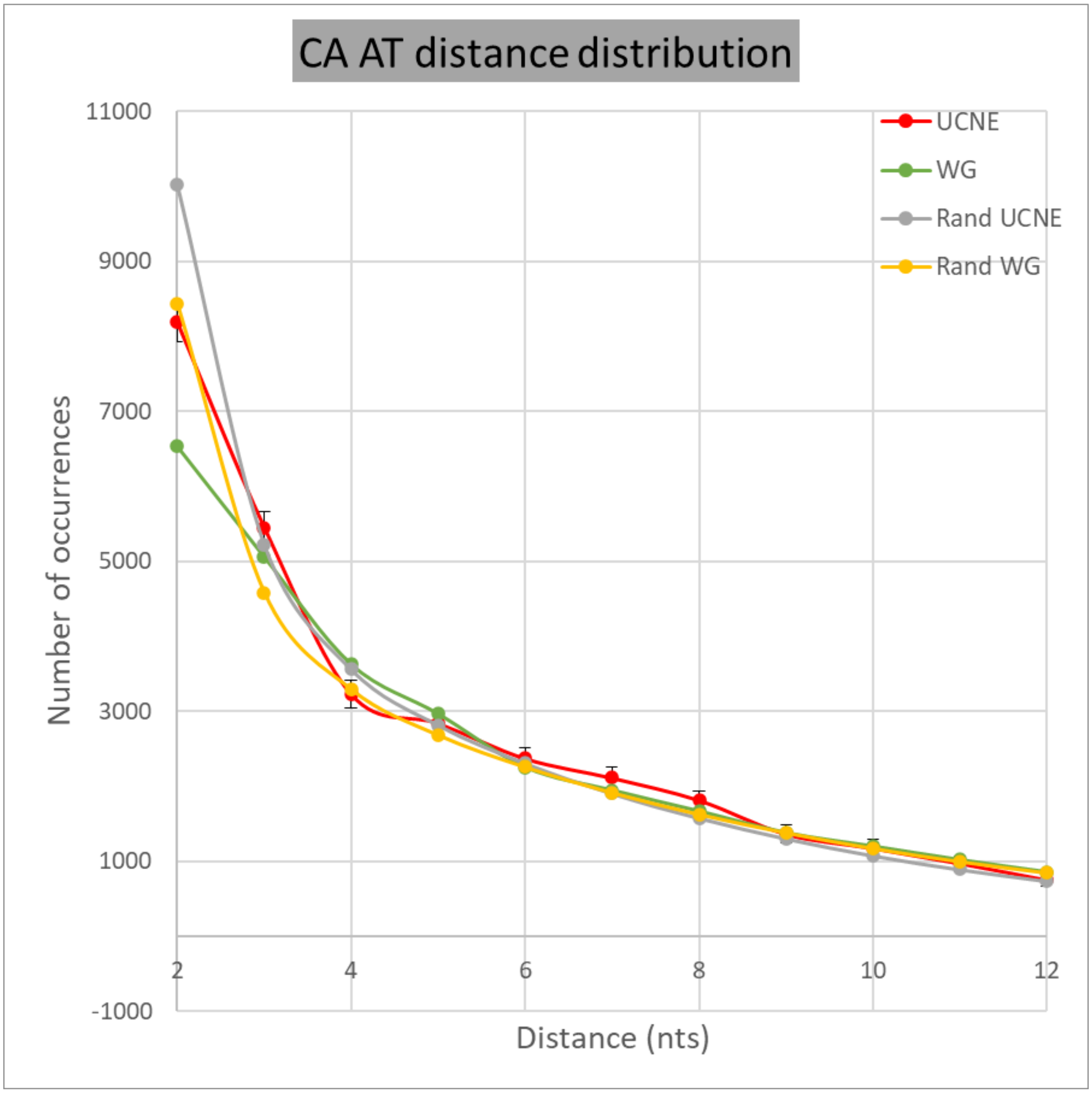

CA AT distance distribution
Number of occurrences
Distance (nts)
UCNE
WG
Rand UCNE
Rand WG
11000
9000
7000
5000
3000
1000
-1000
2
4
6
8
10
12

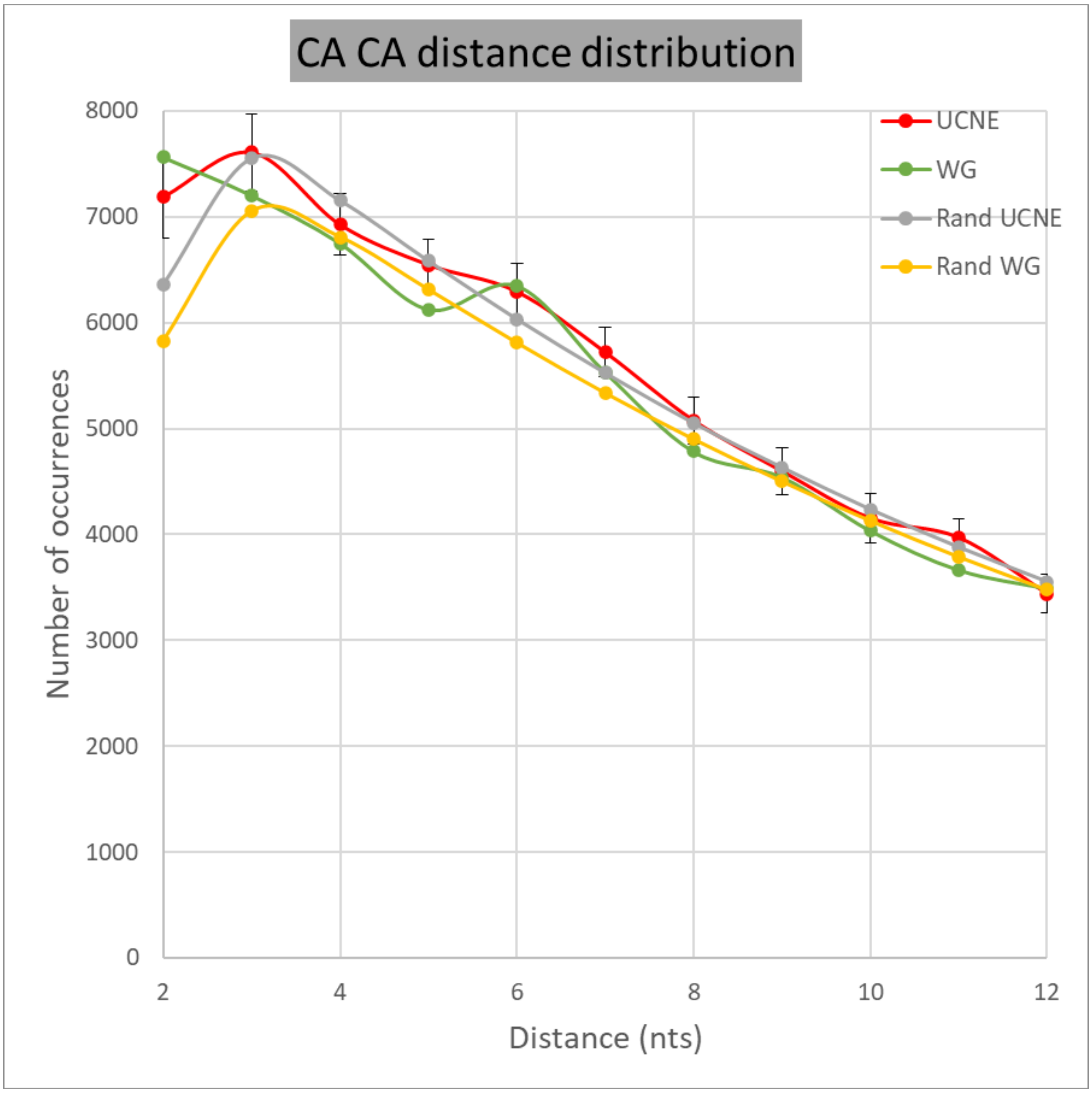
CA CA distance distribution
8000
7000
6000
5000
4000
3000
2000
1000
0
Number of occurrences
2
4
6
8
10
12
Distance (nts)
UCNE
WG
Rand UCNE
Rand WG

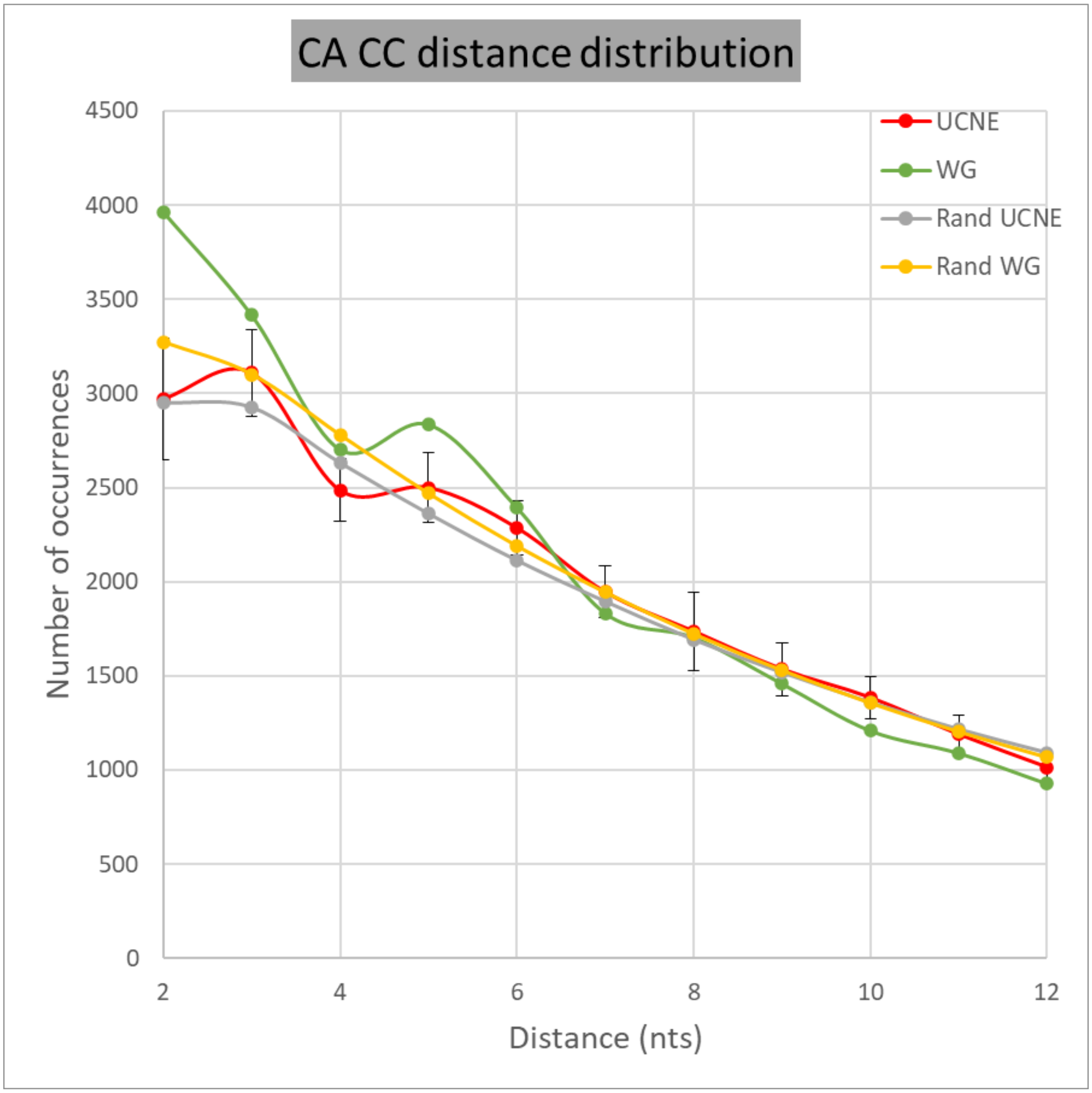
CA CC distance distribution
Number of occurrences
4500
4000
3500
3000
2500
2000
1500
1000
500
0
2
4
6
8
10
12
Distance (nts)
UCNE
WG
Rand UCNE
Rand WG

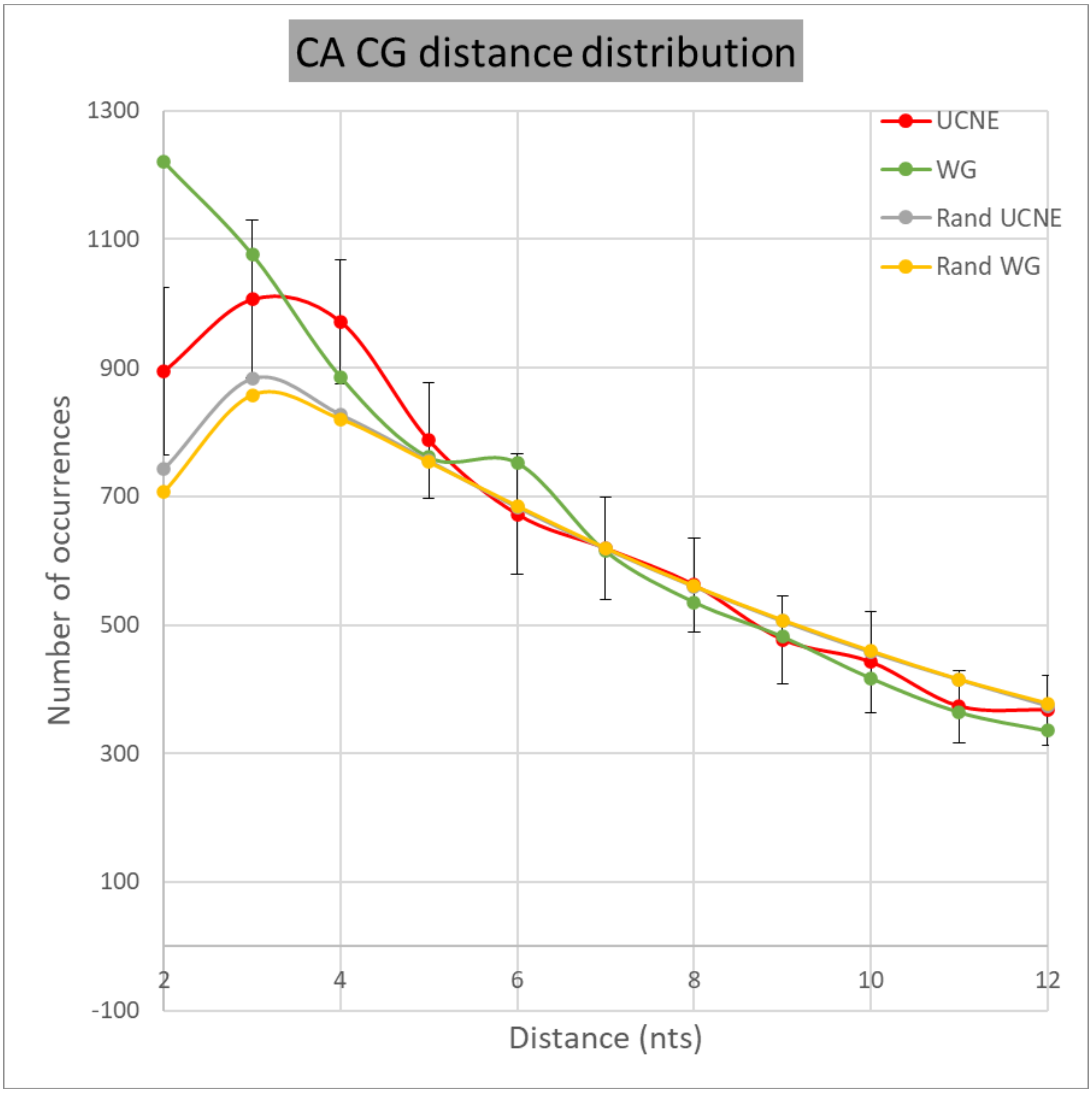
CA CG distance distribution
UCNE
WG
Rand UCNE
Rand WG
Number of occurrences
1300
1100
900
700
500
300
100
-100
2
4
6
8
10
12
Distance (nts)

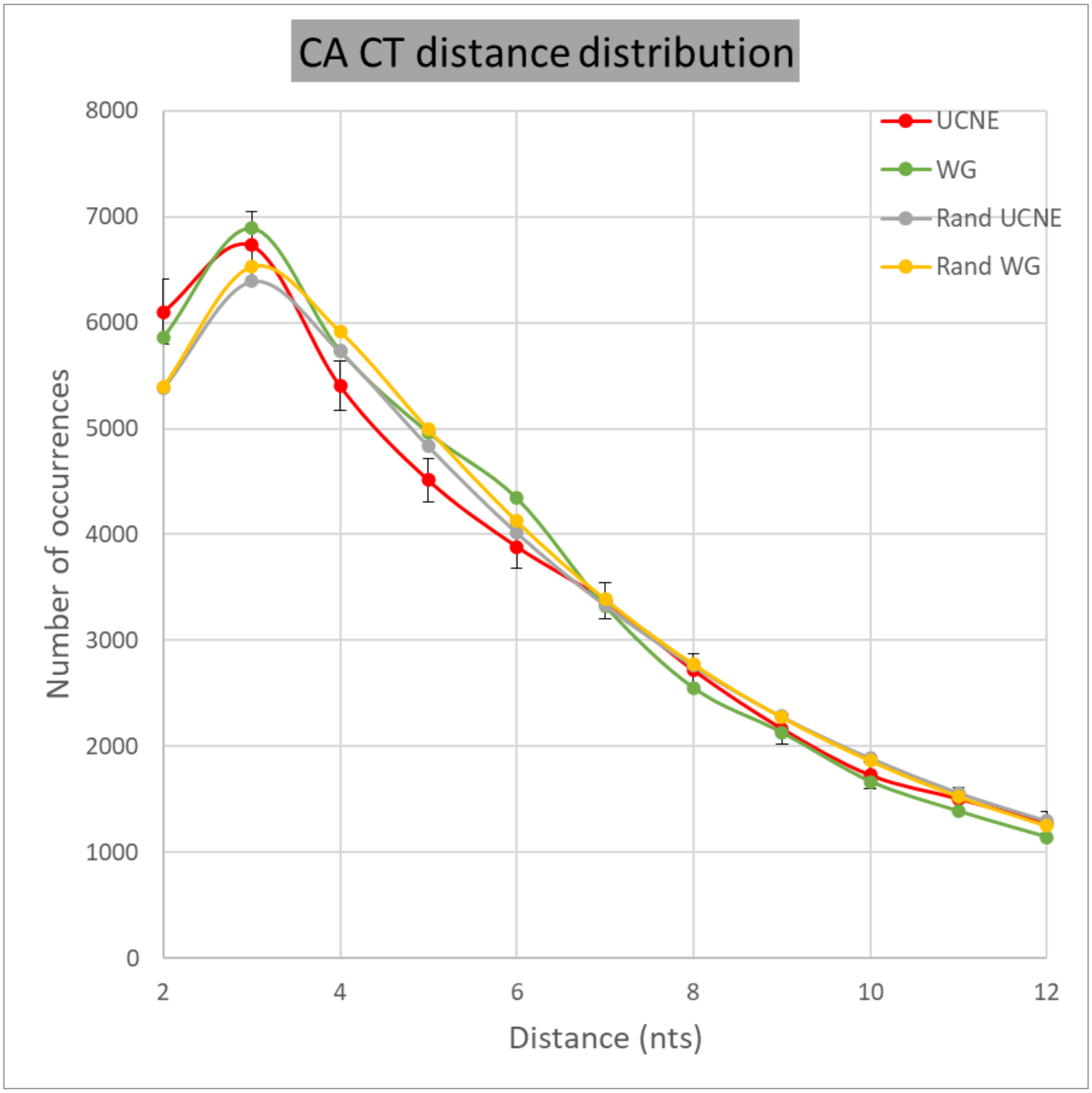
CA CT distance distribution
UCNE
WG
Rand UCNE
Rand WG
Number of occurrences
0
1000
2000
3000
4000
5000
6000
7000
8000
2
4
6
8
10
12
Distance (nts)

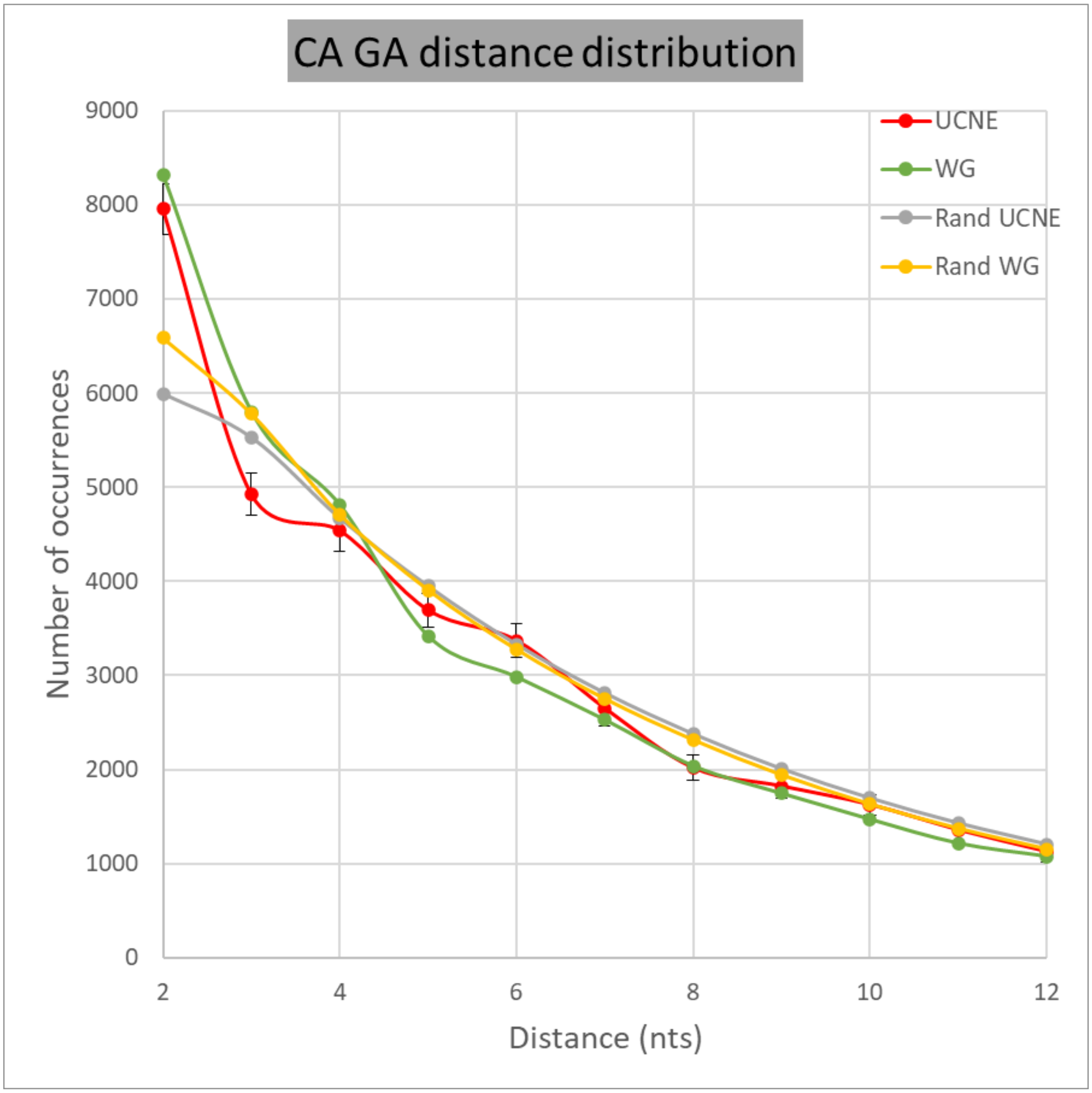

CA GA distance distribution
UCNE
WG
Rand UCNE
Rand WG
Number of occurrences
0
1000
2000
3000
4000
5000
6000
7000
8000
9000
2
4
6
8
10
12
Distance (nts)

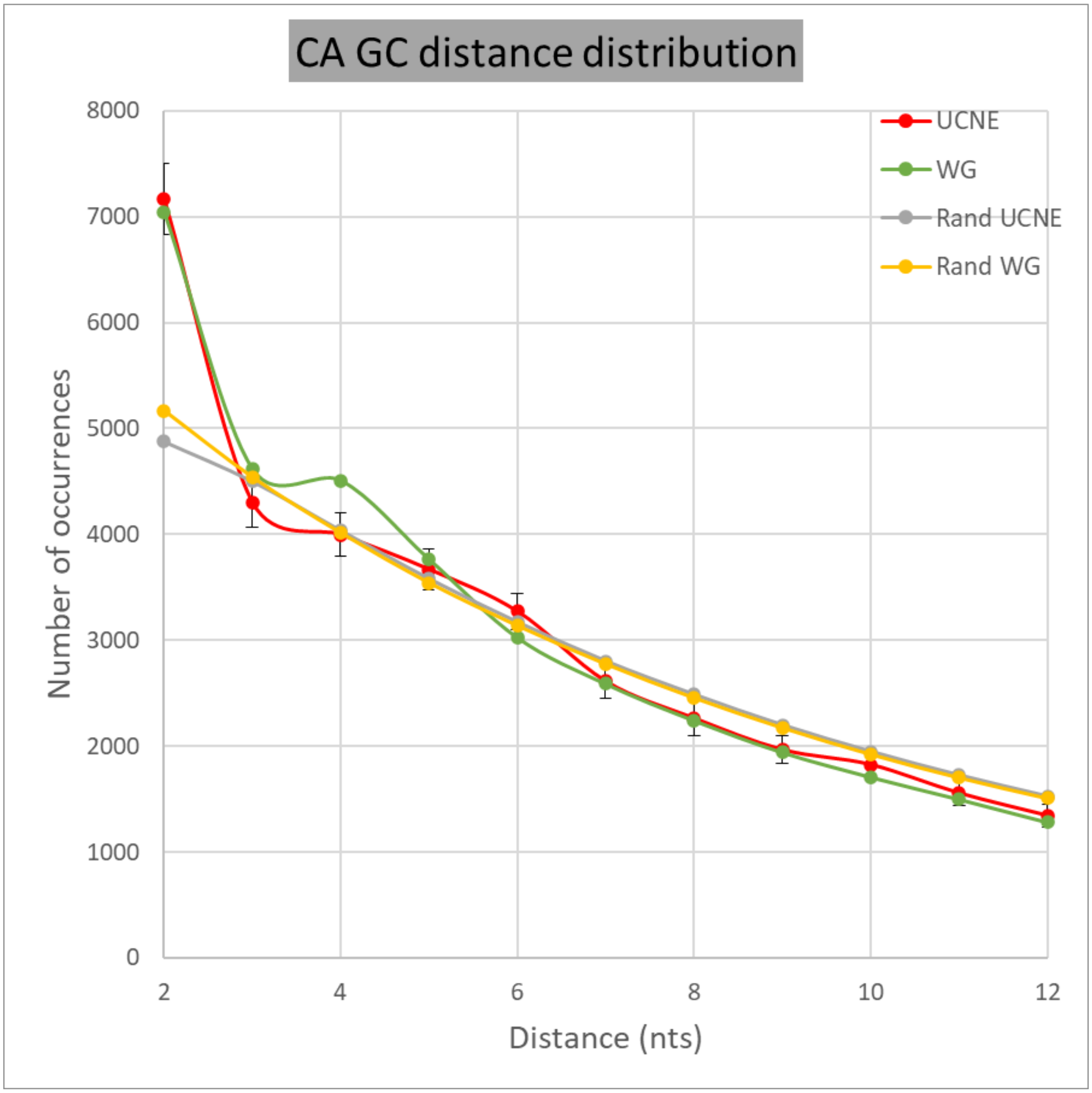

CA GC distance distribution
8000
7000
6000
5000
4000
3000
2000
1000
0
Number of occurrences
2
4
6
8
10
12
Distance (nts)
UCNE
WG
Rand UCNE
Rand WG

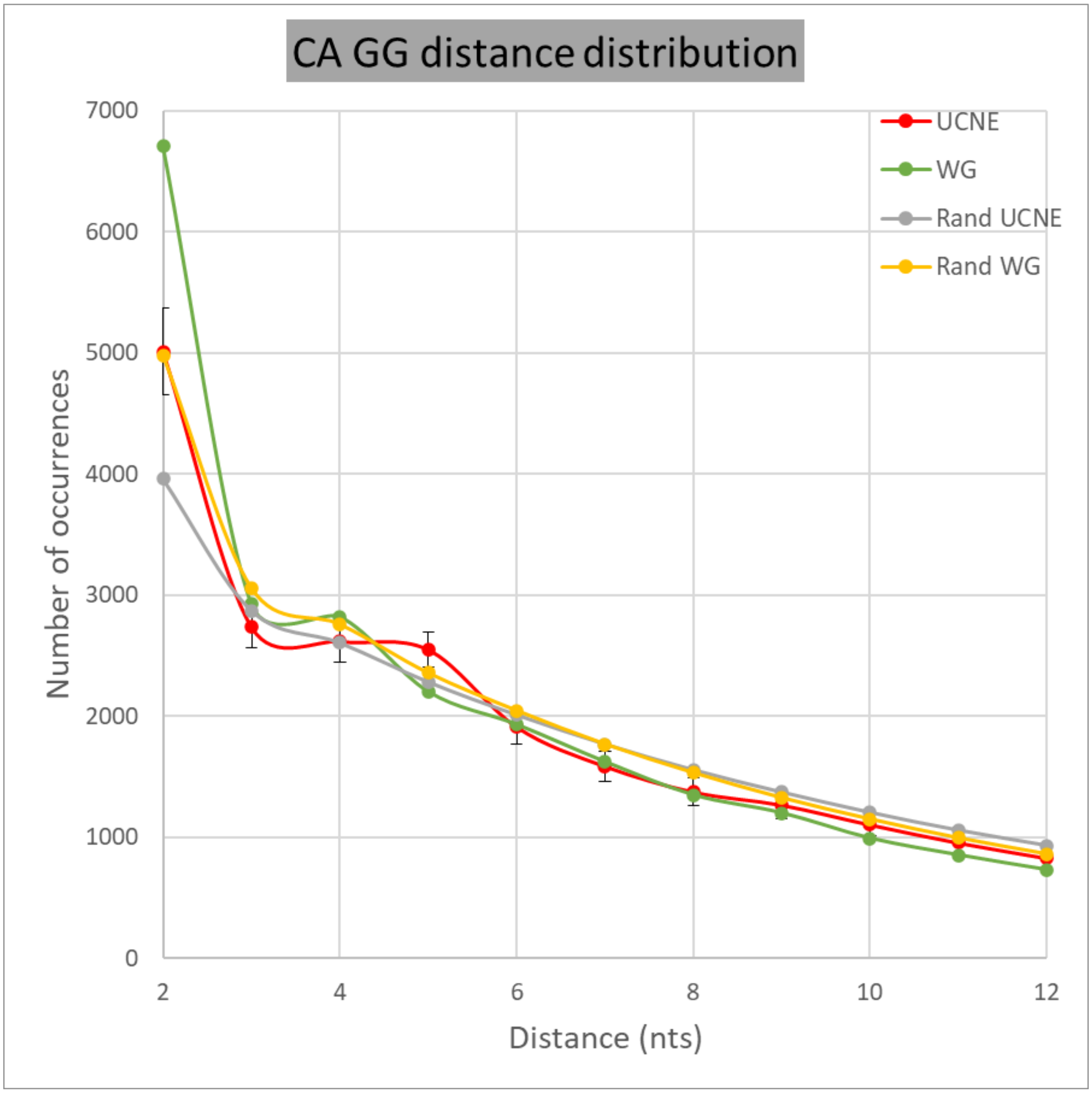
CA GG distance distribution
UCNE
WG
Rand UCNE
Rand WG
Number of occurrences
7000
6000
5000
4000
3000
2000
1000
0
2
4
6
8
10
12
Distance (nts)

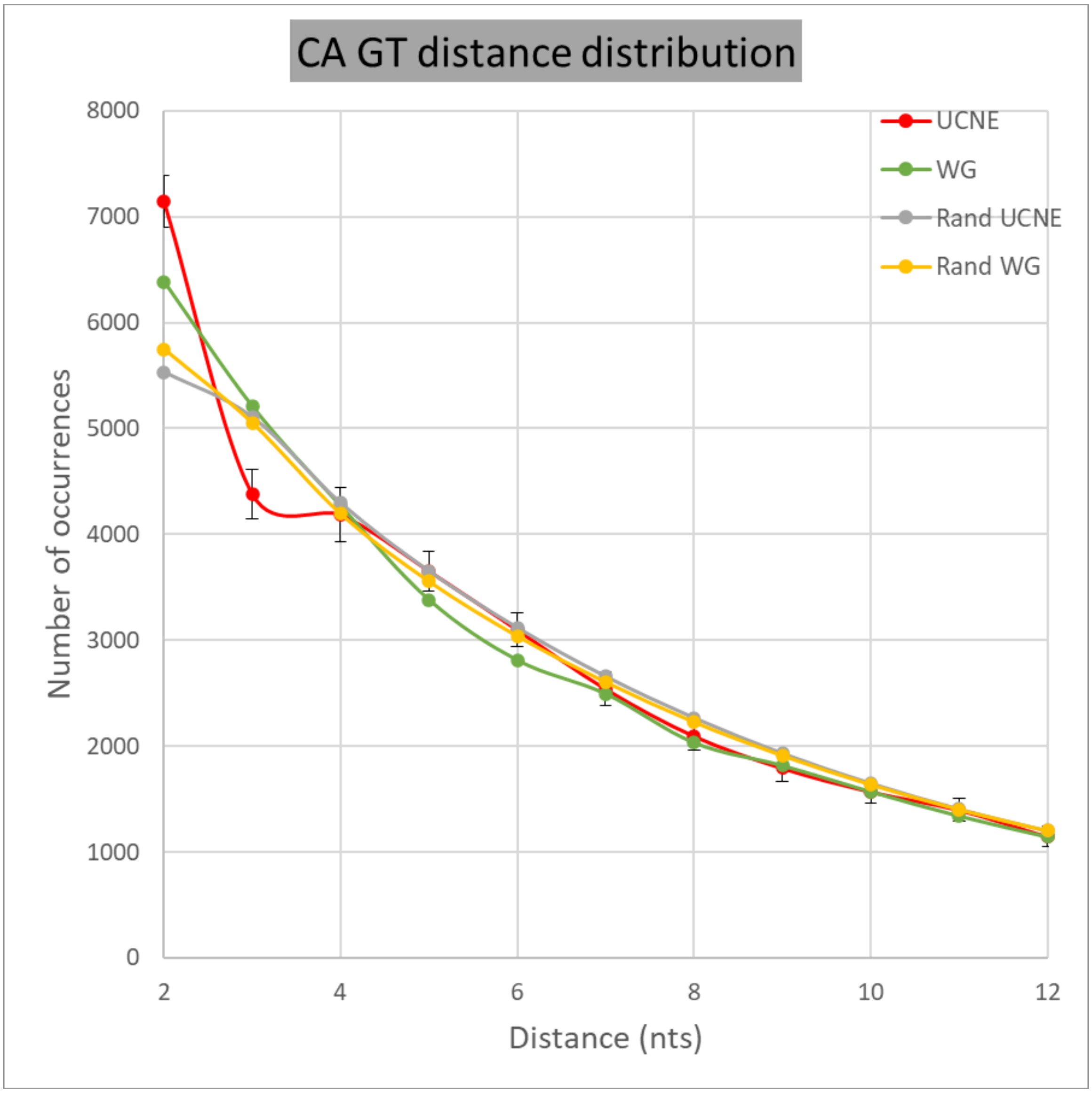
CA GT distance distribution
UCNE
WG
Rand UCNE
Rand WG
Number of occurrences
8000
7000
6000
5000
4000
3000
2000
1000
0
2
4
6
8
10
12
Distance (nts)

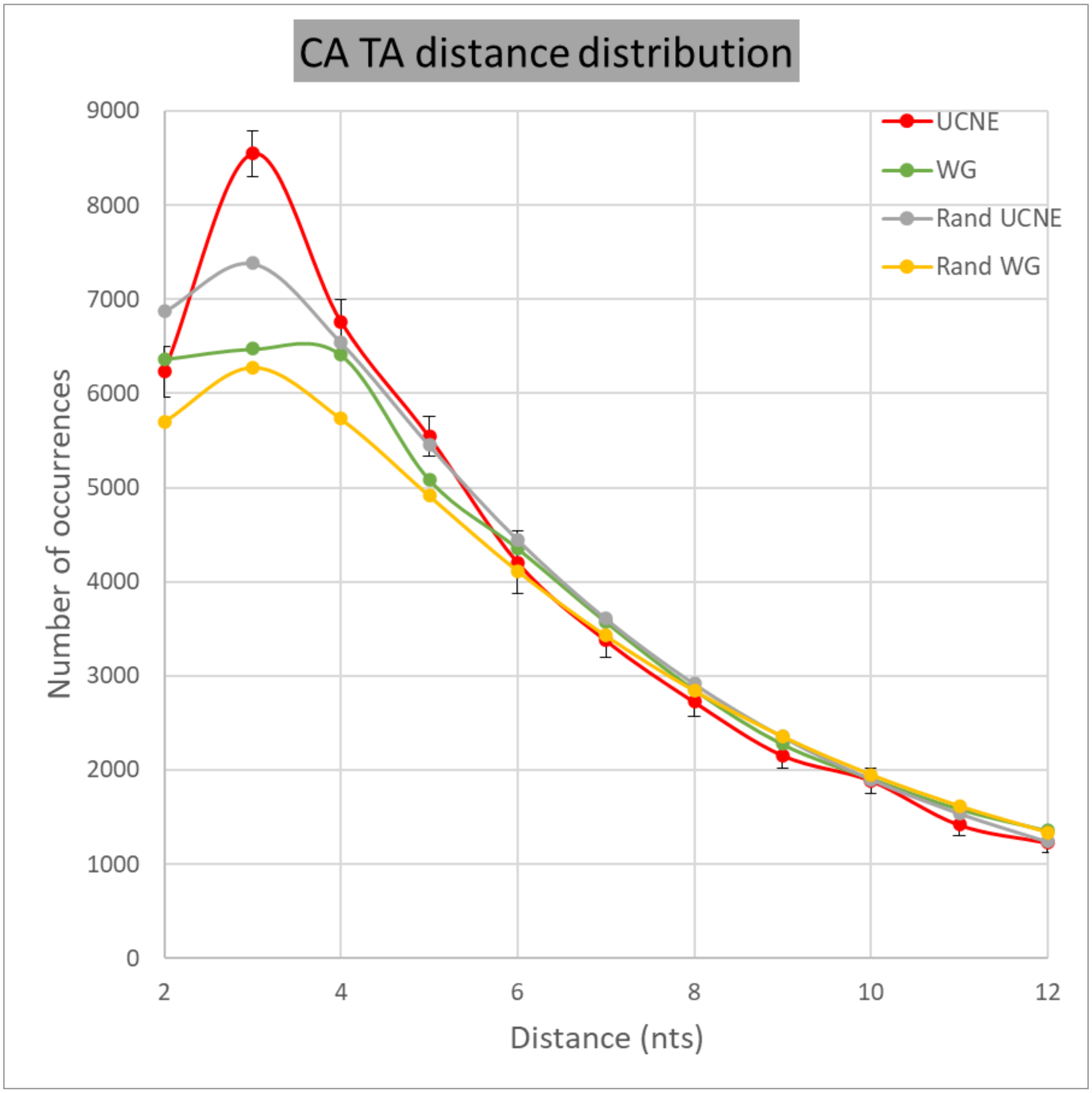
CA TA distance distribution
UCNE
WG
Rand UCNE
Rand WG
Number of occurrences
Distance (nts)
9000
8000
7000
6000
5000
4000
3000
2000
1000
0
2
4
6
8
10
12

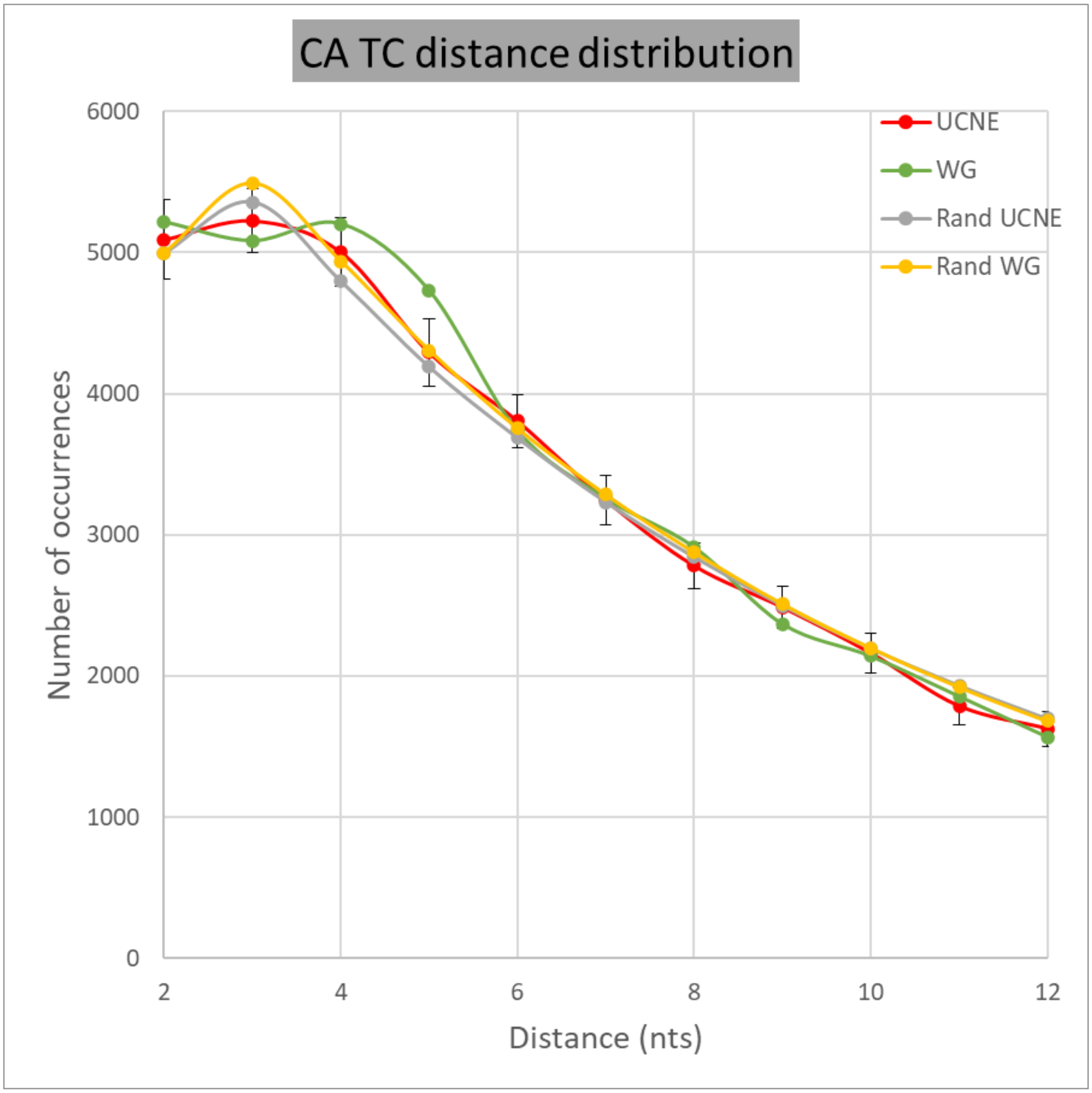

CA TC distance distribution
UCNE
WG
Rand UCNE
Rand WG
Number of occurrences
6000
5000
4000
3000
2000
1000
0
2
4
6
8
10
12
Distance (nts)

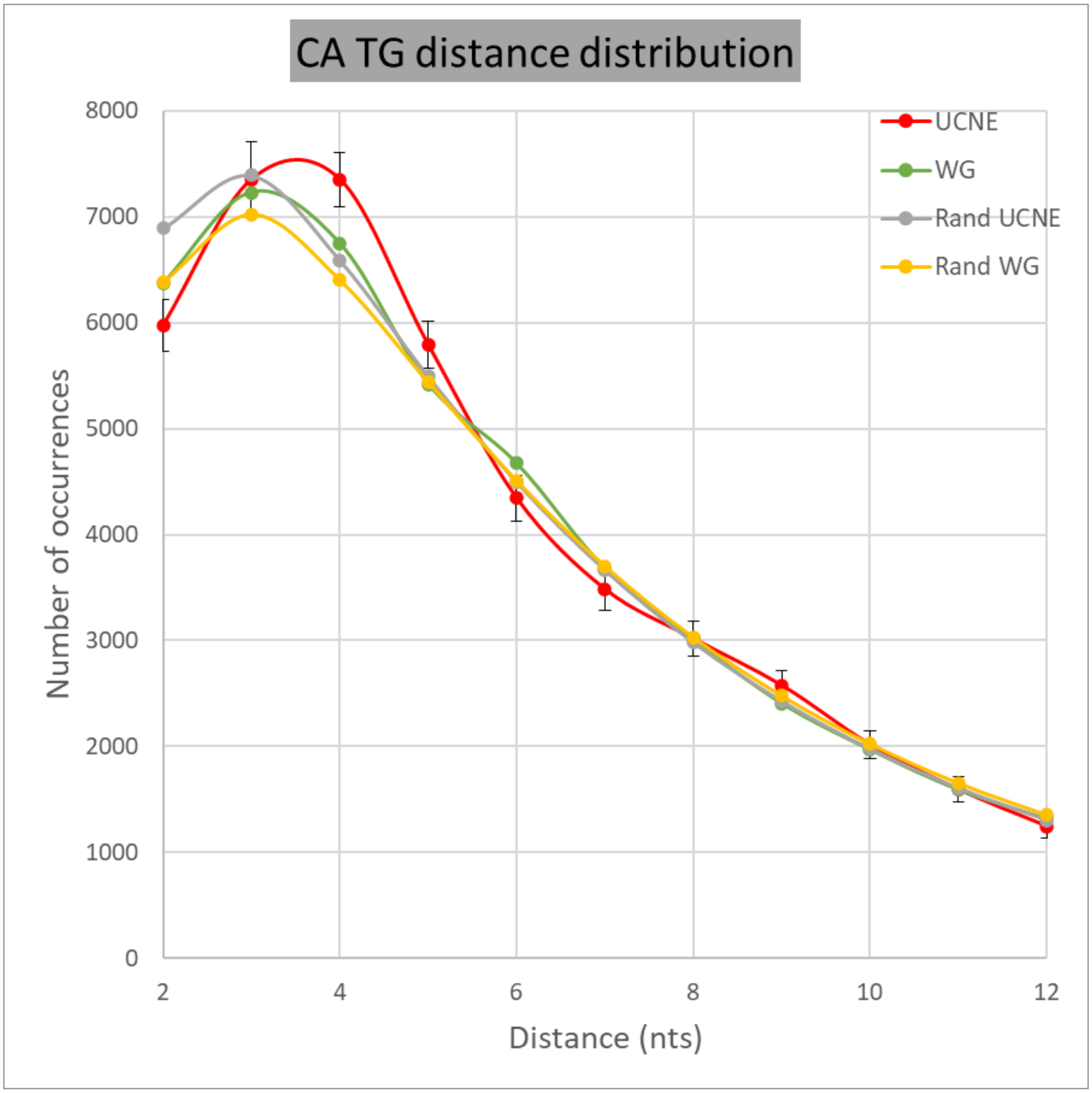

CA TG distance distribution
UCNE
WG
Rand UCNE
Rand WG
Number of occurrences
Distance (nts)
0
1000
2000
3000
4000
5000
6000
7000
8000
2
4
6
8
10
12

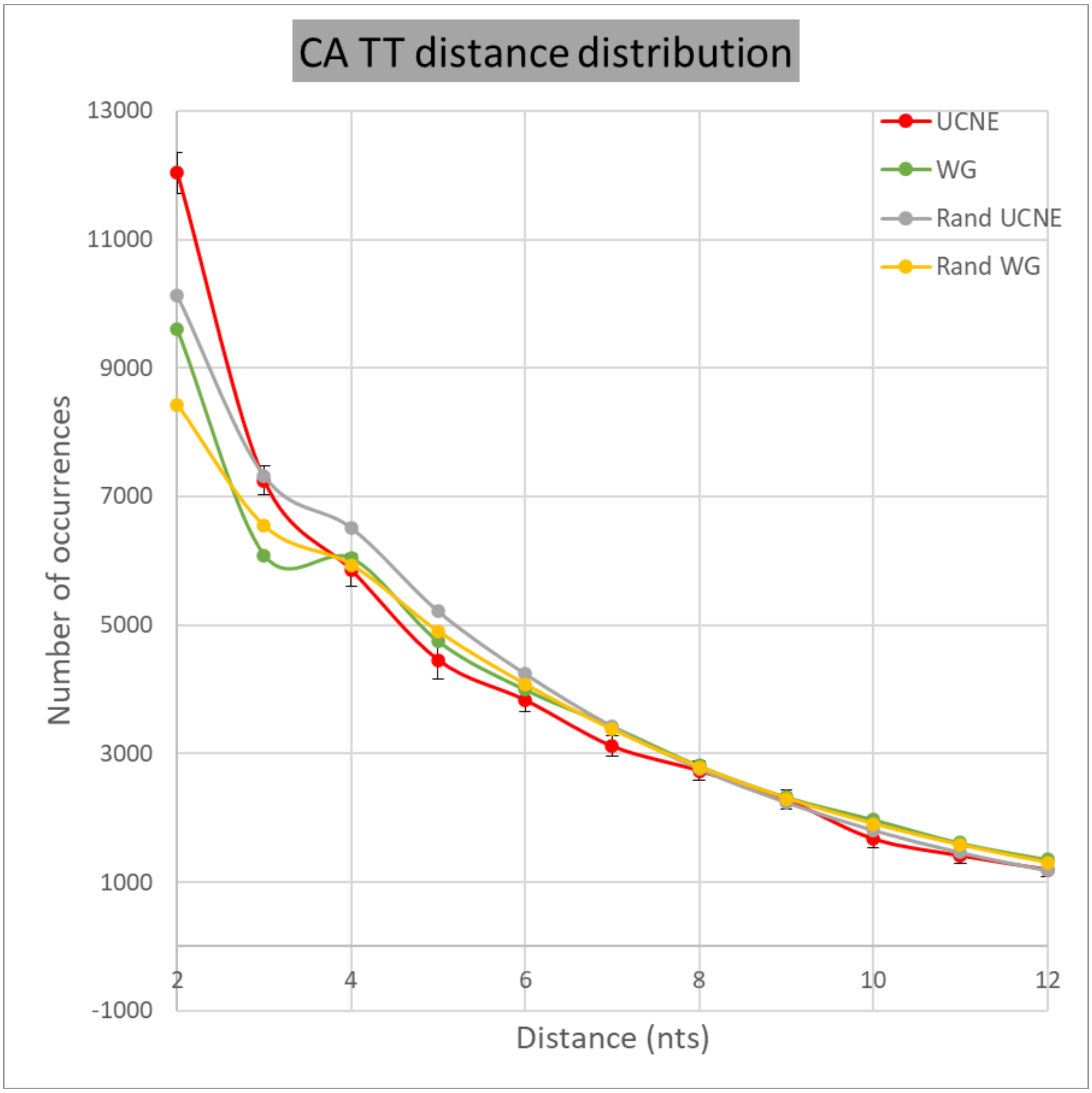
CA TT distance distribution
13000
11000
9000
7000
5000
3000
1000
-1000
Number of occurrences
2
4
6
8
10
12
Distance (nts)
UCNE
WG
Rand UCNE
Rand WG

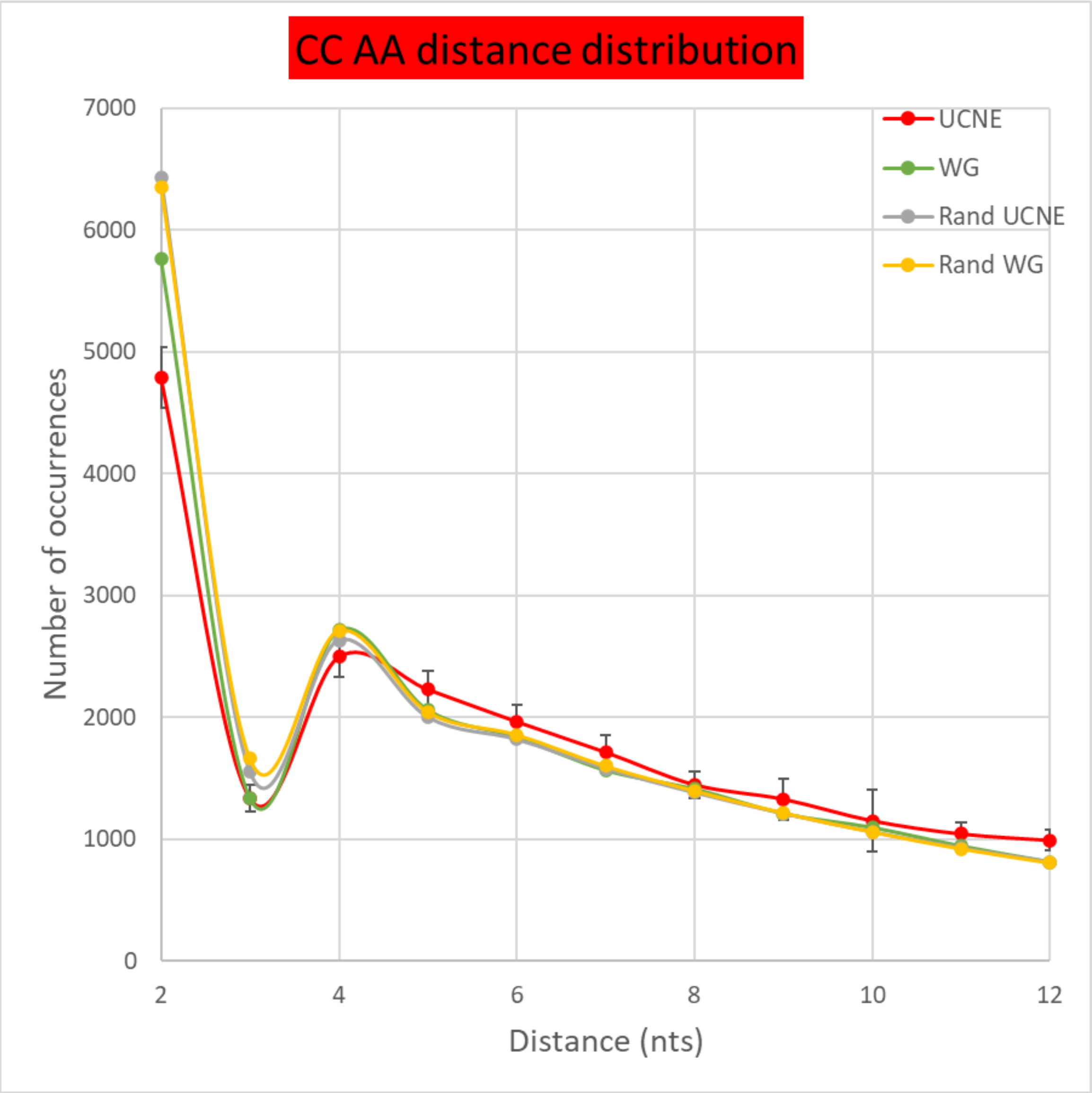
CC AA distance distribution
UCNE
WG
Rand UCNE
Rand WG
7000
6000
5000
4000
3000
2000
1000
0
2
4
6
8
10
12
Number of occurrences
Distance (nts)

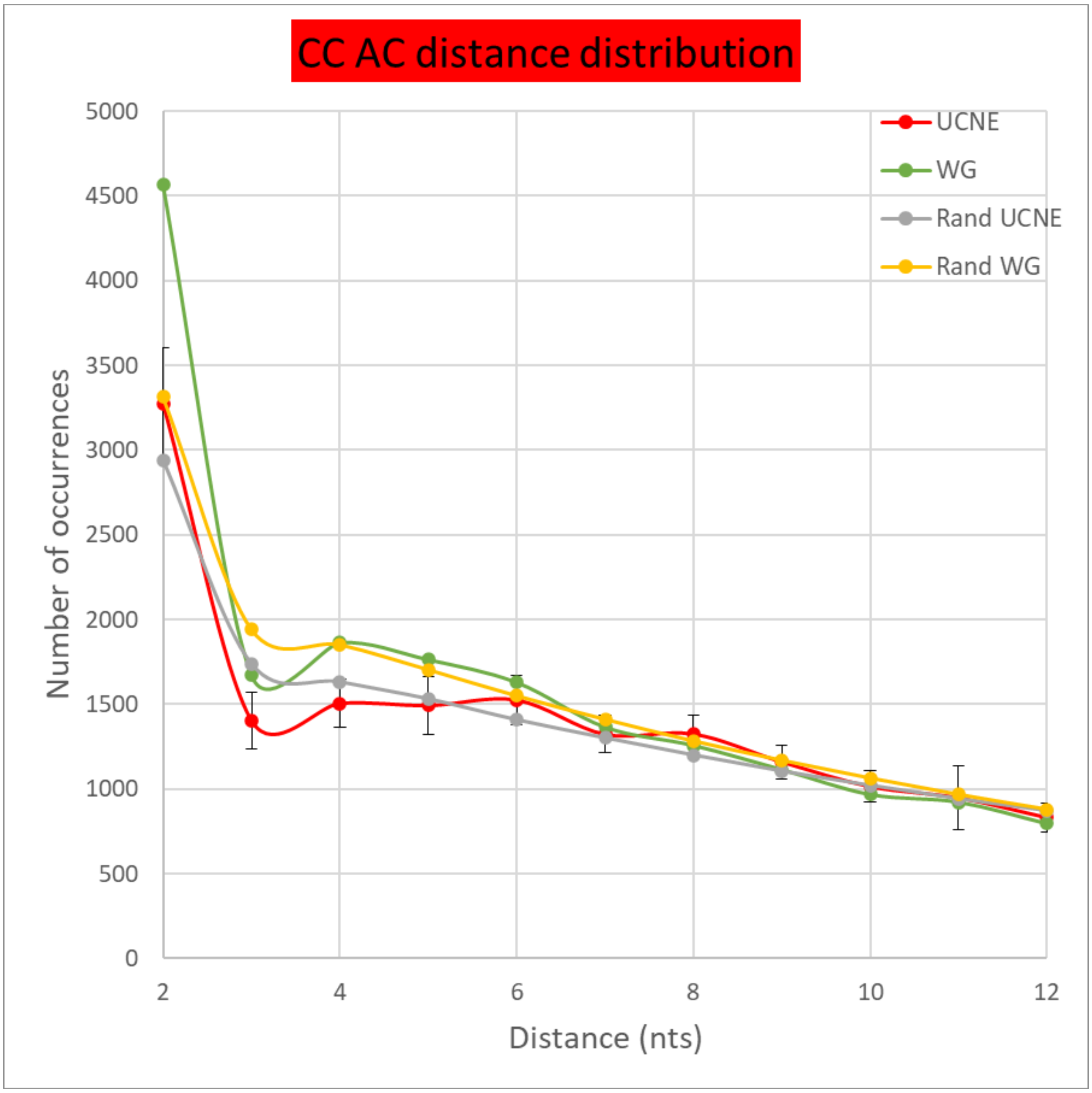

CC AC distance distribution
UCNE
WG
Rand UCNE
Rand WG
Number of occurrences
5000
4500
4000
3500
3000
2500
2000
1500
1000
500
0
2
4
6
8
10
12
Distance (nts)

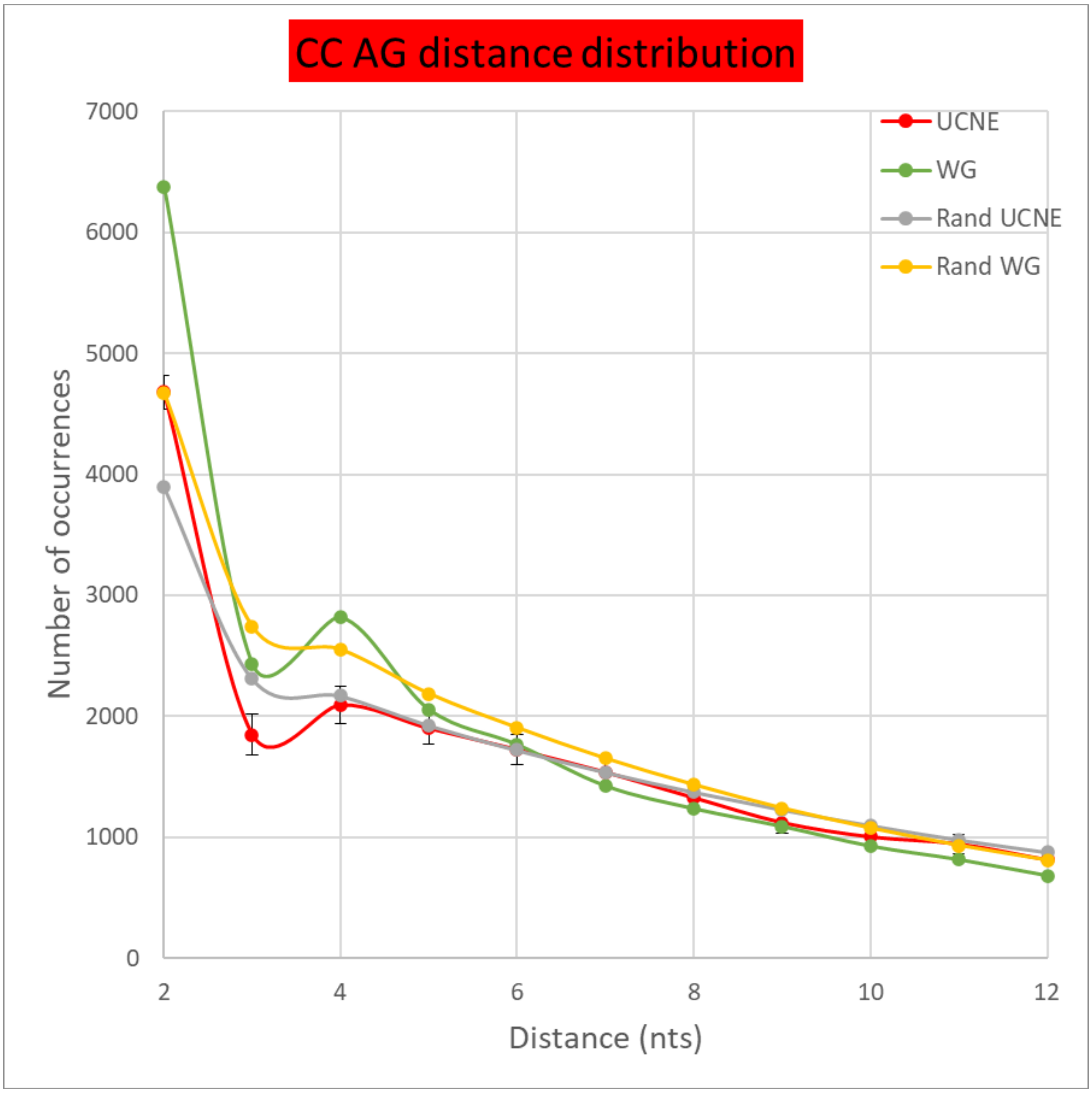

CC AG distance distribution
UCNE
WG
Rand UCNE
Rand WG
Number of occurrences
7000
6000
5000
4000
3000
2000
1000
0
2
4
6
8
10
12
Distance (nts)

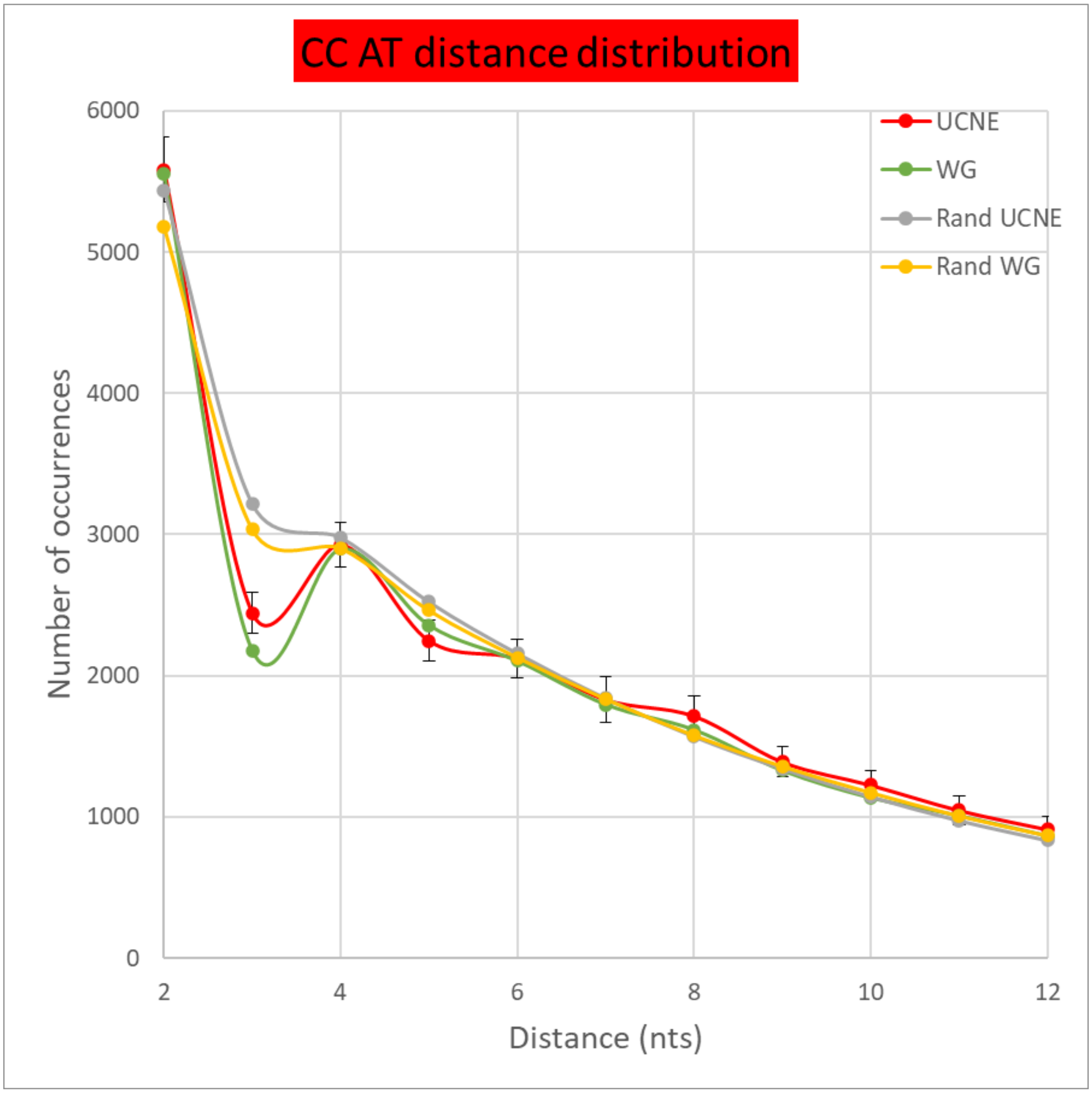
CC AT distance distribution
Number of occurrences
6000
5000
4000
3000
2000
1000
0
2
4
6
8
10
12
Distance (nts)
UCNE
WG
Rand UCNE
Rand WG

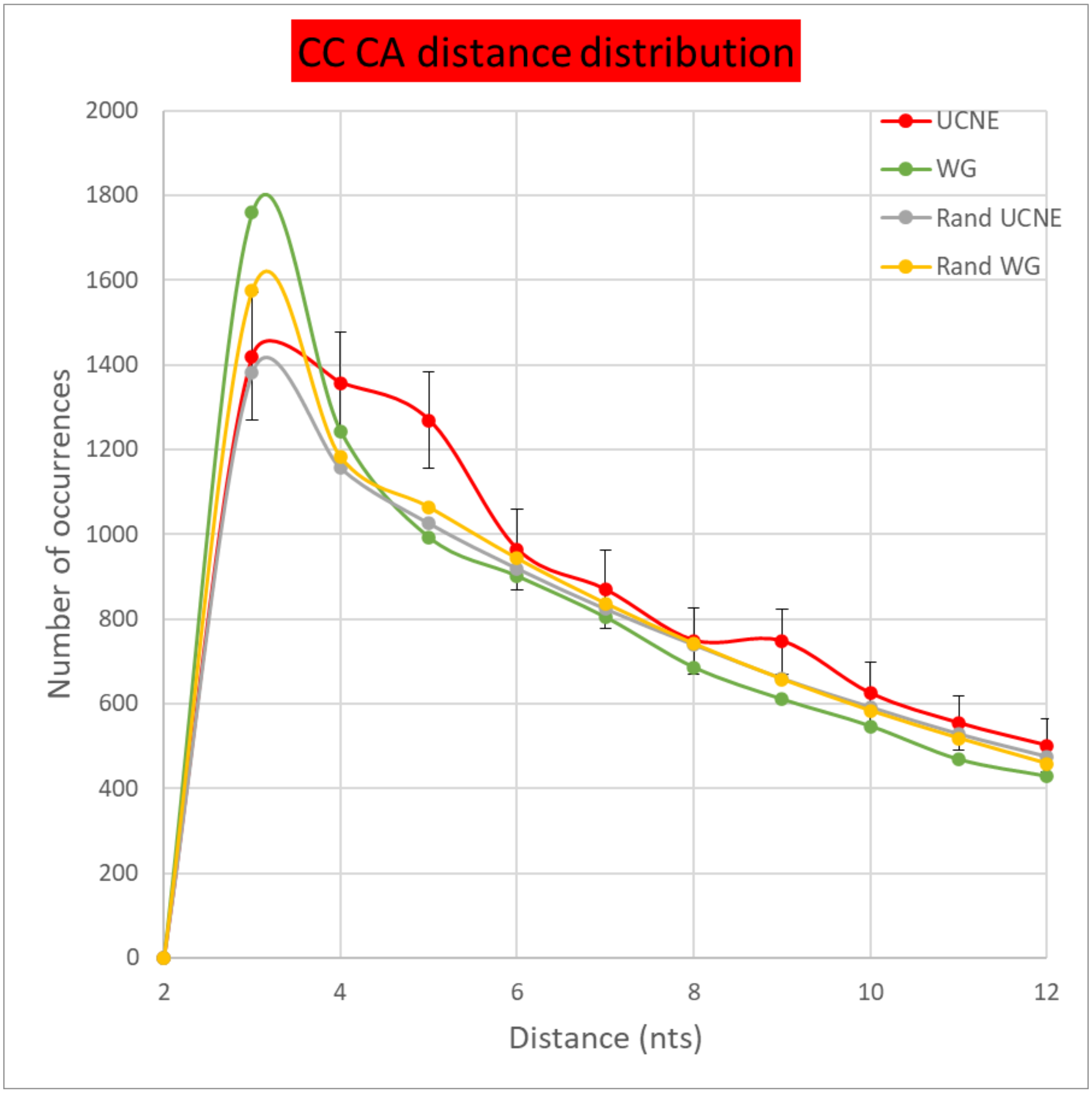
CC CA distance distribution
UCNE
WG
Rand UCNE
Rand WG
Number of occurrences
0
200
400
600
800
1000
1200
1400
1600
1800
2000
2
4
6
8
10
12
Distance (nts)

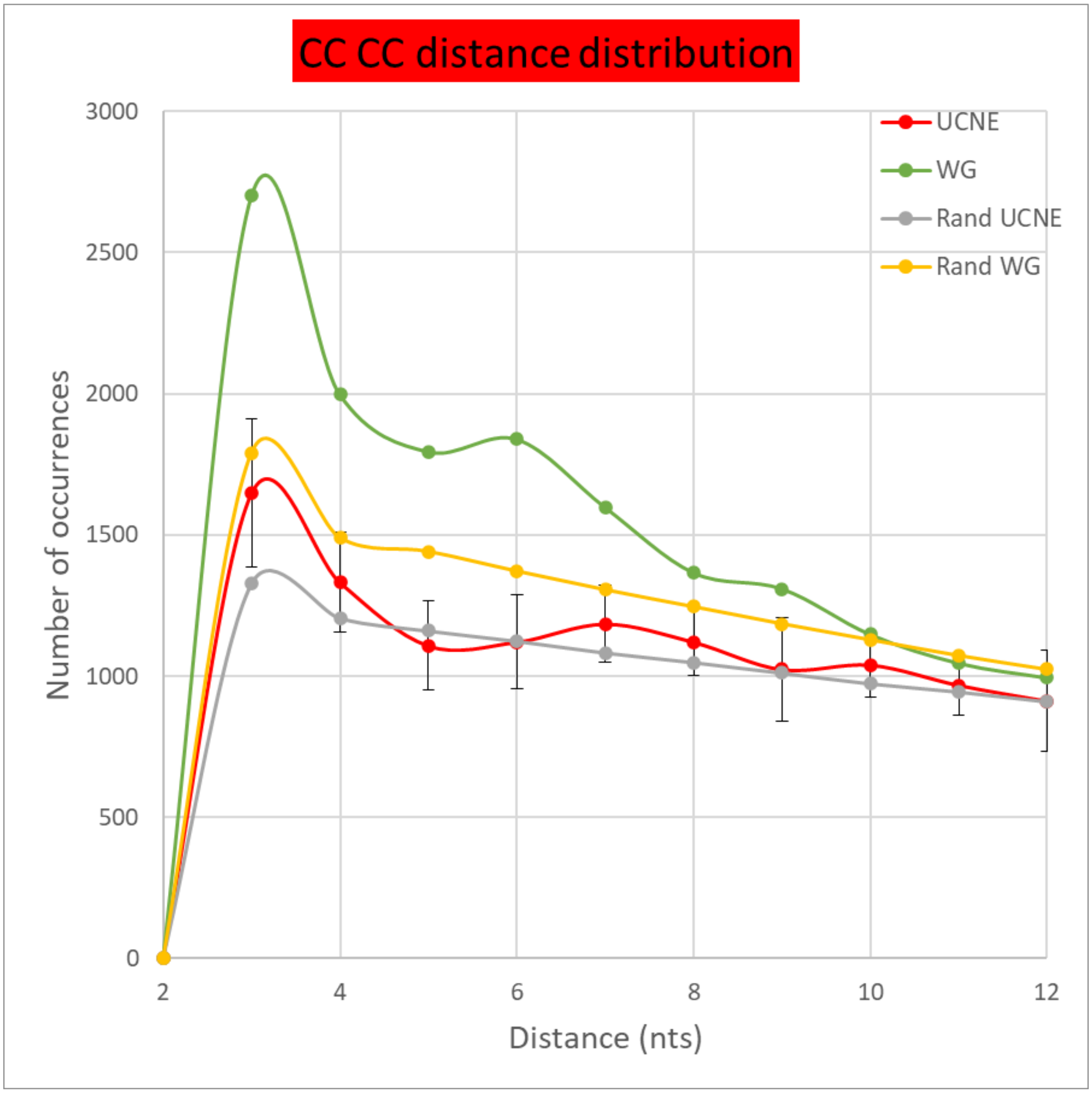
CC CC distance distribution
Number of occurrences
Distance (nts)
UCNE
WG
Rand UCNE
Rand WG
3000
2500
2000
1500
1000
500
0
2
4
6
8
10
12

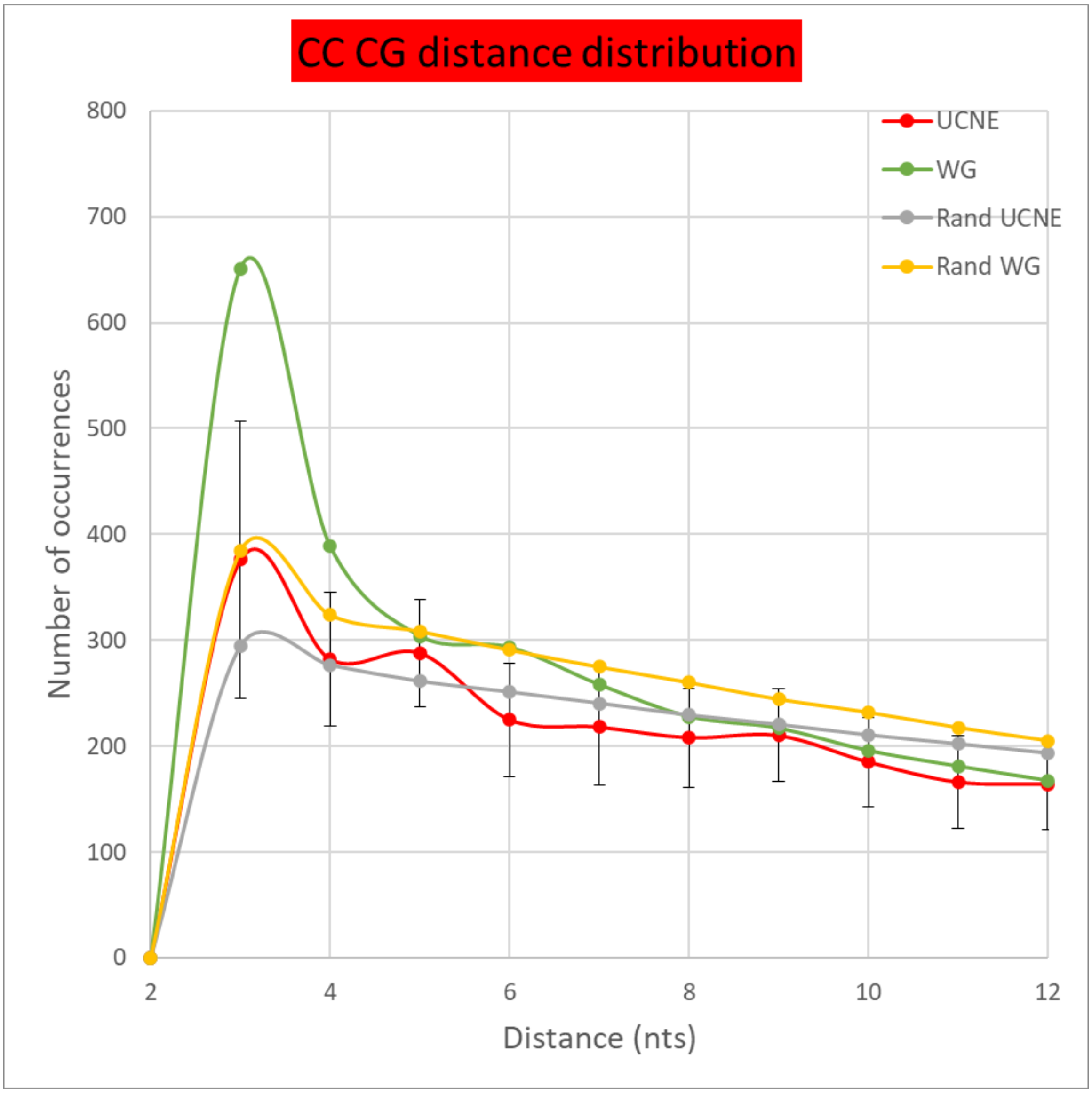
CC CG distance distribution
Number of occurrences
Distance (nts)
UCNE
WG
Rand UCNE
Rand WG
0
100
200
300
400
500
600
700
800
2
4
6
8
10
12

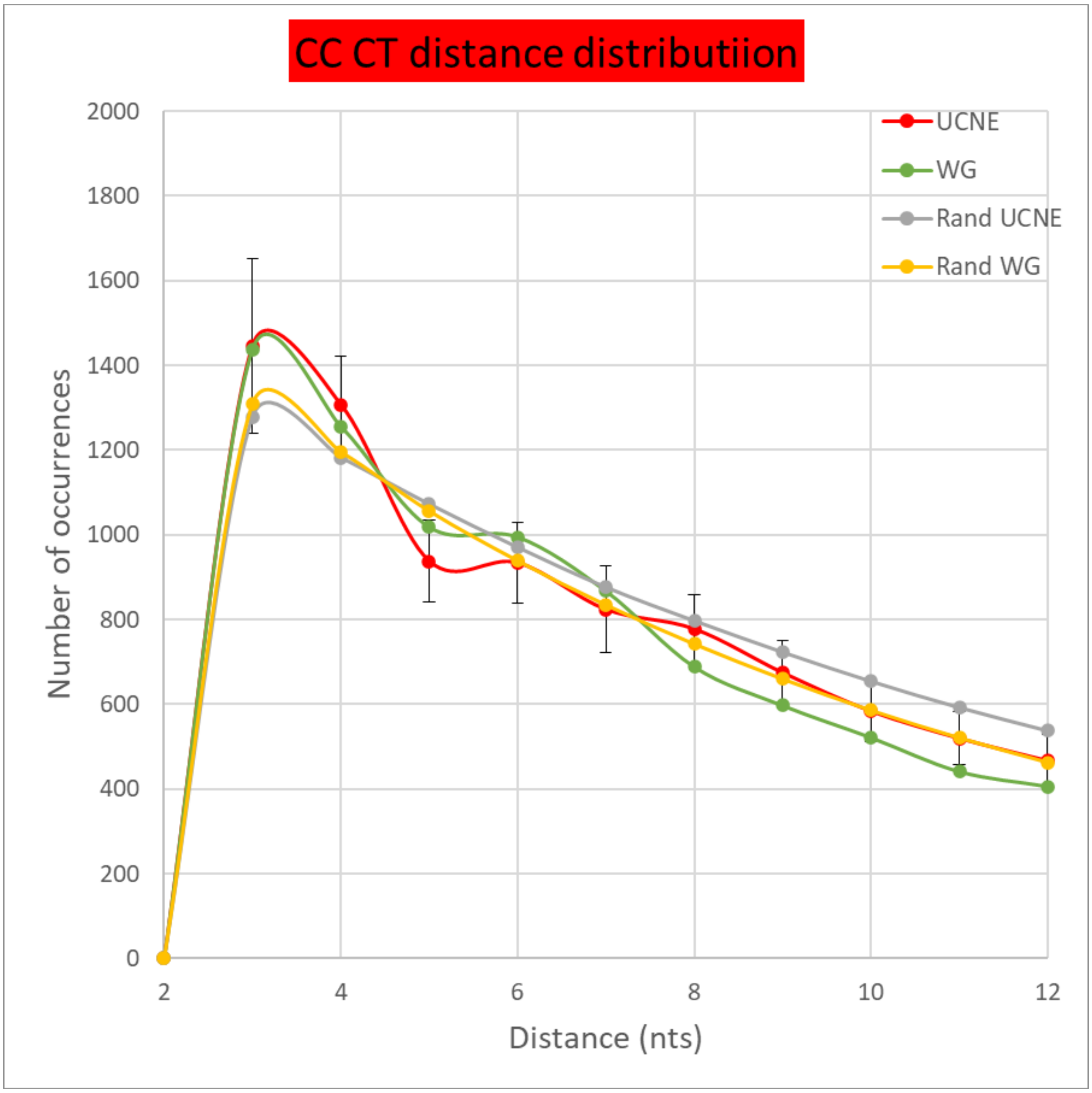

CC CT distance distributiion
2000
1800
1600
1400
1200
1000
800
600
400
200
0
Number of occurrences
2
4
6
8
10
12
Distance (nts)
UCNE
WG
Rand UCNE
Rand WG

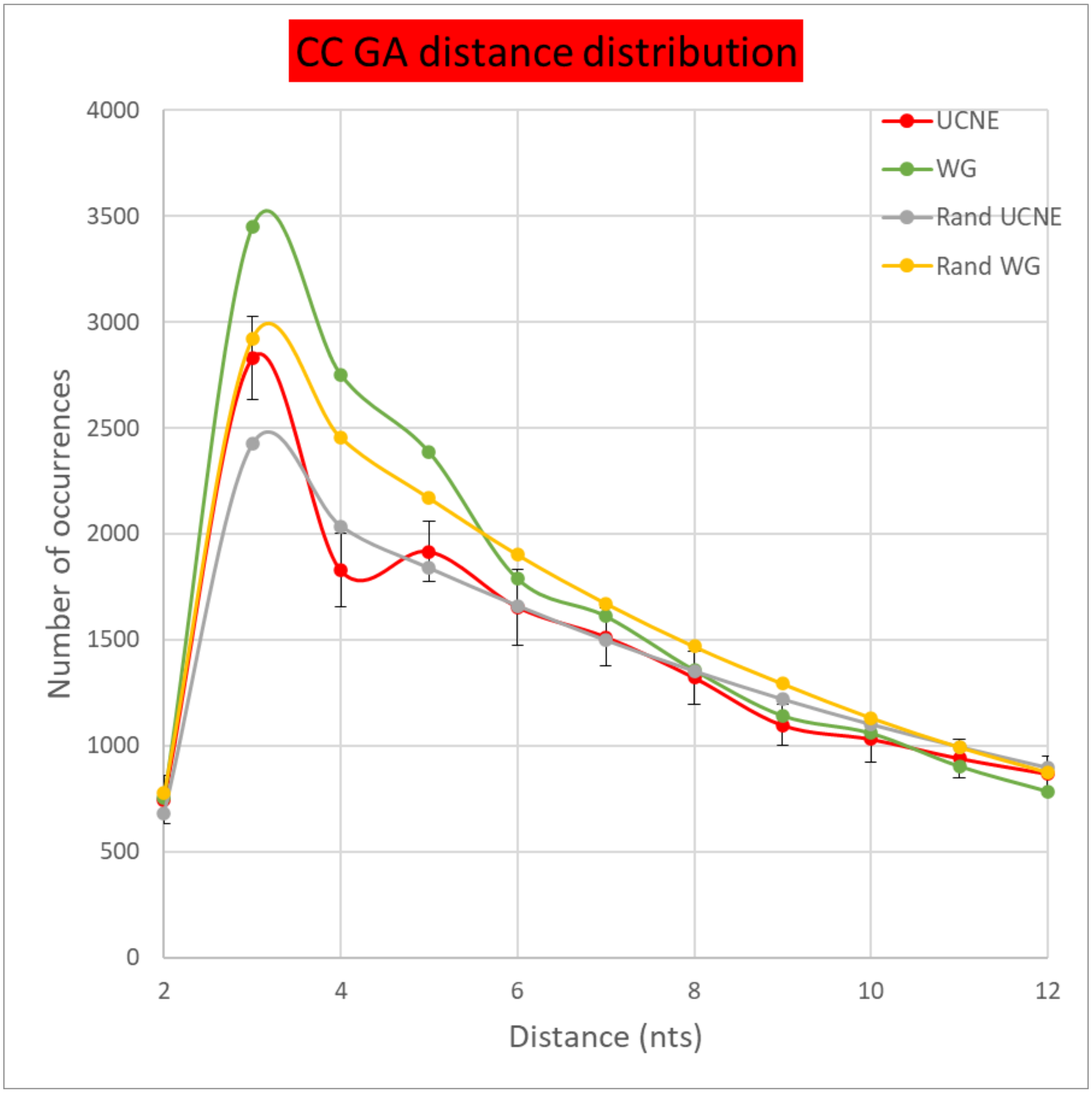

CC GA distance distribution
Number of occurrences
Distance (nts)
UCNE
WG
Rand UCNE
Rand WG
0
500
1000
1500
2000
2500
3000
3500
4000
2
4
6
8
10
12

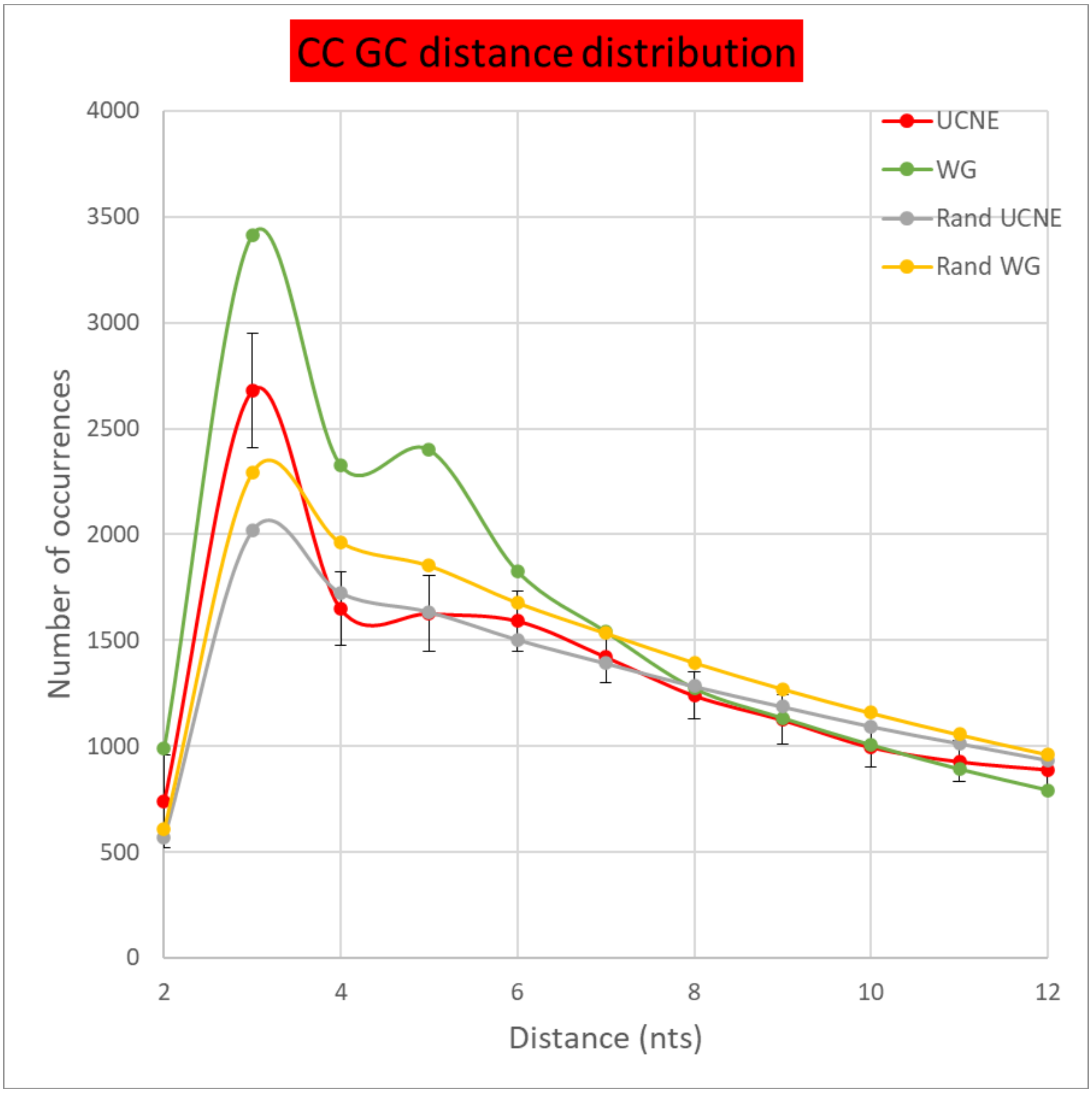

CC GC distance distribution
UCNE
WG
Rand UCNE
Rand WG
Number of occurrences
4000
3500
3000
2500
2000
1500
1000
500
0
2
4
6
8
10
12
Distance (nts)

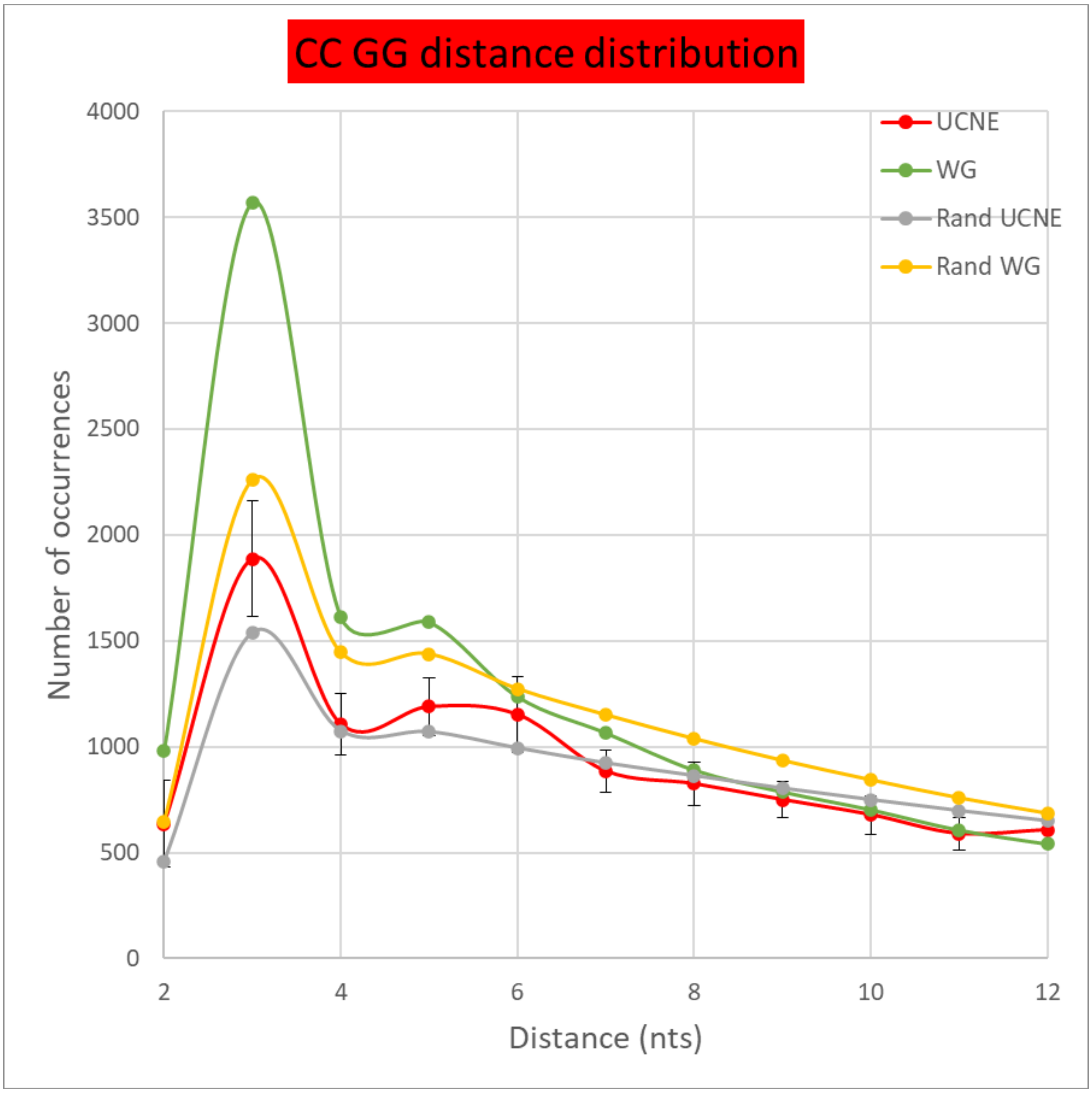

CC GG distance distribution
UCNE
WG
Rand UCNE
Rand WG
Number of occurrences
0
500
1000
1500
2000
2500
3000
3500
4000
2
4
6
8
10
12
Distance (nts)

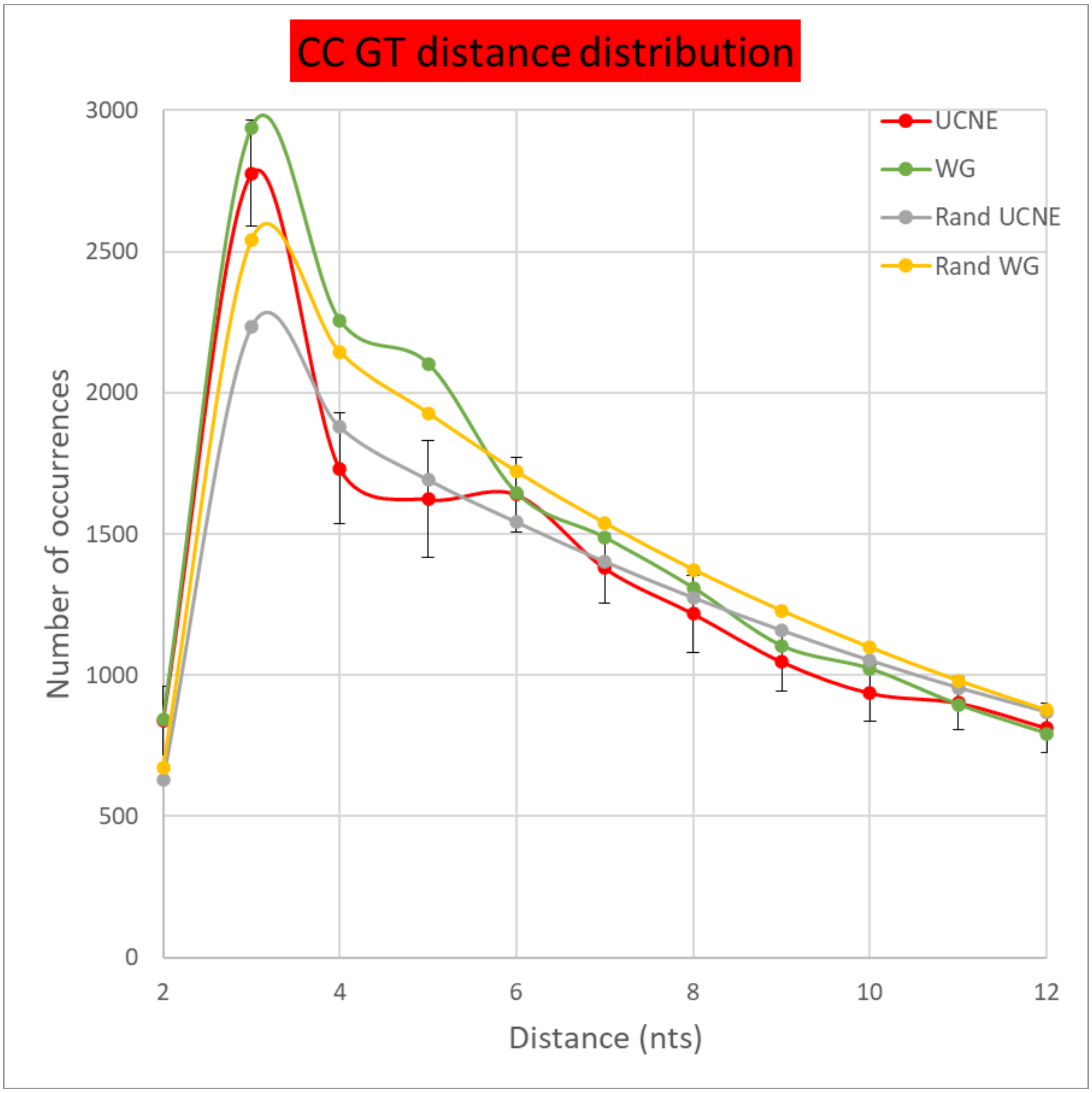
CC GT distance distribution
UCNE
WG
Rand UCNE
Rand WG
Number of occurrences
3000
2500
2000
1500
1000
500
0
2
4
6
8
10
12
Distance (nts)

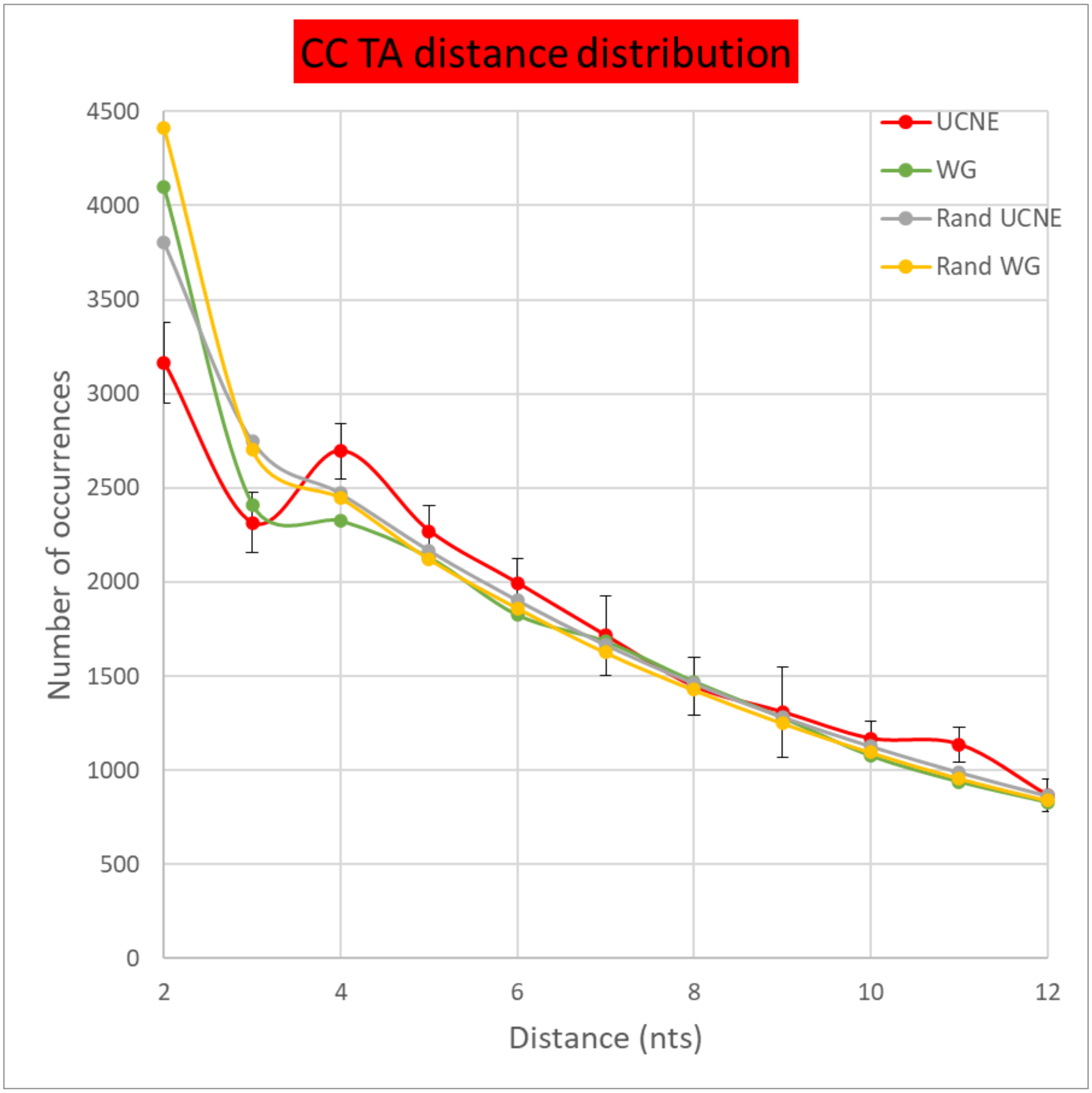
CC TA distance distribution
4500
4000
3500
3000
2500
2000
1500
1000
500
0
2
4
6
8
10
12
Number of occurrences
Distance (nts)
UCNE
WG
Rand UCNE
Rand WG

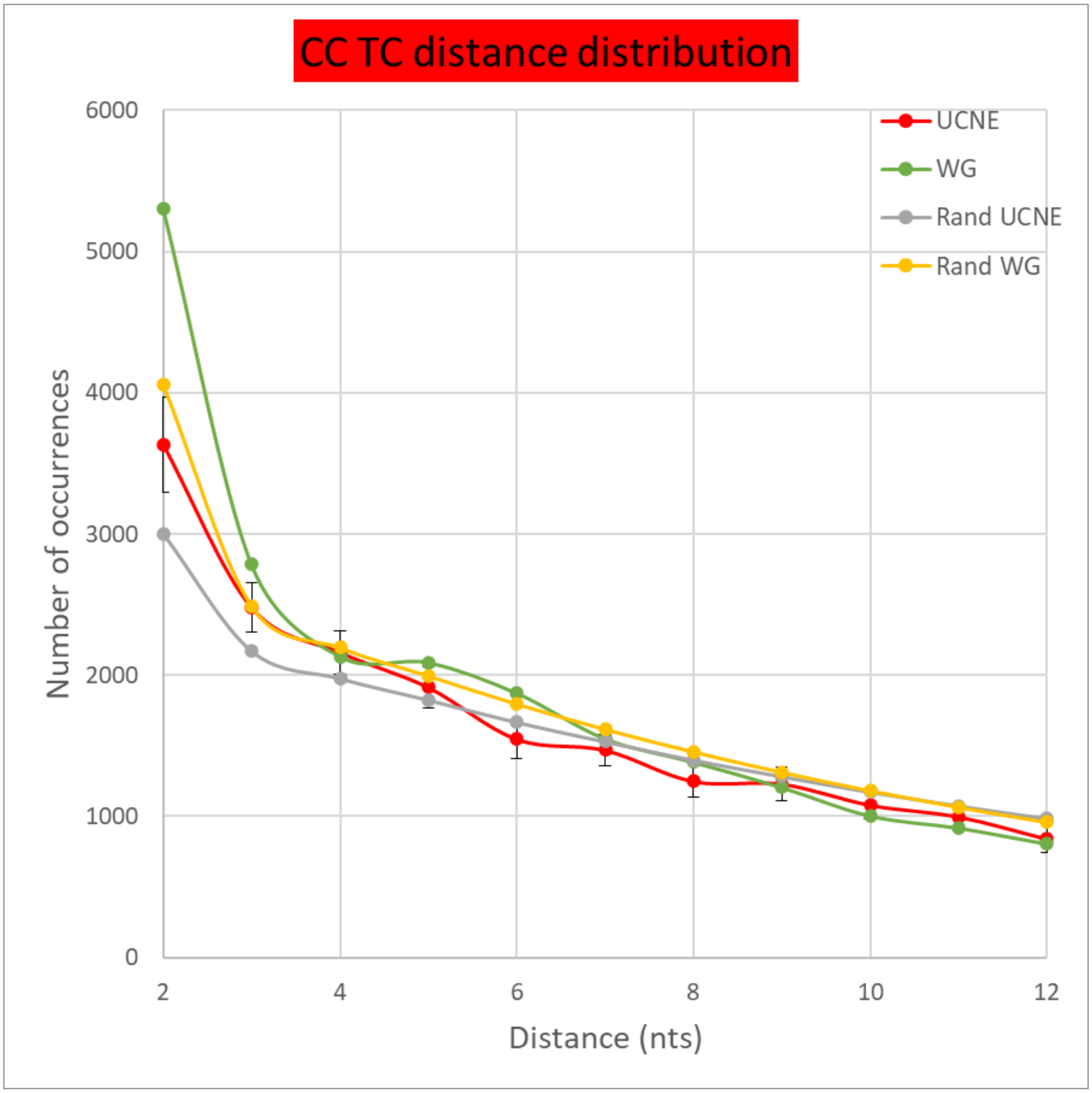

CC TC distance distribution
6000
5000
4000
3000
2000
1000
0
2
4
6
8
10
12
Number of occurrences
Distance (nts)
UCNE
WG
Rand UCNE
Rand WG

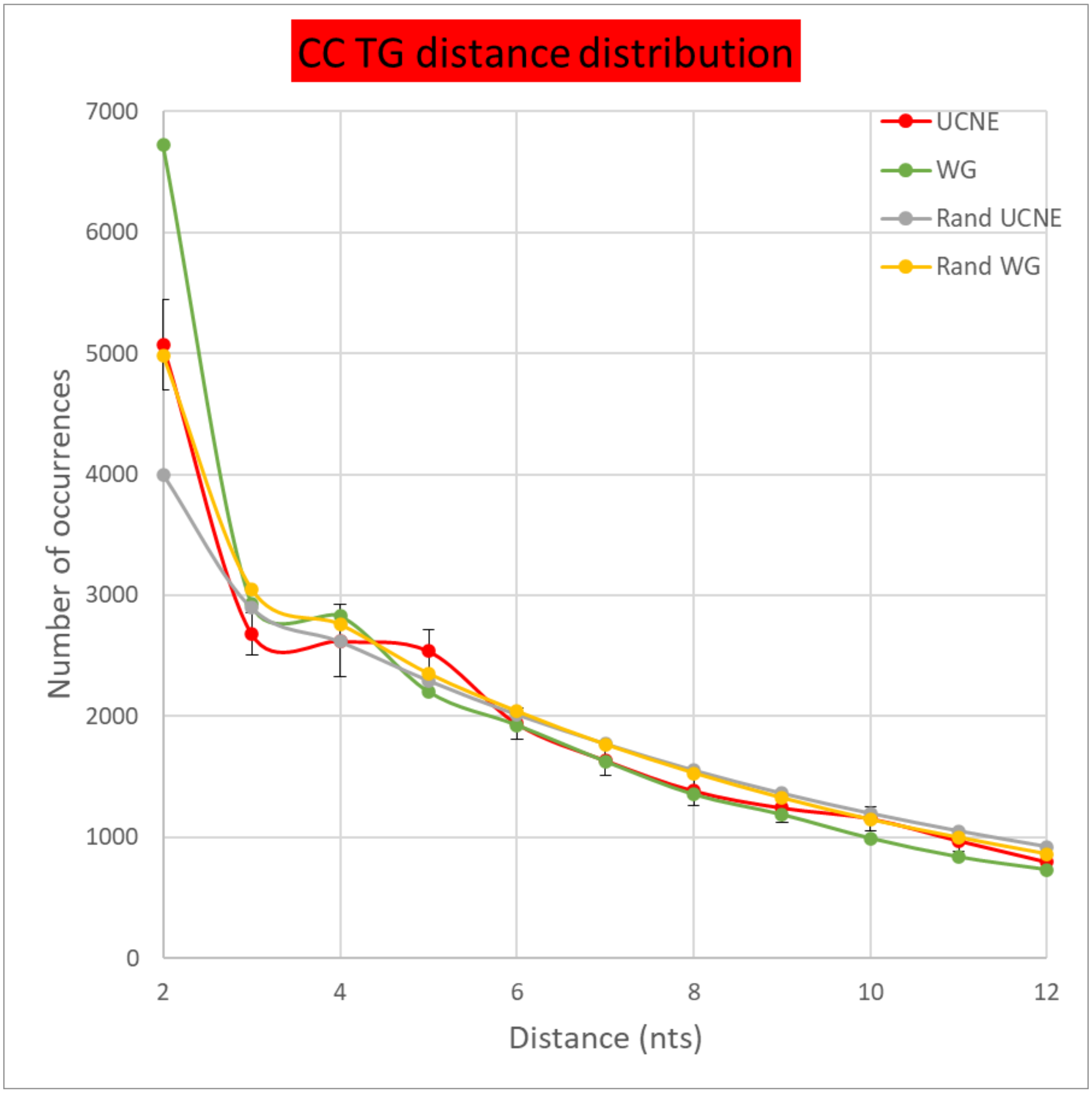

CC TG distance distribution
UCNE
WG
Rand UCNE
Rand WG
Number of occurrences
Distance (nts)
7000
6000
5000
4000
3000
2000
1000
0
2
4
6
8
10
12

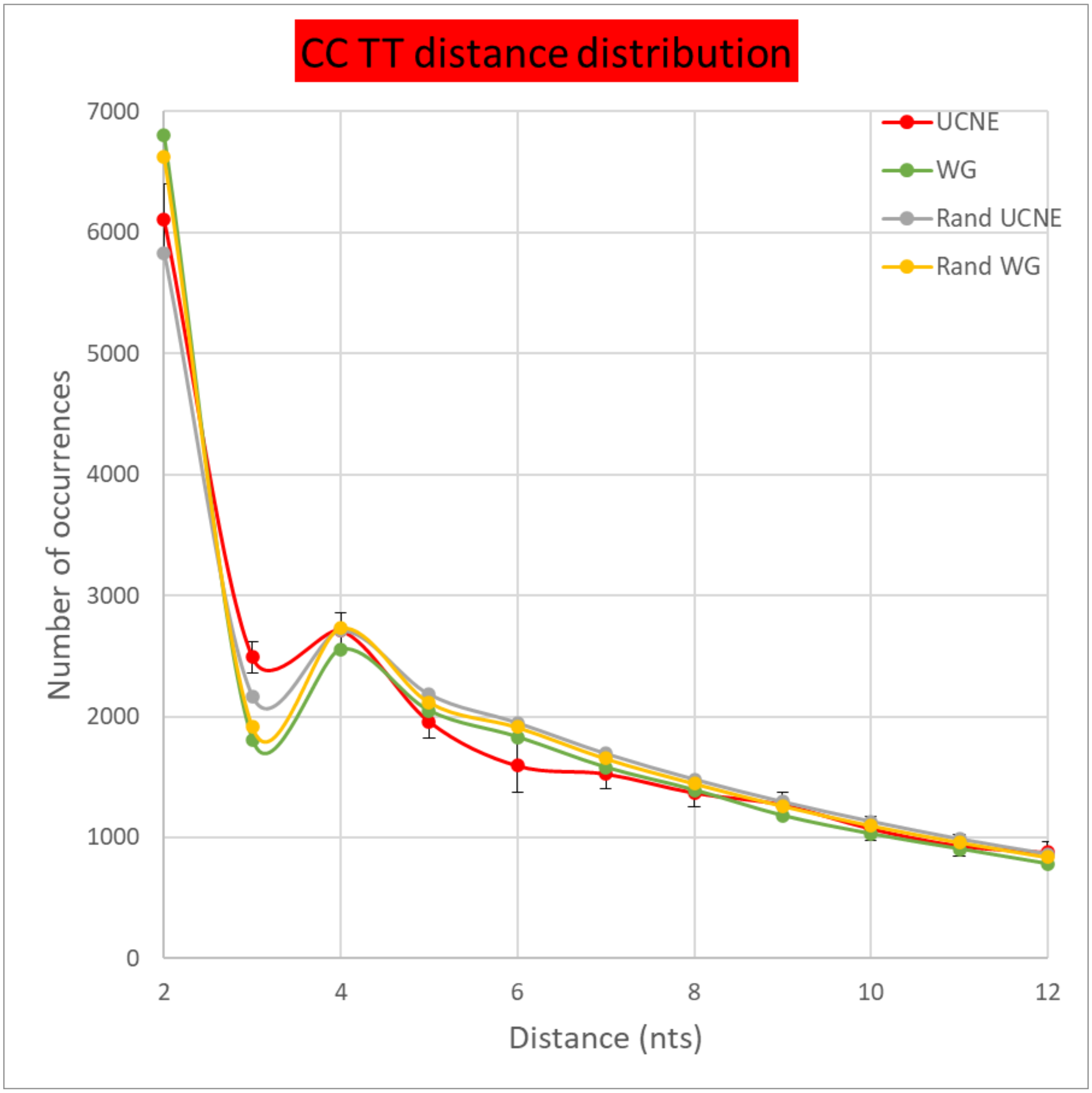

CC TT distance distribution
7000
6000
5000
4000
3000
2000
1000
0
Number of occurrences
2
4
6
8
10
12
Distance (nts)
UCNE
WG
Rand UCNE
Rand WG

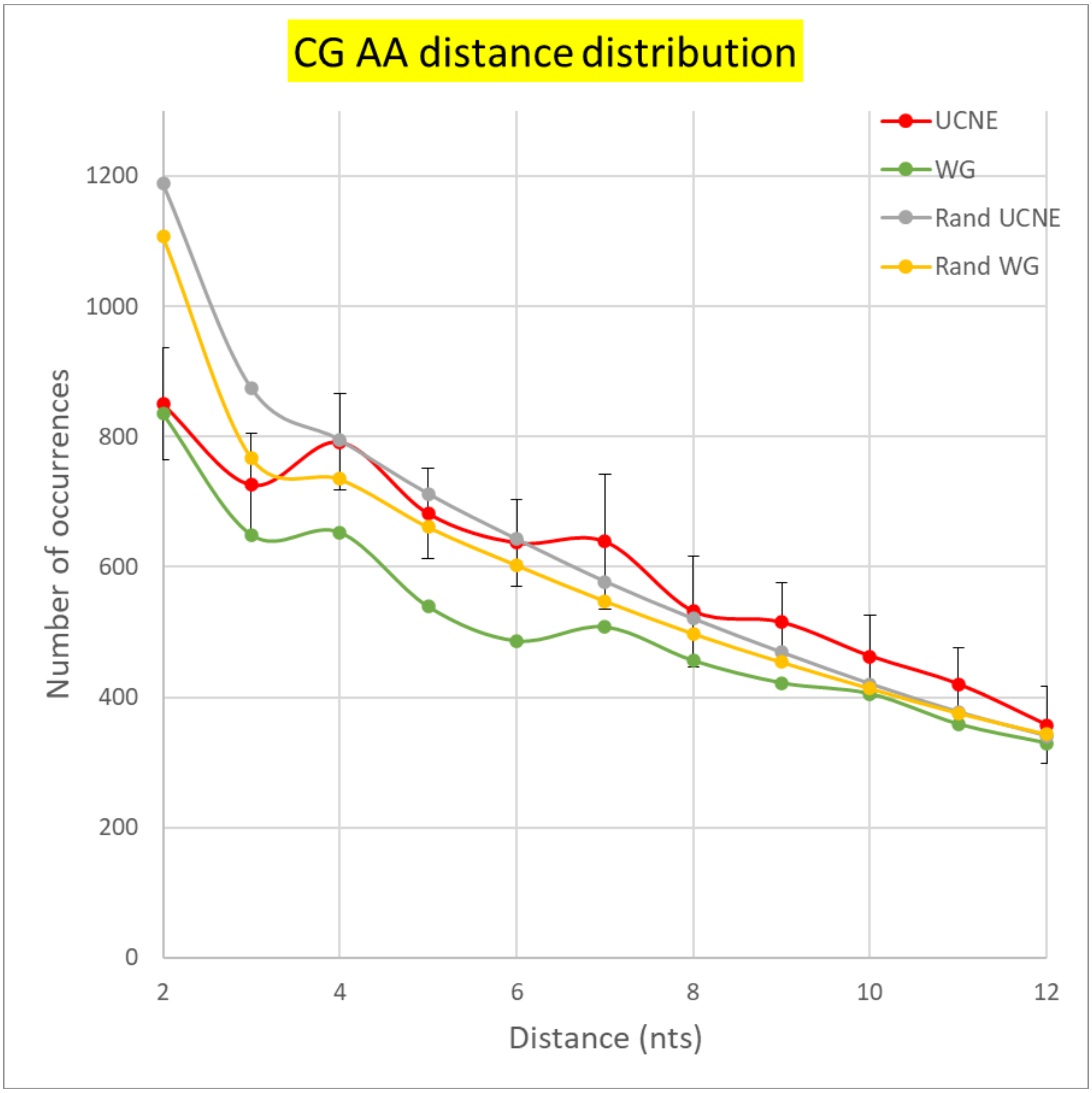

CG AA distance distribution
UCNE
WG
Rand UCNE
Rand WG
Number of occurrences
0
200
400
600
800
1000
1200
2
4
6
8
10
12
Distance (nts)

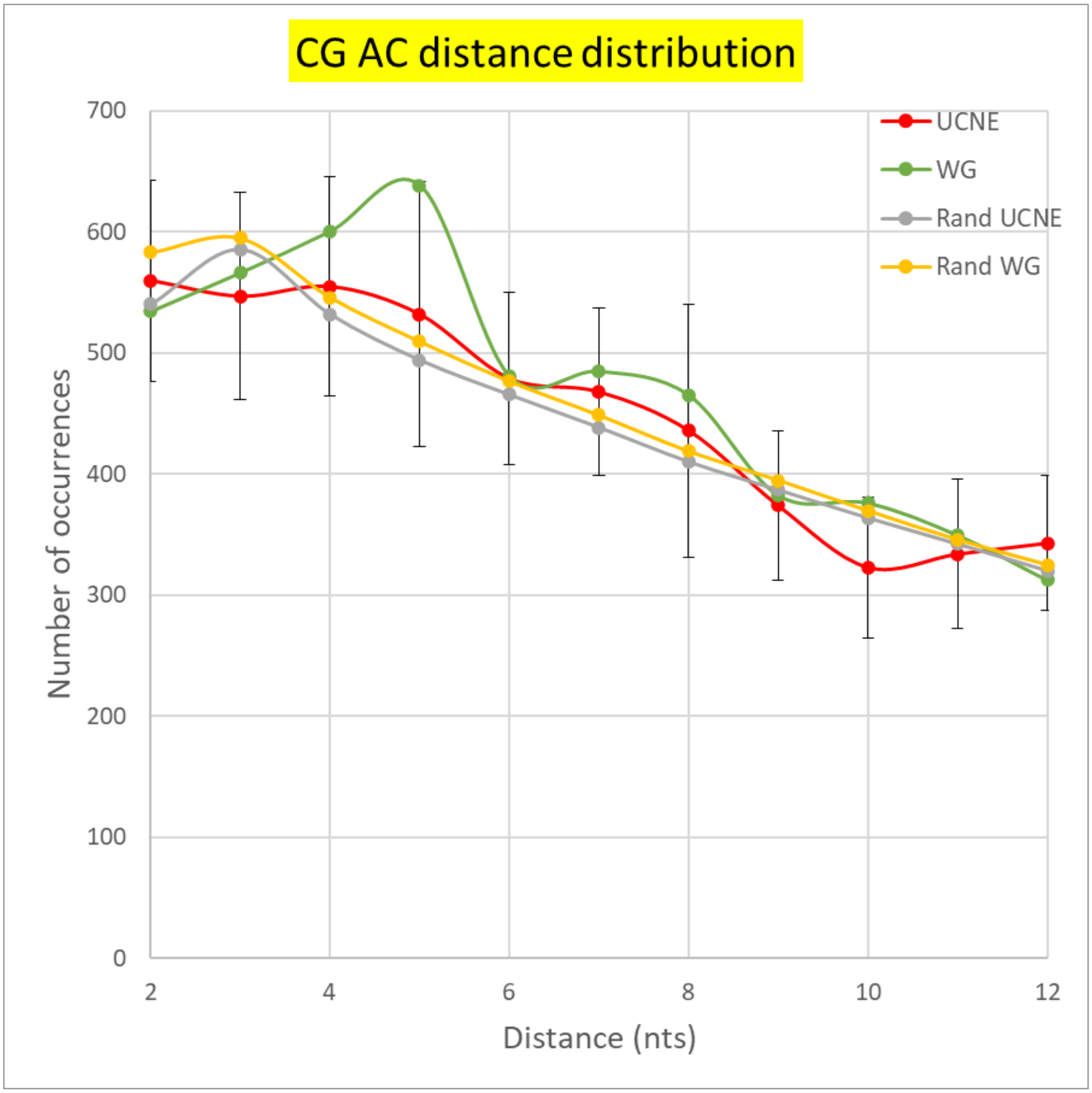
CG AC distance distribution
UCNE
WG
Rand UCNE
Rand WG
Number of occurrences
0
100
200
300
400
500
600
700
2
4
6
8
10
12
Distance (nts)

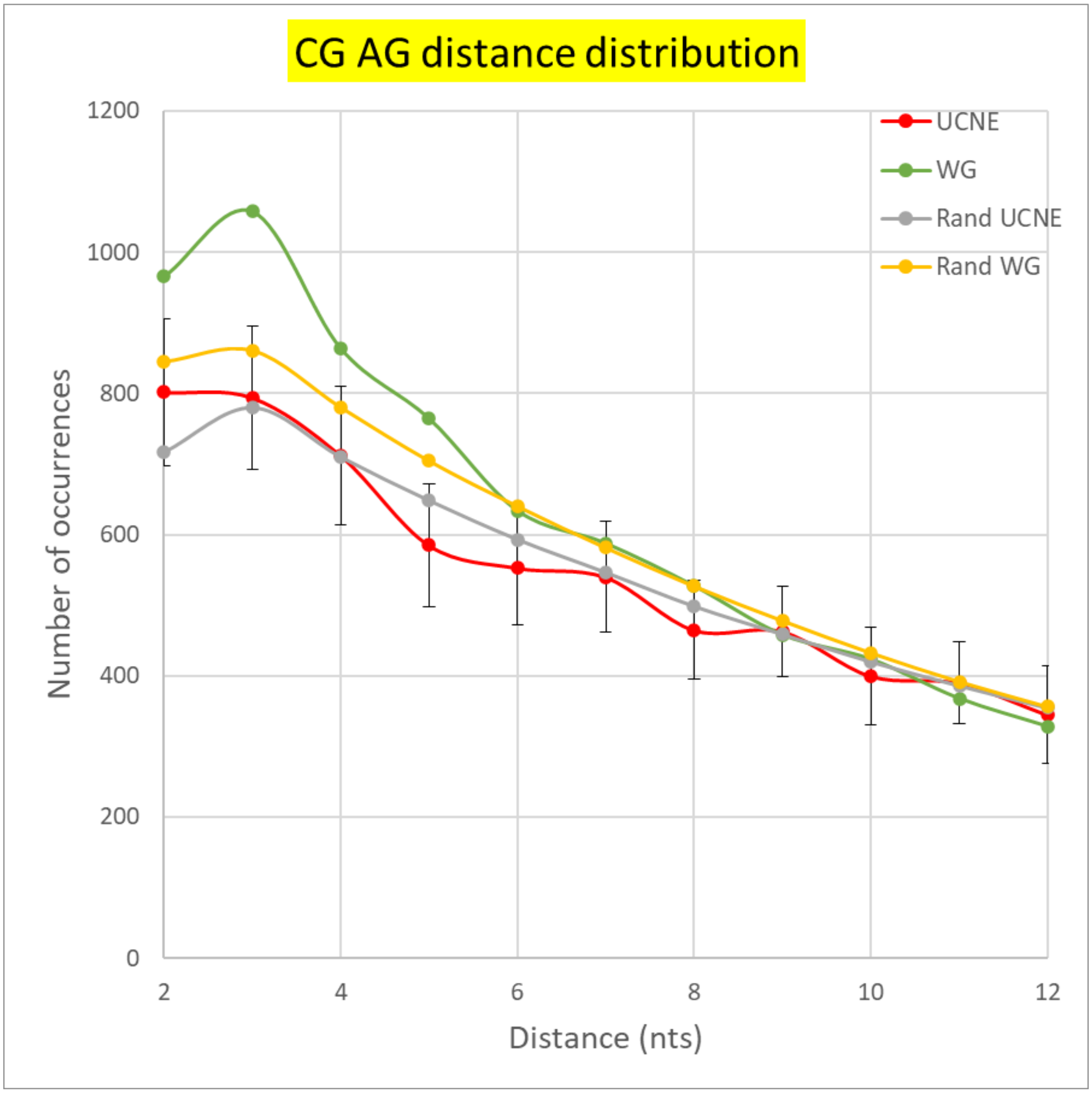
CG AG distance distribution
UCNE
WG
Rand UCNE
Rand WG
Number of occurrences
Distance (nts)
0
200
400
600
800
1000
1200
2
4
6
8
10
12

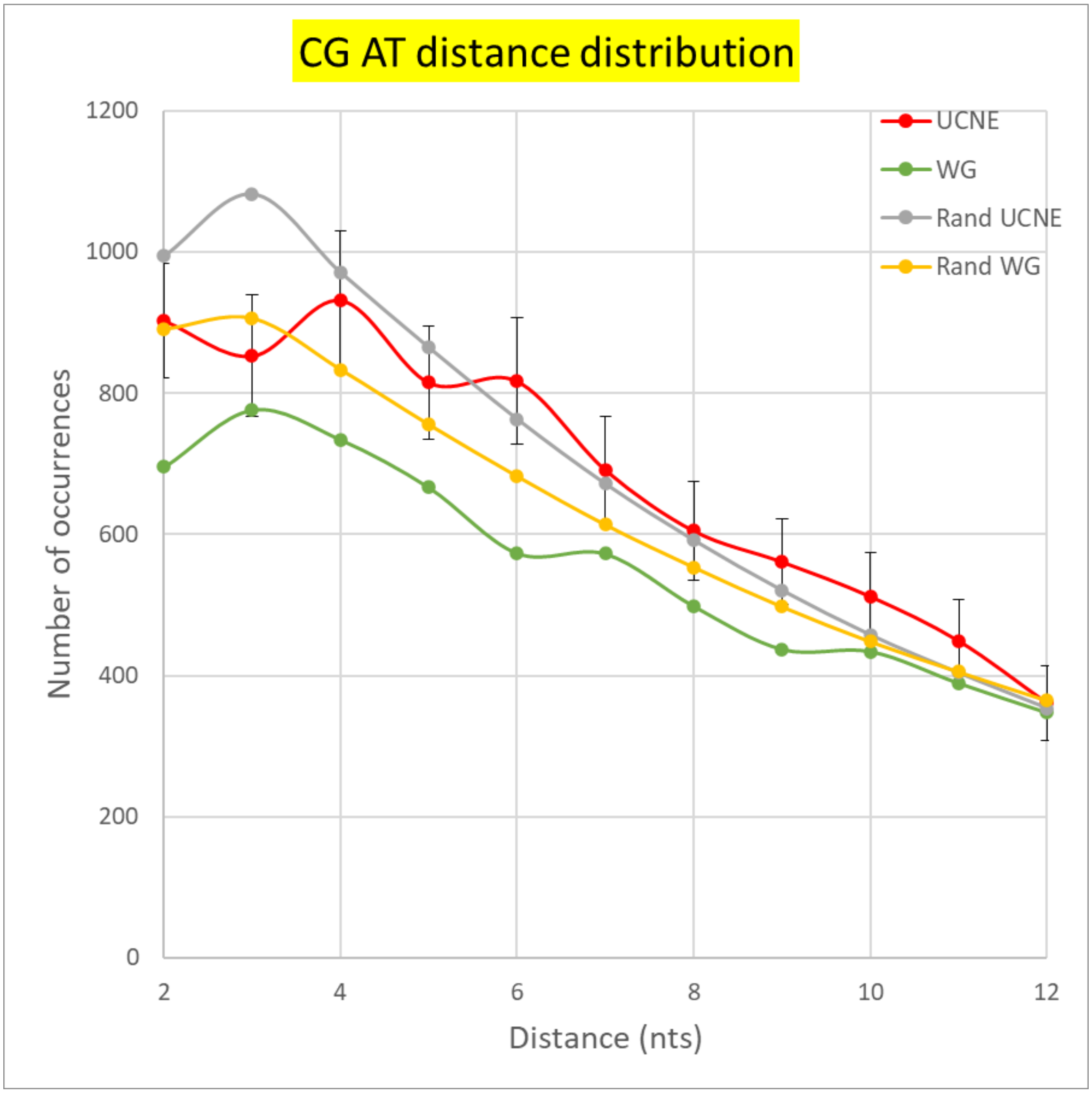
CG AT distance distribution
Number of occurrences
Distance (nts)
1200
1000
800
600
400
200
0
2
4
6
8
10
12
UCNE
WG
Rand UCNE
Rand WG

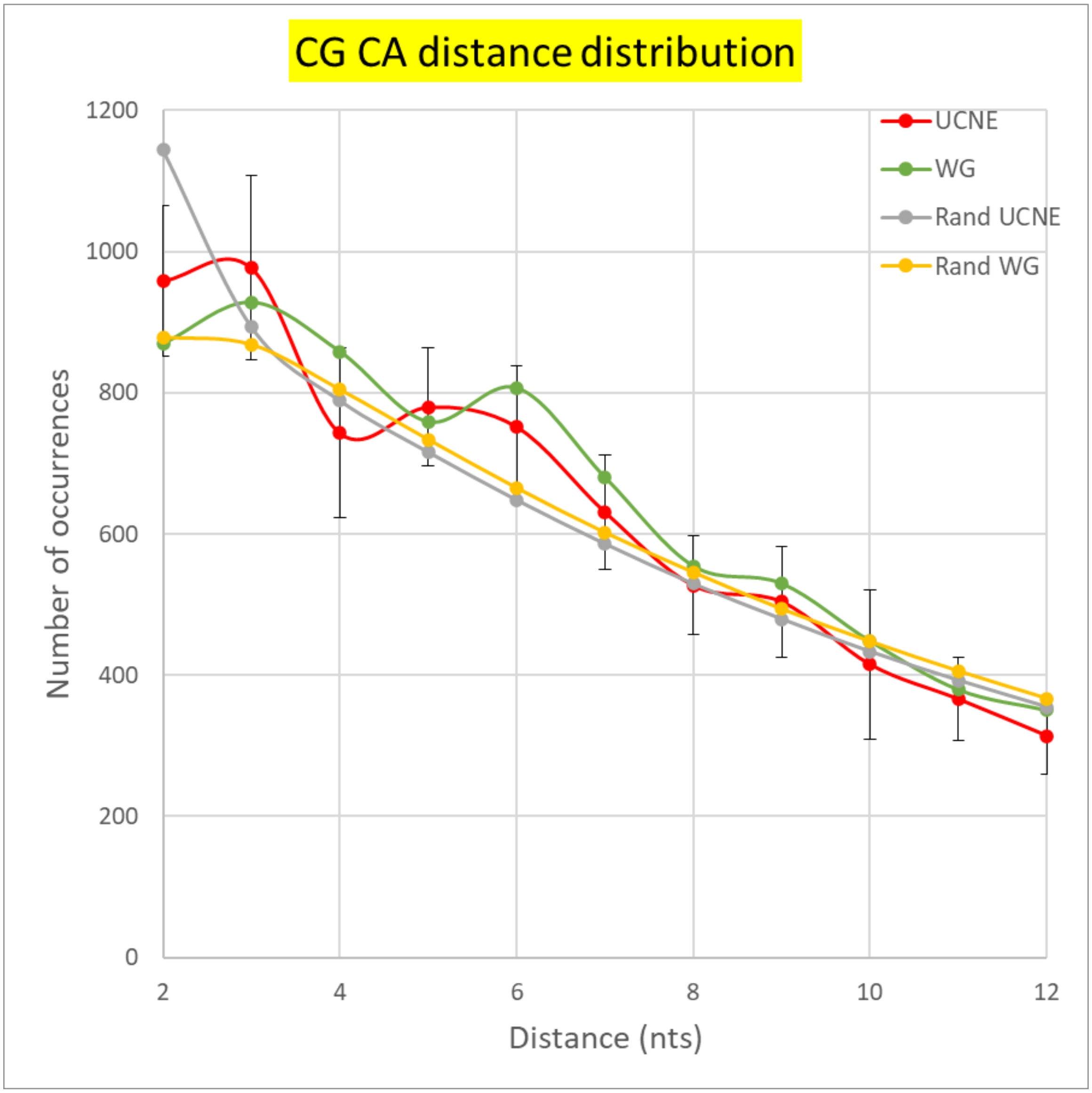

CG CA distance distribution
UCNE
WG
Rand UCNE
Rand WG
Number of occurrences
0
200
400
600
800
1000
1200
2
4
6
8
10
12
Distance (nts)

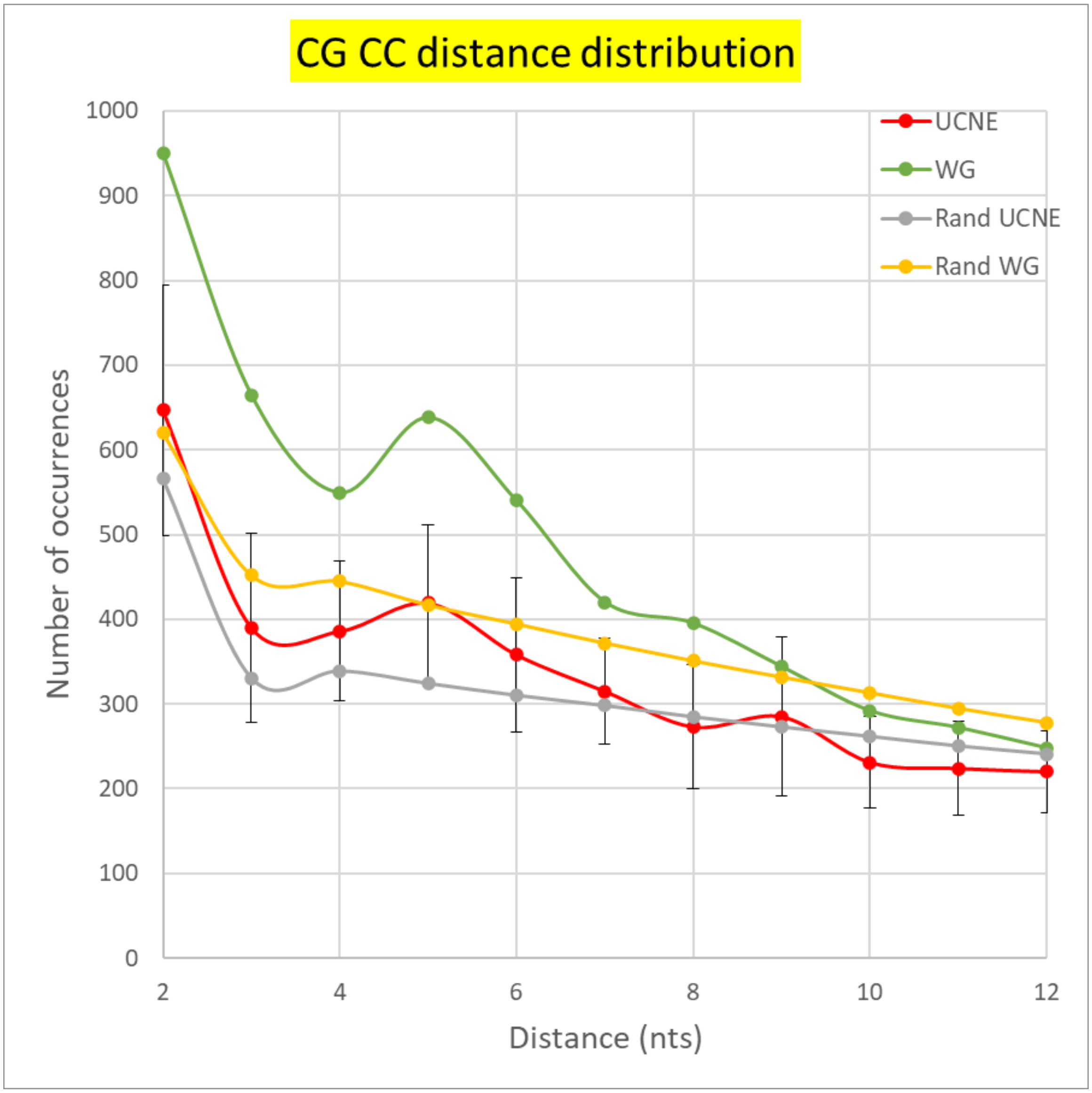
CG CC distance distribution
Number of occurrences
1000
900
800
700
600
500
400
300
200
100
0
2
4
6
8
10
12
Distance (nts)
UCNE
WG
Rand UCNE
Rand WG

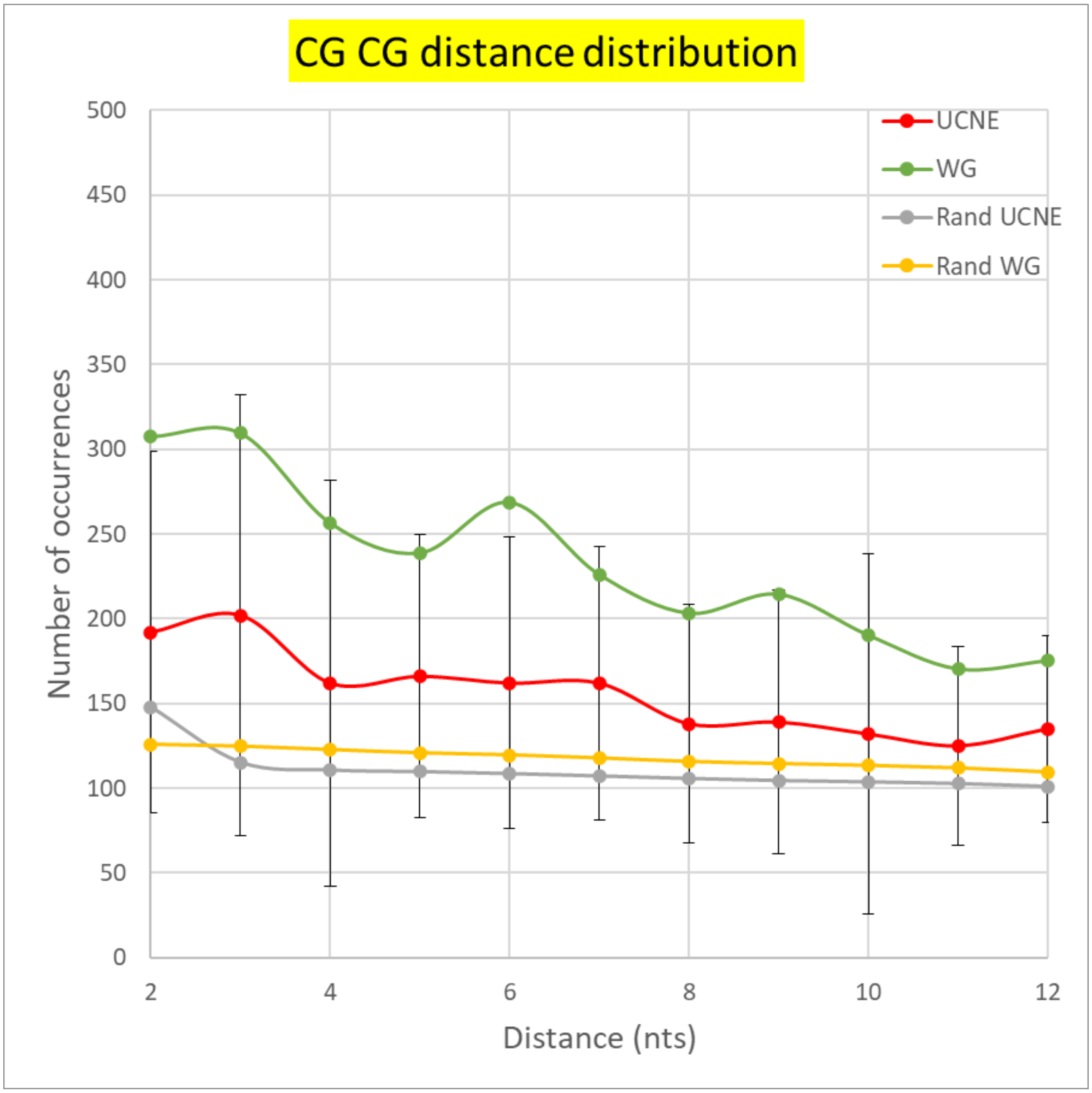

CG CG distance distribution
Number of occurrences
Distance (nts)
UCNE
WG
Rand UCNE
Rand WG
0
50
100
150
200
250
300
350
400
450
500
2
4
6
8
10
12

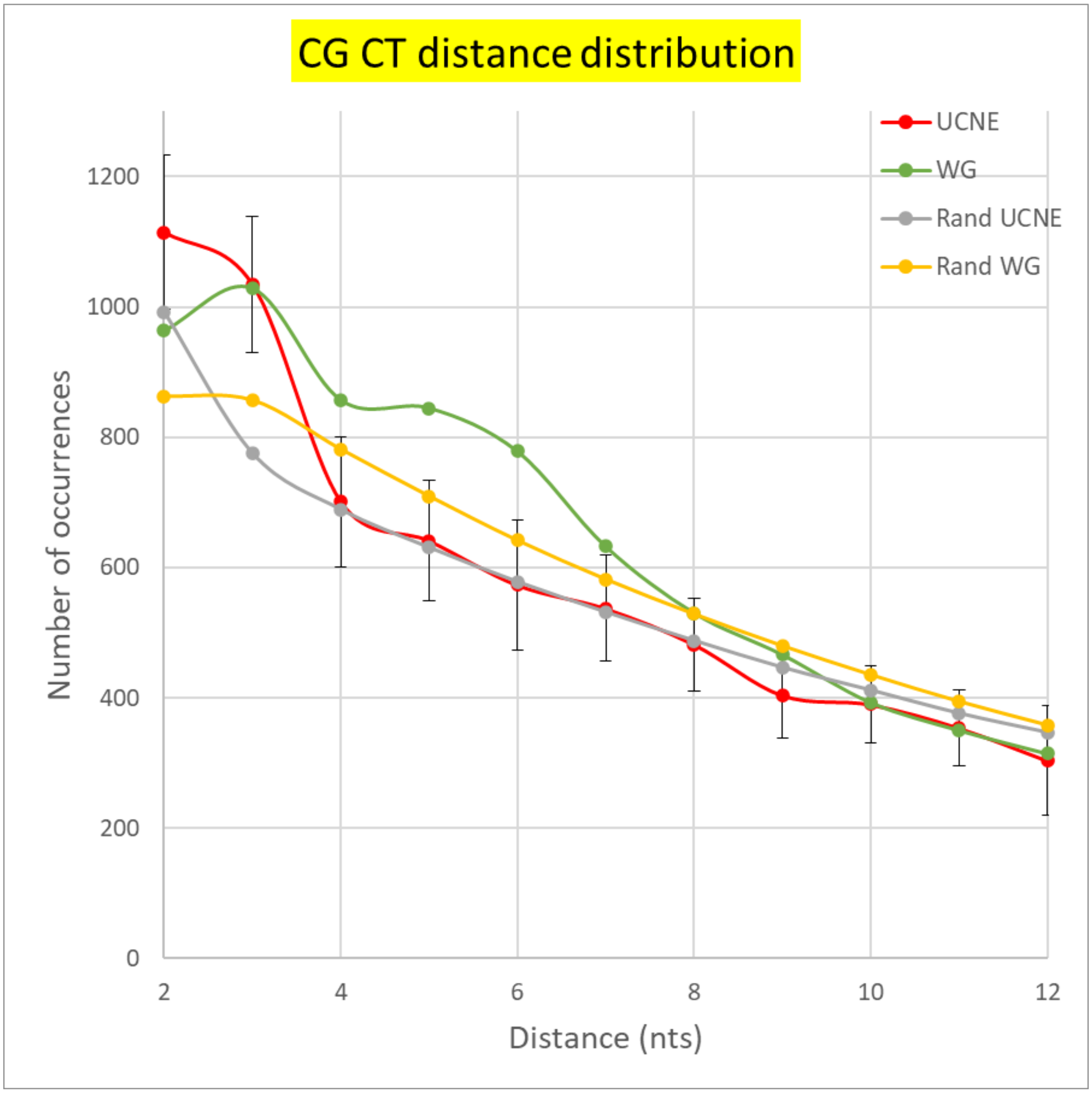
CG CT distance distribution
UCNE
WG
Rand UCNE
Rand WG
Number of occurrences
0
200
400
600
800
1000
1200
2
4
6
8
10
12
Distance (nts)

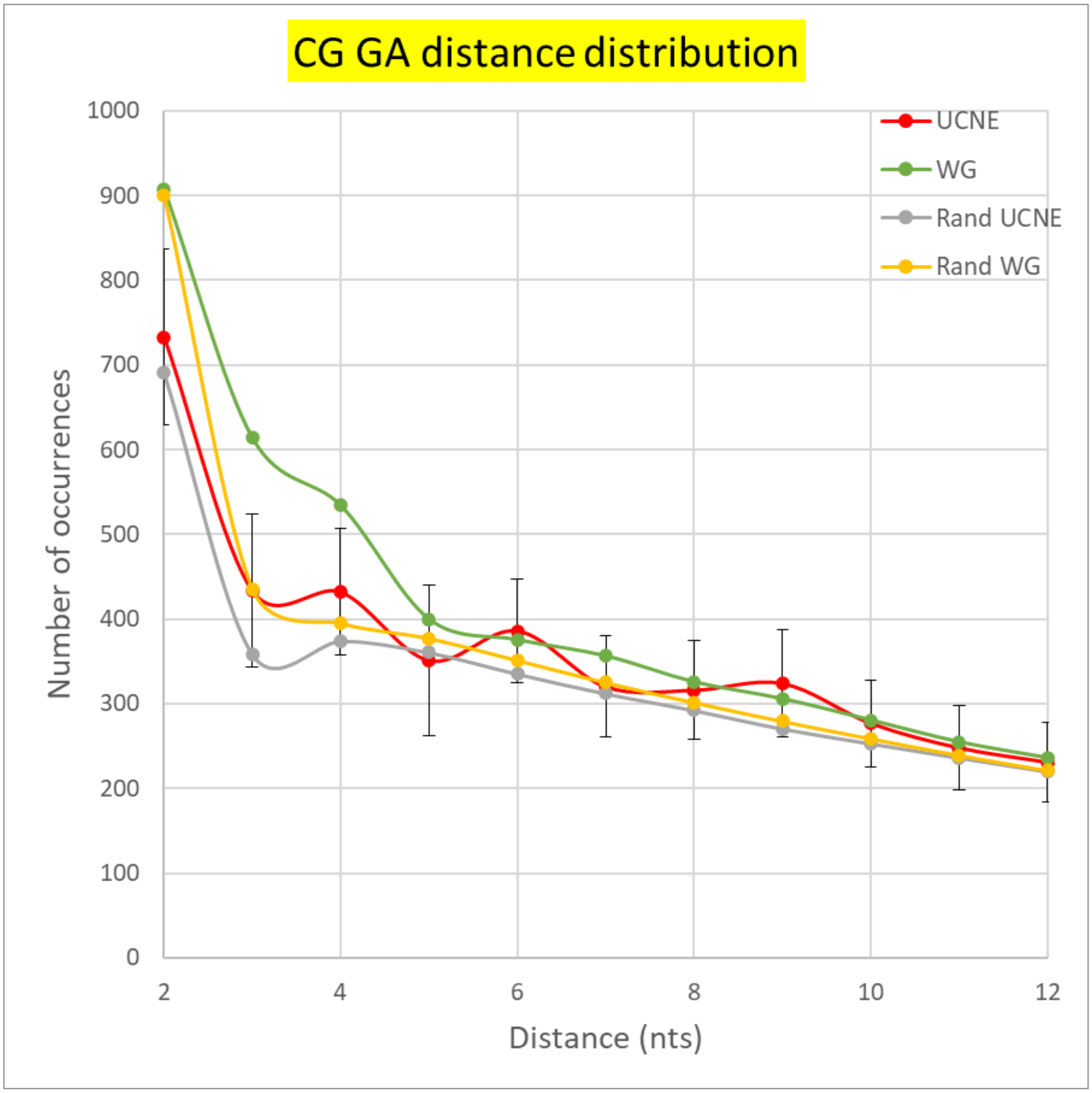
CG GA distance distribution
Number of occurrences
Distance (nts)
UCNE
WG
Rand UCNE
Rand WG
0
100
200
300
400
500
600
700
800
900
1000
2
4
6
8
10
12

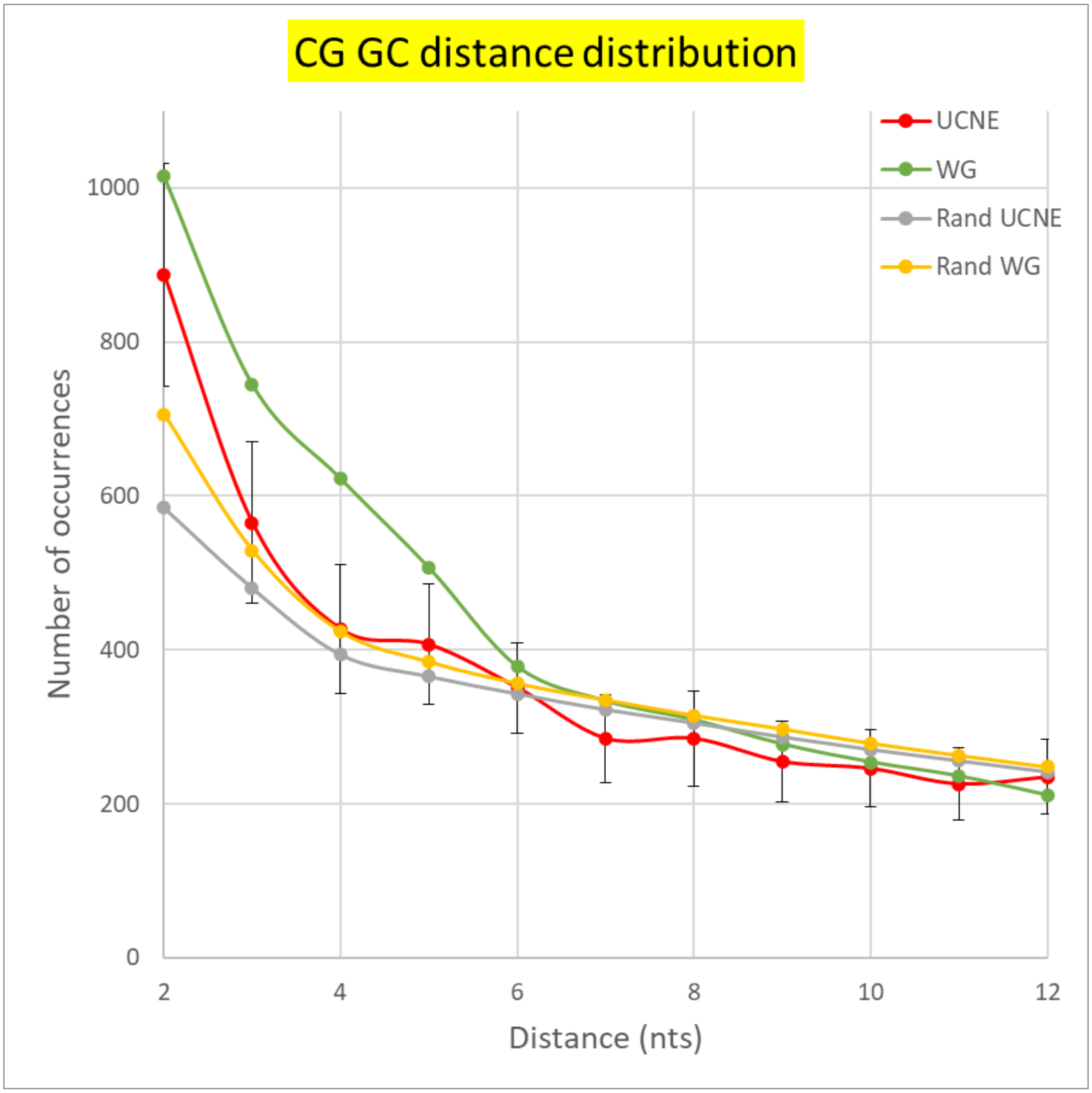

CG GC distance distribution
UCNE
WG
Rand UCNE
Rand WG
Number of occurrences
0
200
400
600
800
1000
2
4
6
8
10
12
Distance (nts)

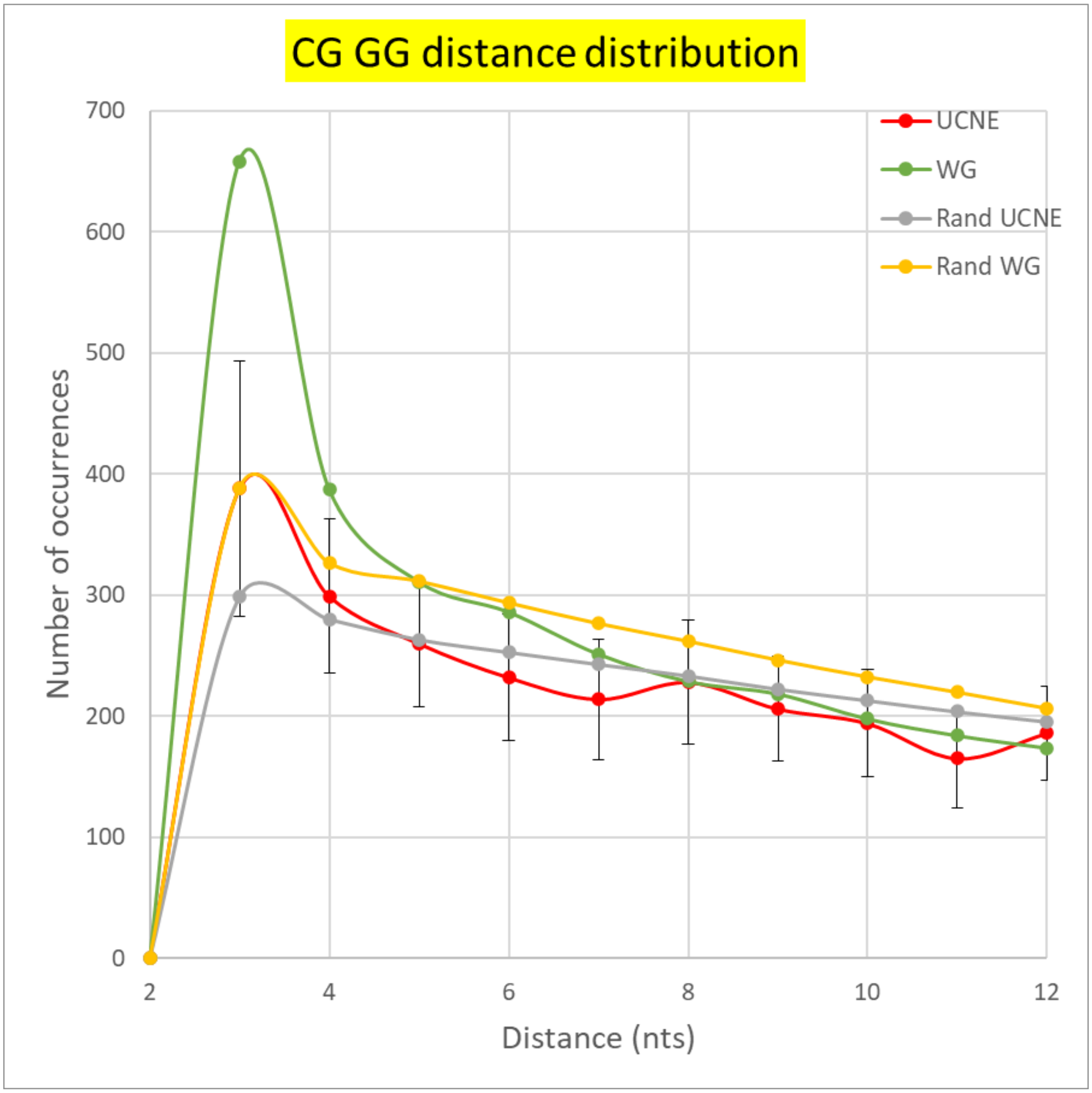

CG GG distance distribution
Number of occurrences
700
600
500
400
300
200
100
0
2
4
6
8
10
12
Distance (nts)
UCNE
WG
Rand UCNE
Rand WG

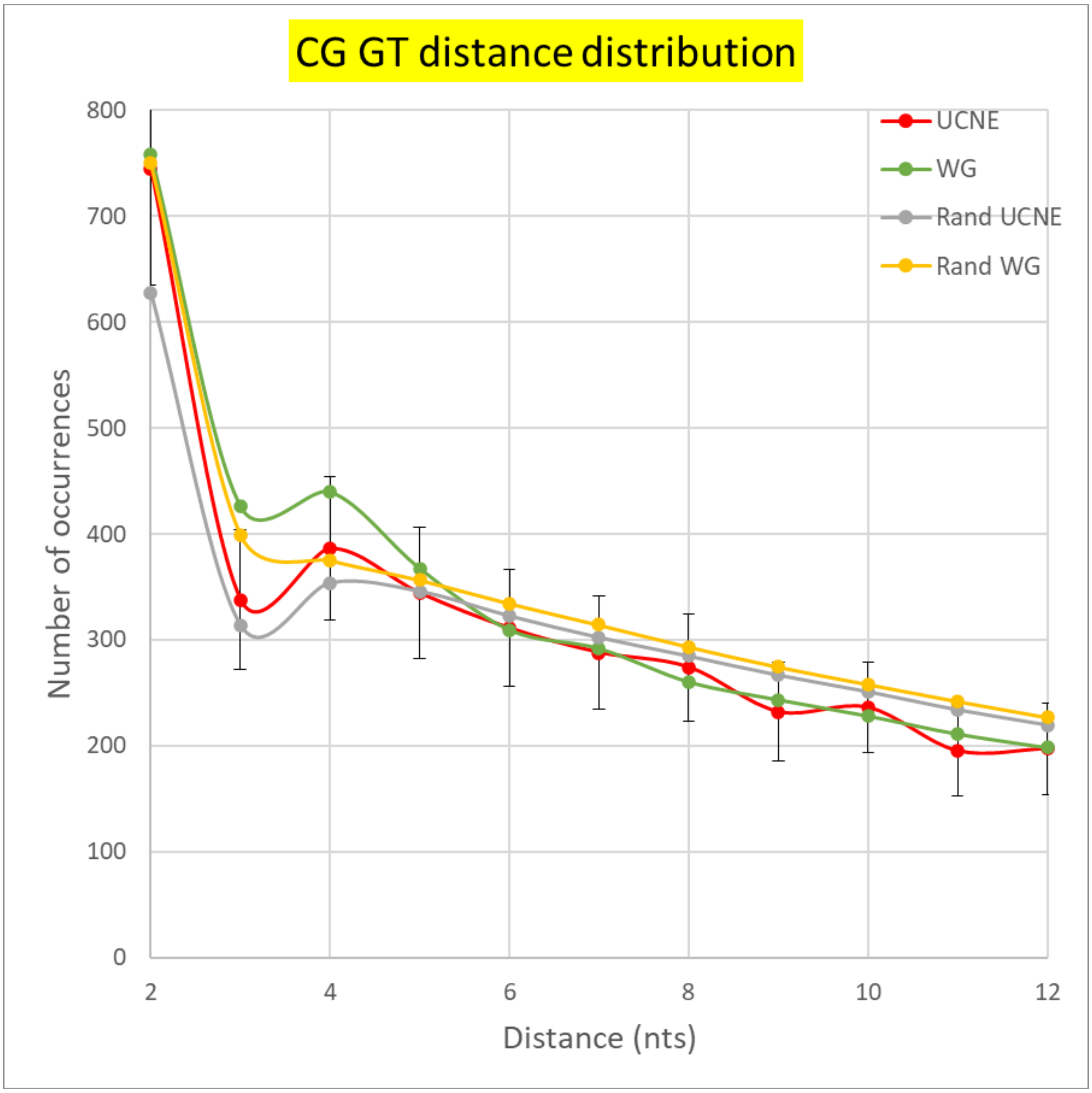

CG GT distance distribution
UCNE
WG
Rand UCNE
Rand WG
Number of occurrences
0
100
200
300
400
500
600
700
800
2
4
6
8
10
12
Distance (nts)

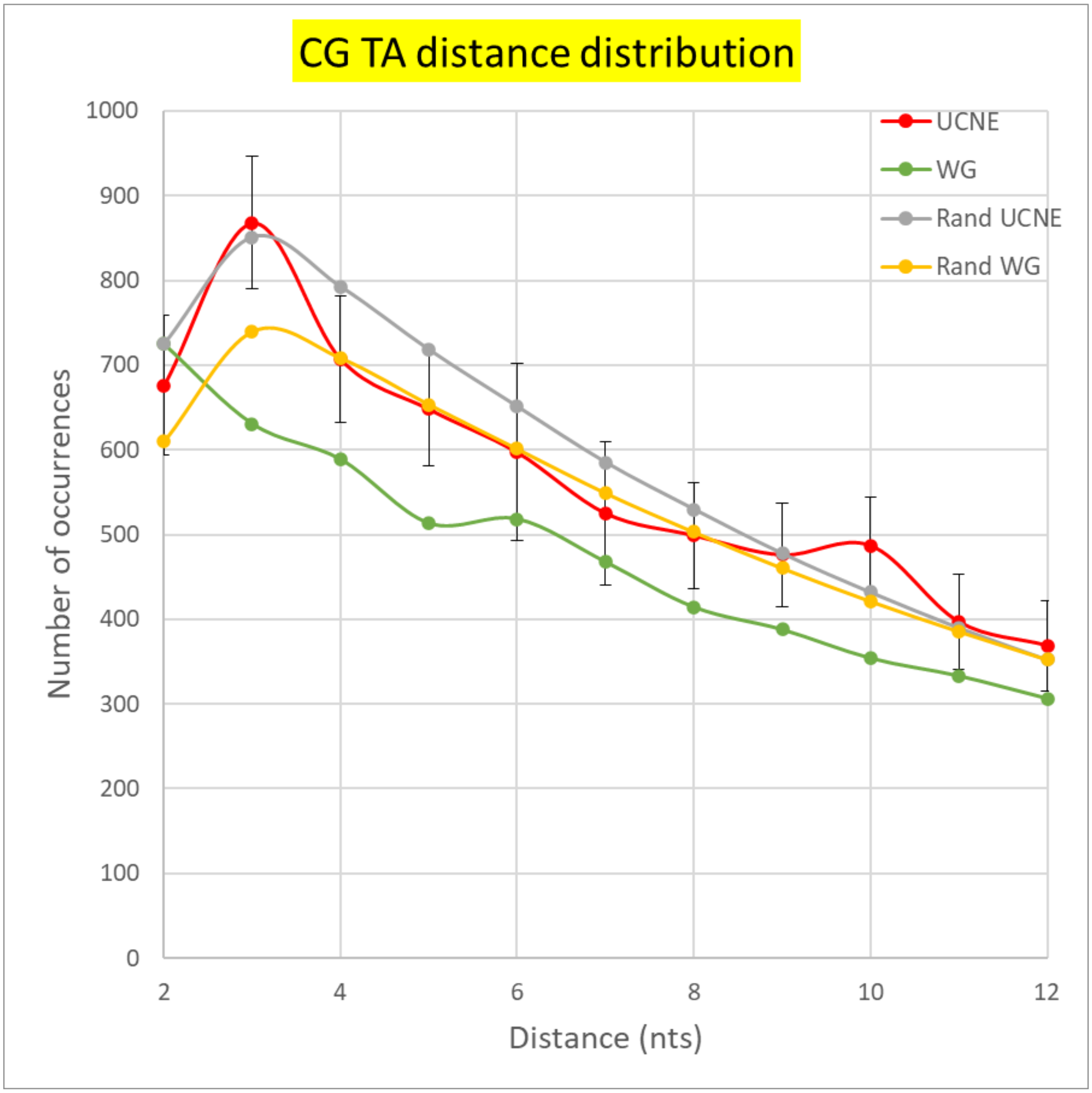
CG TA distance distribution
UCNE
WG
Rand UCNE
Rand WG
Number of occurrences
Distance (nts)
0
100
200
300
400
500
600
700
800
900
1000
2
4
6
8
10
12

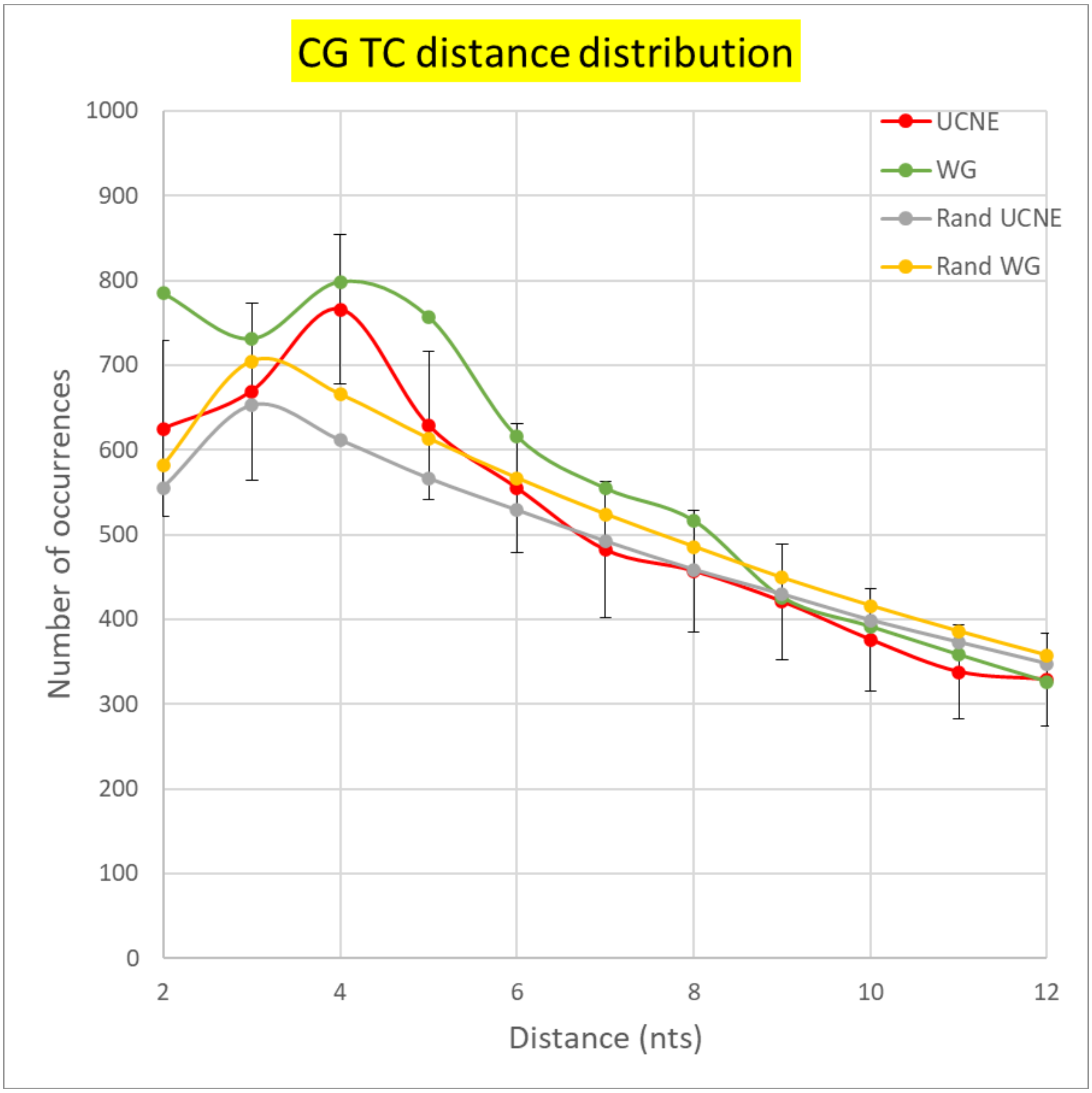
CG TC distance distribution
Number of occurrences
Distance (nts)
UCNE
WG
Rand UCNE
Rand WG
1000
900
800
700
600
500
400
300
200
100
0
2
4
6
8
10
12

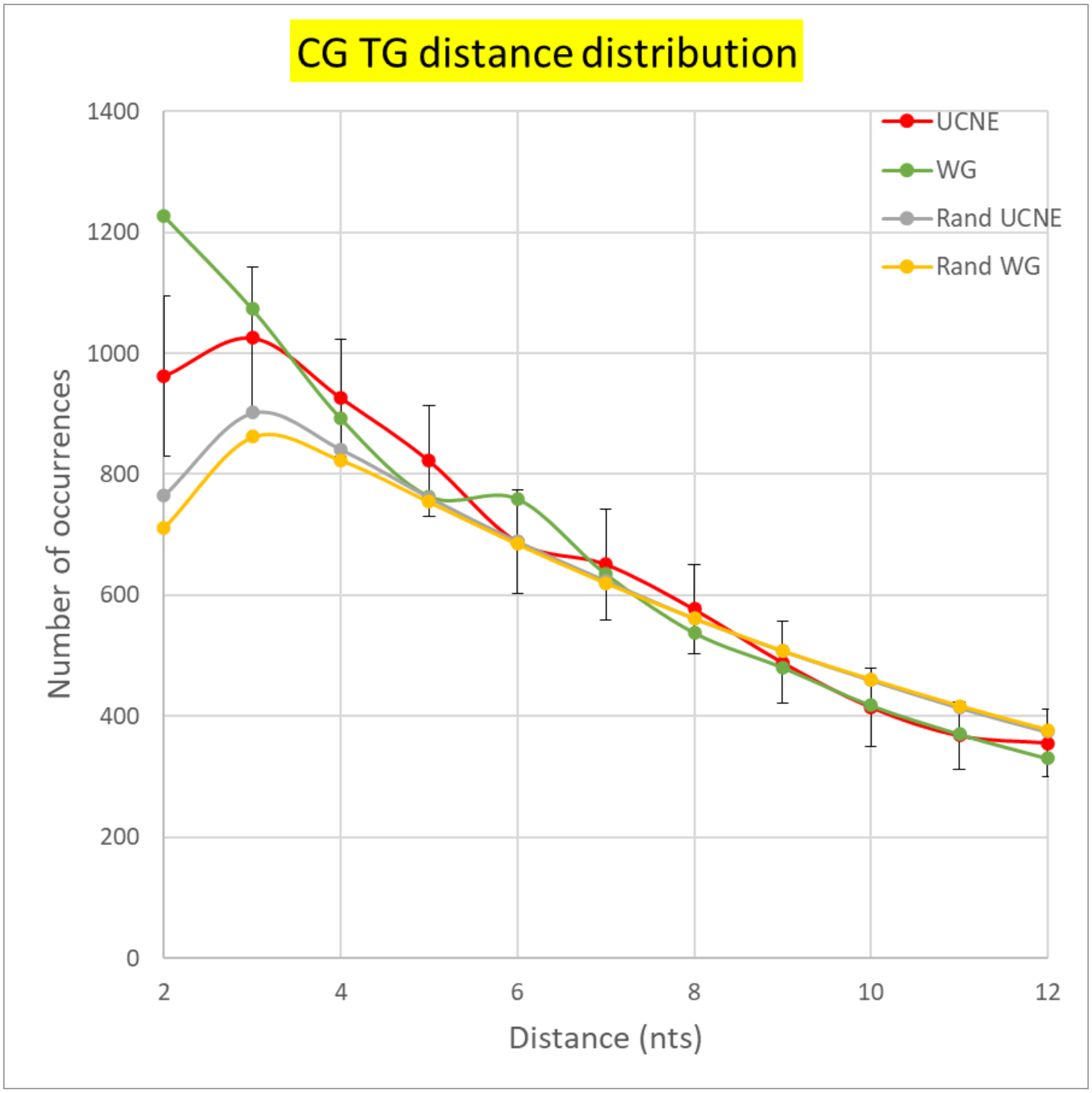

CG TG distance distribution
Number of occurrences
Distance (nts)
UCNE
WG
Rand UCNE
Rand WG
0
200
400
600
800
1000
1200
1400
2
4
6
8
10
12

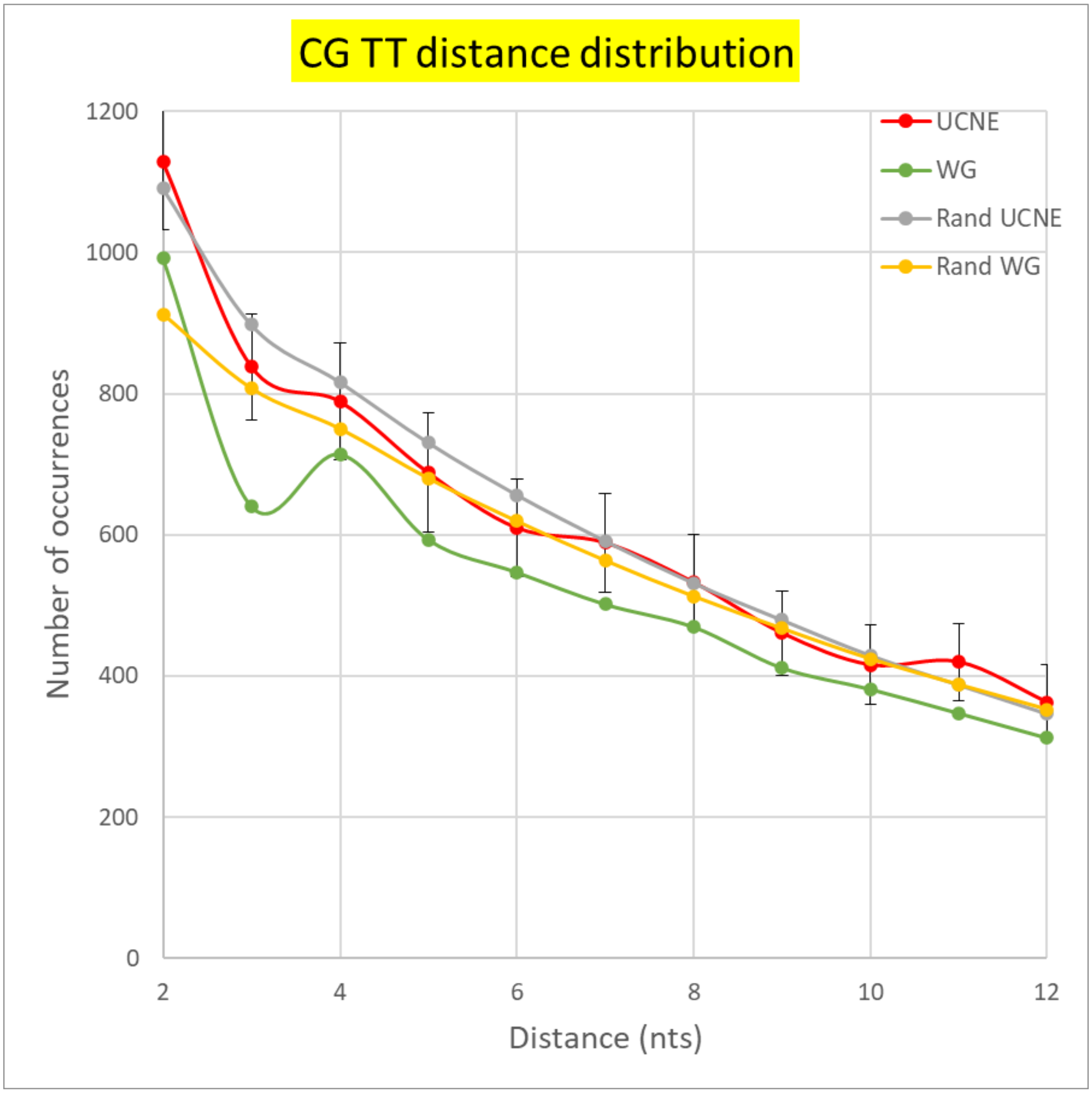

CG TT distance distribution
UCNE
WG
Rand UCNE
Rand WG
Number of occurrences
0
200
400
600
800
1000
1200
2
4
6
8
10
12
Distance (nts)

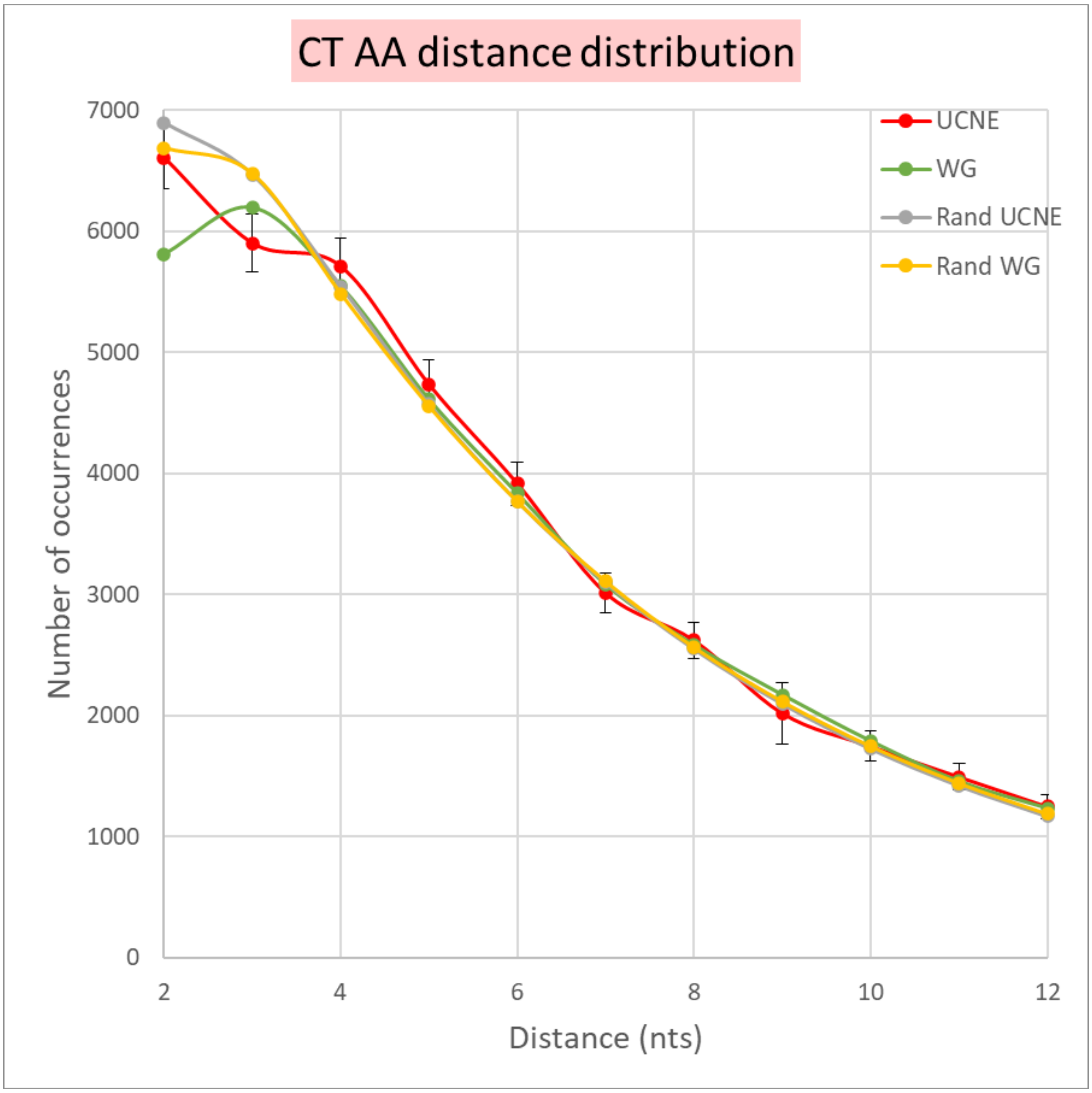

CT AA distance distribution
7000
6000
5000
4000
3000
2000
1000
0
Number of occurrences
2
4
6
8
10
12
Distance (nts)
UCNE
WG
Rand UCNE
Rand WG

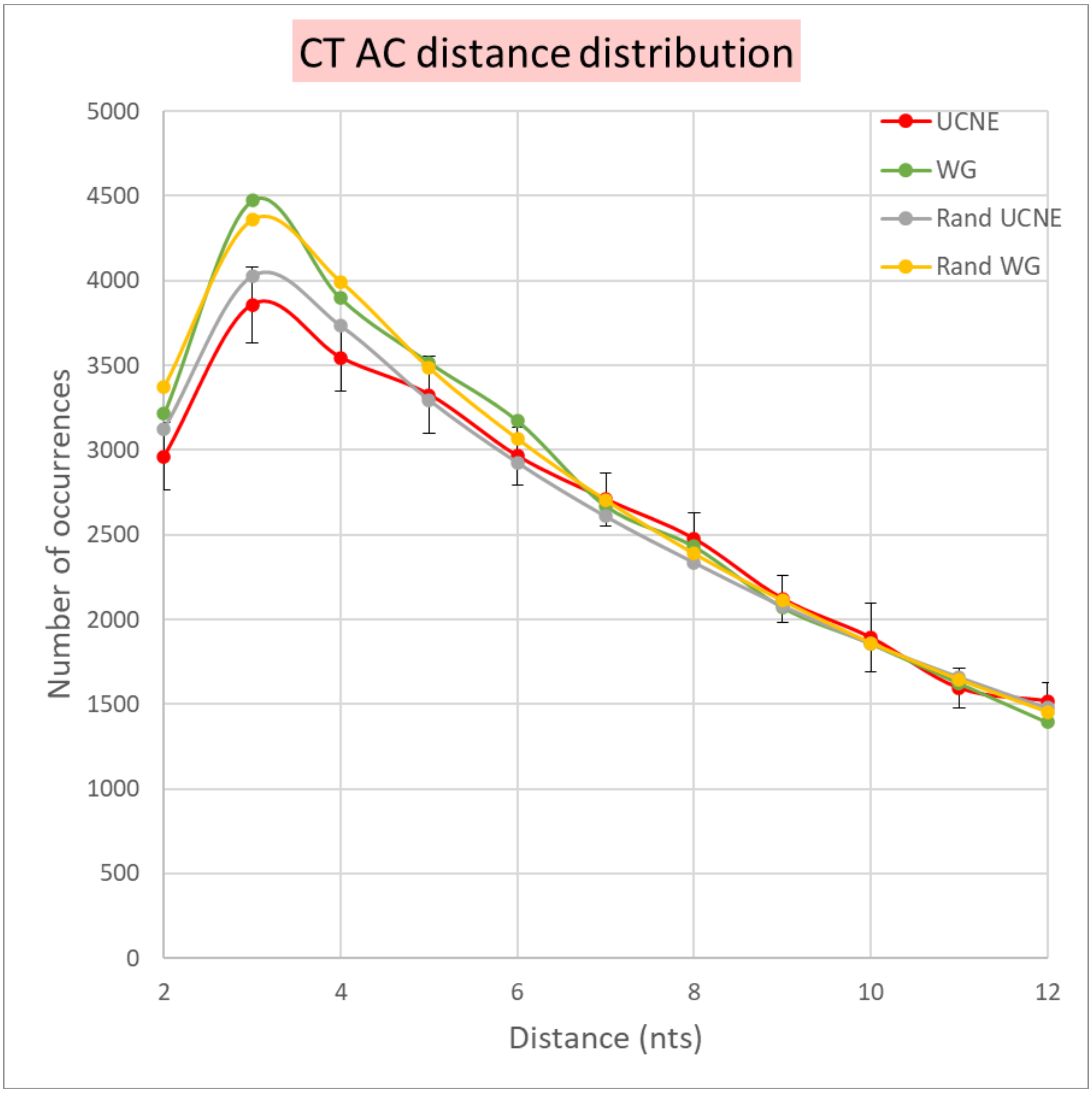
CT AC distance distribution
Number of occurrences
Distance (nts)
UCNE
WG
Rand UCNE
Rand WG
0
500
1000
1500
2000
2500
3000
3500
4000
4500
5000
2
4
6
8
10
12

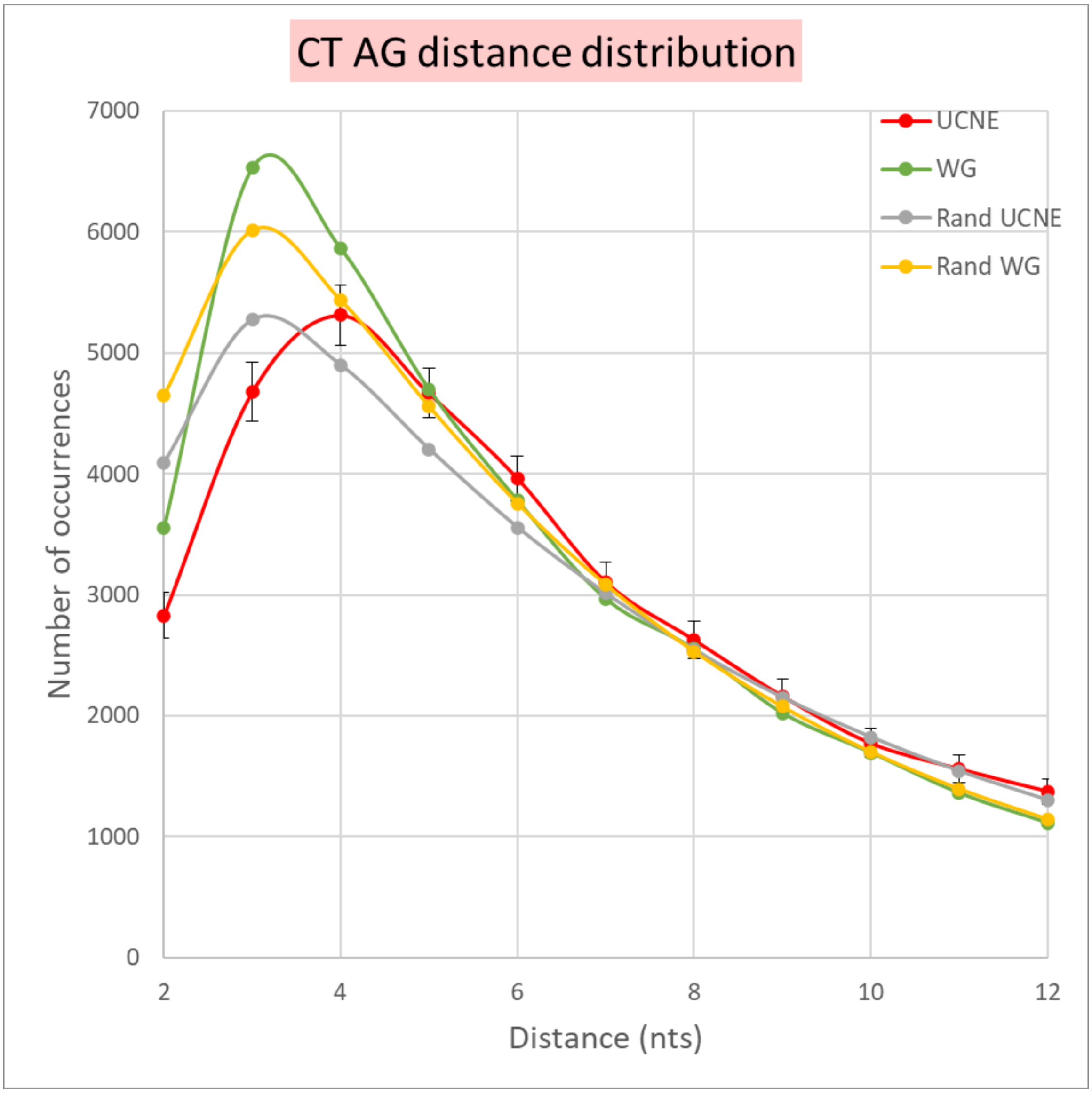
CT AG distance distribution
7000
6000
5000
4000
3000
2000
1000
0
2
4
6
8
10
12
Number of occurrences
Distance (nts)
UCNE
WG
Rand UCNE
Rand WG

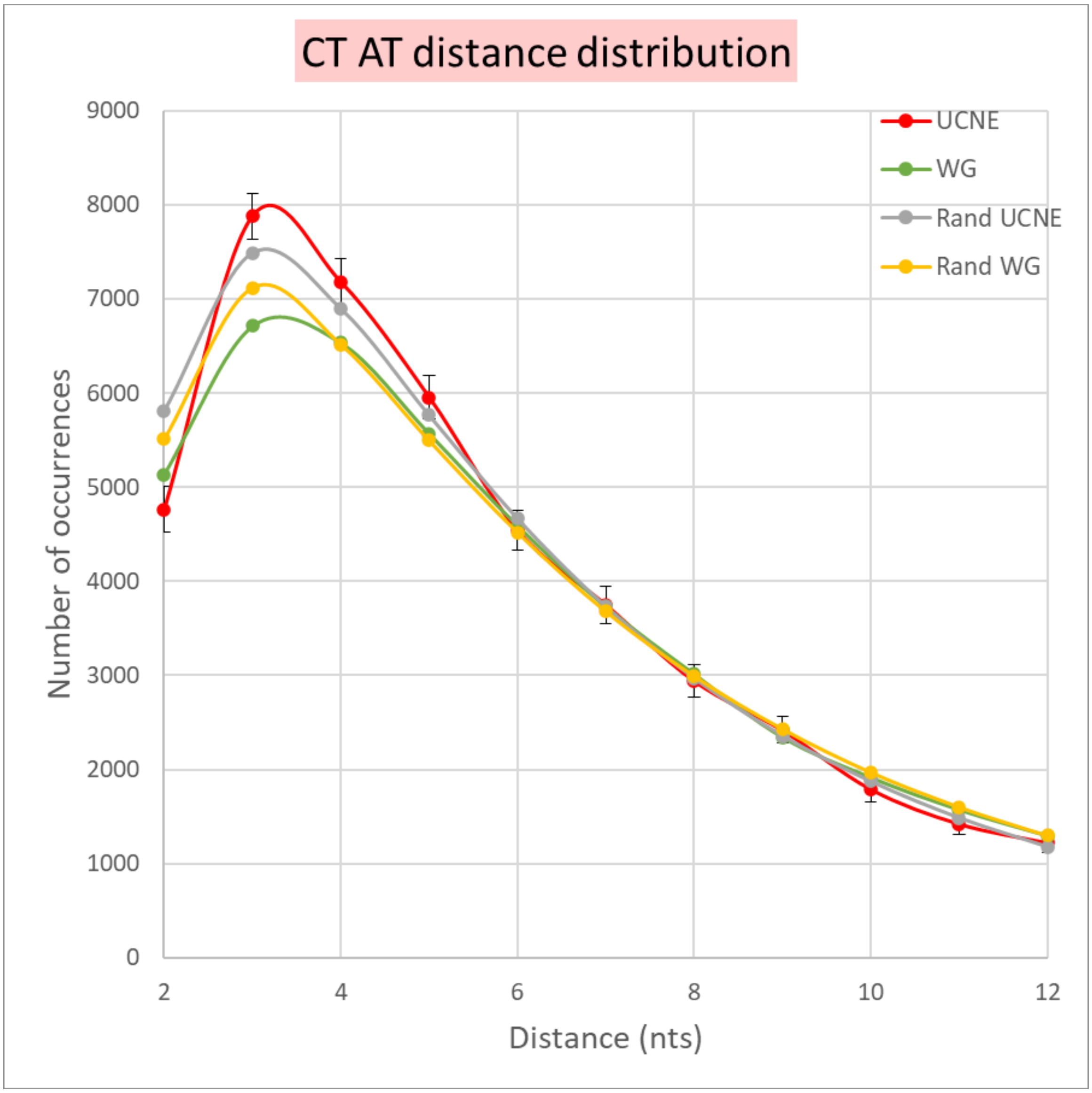
CT AT distance distribution
UCNE
WG
Rand UCNE
Rand WG
Number of occurrences
0
1000
2000
3000
4000
5000
6000
7000
8000
9000
2
4
6
8
10
12
Distance (nts)

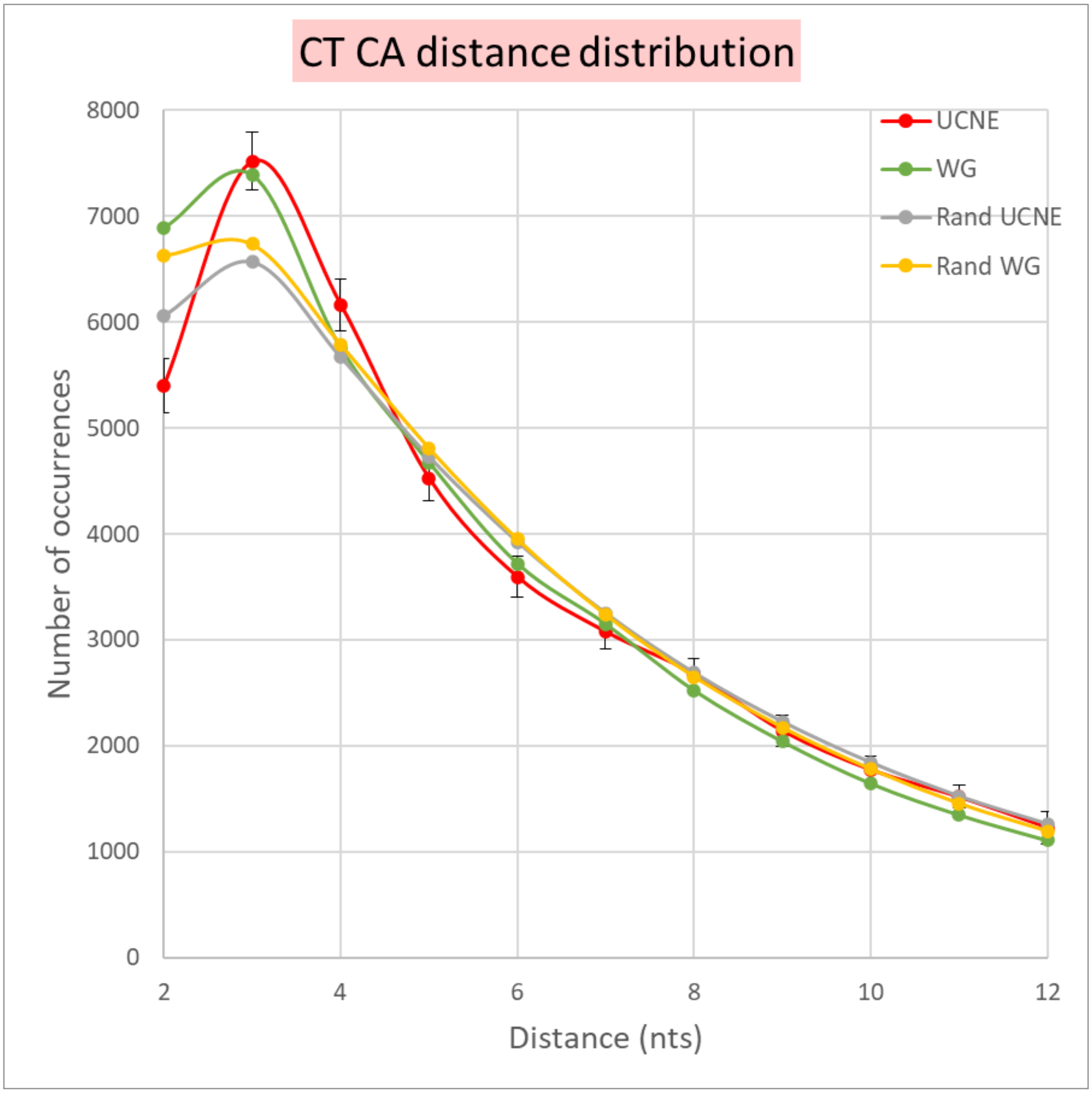

CT CA distance distribution
UCNE
WG
Rand UCNE
Rand WG
Number of occurrences
8000
7000
6000
5000
4000
3000
2000
1000
0
2
4
6
8
10
12
Distance (nts)

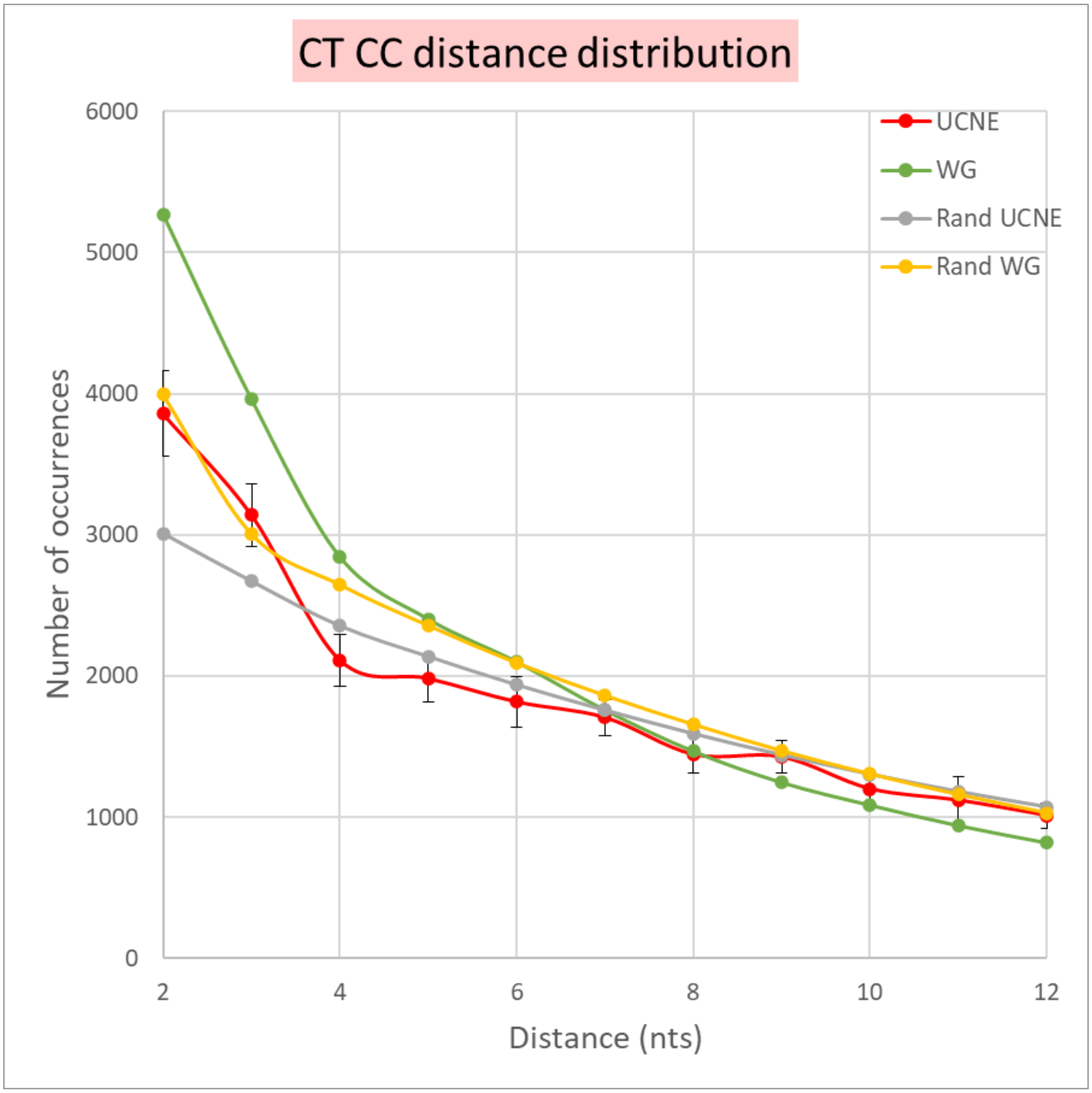

CT CC distance distribution
UCNE
WG
Rand UCNE
Rand WG
Number of occurrences
6000
5000
4000
3000
2000
1000
0
2
4
6
8
10
12
Distance (nts)

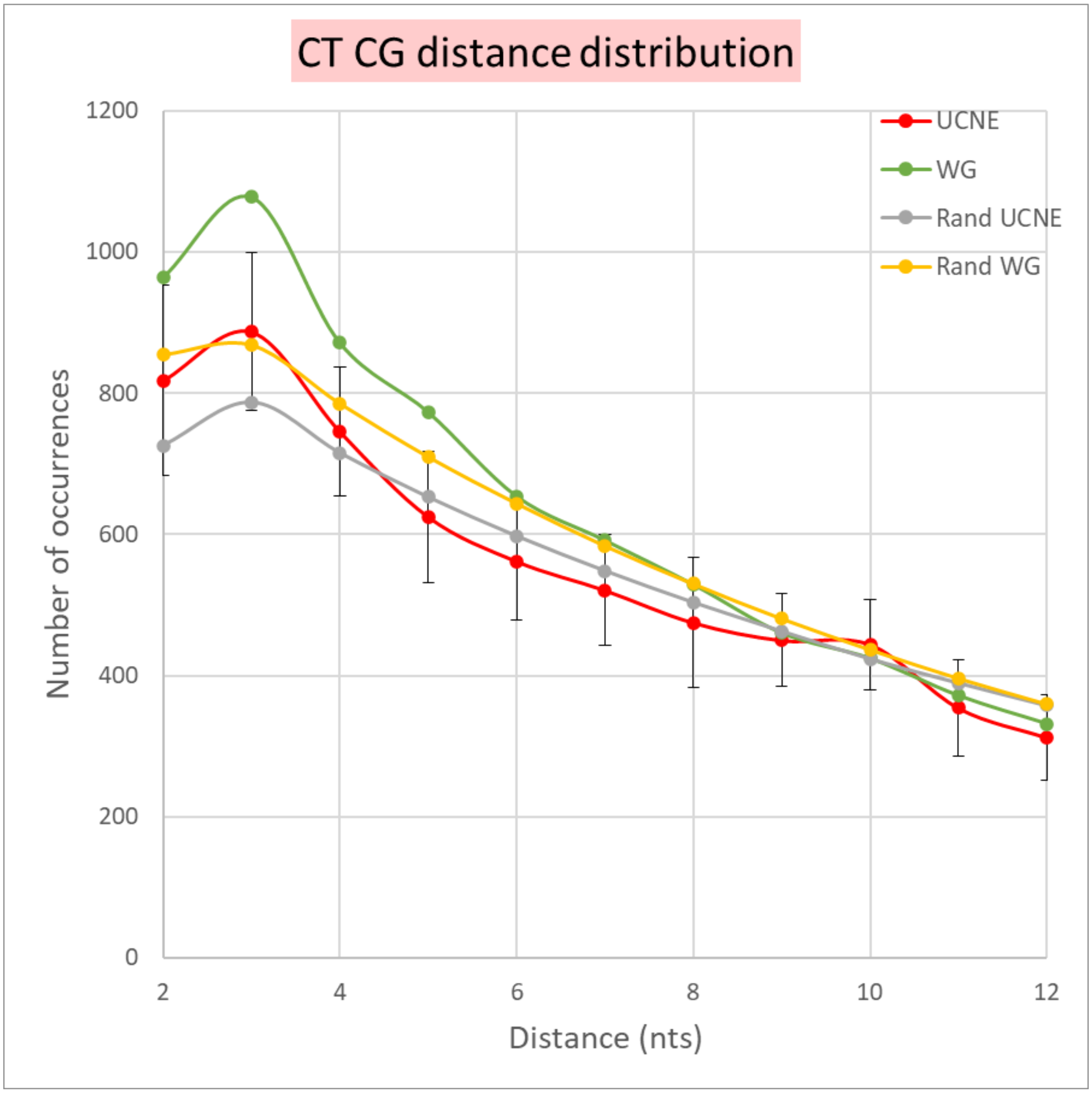
CT CG distance distribution
UCNE
WG
Rand UCNE
Rand WG
Number of occurrences
Distance (nts)
0
200
400
600
800
1000
1200
2
4
6
8
10
12

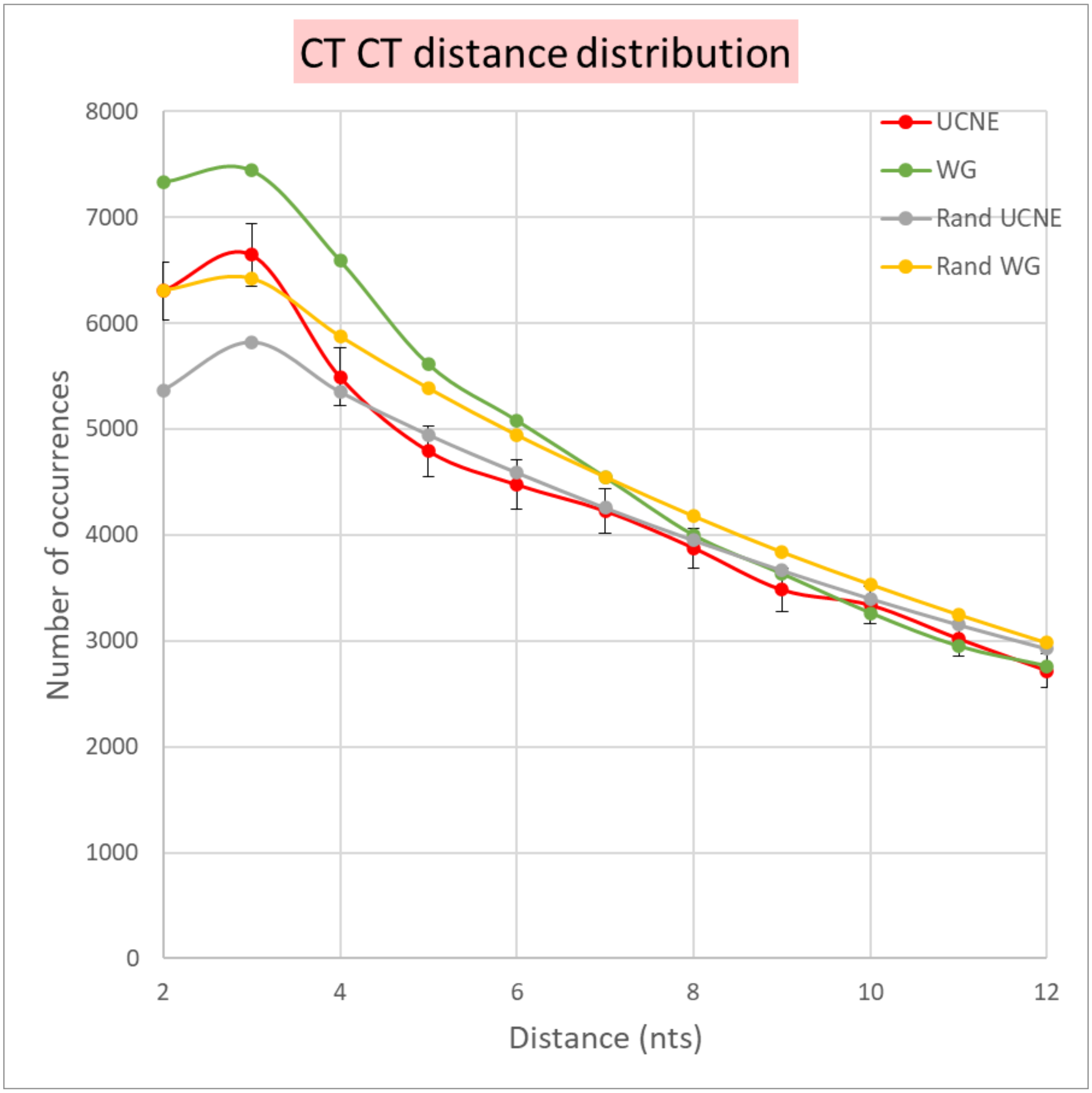

CT CT distance distribution
UCNE
WG
Rand UCNE
Rand WG
Number of occurrences
Distance (nts)
8000
7000
6000
5000
4000
3000
2000
1000
0
2
4
6
8
10
12

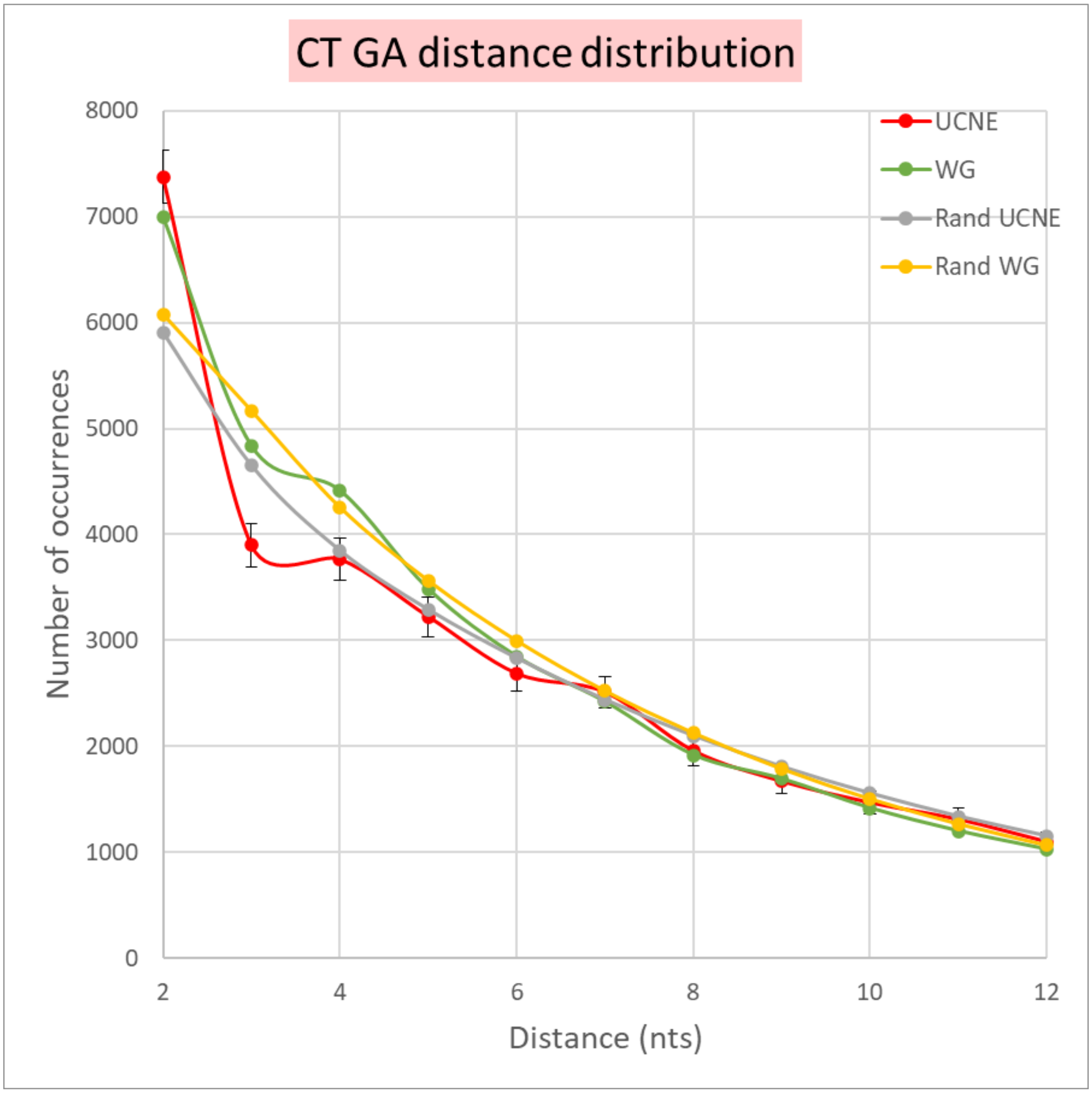

CT GA distance distribution
UCNE
WG
Rand UCNE
Rand WG
8000
7000
6000
5000
4000
3000
2000
1000
0
Number of occurrences
2
4
6
8
10
12
Distance (nts)

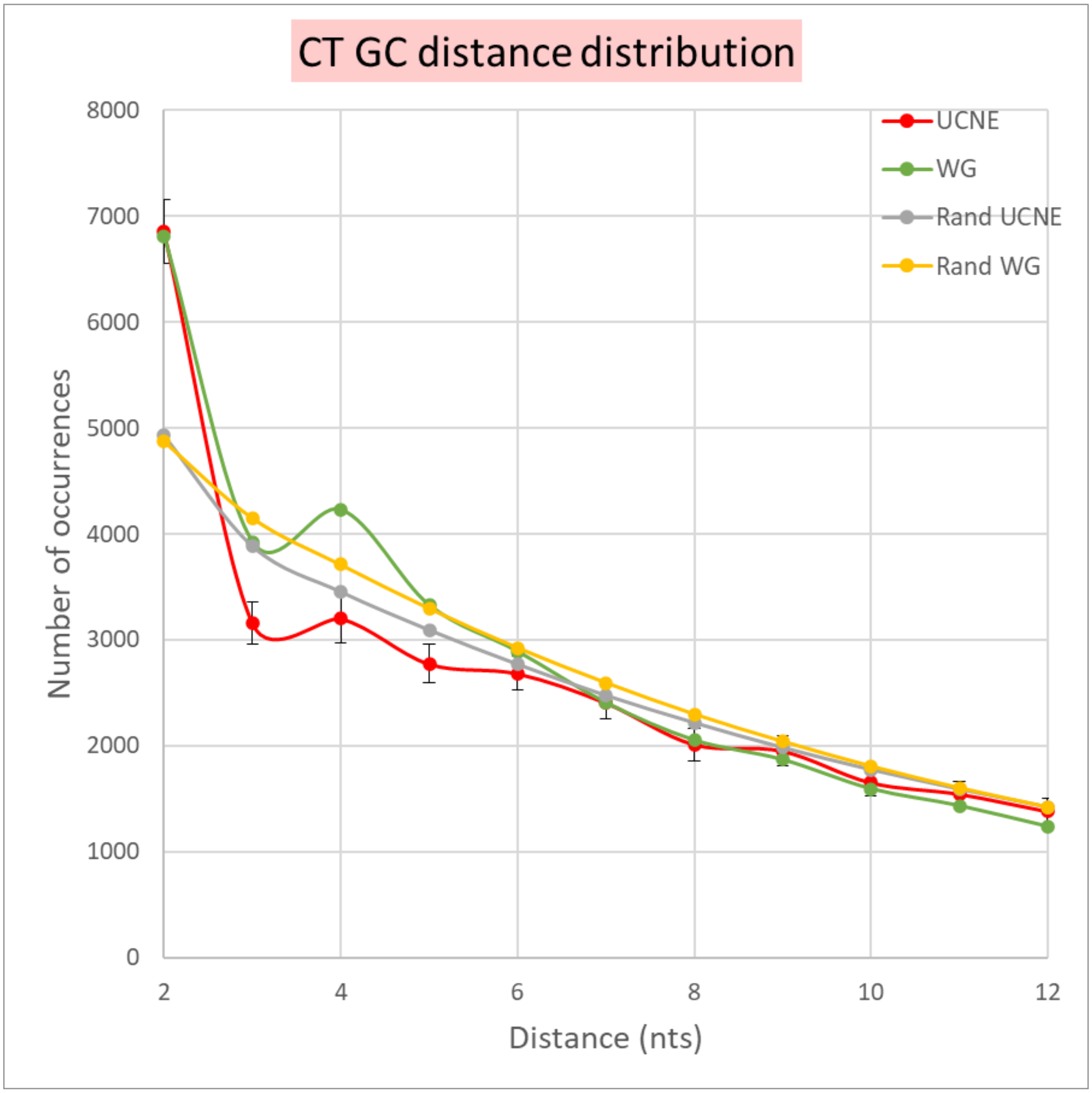

CT GC distance distribution
UCNE
WG
Rand UCNE
Rand WG
Number of occurrences
Distance (nts)
0
1000
2000
3000
4000
5000
6000
7000
8000
2
4
6
8
10
12

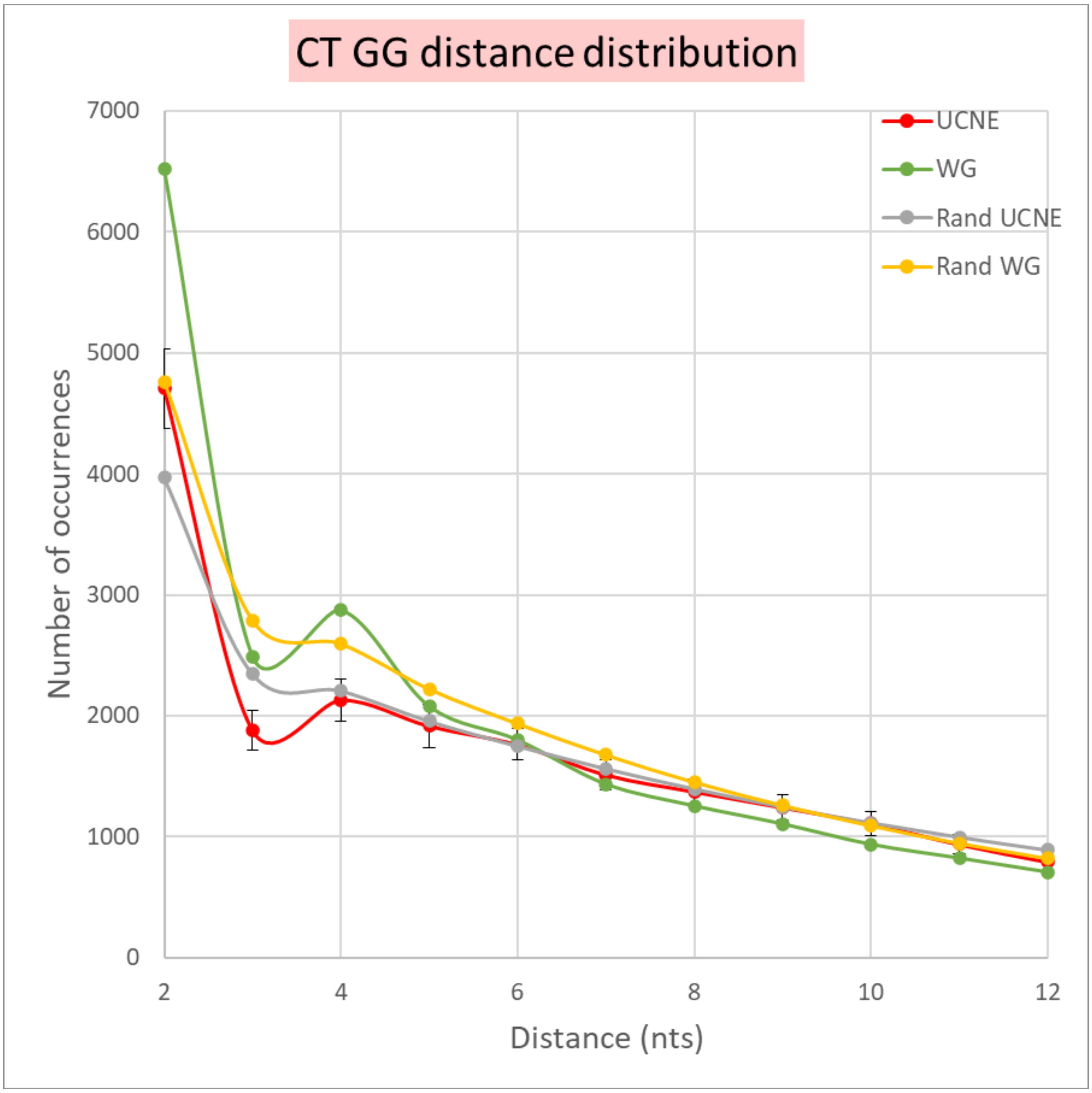

CT GG distance distribution
UCNE
WG
Rand UCNE
Rand WG
Number of occurrences
7000
6000
5000
4000
3000
2000
1000
0
2
4
6
8
10
12
Distance (nts)

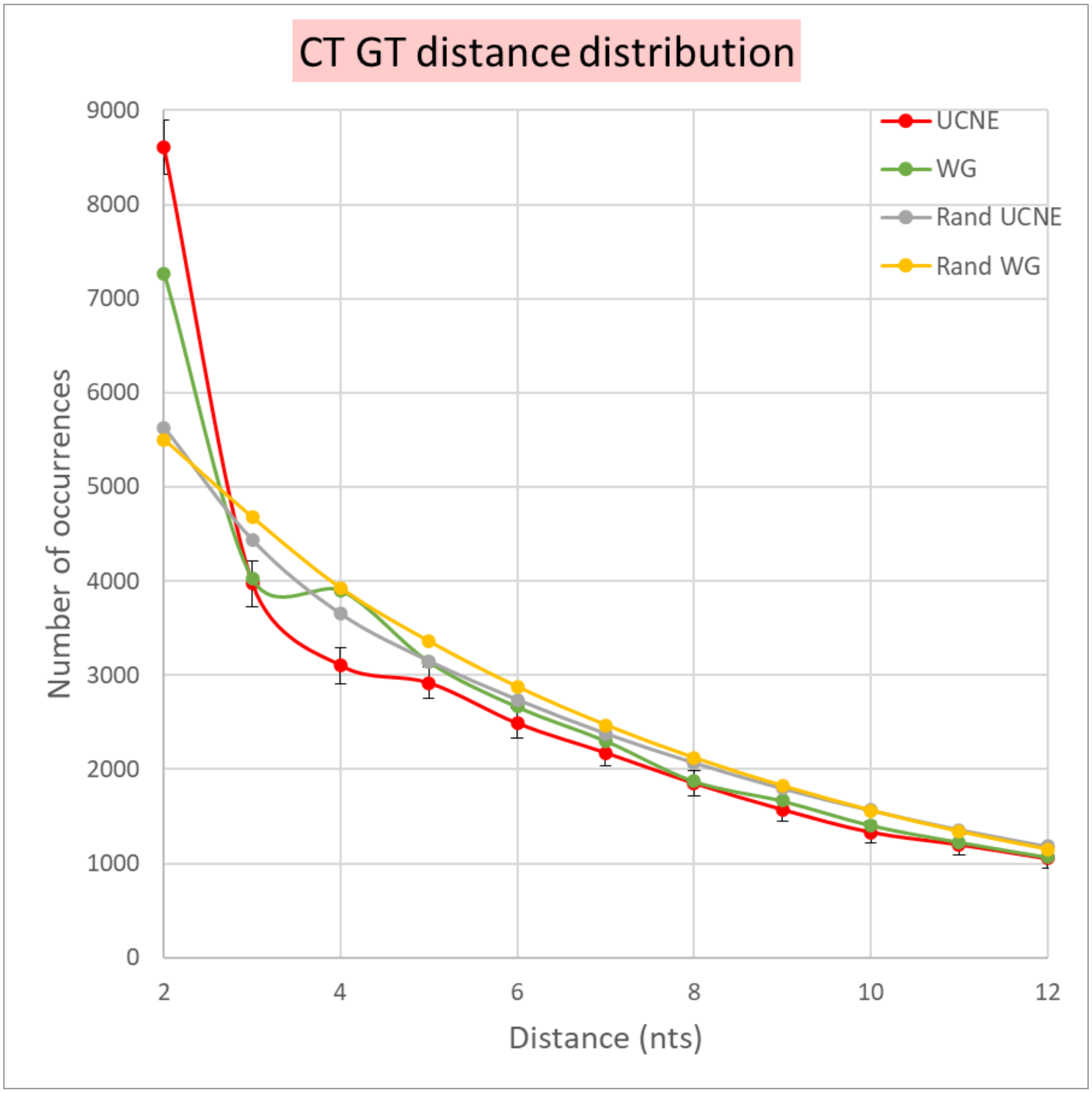

CT GT distance distribution
UCNE
WG
Rand UCNE
Rand WG
Number of occurrences
9000
8000
7000
6000
5000
4000
3000
2000
1000
0
2
4
6
8
10
12
Distance (nts)

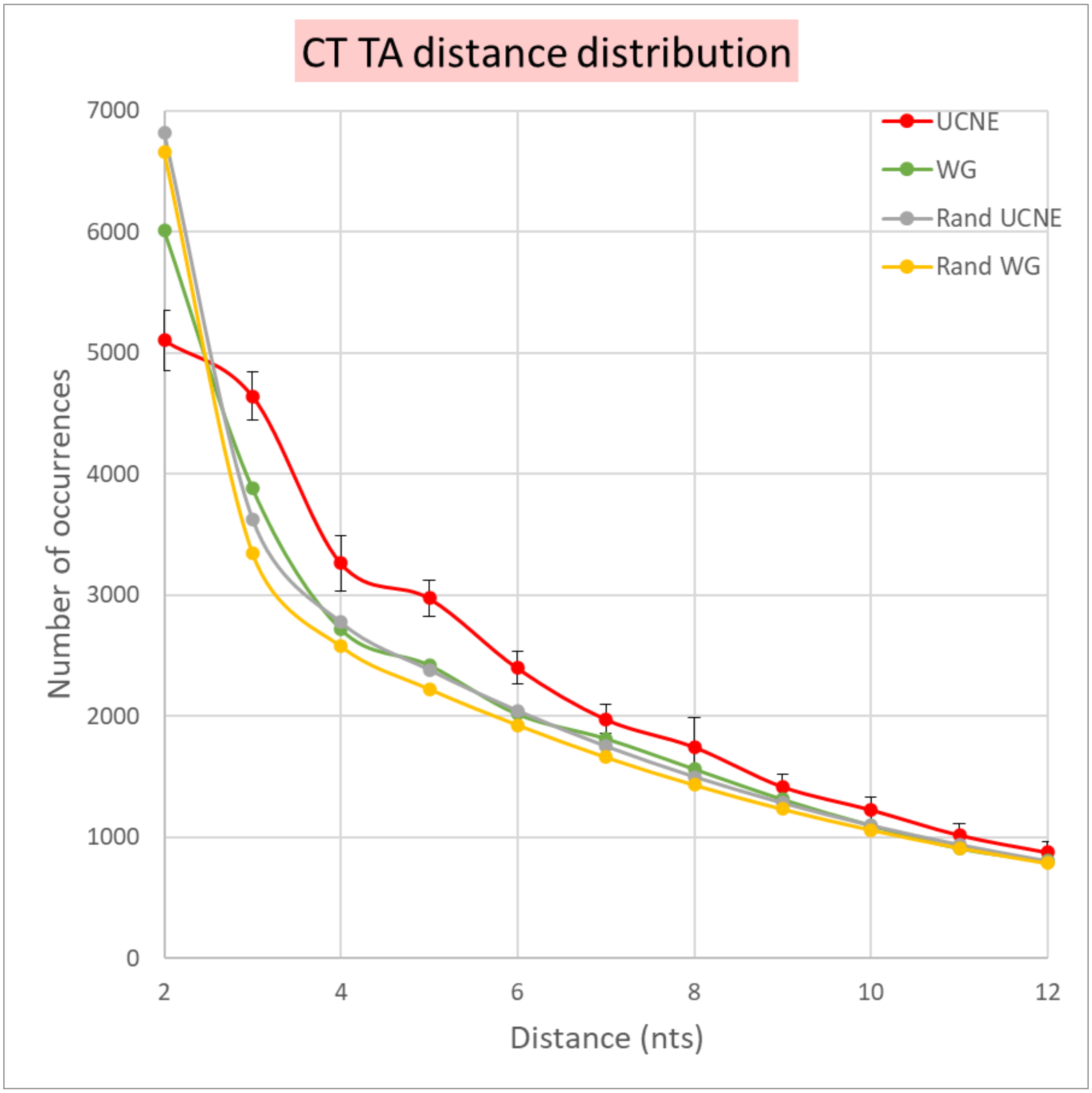

CT TA distance distribution
UCNE
WG
Rand UCNE
Rand WG
Number of occurrences
7000
6000
5000
4000
3000
2000
1000
0
2
4
6
8
10
12
Distance (nts)

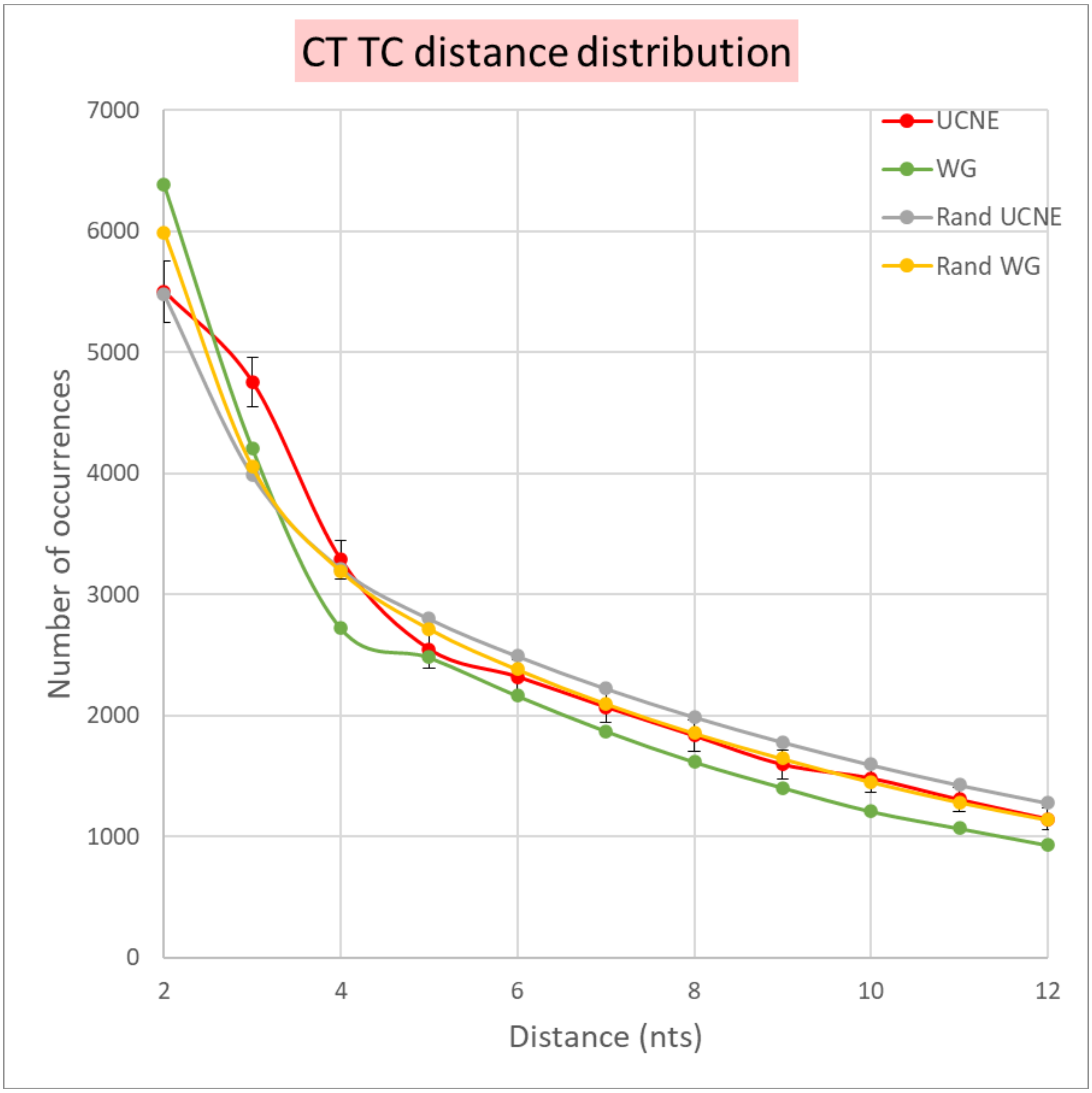
CT TC distance distribution
UCNE
WG
Rand UCNE
Rand WG
Number of occurrences
0
1000
2000
3000
4000
5000
6000
7000
2
4
6
8
10
12
Distance (nts)

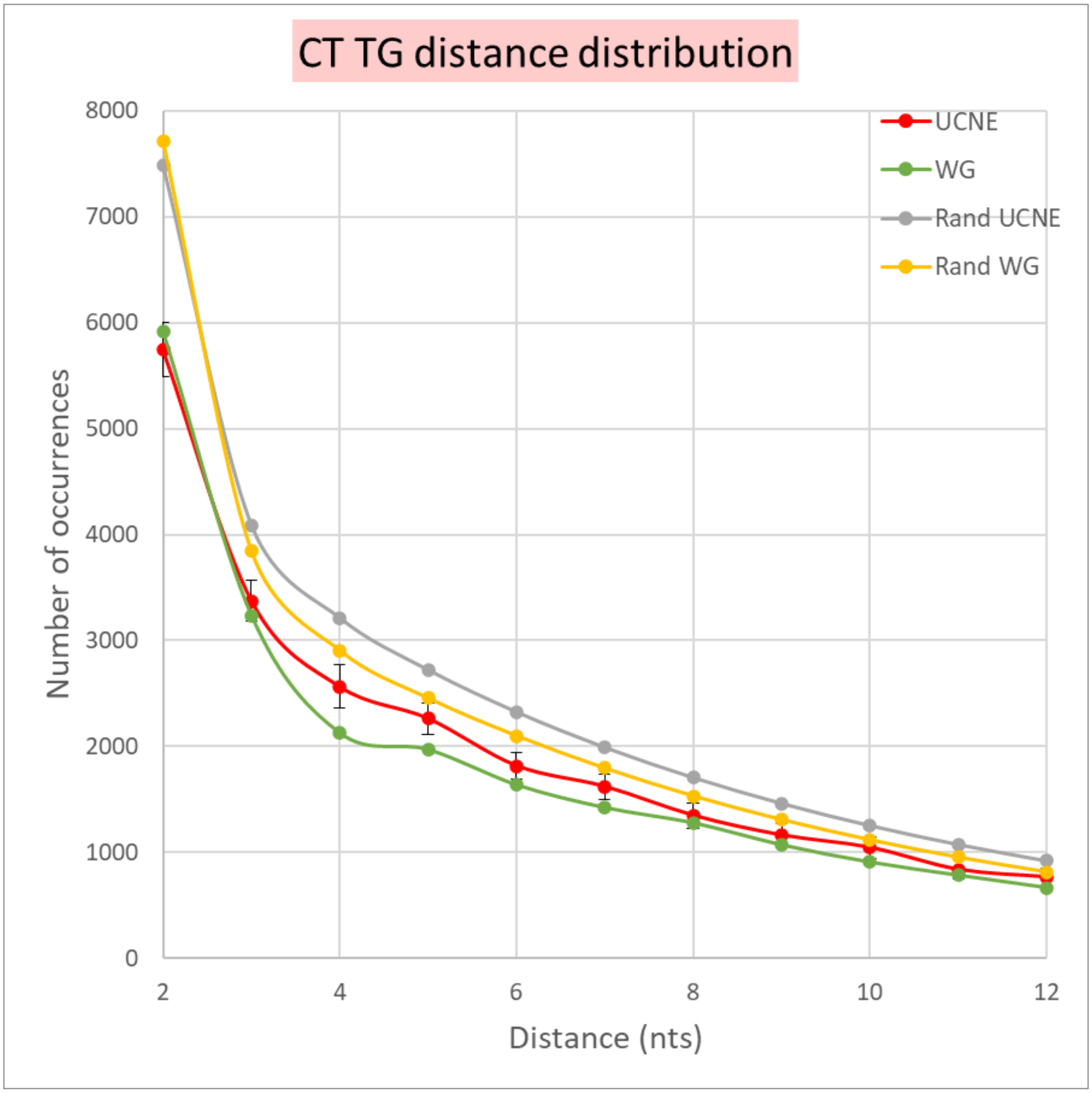
CT TG distance distribution
UCNE
WG
Rand UCNE
Rand WG
Number of occurrences
0
1000
2000
3000
4000
5000
6000
7000
8000
2
4
6
8
10
12
Distance (nts)

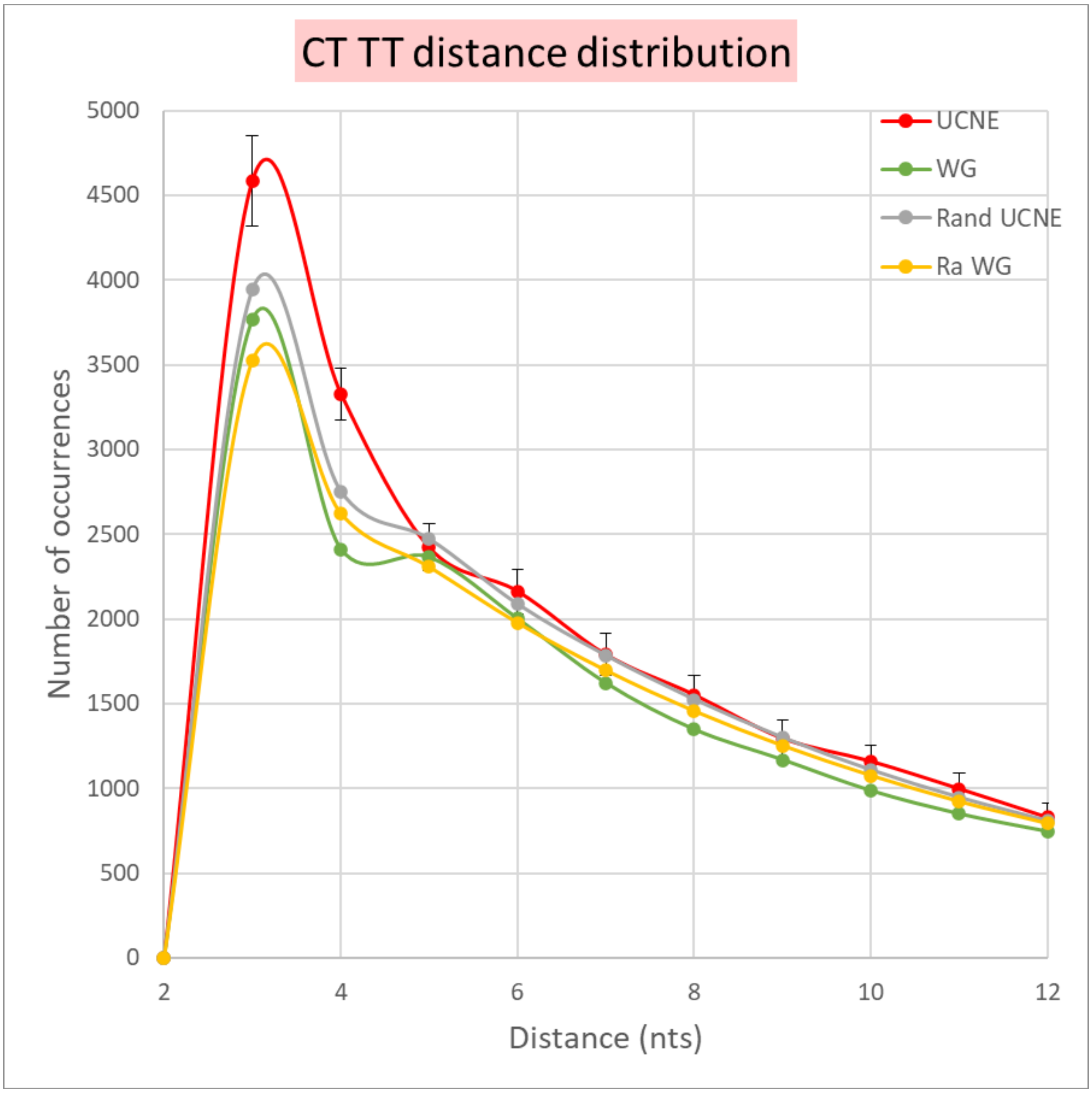
CT TT distance distribution
UCNE
WG
Rand UCNE
Ra WG
Number of occurrences
5000
4500
4000
3500
3000
2500
2000
1500
1000
500
0
2
4
6
8
10
12
Distance (nts)

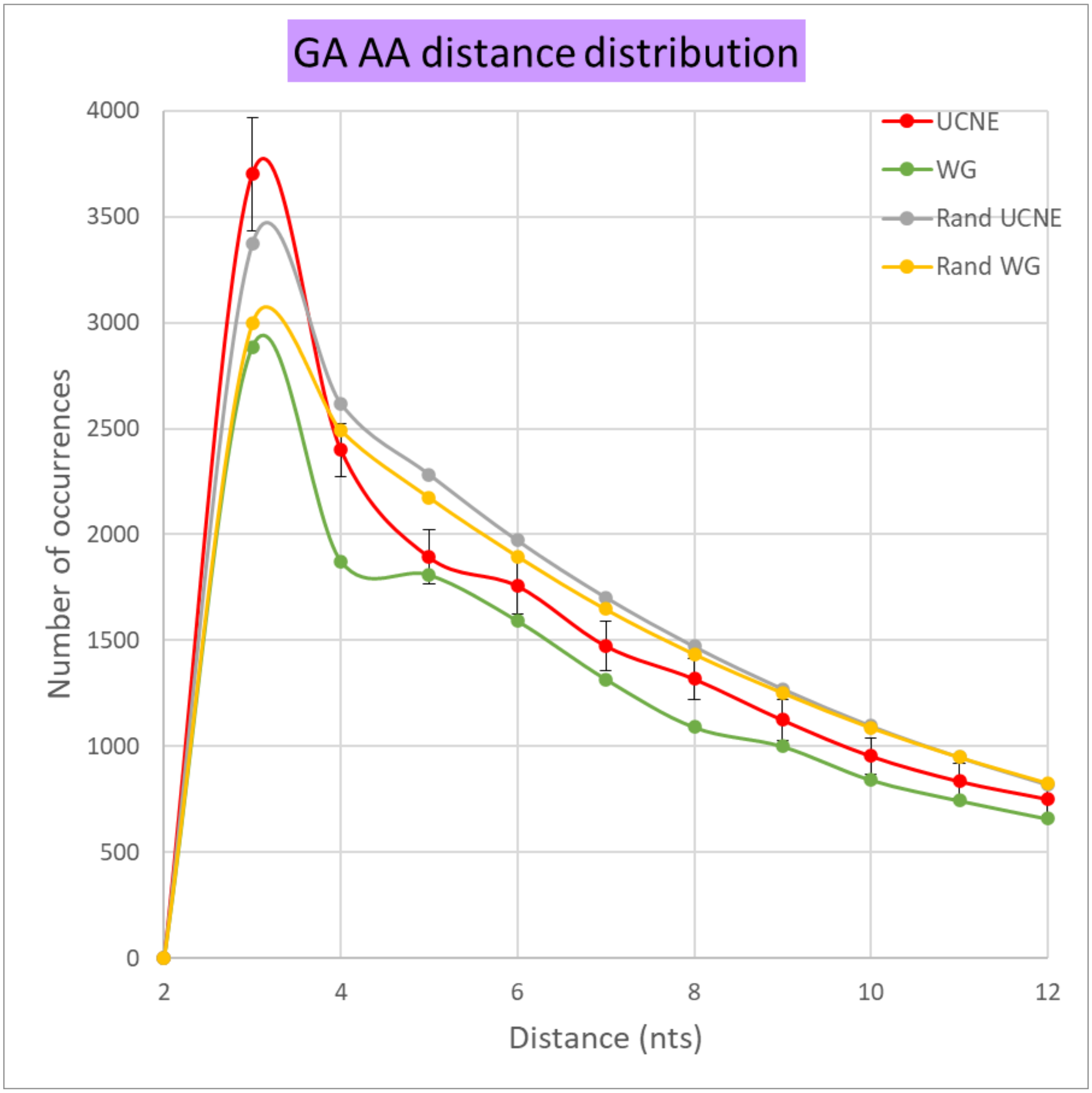

GA AA distance distribution
Number of occurrences
Distance (nts)
UCNE
WG
Rand UCNE
Rand WG
0
500
1000
1500
2000
2500
3000
3500
4000
2
4
6
8
10
12

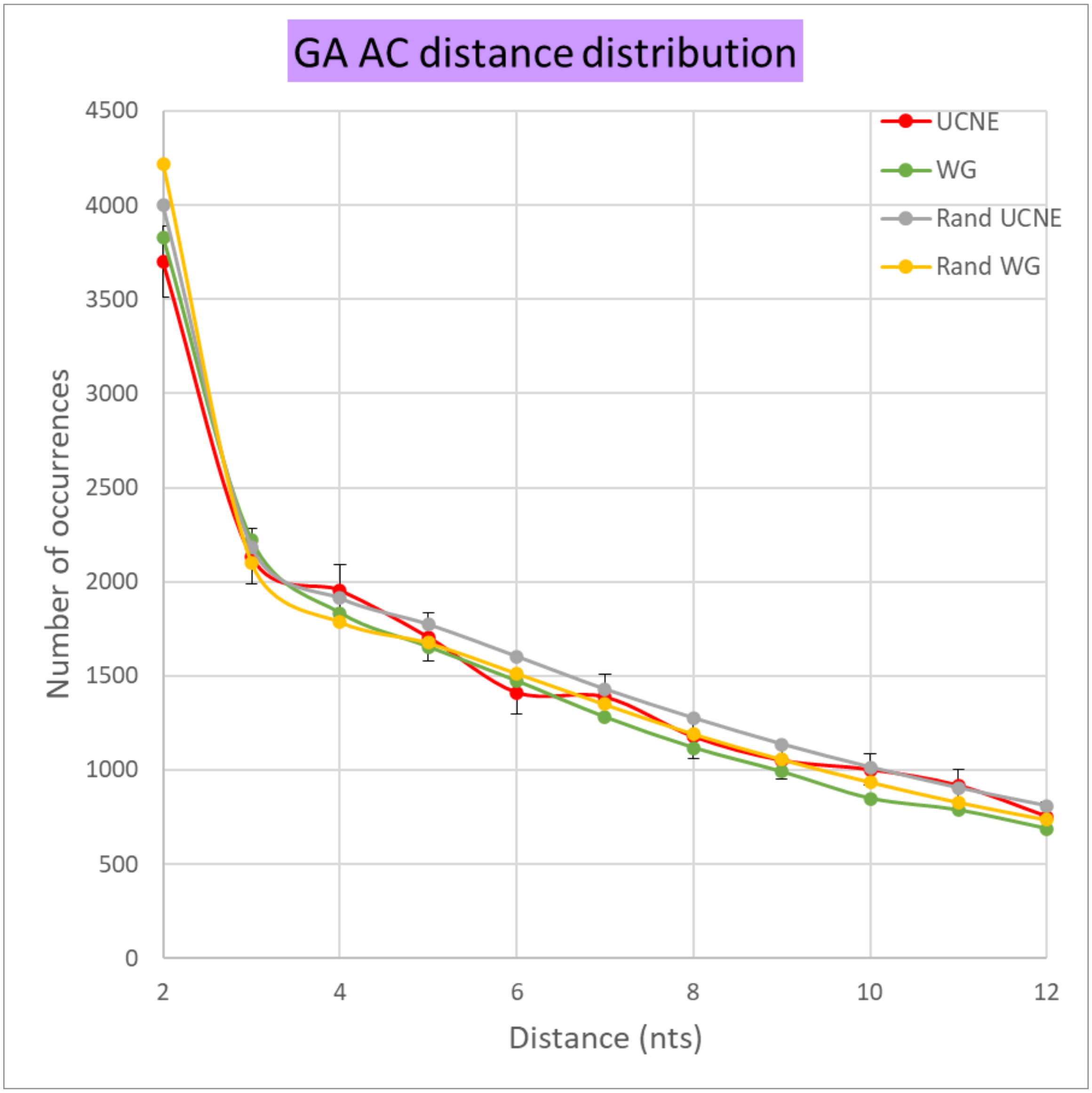
GA AC distance distribution
4500
4000
3500
3000
2500
2000
1500
1000
500
0
Number of occurrences
2
4
6
8
10
12
Distance (nts)
UCNE
WG
Rand UCNE
Rand WG

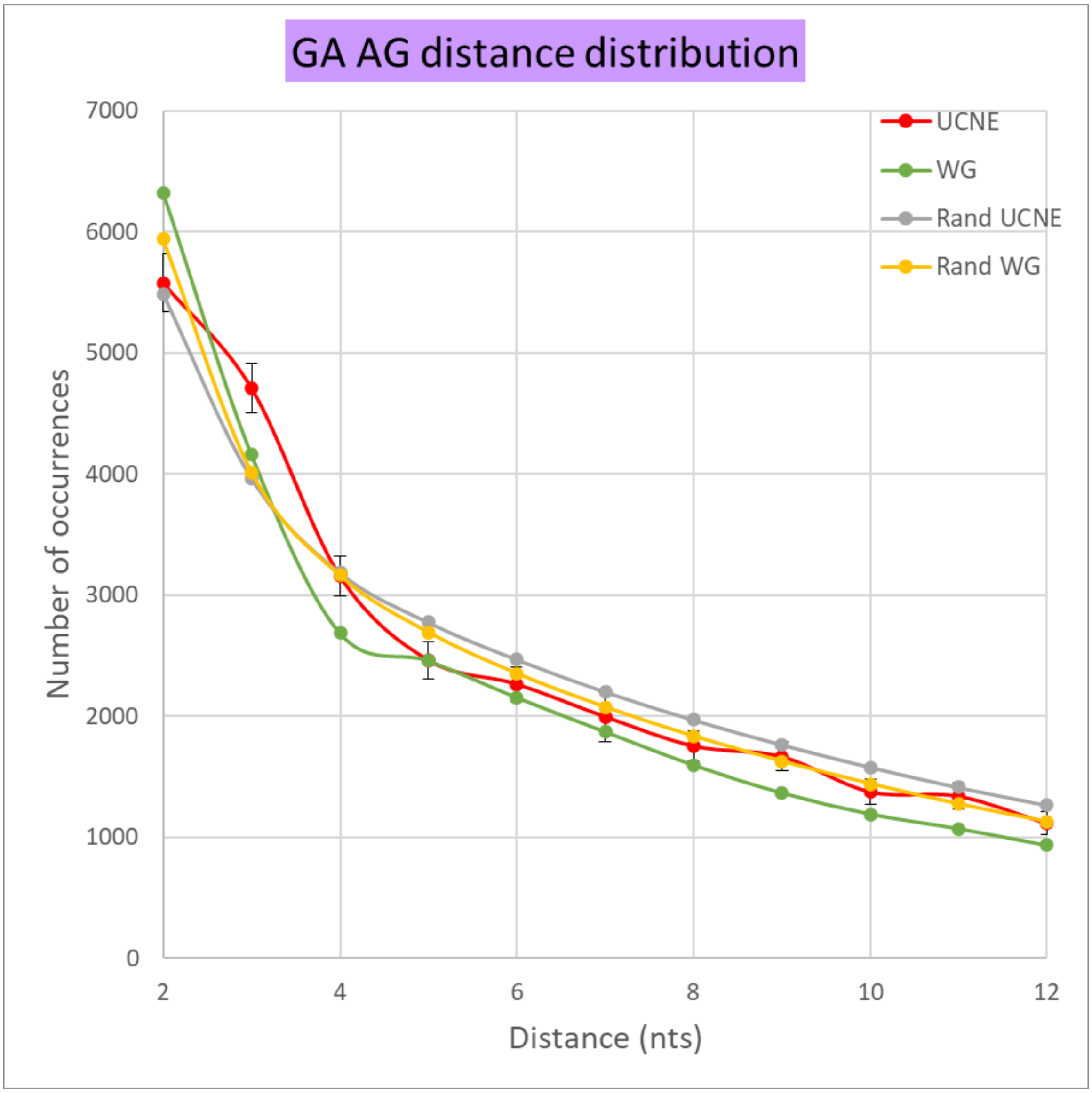

GA AG distance distribution
UCNE
WG
Rand UCNE
Rand WG
Number of occurrences
0
1000
2000
3000
4000
5000
6000
7000
2
4
6
8
10
12
Distance (nts)

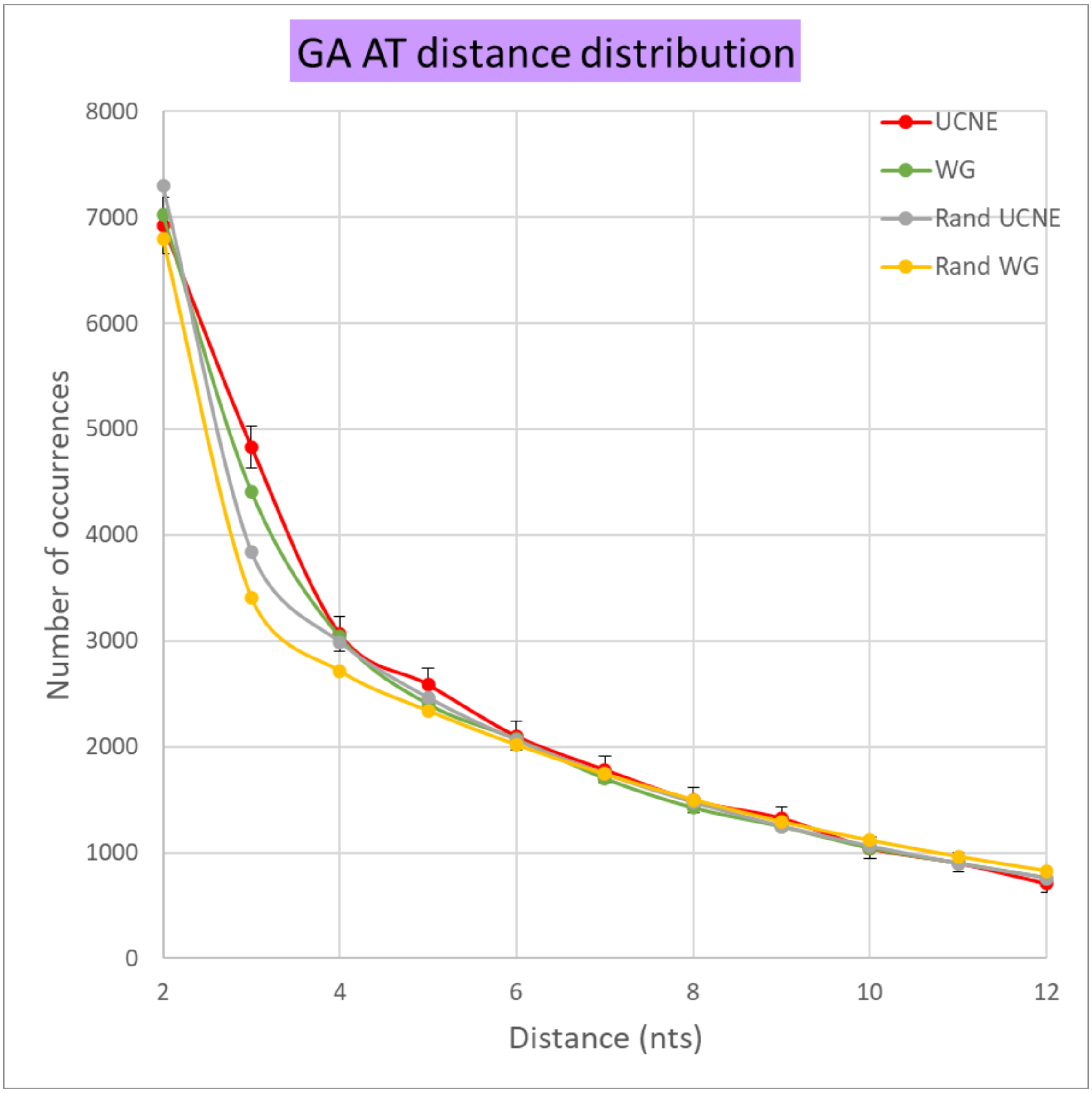

GA AT distance distribution
UCNE
WG
Rand UCNE
Rand WG
Number of occurrences
8000
7000
6000
5000
4000
3000
2000
1000
0
2
4
6
8
10
12
Distance (nts)

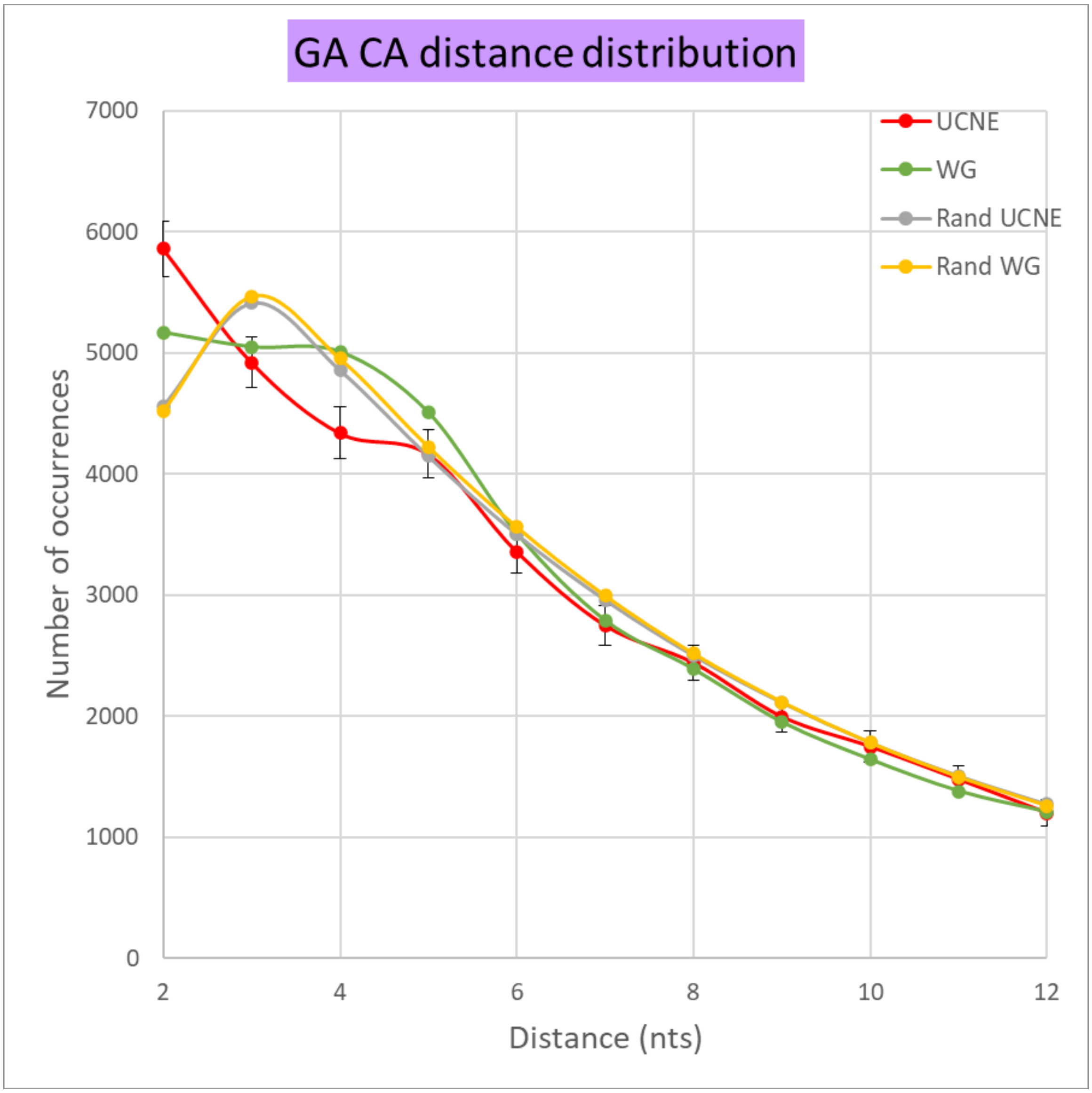

GA CA distance distribution
UCNE
WG
Rand UCNE
Rand WG
7000
6000
5000
4000
3000
2000
1000
0
Number of occurrences
2
4
6
8
10
12
Distance (nts)

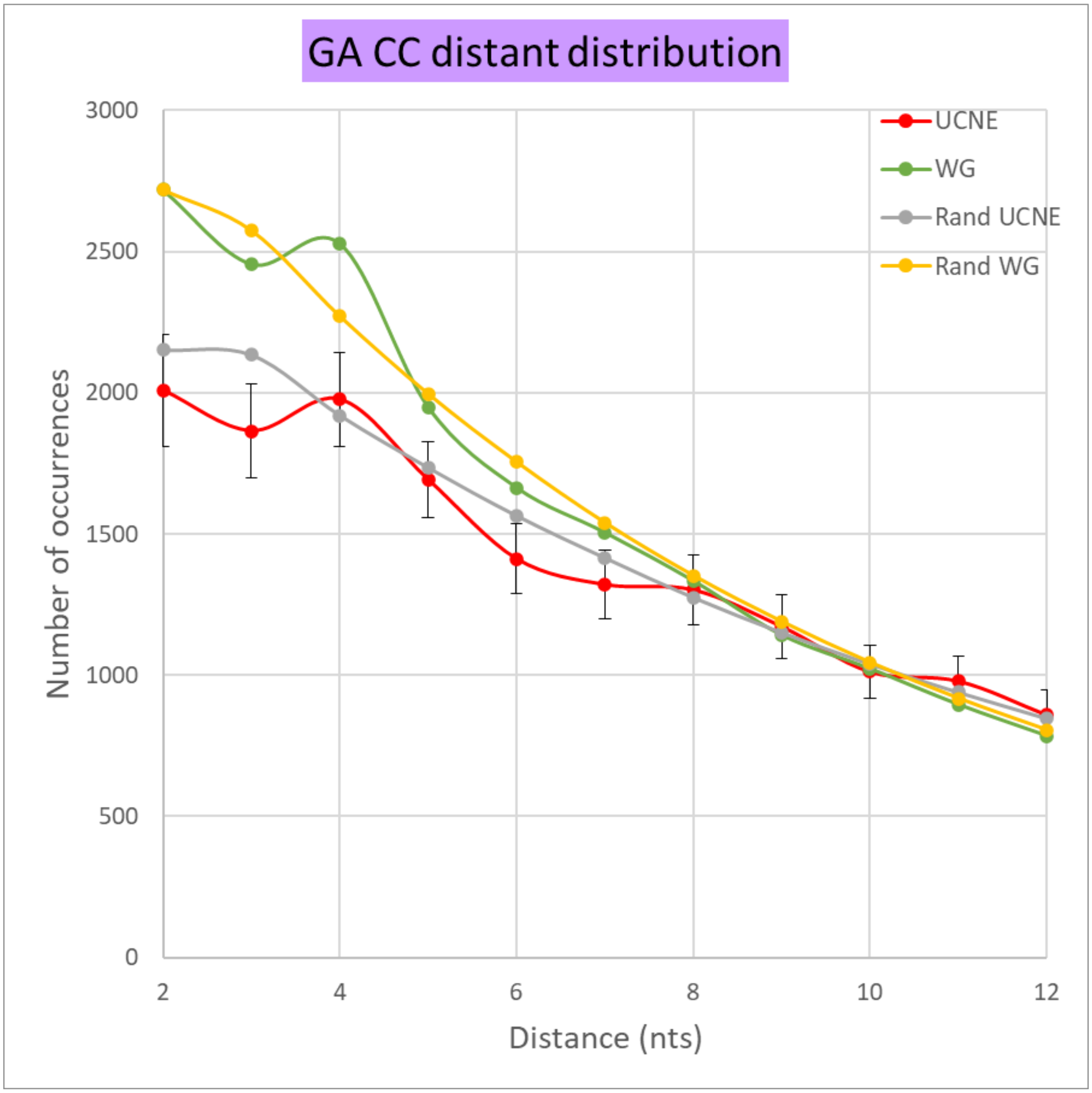

GA CC distant distribution
Number of occurrences
Distance (nts)
UCNE
WG
Rand UCNE
Rand WG
3000
2500
2000
1500
1000
500
0
2
4
6
8
10
12

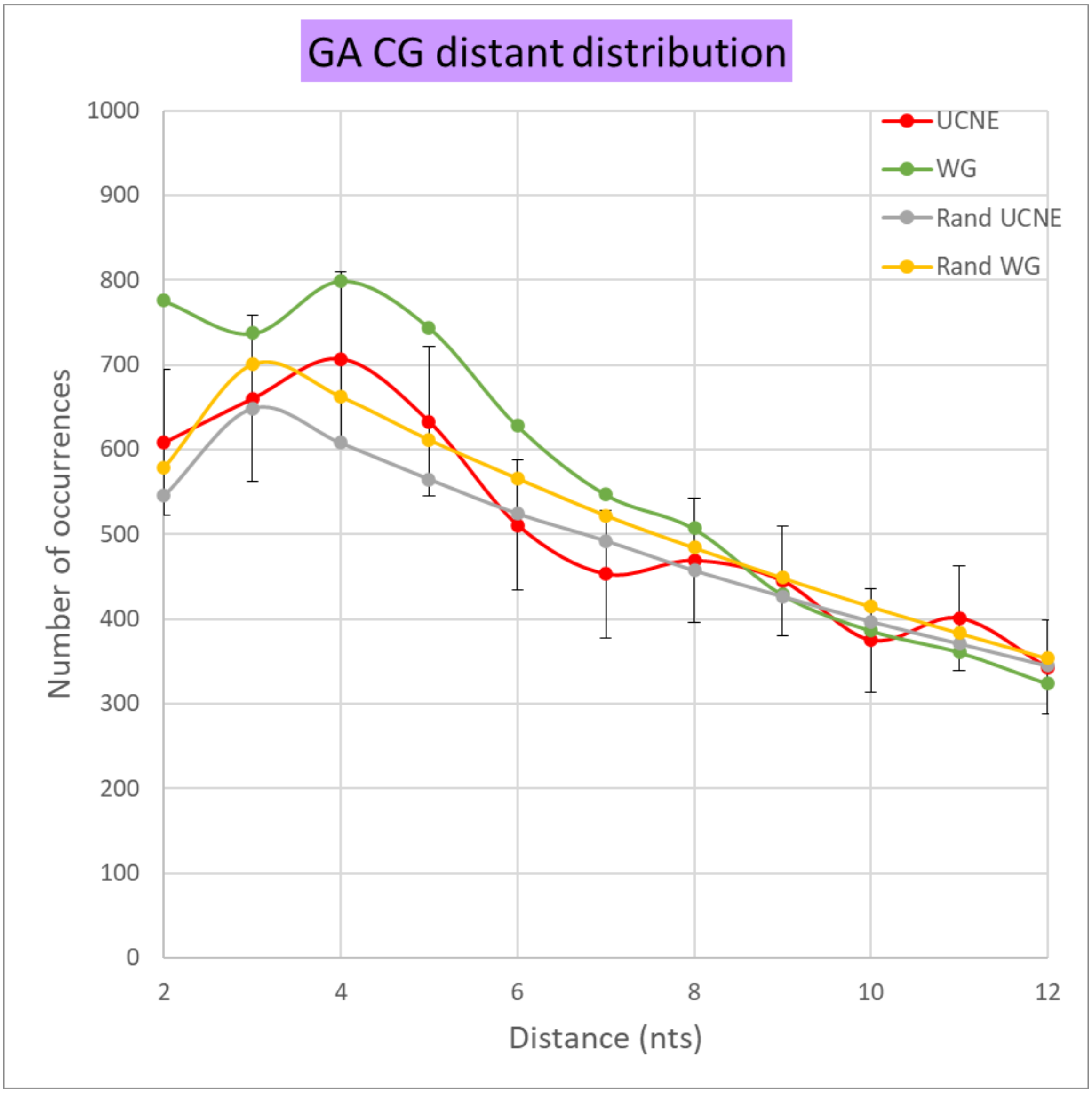
GA CG distant distribution
Number of occurrences
1000
900
800
700
600
500
400
300
200
100
0
2
4
6
8
10
12
Distance (nts)
UCNE
WG
Rand UCNE
Rand WG

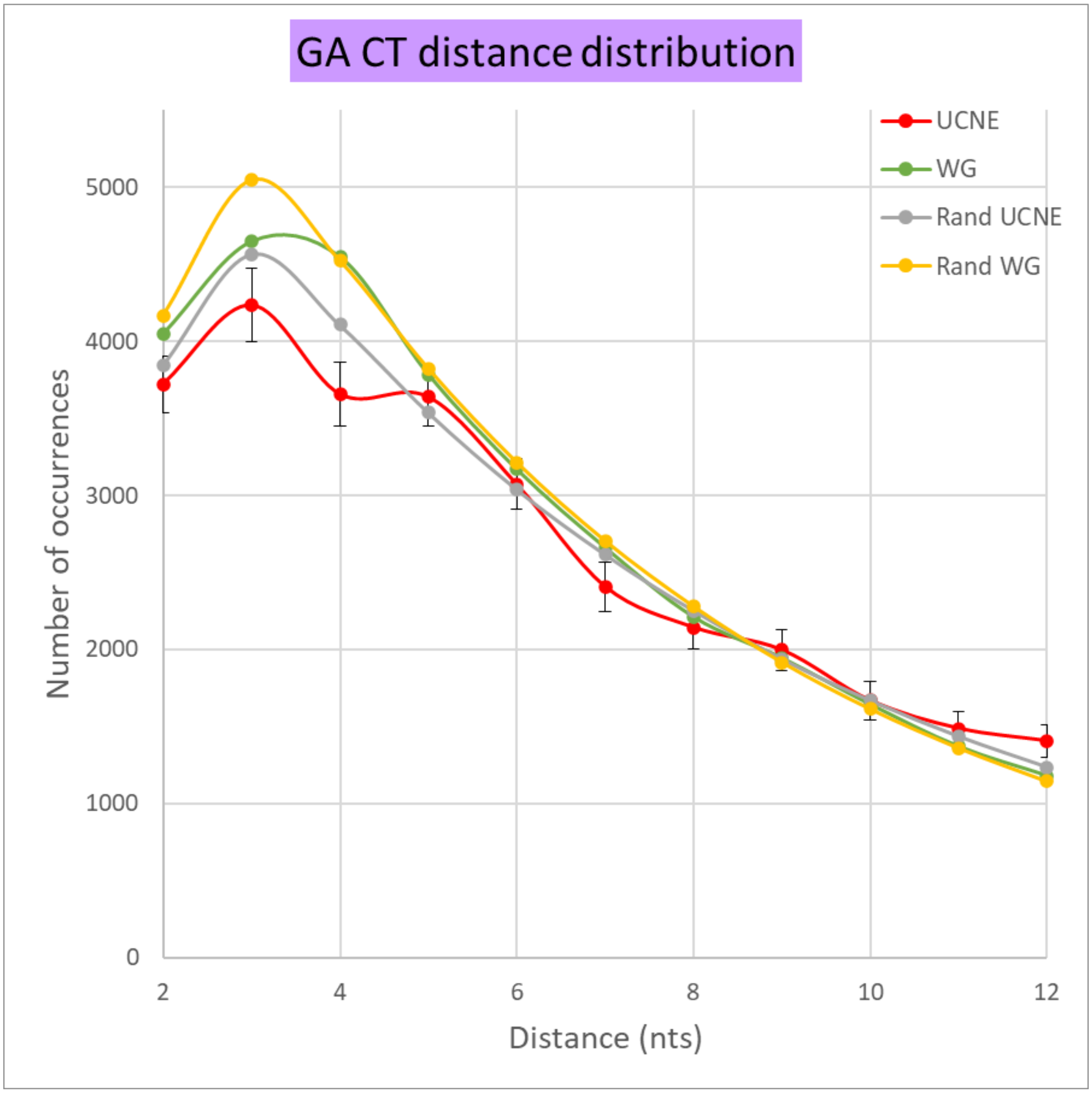

GA CT distance distribution
UCNE
WG
Rand UCNE
Rand WG
Number of occurrences
0
1000
2000
3000
4000
5000
2
4
6
8
10
12
Distance (nts)

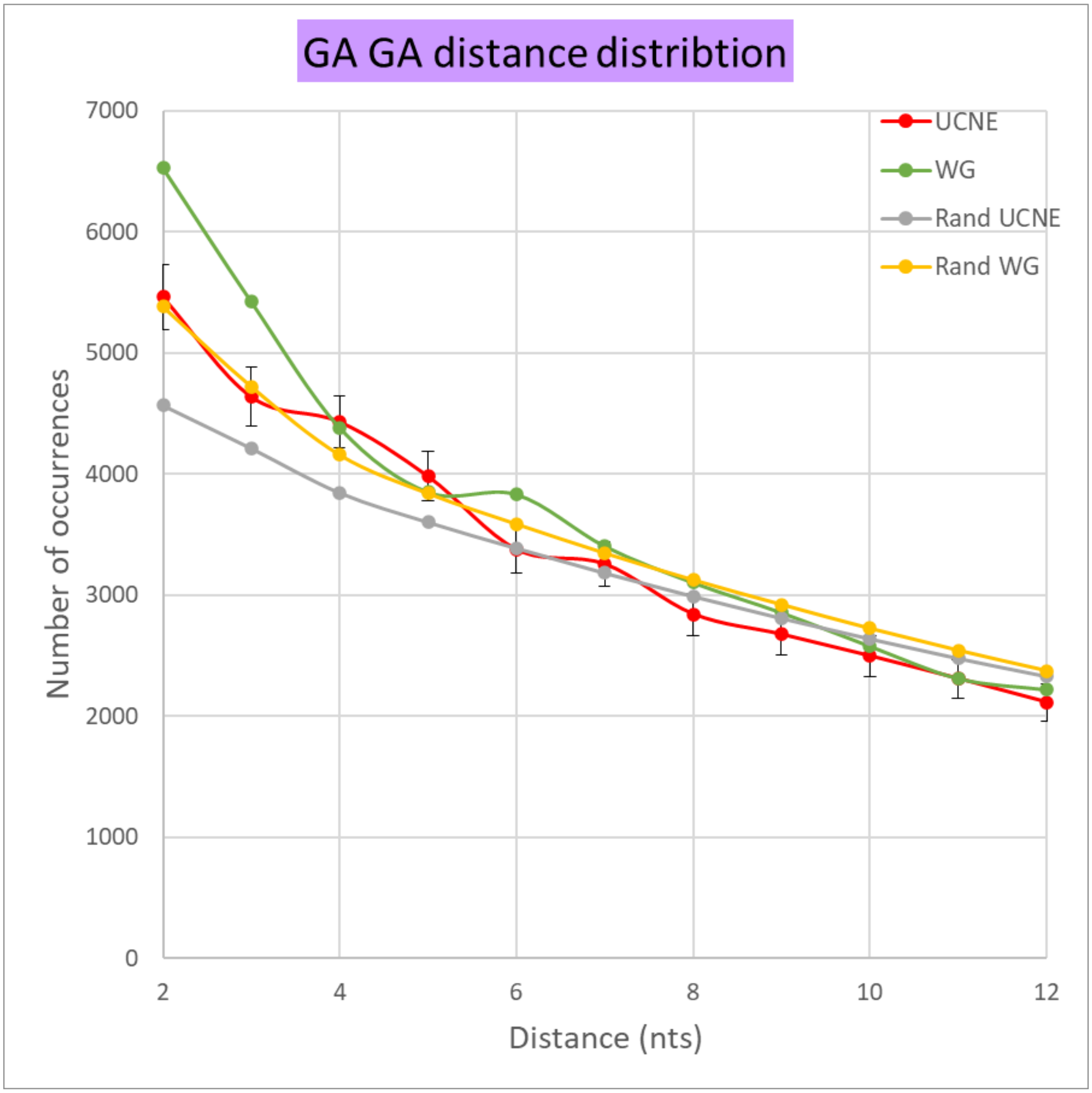
GA GA distance distribtion
UCNE
WG
Rand UCNE
Rand WG
Number of occurrences
Distance (nts)
0
1000
2000
3000
4000
5000
6000
7000
2
4
6
8
10
12

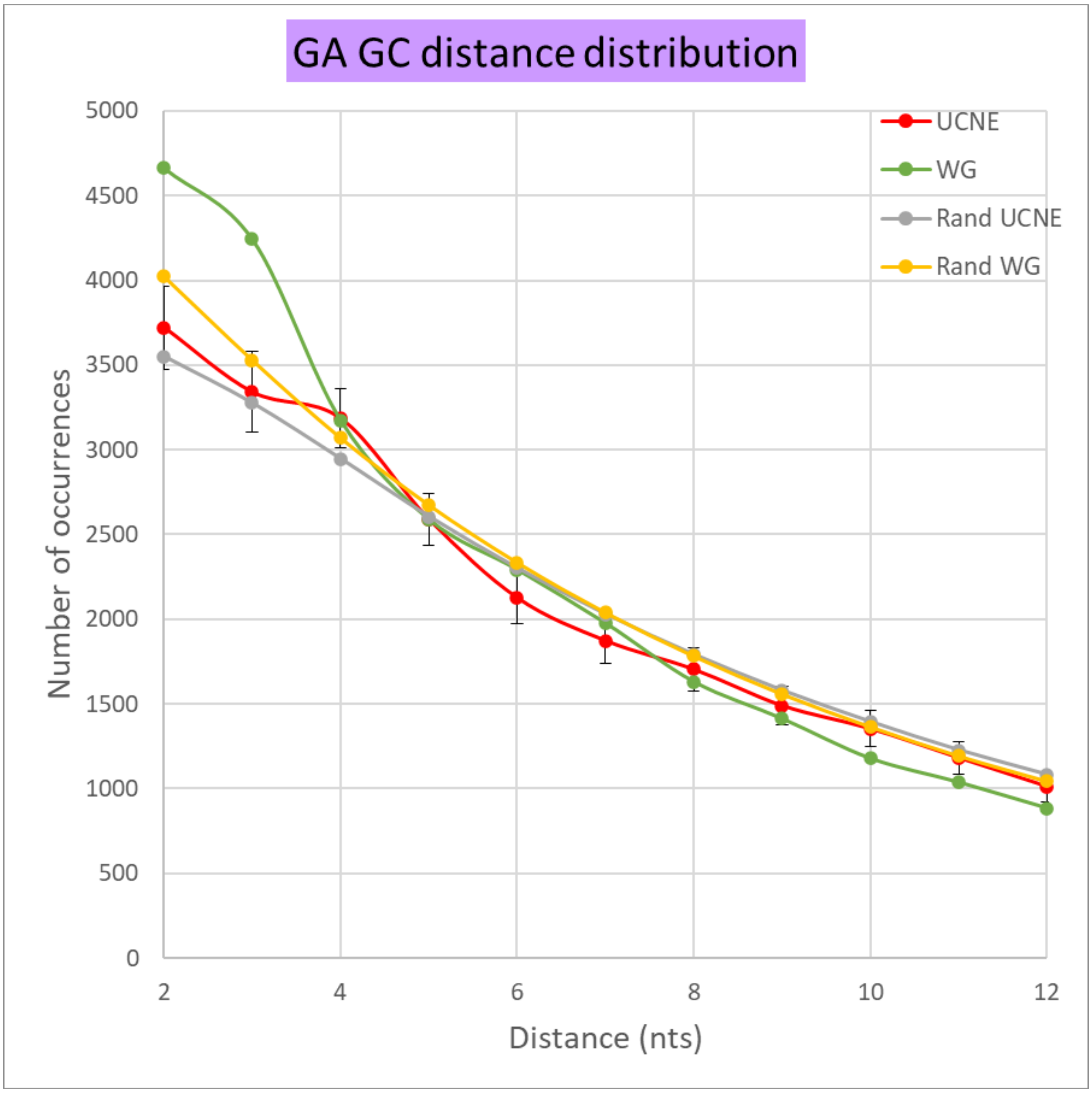
GA GC distance distribution
UCNE
WG
Rand UCNE
Rand WG
Number of occurrences
0
500
1000
1500
2000
2500
3000
3500
4000
4500
5000
2
4
6
8
10
12
Distance (nts)

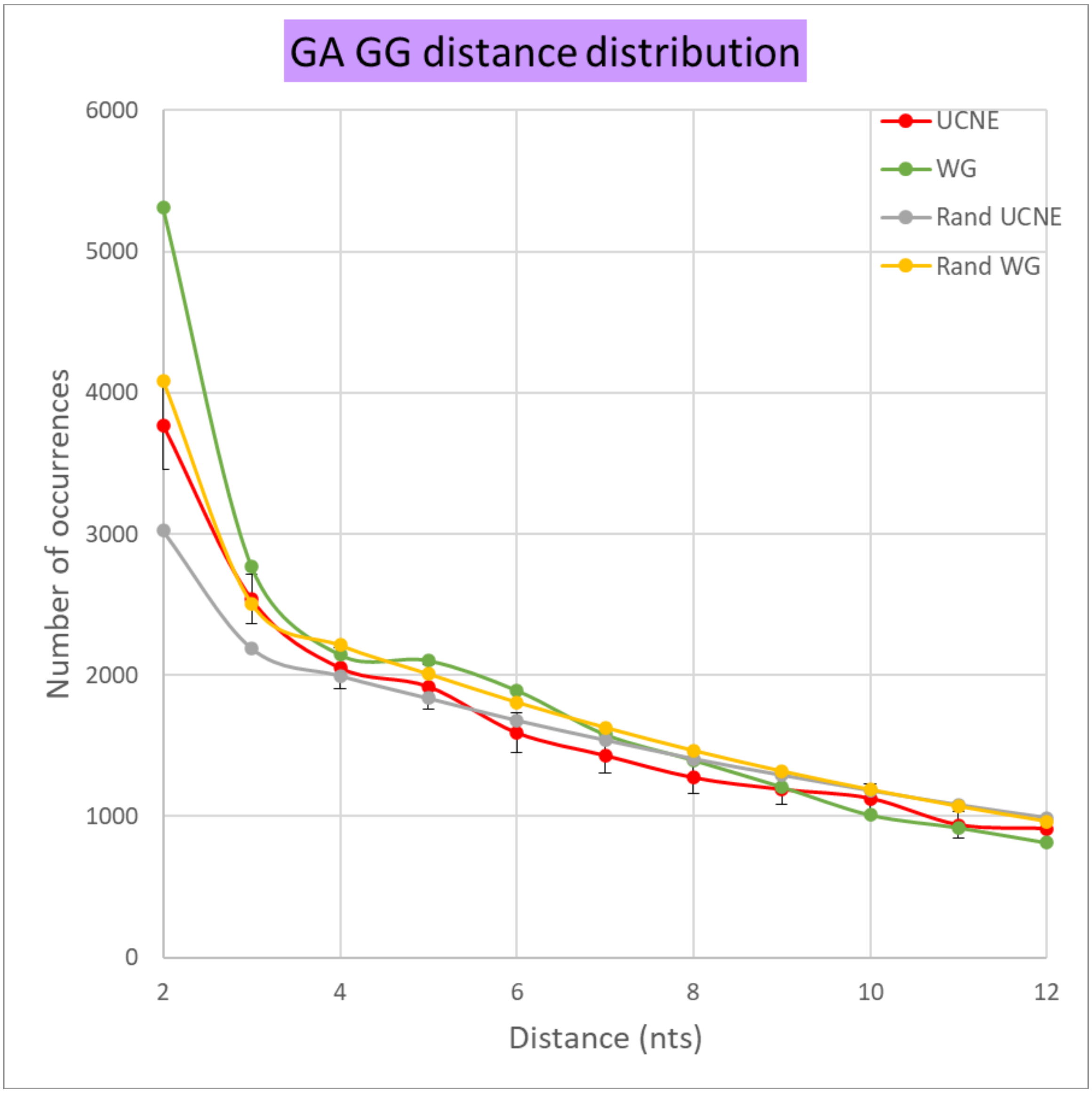

GA GG distance distribution
6000
5000
4000
3000
2000
1000
0
Number of occurrences
2
4
6
8
10
12
Distance (nts)
UCNE
WG
Rand UCNE
Rand WG

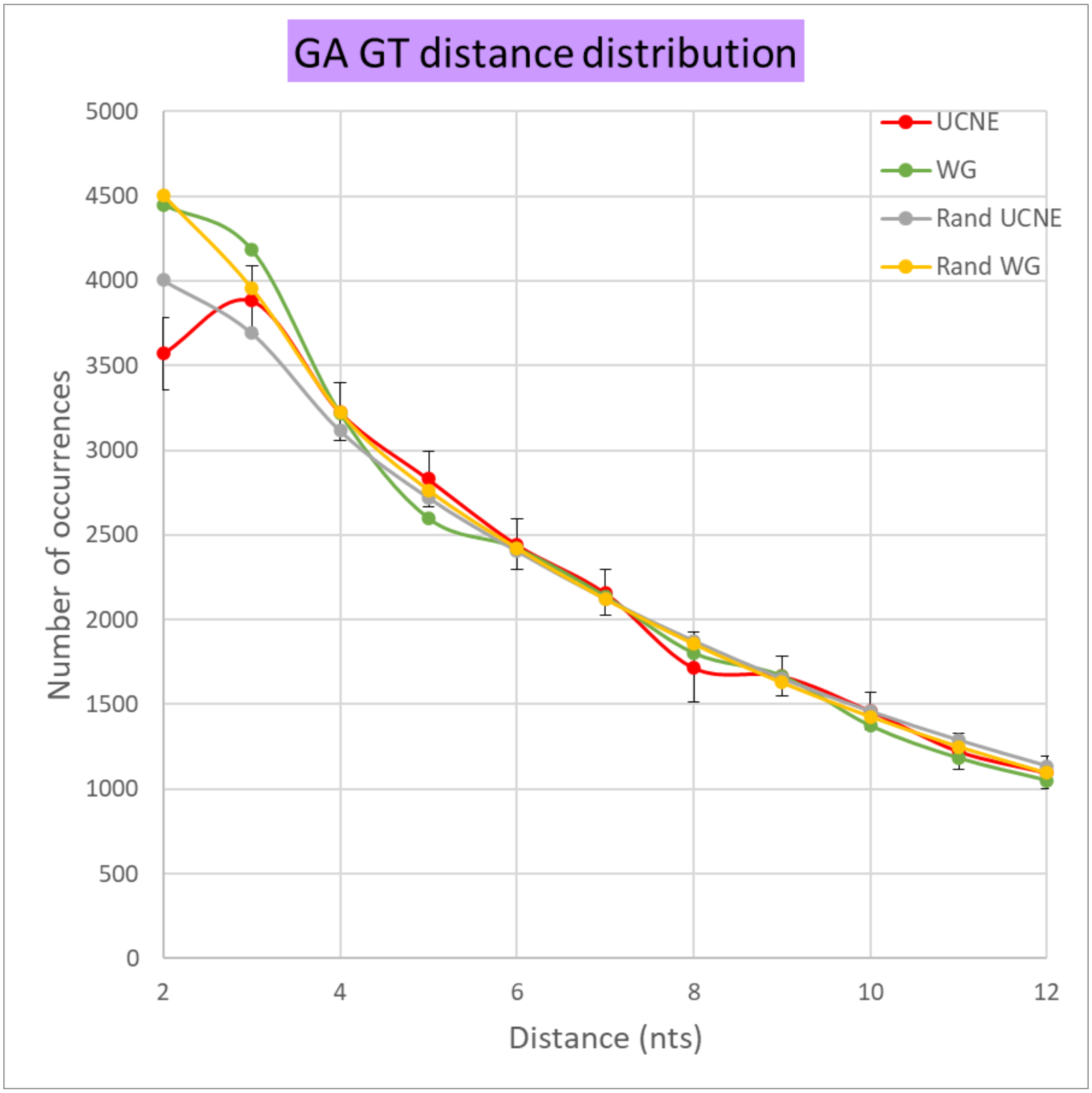

GA GT distance distribution
UCNE
WG
Rand UCNE
Rand WG
Number of occurrences
Distance (nts)
5000
4500
4000
3500
3000
2500
2000
1500
1000
500
0
2
4
6
8
10
12

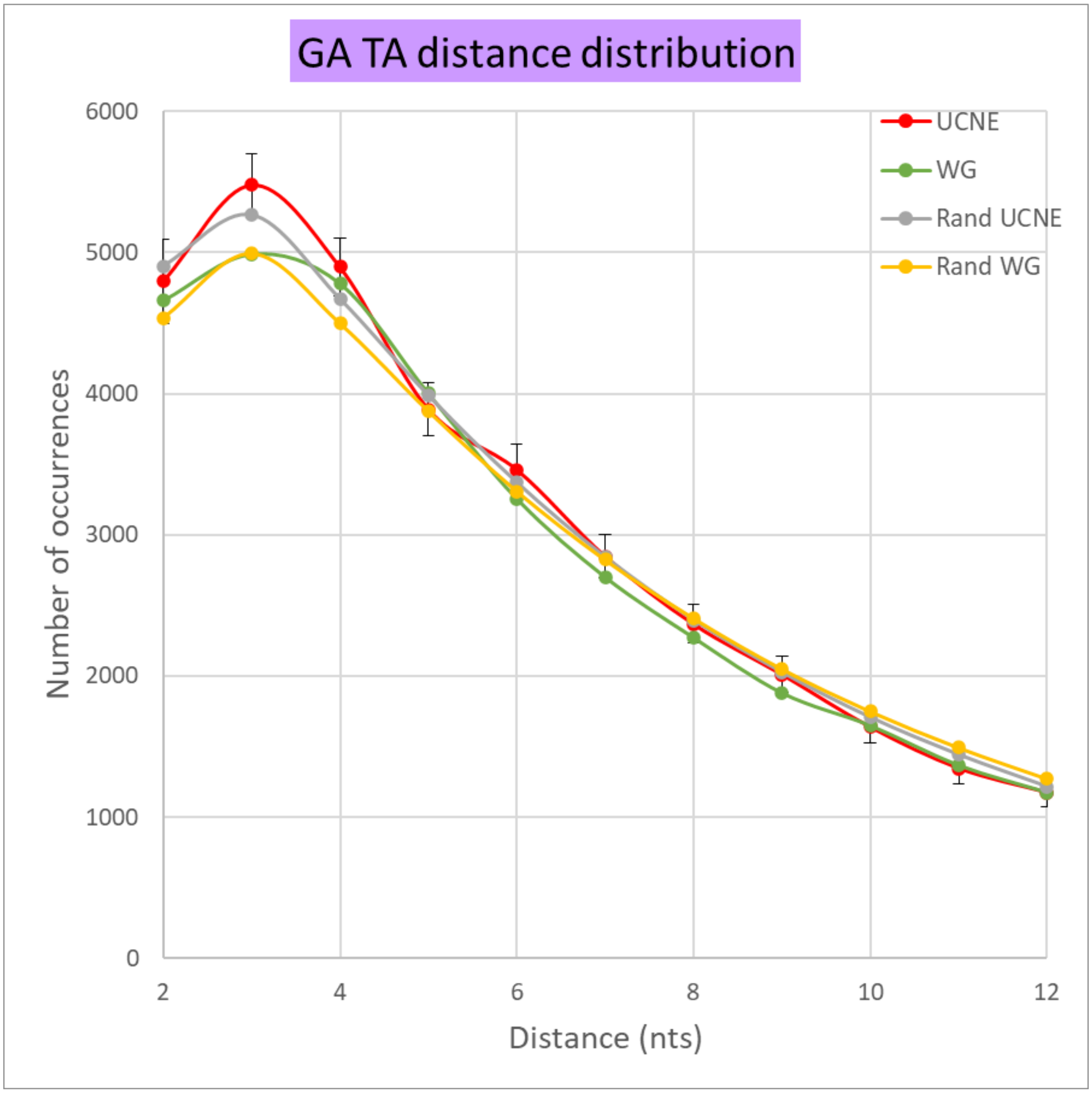
GA TA distance distribution
6000
5000
4000
3000
2000
1000
0
Number of occurrences
2
4
6
8
10
12
Distance (nts)
UCNE
WG
Rand UCNE
Rand WG

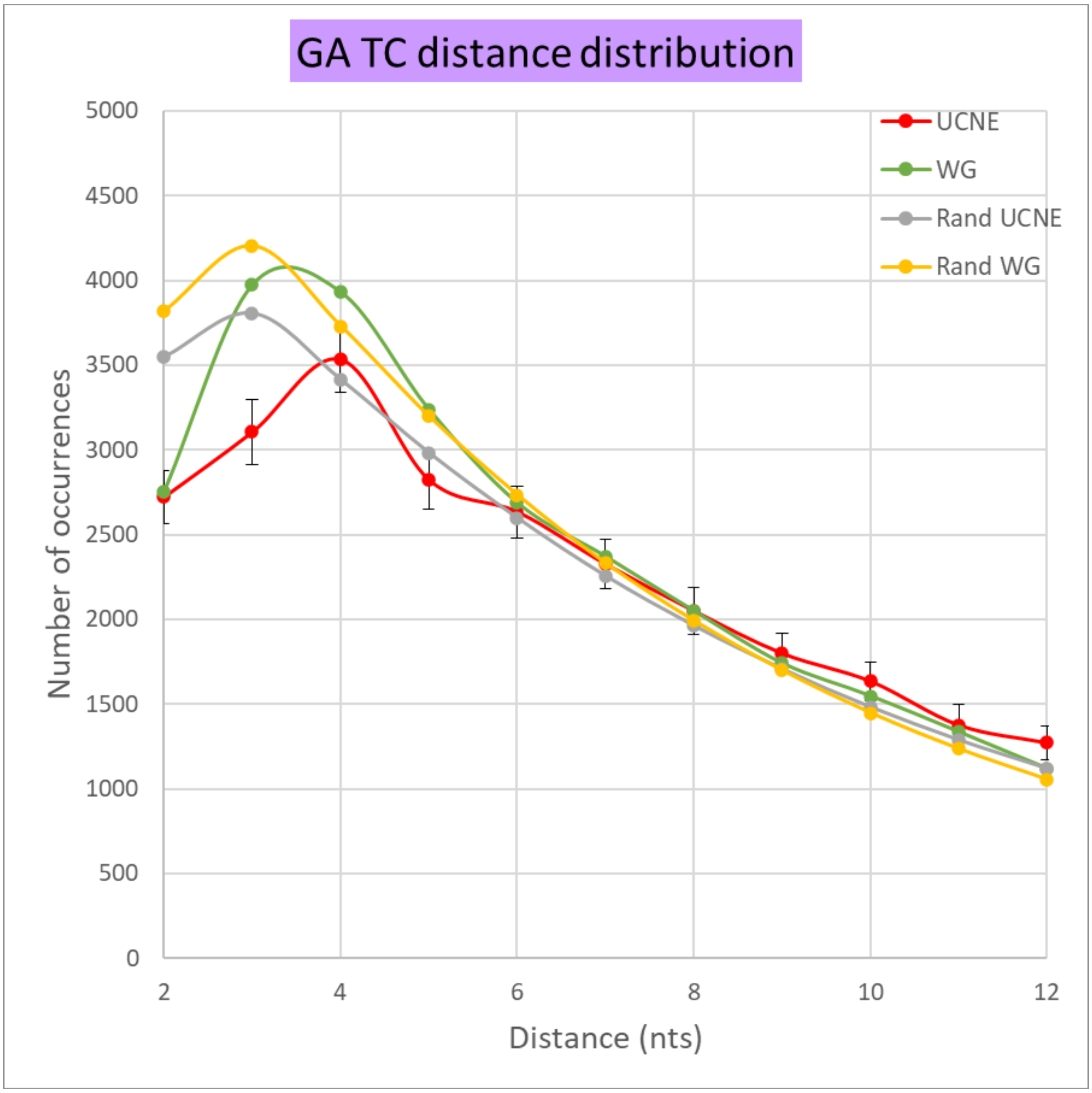

GA TC distance distribution
5000
4500
4000
3500
3000
2500
2000
1500
1000
500
0
Number of occurrences
2
4
6
8
10
12
Distance (nts)
UCNE
WG
Rand UCNE
Rand WG

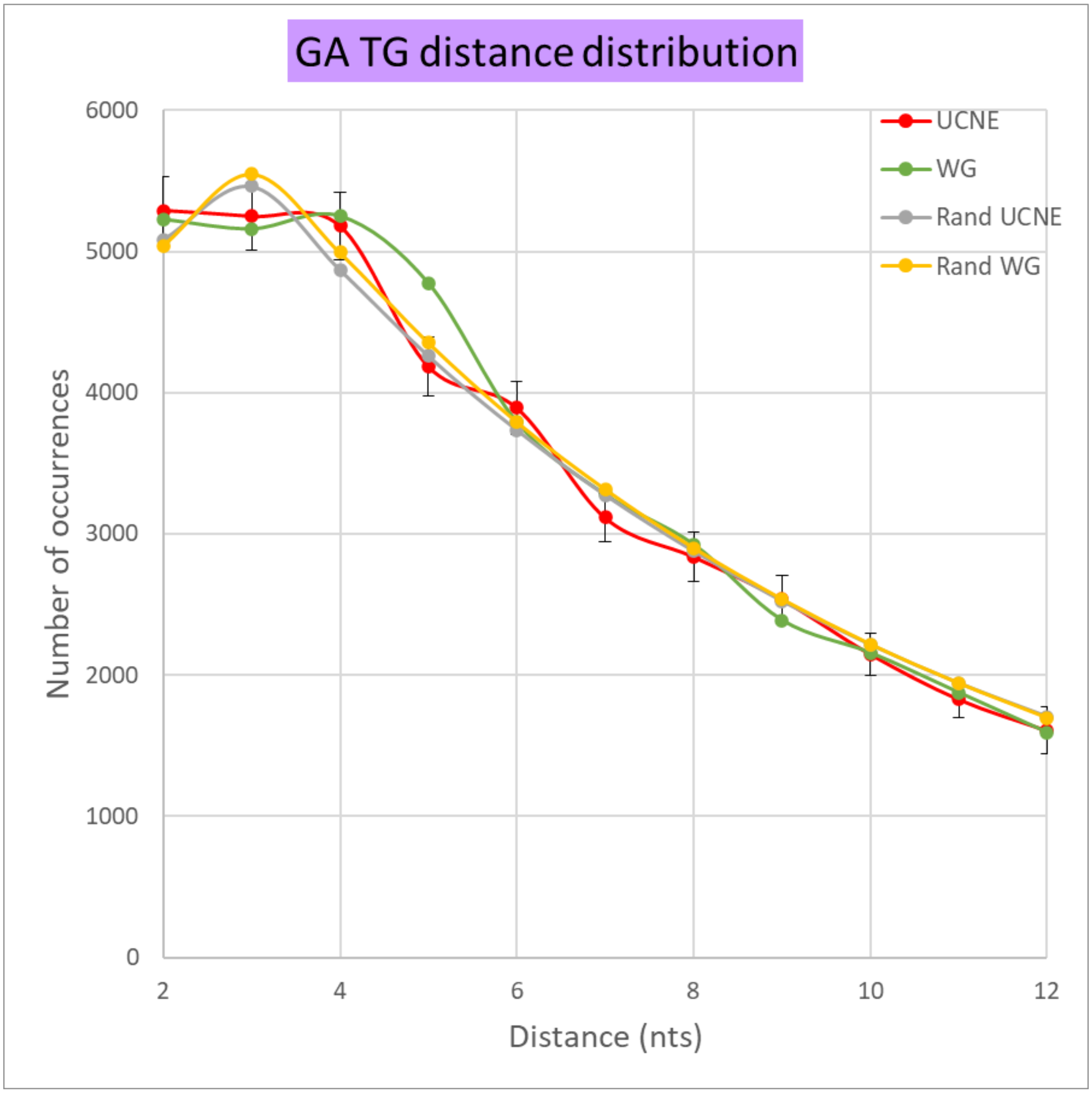

GA TG distance distribution
6000
5000
4000
3000
2000
1000
0
Number of occurrences
2
4
6
8
10
12
Distance (nts)
UCNE
WG
Rand UCNE
Rand WG

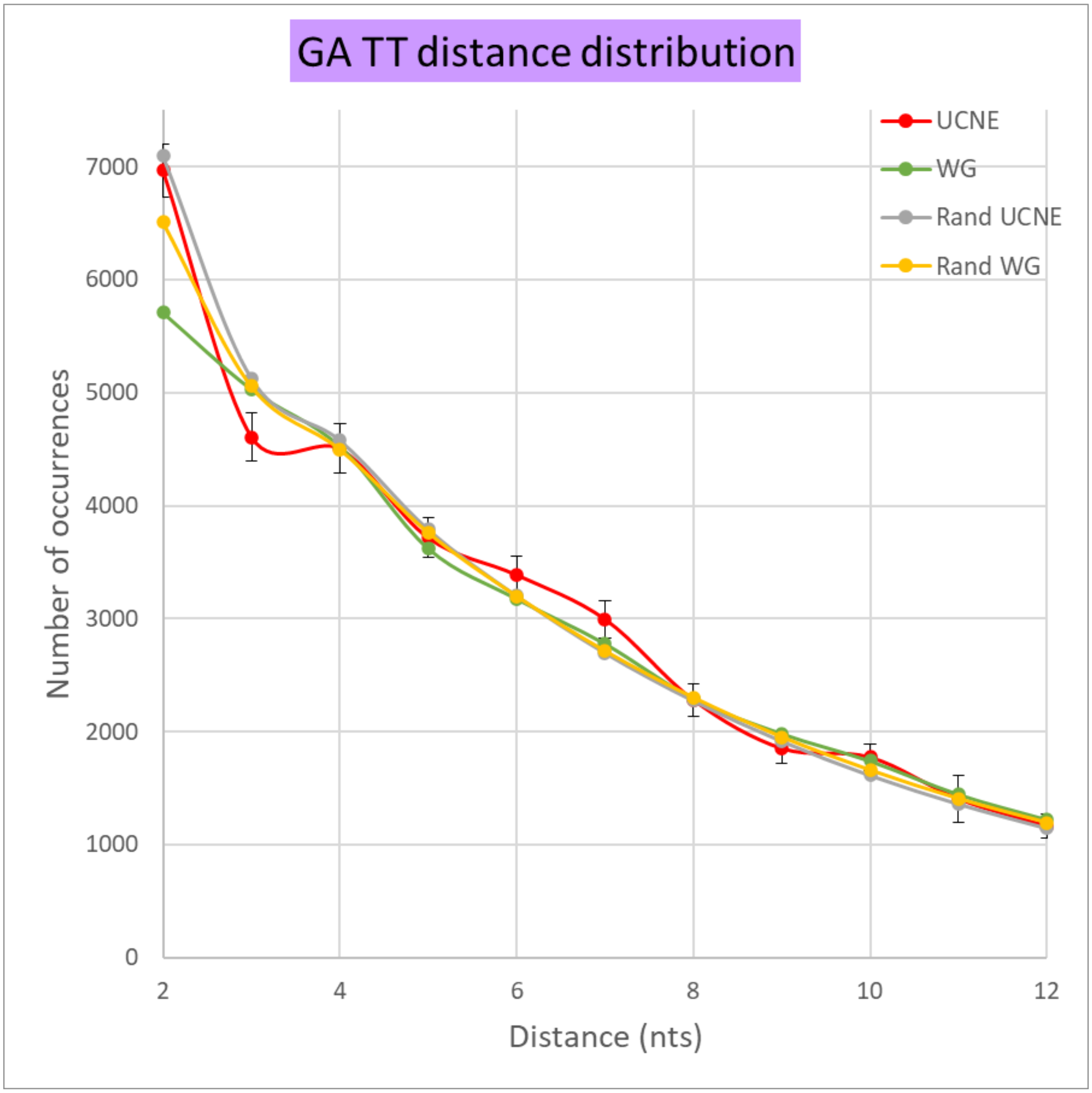

GA TT distance distribution
UCNE
WG
Rand UCNE
Rand WG
Number of occurrences
0
1000
2000
3000
4000
5000
6000
7000
2
4
6
8
10
12
Distance (nts)

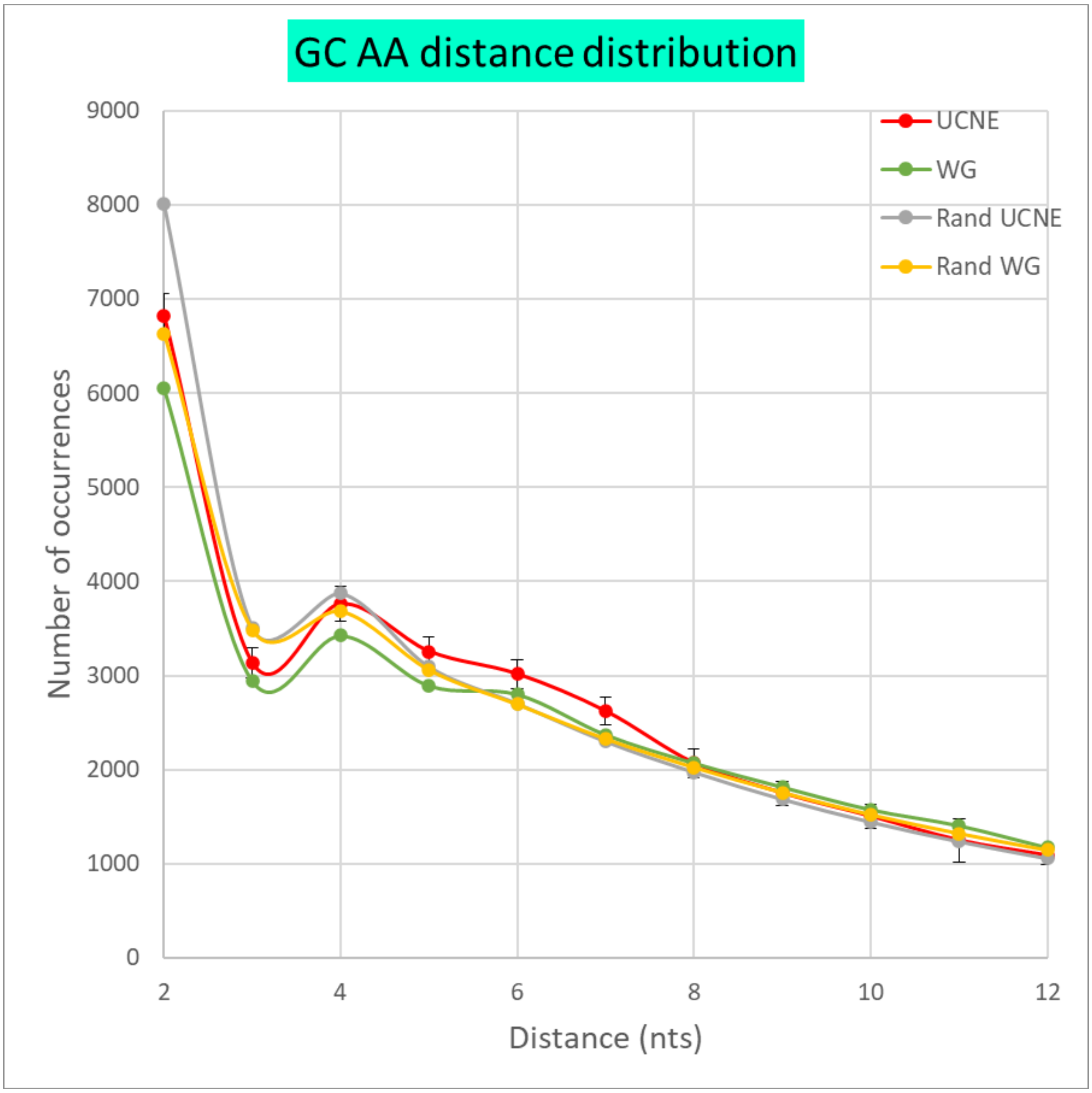
GC AA distance distribution
Number of occurrences
Distance (nts)
UCNE
WG
Rand UCNE
Rand WG
9000
8000
7000
6000
5000
4000
3000
2000
1000
0
2
4
6
8
10
12

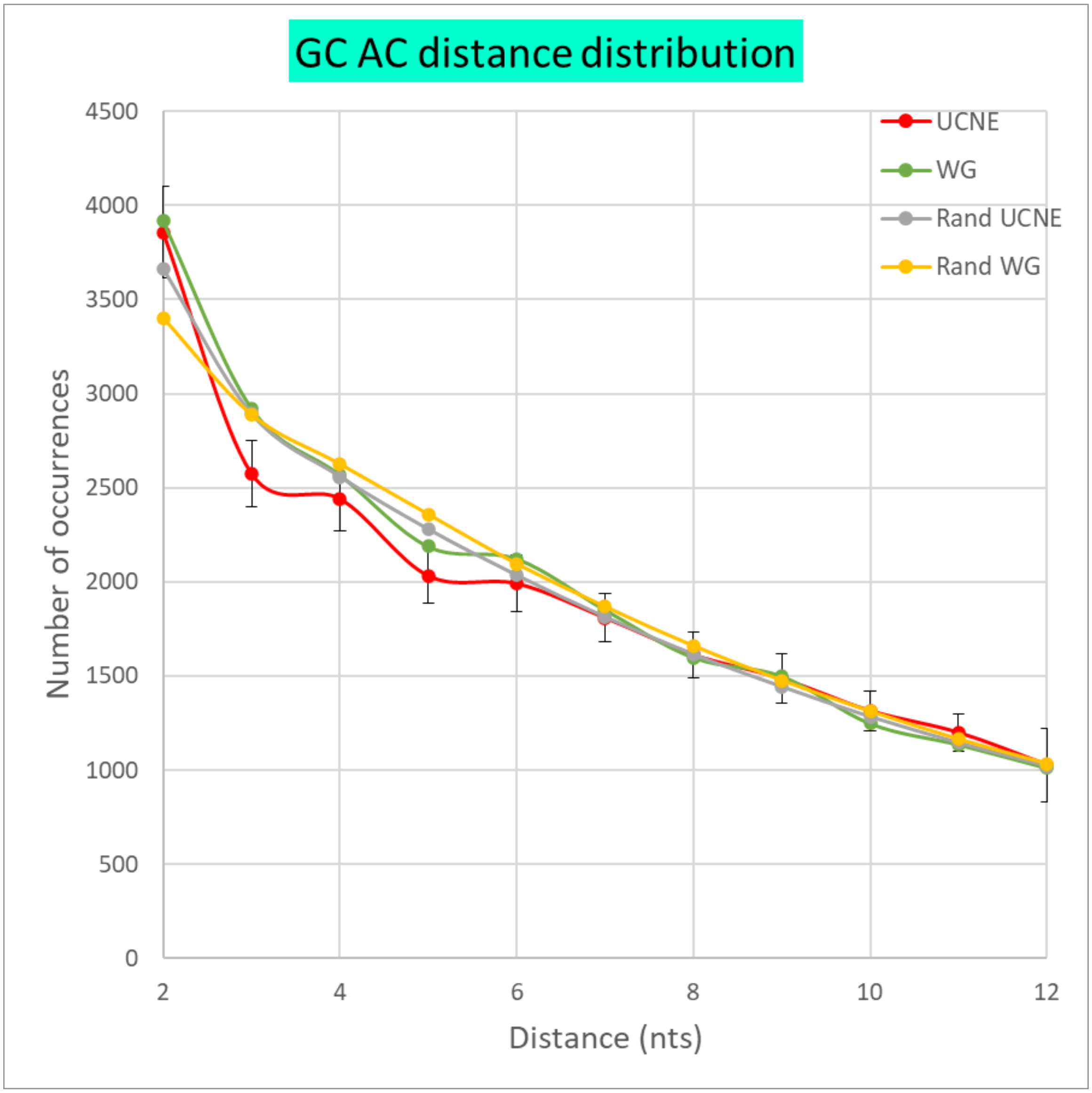
GC AC distance distribution
4500
4000
3500
3000
2500
2000
1500
1000
500
0
Number of occurrences
2
4
6
8
10
12
Distance (nts)
UCNE
WG
Rand UCNE
Rand WG

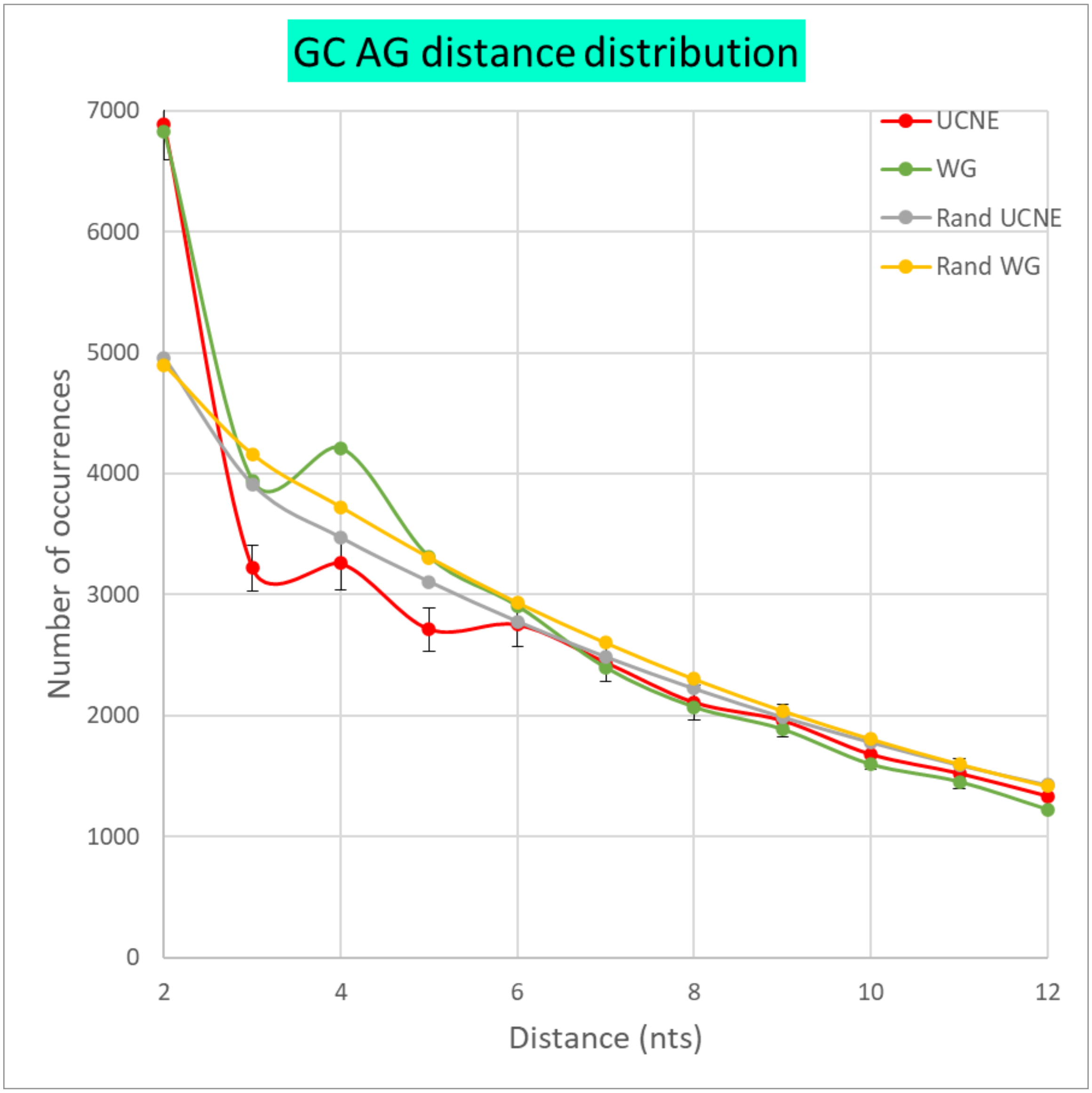
GC AG distance distribution
UCNE
WG
Rand UCNE
Rand WG
Number of occurrences
7000
6000
5000
4000
3000
2000
1000
0
2
4
6
8
10
12
Distance (nts)

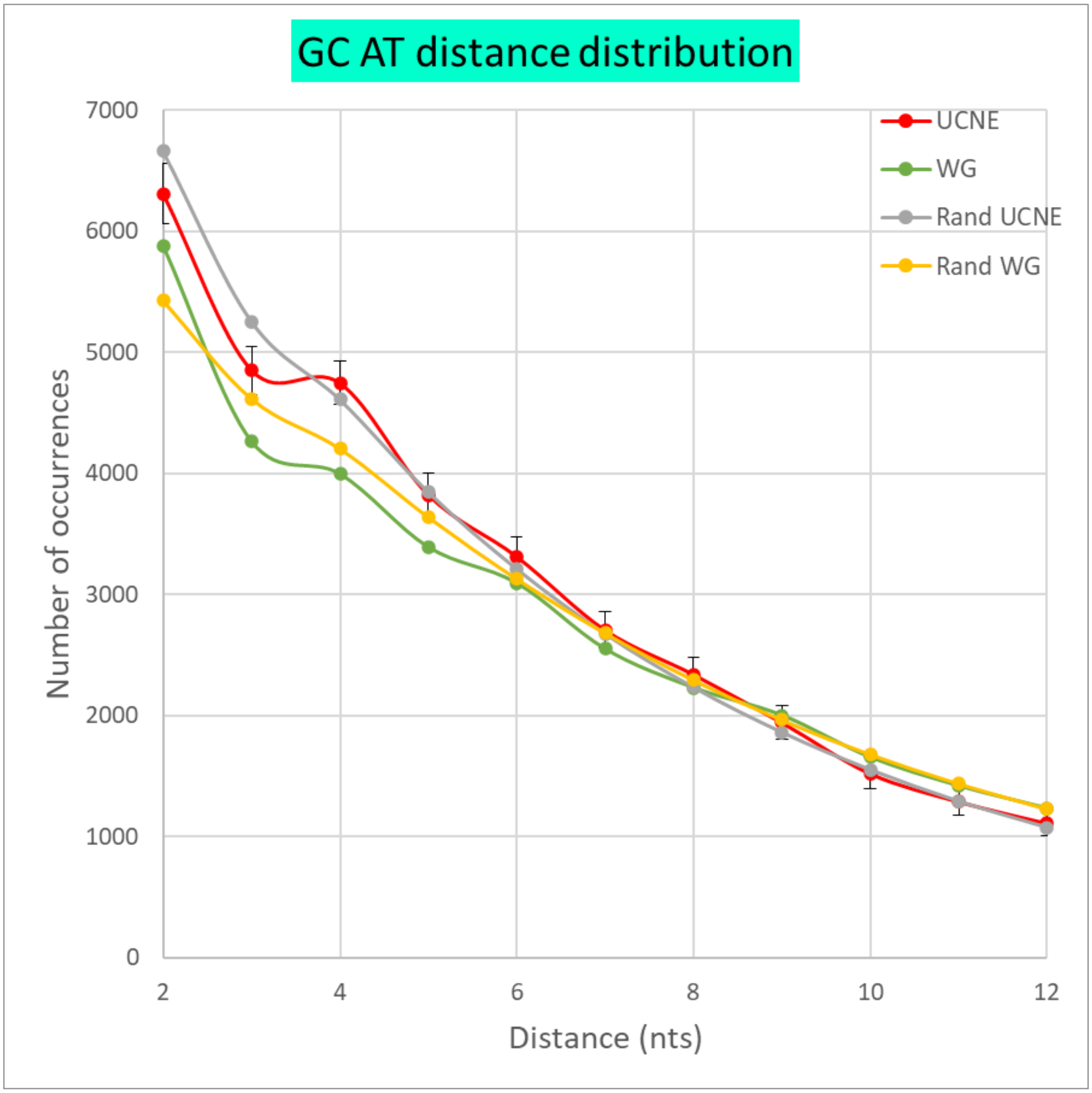
GC AT distance distribution
7000
6000
5000
4000
3000
2000
1000
0
Number of occurrences
2
4
6
8
10
12
Distance (nts)
UCNE
WG
Rand UCNE
Rand WG

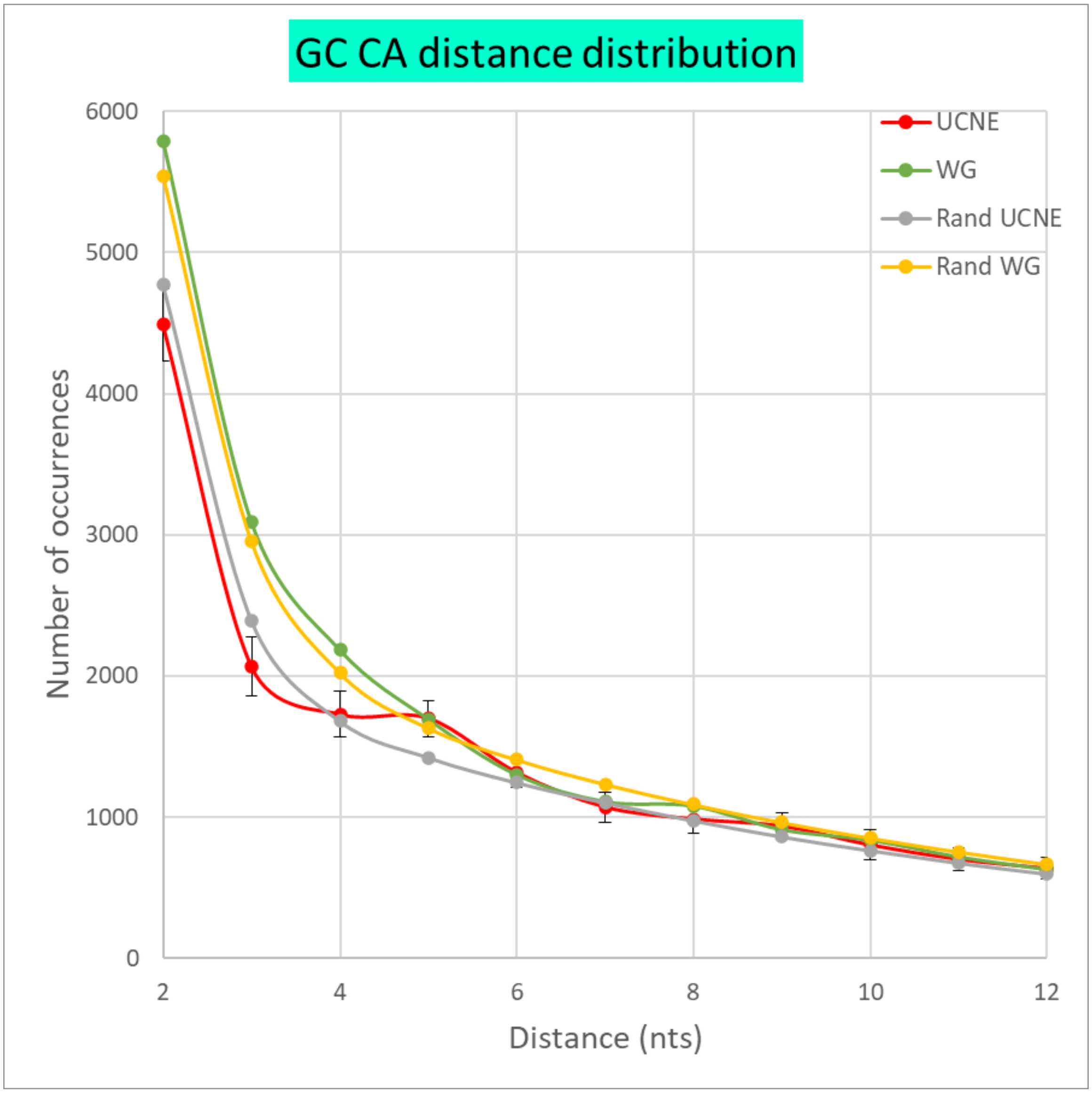
GC CA distance distribution
UCNE
WG
Rand UCNE
Rand WG
Number of occurrences
6000
5000
4000
3000
2000
1000
0
2
4
6
8
10
12
Distance (nts)

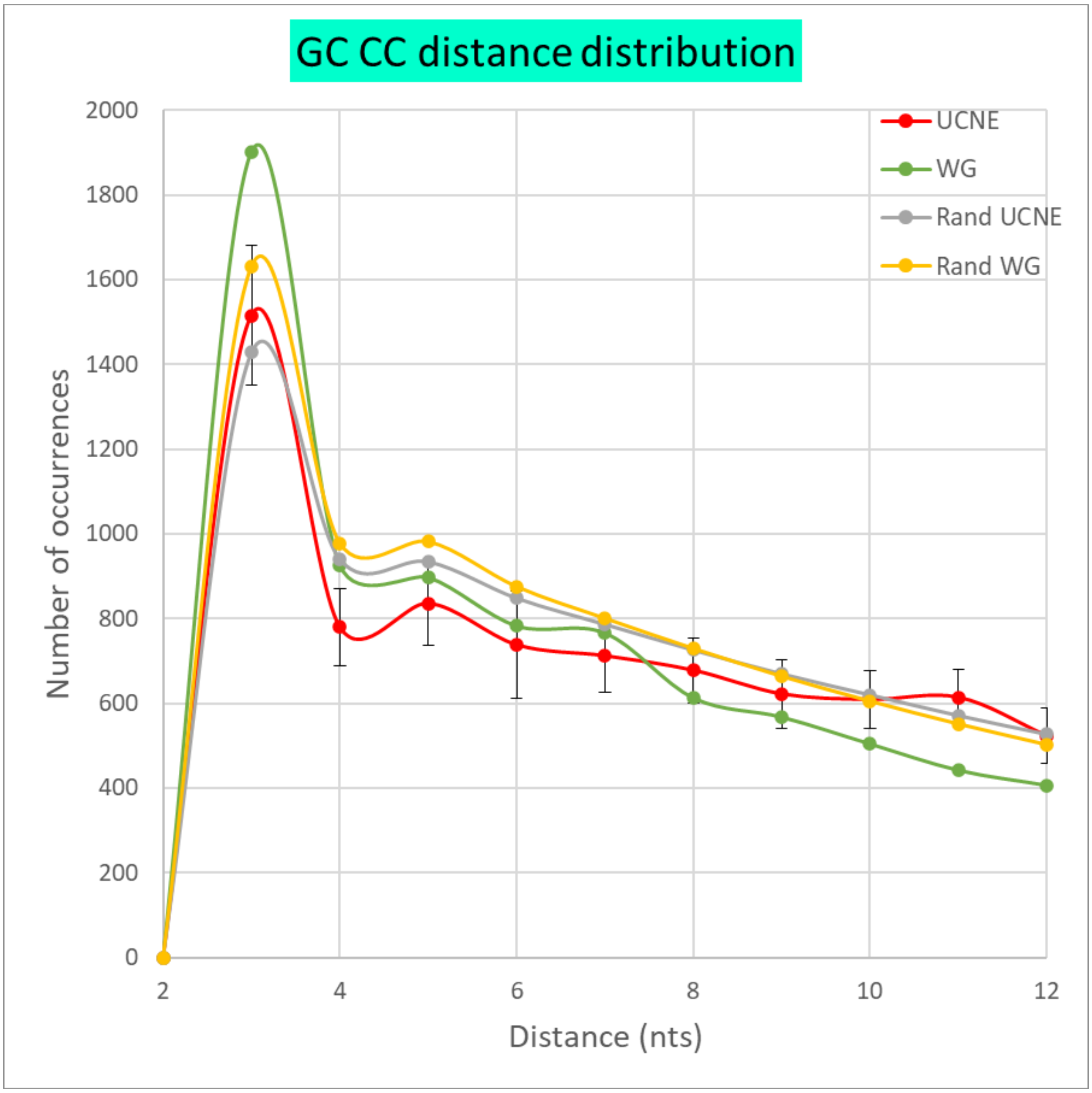
GC CC distance distribution
Number of occurrences
Distance (nts)
UCNE
WG
Rand UCNE
Rand WG
0
200
400
600
800
1000
1200
1400
1600
1800
2000
2
4
6
8
10
12

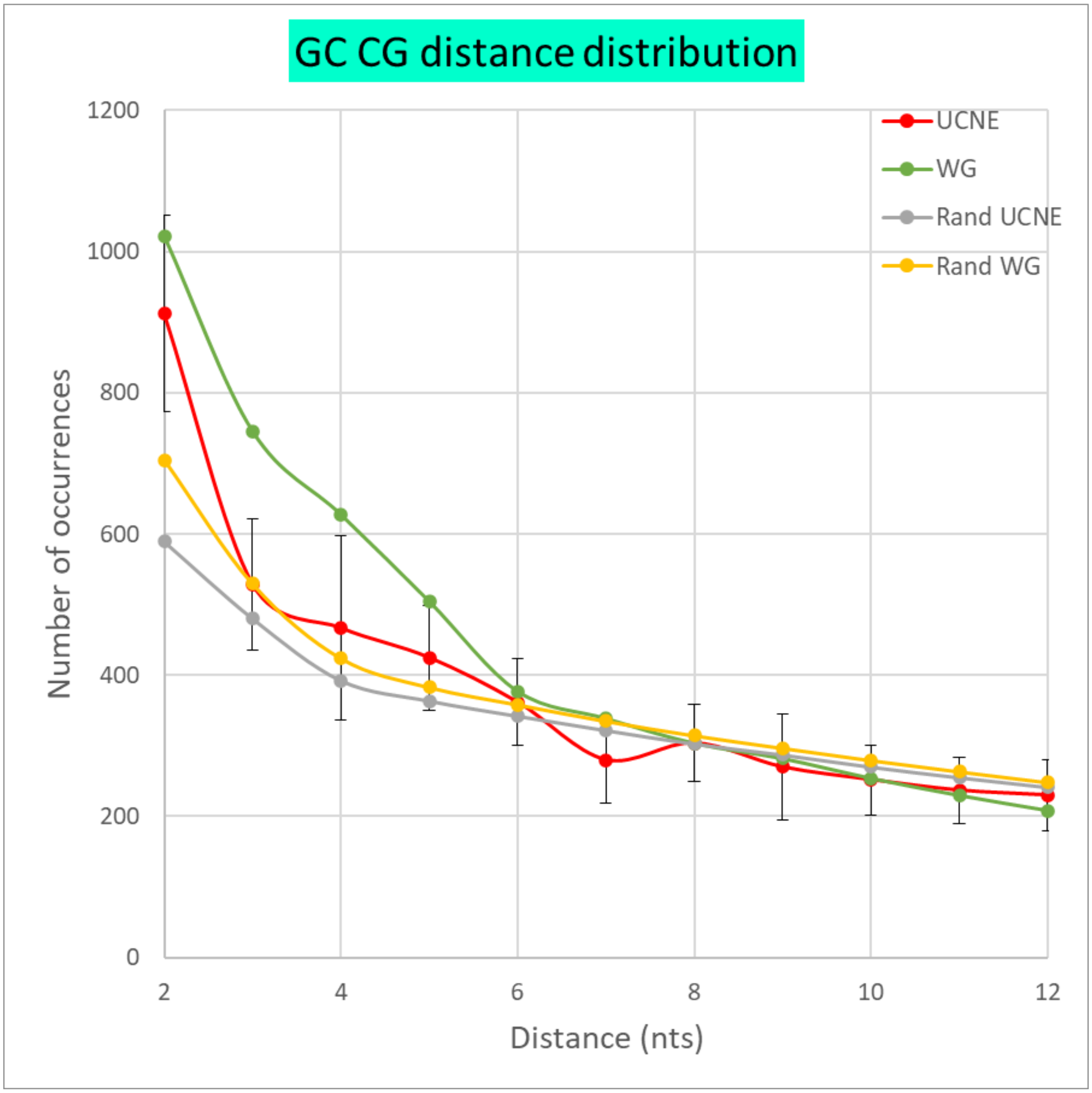
GC CG distance distribution
Number of occurrences
0
200
400
600
800
1000
1200
2
4
6
8
10
12
Distance (nts)
UCNE
WG
Rand UCNE
Rand WG

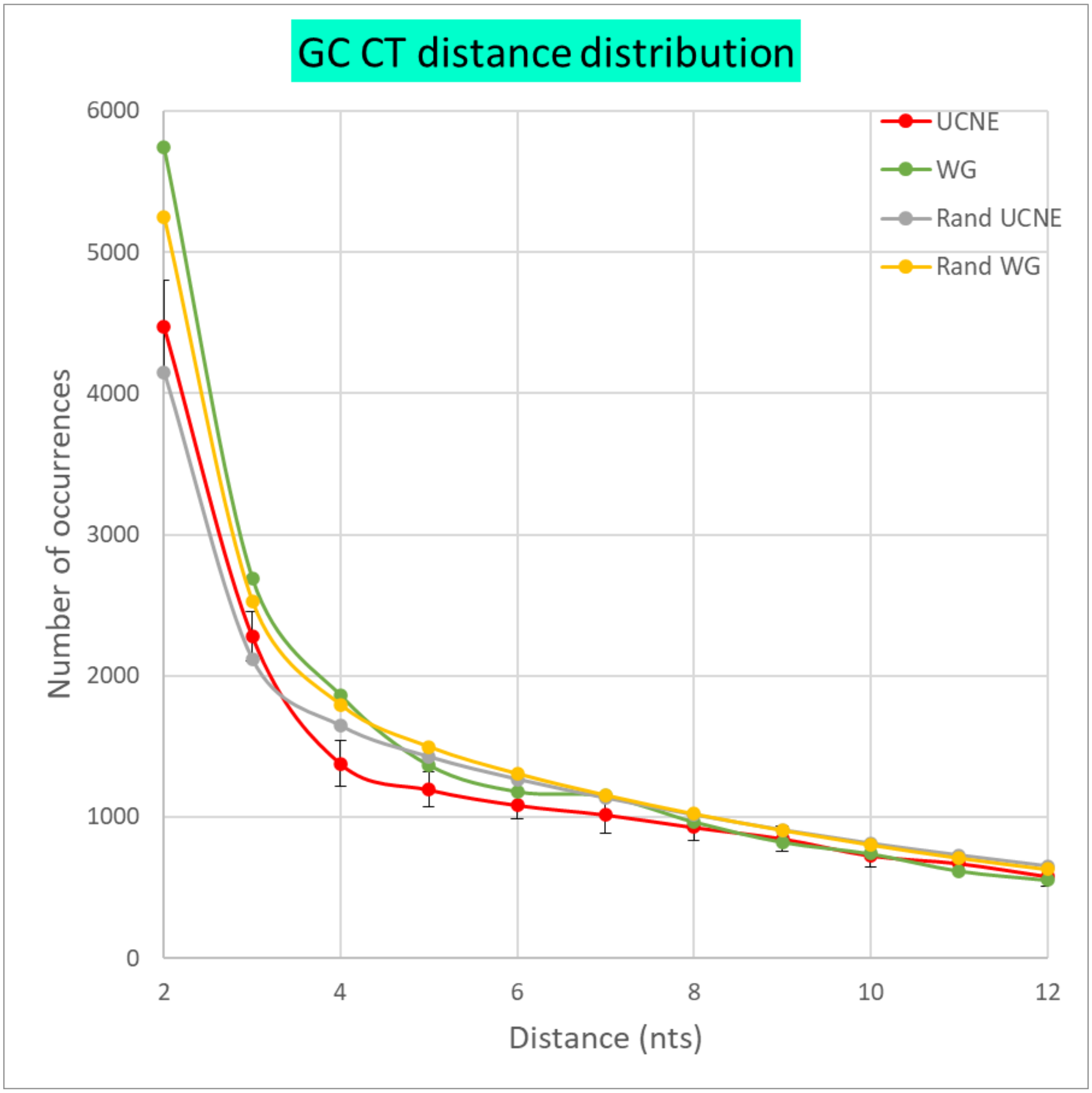
GC CT distance distribution
Number of occurrences
6000
5000
4000
3000
2000
1000
0
2
4
6
8
10
12
Distance (nts)
UCNE
WG
Rand UCNE
Rand WG

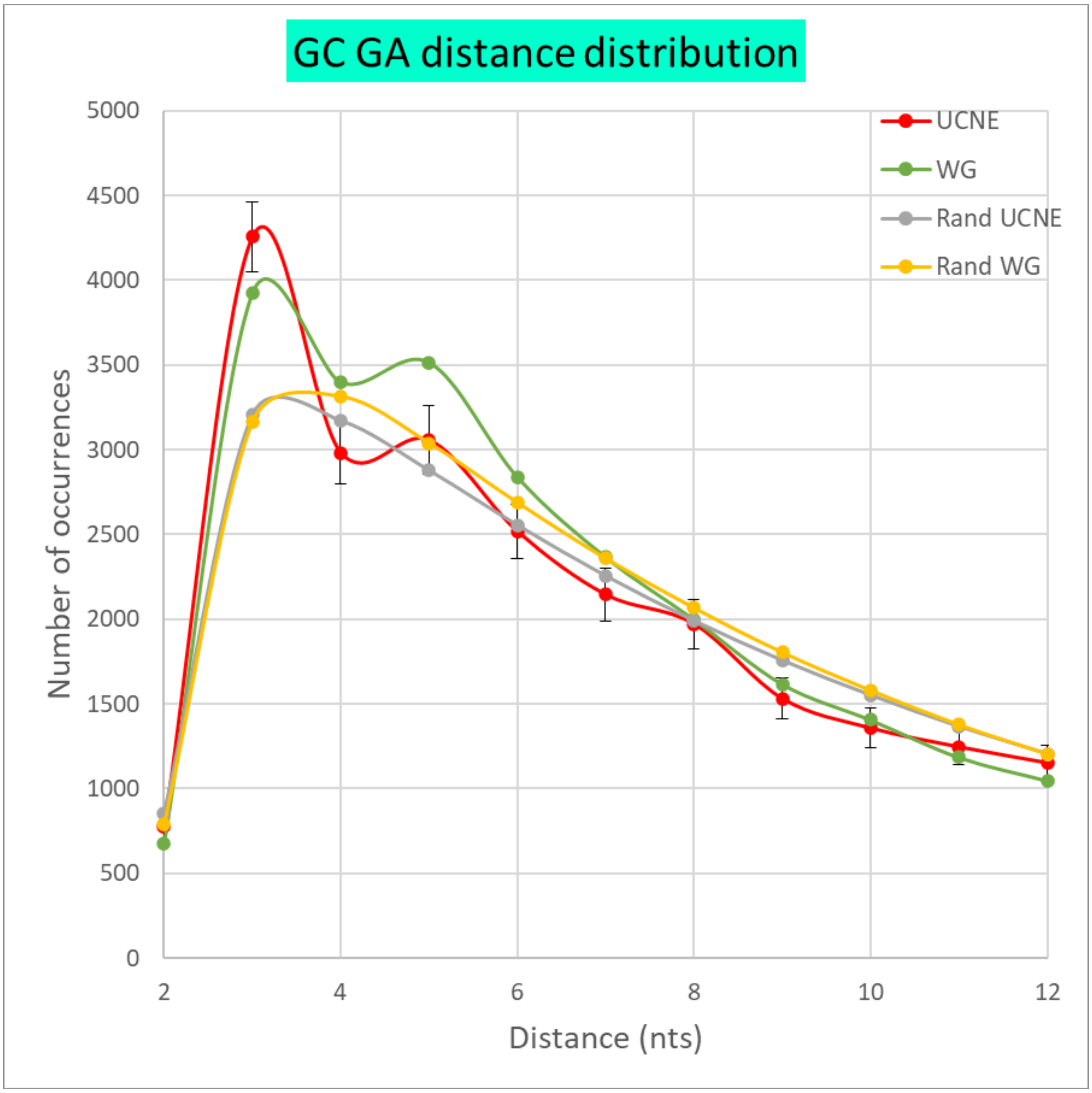
GC GA distance distribution
5000
4500
4000
3500
3000
2500
2000
1500
1000
500
0
2
4
6
8
10
12
Number of occurrences
Distance (nts)
UCNE
WG
Rand UCNE
Rand WG

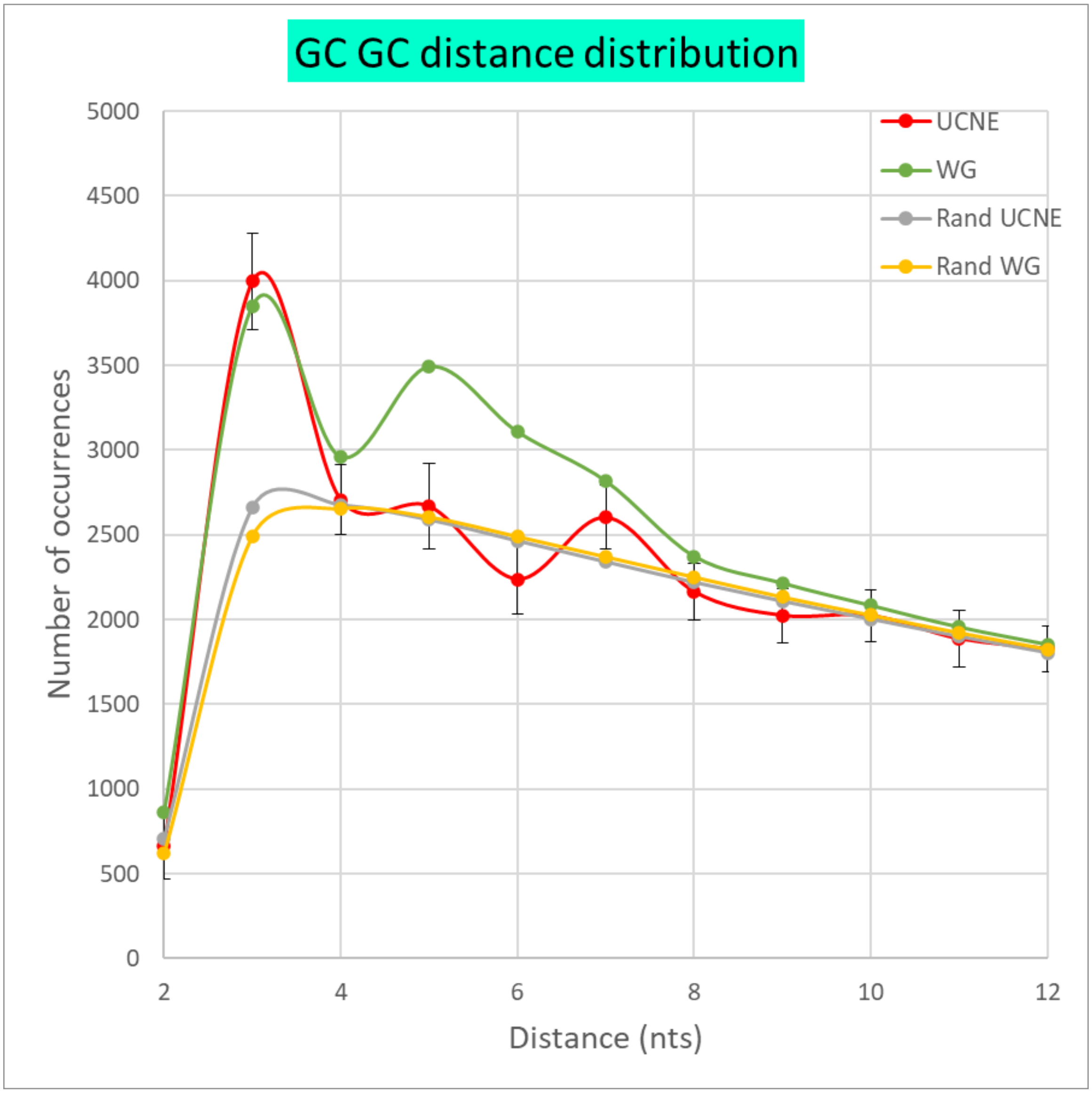
GC GC distance distribution
Number of occurrences
5000
4500
4000
3500
3000
2500
2000
1500
1000
500
0
2
4
6
8
10
12
Distance (nts)
UCNE
WG
Rand UCNE
Rand WG

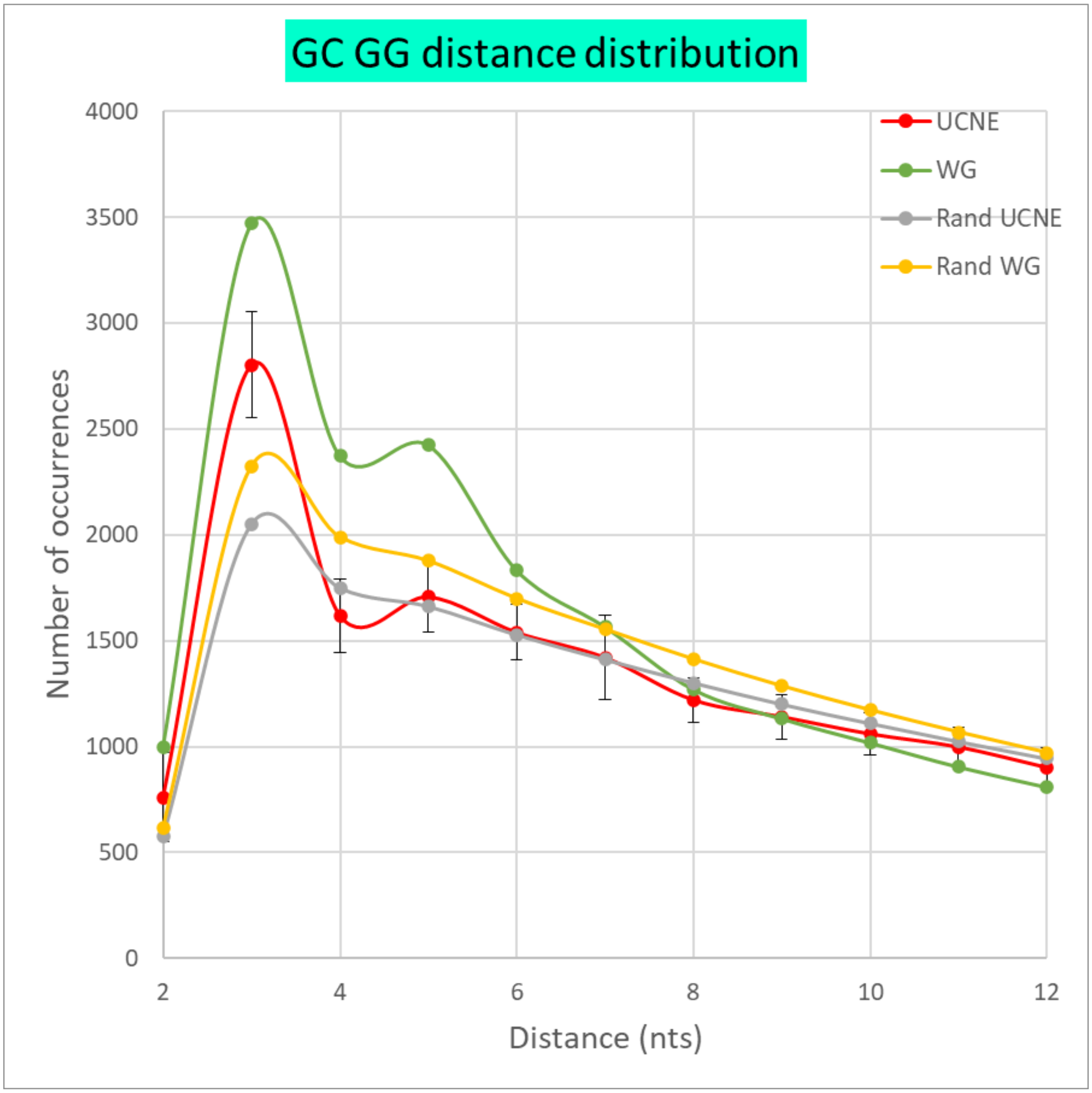
GC GG distance distribution
UCNE
WG
Rand UCNE
Rand WG
Number of occurrences
0
500
1000
1500
2000
2500
3000
3500
4000
2
4
6
8
10
12
Distance (nts)

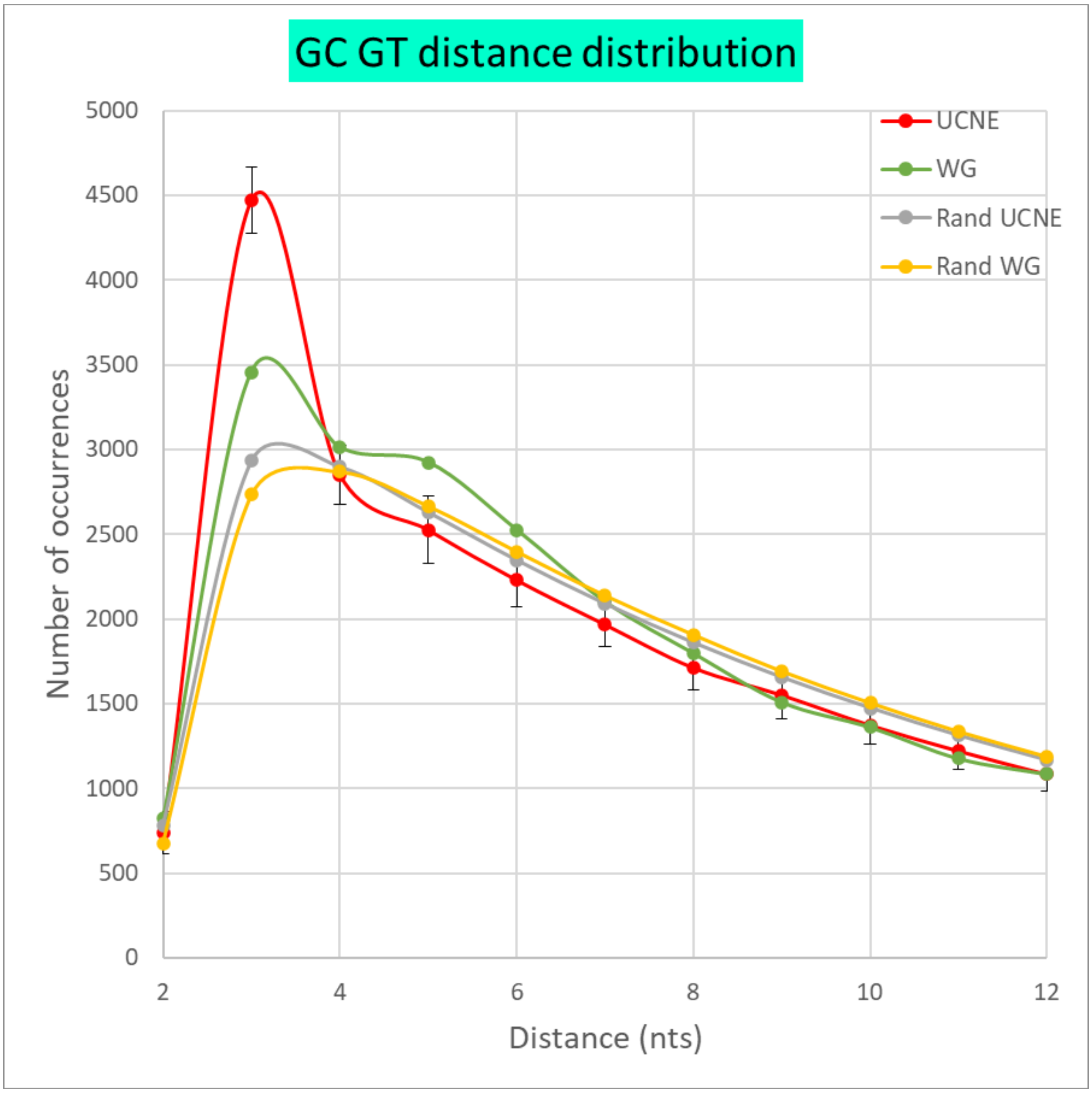
GC GT distance distribution
Number of occurrences
Distance (nts)
0
500
1000
1500
2000
2500
3000
3500
4000
4500
5000
2
4
6
8
10
12
UCNE
WG
Rand UCNE
Rand WG

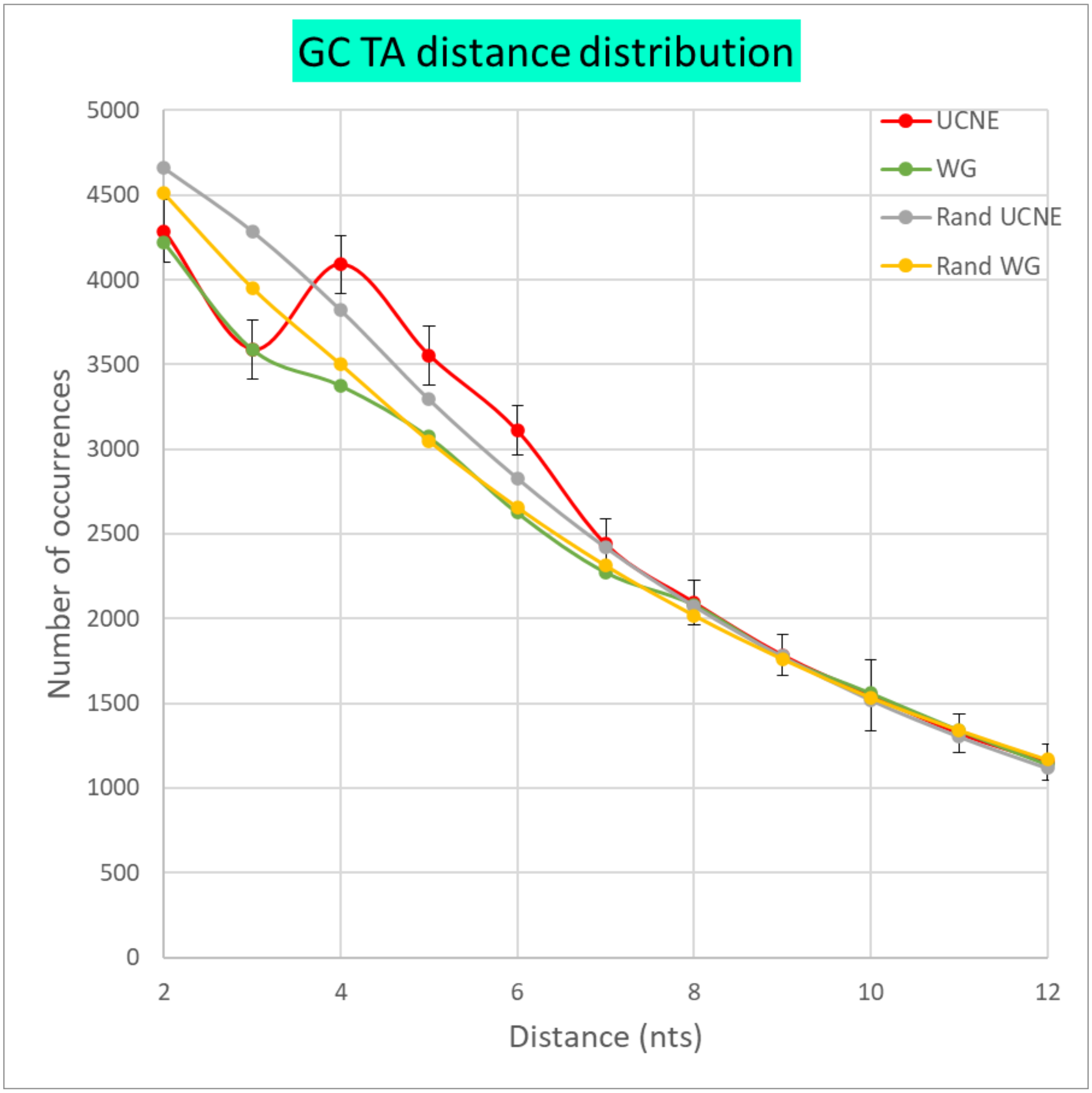
GC TA distance distribution
Number of occurrences
Distance (nts)
UCNE
WG
Rand UCNE
Rand WG
0
500
1000
1500
2000
2500
3000
3500
4000
4500
5000
2
4
6
8
10
12

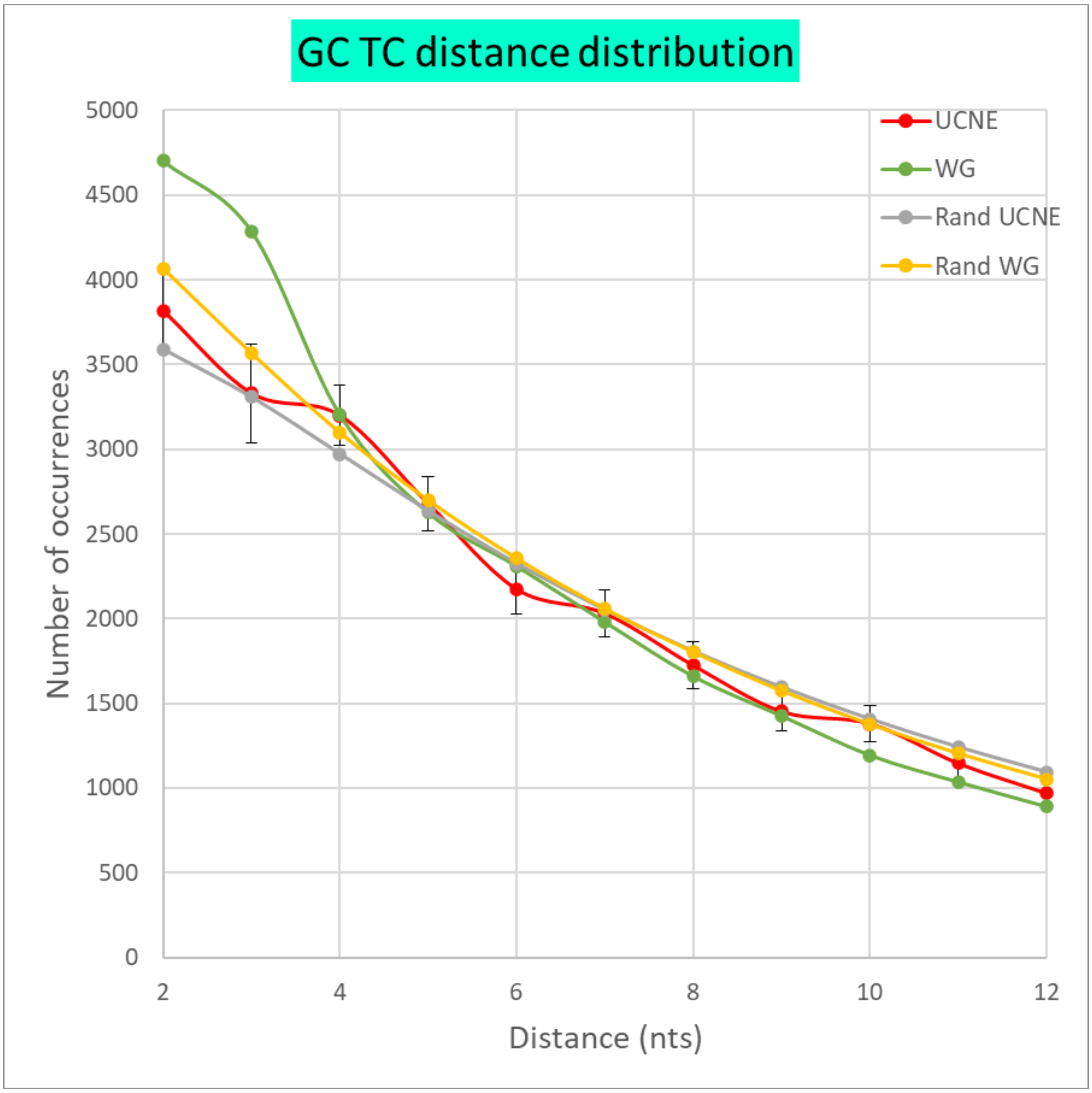

GC TC distance distribution
UCNE
WG
Rand UCNE
Rand WG
Number of occurrences
5000
4500
4000
3500
3000
2500
2000
1500
1000
500
0
2
4
6
8
10
12
Distance (nts)

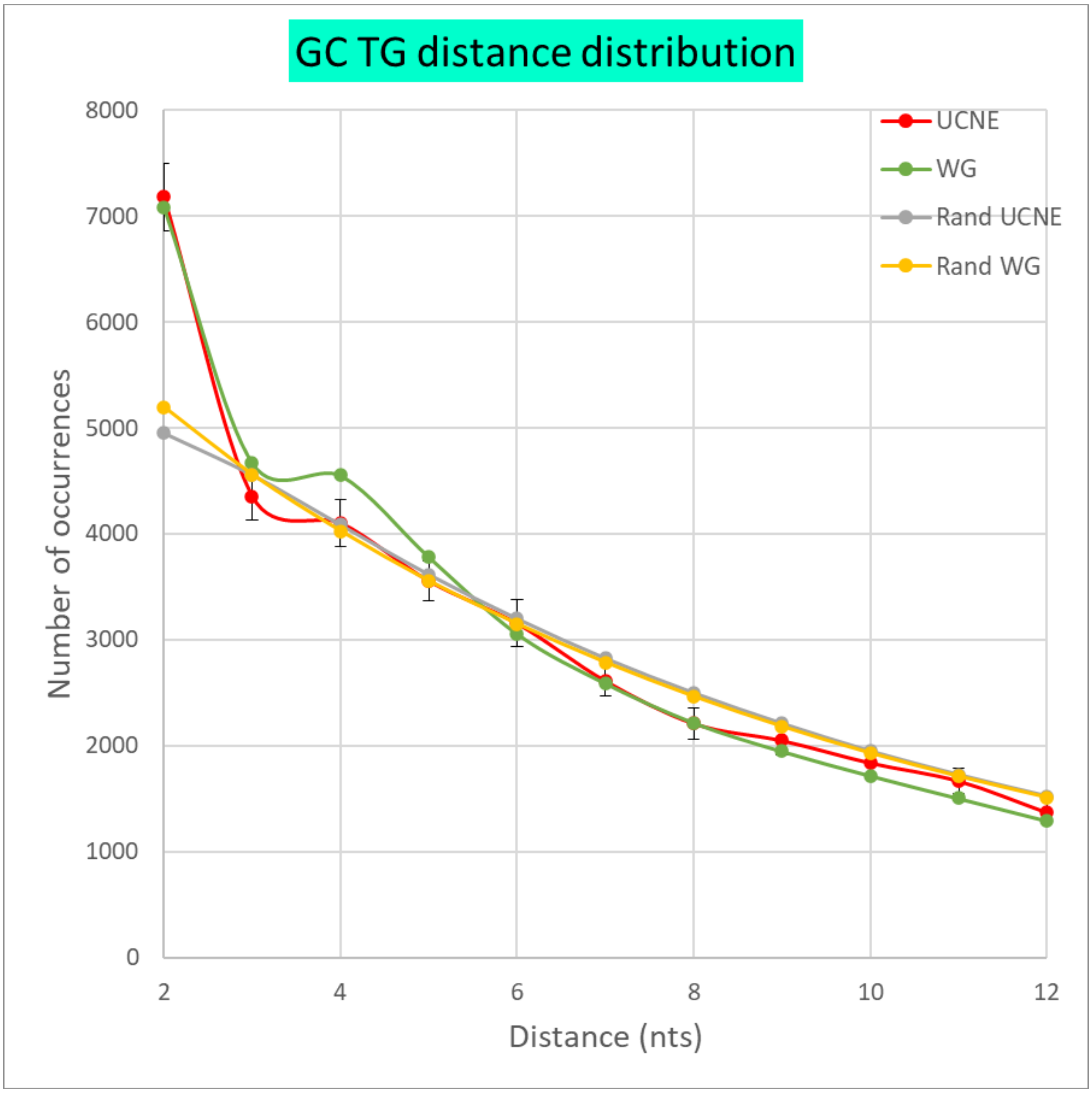

GC TG distance distribution
UCNE
WG
Rand UCNE
Rand WG
Number of occurrences
8000
7000
6000
5000
4000
3000
2000
1000
0
2
4
6
8
10
12
Distance (nts)

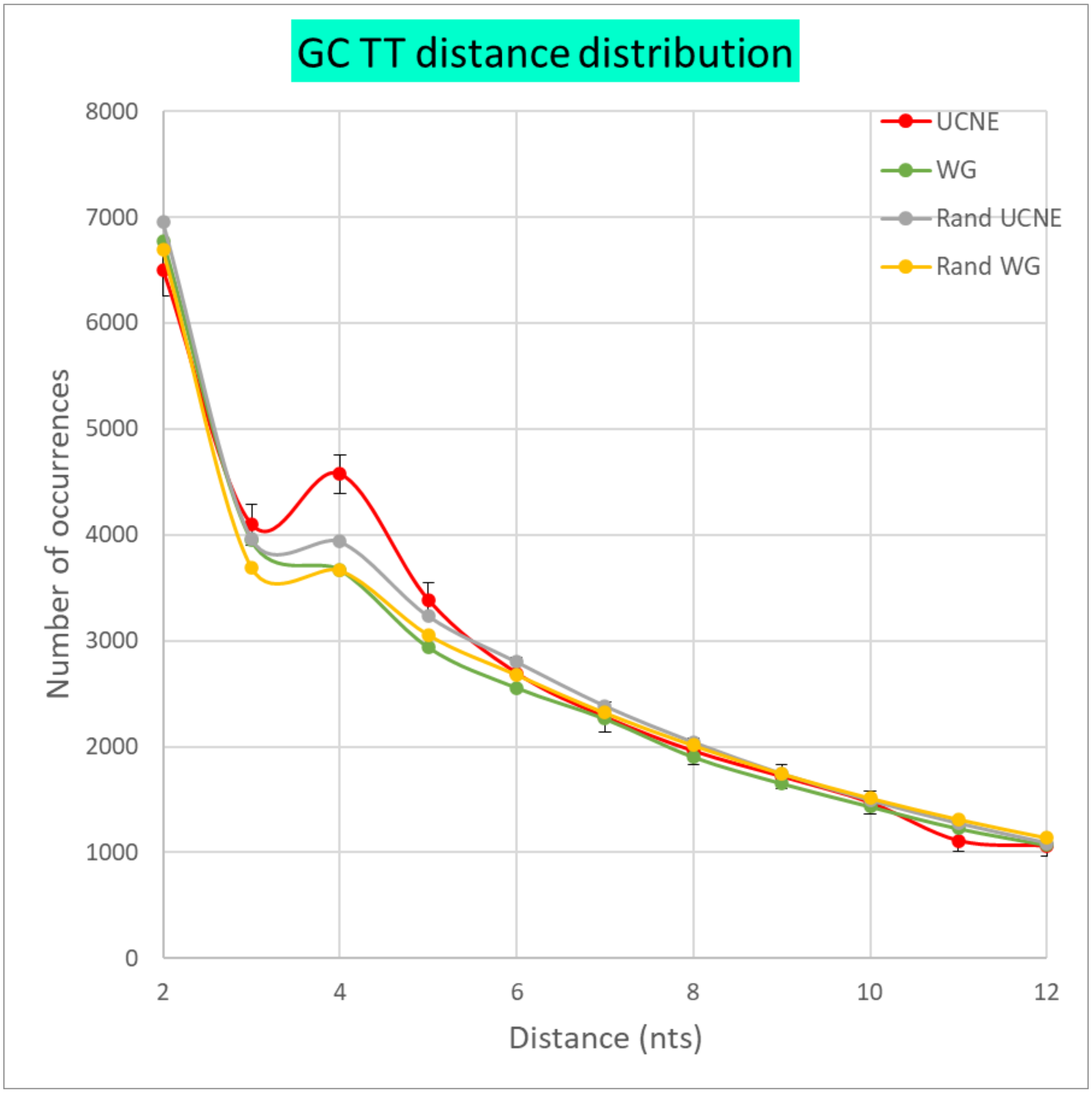

GC TT distance distribution
UCNE
WG
Rand UCNE
Rand WG
Number of occurrences
0
1000
2000
3000
4000
5000
6000
7000
8000
2
4
6
8
10
12
Distance (nts)

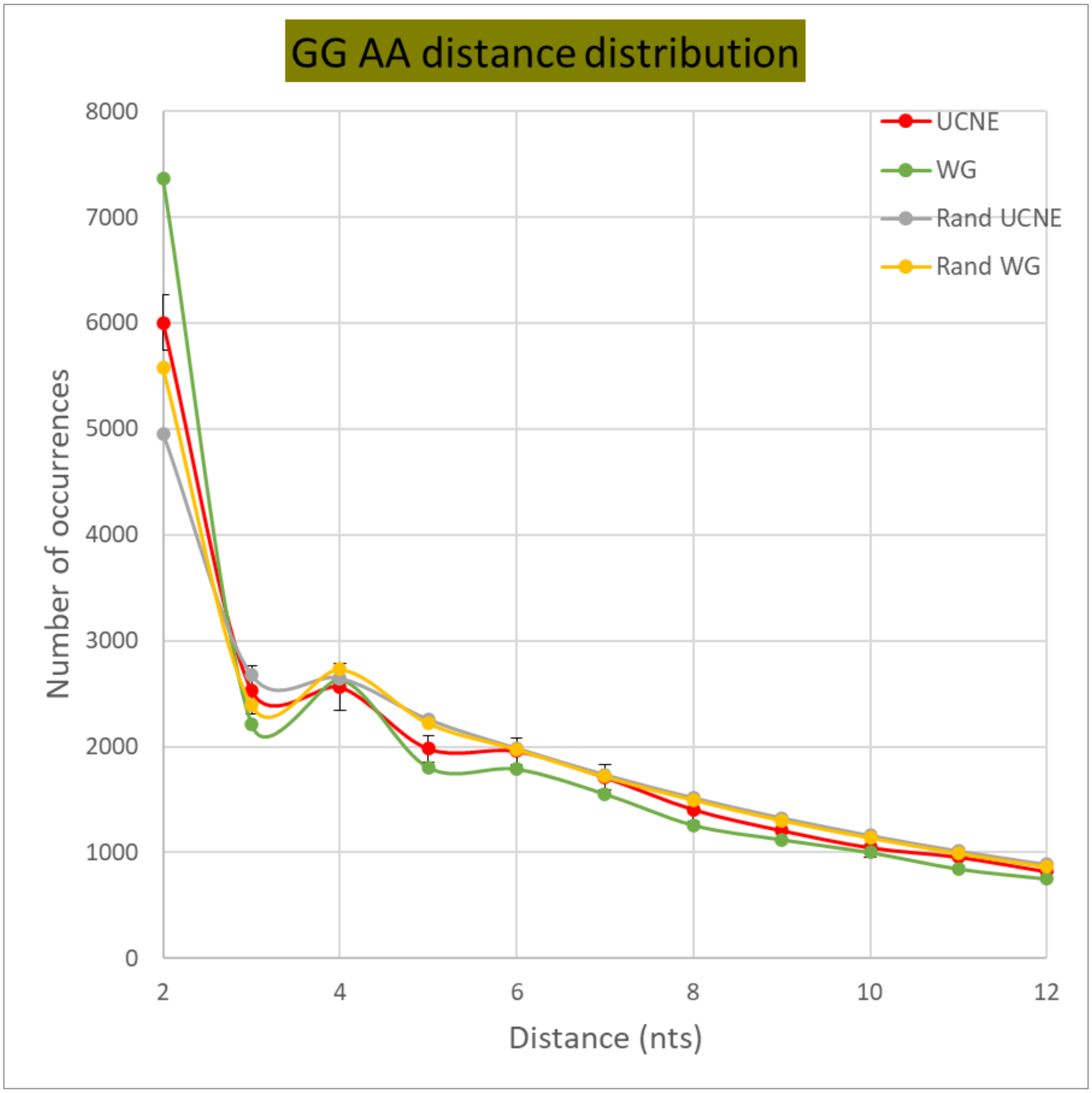

GG AA distance distribution
Number of occurrences
Distance (nts)
UCNE
WG
Rand UCNE
Rand WG
0
1000
2000
3000
4000
5000
6000
7000
8000
2
4
6
8
10
12

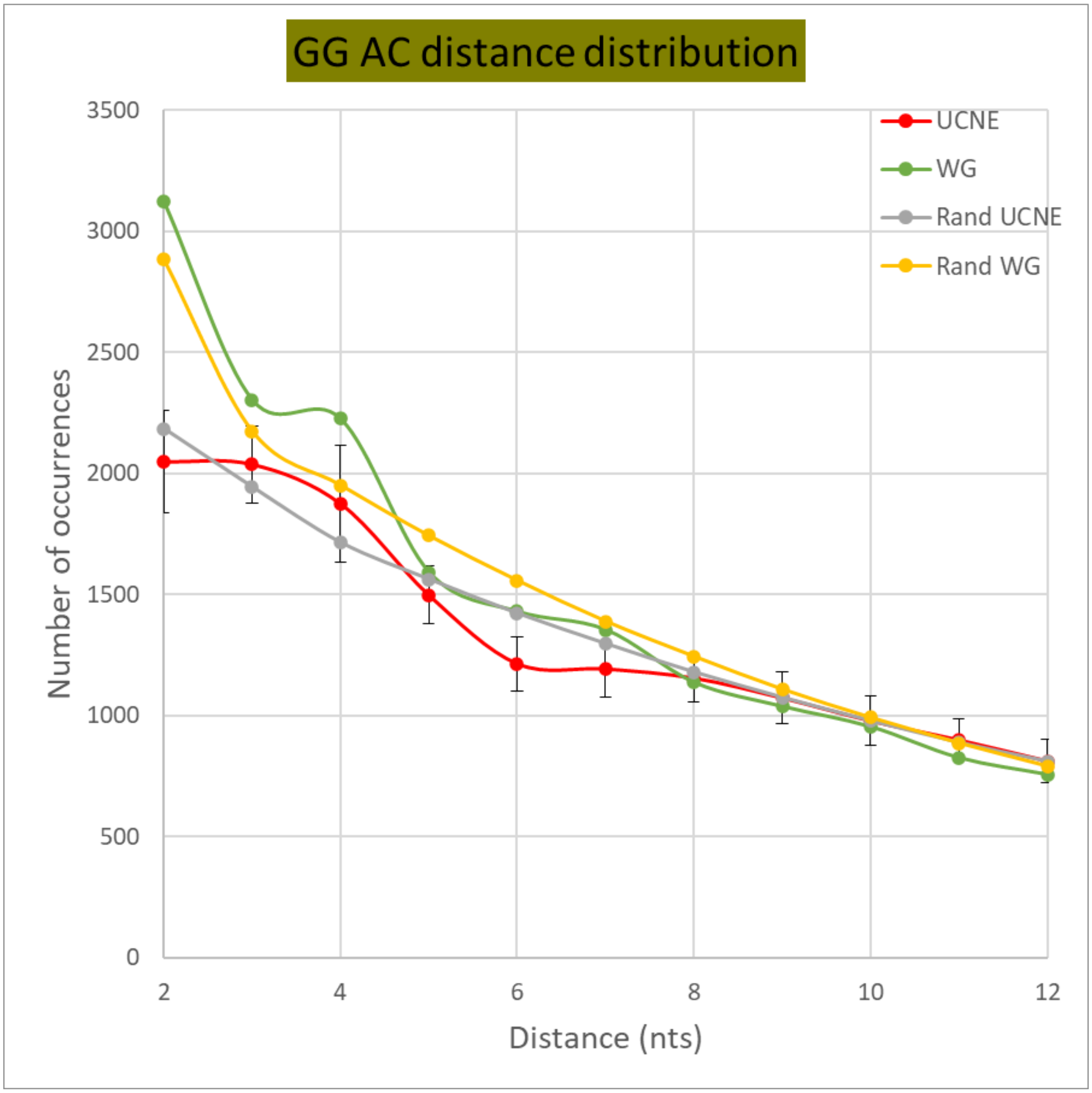
GG AC distance distribution
Number of occurrences
Distance (nts)
UCNE
WG
Rand UCNE
Rand WG
0
500
1000
1500
2000
2500
3000
3500
2
4
6
8
10
12

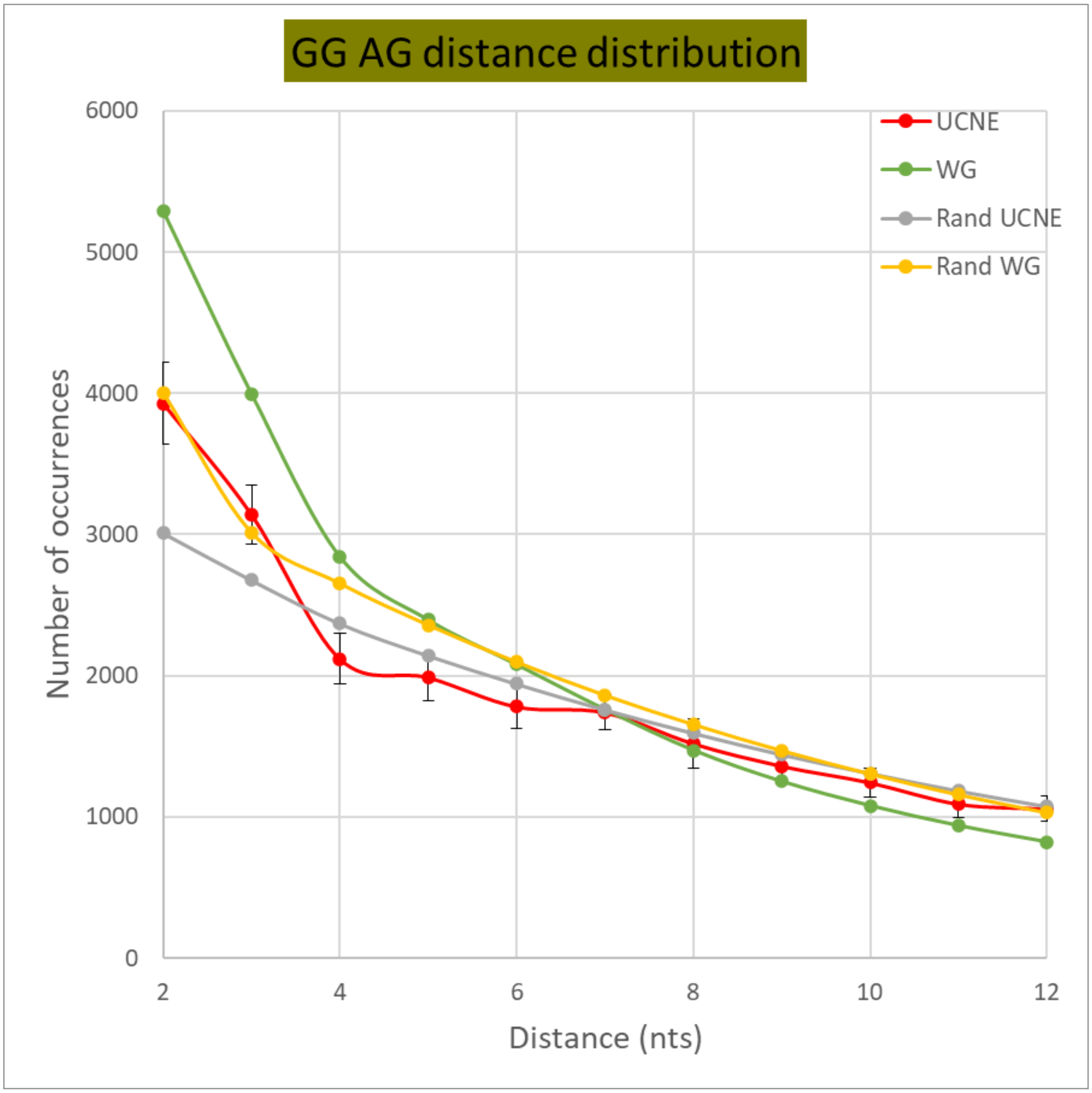
GG AG distance distribution
6000
5000
4000
3000
2000
1000
0
2
4
6
8
10
12
Number of occurrences
Distance (nts)
UCNE
WG
Rand UCNE
Rand WG

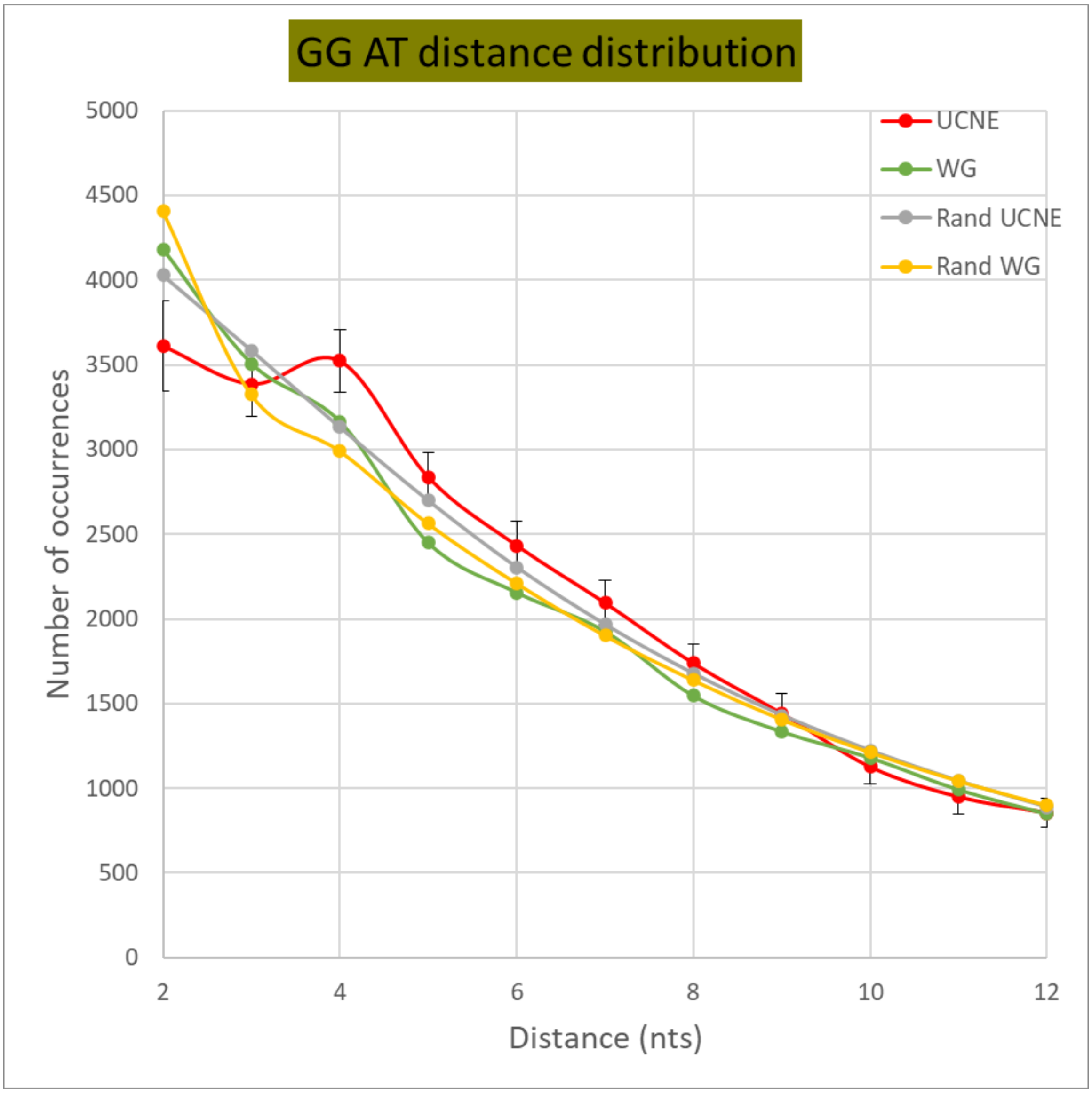
GG AT distance distribution
UCNE
WG
Rand UCNE
Rand WG
Number of occurrences
5000
4500
4000
3500
3000
2500
2000
1500
1000
500
0
2
4
6
8
10
12
Distance (nts)

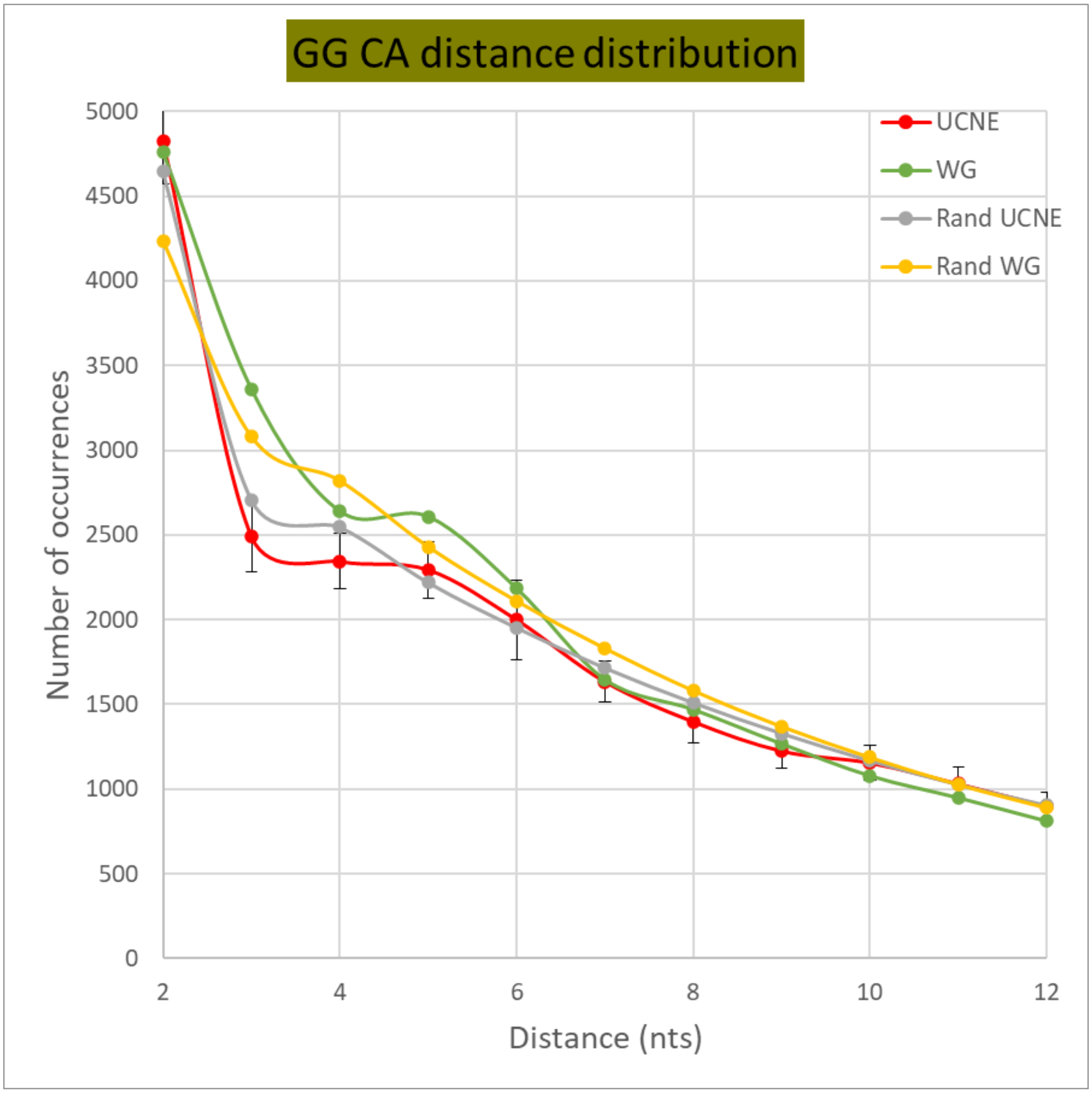
GG CA distance distribution
Number of occurrences
Distance (nts)
UCNE
WG
Rand UCNE
Rand WG
0
500
1000
1500
2000
2500
3000
3500
4000
4500
5000
2
4
6
8
10
12

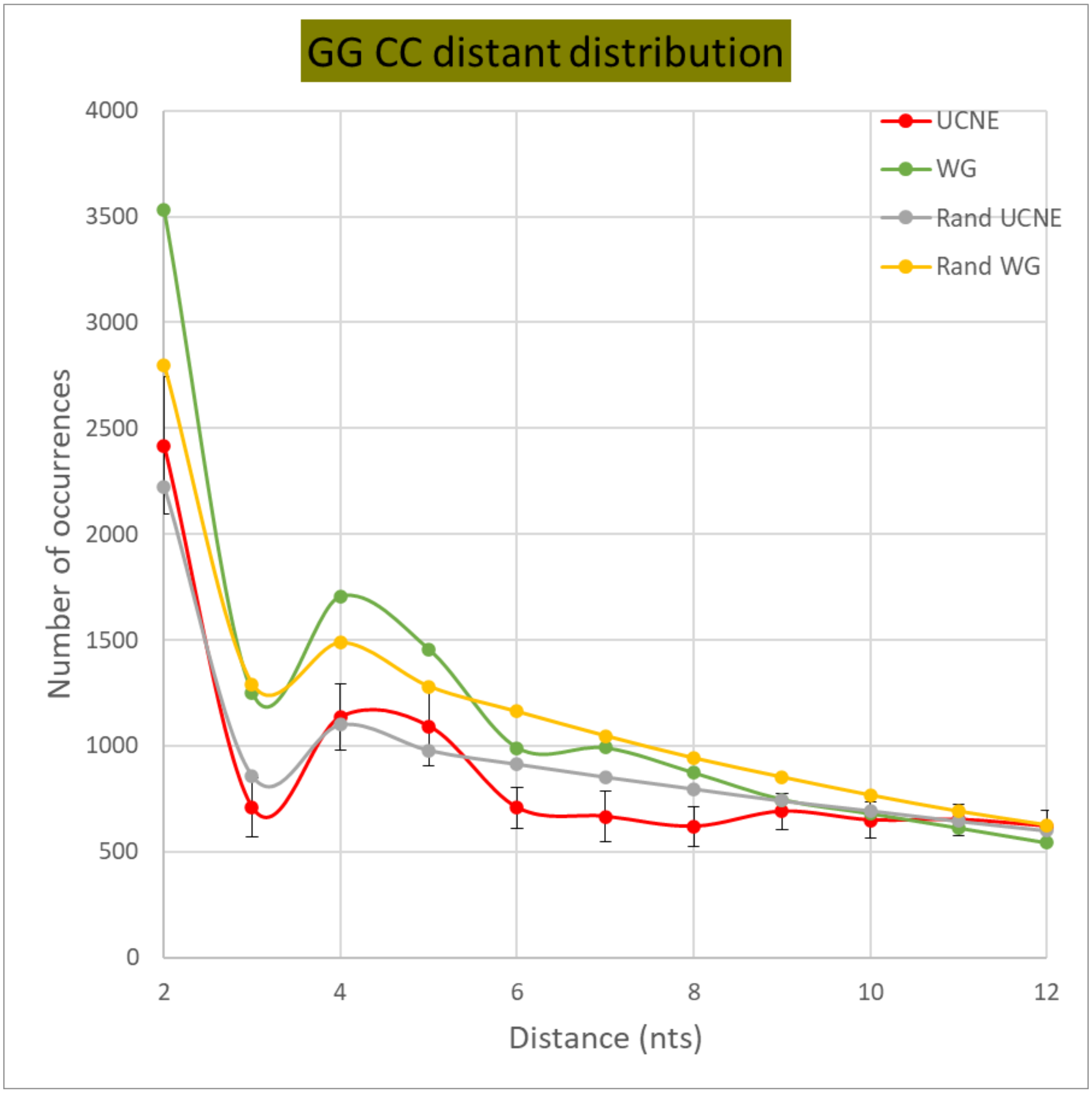

GG CC distant distribution
UCNE
WG
Rand UCNE
Rand WG
Number of occurrences
0
500
1000
1500
2000
2500
3000
3500
4000
2
4
6
8
10
12
Distance (nts)

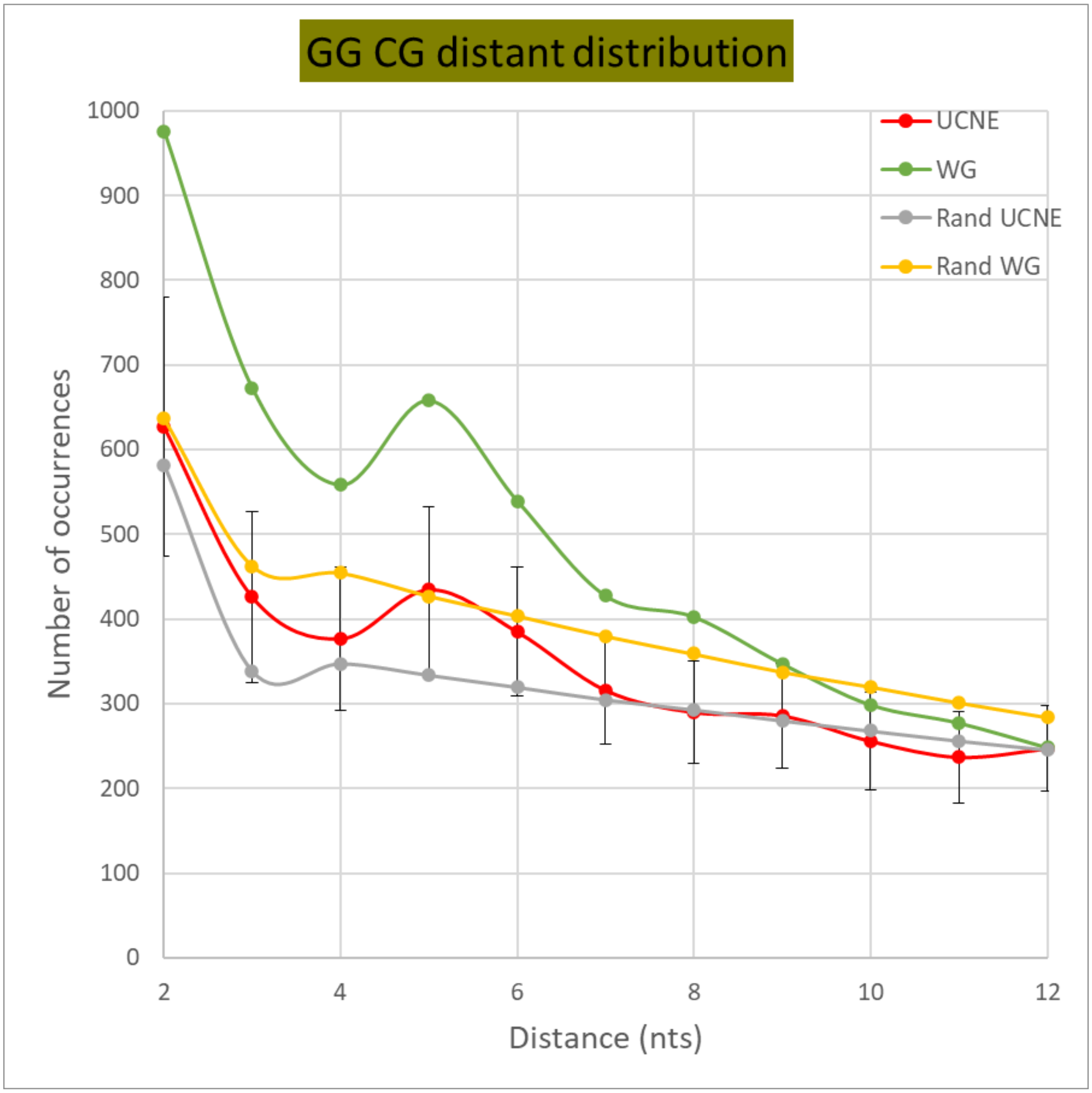

GG CG distant distribution
Number of occurrences
Distance (nts)
UCNE
WG
Rand UCNE
Rand WG
0
100
200
300
400
500
600
700
800
900
1000
2
4
6
8
10
12

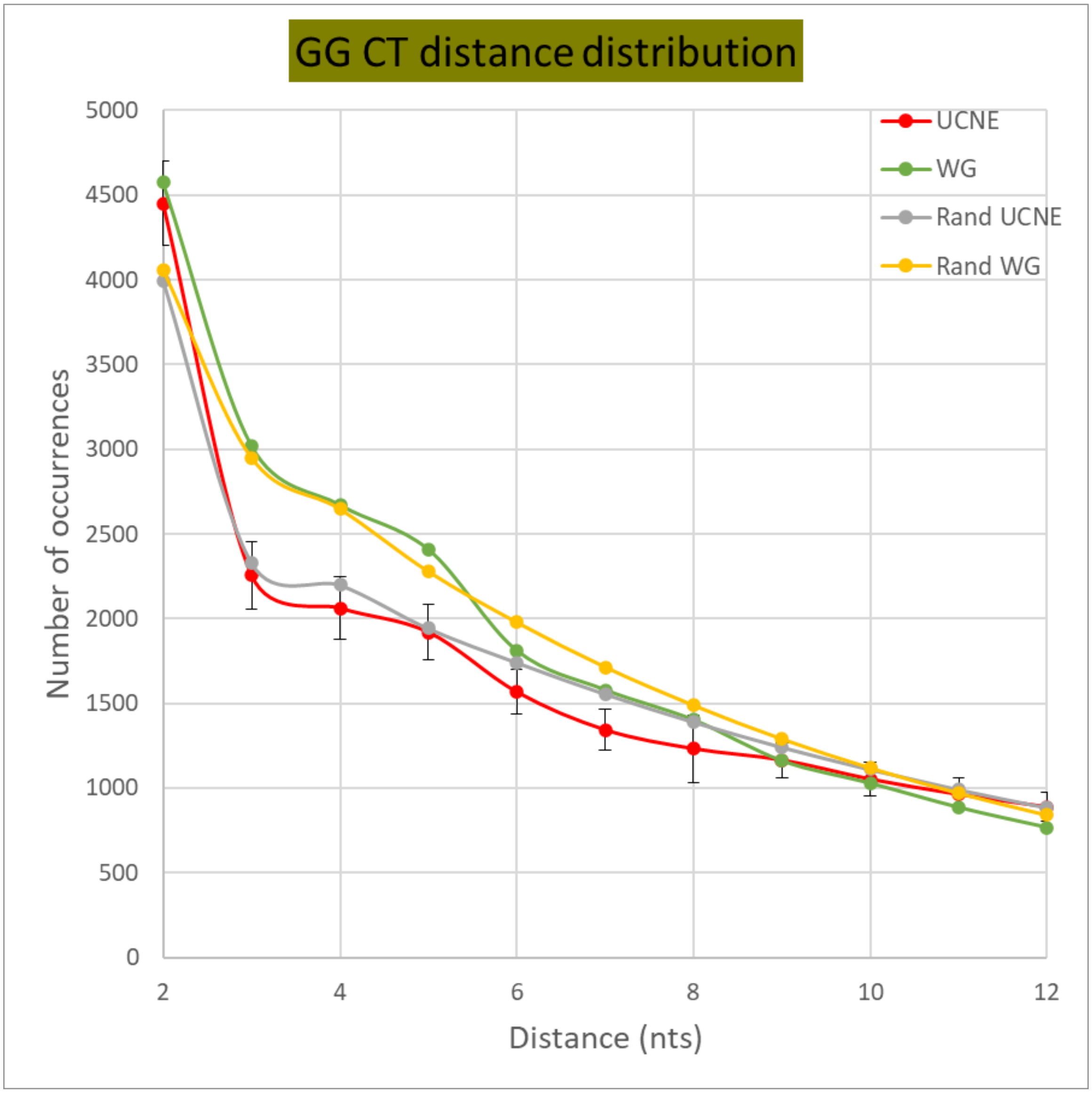

GG CT distance distribution
UCNE
WG
Rand UCNE
Rand WG
Number of occurrences
Distance (nts)
0
500
1000
1500
2000
2500
3000
3500
4000
4500
5000
2
4
6
8
10
12

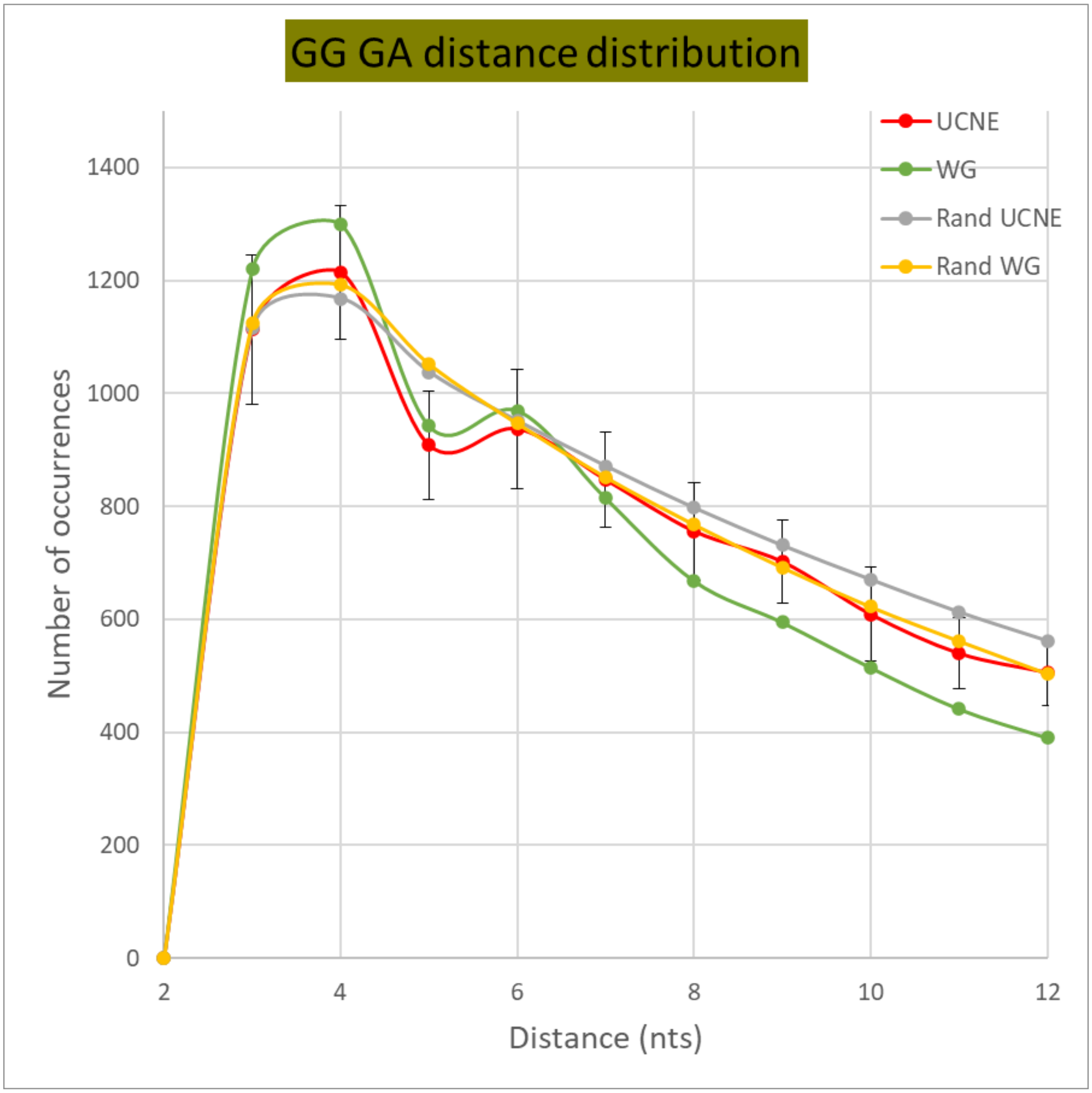
GG GA distance distribution
UCNE
WG
Rand UCNE
Rand WG
Number of occurrences
Distance (nts)
0
200
400
600
800
1000
1200
1400
2
4
6
8
10
12

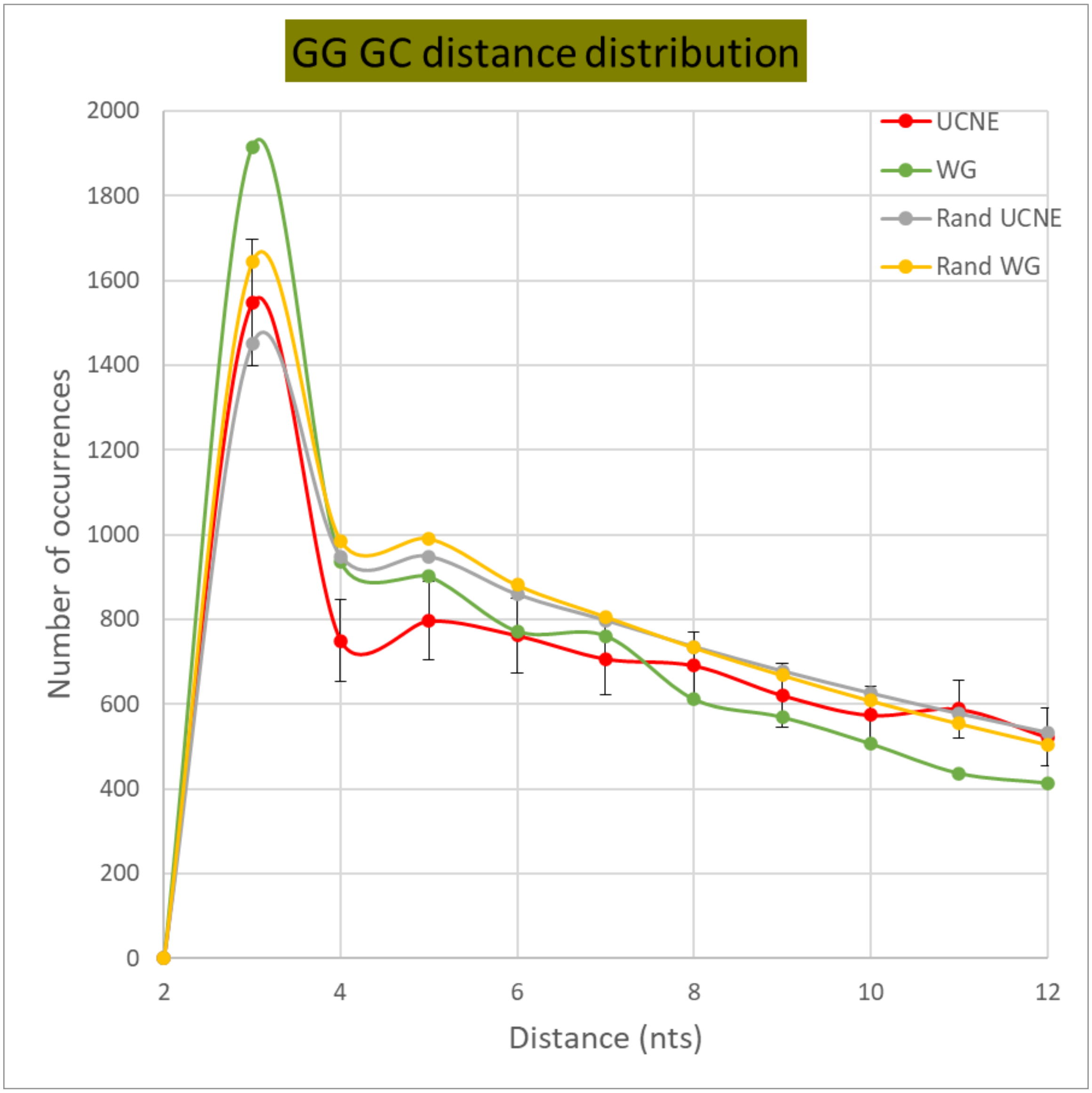

GG GC distance distribution
Number of occurrences
Distance (nts)
UCNE
WG
Rand UCNE
Rand WG
0
200
400
600
800
1000
1200
1400
1600
1800
2000
2
4
6
8
10
12

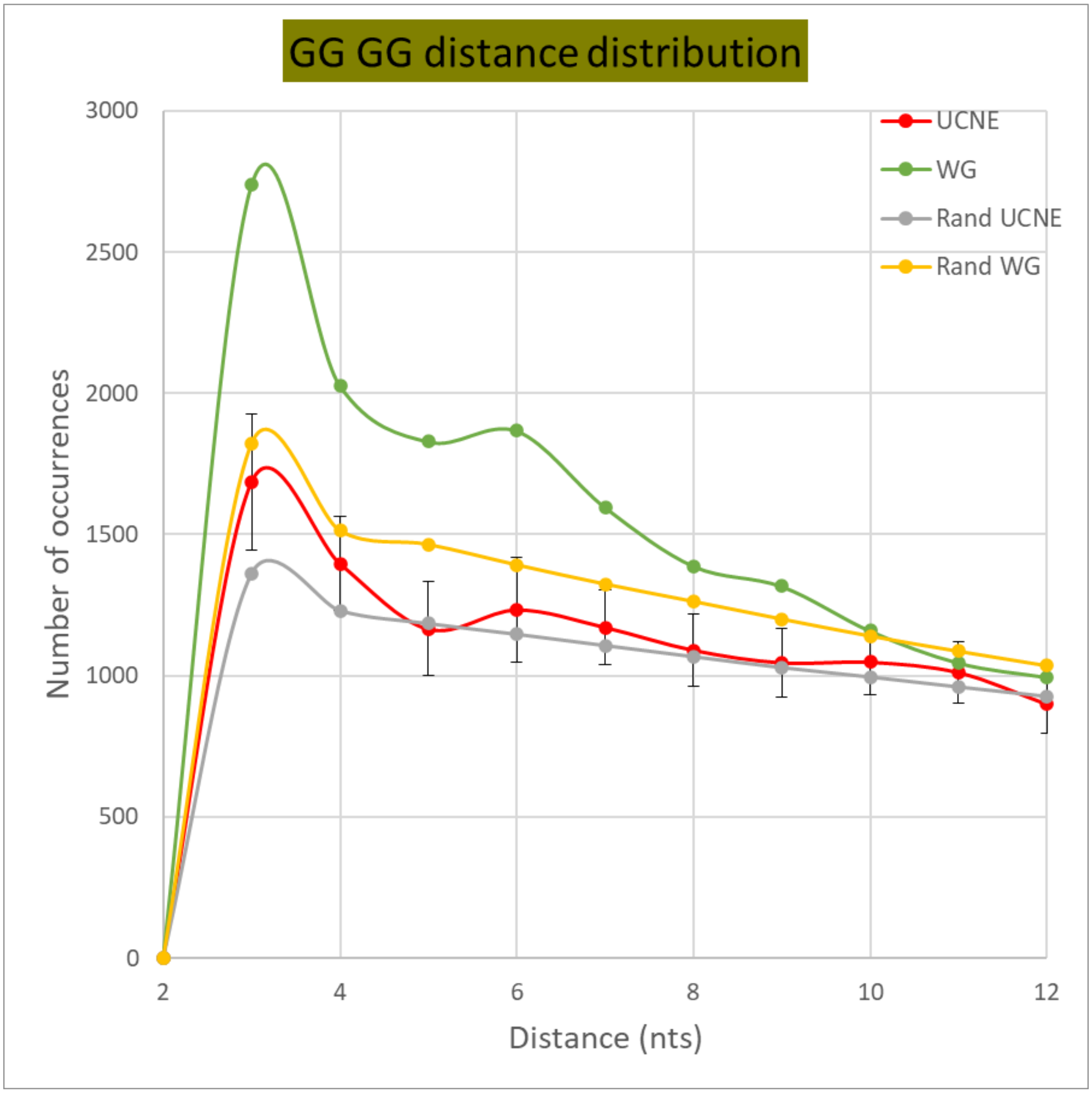
GG GG distance distribution
UCNE
WG
Rand UCNE
Rand WG
Number of occurrences
Distance (nts)
3000
2500
2000
1500
1000
500
0
2
4
6
8
10
12

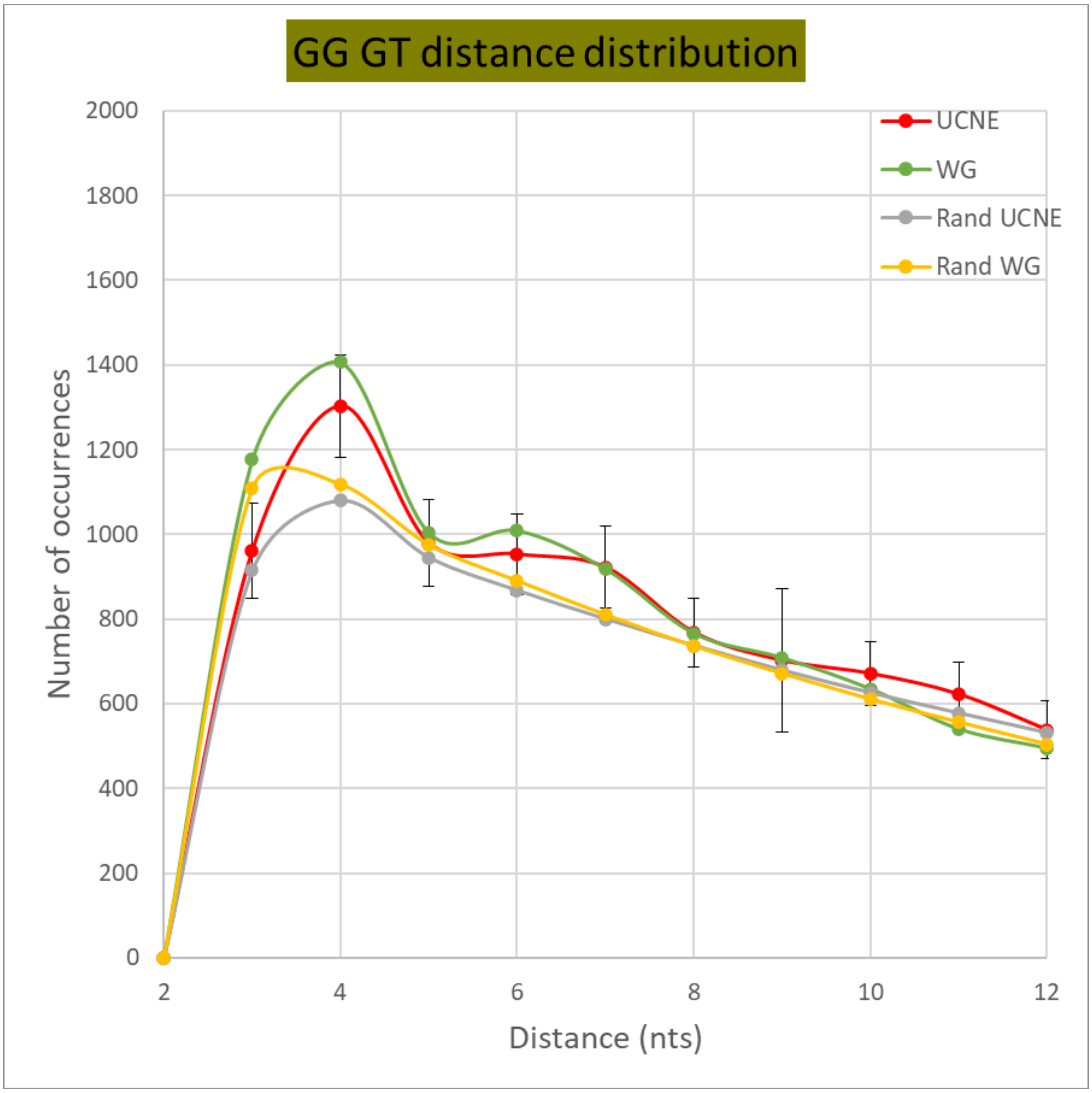

GG GT distance distribution
UCNE
WG
Rand UCNE
Rand WG
2000
1800
1600
1400
1200
1000
800
600
400
200
0
Number of occurrences
2
4
6
8
10
12
Distance (nts)

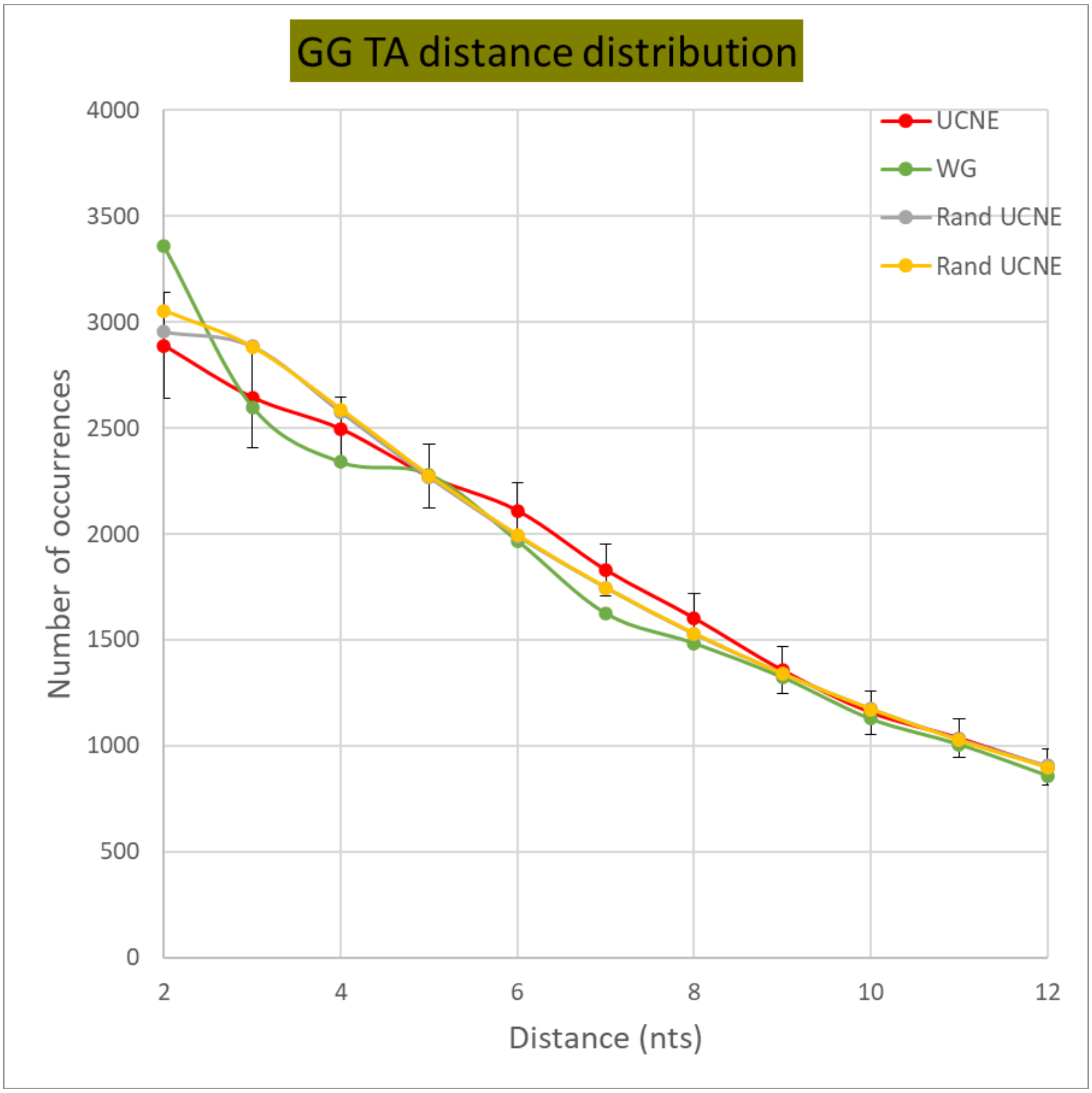
GG TA distance distribution
Number of occurrences
4000
3500
3000
2500
2000
1500
1000
500
0
2
4
6
8
10
12
Distance (nts)
UCNE
WG
Rand UCNE
Rand UCNE

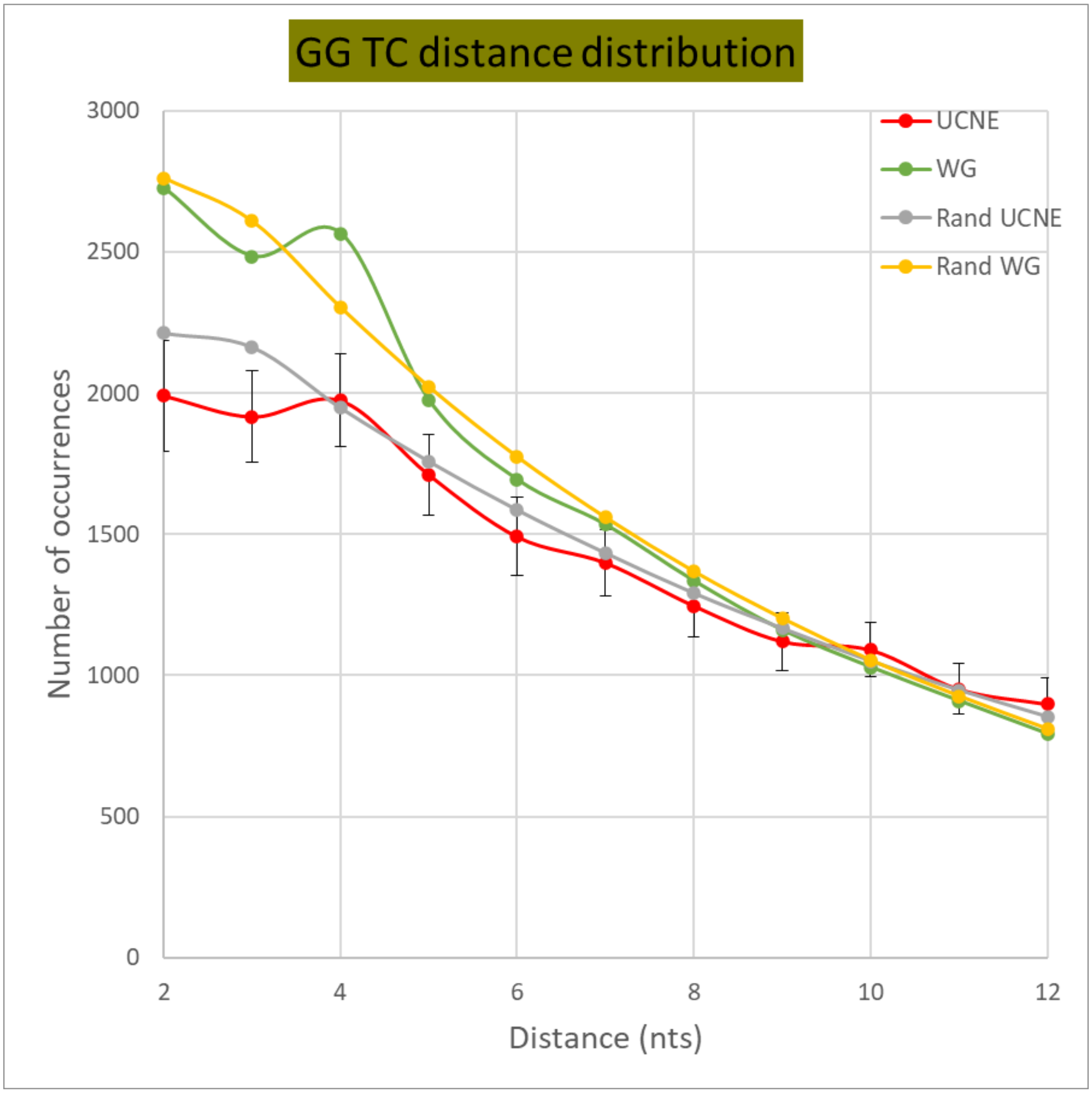

GG TC distance distribution
UCNE
WG
Rand UCNE
Rand WG
Number of occurrences
0
500
1000
1500
2000
2500
3000
2
4
6
8
10
12
Distance (nts)

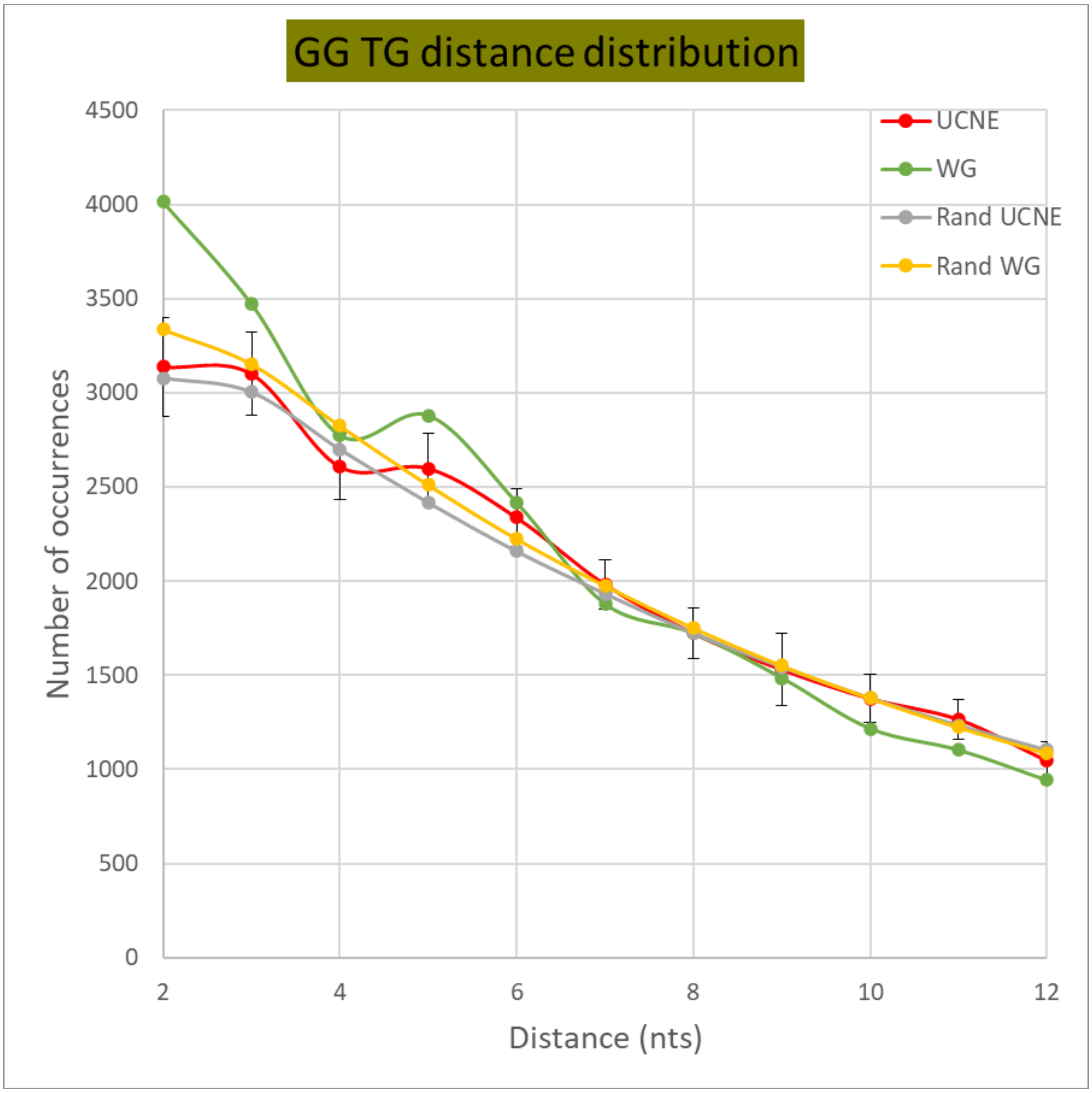
GG TG distance distribution
Number of occurrences
Distance (nts)
UCNE
WG
Rand UCNE
Rand WG
4500
4000
3500
3000
2500
2000
1500
1000
500
0
2
4
6
8
10
12

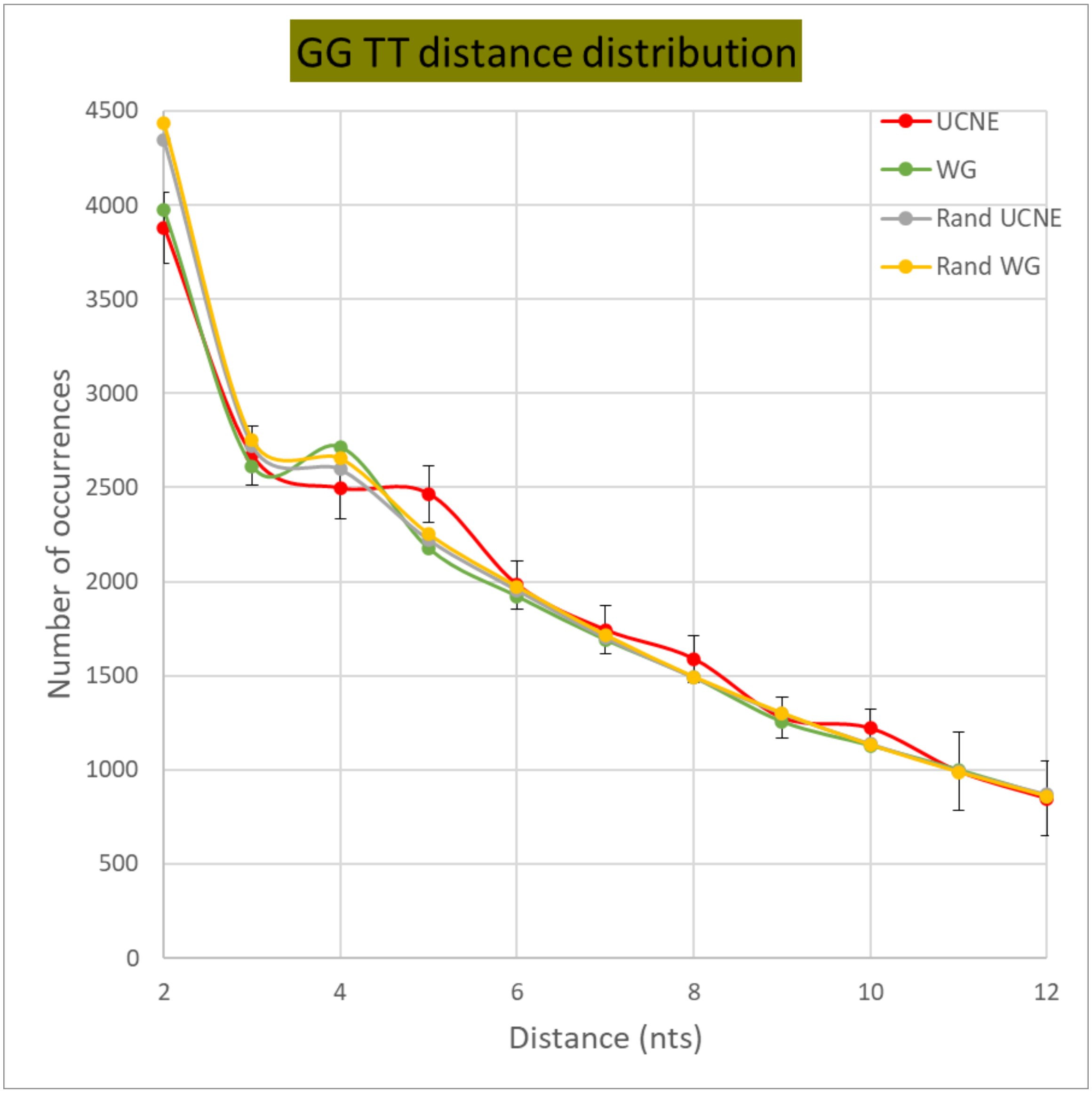

GG TT distance distribution
UCNE
WG
Rand UCNE
Rand WG
Number of occurrences
Distance (nts)
0
500
1000
1500
2000
2500
3000
3500
4000
4500
2
4
6
8
10
12

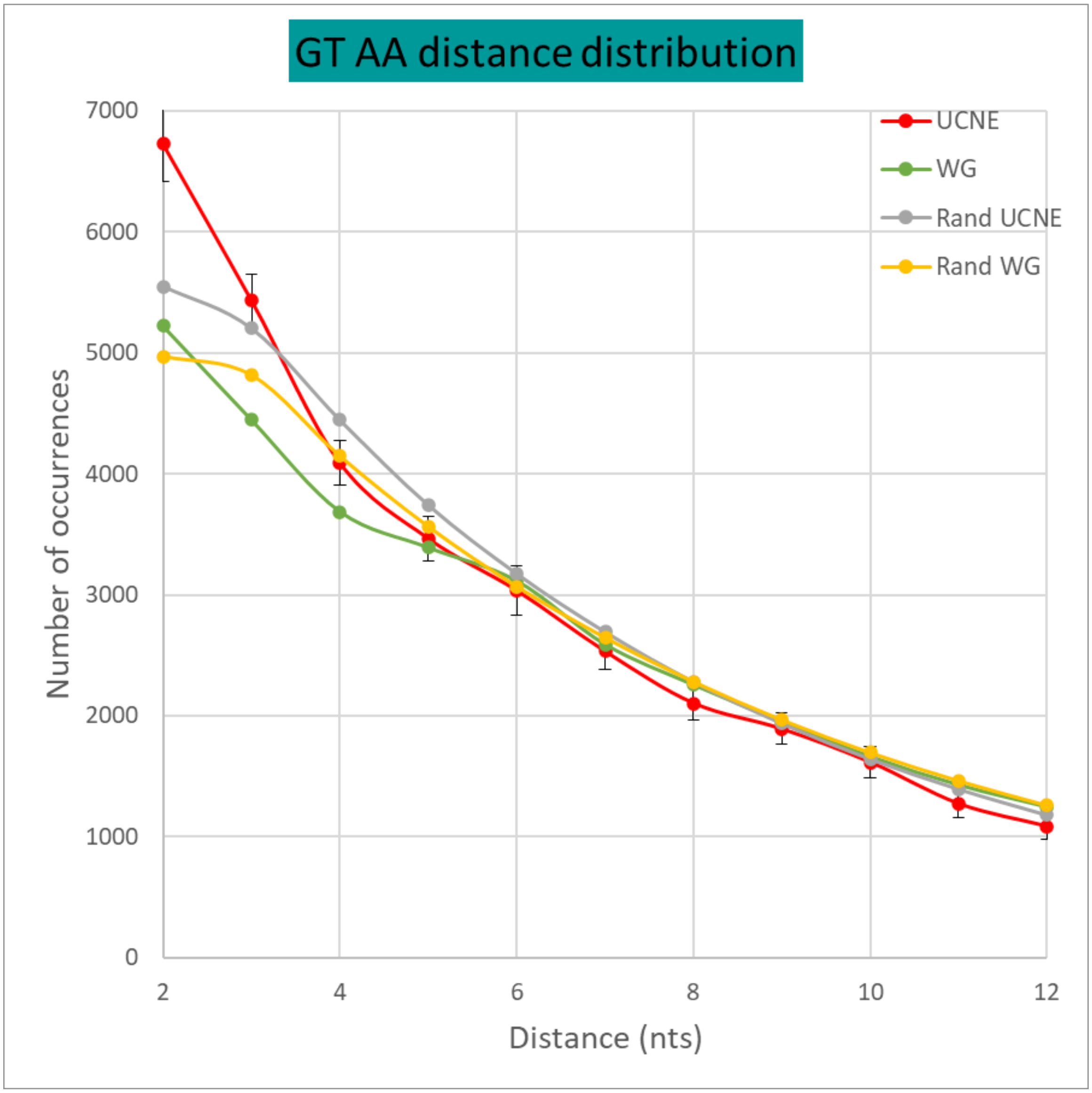

GT AA distance distribution
Number of occurrences
Distance (nts)
UCNE
WG
Rand UCNE
Rand WG
7000
6000
5000
4000
3000
2000
1000
0
2
4
6
8
10
12

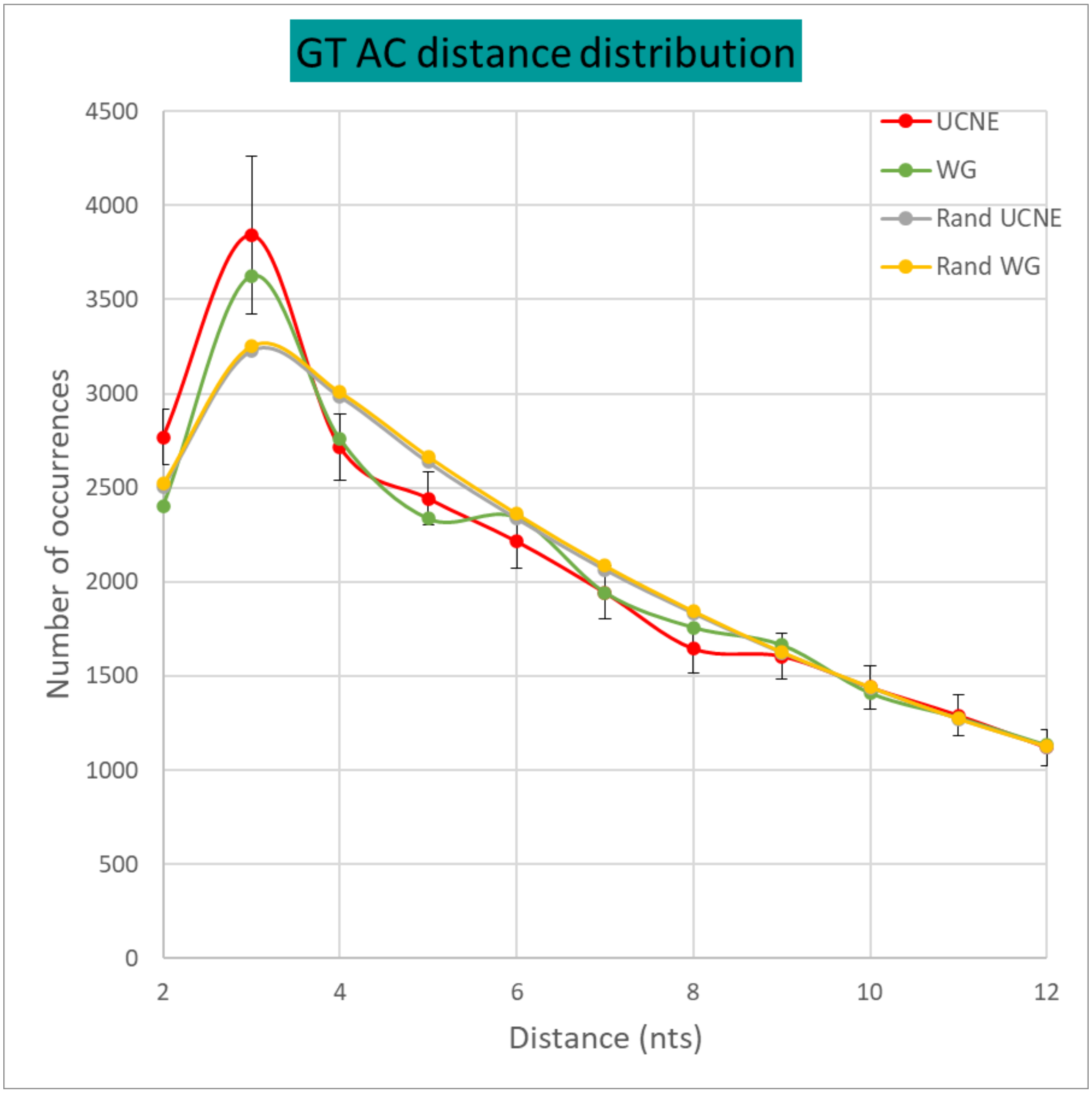
GT AC distance distribution
UCNE
WG
Rand UCNE
Rand WG
Number of occurrences
4500
4000
3500
3000
2500
2000
1500
1000
500
0
2
4
6
8
10
12
Distance (nts)

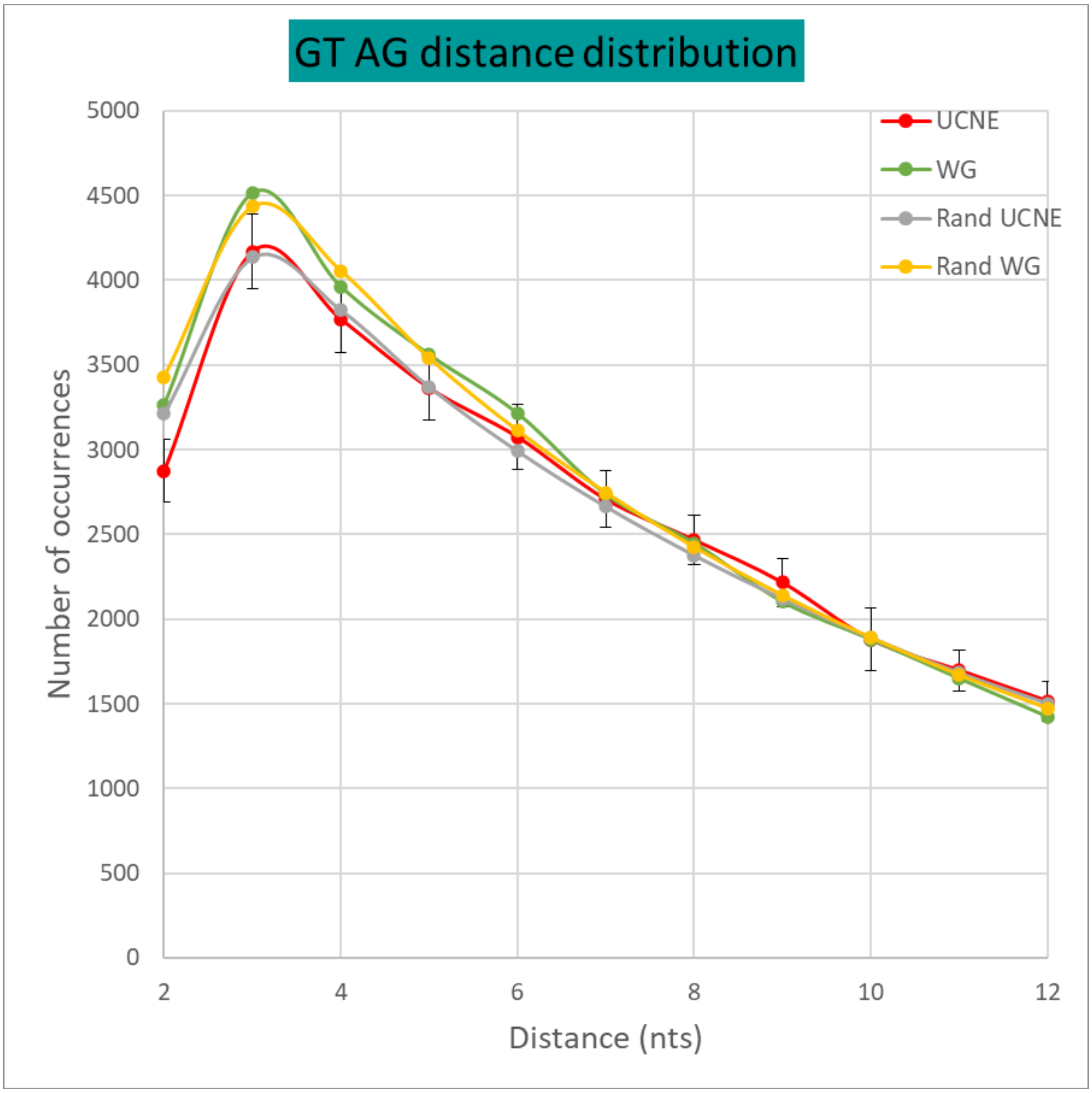
GT AG distance distribution
Number of occurrences
Distance (nts)
UCNE
WG
Rand UCNE
Rand WG
0
500
1000
1500
2000
2500
3000
3500
4000
4500
5000
2
4
6
8
10
12

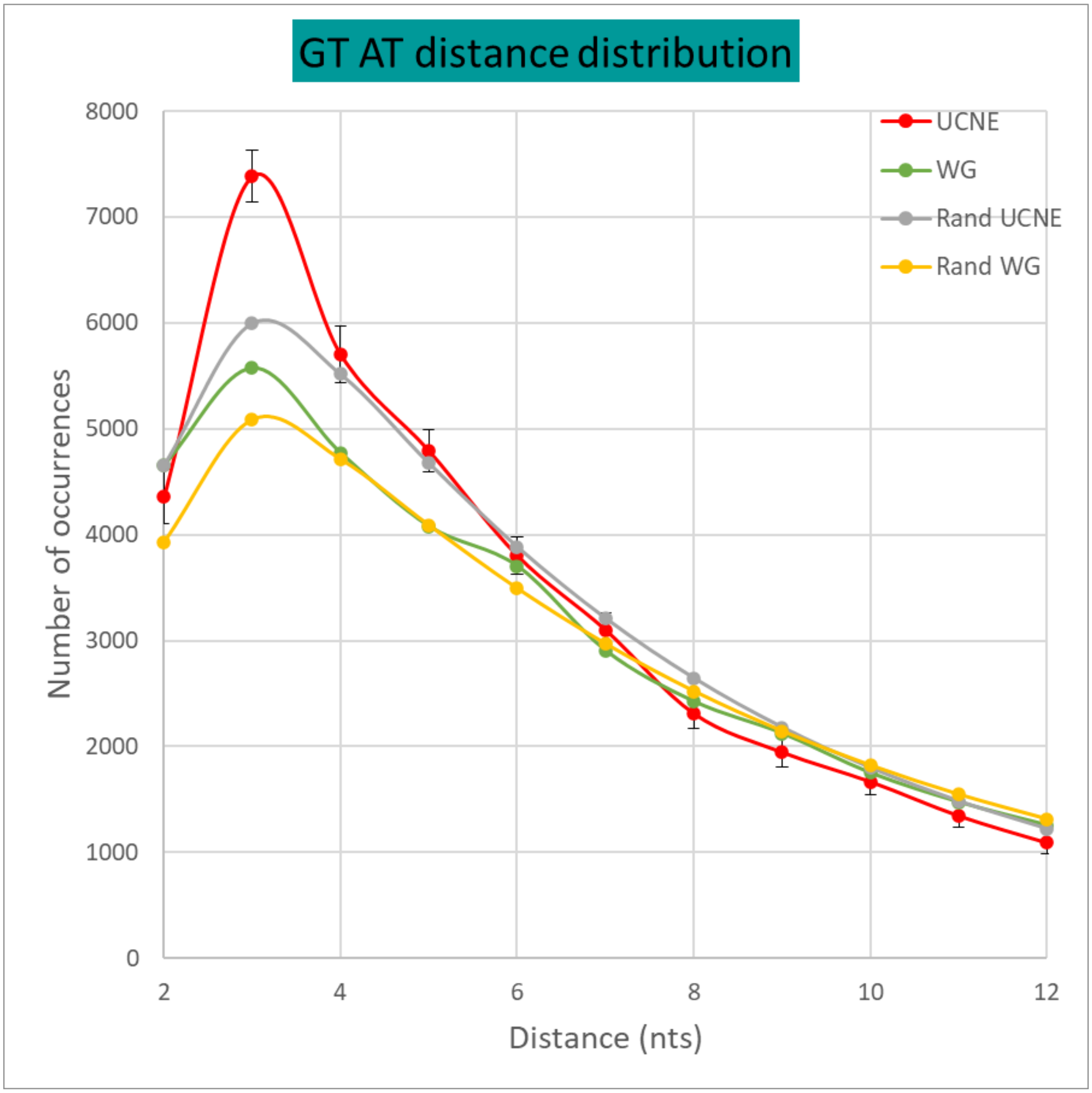

GT AT distance distribution
UCNE
WG
Rand UCNE
Rand WG
8000
7000
6000
5000
4000
3000
2000
1000
0
Number of occurrences
2
4
6
8
10
12
Distance (nts)

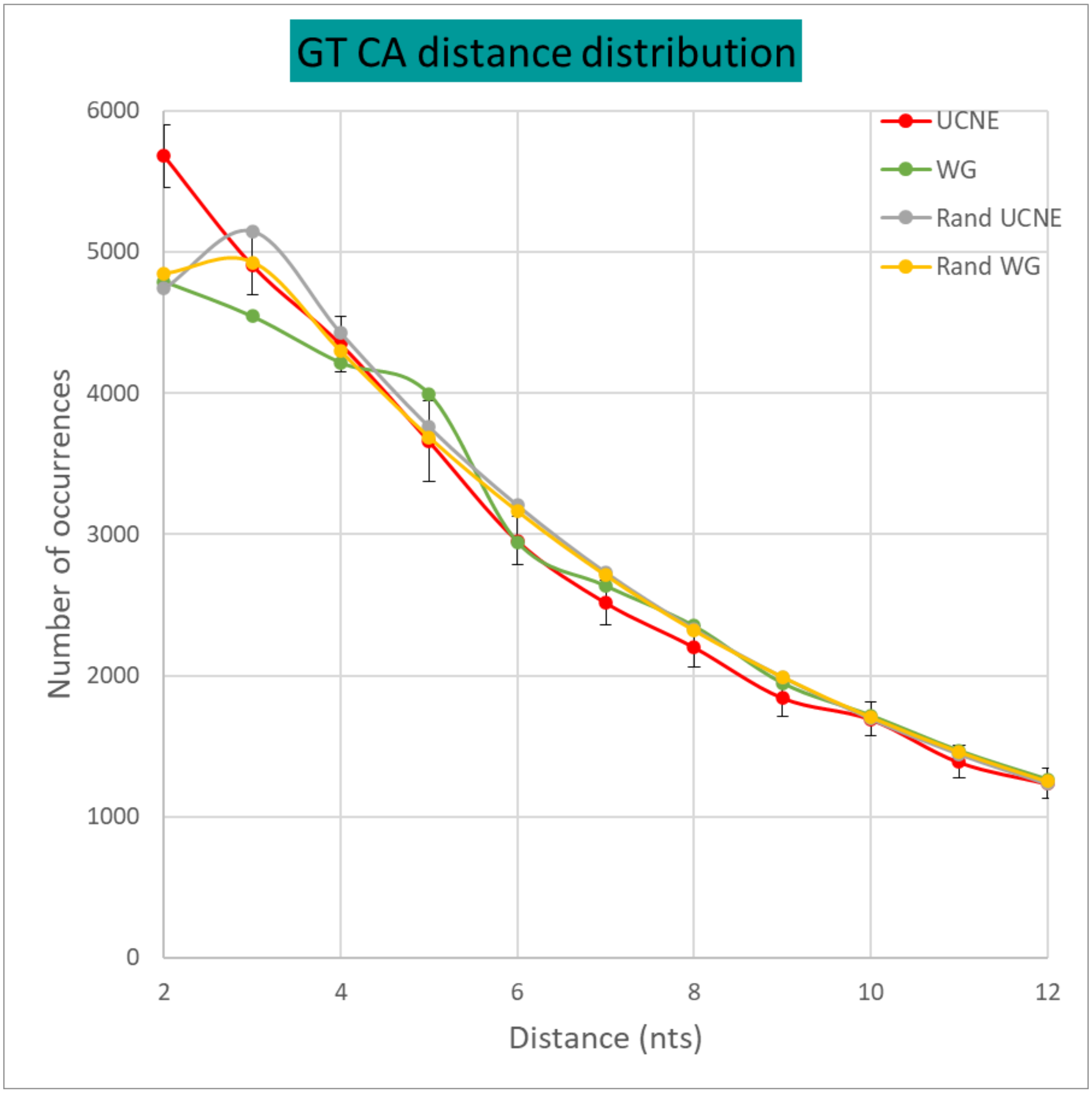
GT CA distance distribution
UCNE
WG
Rand UCNE
Rand WG
Number of occurrences
0
1000
2000
3000
4000
5000
6000
2
4
6
8
10
12
Distance (nts)

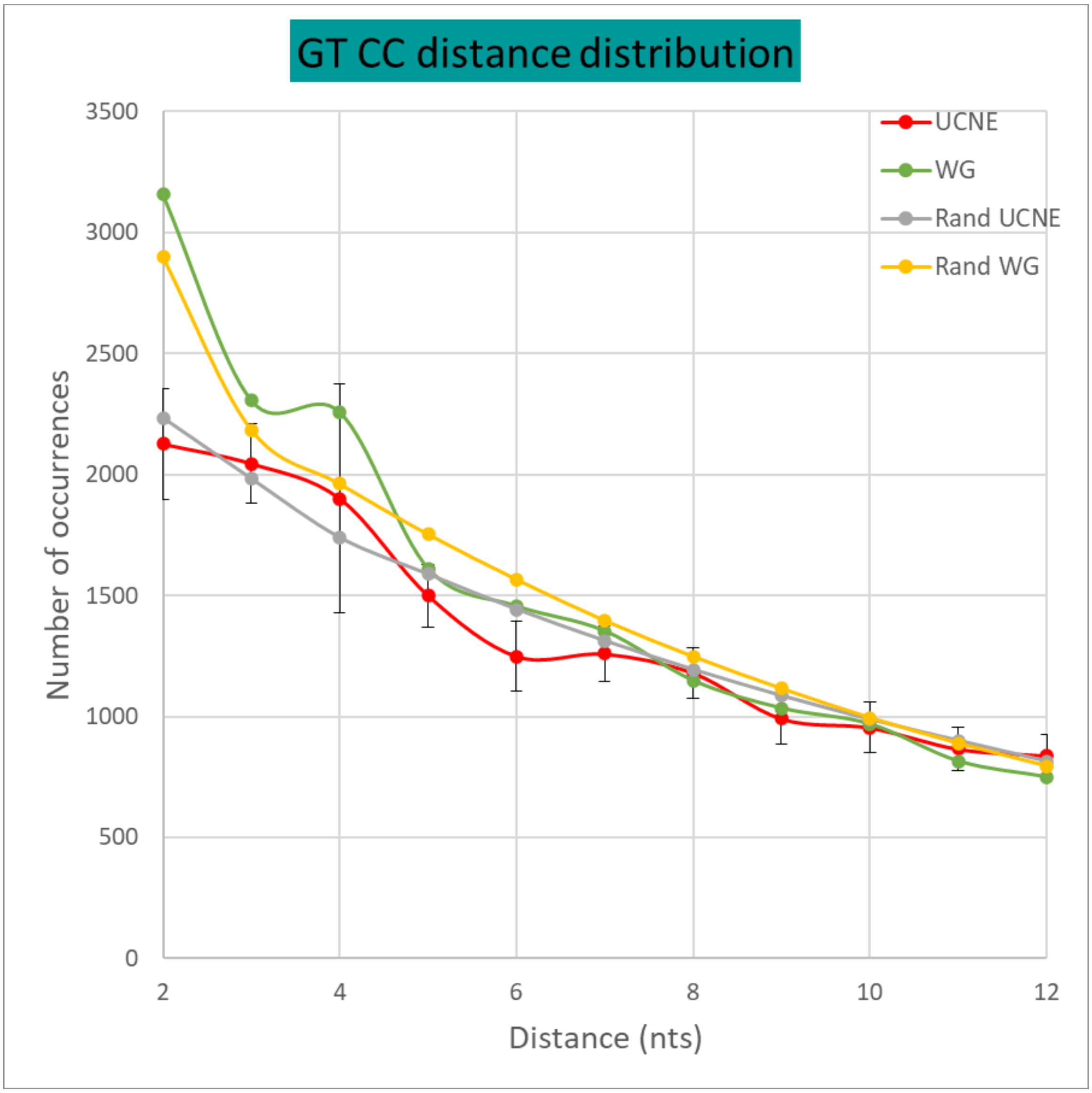
GT CC distance distribution
Number of occurrences
Distance (nts)
UCNE
WG
Rand UCNE
Rand WG
0
500
1000
1500
2000
2500
3000
3500
2
4
6
8
10
12

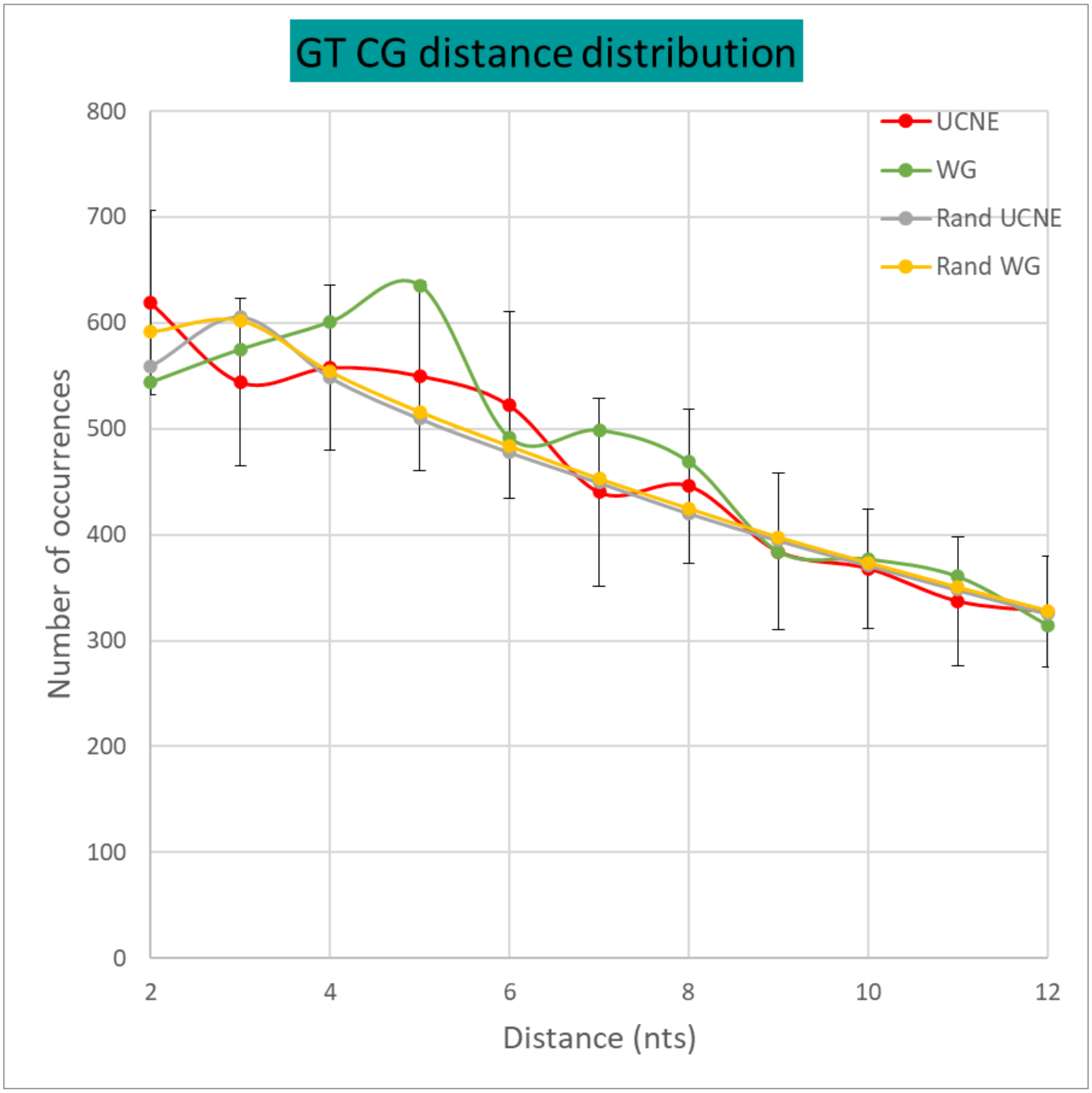
GT CG distance distribution
800
700
600
500
400
300
200
100
0
Number of occurrences
2
4
6
8
10
12
Distance (nts)
UCNE
WG
Rand UCNE
Rand WG

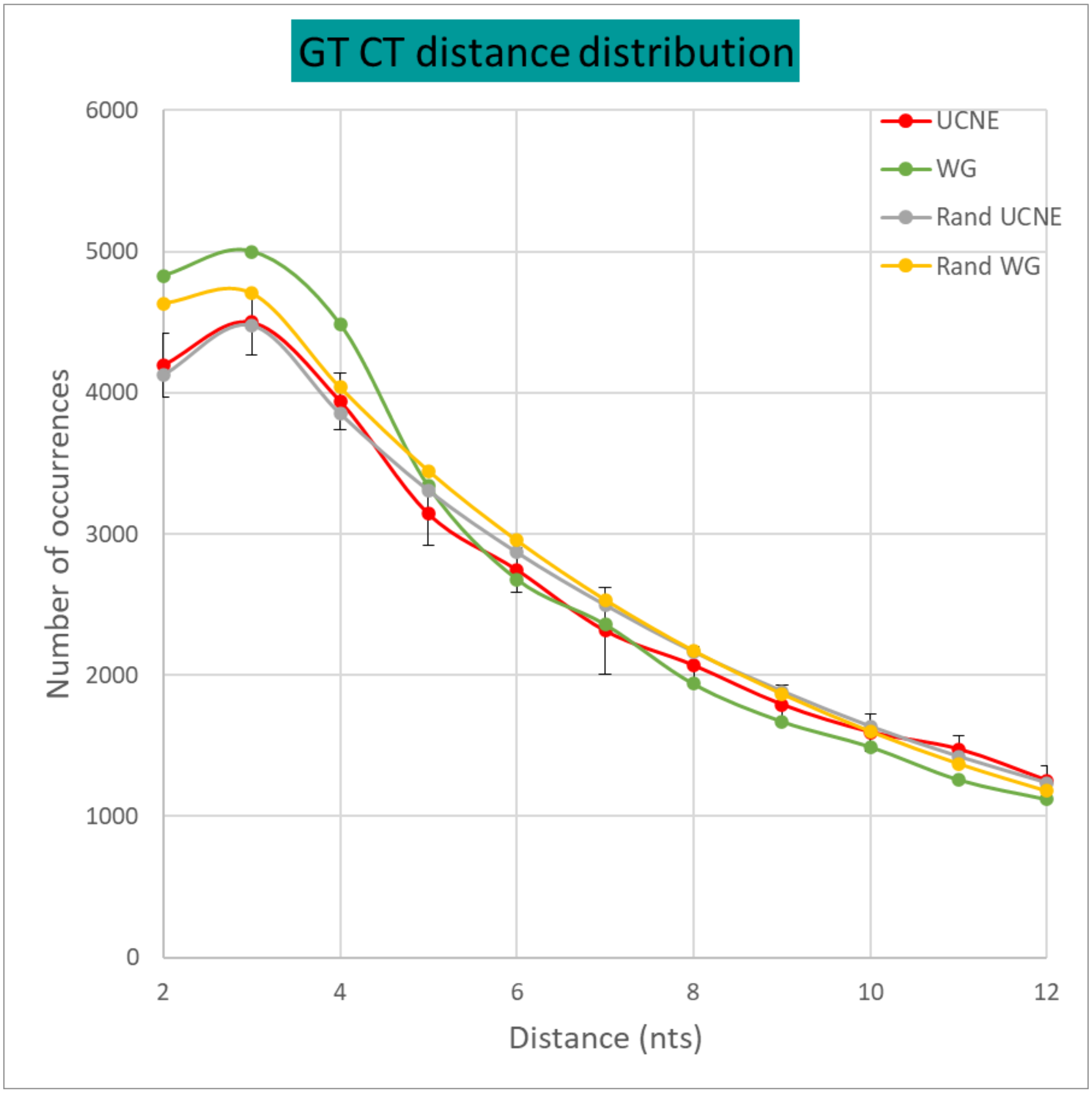

GT CT distance distribution
Number of occurrences
6000
5000
4000
3000
2000
1000
0
2
4
6
8
10
12
Distance (nts)
UCNE
WG
Rand UCNE
Rand WG

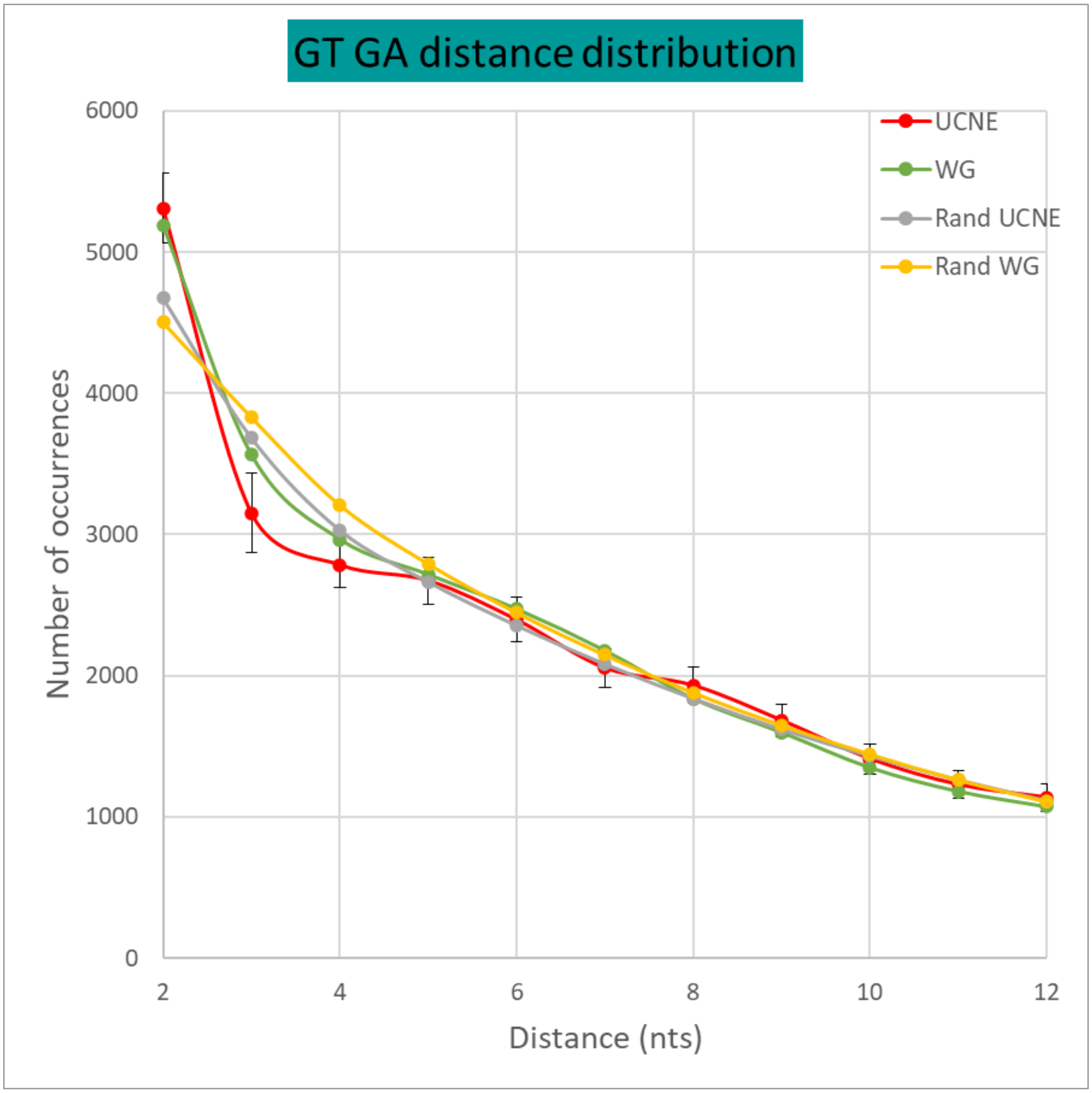

GT GA distance distribution
UCNE
WG
Rand UCNE
Rand WG
Number of occurrences
Distance (nts)
0
1000
2000
3000
4000
5000
6000
2
4
6
8
10
12

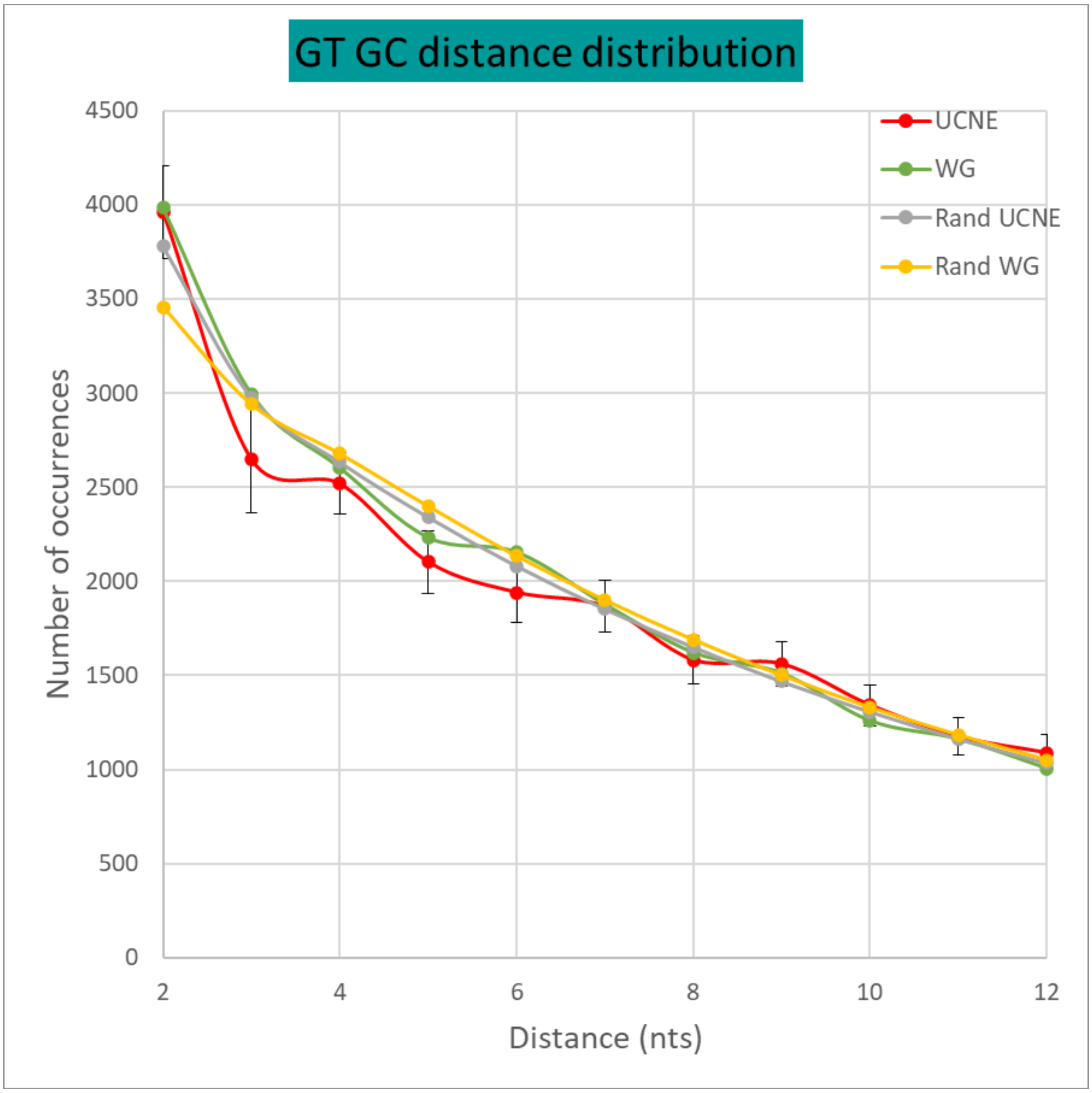
GT GC distance distribution
4500
4000
3500
3000
2500
2000
1500
1000
500
0
Number of occurrences
2
4
6
8
10
12
Distance (nts)
UCNE
WG
Rand UCNE
Rand WG

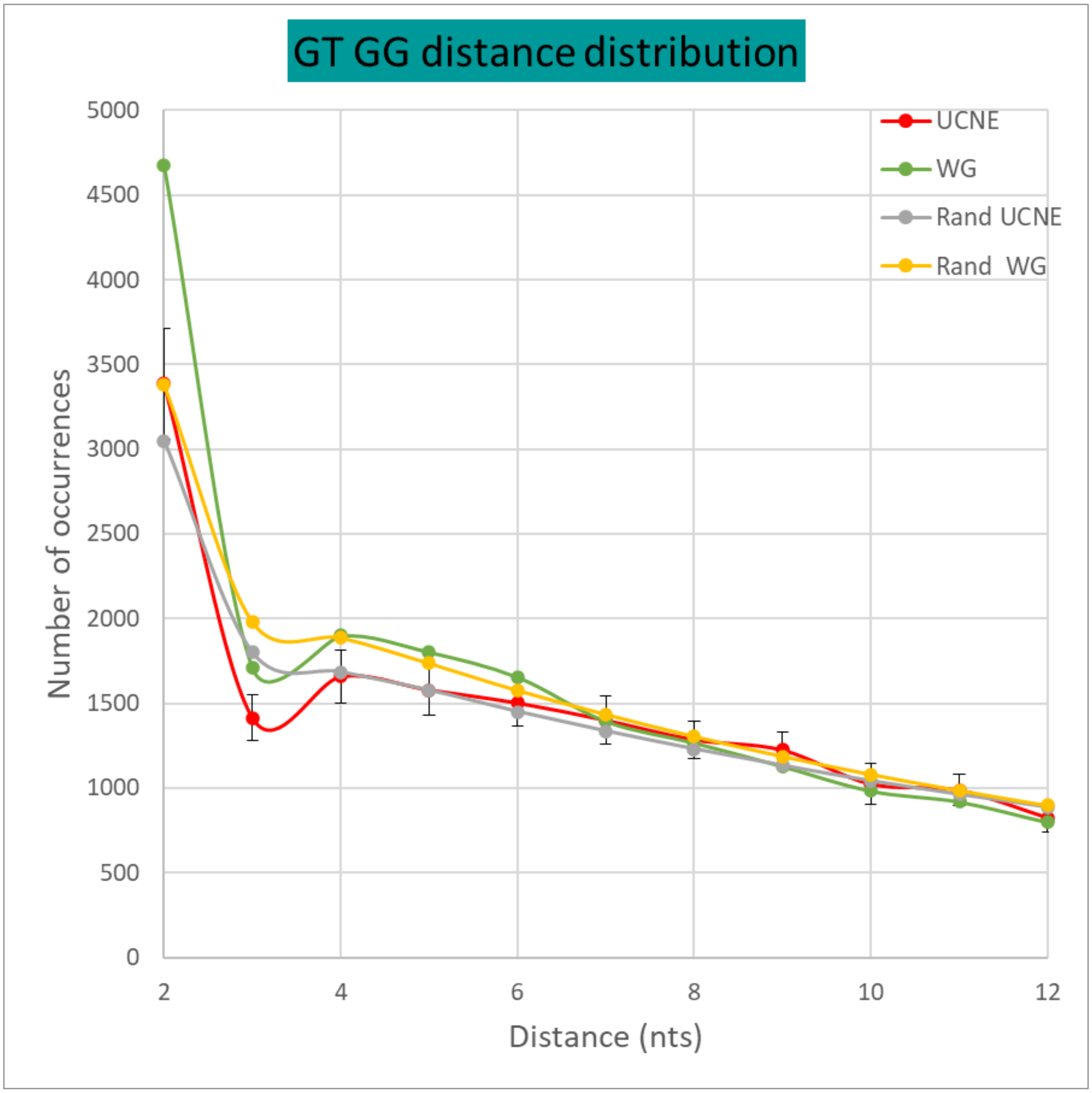
GT GG distance distribution
UCNE
WG
Rand UCNE
Rand WG
Number of occurrences
5000
4500
4000
3500
3000
2500
2000
1500
1000
500
0
2
4
6
8
10
12
Distance (nts)

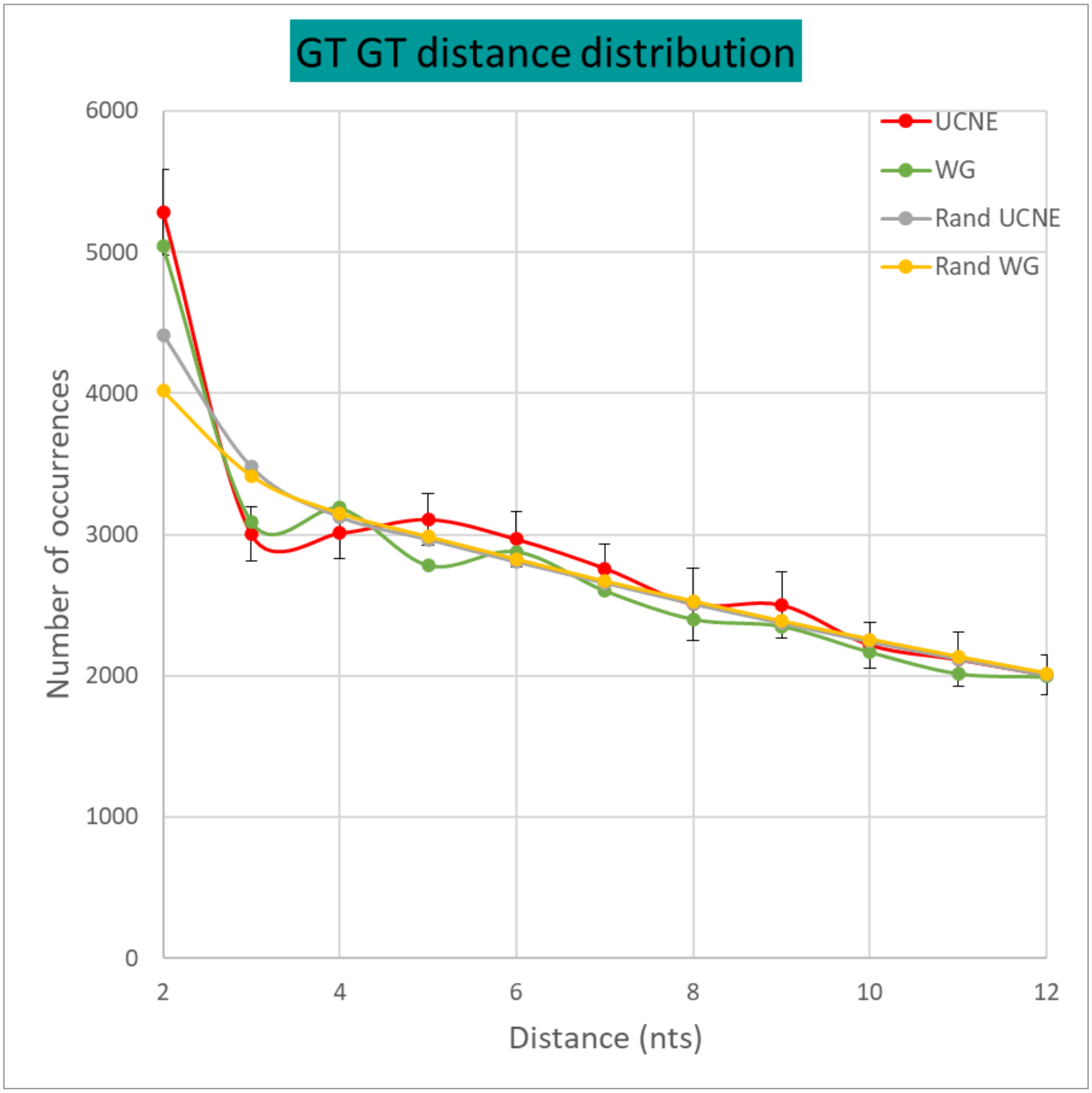

GT GT distance distribution
6000
5000
4000
3000
2000
1000
0
Number of occurrences
2
4
6
8
10
12
Distance (nts)
UCNE
WG
Rand UCNE
Rand WG

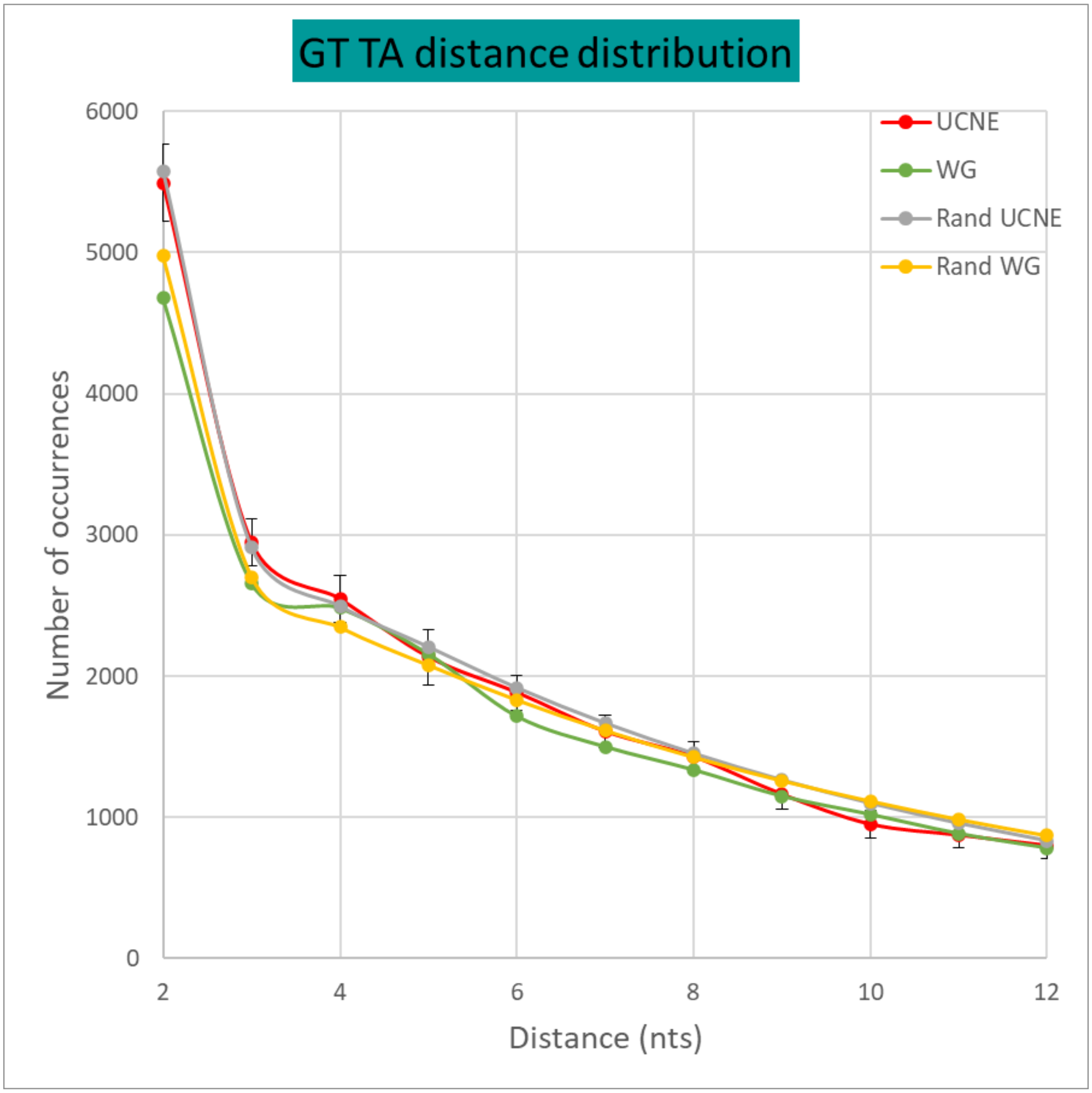
GT TA distance distribution
UCNE
WG
Rand UCNE
Rand WG
Number of occurrences
Distance (nts)
0
1000
2000
3000
4000
5000
6000
2
4
6
8
10
12

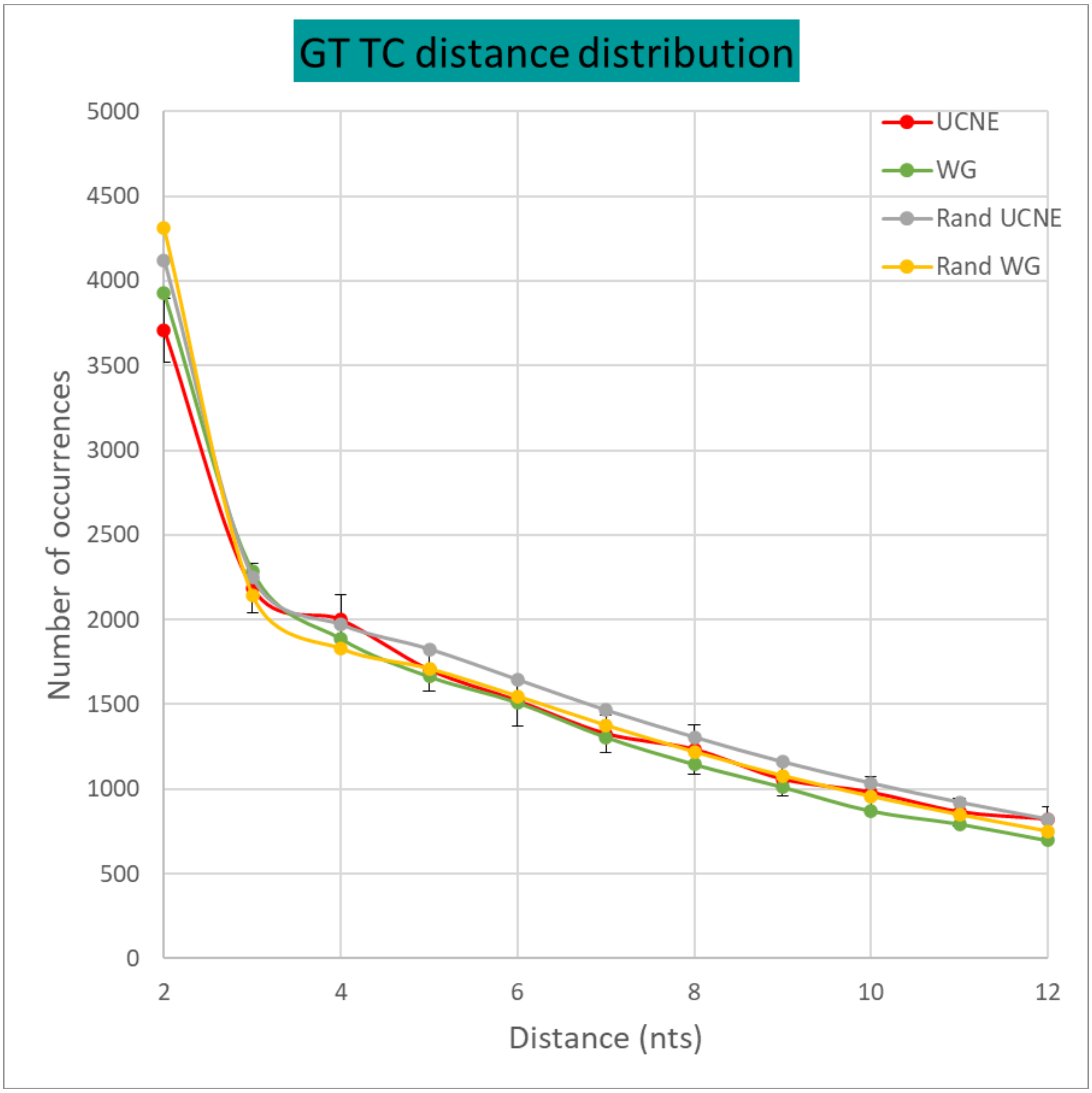

GT TC distance distribution
UCNE
WG
Rand UCNE
Rand WG
Number of occurrences
Distance (nts)
0
500
1000
1500
2000
2500
3000
3500
4000
4500
5000
2
4
6
8
10
12

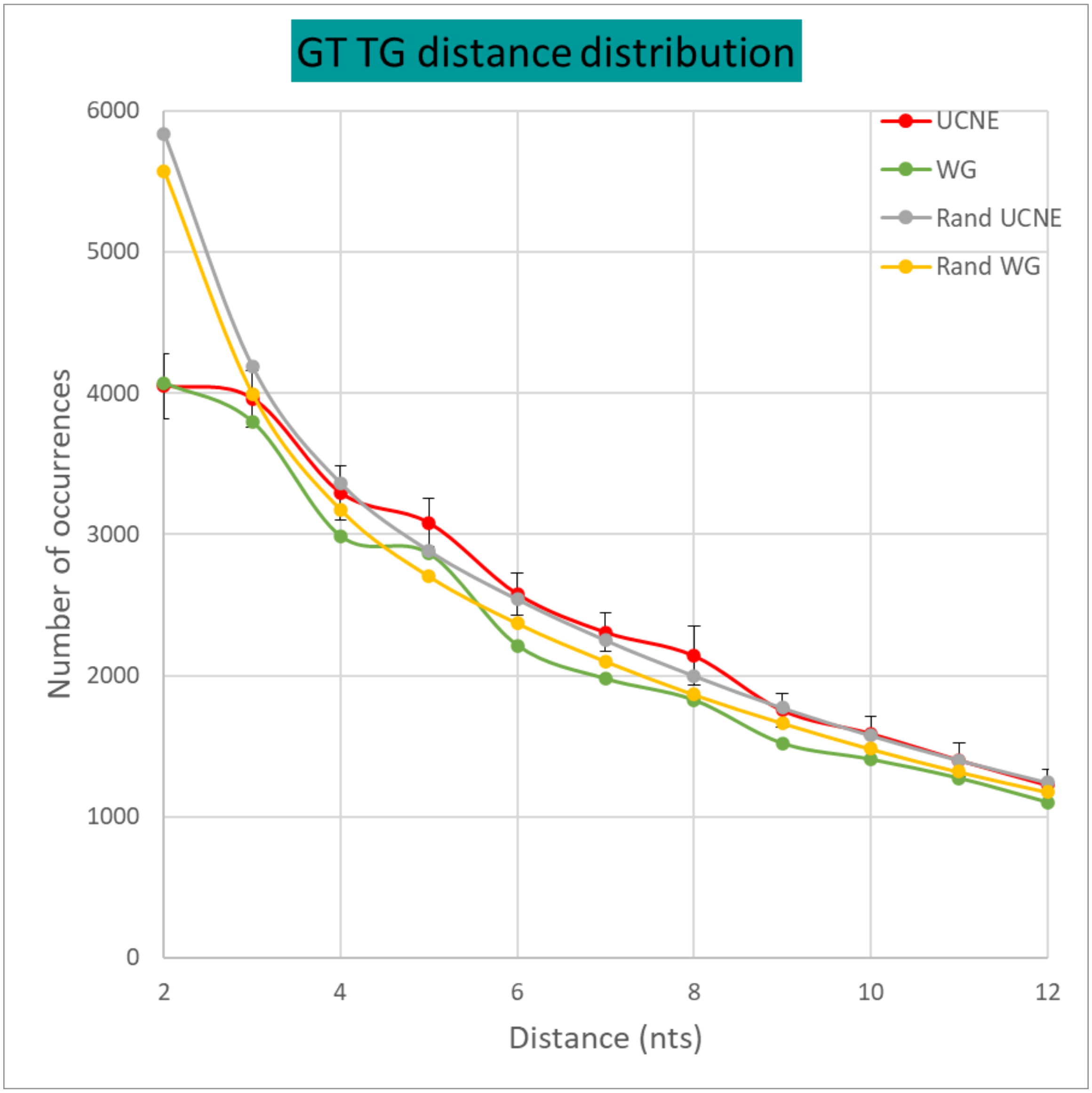

GT TG distance distribution
UCNE
WG
Rand UCNE
Rand WG
Number of occurrences
6000
5000
4000
3000
2000
1000
0
2
4
6
8
10
12
Distance (nts)

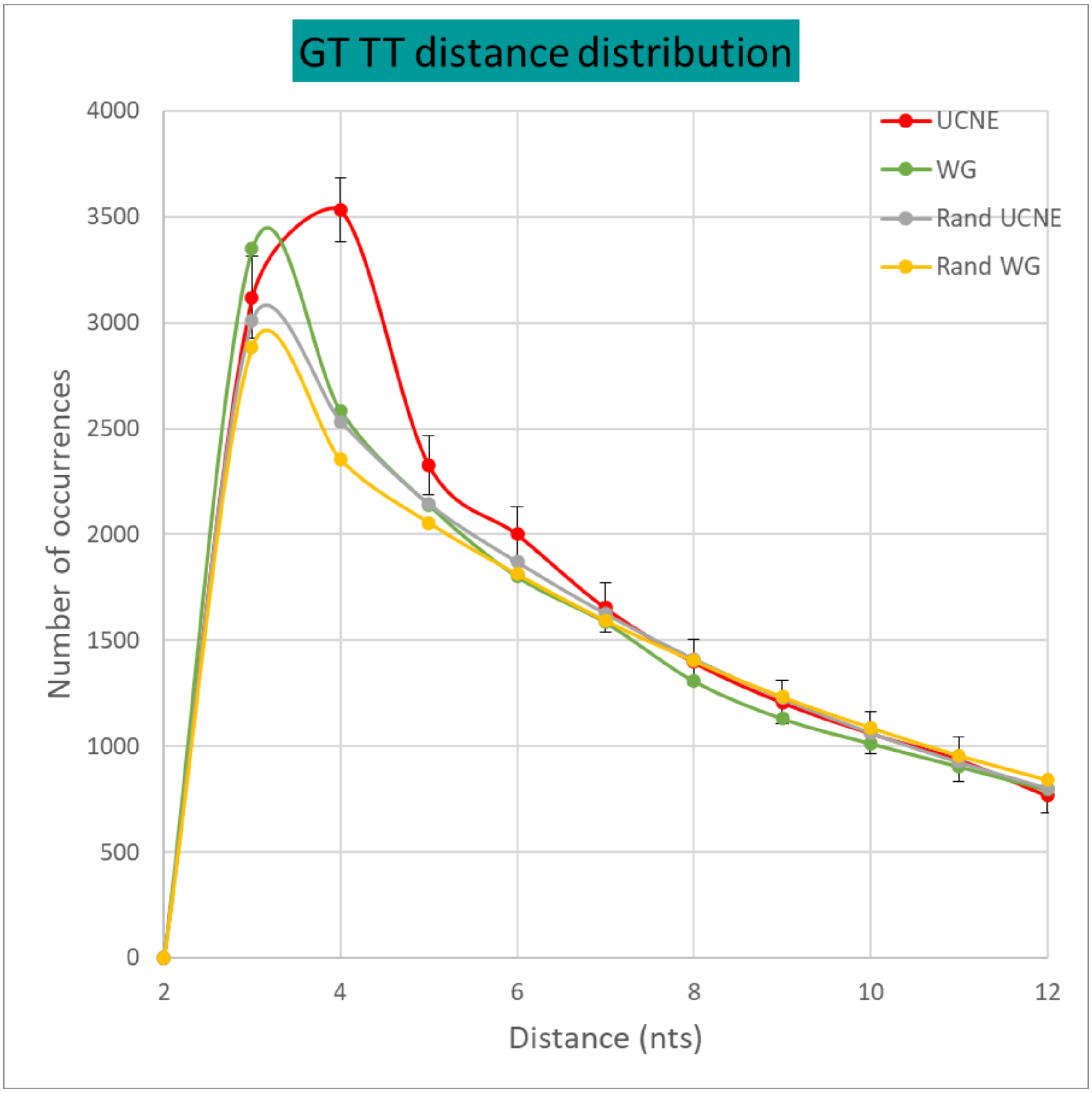

GT TT distance distribution
4000
3500
3000
2500
2000
1500
1000
500
0
Number of occurrences
2
4
6
8
10
12
Distance (nts)
UCNE
WG
Rand UCNE
Rand WG

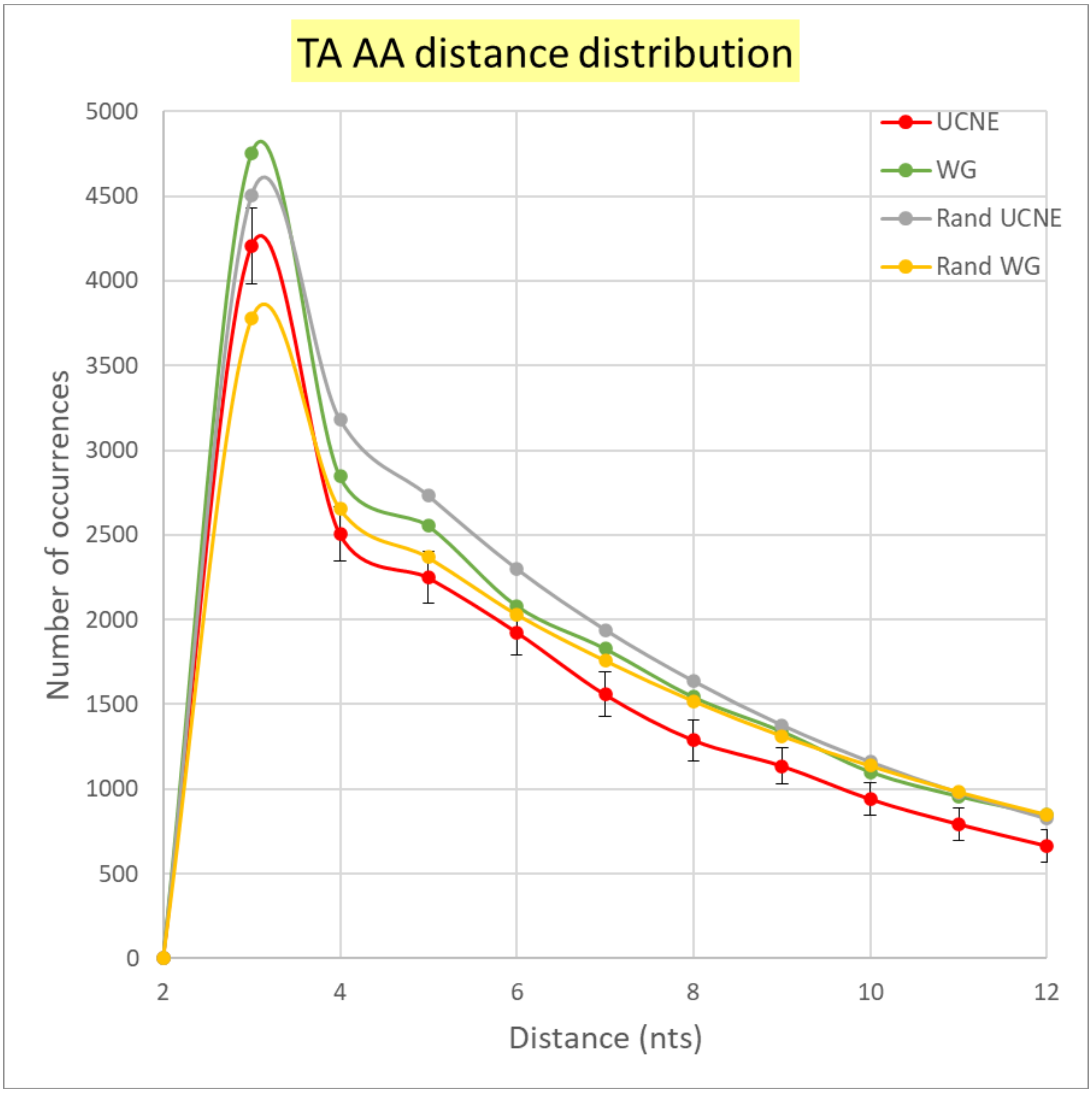
TA AA distance distribution
UCNE
WG
Rand UCNE
Rand WG
Number of occurrences
Distance (nts)
5000
4500
4000
3500
3000
2500
2000
1500
1000
500
0
2
4
6
8
10
12

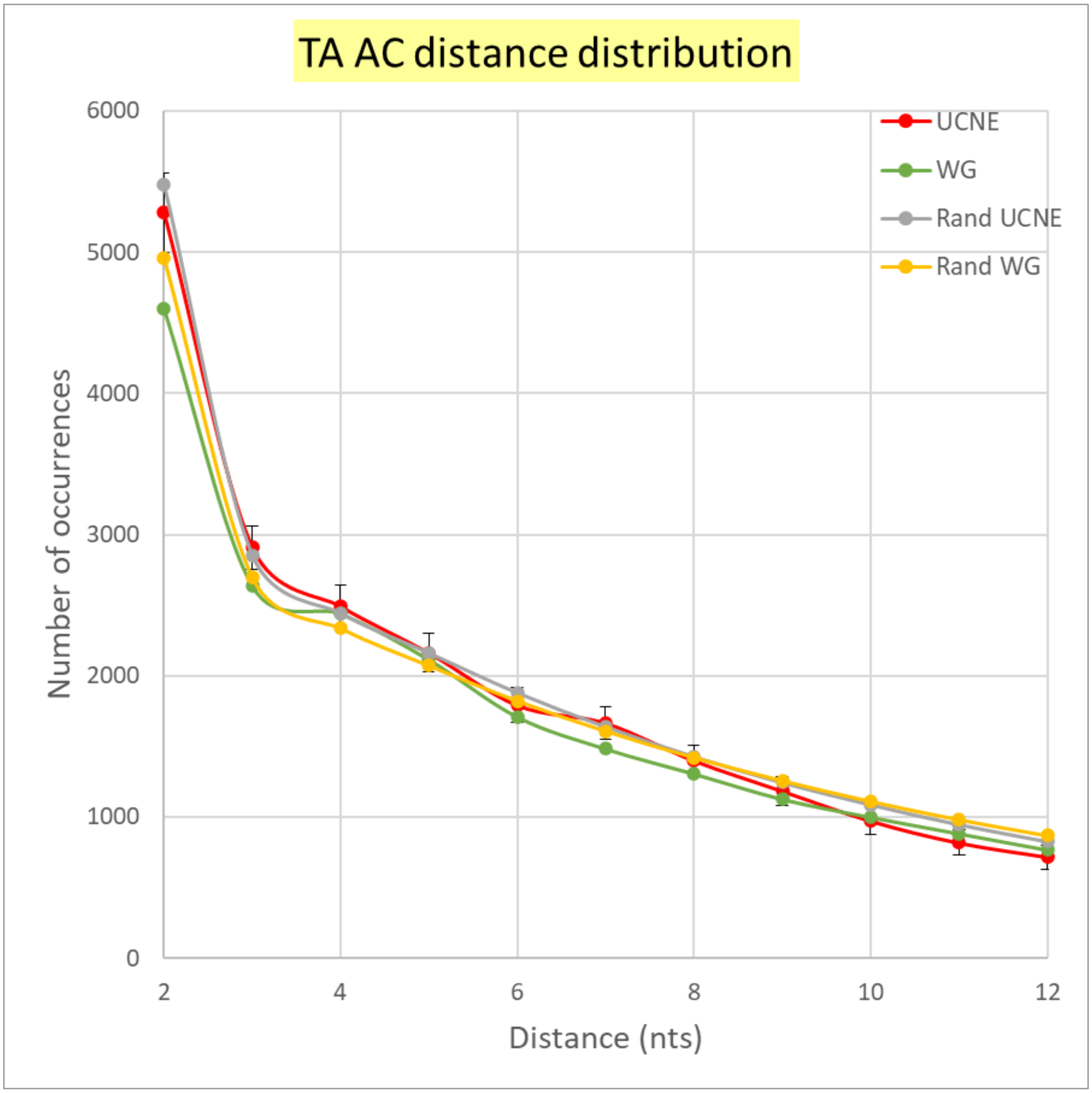
TA AC distance distribution
Number of occurrences
Distance (nts)
UCNE
WG
Rand UCNE
Rand WG
0
1000
2000
3000
4000
5000
6000
2
4
6
8
10
12

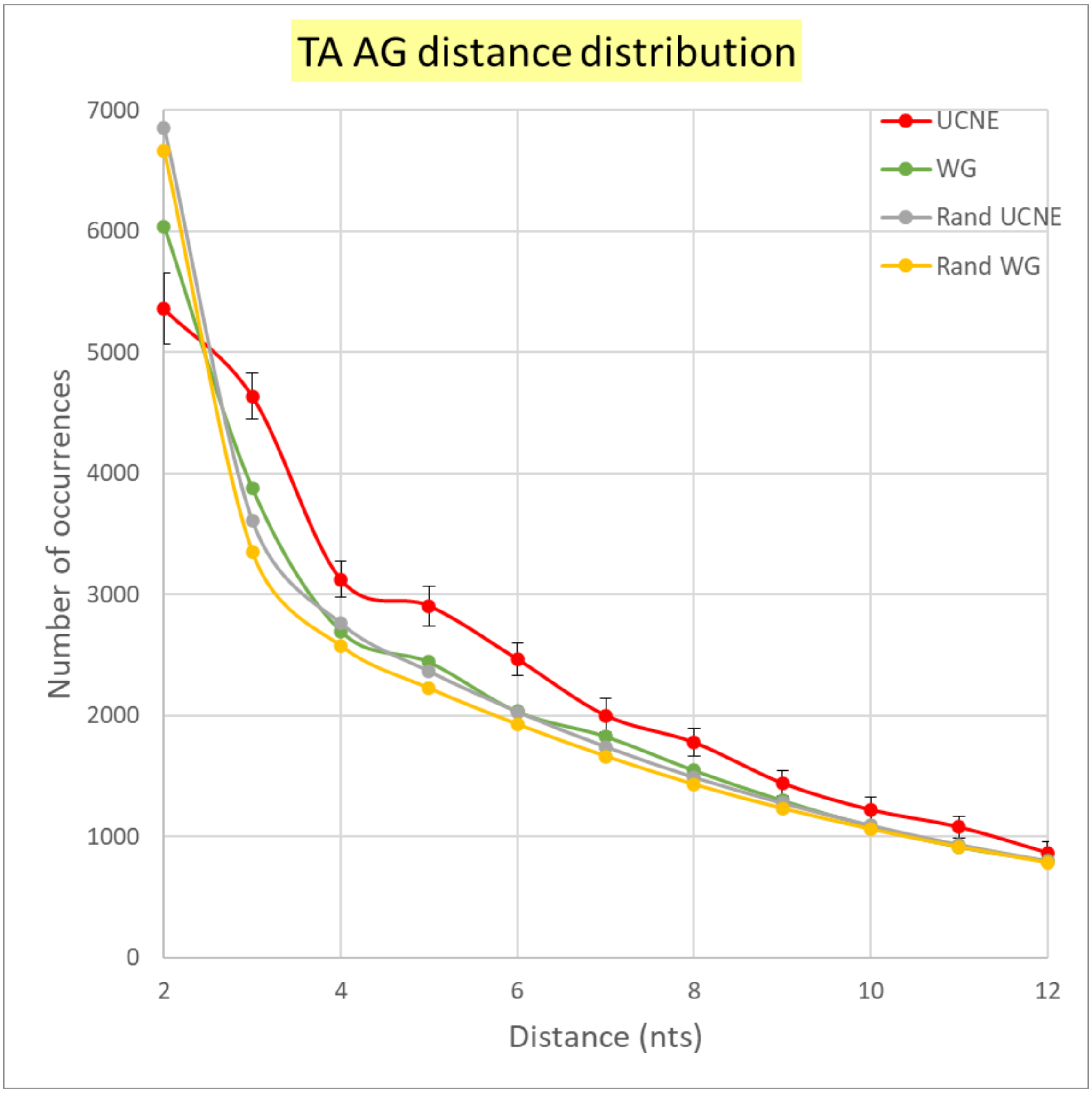

TA AG distance distribution
UCNE
WG
Rand UCNE
Rand WG
Number of occurrences
7000
6000
5000
4000
3000
2000
1000
0
2
4
6
8
10
12
Distance (nts)

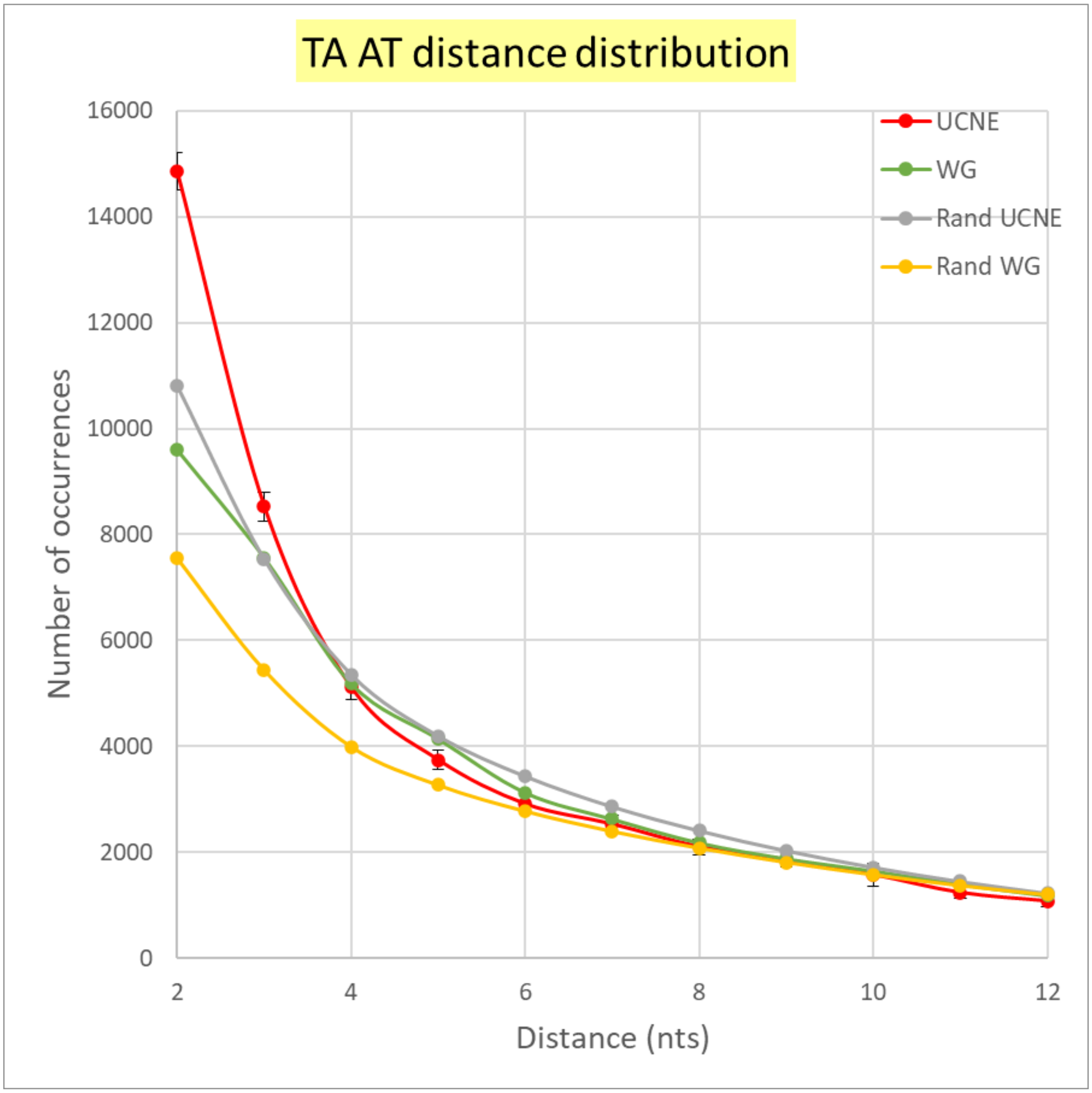

TA AT distance distribution
16000
14000
12000
10000
8000
6000
4000
2000
0
2
4
6
8
10
12
Number of occurrences
Distance (nts)
UCNE
WG
Rand UCNE
Rand WG

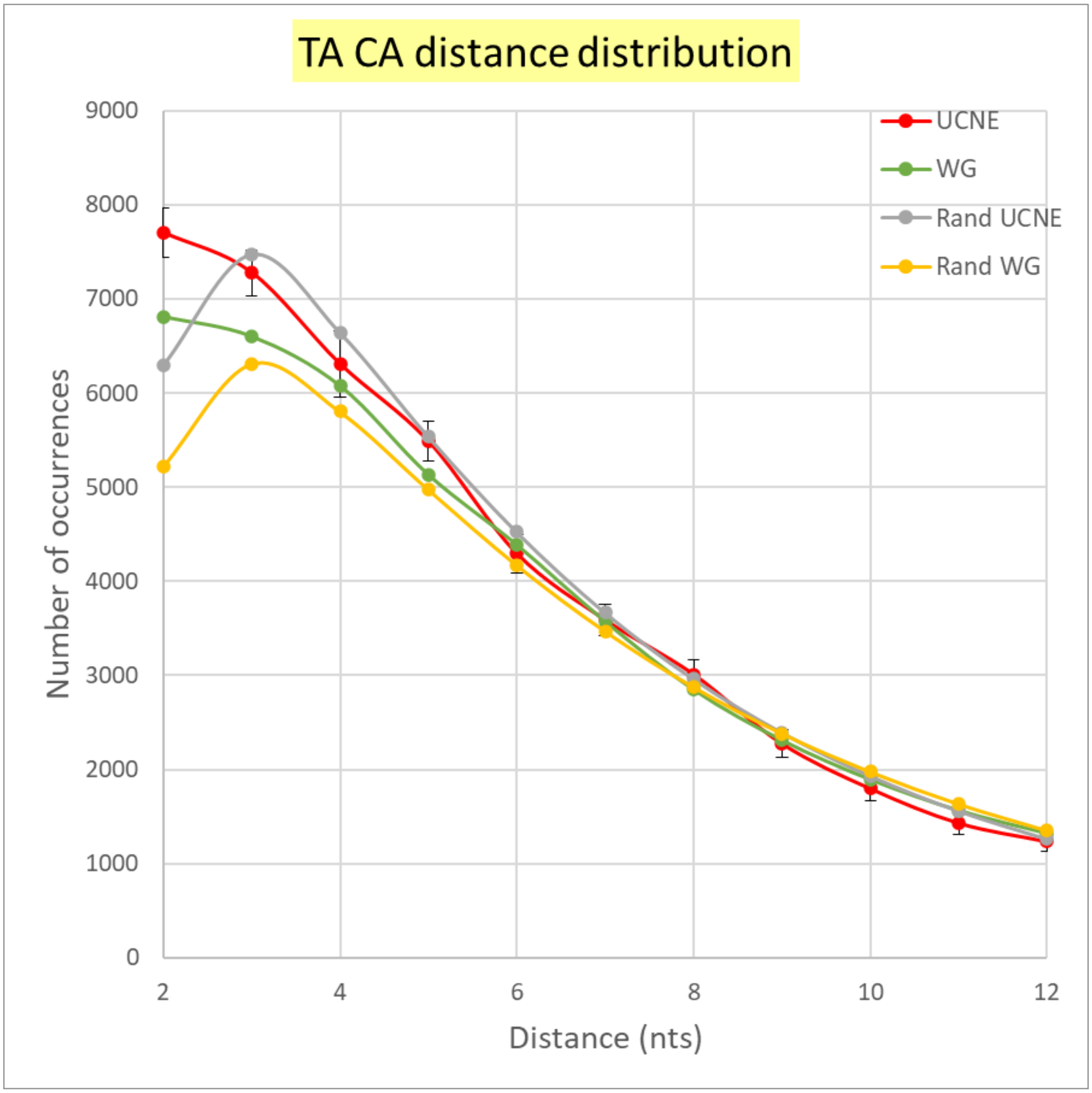

TA CA distance distribution
UCNE
WG
Rand UCNE
Rand WG
Number of occurrences
9000
8000
7000
6000
5000
4000
3000
2000
1000
0
2
4
6
8
10
12
Distance (nts)

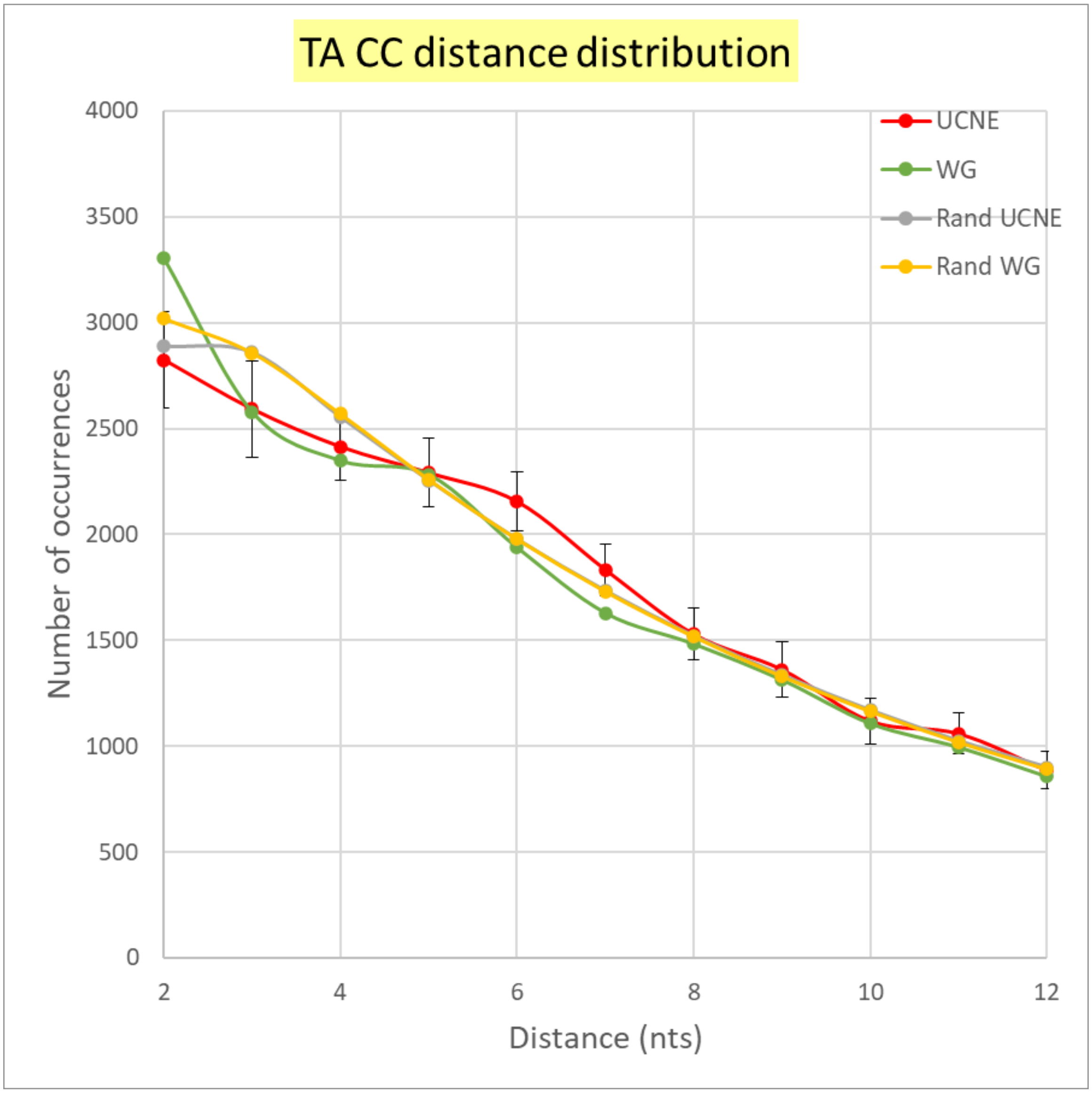

TA CC distance distribution
UCNE
WG
Rand UCNE
Rand WG
Number of occurrences
0
500
1000
1500
2000
2500
3000
3500
4000
2
4
6
8
10
12
Distance (nts)

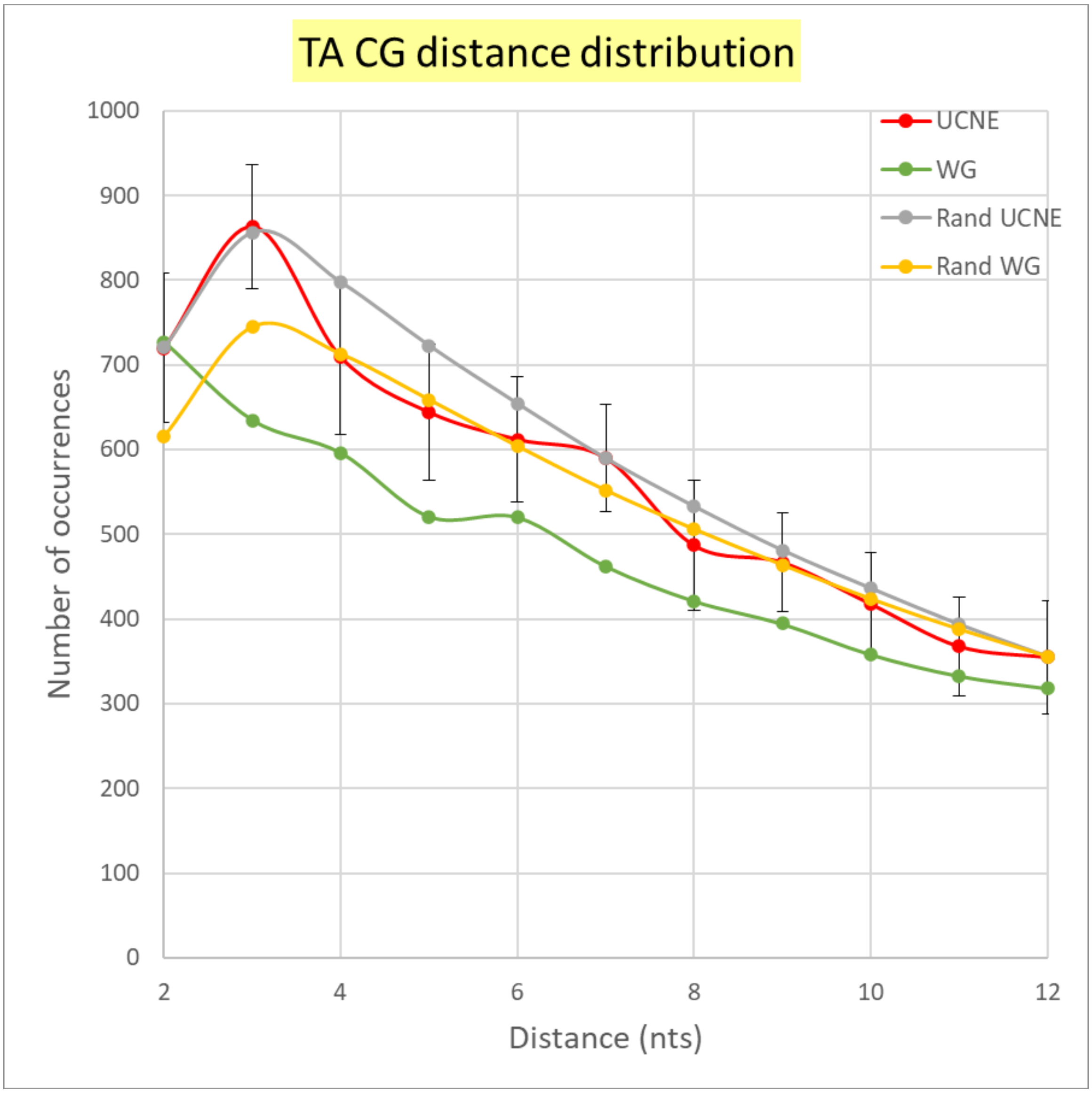

TA CG distance distribution
Number of occurrences
Distance (nts)
UCNE
WG
Rand UCNE
Rand WG
0
100
200
300
400
500
600
700
800
900
1000
2
4
6
8
10
12

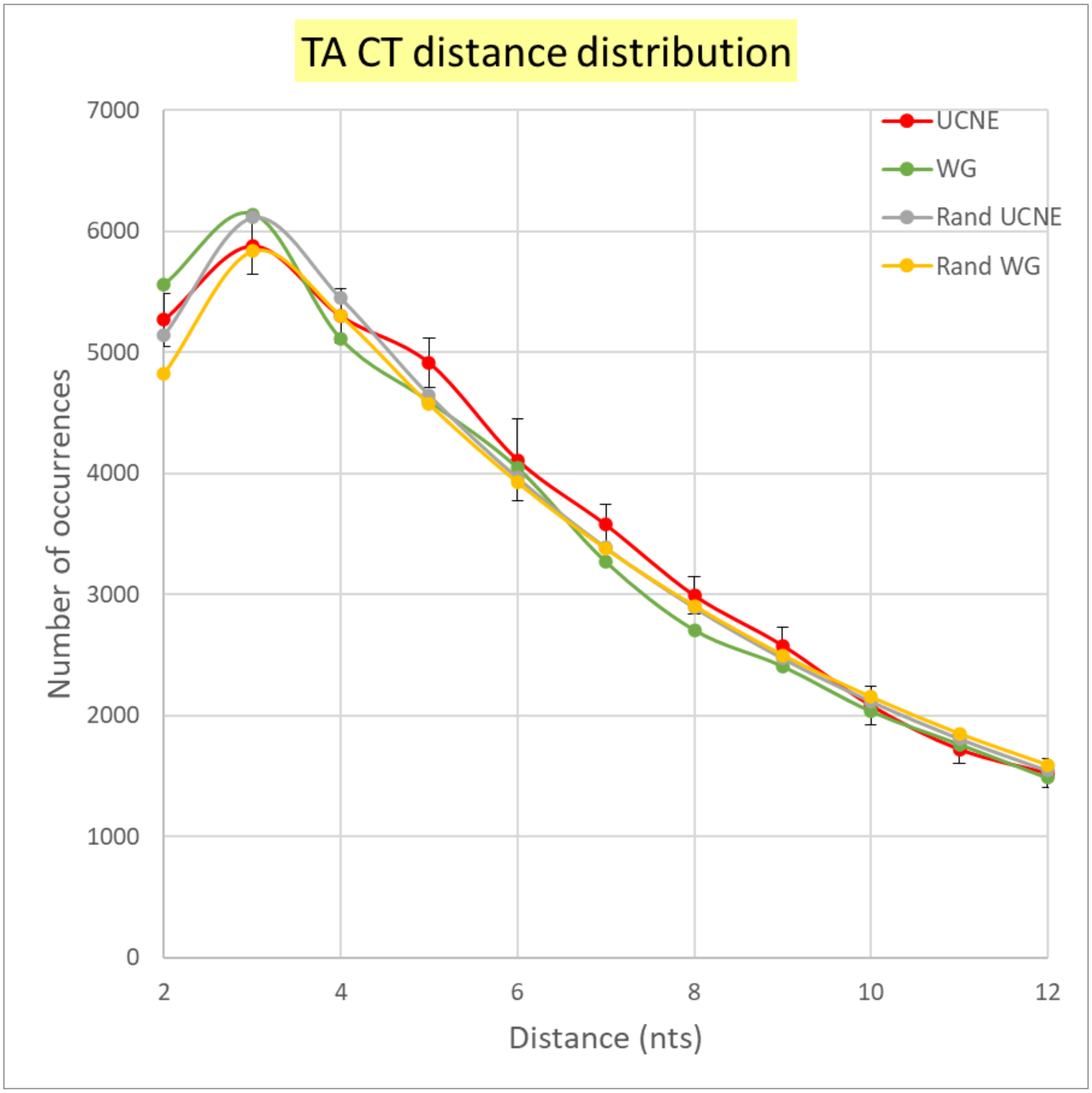

TA CT distance distribution
UCNE
WG
Rand UCNE
Rand WG
7000
6000
5000
4000
3000
2000
1000
0
Number of occurrences
2
4
6
8
10
12
Distance (nts)

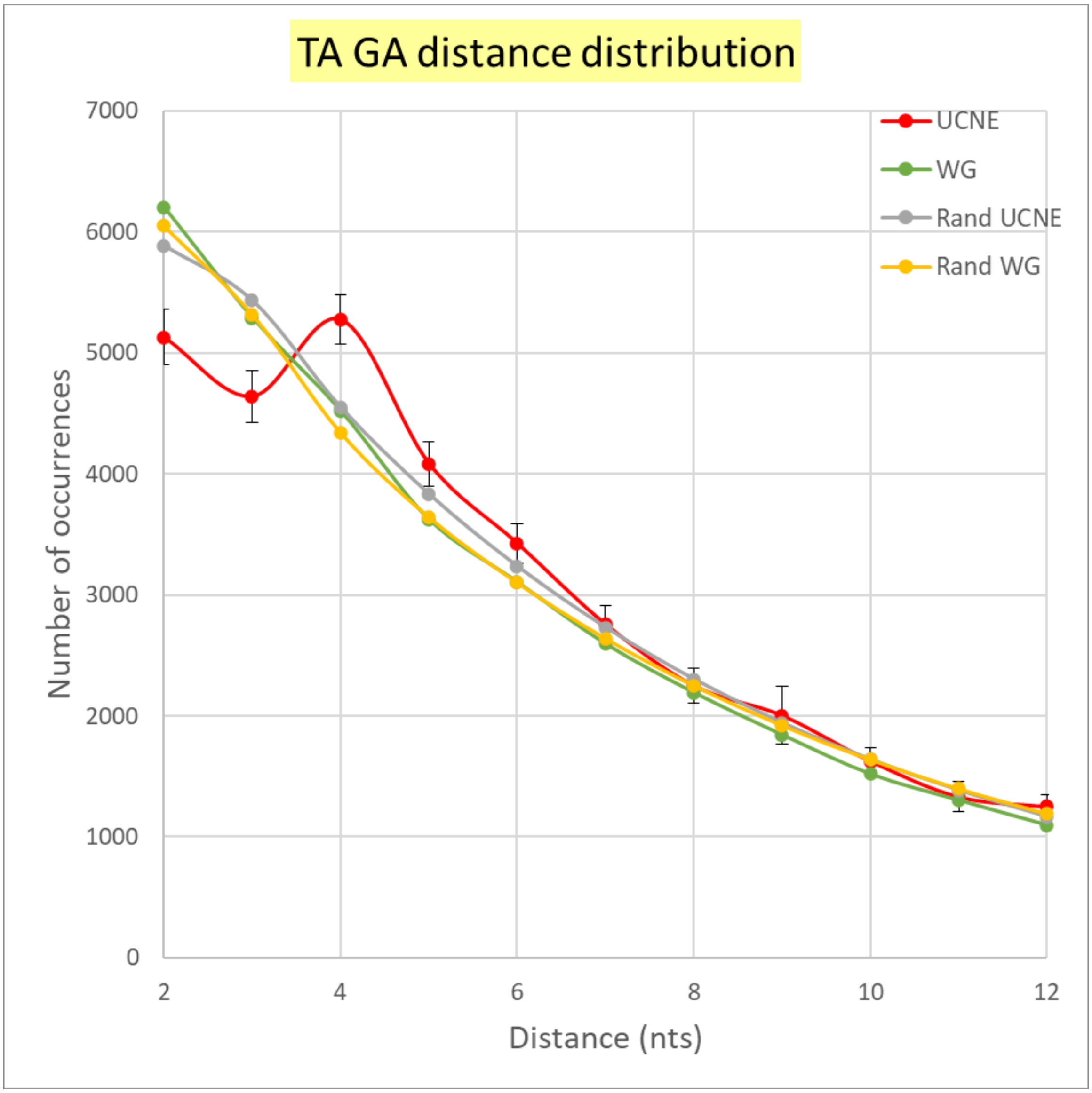

TA GA distance distribution
UCNE
WG
Rand UCNE
Rand WG
Number of occurrences
Distance (nts)
7000
6000
5000
4000
3000
2000
1000
0
2
4
6
8
10
12

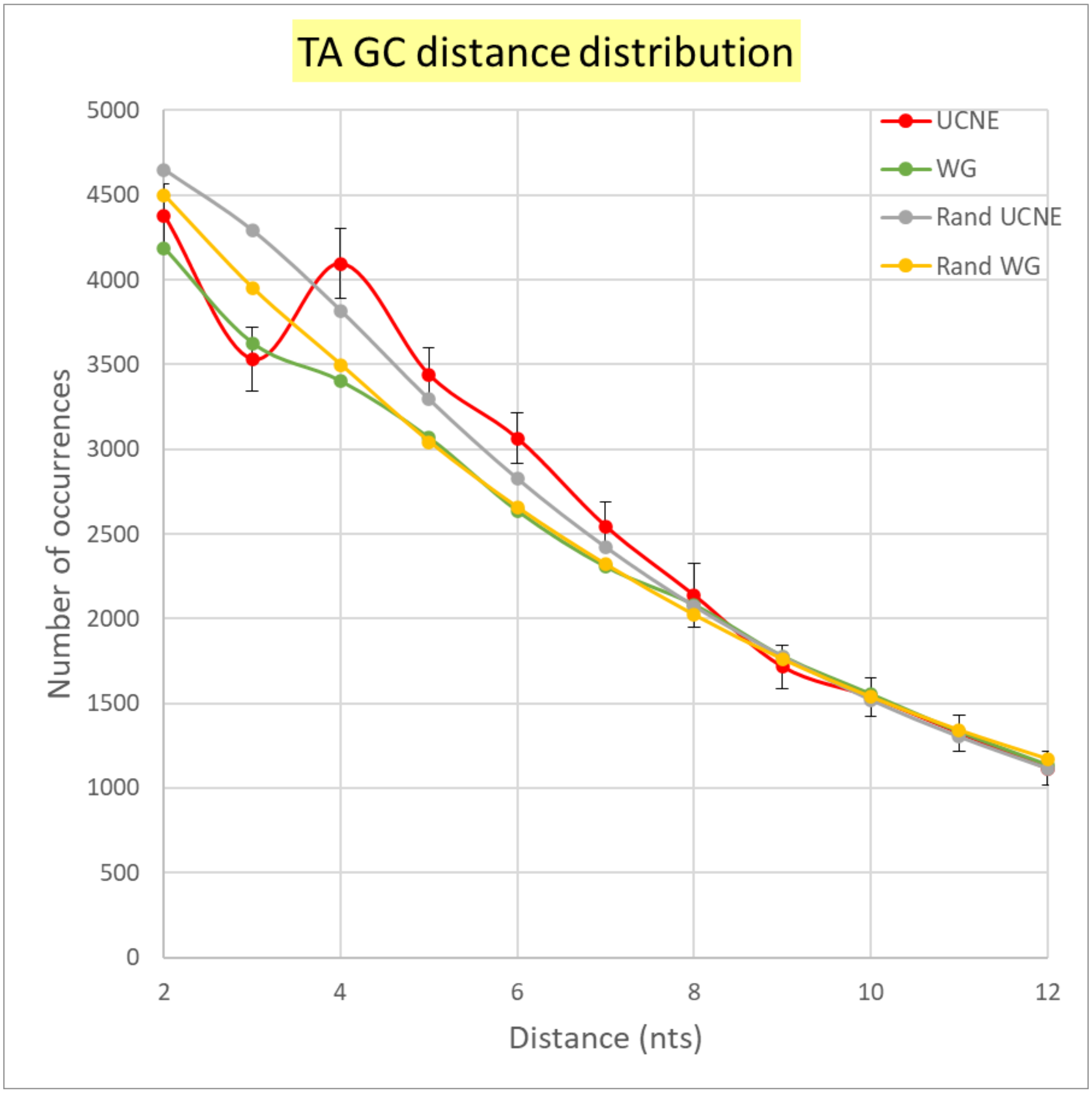
TA GC distance distribution
UCNE
WG
Rand UCNE
Rand WG
Number of occurrences
5000
4500
4000
3500
3000
2500
2000
1500
1000
500
0
2
4
6
8
10
12
Distance (nts)

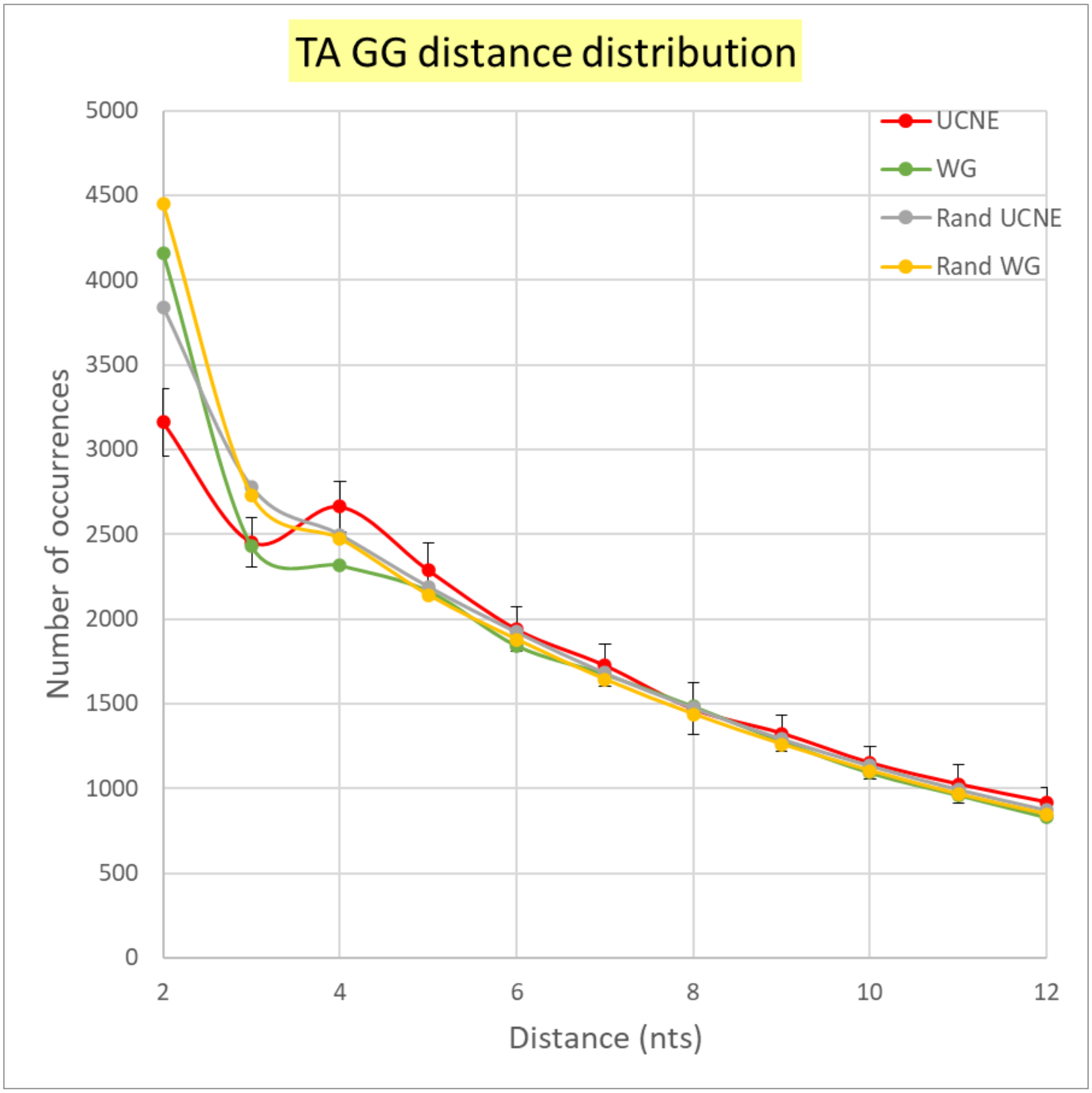

TA GG distance distribution
Number of occurrences
5000
4500
4000
3500
3000
2500
2000
1500
1000
500
0
2
4
6
8
10
12
Distance (nts)
UCNE
WG
Rand UCNE
Rand WG

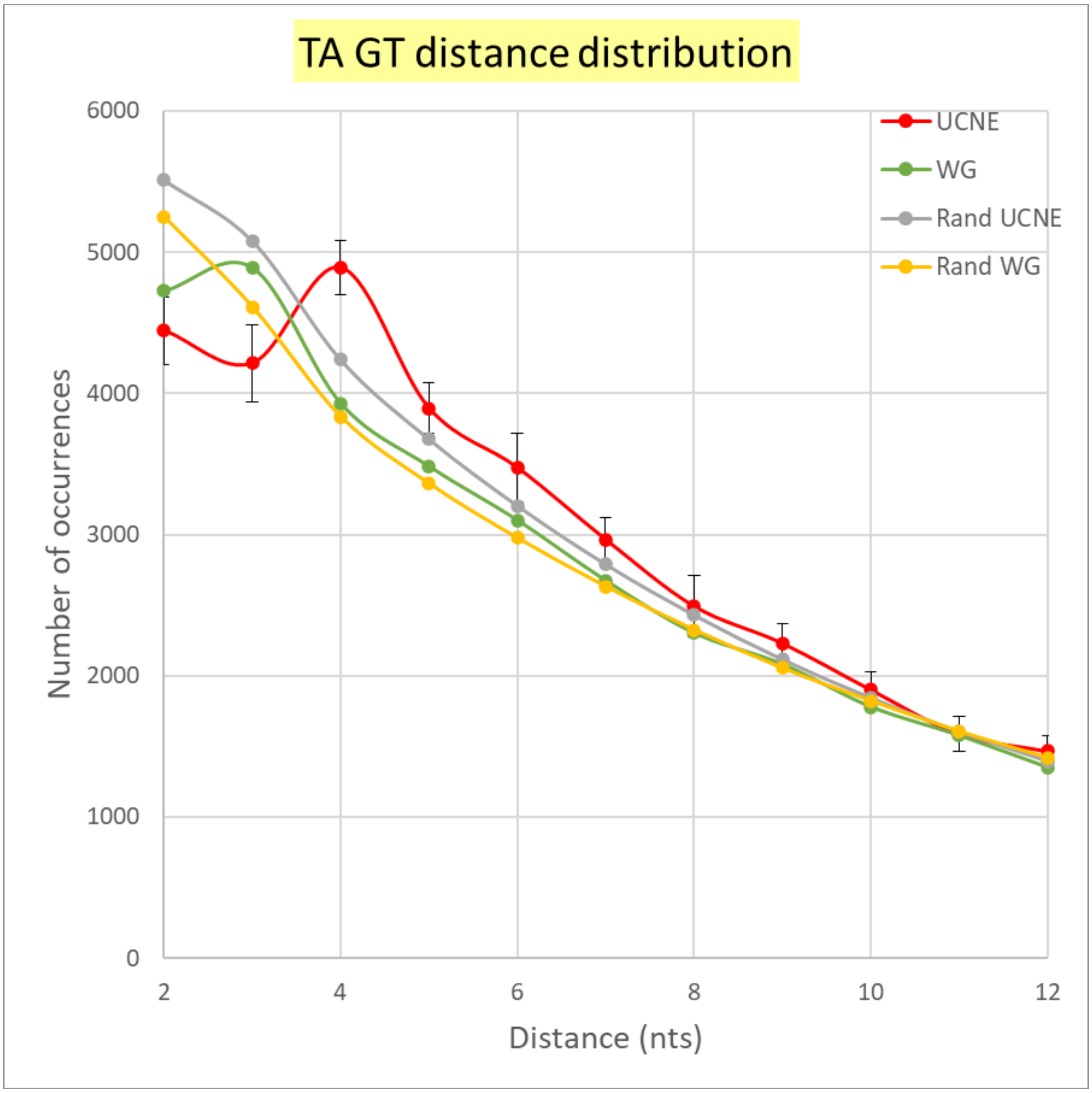

TA GT distance distribution
UCNE
WG
Rand UCNE
Rand WG
Number of occurrences
6000
5000
4000
3000
2000
1000
0
2
4
6
8
10
12
Distance (nts)

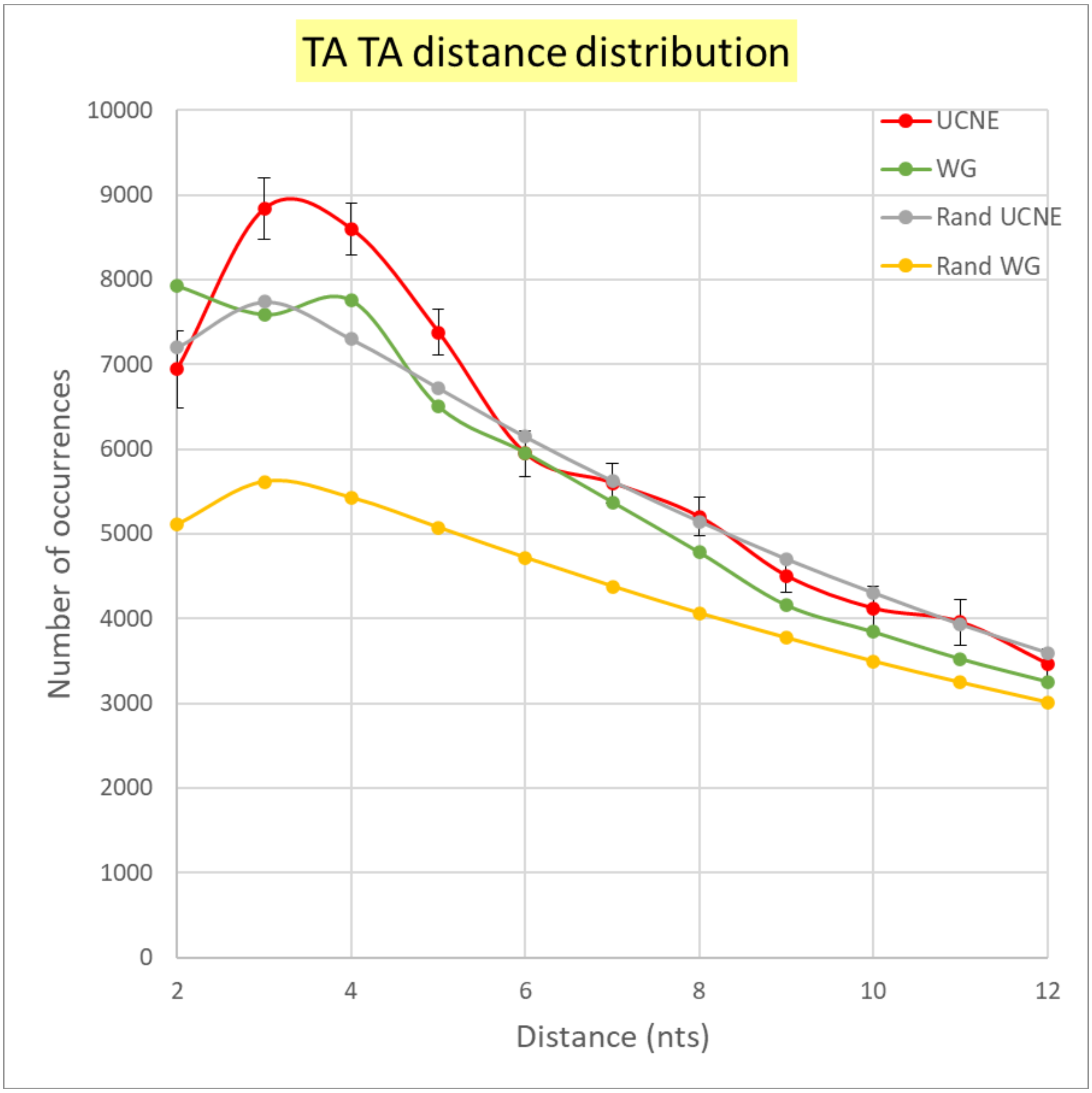

TA TA distance distribution
10000
9000
8000
7000
6000
5000
4000
3000
2000
1000
0
2
4
6
8
10
12
Number of occurrences
Distance (nts)
UCNE
WG
Rand UCNE
Rand WG

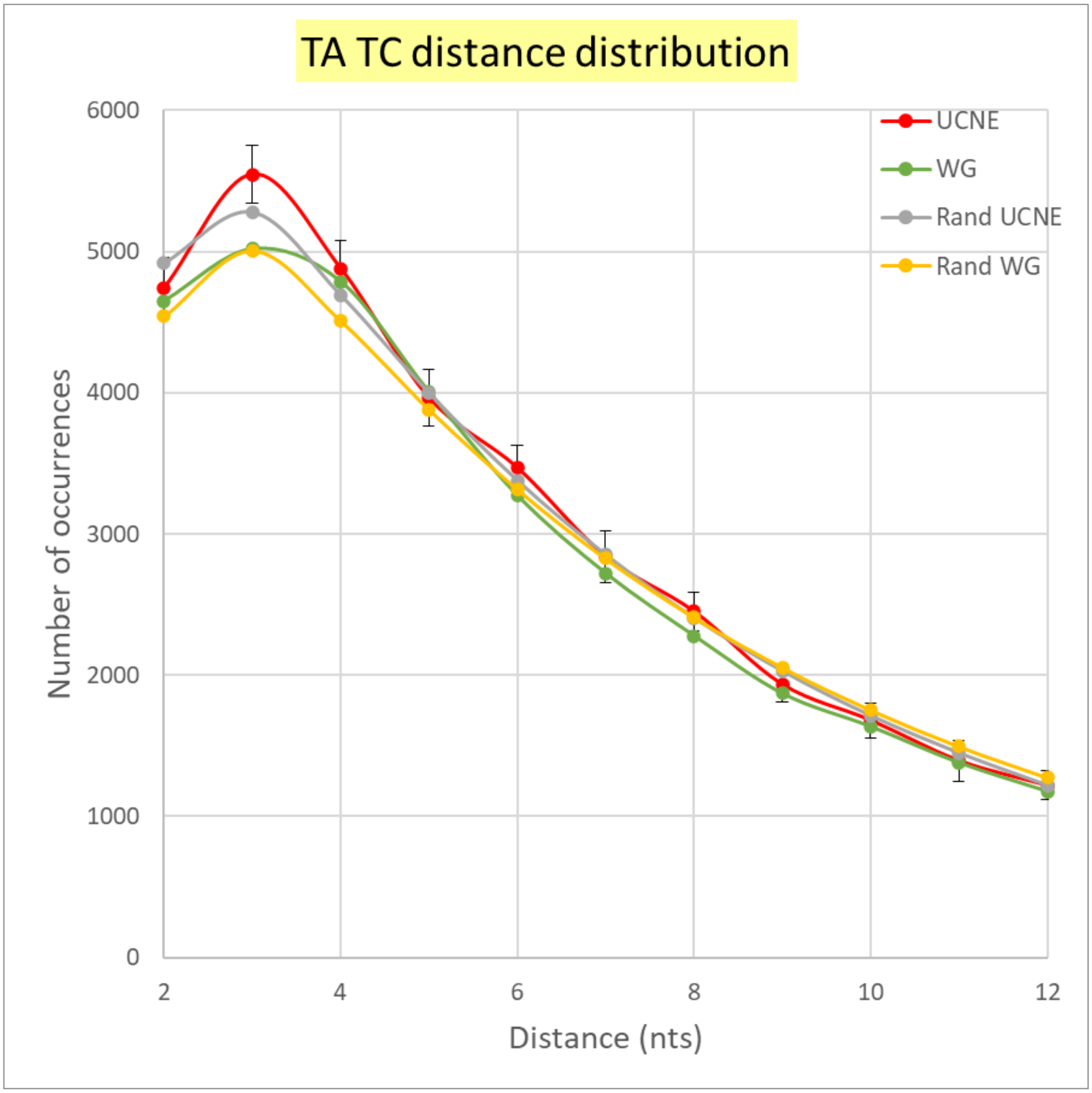

TA TC distance distribution
Number of occurrences
6000
5000
4000
3000
2000
1000
0
2
4
6
8
10
12
Distance (nts)
UCNE
WG
Rand UCNE
Rand WG

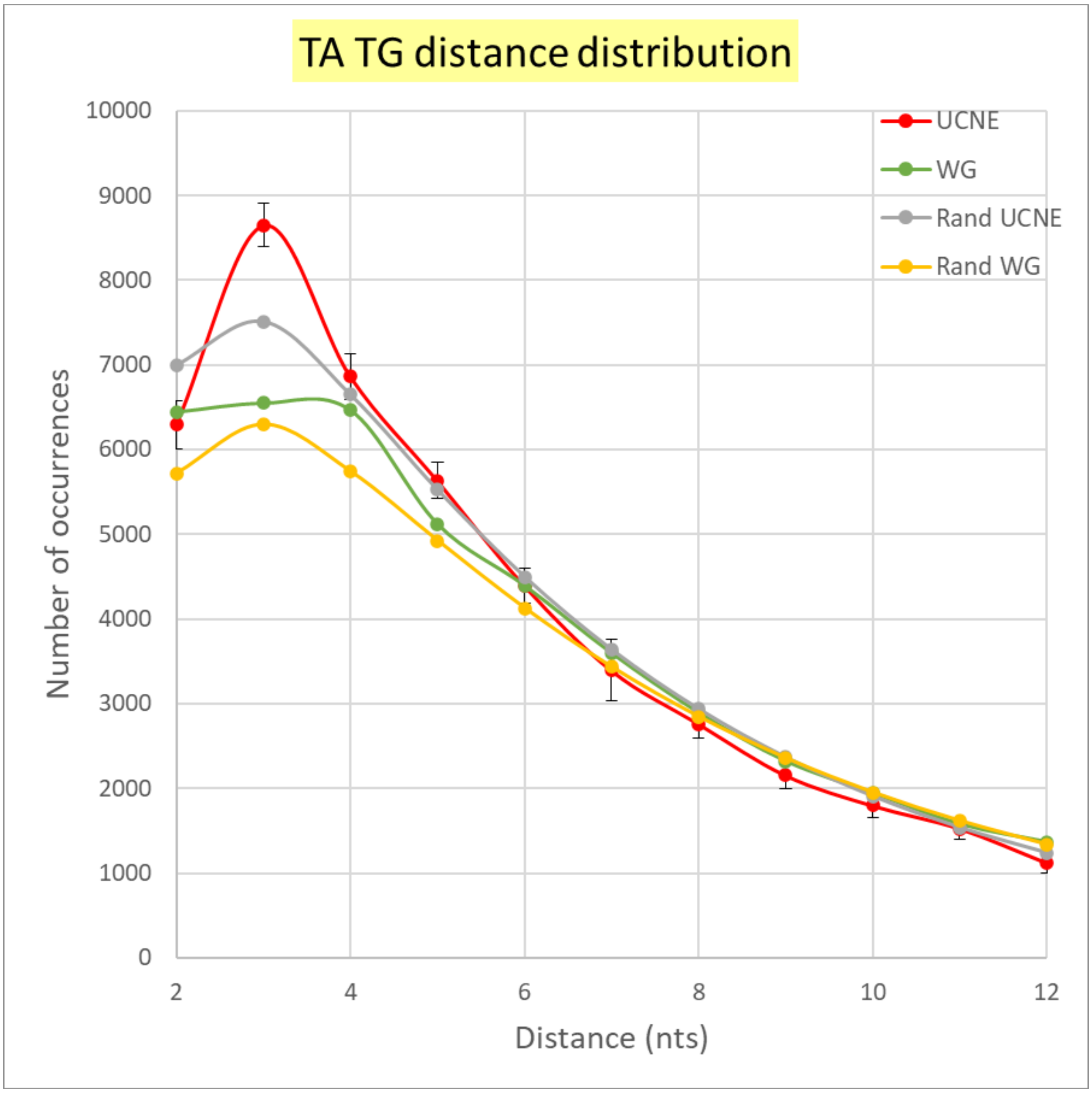
TA TG distance distribution
10000
9000
8000
7000
6000
5000
4000
3000
2000
1000
0
2
4
6
8
10
12
Number of occurrences
Distance (nts)
UCNE
WG
Rand UCNE
Rand WG

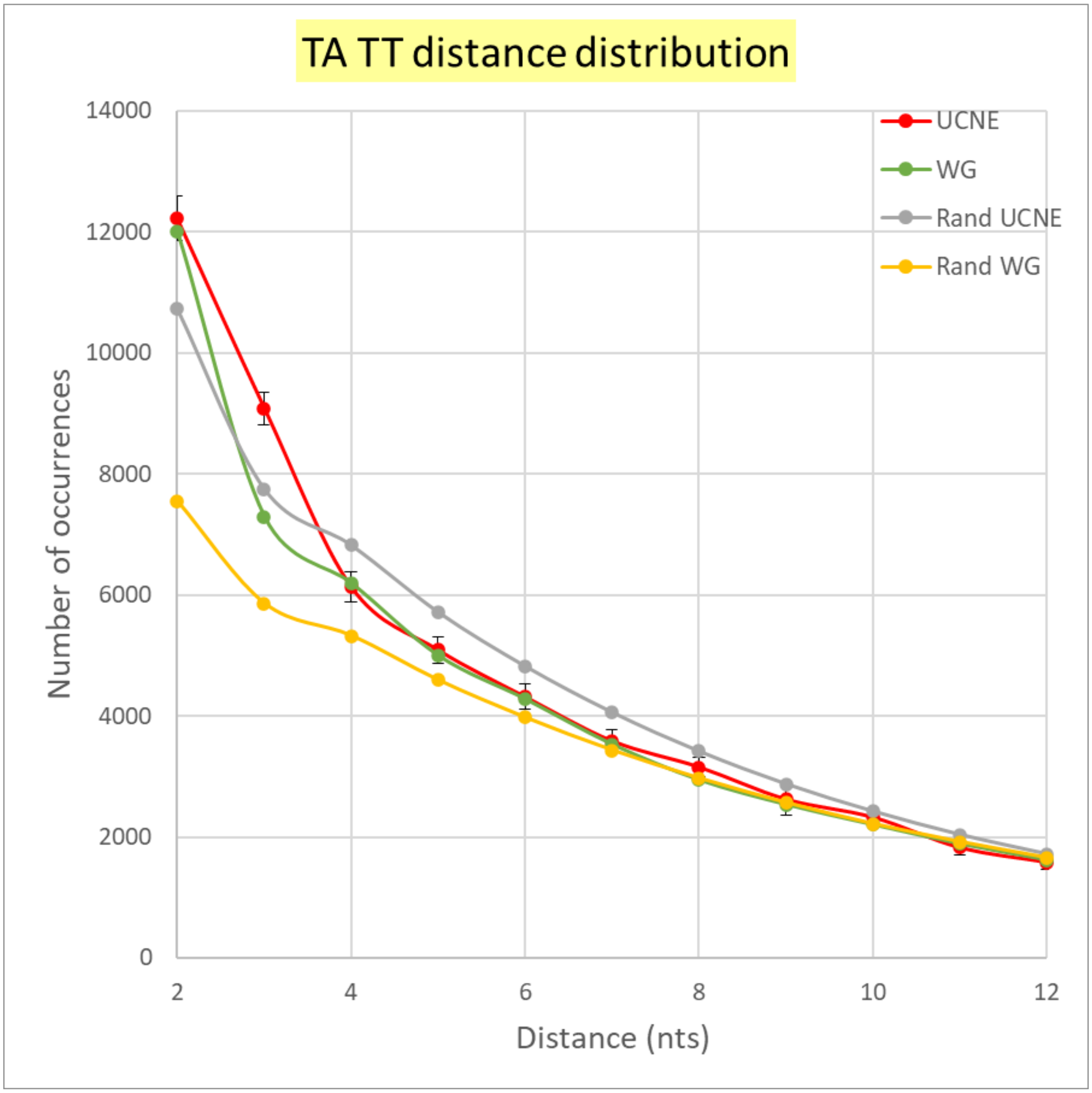
TA TT distance distribution
UCNE
WG
Rand UCNE
Rand WG
Number of occurrences
14000
12000
10000
8000
6000
4000
2000
0
2
4
6
8
10
12
Distance (nts)

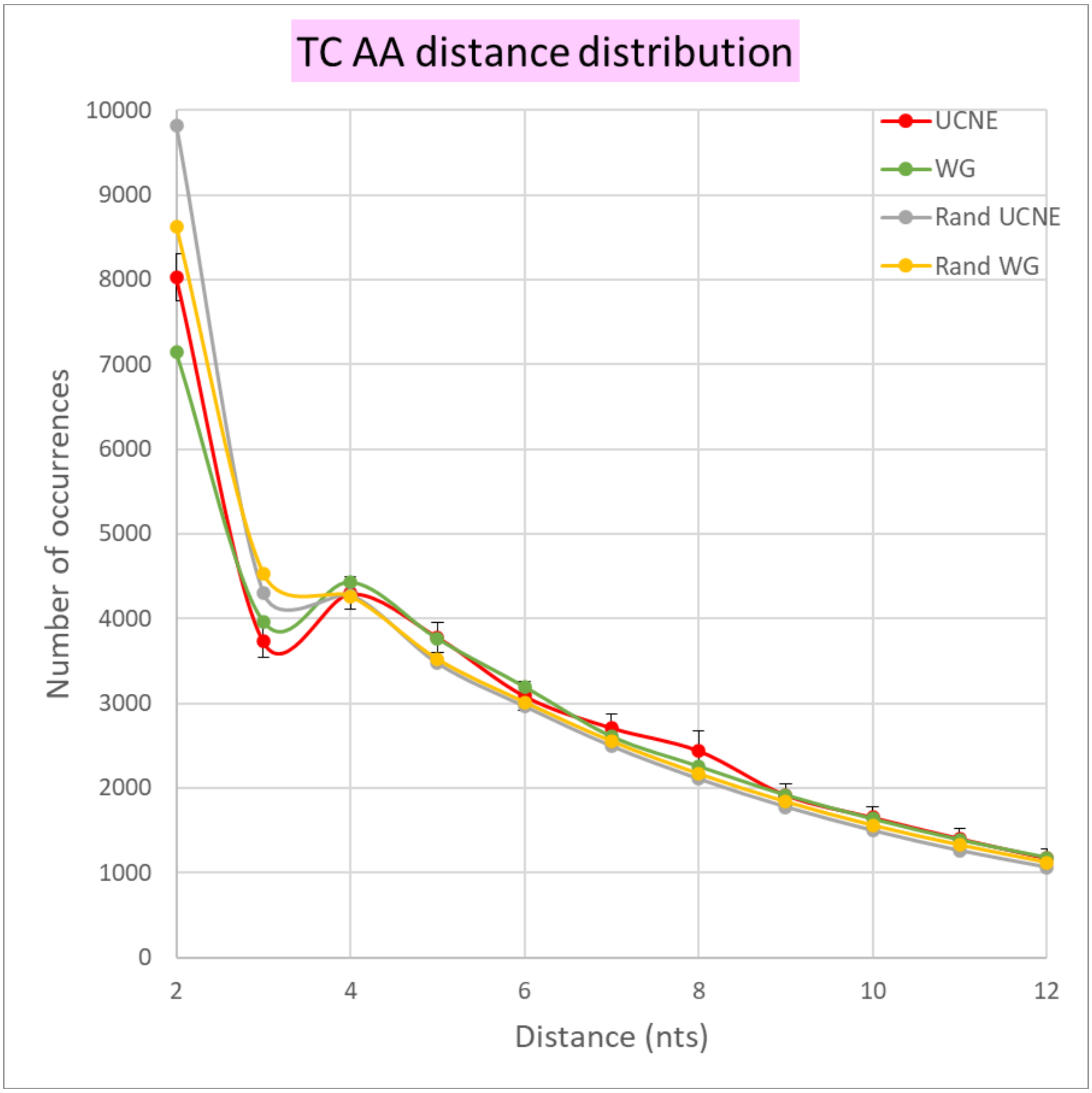
TC AA distance distribution
Number of occurrences
10000
9000
8000
7000
6000
5000
4000
3000
2000
1000
0
2
4
6
8
10
12
Distance (nts)
UCNE
WG
Rand UCNE
Rand WG

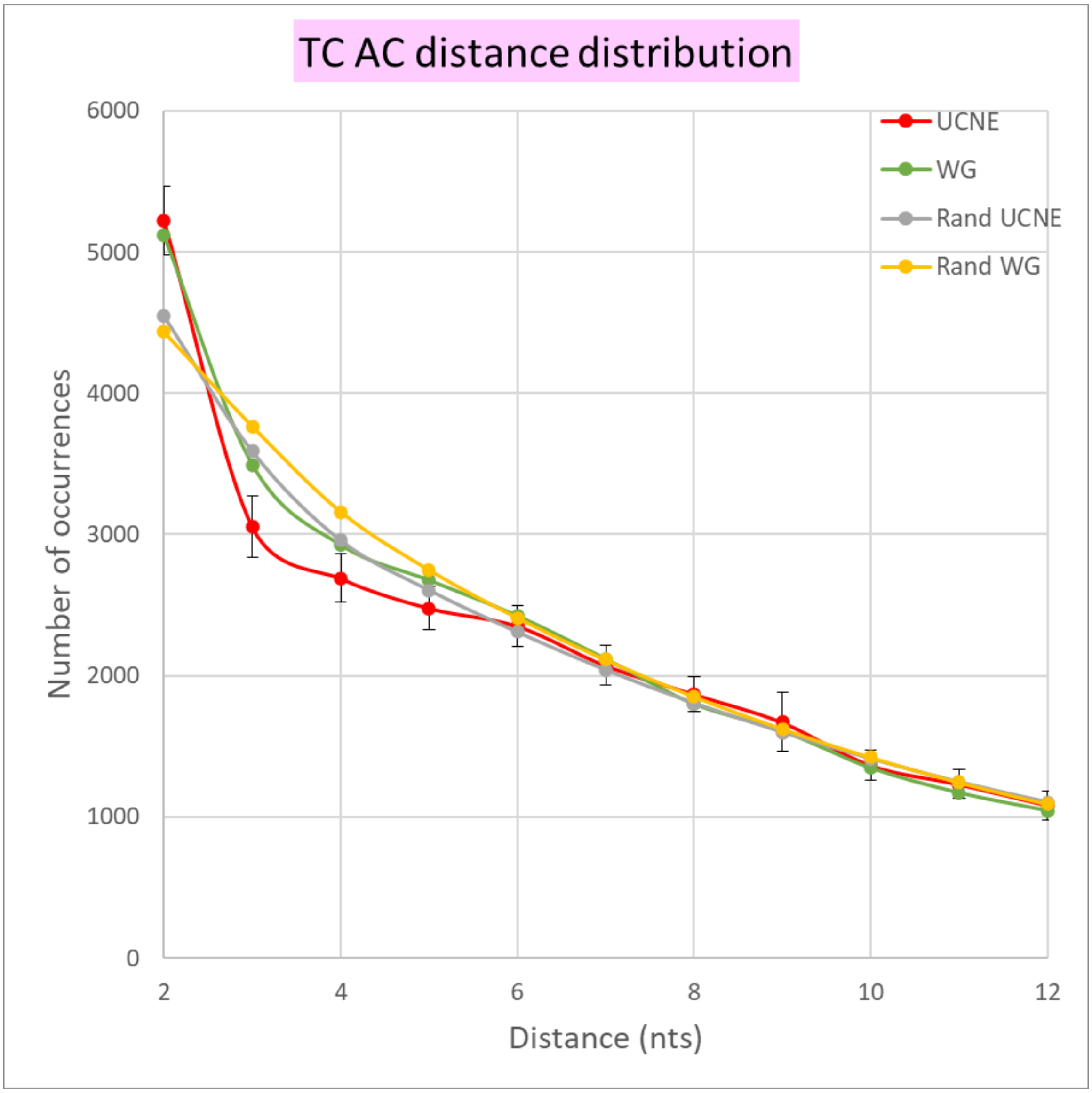

TC AC distance distribution
Number of occurrences
Distance (nts)
6000
5000
4000
3000
2000
1000
0
2
4
6
8
10
12
UCNE
WG
Rand UCNE
Rand WG

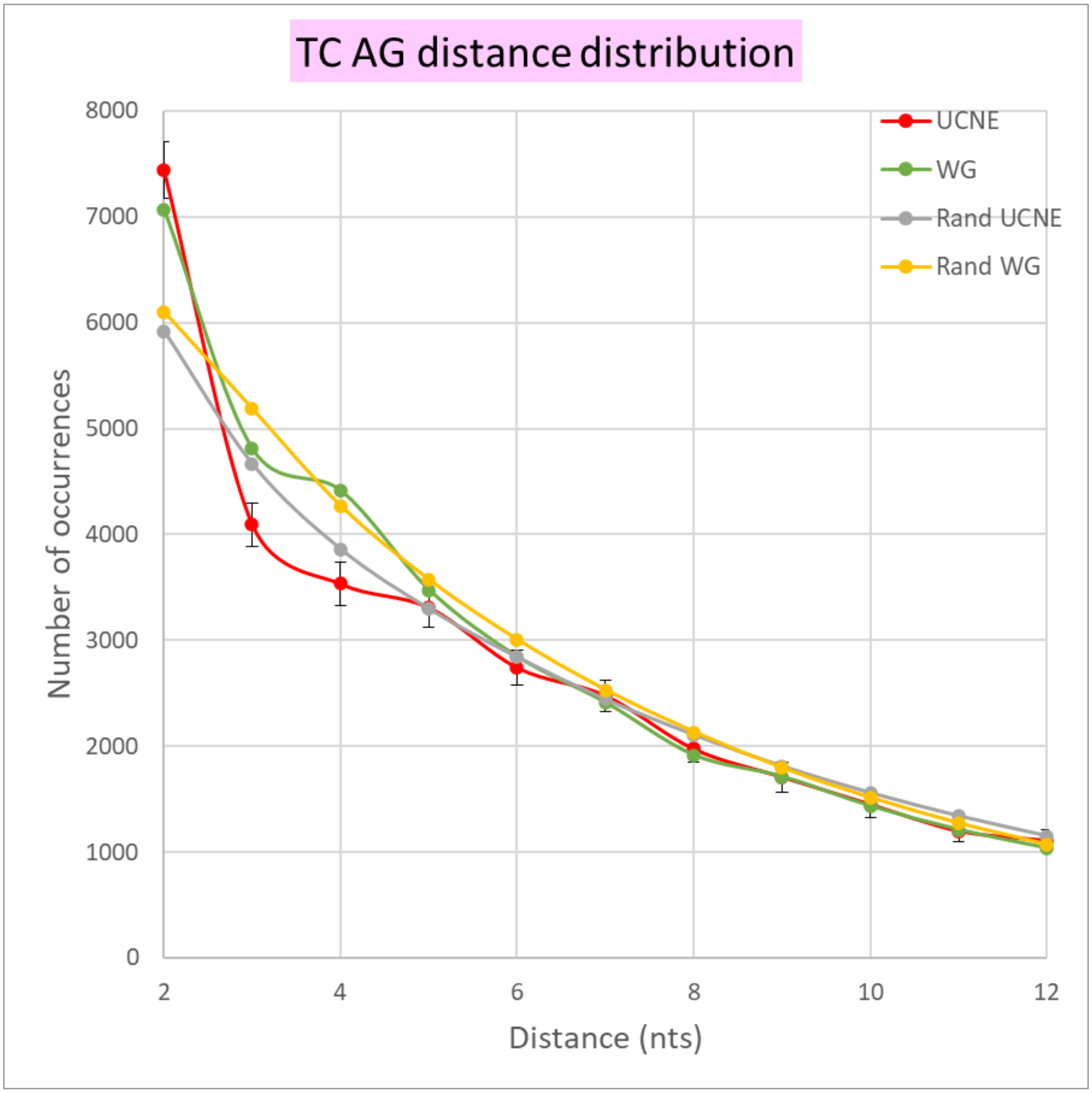
TC AG distance distribution
8000
7000
6000
5000
4000
3000
2000
1000
0
2
4
6
8
10
12
Number of occurrences
Distance (nts)
UCNE
WG
Rand UCNE
Rand WG

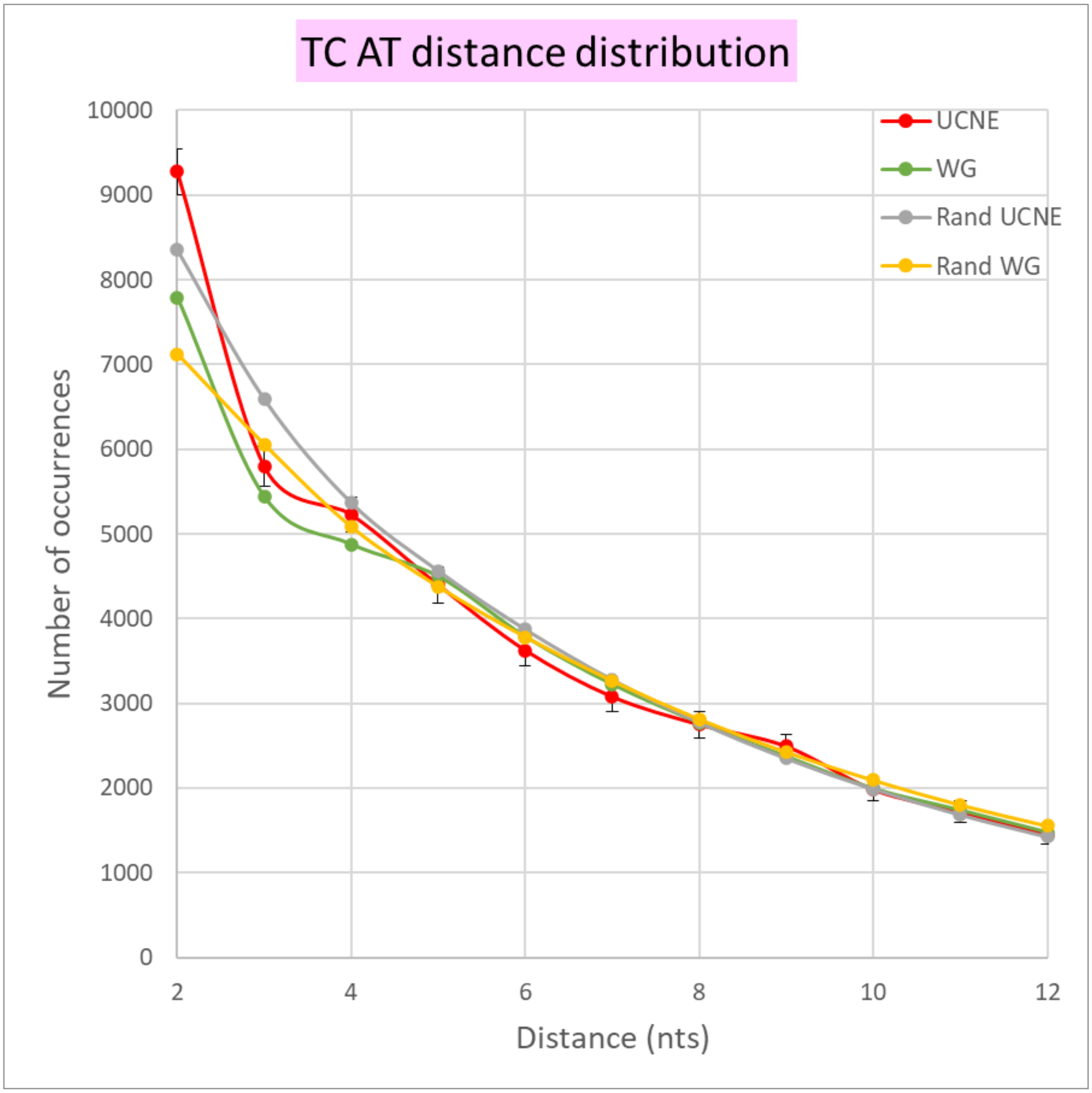

TC AT distance distribution
Number of occurrences
Distance (nts)
UCNE
WG
Rand UCNE
Rand WG
0
1000
2000
3000
4000
5000
6000
7000
8000
9000
10000
2
4
6
8
10
12

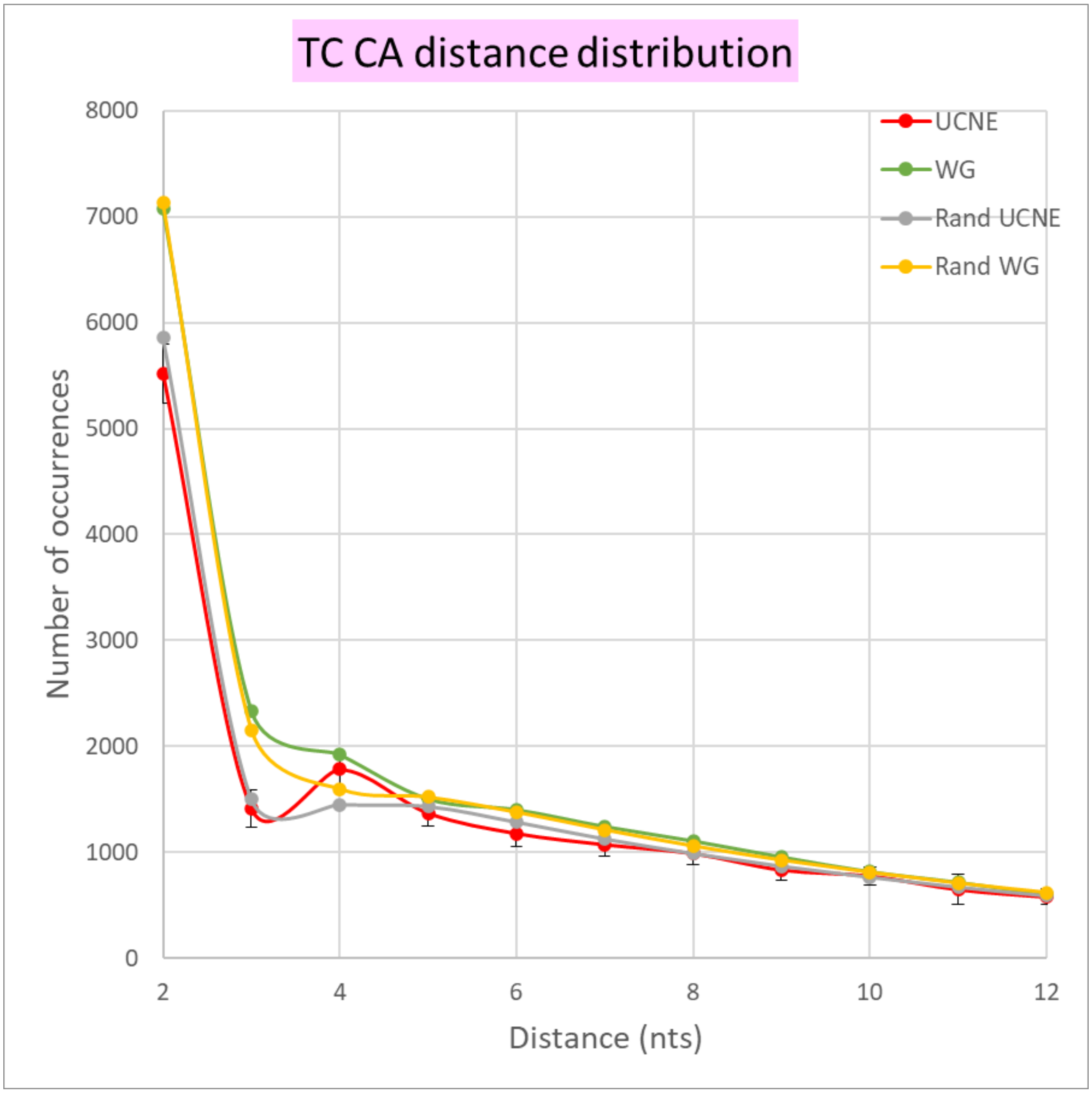
TC CA distance distribution
8000
7000
6000
5000
4000
3000
2000
1000
0
Number of occurrences
2
4
6
8
10
12
Distance (nts)
UCNE
WG
Rand UCNE
Rand WG

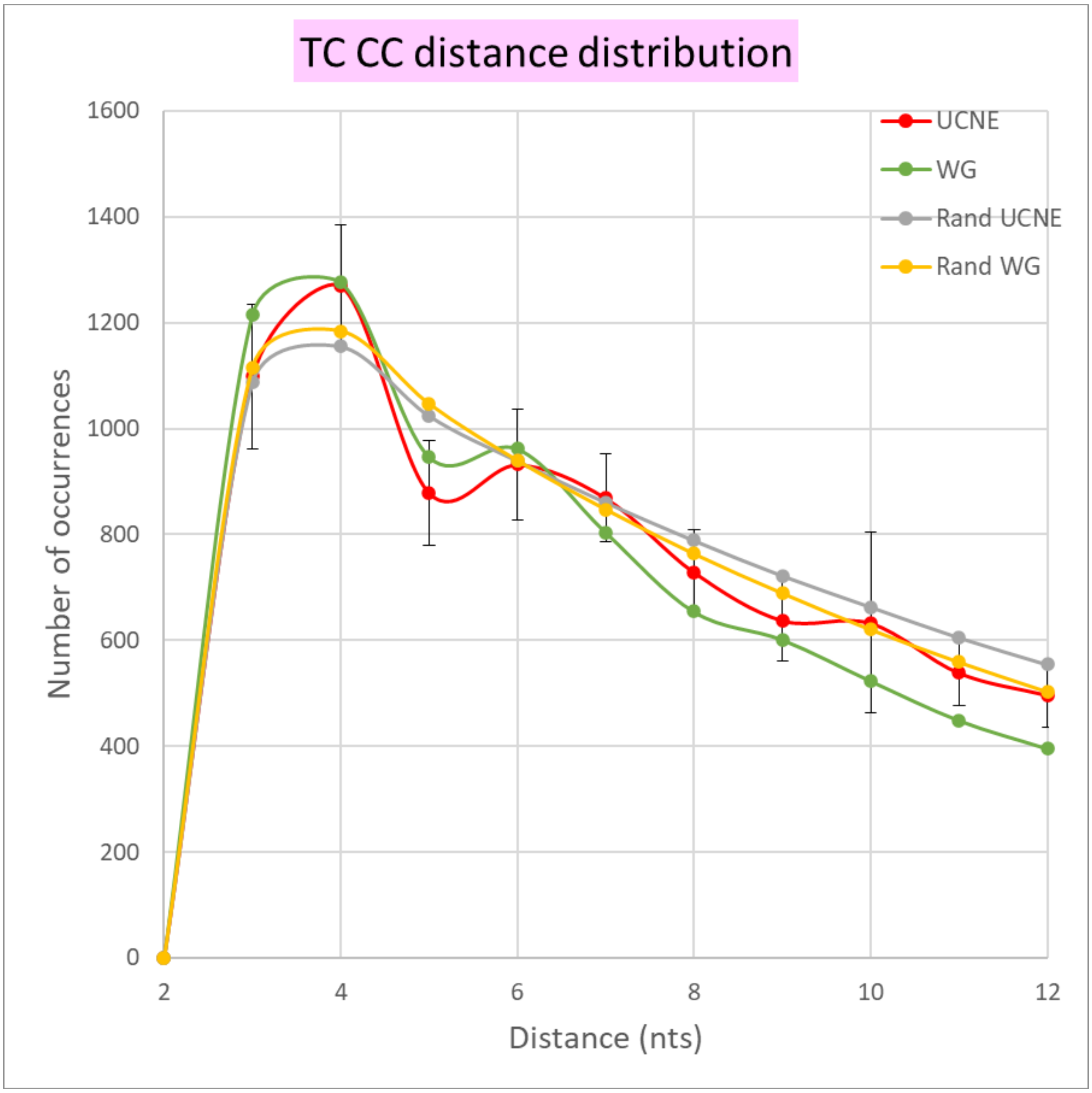
TC CC distance distribution
UCNE
WG
Rand UCNE
Rand WG
Number of occurrences
1600
1400
1200
1000
800
600
400
200
0
2
4
6
8
10
12
Distance (nts)

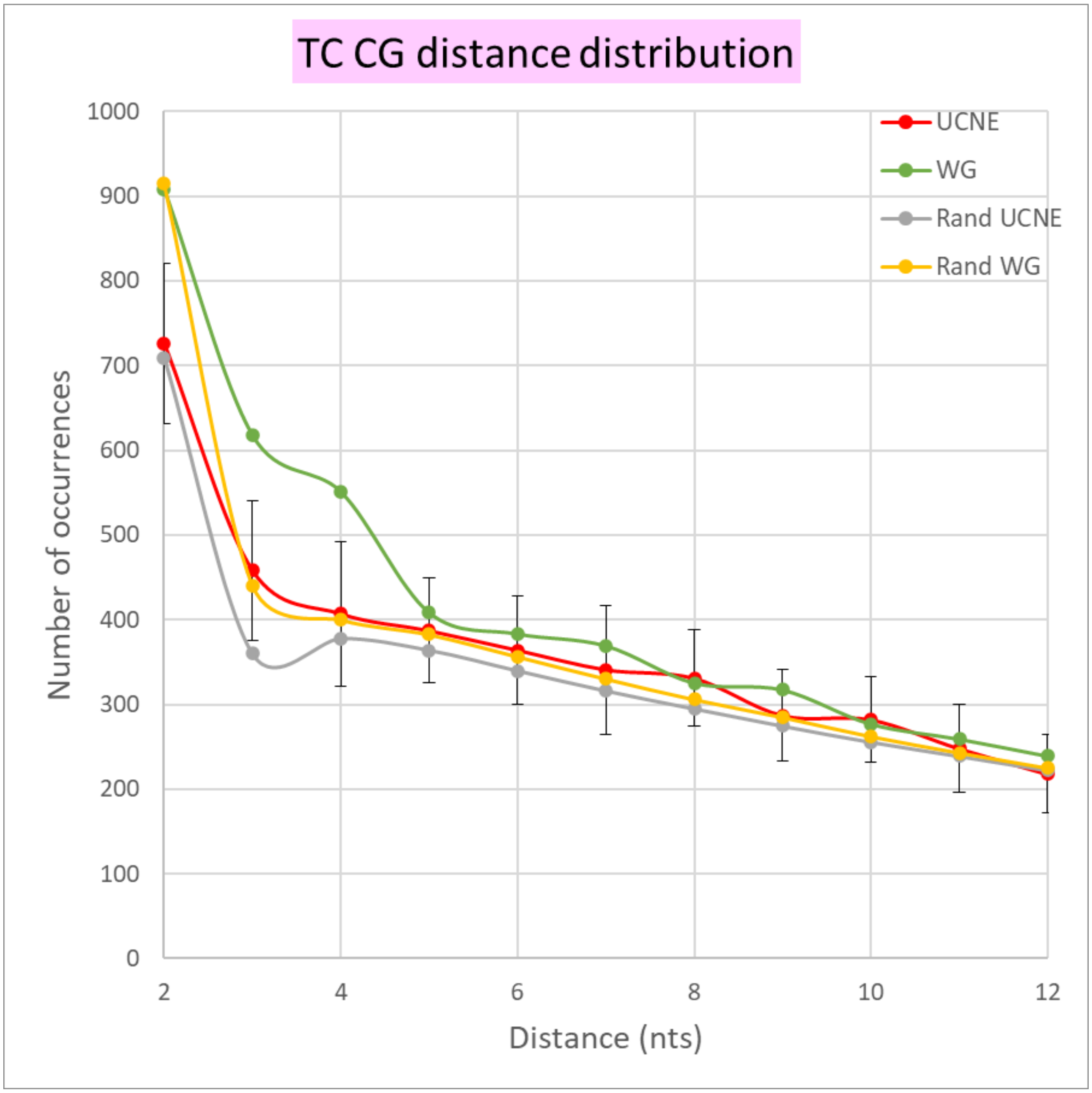

TC CG distance distribution
1000
900
800
700
600
500
400
300
200
100
0
Number of occurrences
2
4
6
8
10
12
Distance (nts)
UCNE
WG
Rand UCNE
Rand WG

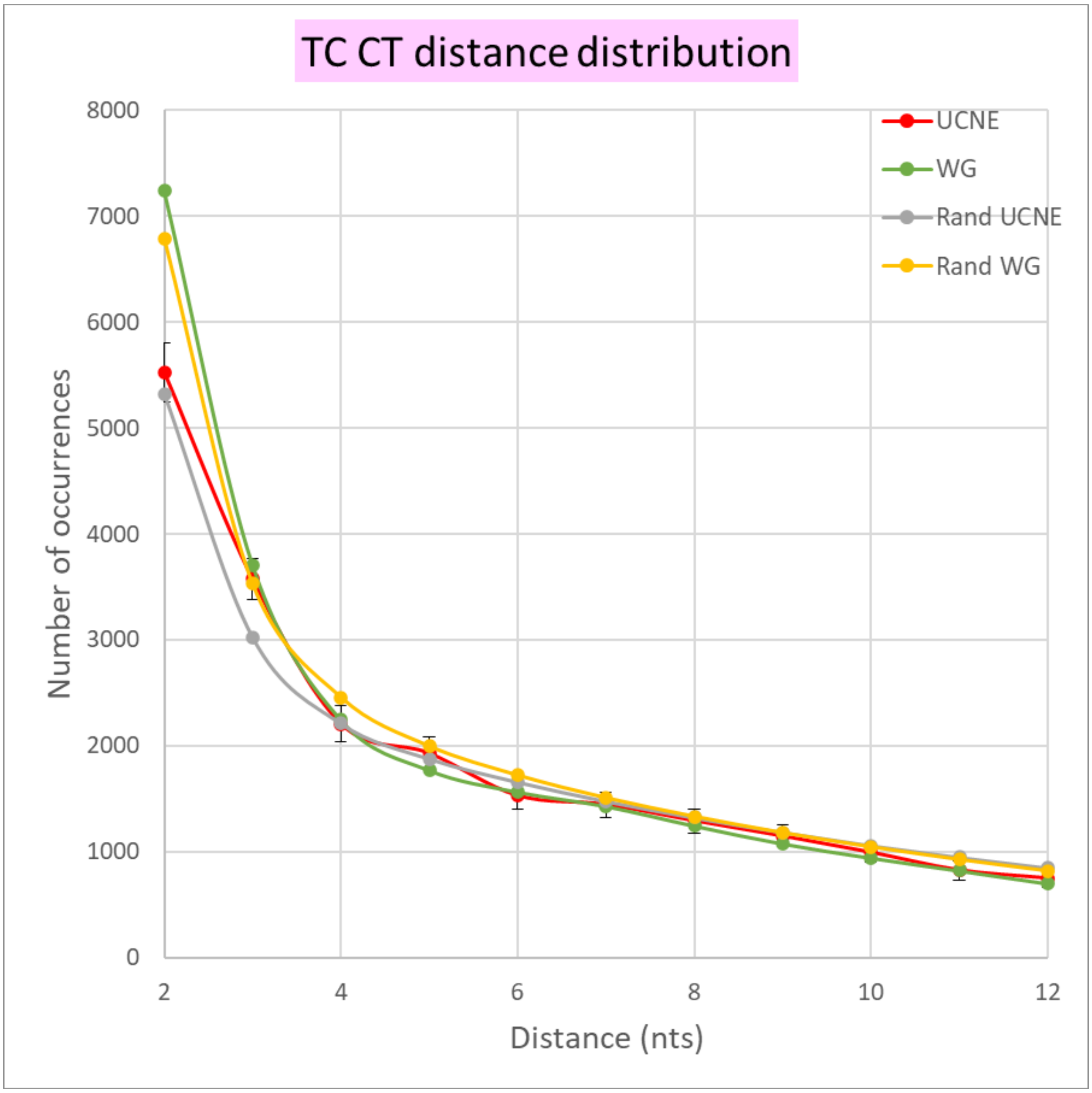

TC CT distance distribution
UCNE
WG
Rand UCNE
Rand WG
Number of occurrences
0
1000
2000
3000
4000
5000
6000
7000
8000
2
4
6
8
10
12
Distance (nts)

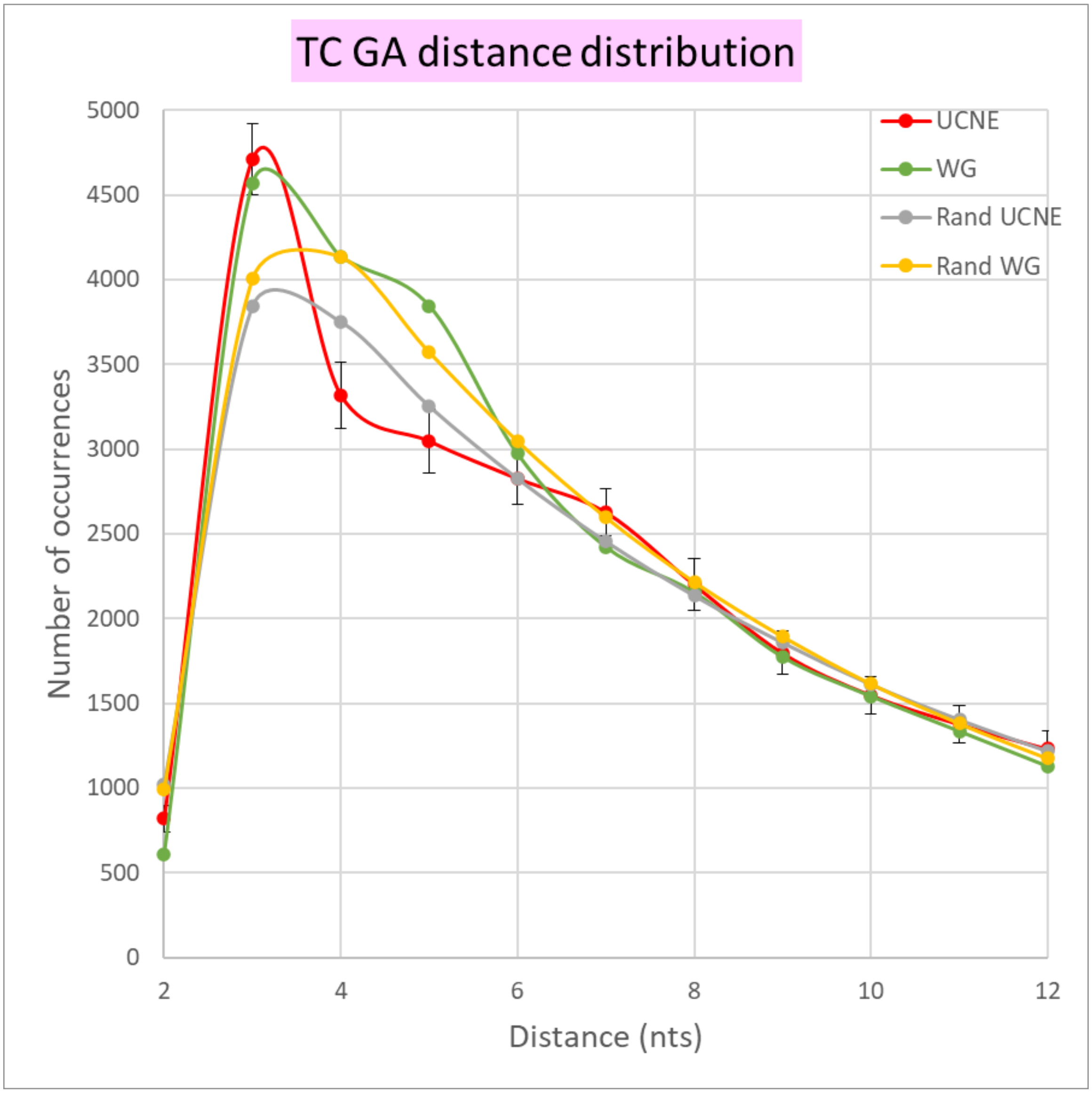
TC GA distance distribution
UCNE
WG
Rand UCNE
Rand WG
Number of occurrences
Distance (nts)
0
500
1000
1500
2000
2500
3000
3500
4000
4500
5000
2
4
6
8
10
12

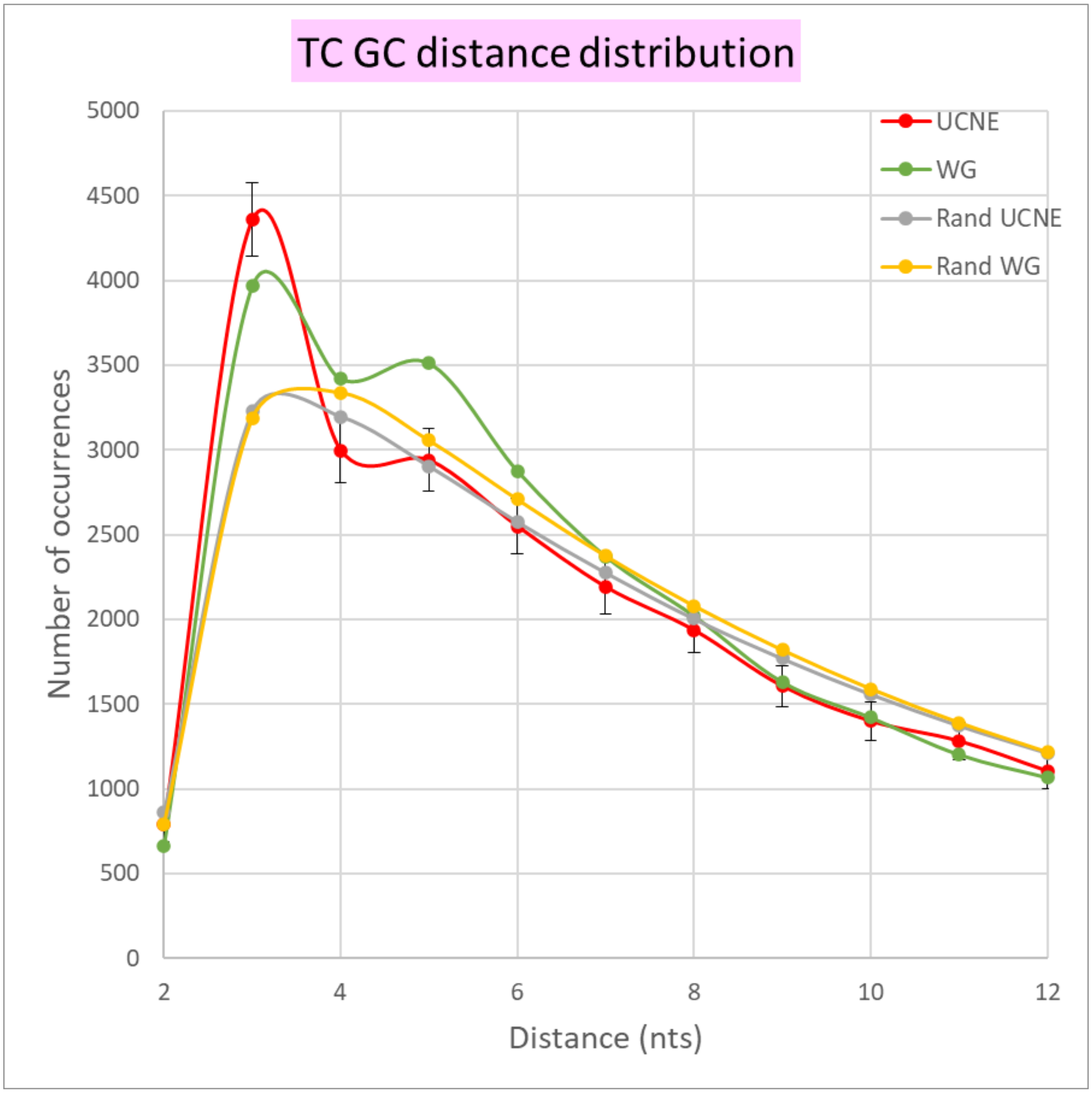

TC GC distance distribution
5000
4500
4000
3500
3000
2500
2000
1500
1000
500
0
Number of occurrences
2
4
6
8
10
12
Distance (nts)
UCNE
WG
Rand UCNE
Rand WG

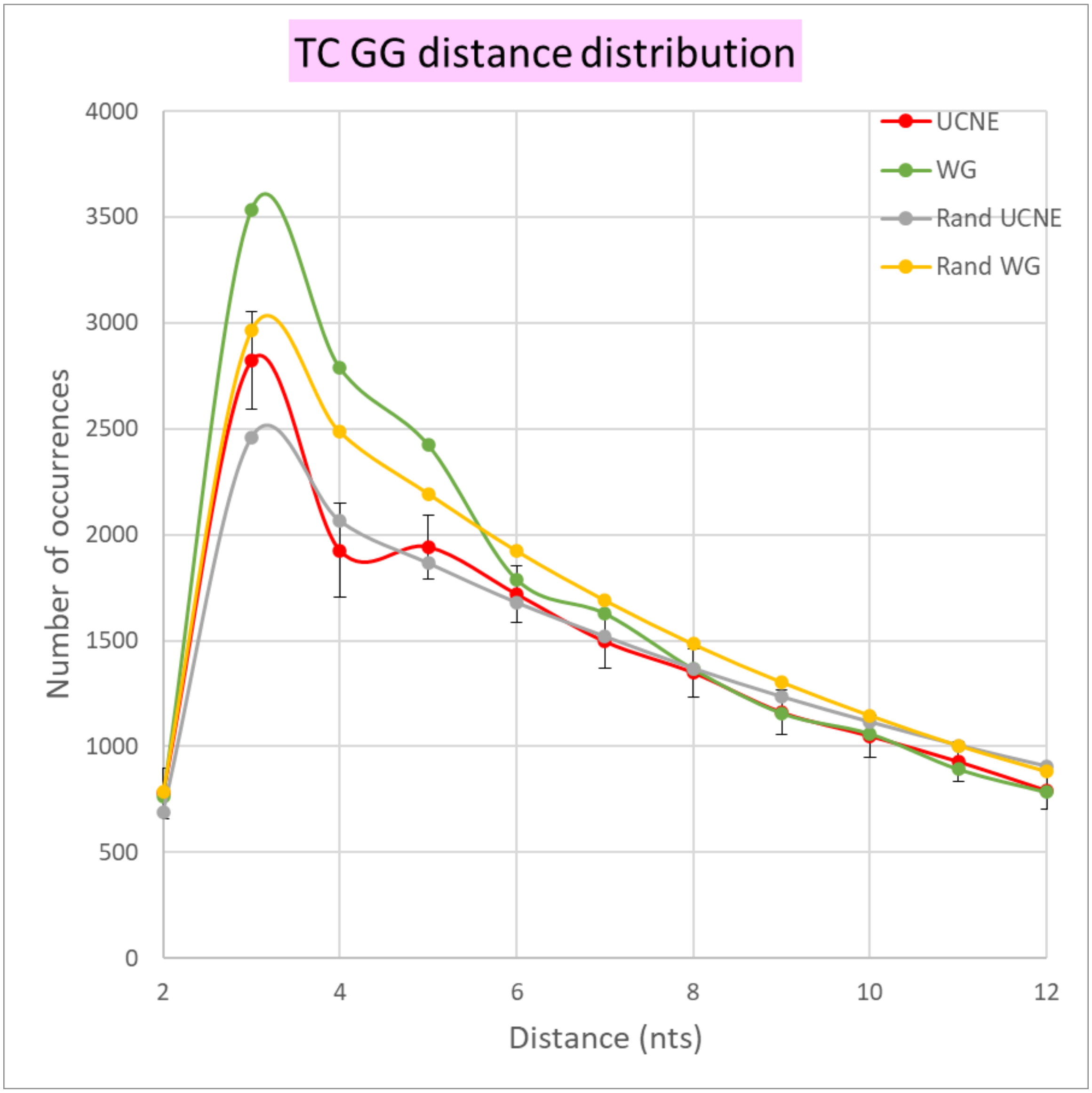

TC GG distance distribution
UCNE
WG
Rand UCNE
Rand WG
Number of occurrences
Distance (nts)
0
500
1000
1500
2000
2500
3000
3500
4000
2
4
6
8
10
12

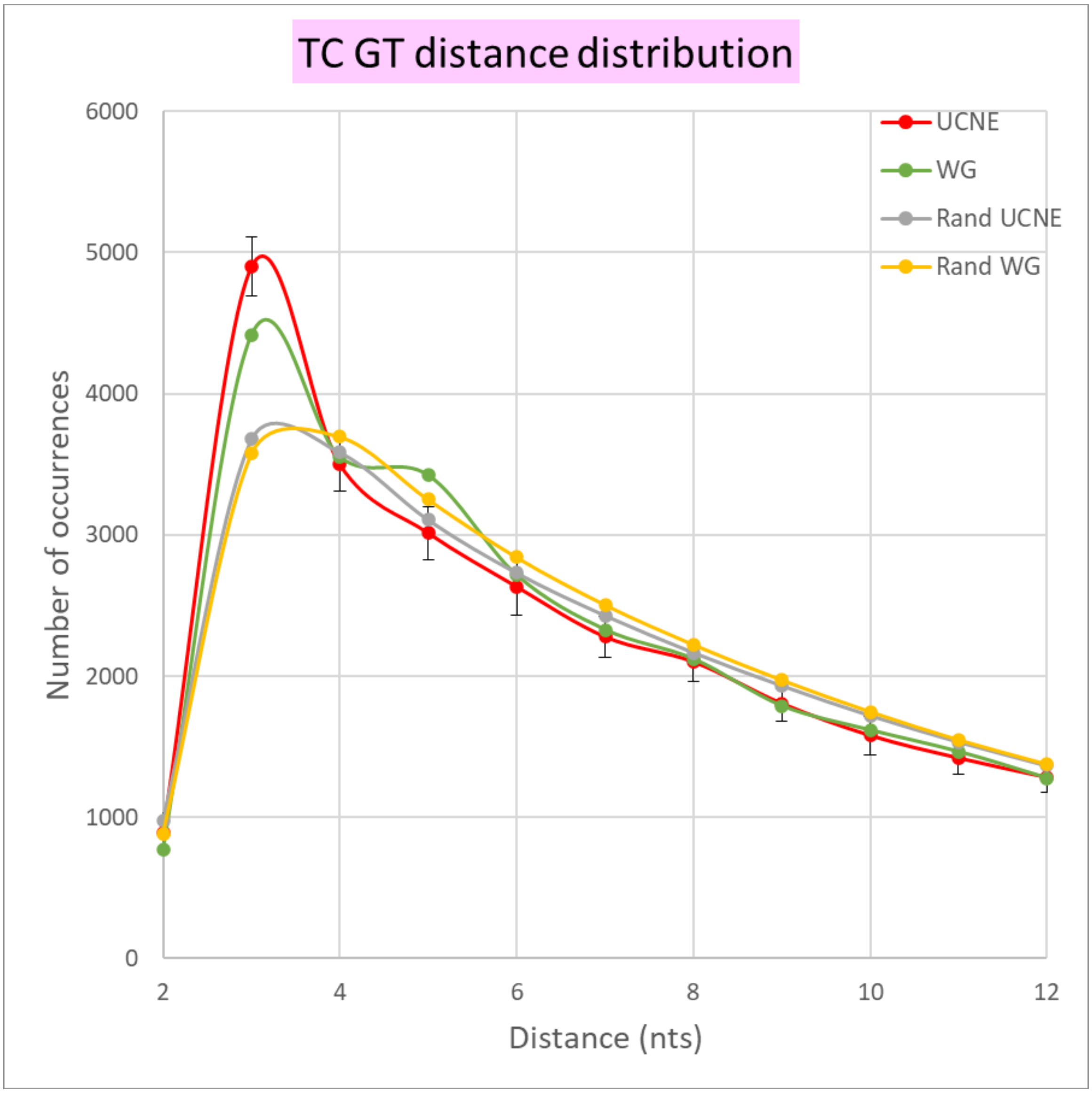

TC GT distance distribution
6000
5000
4000
3000
2000
1000
0
Number of occurrences
2
4
6
8
10
12
Distance (nts)
UCNE
WG
Rand UCNE
Rand WG

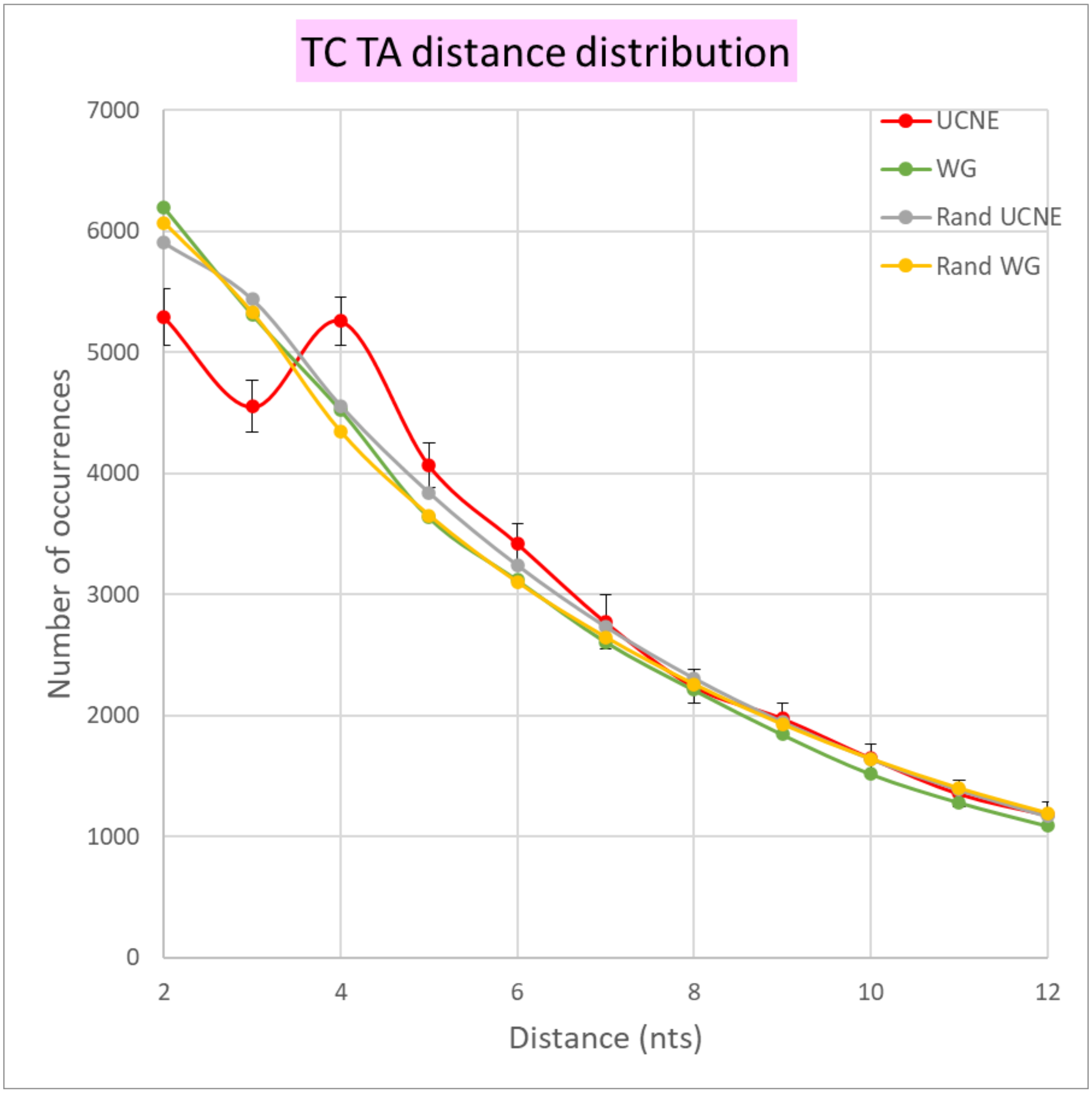

TC TA distance distribution
7000
6000
5000
4000
3000
2000
1000
0
Number of occurrences
2
4
6
8
10
12
Distance (nts)
UCNE
WG
Rand UCNE
Rand WG

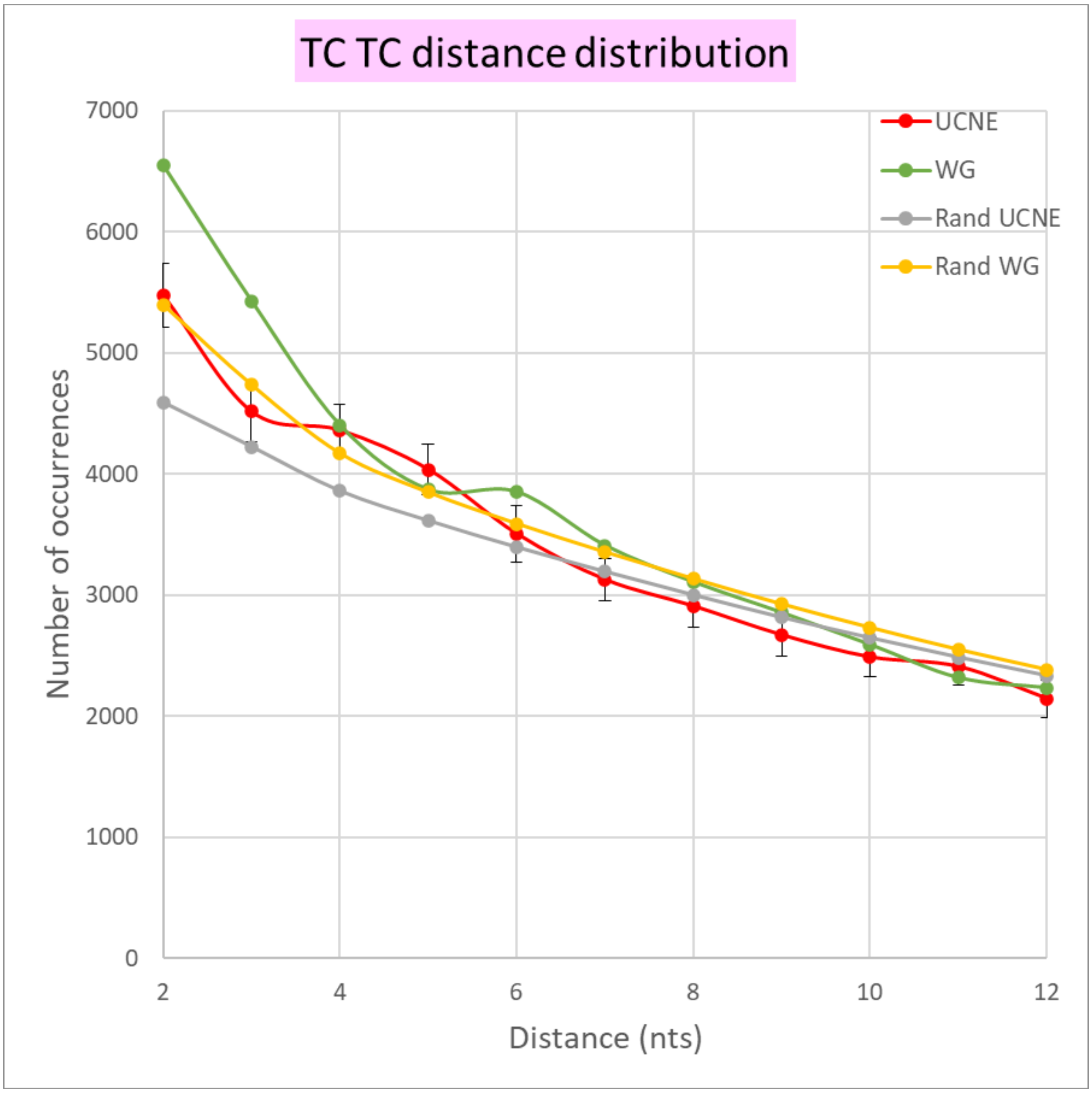
TC TC distance distribution
UCNE
WG
Rand UCNE
Rand WG
Number of occurrences
Distance (nts)
0
1000
2000
3000
4000
5000
6000
7000
2
4
6
8
10
12

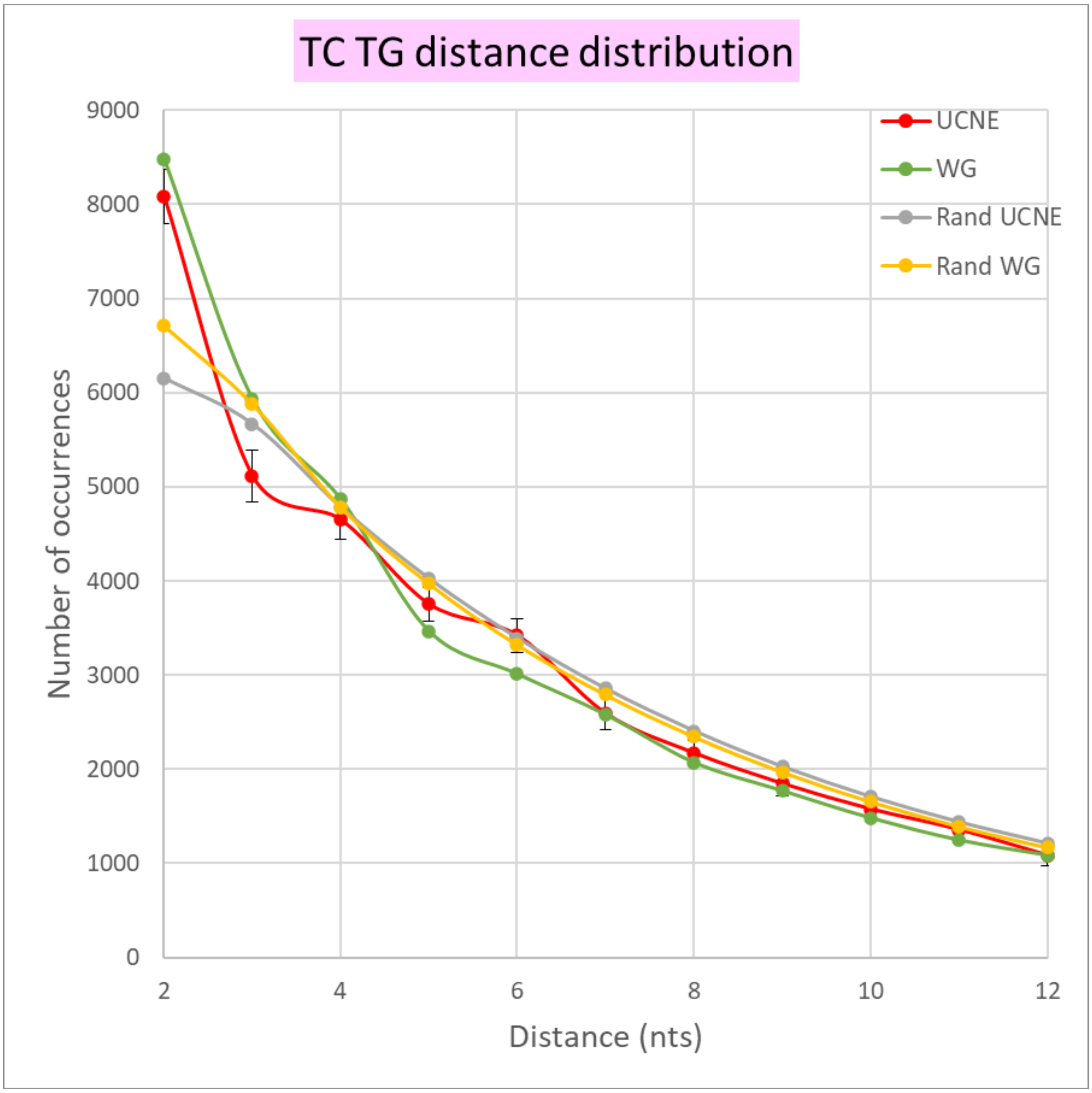
TC TG distance distribution
UCNE
WG
Rand UCNE
Rand WG
Number of occurrences
Distance (nts)
0
1000
2000
3000
4000
5000
6000
7000
8000
9000
2
4
6
8
10
12

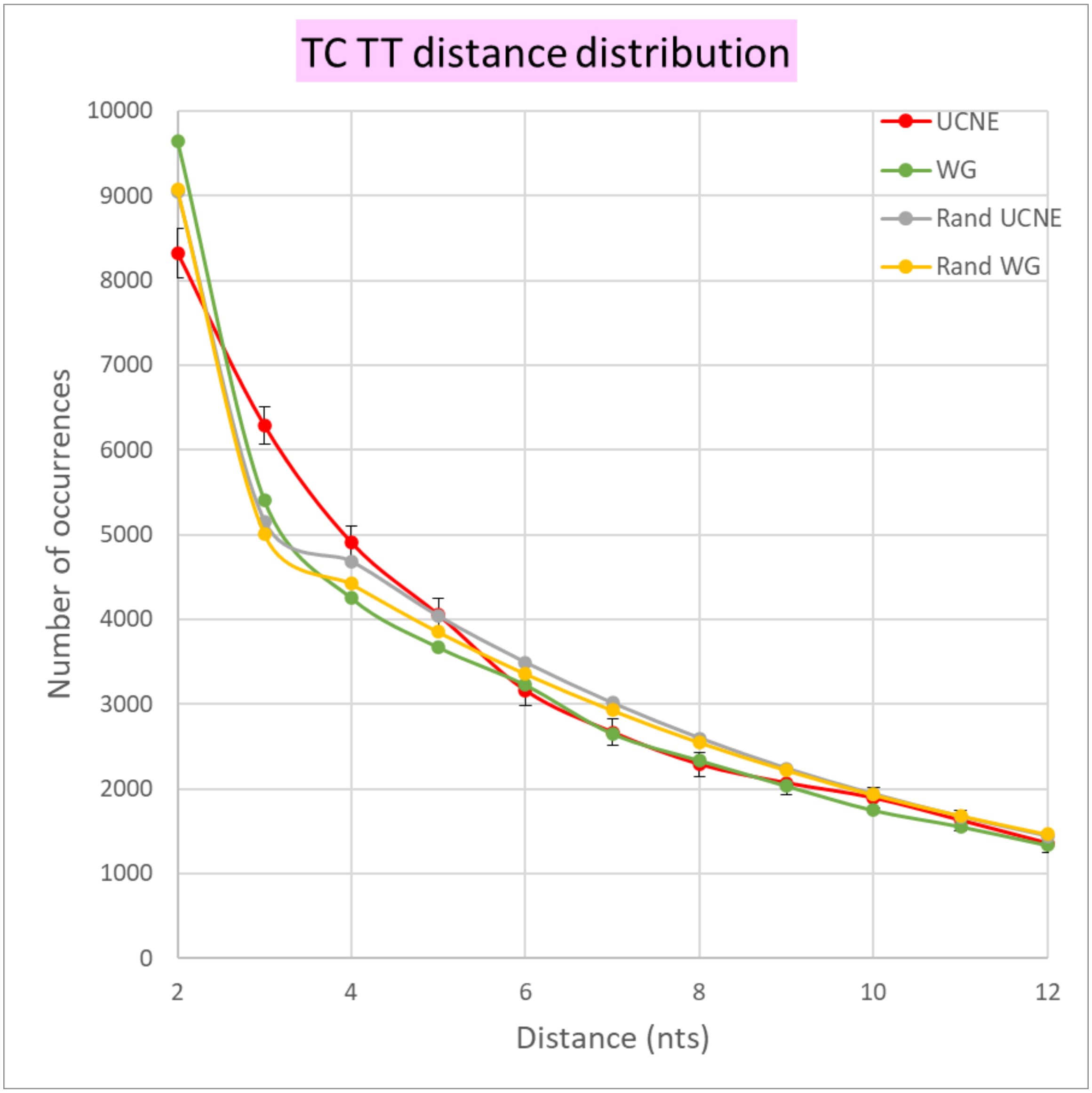
TC TT distance distribution
UCNE
WG
Rand UCNE
Rand WG
Number of occurrences
10000
9000
8000
7000
6000
5000
4000
3000
2000
1000
0
2
4
6
8
10
12
Distance (nts)

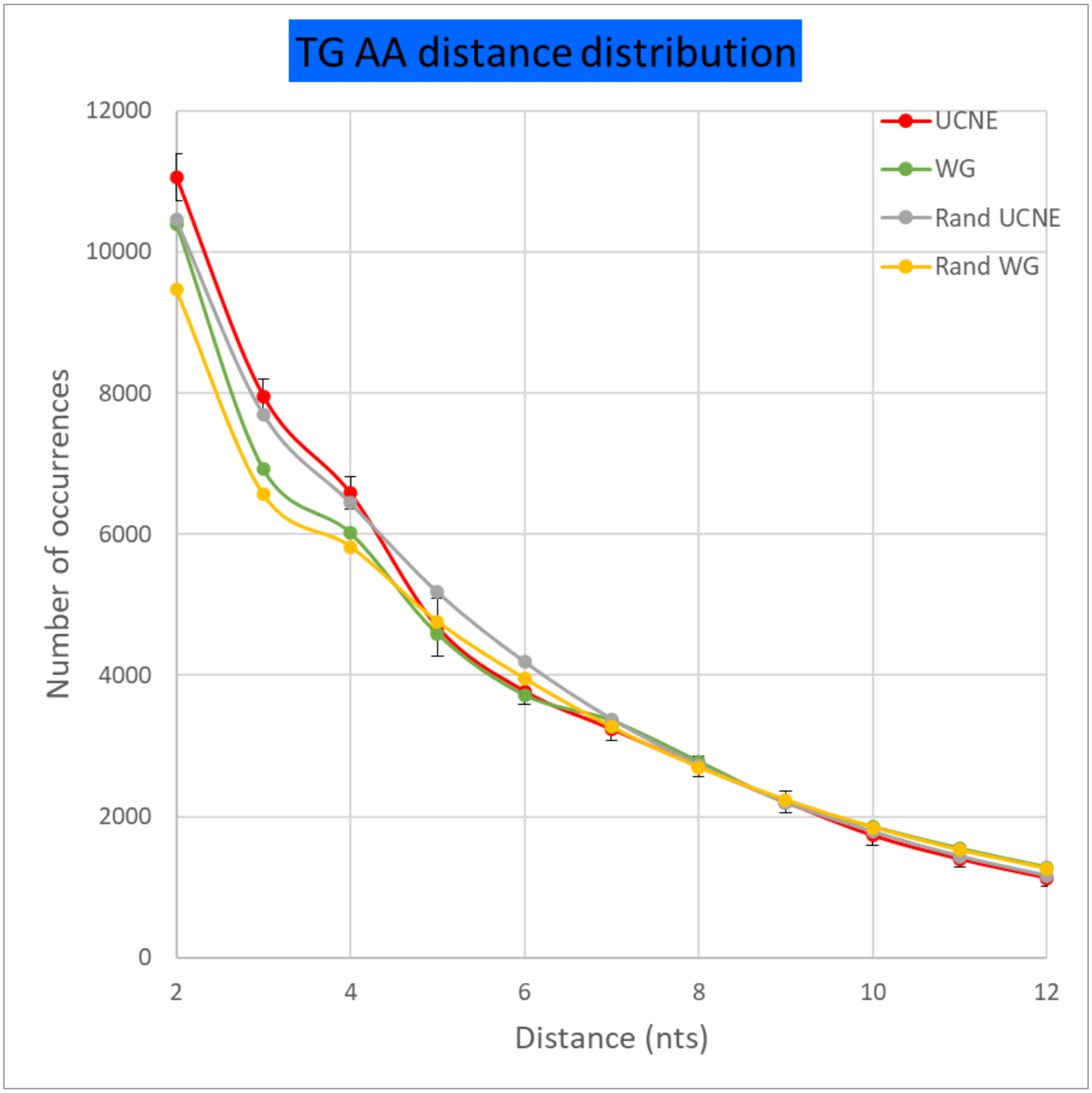
TG AA distance distribution
Number of occurrences
Distance (nts)
UCNE
WG
Rand UCNE
Rand WG
0
2000
4000
6000
8000
10000
12000
2
4
6
8
10
12

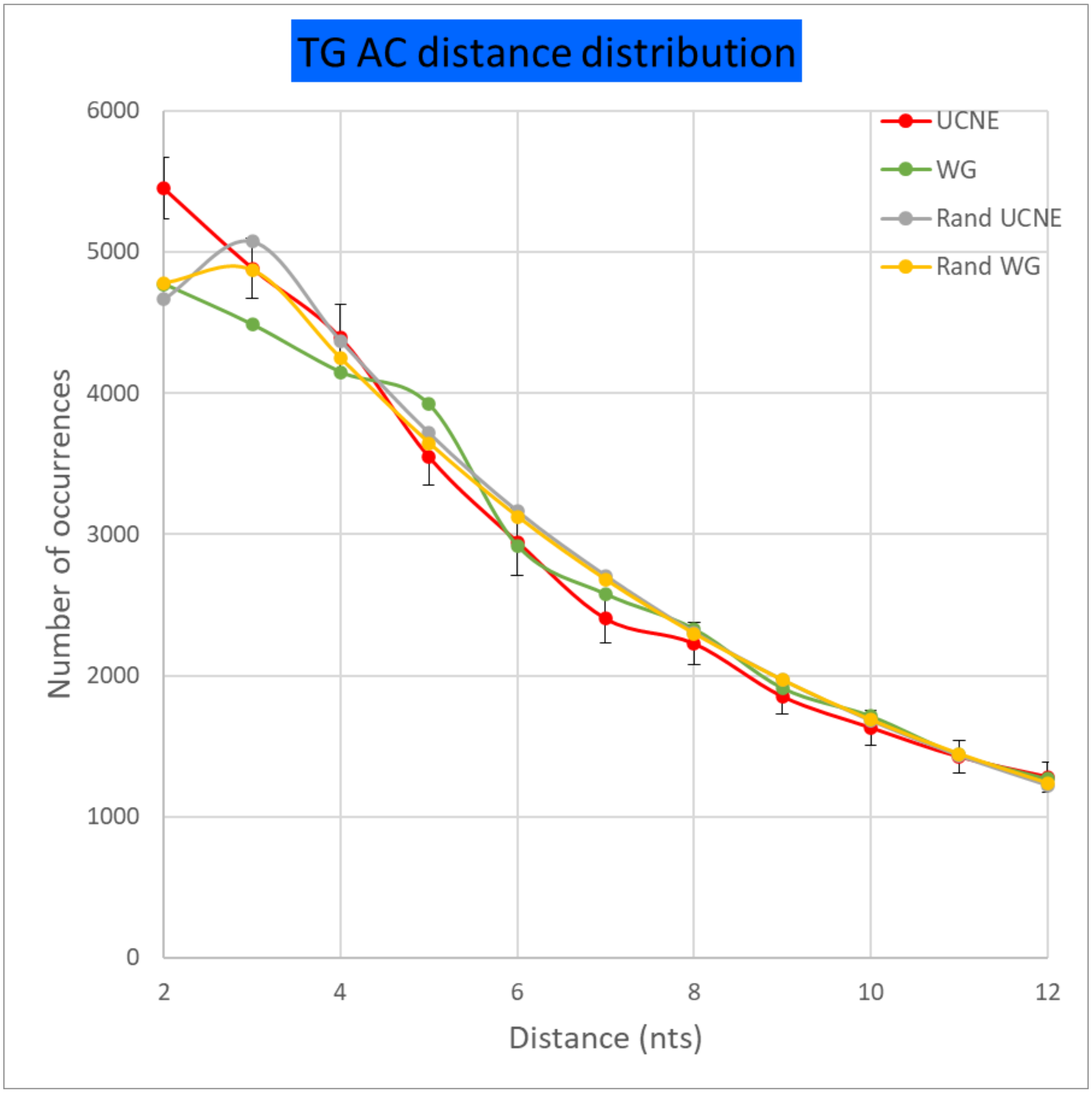

TG AC distance distribution
Number of occurrences
Distance (nts)
UCNE
WG
Rand UCNE
Rand WG
6000
5000
4000
3000
2000
1000
0
2
4
6
8
10
12

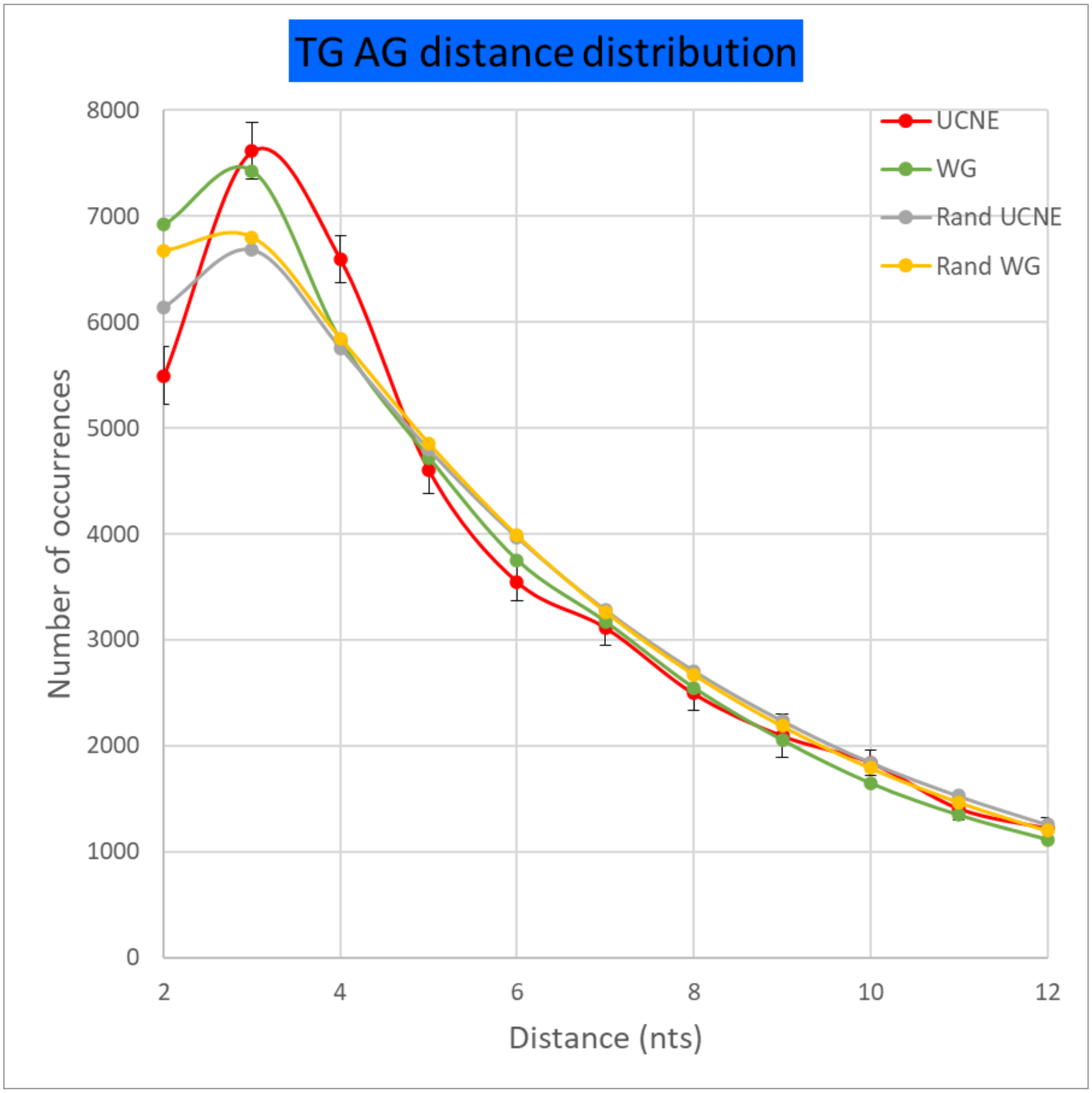
TG AG distance distribution
Number of occurrences
Distance (nts)
UCNE
WG
Rand UCNE
Rand WG
0
1000
2000
3000
4000
5000
6000
7000
8000
2
4
6
8
10
12

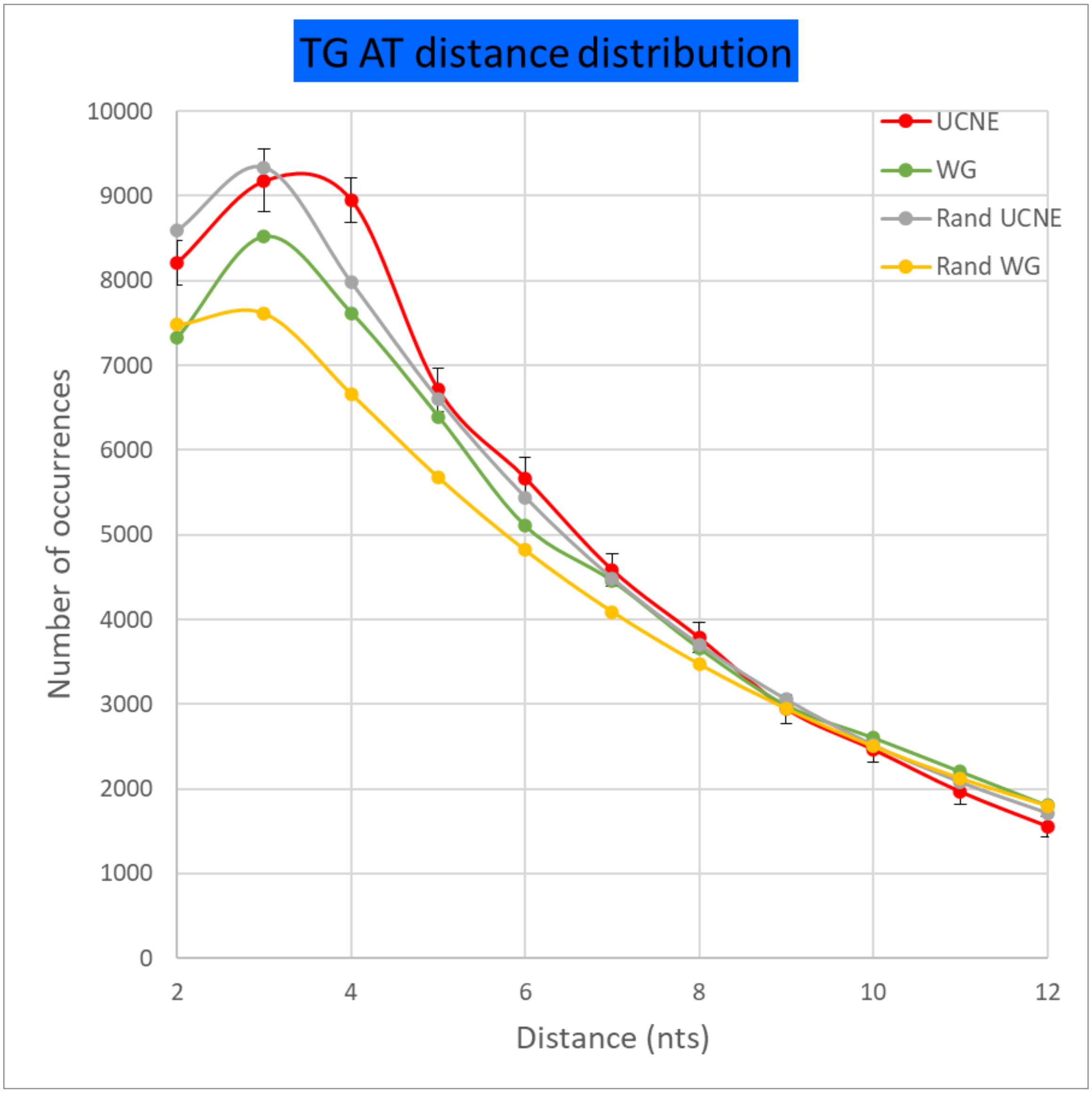
TG AT distance distribution
UCNE
WG
Rand UCNE
Rand WG
Number of occurrences
10000
9000
8000
7000
6000
5000
4000
3000
2000
1000
0
2
4
6
8
10
12
Distance (nts)

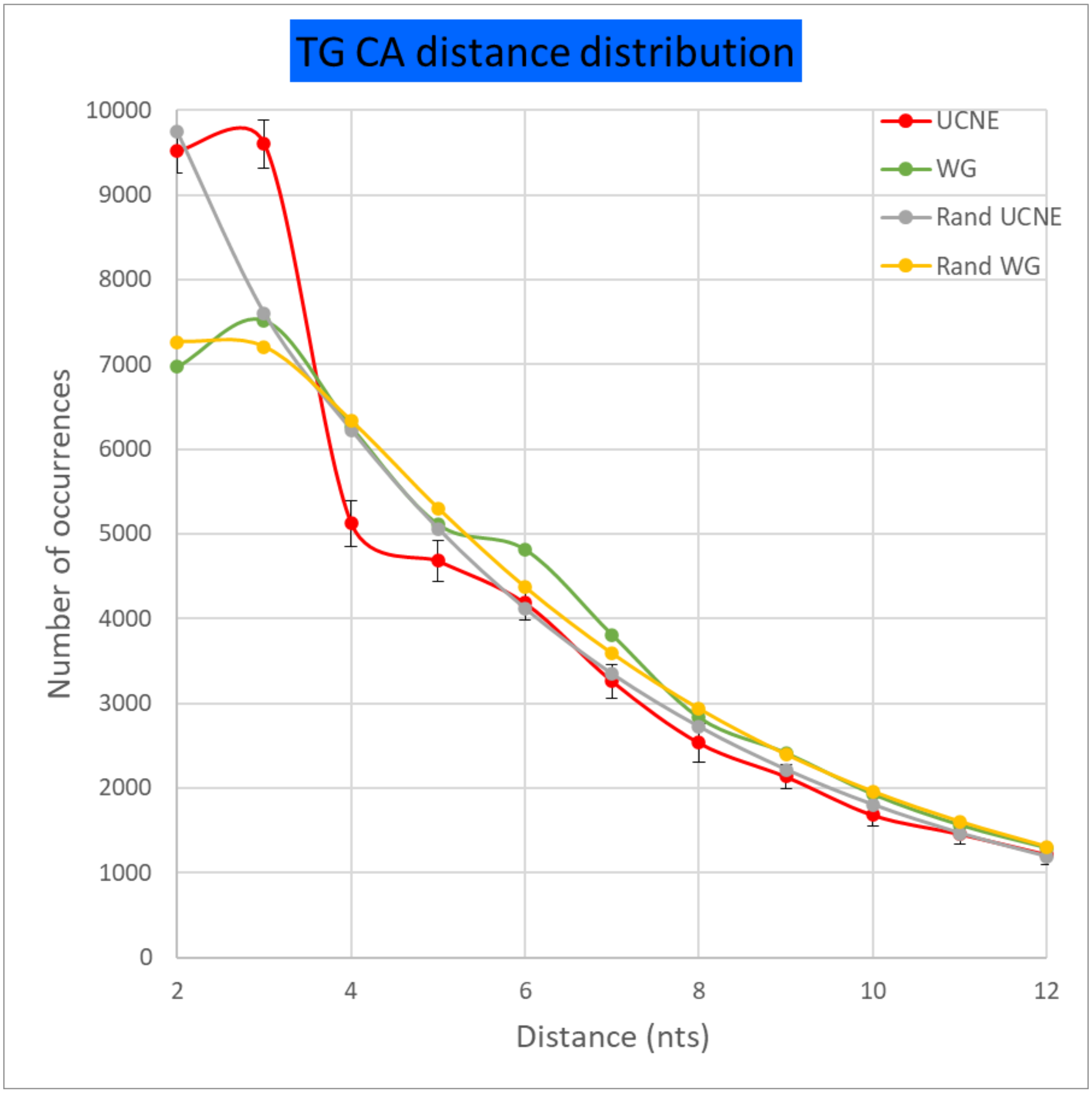
TG CA distance distribution
Number of occurrences
10000
9000
8000
7000
6000
5000
4000
3000
2000
1000
0
2
4
6
8
10
12
Distance (nts)
UCNE
WG
Rand UCNE
Rand WG

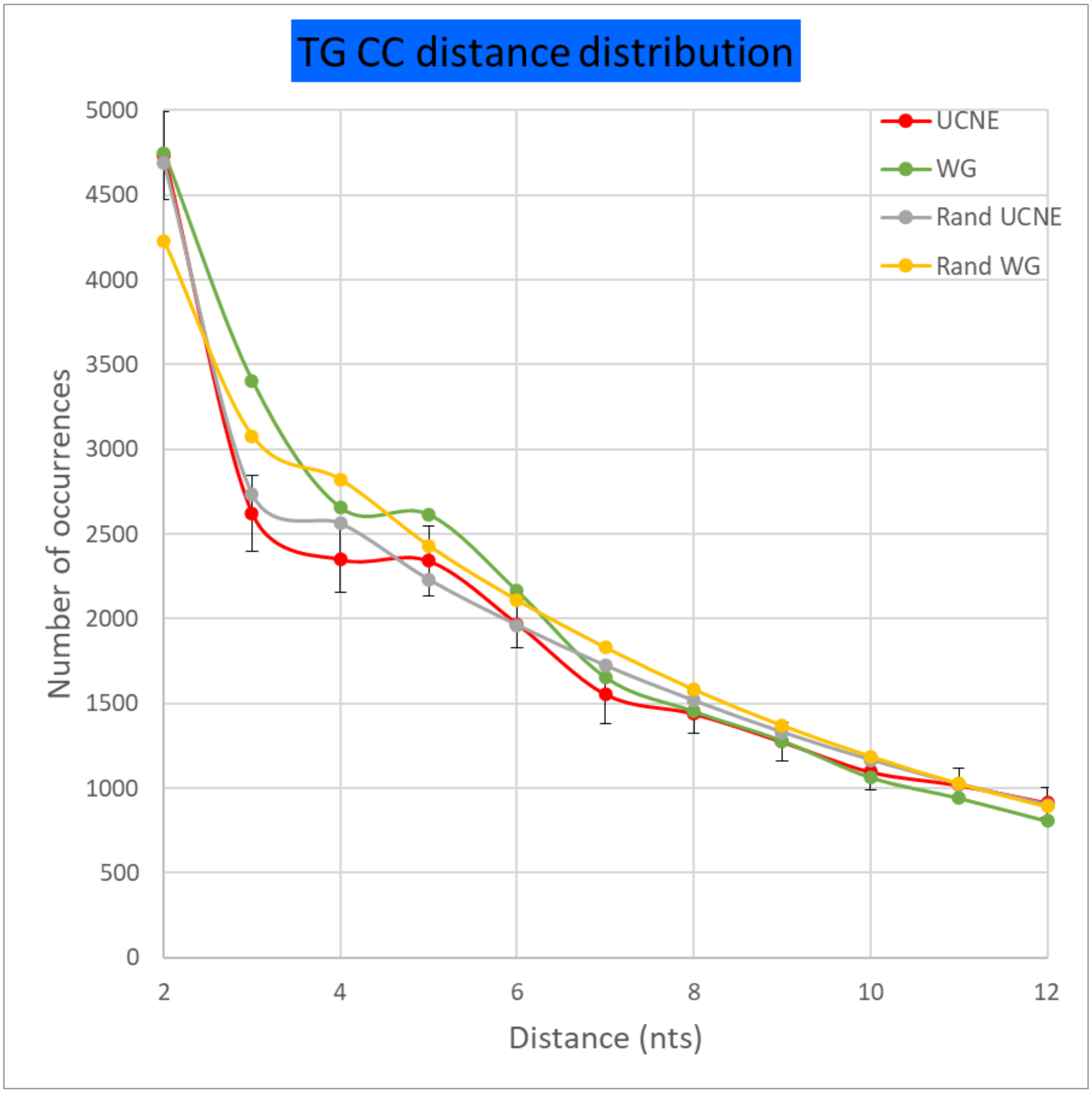

TG CC distance distribution
Number of occurrences
Distance (nts)
UCNE
WG
Rand UCNE
Rand WG
0
500
1000
1500
2000
2500
3000
3500
4000
4500
5000
2
4
6
8
10
12

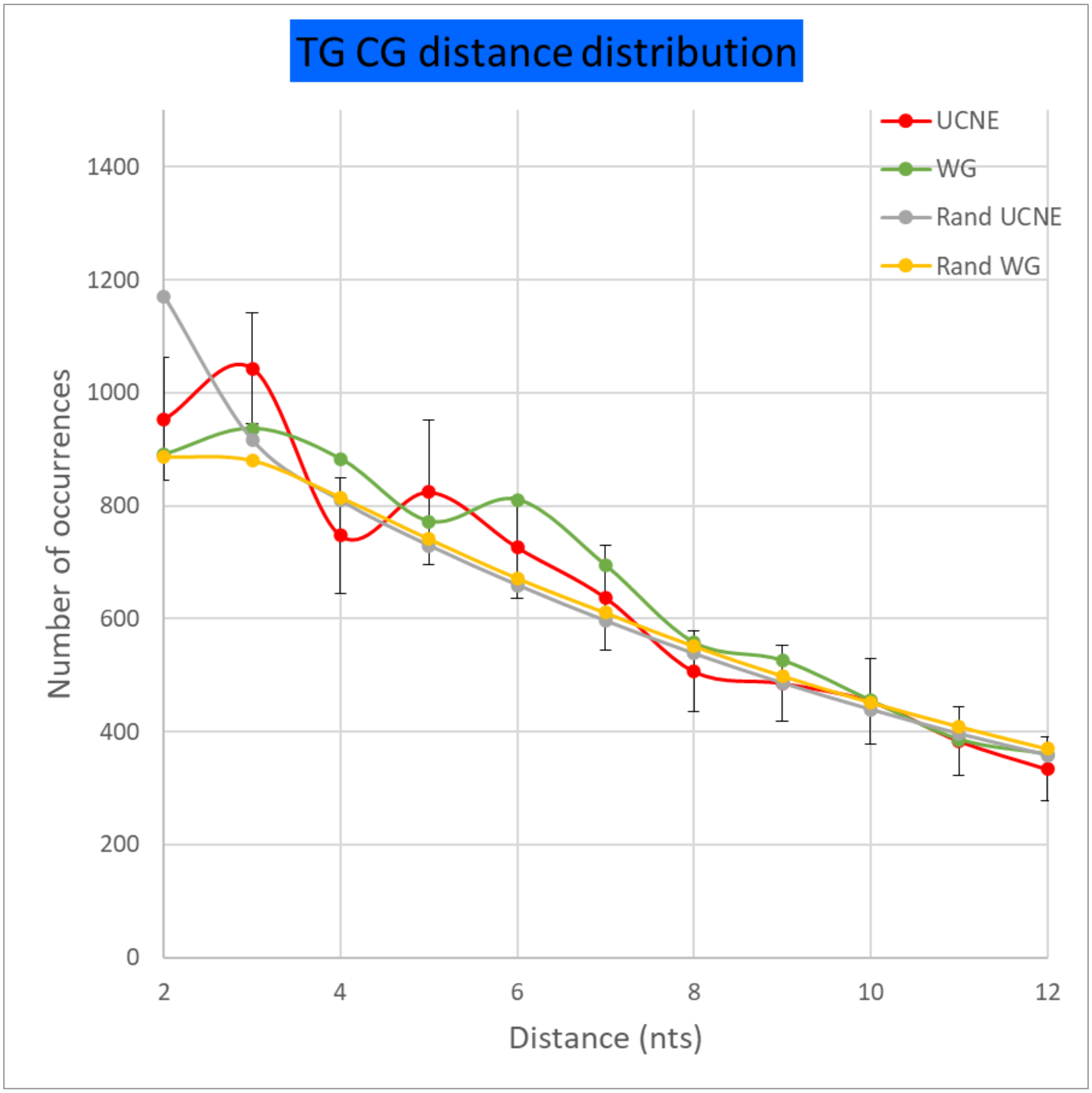
TG CG distance distribution
UCNE
WG
Rand UCNE
Rand WG
Number of occurrences
0
200
400
600
800
1000
1200
1400
2
4
6
8
10
12
Distance (nts)

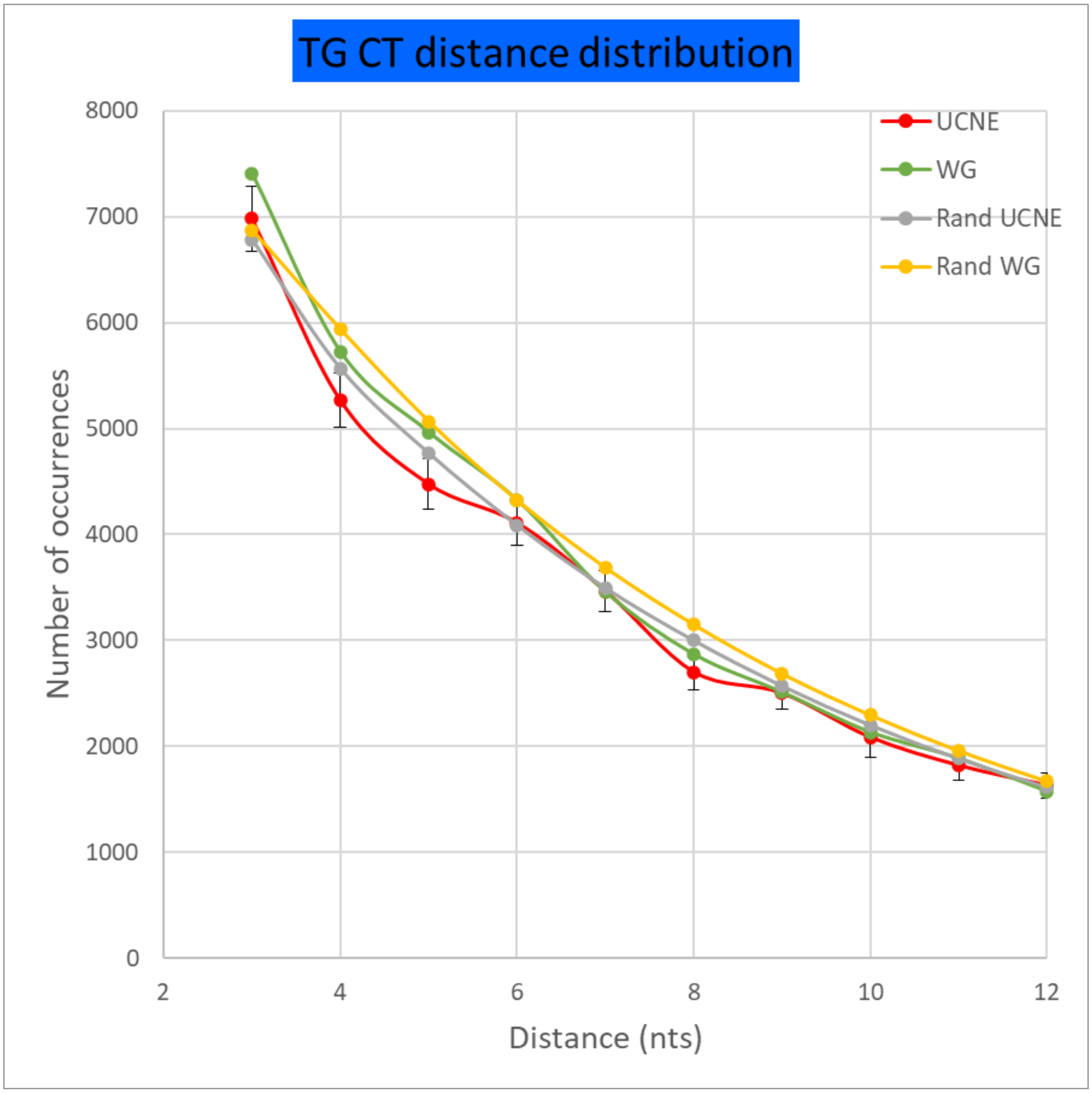

TG CT distance distribution
UCNE
WG
Rand UCNE
Rand WG
Number of occurrences
8000
7000
6000
5000
4000
3000
2000
1000
0
2
4
6
8
10
12
Distance (nts)

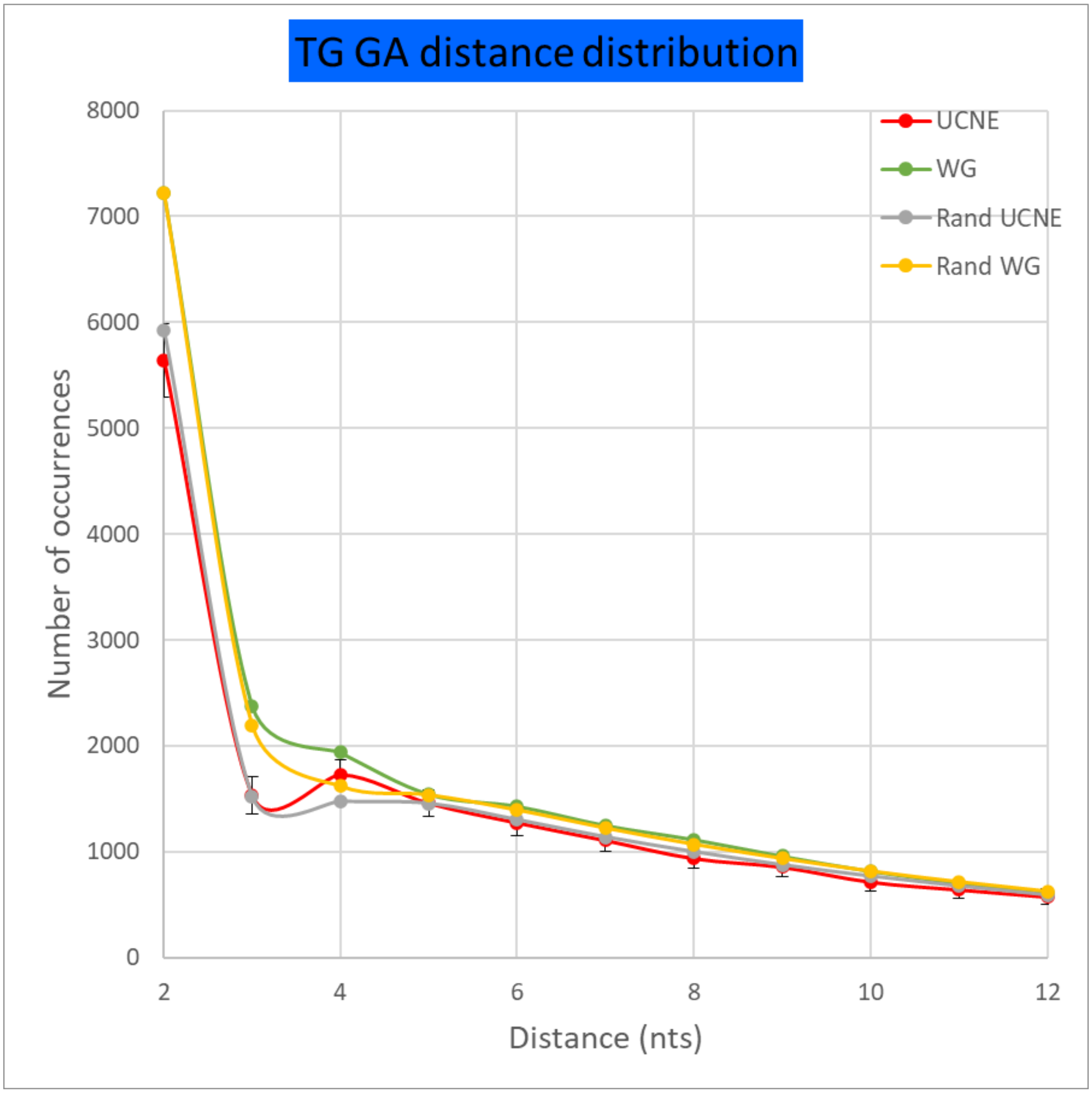

TG GA distance distribution
8000
7000
6000
5000
4000
3000
2000
1000
0
Number of occurrences
2
4
6
8
10
12
Distance (nts)
UCNE
WG
Rand UCNE
Rand WG

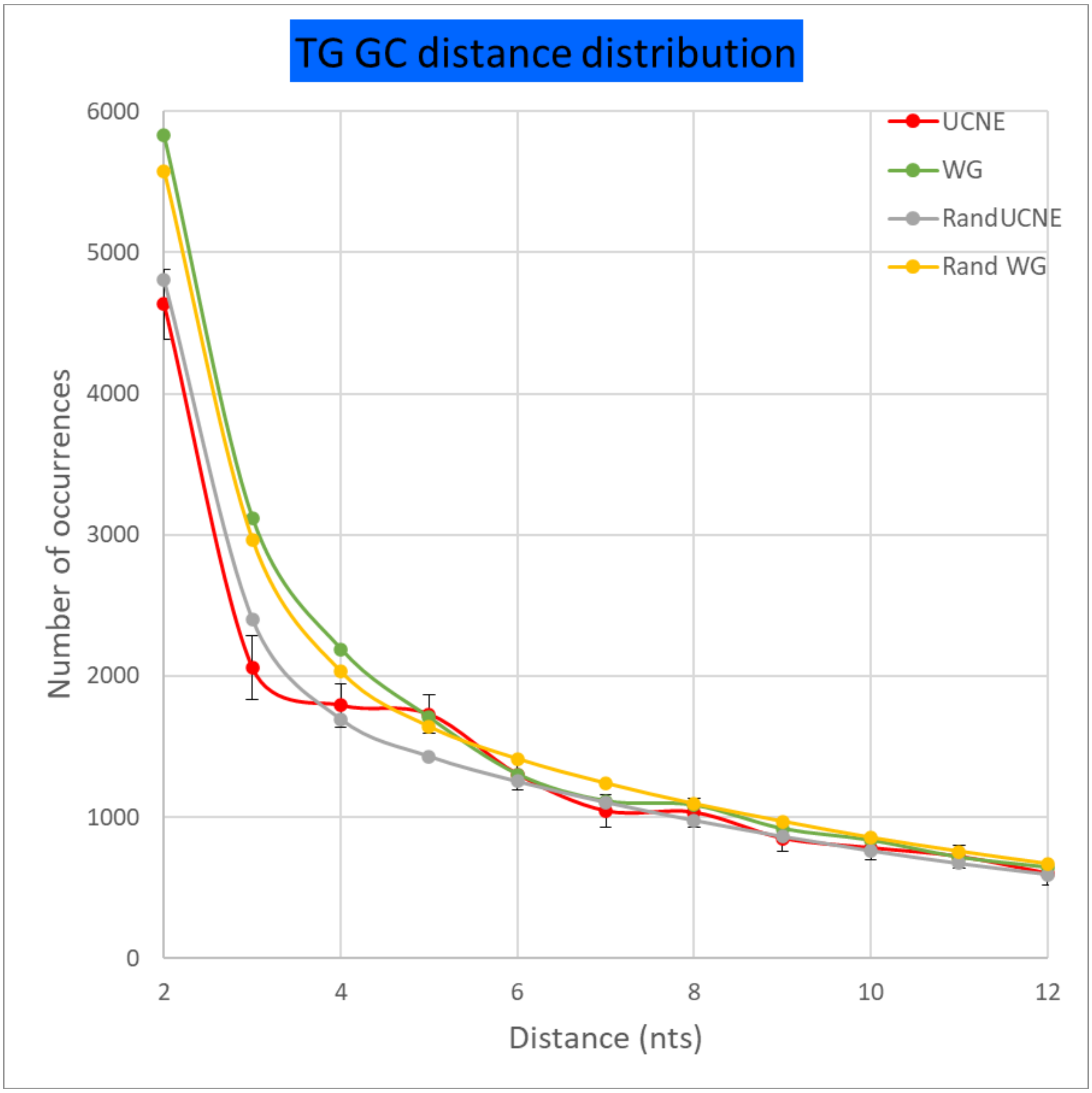
TG GC distance distribution
6000
5000
4000
3000
2000
1000
0
2
4
6
8
10
12
Number of occurrences
Distance (nts)
UCNE
WG
RandUCNE
Rand WG

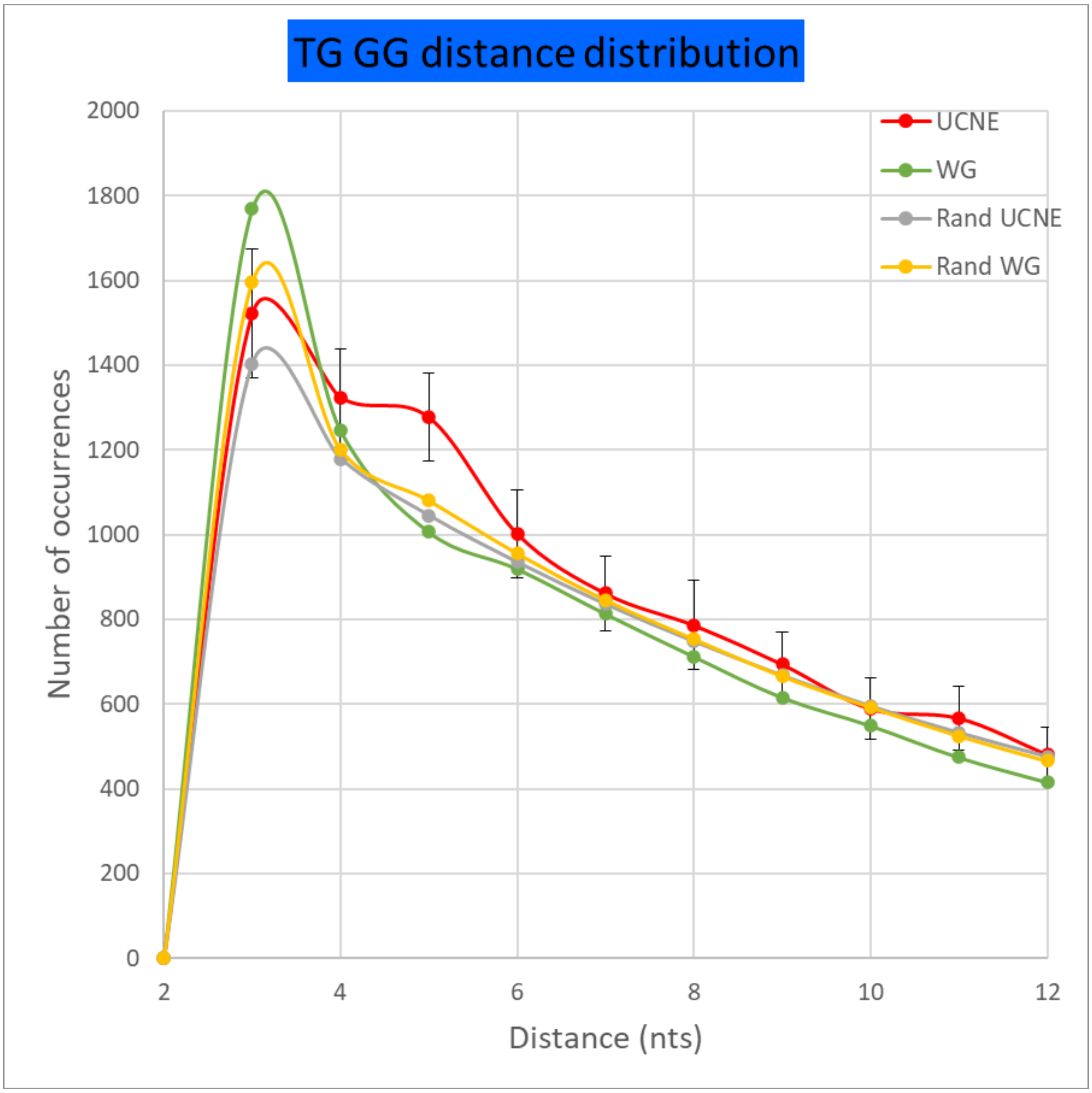
TG GG distance distribution
UCNE
WG
Rand UCNE
Rand WG
Number of occurrences
Distance (nts)
2000
1800
1600
1400
1200
1000
800
600
400
200
0
2
4
6
8
10
12

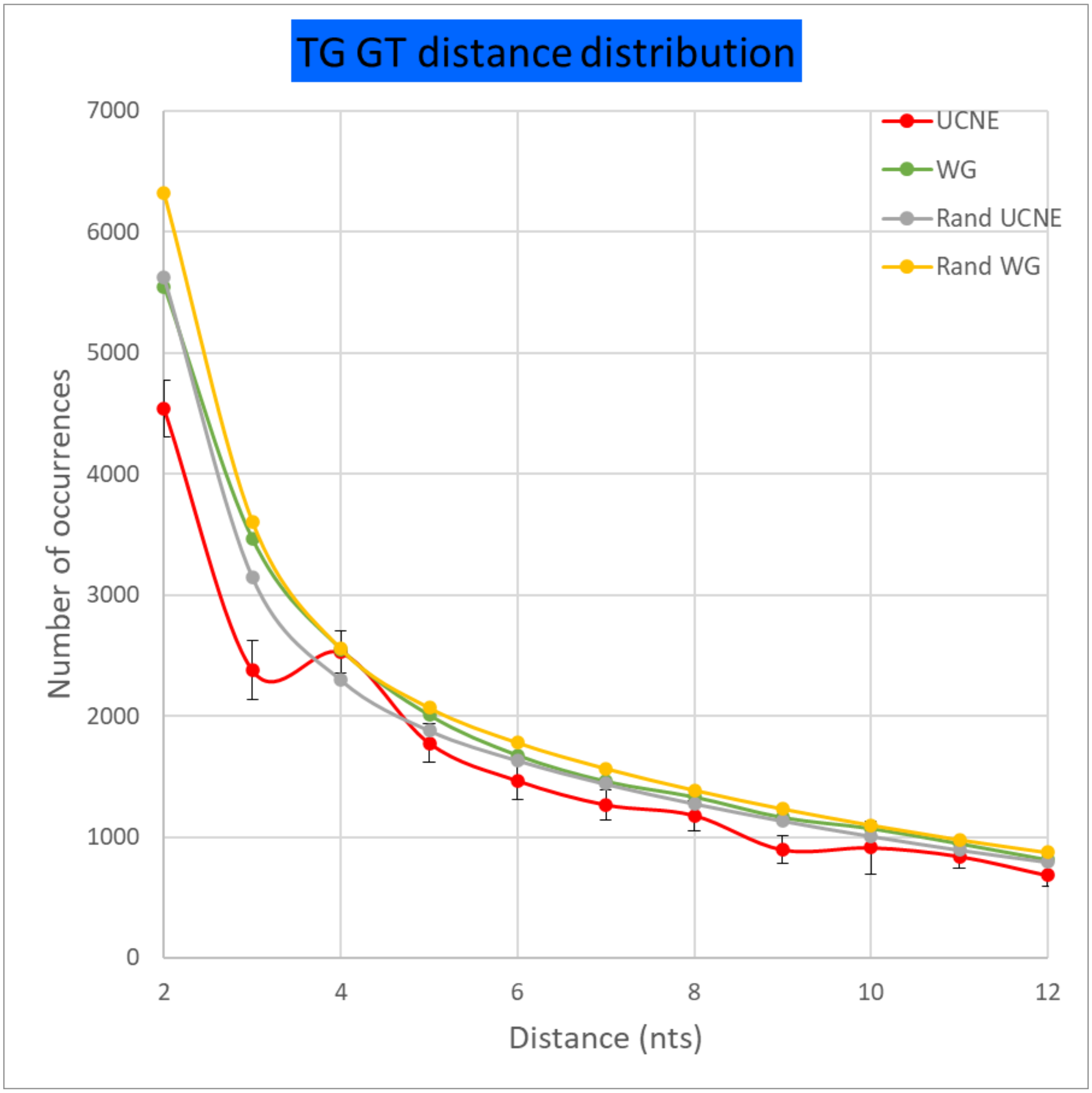
TG GT distance distribution
Number of occurrences
Distance (nts)
UCNE
WG
Rand UCNE
Rand WG
0
1000
2000
3000
4000
5000
6000
7000
2
4
6
8
10
12

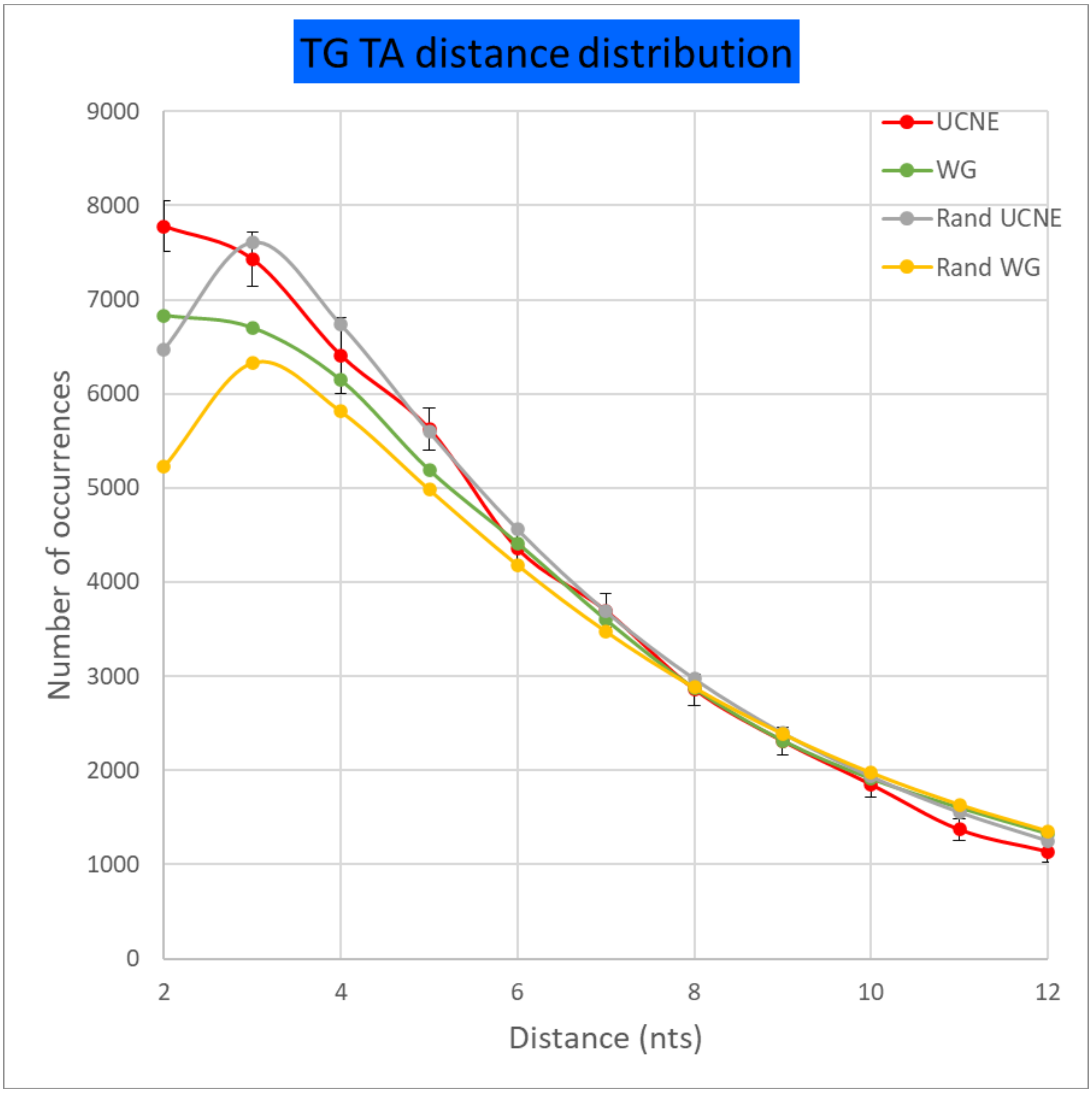
TG TA distance distribution
UCNE
WG
Rand UCNE
Rand WG
Number of occurrences
9000
8000
7000
6000
5000
4000
3000
2000
1000
0
2
4
6
8
10
12
Distance (nts)

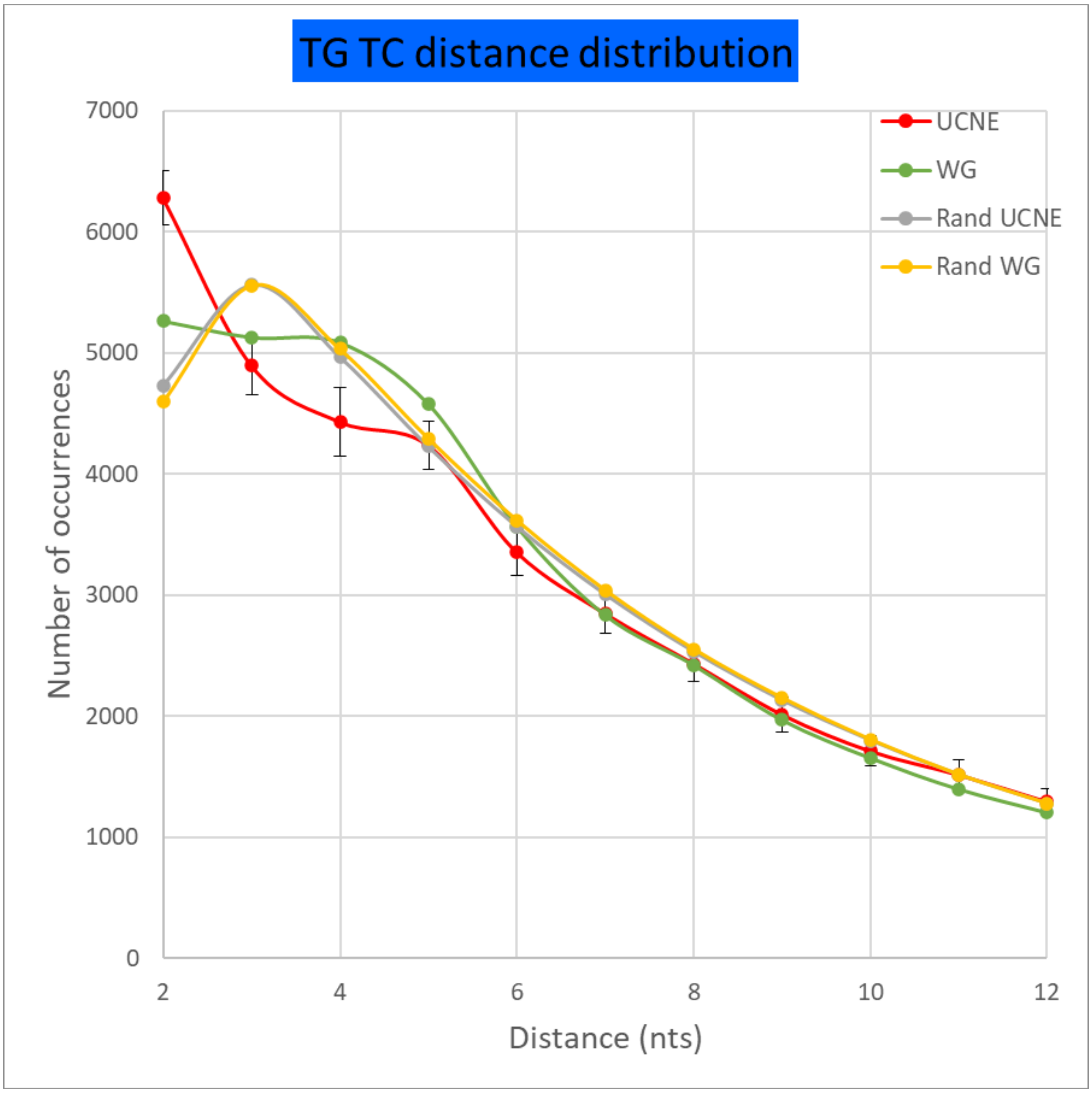
TG TC distance distribution
7000
6000
5000
4000
3000
2000
1000
0
Number of occurrences
2
4
6
8
10
12
Distance (nts)
UCNE
WG
Rand UCNE
Rand WG

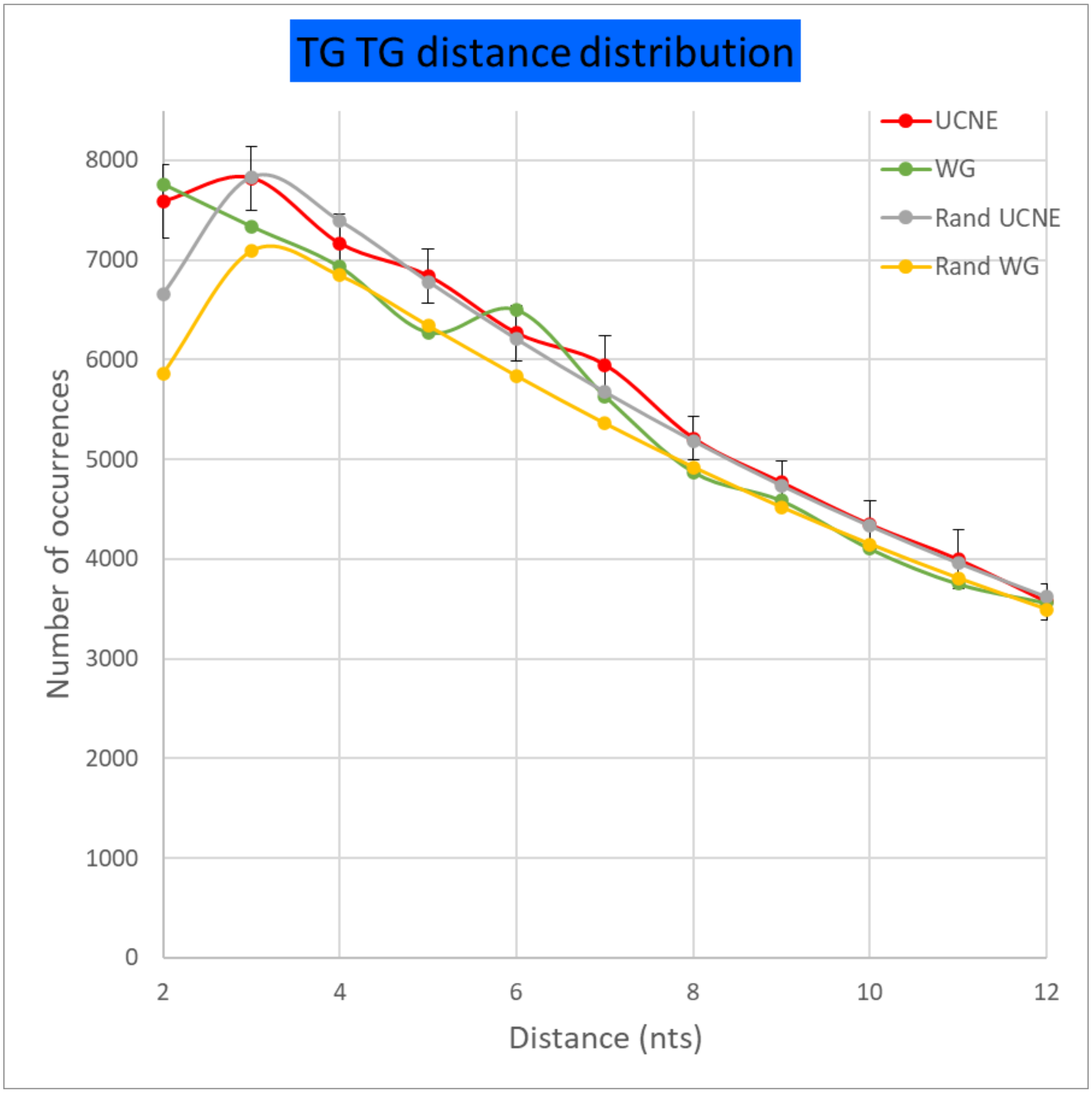

TG TG distance distribution
UCNE
WG
Rand UCNE
Rand WG
Number of occurrences
0
1000
2000
3000
4000
5000
6000
7000
8000
2
4
6
8
10
12
Distance (nts)

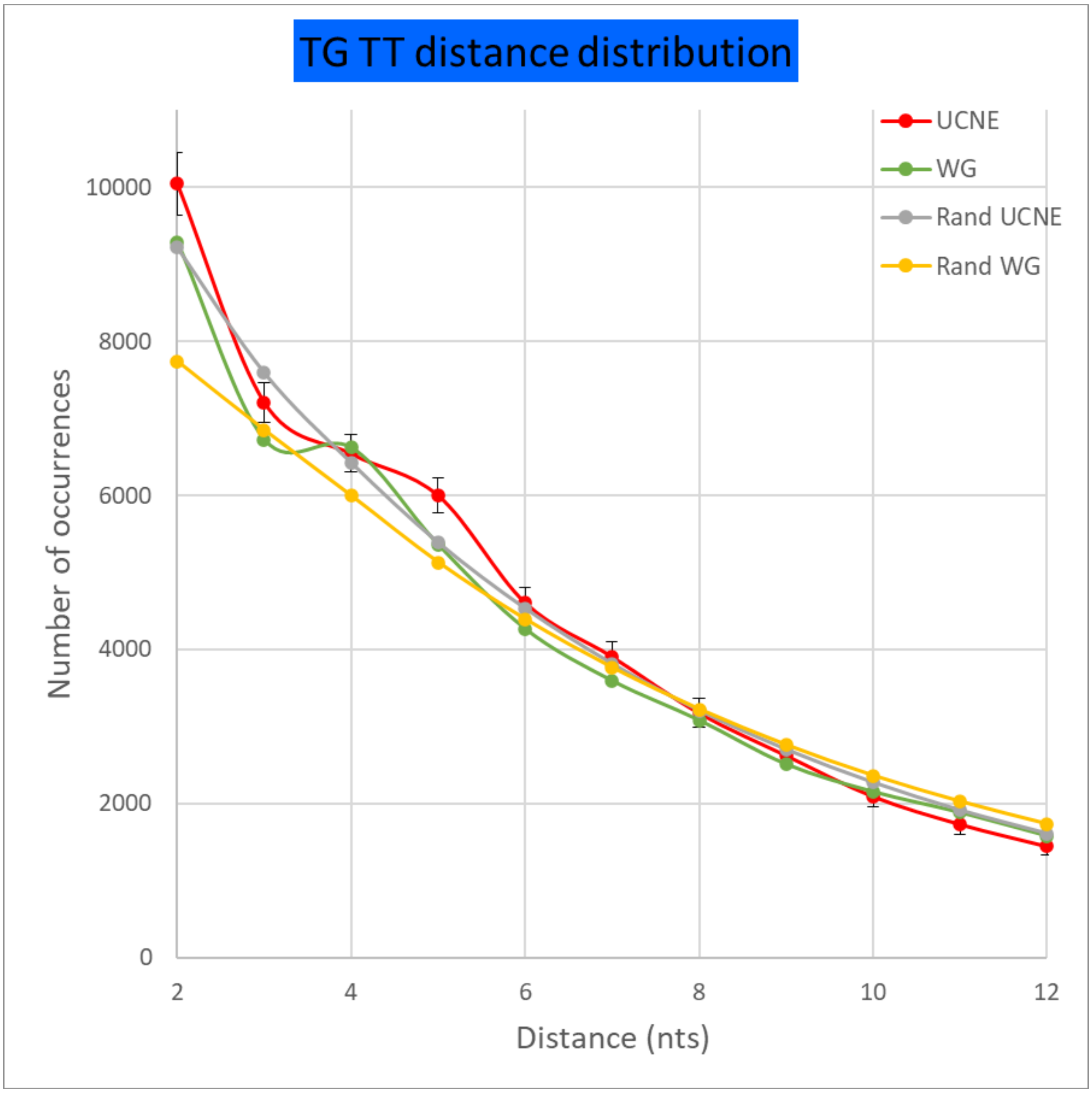

TG TT distance distribution
UCNE
WG
Rand UCNE
Rand WG
10000
8000
6000
4000
2000
0
2
4
6
8
10
12
Number of occurrences
Distance (nts)

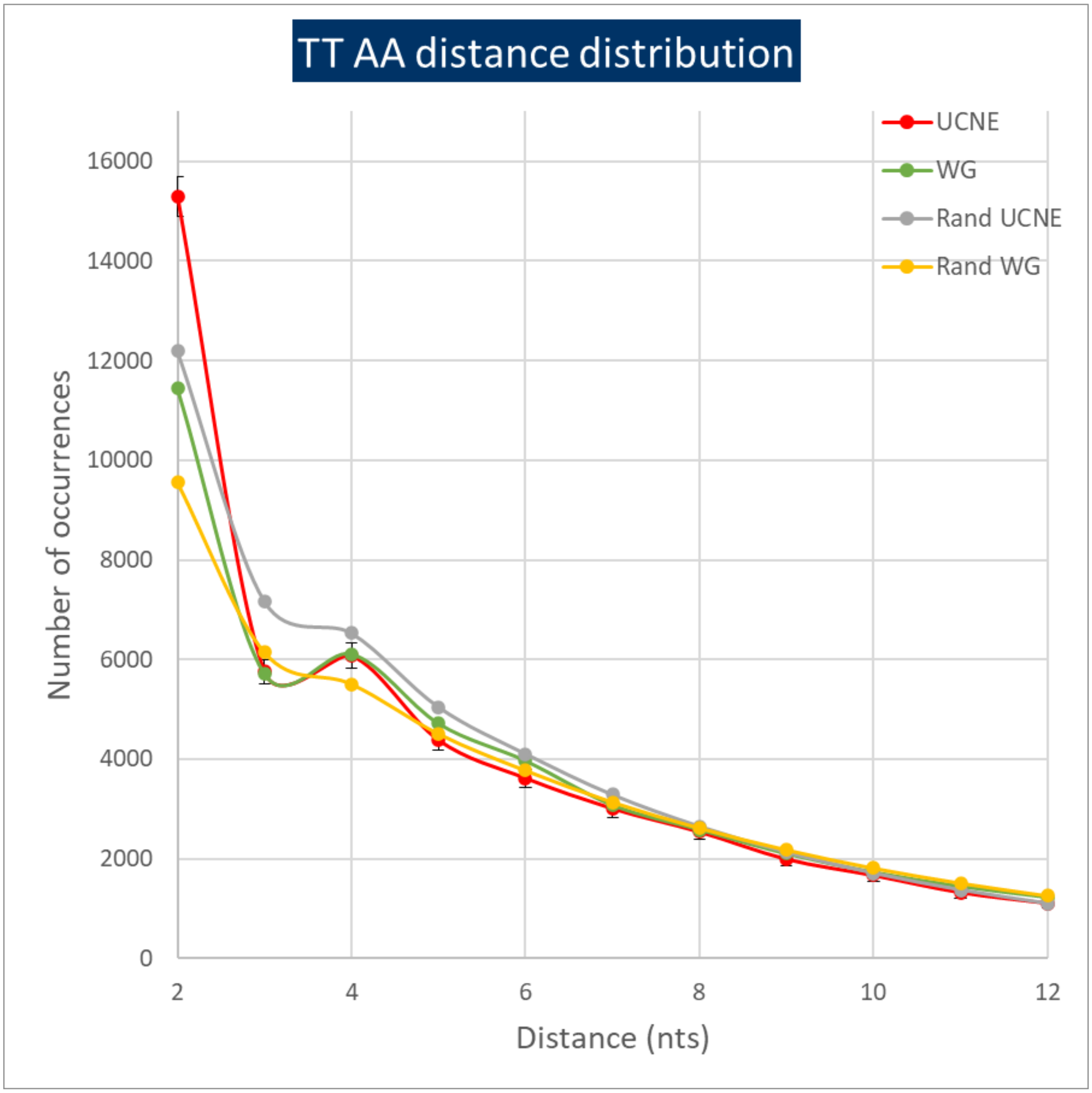

TT AA distance distribution
UCNE
WG
Rand UCNE
Rand WG
Number of occurrences
0
2000
4000
6000
8000
10000
12000
14000
16000
2
4
6
8
10
12
Distance (nts)

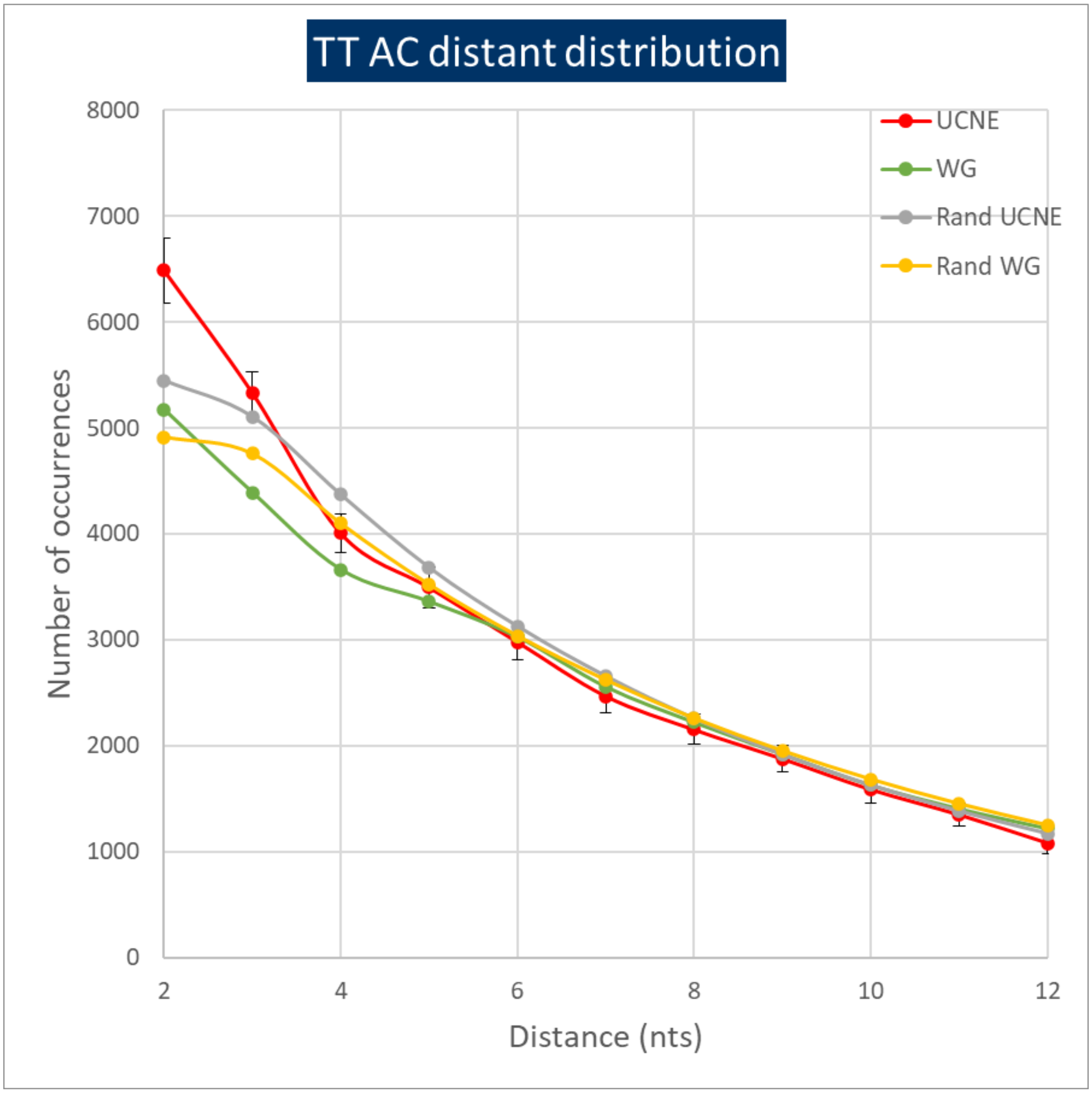

TT AC distant distribution
UCNE
WG
Rand UCNE
Rand WG
Number of occurrences
8000
7000
6000
5000
4000
3000
2000
1000
0
2
4
6
8
10
12
Distance (nts)

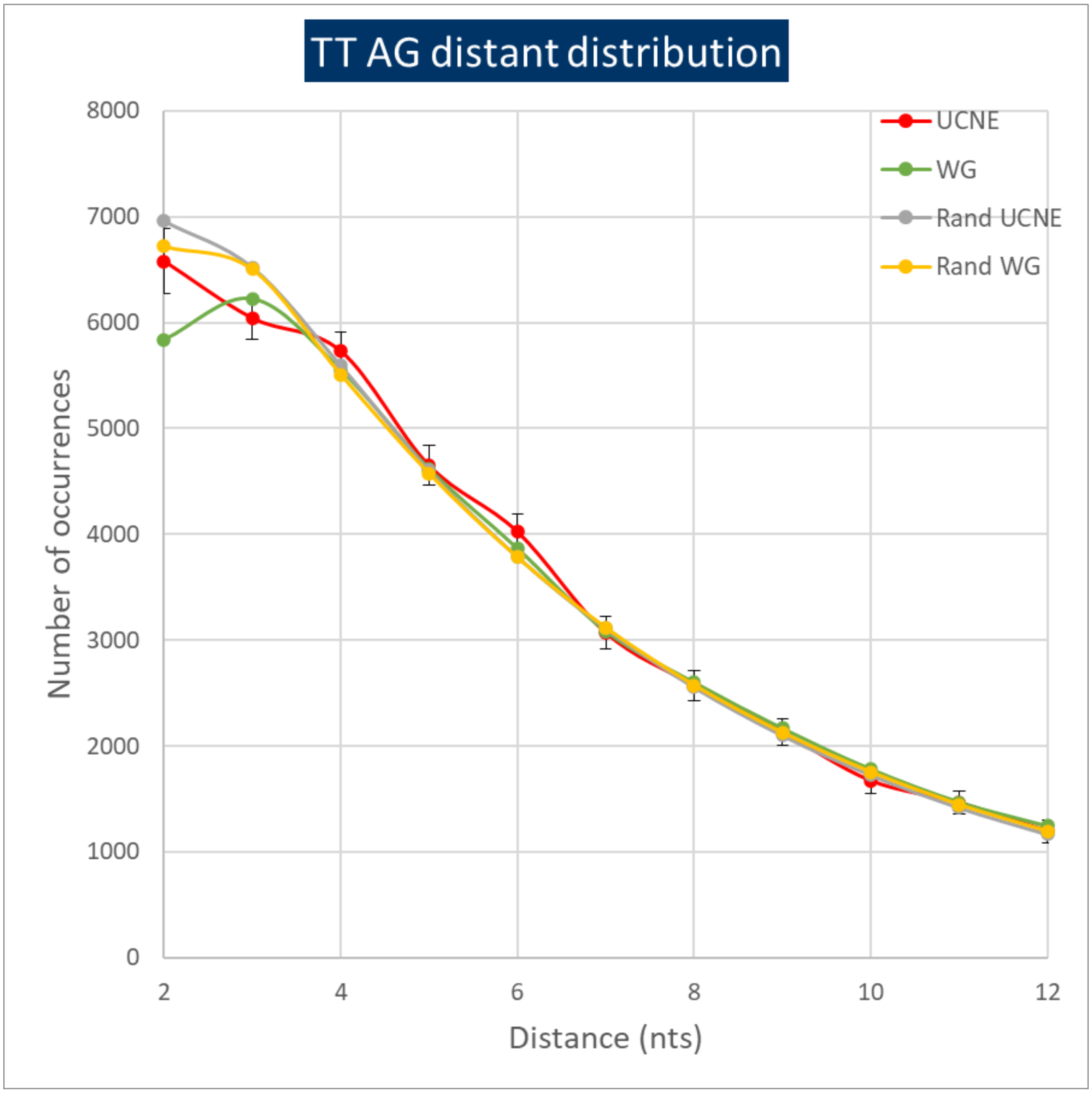
TT AG distant distribution
8000
7000
6000
5000
4000
3000
2000
1000
0
Number of occurrences
2
4
6
8
10
12
Distance (nts)
UCNE
WG
Rand UCNE
Rand WG

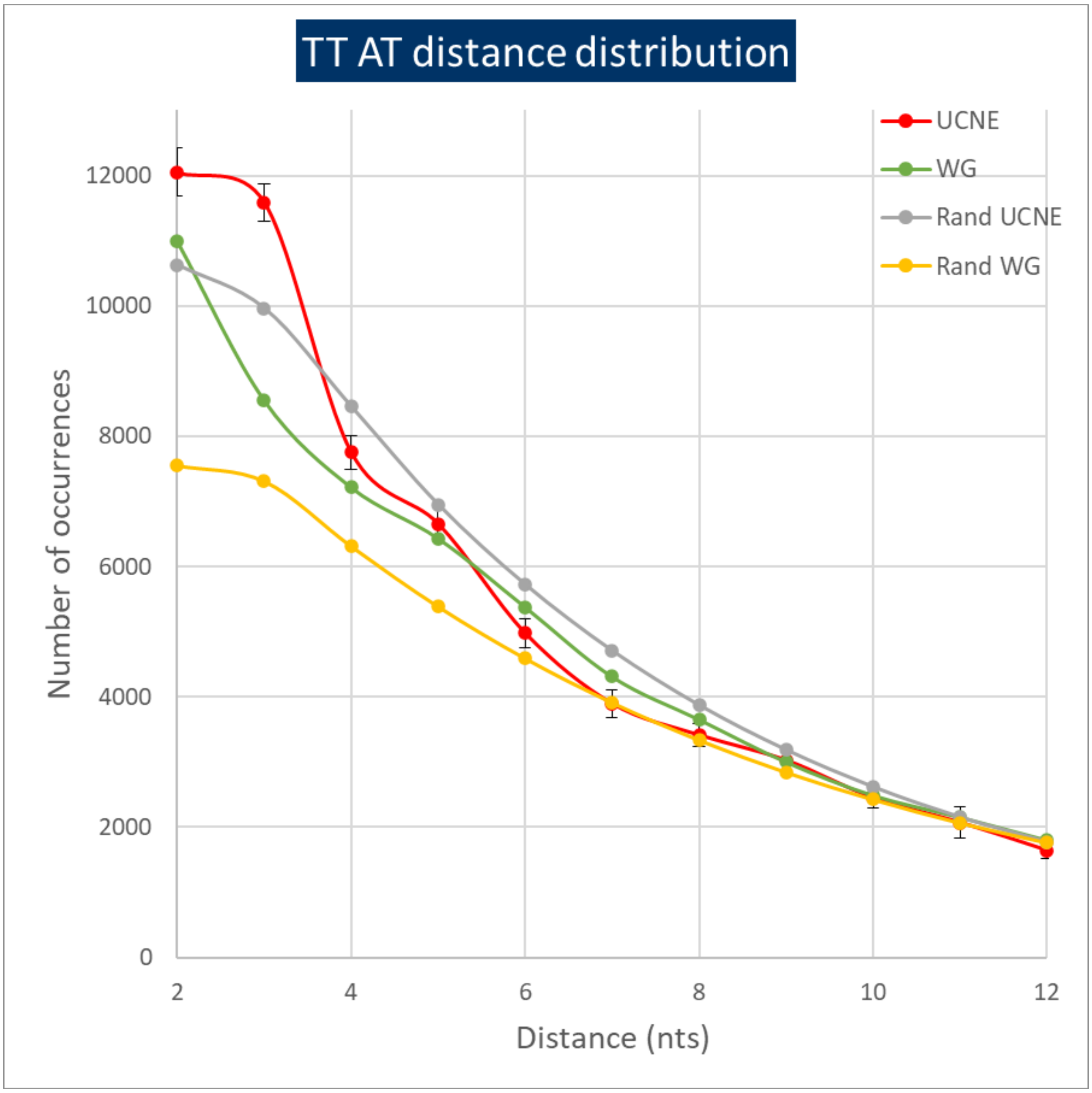

TT AT distance distribution
UCNE
WG
Rand UCNE
Rand WG
Number of occurrences
12000
10000
8000
6000
4000
2000
0
2
4
6
8
10
12
Distance (nts)

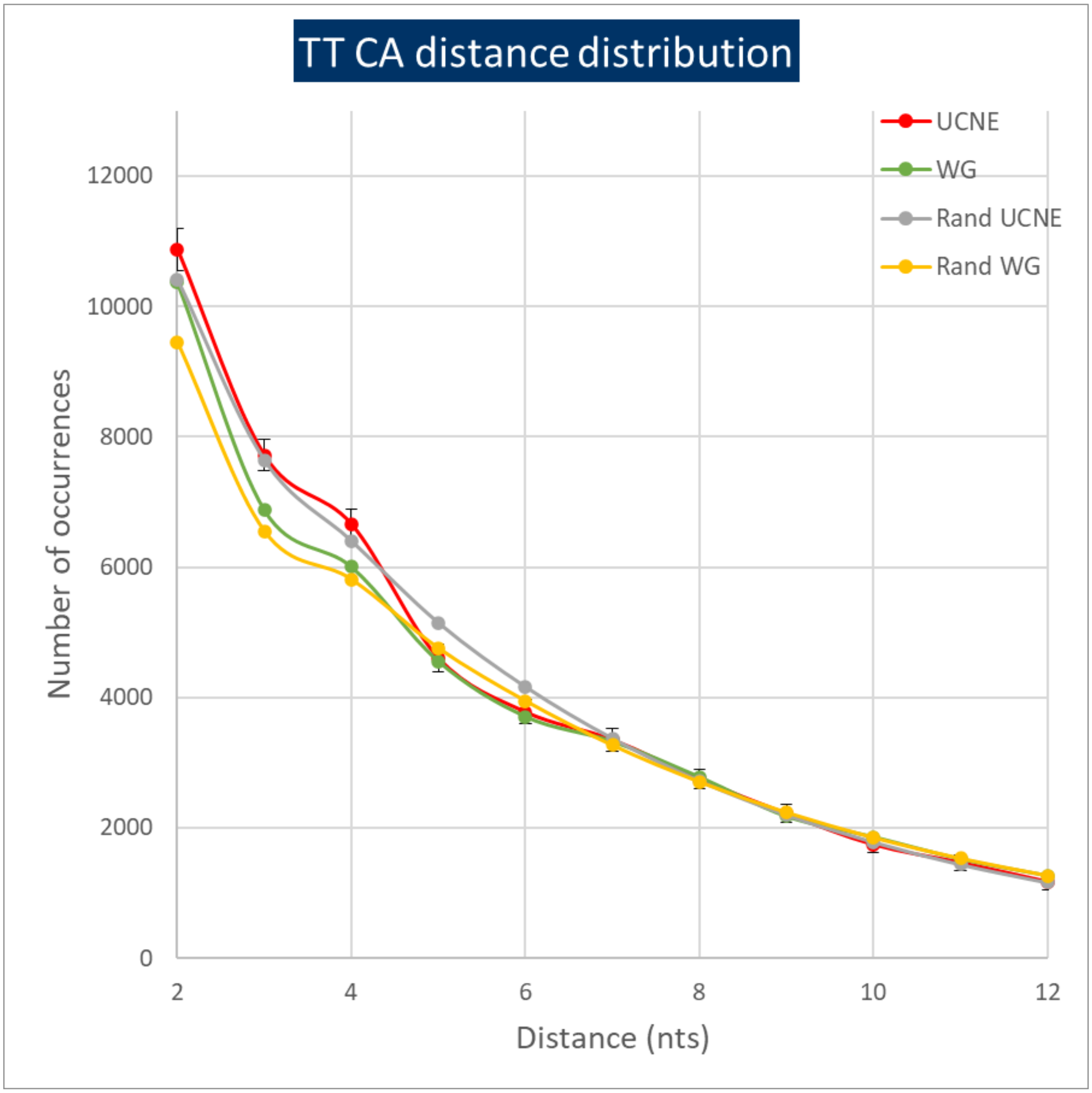
TT CA distance distribution
UCNE
WG
Rand UCNE
Rand WG
Number of occurrences
0
2000
4000
6000
8000
10000
12000
2
4
6
8
10
12
Distance (nts)

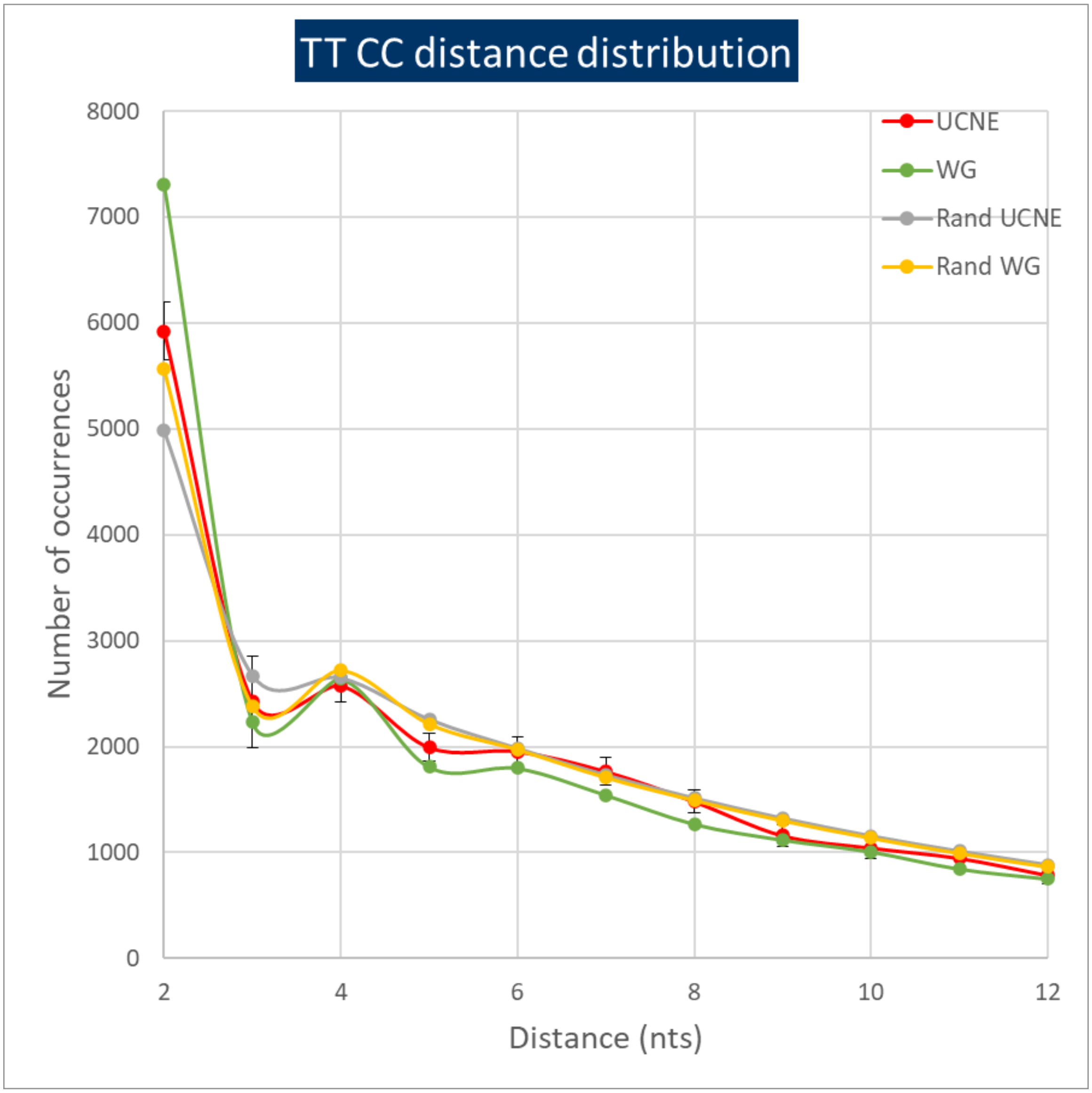
TT CC distance distribution
8000
7000
6000
5000
4000
3000
2000
1000
0
Number of occurrences
2
4
6
8
10
12
Distance (nts)
UCNE
WG
Rand UCNE
Rand WG

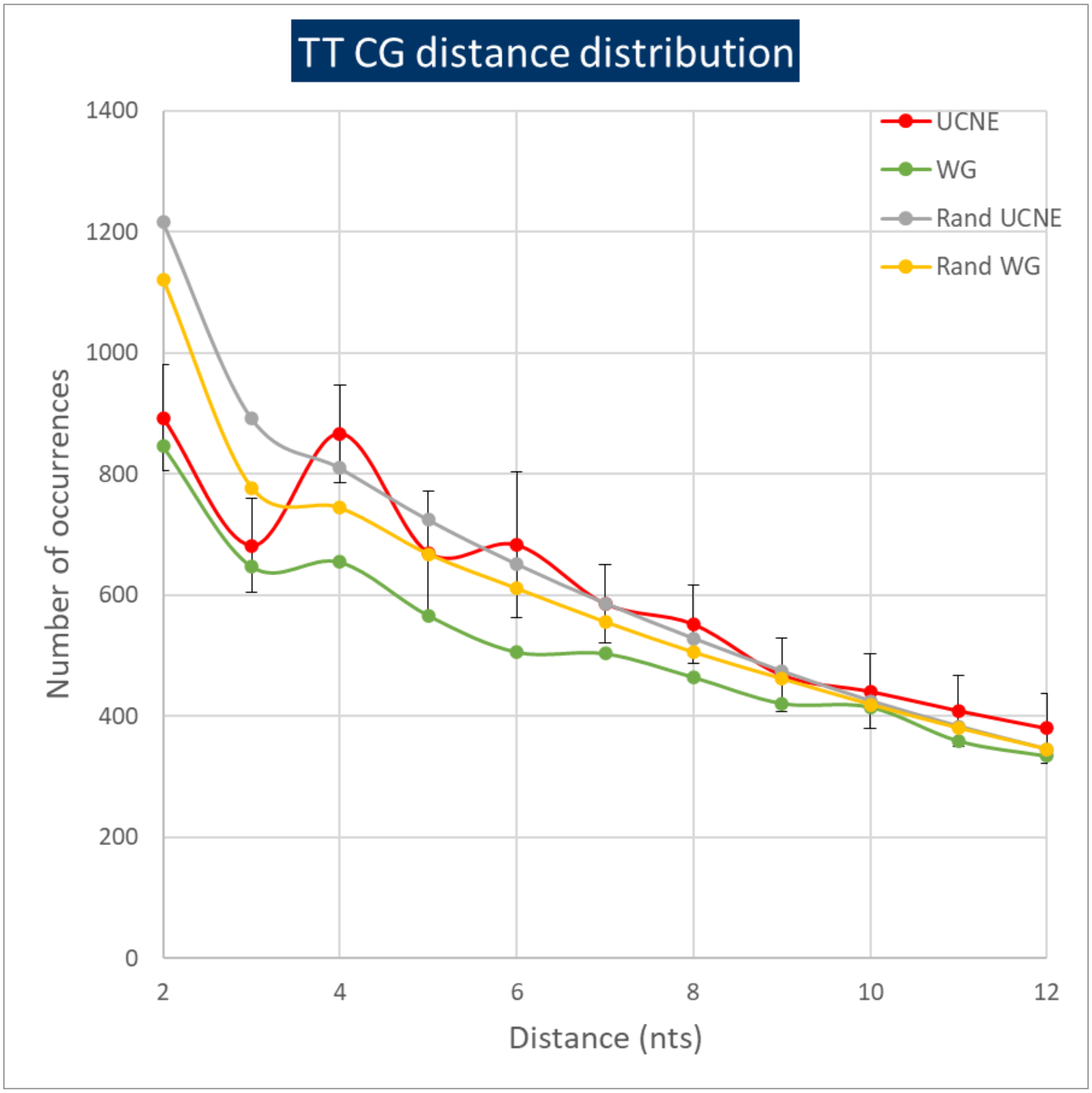
TT CG distance distribution
Number of occurrences
Distance (nts)
UCNE
WG
Rand UCNE
Rand WG
0
200
400
600
800
1000
1200
1400
2
4
6
8
10
12

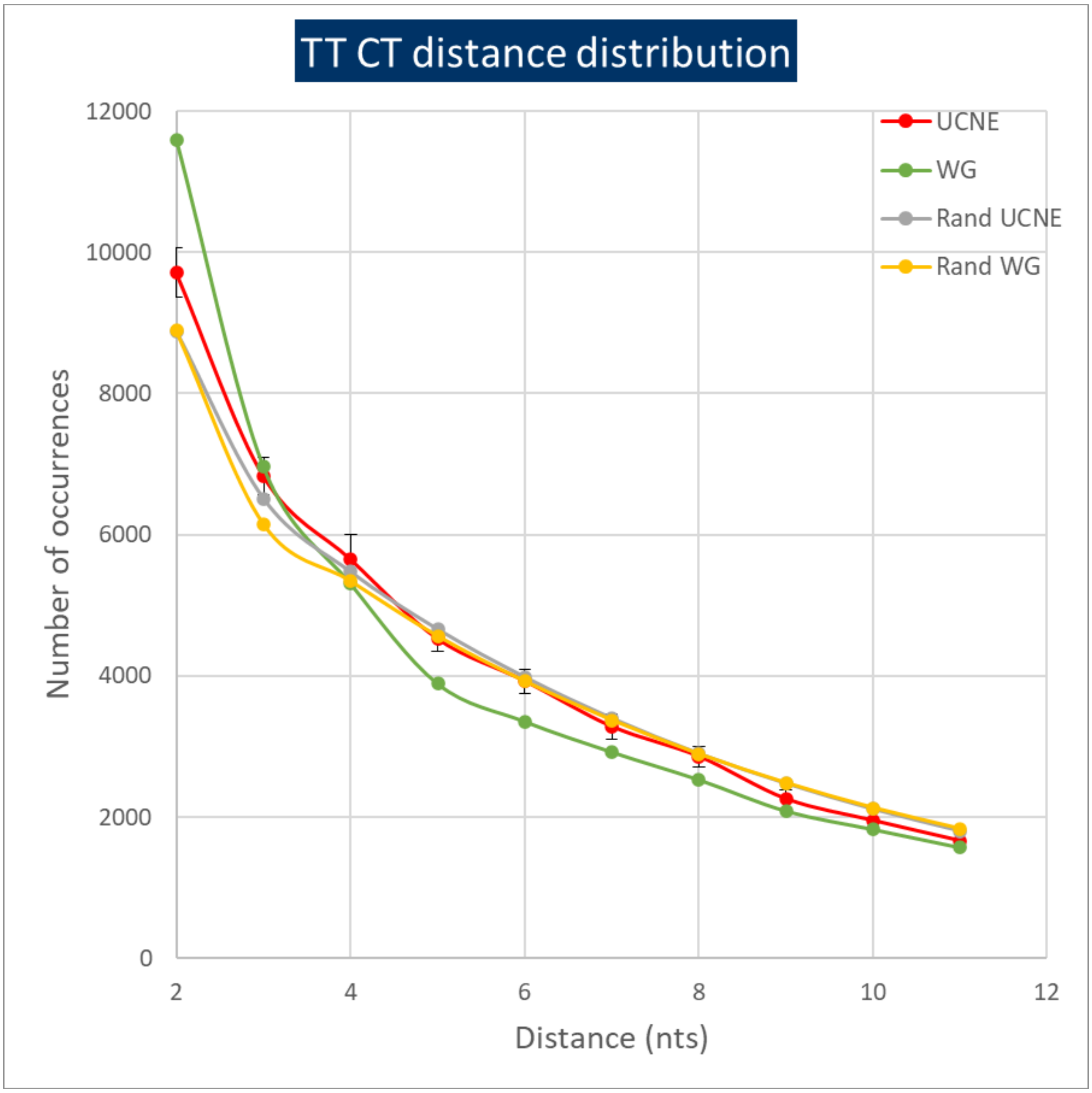
TT CT distance distribution
UCNE
WG
Rand UCNE
Rand WG
Number of occurrences
12000
10000
8000
6000
4000
2000
0
2
4
6
8
10
12
Distance (nts)

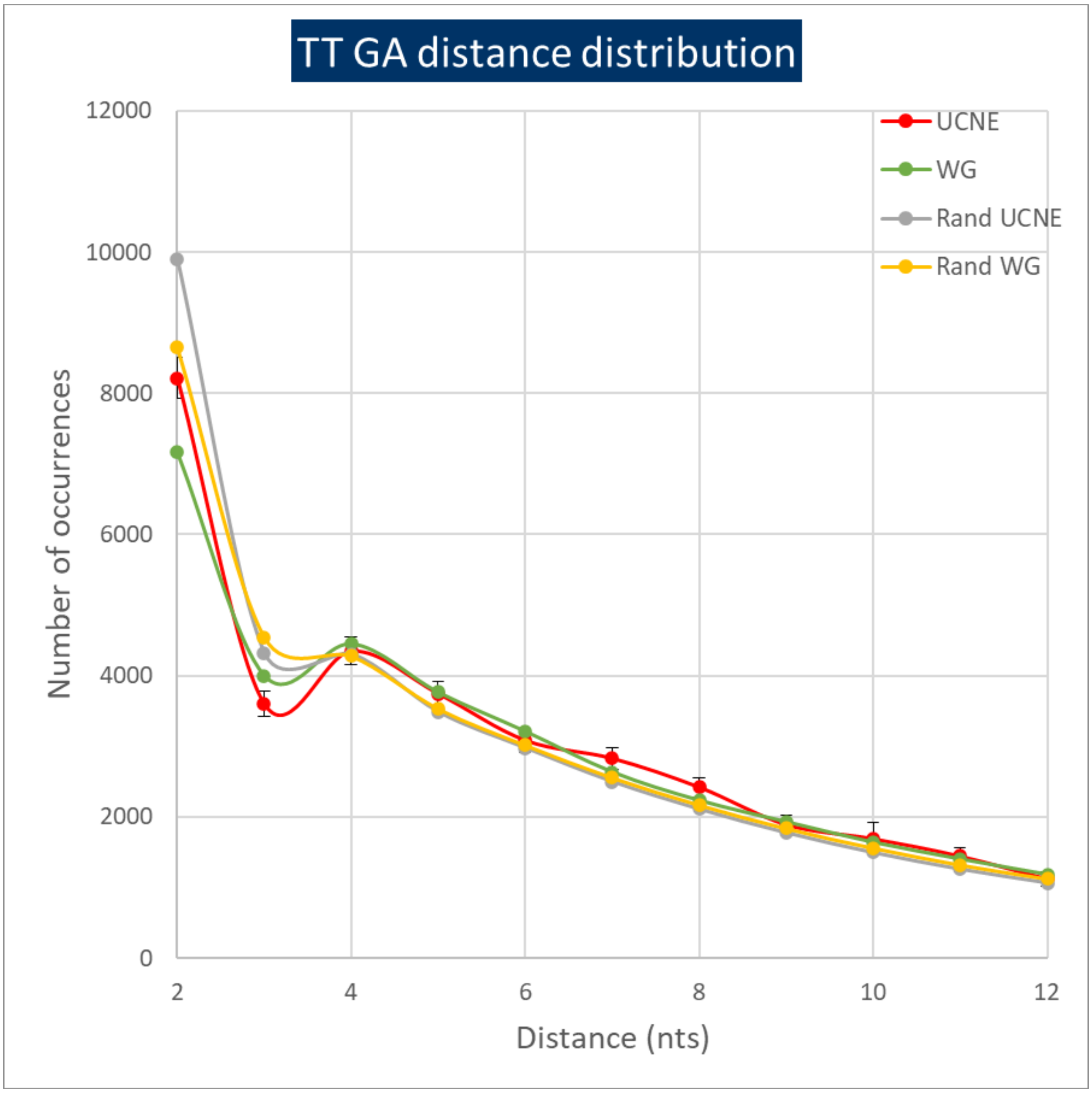
TT GA distance distribution
Number of occurrences
0
2000
4000
6000
8000
10000
12000
2
4
6
8
10
12
Distance (nts)
UCNE
WG
Rand UCNE
Rand WG

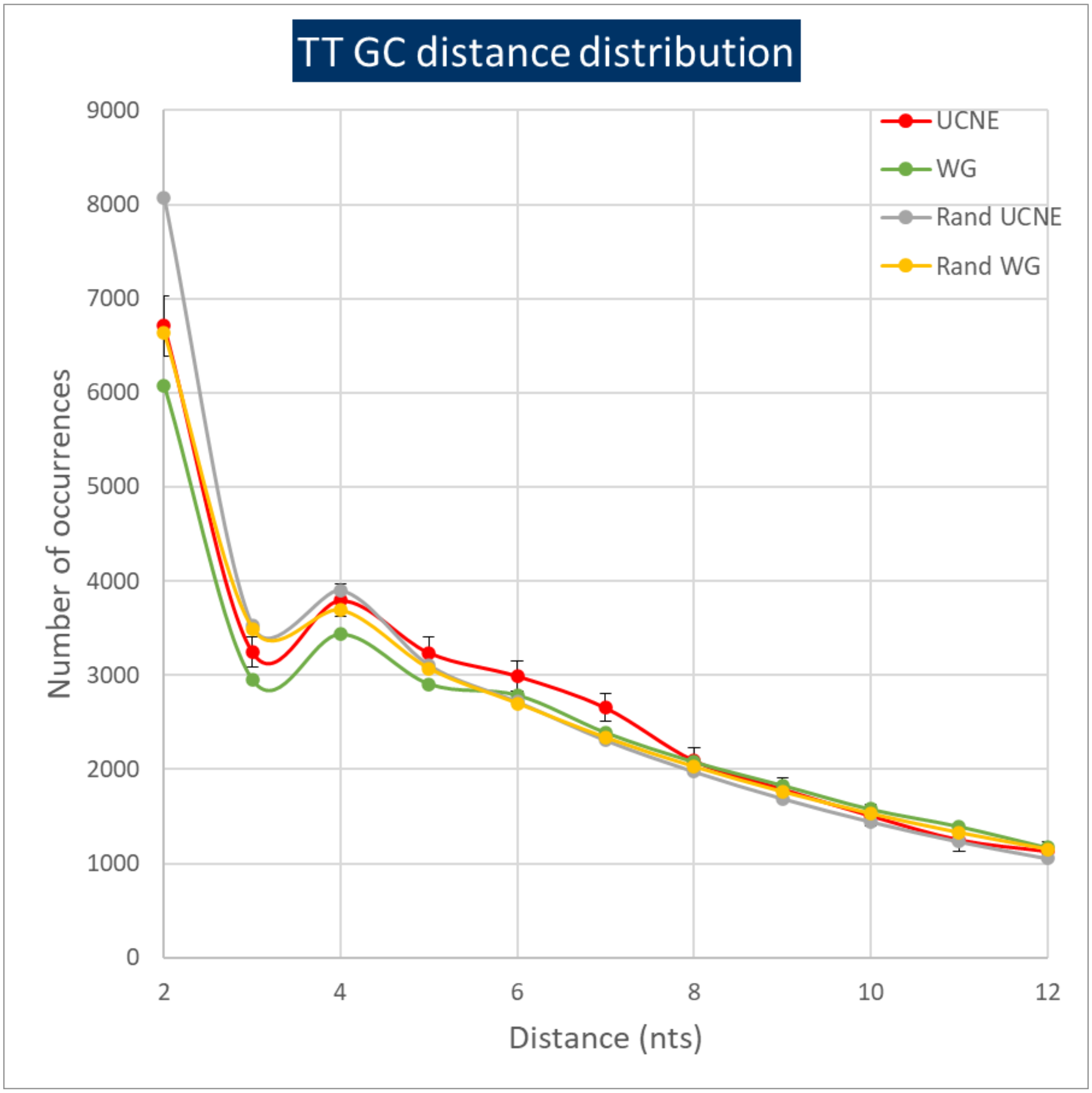

TT GC distance distribution
UCNE
WG
Rand UCNE
Rand WG
Number of occurrences
9000
8000
7000
6000
5000
4000
3000
2000
1000
0
2
4
6
8
10
12
Distance (nts)

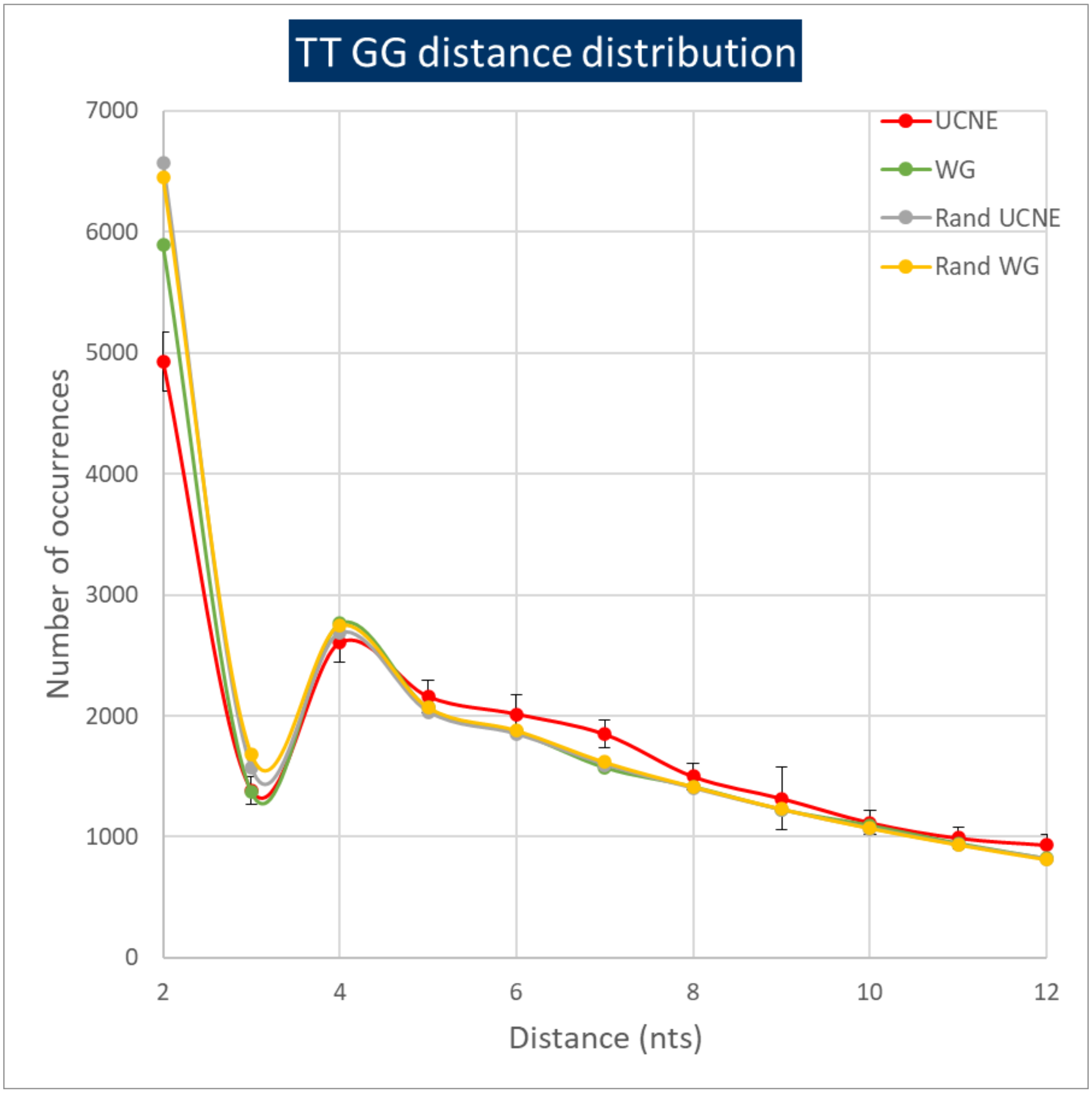
TT GG distance distribution
UCNE
WG
Rand UCNE
Rand WG
Number of occurrences
0
1000
2000
3000
4000
5000
6000
7000
2
4
6
8
10
12
Distance (nts)

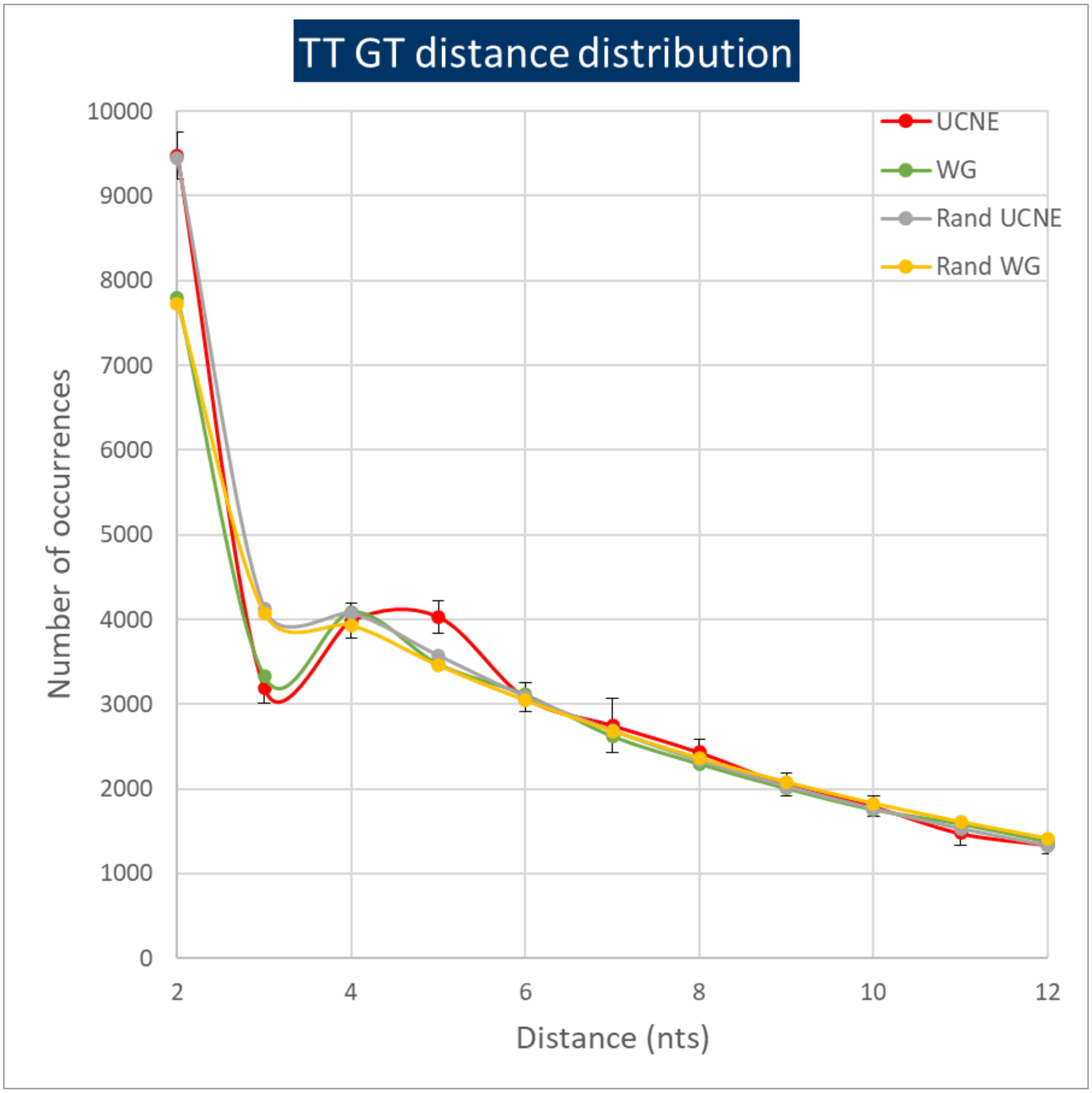

TT GT distance distribution
Number of occurrences
Distance (nts)
UCNE
WG
Rand UCNE
Rand WG
10000
9000
8000
7000
6000
5000
4000
3000
2000
1000
0
2
4
6
8
10
12

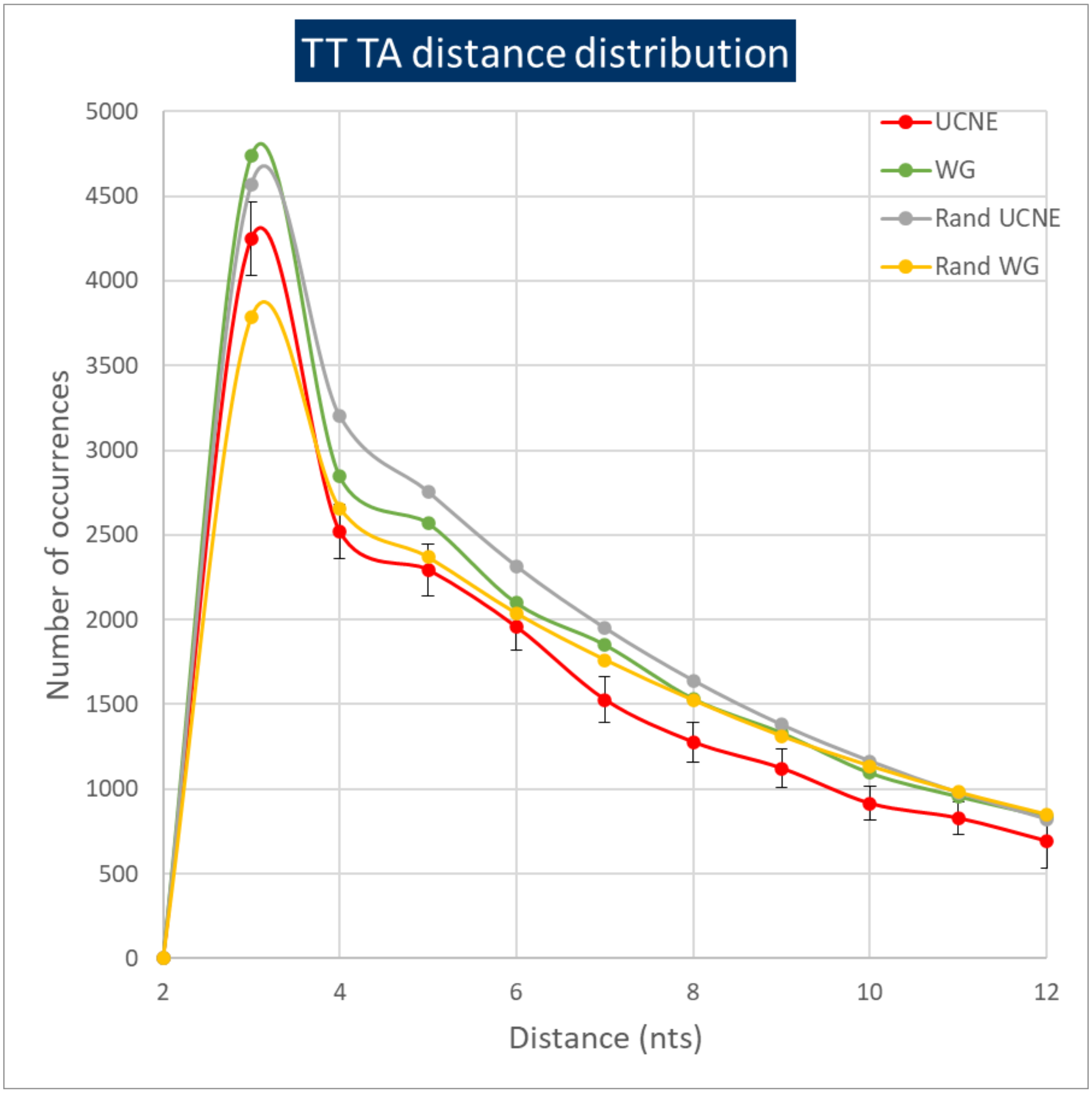
TT TA distance distribution
UCNE
WG
Rand UCNE
Rand WG
Number of occurrences
0
500
1000
1500
2000
2500
3000
3500
4000
4500
5000
2
4
6
8
10
12
Distance (nts)

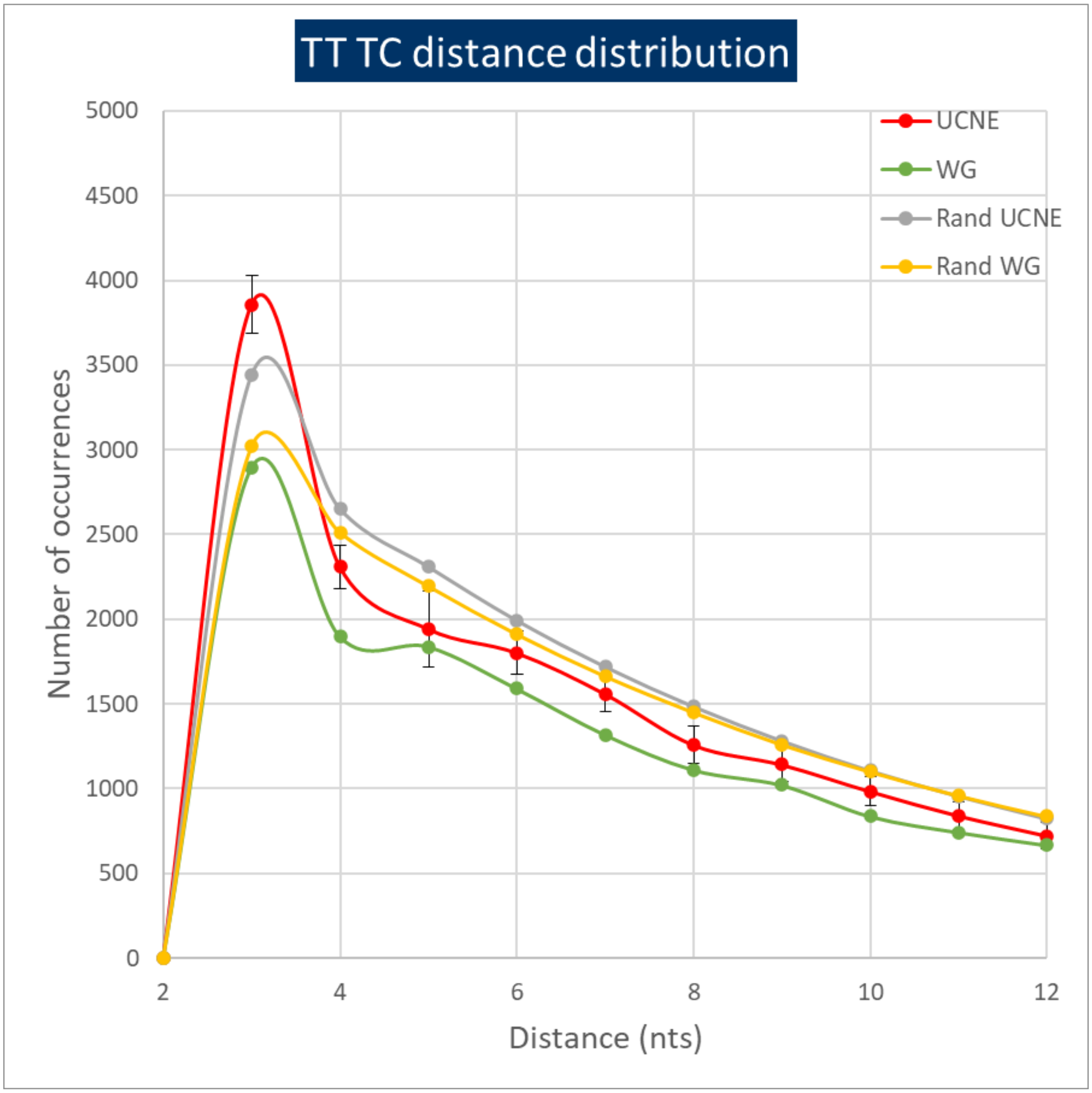

TT TC distance distribution
Number of occurrences
5000
4500
4000
3500
3000
2500
2000
1500
1000
500
0
2
4
6
8
10
12
Distance (nts)
UCNE
WG
Rand UCNE
Rand WG

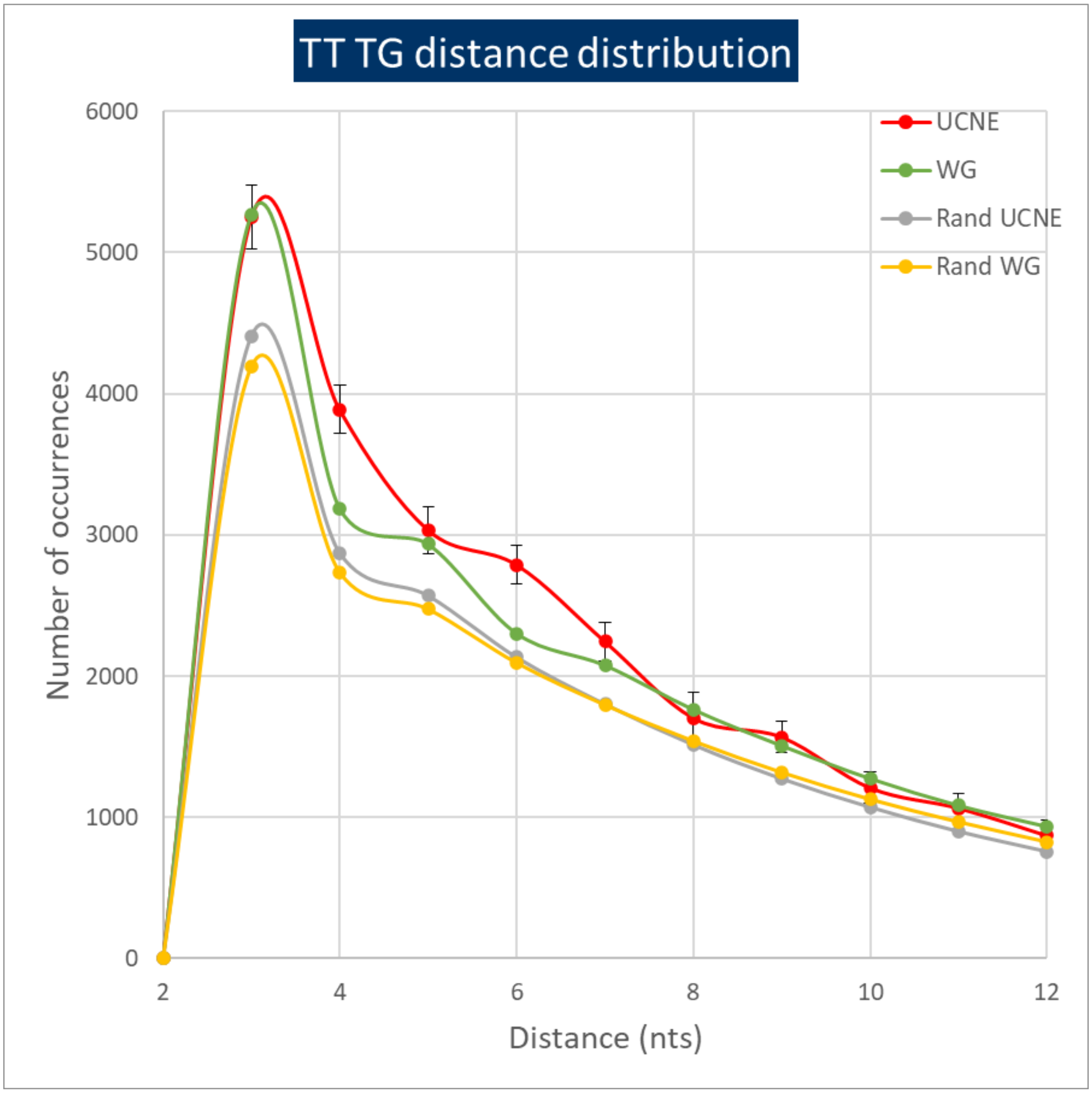
TT TG distance distribution
UCNE
WG
Rand UCNE
Rand WG
Number of occurrences
0
1000
2000
3000
4000
5000
6000
2
4
6
8
10
12
Distance (nts)

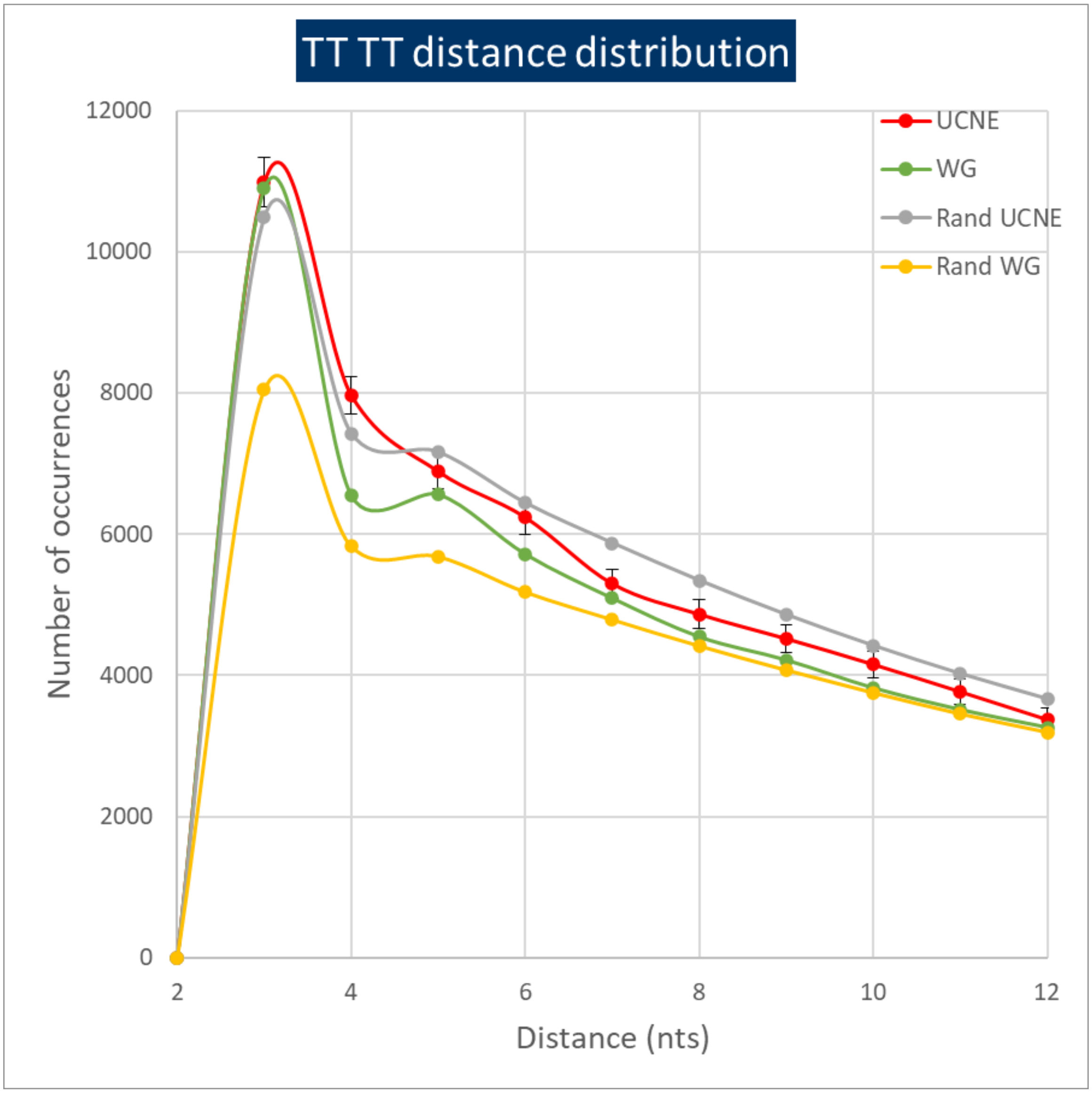
TT TT distance distribution
UCNE
WG
Rand UCNE
Rand WG
Number of occurrences
0
2000
4000
6000
8000
10000
12000
2
4
6
8
10
12
Distance (nts)
